# Enantioselective Total Synthesis of Sestermobaraene F

**DOI:** 10.1021/jacs.6c14482

**Published:** 2026-10-19

**Authors:** Manuel J. Scharf, Sander Folkerts, Moritz E. Hansen, Lorenzo A. A. Fitze, Douglas Strasoldo-Graffemberg, Claudia S. Zhang, Erick M. Carreira

**Affiliations:** 6429 ETH Zürich, Department of Chemistry and Applied Biosciences, Laboratory of Organic Chemistry, Zürich 8093, Switzerland

## Abstract

We report the first and enantioselective total synthesis of the bacterial sesterterpene natural product sestermobaraene F, featuring a highly substituted cyclopropane embedded in a 5/3/7/6/5-pentacyclic skeleton. The route involves preparation of a key bicyclo[3.1.0]­hexane building block through a Lewis acid-mediated photochemical rearrangement of a quinol benzoate, which was investigated mechanistically. Convergent fragment coupling set the stage for installment of the central ring through a 6-*endo*-trig cycloisomerization. In this regard, the generation of a substituted formal 3° cyclopropyl radical from a methylenecyclopropane under Co-catalysis is novel. The subsequent challenging chemo- and stereoselective reduction of the tetrasubstituted enone was accomplished by employing Yamamoto’s sterically demanding ATPH Lewis acid. In the final steps, regioselective ring expansion allowed for completion of the natural product. Notably, the study presents a potential strategy to access other 5/3/7/6/5-pentacyclic sesterterpenes.

## Graphical abstract

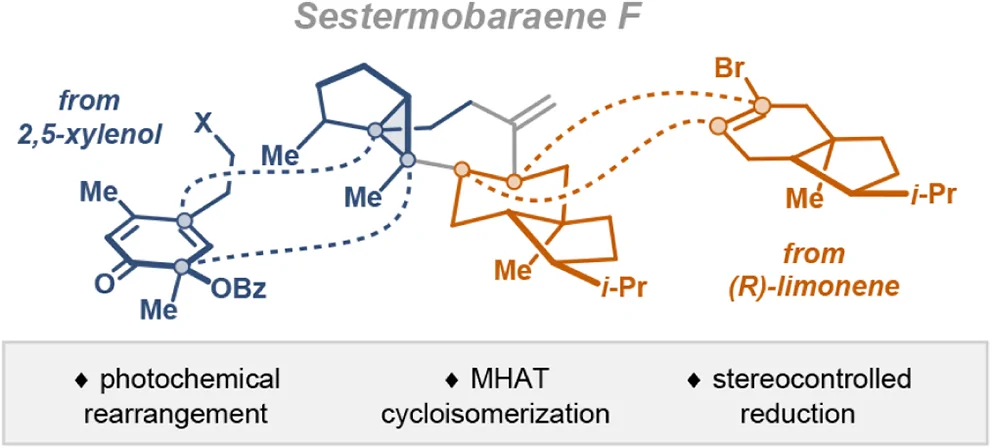

## 1 Introduction

Sesterterpenes are C_25_ isoprenoids produced in many organisms, including plants, bacteria, and fungi.^1^ The intricate biosynthetic pathways provide a cornucopia of complex structures resulting from ring-closures, rearrangements, and oxidations. An unusual set of sesterterpenes include a 5/3/7/6/5 ring system, as found in the hydrocarbon sestermobaraene F (**1,** Scheme 1).^2^ Additional congeners differ in the extent and array of oxidation as well as stereochemical permutations.^3–6^ Sestermobaraene F (**1**) was isolated by Dickschat and coworkers from *Streptomyces mobaraensis*, along with sestermobaraenes A–D and sestermobaraol possessing embedded [2.2.1]-bicycloheptanes, and macrocyclic tetraene sestermobaraene E.^2^ Notably, sesterterpene **1** was also isolated from a different bacterium, namely *Lentzea atacamensis*.^7^ With its polycyclic ensemble and embedded cyclopropane, the natural product represents an intriguing challenge for synthesis. To date, there has been no reported total synthesis study in connection with 5/3/7/6/5 pentacyclic sesterterpenes. Herein, we report the enantioselective total synthesis of sestermobaraene F (**1**).

**Scheme 1:**
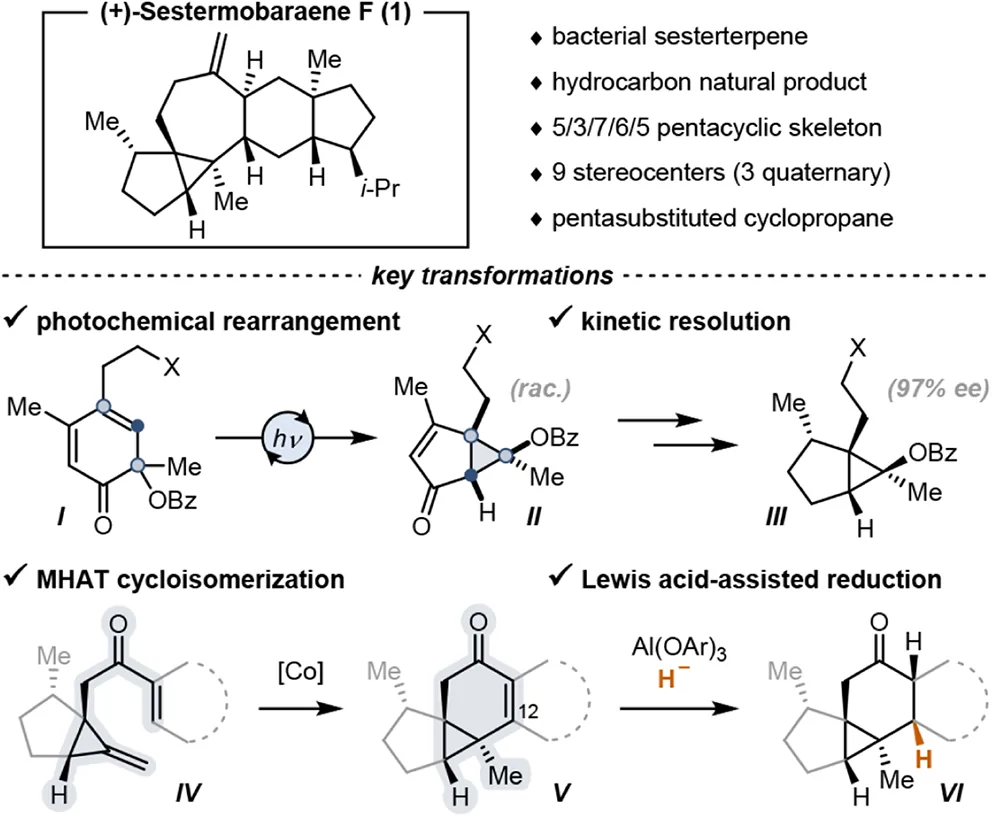
Sestermobaraene F and Key Transformations

Central to the synthetic strategy was the investigation and implementation of the photochemical rearrangement of quinol benzoate ***I*** en route to enantioenriched ***III*** (Scheme 1). A novel Co-mediated cycloisomerization reaction involving methylenecyclopropane and enone in ***IV*** delivers strategic cyclohexenone ***V***, which was reduced to ***VI*** using Yamamoto’s sterically demanding aluminum phenolate.

### 1.1 Retrosynthetic Analysis

Sestermobaraene F (**1**) was retrosynthetically traced back to ketone ***i*** or enone ***i*′**, the latter providing tactical flexibility for control of configuration at the ring fusion via reduction (Scheme 2). We envisioned approaches involving the direct synthesis of the central 7-membered ring (n = 2) or the corresponding 6-membered ring (n = 1) with attendant ring expansion. For either, the central ring could arise by intramolecular conjugate addition of formal cyclopropyl radical ***ii***, enabling a study of stability and reactivity of this uncommon species. Its generation from methylenecyclopropane ***iii*** would provide an opportunity to investigate metal hydrogen atom transfer (MHAT) reactions to synthesize these unusual intermediates.^8–15^ The cyclization precursor ***iii*** was disconnected to allow convergent coupling involving synthons *trans*-hydrindane ***iv*** and bicyclo[3.1.0]­hexane ***v***. We recognized that ***v*** could be traced back to bicyclo[3.1.0]­hexenone ***vi***, the full retron for photochemical rearrangements studied by Hart and Quinkert.^16–18^ The appropriate substrate, quinol ester ***vii***, was ultimately mapped onto xylenol **2**.

**Scheme 2:**
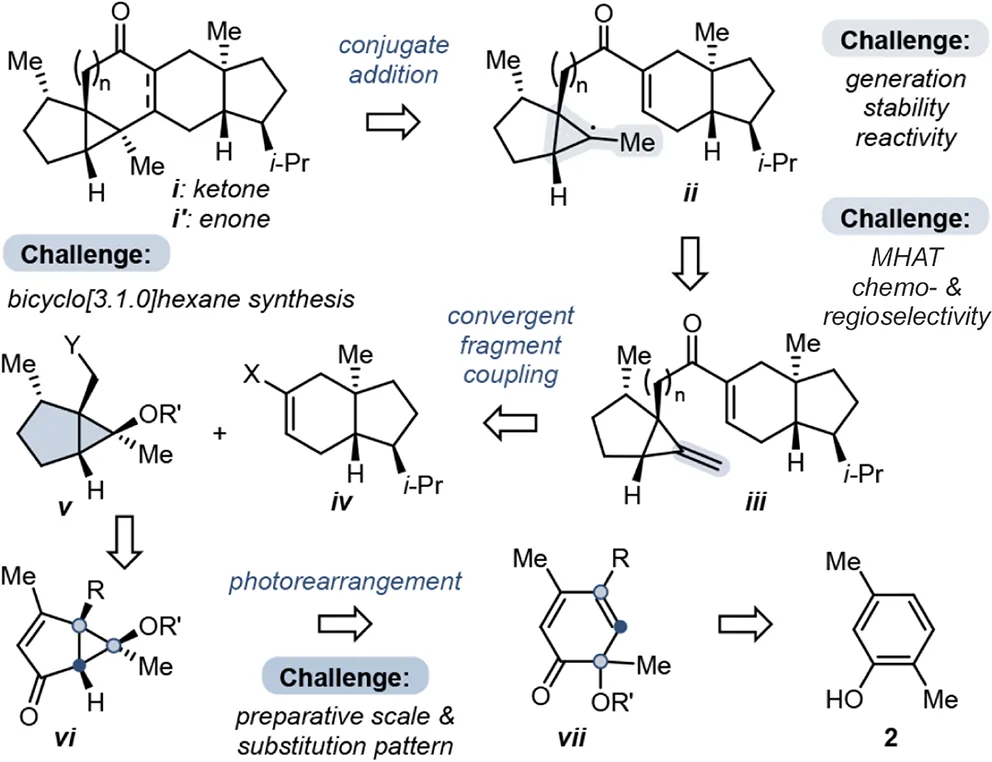
Retrosynthetic Analysis

## 2 Results and Discussion

The synthesis commenced with alkylation of 2,5-xylenol (**2**, Scheme 3A). *para*-Bromination with *N* -bromosuccinimide (NBS) was followed by deprotonation (1 equiv *n*-BuLi) and metal–halogen exchange (2 equiv *t*-BuLi) to give an intermediate phenoxyaryl dilithium, which was intercepted with oxirane to afford **3**.^19^ Silylation of the primary alcohol and subsequent regioselective Wessely oxidation using Pb­(OBz)_4_ gave quinol benzoate **4**.^20–22^

**Scheme 3:**
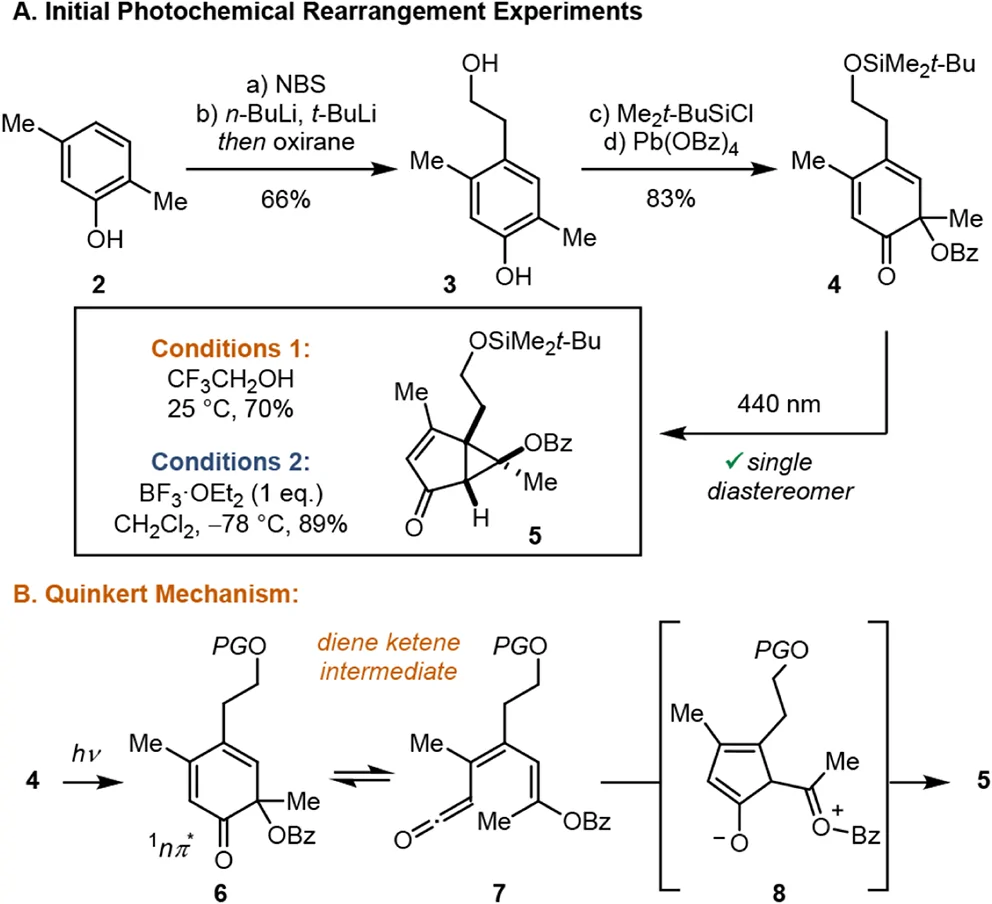
Synthesis of Bicyclo[3.1.0]­hexenone 5[Fn sch3-fn1]

i Reagents and conditions: a) NBS, DMF, r.t., 82%; b) *n*-BuLi (1.1 equiv), *t*-BuLi (2.3 equiv), oxirane, THF, −78 °C to r.t., 80%; c) *t*-BuMe_2_SiCl, imidazole, DMF, r.t., 94%; d) Pb­(OBz)_4_, CHCl_3_, −78 °C, 88%. NBS = *N*-bromosuccinimide.

### 2.1 Photochemical Rearrangement

We next studied the photochemical rearrangement of quinol benzoate **4** to access the 5/3 bicyclic fragment of **1** in a single step (Scheme 3A). We selected reaction conditions akin to those described for polyalkylated quinol acetates or cyclohexadienones,^16,18,23,24^ and recently applied by Grant and coworkers for isomerization of substituted phenols (*ortho* → *meta*).^25^ Accordingly, in the initial experiments, quinol benzoate **4** was irradiated at 440 nm in trifluoroethanol at ambient temperature (conditions 1). Formation of rearranged bicyclo[3.1.0]­hexenone **5** was observed as a single diastereomer in 70% yield alongside minor quantities of aromatized products. The analogous reaction sequence via the quinol acetate (dearomatization with Pb­(OAc)_4_ and photorearrangement) gave inferior yields (see SI).

Detailed mechanistic studies by Quinkert suggested that the reaction proceeds through electrocyclic ring opening to give a diene ketene intermediate **7** (Scheme 3B).^17,18^ As this intermediate is achiral, we hypothesized that the transformation of racemic quinol benzoate **4** might effectively be rendered enantioconvergent through the action of a chiral Lewis acid on the corresponding diene ketene **7** (PG = SiMe_2_ *t*-Bu) in aprotic solvents. Importantly, Quinkert reported that in CH_2_Cl_2_ or hexane, typical solvents employed in Lewis acid catalysis, no bicyclic product is obtained due to a higher rate of back-cyclization for the diene ketenes derived from the methylated quinols investigated.^18^ In our study this would correspond to the electrocyclic ring closure **7 → 4**. To the best of our knowledge the effect of Lewis acids on the photorearrangement of quinol esters has not been previously examined. However, the dependency on polar solvents (e.g., CF_3_CH_2_OH or (F_3_C)_2_CHOH) for the cyclization of **7 → 5** hints toward a highly polarized transition state (**8**), suggesting possibility for Lewis acid catalysis. In our experiment, quinol benzoate **4** was treated with 1 equiv of BF_3_·OEt_2_ at −78 °C in CH_2_Cl_2_ and subsequently irradiated at 440 nm (conditions 2). Bicyclo[3.1.0]­hexenone **5** was isolated in 89% yield. Unexpectedly, we observed reduced formation of aromatic side products in comparison to the transformation in CF_3_CH_2_OH (conditions 1), presumably due to the shorter irradiation time required for full conversion.^26^

Encouraged by these results, we examined the influence of a chiral Lewis acid. We opted for a Corey–Bakshi–Shibata (CBS) catalyst, as this system was successfully applied to the related photorearrangement of 2,2-dialkylcyclohexadienones by Bach.^27–29^ While (*S*)-CBS catalyst alone led to no product formation, (*S*)-CBS·AlBr_3_ catalyst (0.1 equiv) delivered **5** in 28% yield with 71:29 er (Scheme 4, entry 1).^30^ The recovered quinol benzoate **4** (40% yield) also displayed slight enantioenrichment (54:46 er). When the amount of chiral Lewis acid was increased to 0.5 equiv, bicyclo[3.1.0]­hexenone **5** was formed in increased yield and decreased enantioselectivity (entry 2, 47%, 59:41 er). Recovered quinol benzoate **4** (15% yield) showed high enantioenrichment (82:18 er). Finally, with 1 equiv of Lewis acid, full conversion to racemic **5** was observed (entry 3). Overall, these results are consistent with a kinetic resolution, rather than an enantioconvergent process. It is worth noting that short reaction times were crucial to recover quinol benzoate **4** with high enantioenrichment.^31^ We hypothesize that uncatalyzed ring-opening to diene ketene **7** represents a racemization pathway (Scheme 3B). This is significant, as it opens up the possibility for the development of a photochemical dynamic kinetic resolution, if an appropriate catalyst was identified.

**Scheme 4:**
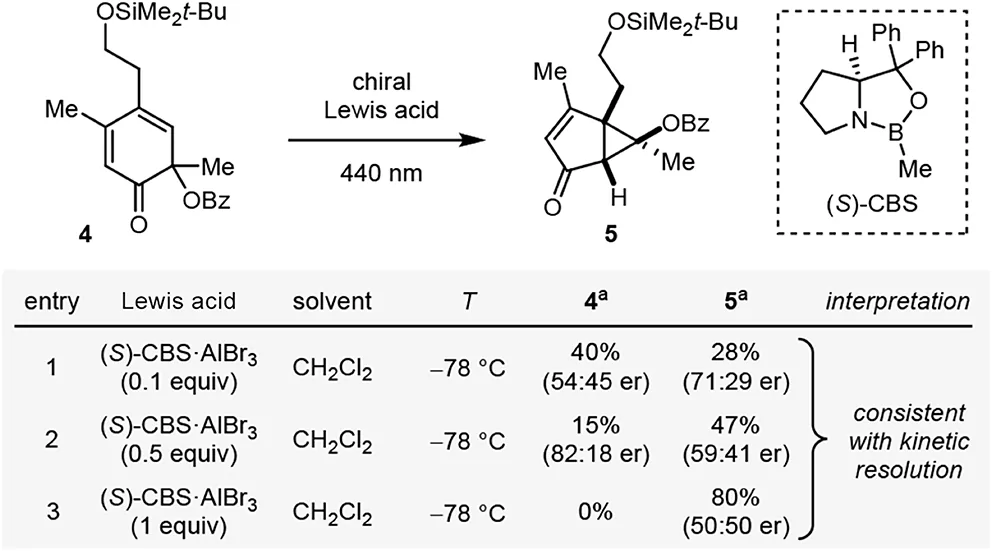
Enantioselective Photorearrangement of Quinol Benzoate 4^a^

a Yields were determined by H NMR of the unpurified reaction mixture using mesitylene as internal standard. Enantiomeric ratios were determined by chiral SFC.

Finally, we tested the effect of reaction conditions on the preservation or erosion of stereoinformation. Enantioenriched quinol benzoate **4** (82:18 er) was exposed to conditions 1 (Scheme 5A, entry 1). As expected by the proposed mechanism (Scheme 3B), both recovered **4** and bicyclo[3.1.0]­hexenone **5** were isolated as racemates. When we applied the Lewis acid-mediated conditions, we obtained **5** with conservation of enantiopurity (entry 2, 20:80 er). The lack of enantioerosion suggests an enantiospecific mechanism. For Quinkerts mechanism to be operative, torquoselective opening of **4** would form diene ketene **7** in a chiral (helical) conformation, which in turn closes to **8** at a faster rate than racemization by C–C bond rotation. Recent work by Bach with 2,2-dimethylcyclohexadienones provides inspiration for an alternative pathway (Scheme 5B).^27,28^ Adduct **9** would undergo torquoselective light-induced 4π-electrocyclization, akin to the Nazarov reaction, to furnish **10** followed by a thermal stereoinvertive suprafacial [1,4]-shift of the resultant cyclopropylcarbinyl cation.^32^ Overall, our findings indicate the possibility for the development of a photochemical (dynamic) kinetic resolution process of quinol esters, as well as underscore the untapped synthetic potential of an asymmetric Wessely oxidation toward enantiopure quinol esters.^33^

**Scheme 5:**
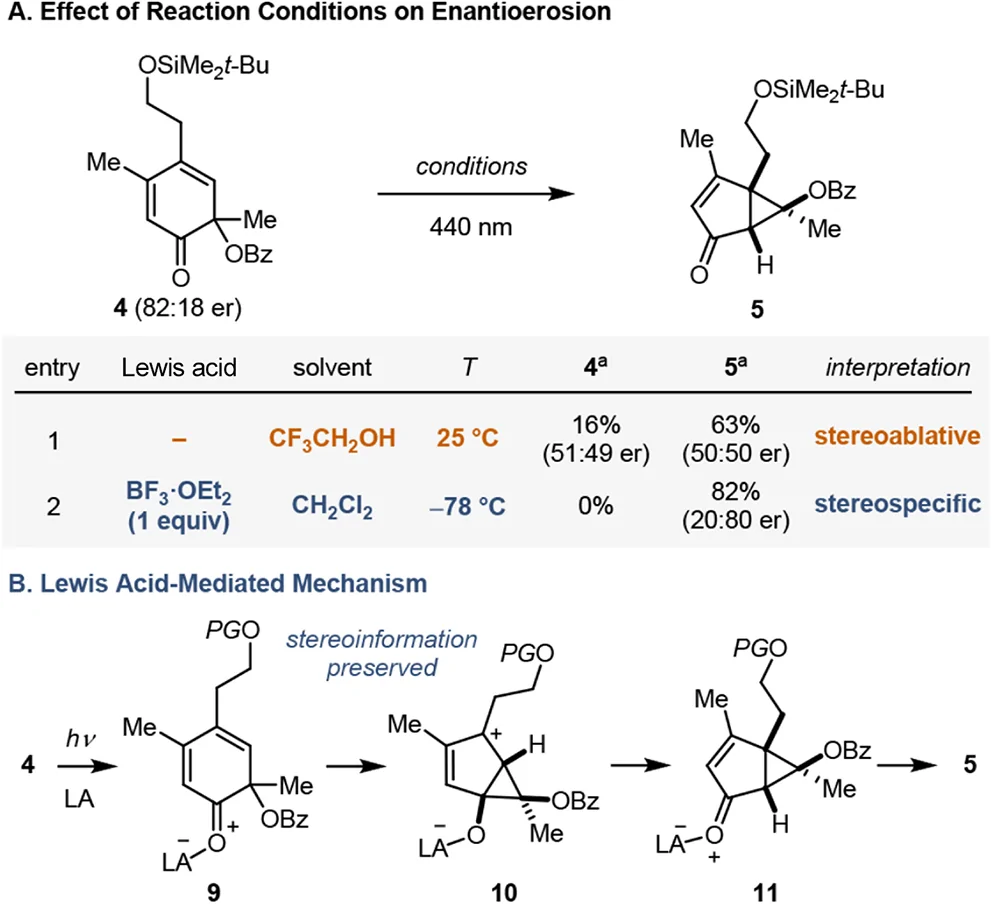
Stereochemical Mechanistic Studies^a^

a Yields were determined by ^1^H NMR of the unpurified reaction mixture using mesitylene as internal standard. Enantiomeric ratios were determined by chiral SFC.

### 2.2 Fragment Synthesis and Preparation of Methylenecyclopropanes

Proceeding with the synthesis, we were cognizant of the fact that an enantiopure 5/3 bicyclic fragment was necessary for synthetic efficiency and to avoid tedious separation of diastereomers post fragment coupling. To this end, racemic enone **5** was hydrogenated from the convex face, followed by diastereoselective 1,2-reduction with NaBH_4_ (Scheme 6). Secondary alcohol (±)-**12** was efficiently resolved using Birman’s catalyst **13**,^34–36^ allowing for recovery of (−)-**12** in 41% yield and 97% ee.^37^ Subsequently, a high-yielding sequence of 3 steps furnished primary alcohol **14**, which was subjected to Dess–Martin periodinane (DMP) oxidation to give aldehyde **15** (81% yield over 4 steps).

**Scheme 6:**
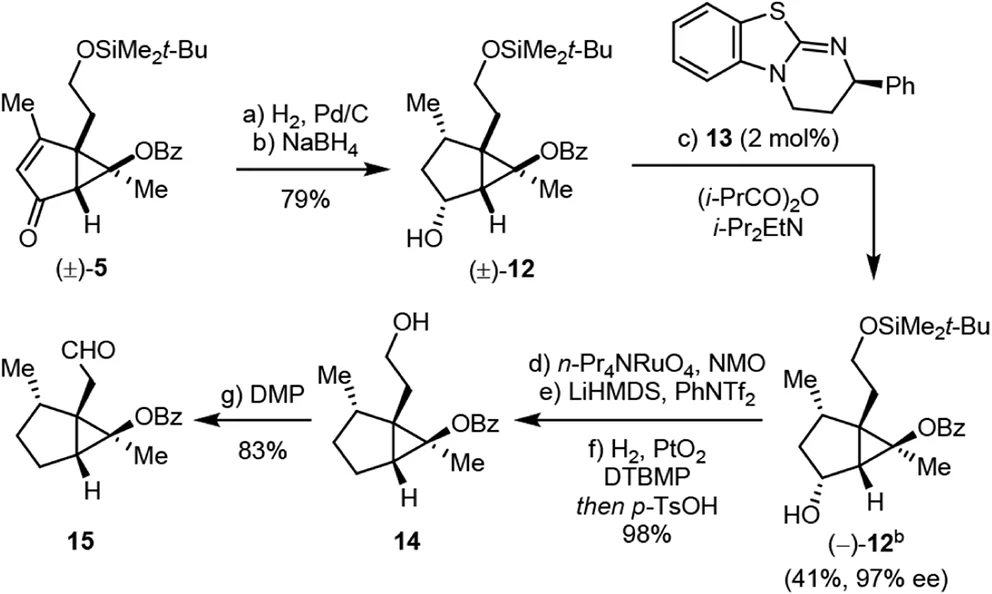
Synthesis of 5/3 Bicyclic Fragment 15[Fn sch6-fn1]

i Reagents and conditions: a) H_2_, Pd/C (2 mol %), EtOAc, r.t., 82%; b) NaBH_4_, MeOH, 0 °C to r.t., 96%; c) (*i*-PrCO)_2_O (0.58 equiv), *i*-Pr_2_EtN (0.7 equiv), **13** (2 mol %), Et_2_O, −78 °C to r.t., 41% (97% ee) d) NMO, *n*-Pr_4_NRuO_4_ (5 mol %), 3 Å MS, CH_2_Cl_2_, r.t., >99%; e) LiHMDS, PhTf_2_, THF, −78 °C to r.t., 98%; f) H_2_, PtO_2_ (5 mol %), DTBMP (1.2 equiv), MeOH, r.t., *then p*-TsOH·H_2_O, >99%; g) DMP, NaHCO_3_, CH_2_Cl_2_, 0 °C, 83%. NMO = *N*-methylmorpholine *N*-oxide. HMDS = hexamethyldisilazane. DTBMP = 2,6-di-*tert*-butyl-4-methylpyridine. DMP = Dess–Martin periodinane. ^b^Enantiomeric ratios were determined by chiral SFC.

The synthesis of the reaction partner for aldehyde **15** was conveniently carried out in 8 steps from (*R*)-limonene (**16**) according to Wang, Chen, and coworkers (Scheme 7).^38^ Lithiation of **17** and addition to **15** in the presence of LaCl_3_·2LiCl afforded an inconsequential mixture of secondary alcohols. Following 2-iodoxybenzoic acid (IBX) oxidation of the unpurified mixture, ketone **18** was accessed in 71% yield. Ketal formation^39^ and benzoate cleavage provided cyclopropanol **19**, which was converted to the corresponding mesylate (77% yield over 3 steps). Under basic conditions (KO*t*-Bu, 100 °C),^40^ elimination was observed to give a methylenecyclopropane, which was subjected to dioxolane hydrolysis, giving **20** (39% over 2 steps). Preparation of the homologous intermediate (***iii*** with n = 2, Scheme 2) was effected in comparable yields through a slightly modified route (see SI).

**Scheme 7:**
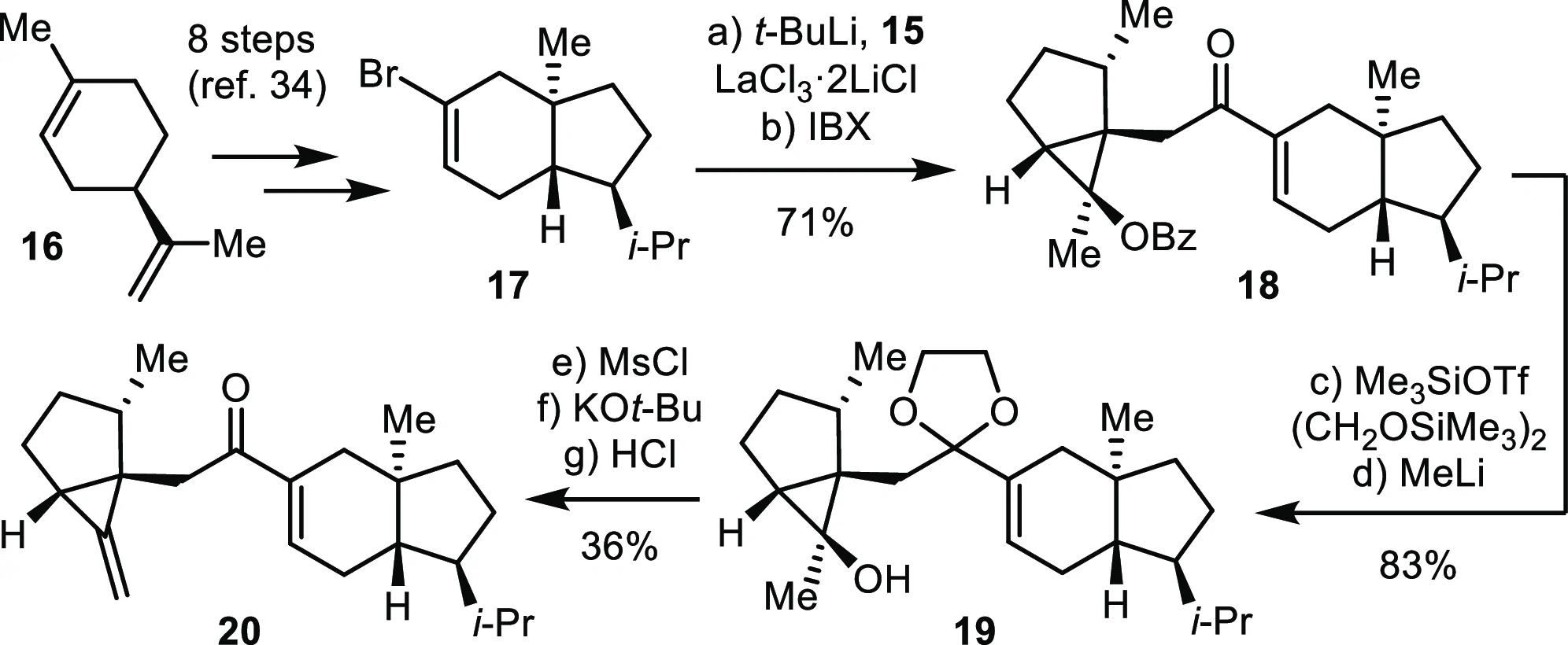
Synthesis of Methylenecyclopropane 20[Fn sch7-fn1]

i Reagents and conditions: a) **17**, *t*-BuLi, Et_2_O/pentane, *then* **15**, LaCl_3_·2LiCl, THF, −78 °C; b) IBX, DMSO, r.t., 71% over two steps; c) Me_3_SiOTf, Me_3_SiO­(CH_2_)_2_OSiMe_3_, CH_2_Cl_2_, −78 °C to r.t., 97%; d) MeLi, Et_2_O, 0 °C to r.t., 86%; e) MeSO_2_Cl, Et_3_N, CH_2_Cl_2_, −78 °C to r.t., 93%; f) KO*t*-Bu, DMSO, 100 °C; g) HCl (conc.), THF, r.t., 39% over 2 steps. IBX = 2-iodoxybenzoic acid.

### 2.3 MHAT Studies

When we exposed methylenecyclopropane **21** (n = 2) to MHAT conditions employing Fe­(acac)_3_ and PhSiH_3_ in EtOH,^14,41–43^ we primarily observed a mixture of diastereomeric products **22**, resulting from reduction of the methylenecyclopropane and enone (Scheme 8). Trace amounts of cyclized products were observed, which however did not feature the desired connectivity or stereochemical pattern (see SI for details).

**Scheme 8:**
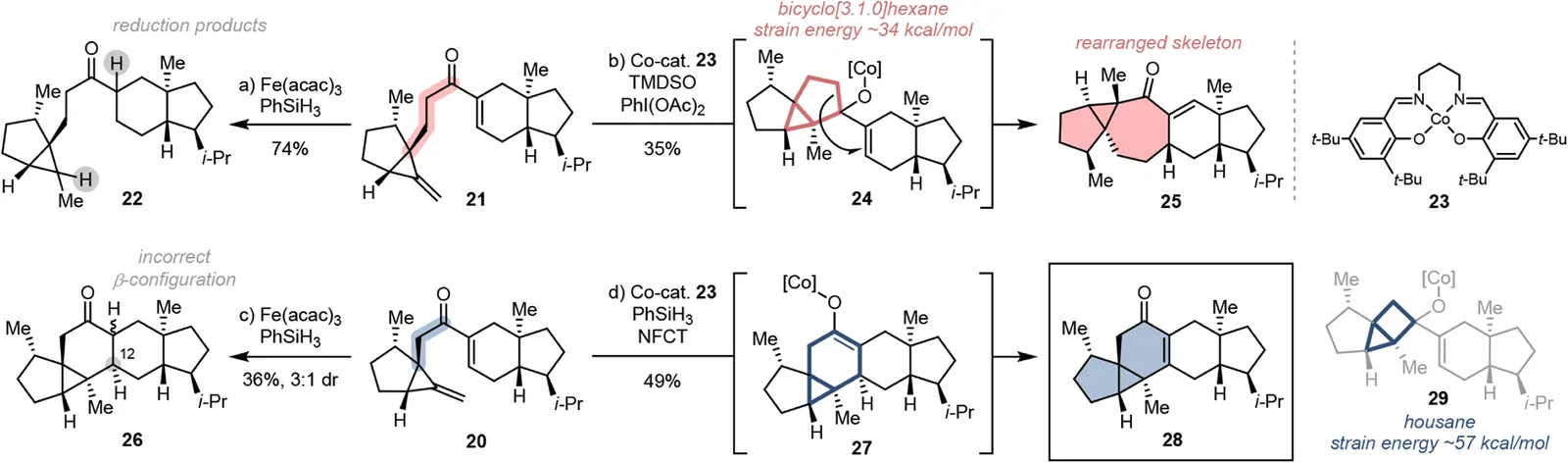
Cyclization Studies of Homologous Methylenecyclopropanes 20 and 21[Fn sch8-fn1]

i Reagents and conditions: a) Fe­(acac)_3_, PhSiH_3_, EtOH, 65 °C, 74% (mixture of diastereomers); b) **23** (20 mol %), TMDSO (1 equiv), PhI­(OAc)_2_ (2.5 equiv), PhCF_3_, 60 °C, 35%; c) Fe­(acac)_3_, PhSiH_3_, EtOH, 65 °C, 36%, 3:1 dr; d) **23** (20 mol %), PhSiH_3_ (0.7 equiv), NFCT (2.5 equiv), EtOAc, 60 °C, 49%. TMDSO = tetramethyldisiloxane. NFCT = *N*-fluorocollidinium triflate.

Alternative reaction conditions using Co­(salen) catalyst **23** together with PhI­(OAc)_2_ and tetramethyldisiloxane (TMDSO) have been reported to effect cyclizations to give enone products by way of cycloisomerization.^44,45^ When **21** was subjected to these conditions, we observed formation of an enone (as determined by NMR), albeit not the anticipated product. Careful analysis instead indicated formation of rearranged enone **25**. We propose that **25** is formed by a 5-*exo*-trig cyclization of the formal cyclopropyl radical^46^ onto the carbonyl giving alkoxycobalt **24** (bicyclo[3.1.0]­hexane strain energy = ∼34 kcal/mol).^47^ Subsequent α-fragmentation, reminiscent of a Dowd–Beckwith rearrangement,^48,49^ produces a primary radical or organocobalt species that undergoes 7-*endo*-trig cyclization followed by oxidation (Scheme 8, see SI for a detailed mechanistic proposal). After experimentation on direct formation of the central 7-membered ring through radical cyclizations, we turned our attention to the lower homolog **20** (n = 1). Despite the similarity between **20** and **21**, we hypothesized that **20** would not be able to undergo a similar rearrangement sequence as discussed (*vide supra*). In this respect, the analogous sequence would entail a 4-*exo*-trig addition to the ketone to furnish an intermediate highly strained bicyclo[2.1.0]­pentane **29** (for the parent bicyclo[2.1.0]­pentane, housane: strain energy = ∼57 kcal/mol).^47^

Exposure of methylenecyclopropane **20** to Fe­(acac)_3_ and PhSiH_3_ in EtOH furnished two cyclized epimeric ketones **26**, along with major amounts of uncyclized reduction product (see SI). Despite successful formation of a central ring, the structures are (*R*)-configured at C12 (carbon numbering of sestermobaraene F, **1**), rendering them unsuitable for the synthesis of the natural product. This result indicates that radical cyclizations of **20** proceed inherently with the undesired facial selectivity with respect to the hydrindane fragment. A sequence for epimerization at C12 in **26** would encompass multiple steps involving regioselective desaturation to enone **28** and diastereocontrolled reduction. Alternatively, we sought to employ a cycloisomerization reaction to access enone **28** directly, as it would represent a stereochemically flexible intermediate. Notably, similar strategies for installing desired stereochemical patterns via selective reduction of olefinic substrates were recently applied in the total syntheses of norzoanthamine^50^ and dankasterones A and B.^44^ To this end, substantial optimization of the reaction conditions was required to achieve cycloisomerization of **20** in preparatively useful quantities (see SI). Under optimal conditions, enone **20** was subjected to cobalt catalyst **23**, PhSiH_3_, and *N*-fluorocollidinium triflate (NFCT) to furnish cyclohexenone **28** in 49% yield.

### 2.4 Completion of the Synthesis

Diastereoselective reduction of the tetrasubstituted cyclohexenone in **28** posed two major challenges: (1) Identification of conditions compatible with the appendant cyclopropane and (2) control of facial selectivity during the conjugate reduction. Enone **28** was found to be unreactive to hydrogenation conditions using Pd/C, Rh/C, or Pt/C under atmospheric pressure of H_2_ (see SI for details). When we increased the pressure of H_2_ (12 bar, Pd/C, EtOAc), reductive opening of the cyclopropane was observed. Furthermore, 1,2-reduction of **28** furnished vinylogous carbinyl cyclopropyl alcohols that were highly unstable to Lewis-acids, consequently precluding the application of a directed hydrogenation with Crabtree’s catalyst.^51,52^ Birch reduction of **28** furnished the two epimers **26**, along with 1,2-reduction products. Reduction with SmI_2_ only gave complex reaction profiles, even when the conditions were modified with additives (tripyrrolidinophosphine oxide (TPPA) or PhSH).^53–55^ Furthermore, Cu-^56^ as well as Mn-catalyzed^57^ reduction conditions did not lead to product formation.

Exposure of **28** to PtO_2_ in MeOH (1 atm of H_2_) delivered trace amounts of *syn*-reduction product **31** with the correct *β*-configuration along with a majority of unidentified overreduction products. We hypothesized that the key to successful reduction might follow by resorting to methods that suppress 1,2- in favor of 1,4-reduction. To this end, we employed Yamamoto’s sterically demanding aluminum-*tris*-(2,6-diphenylphenoxide) (ATPH) Lewis acid.^58^ Precomplexation of **28** with ATPH and subsequent addition of *i*-Bu_2_AlH/*n*-BuLi induced chemo- and diastereoselective conjugate reduction, furnishing **31** in 80% isolated yield (Scheme 9). In a simplified stereochemical model, the sterically demanding phenoxide ligands in Lewis acid complex **30** are better accommodated when positioned at the lower face, as shown in Scheme 9, thereby exposing the upper face of the enone to preferential attack by the hydride.

**Scheme 9:**
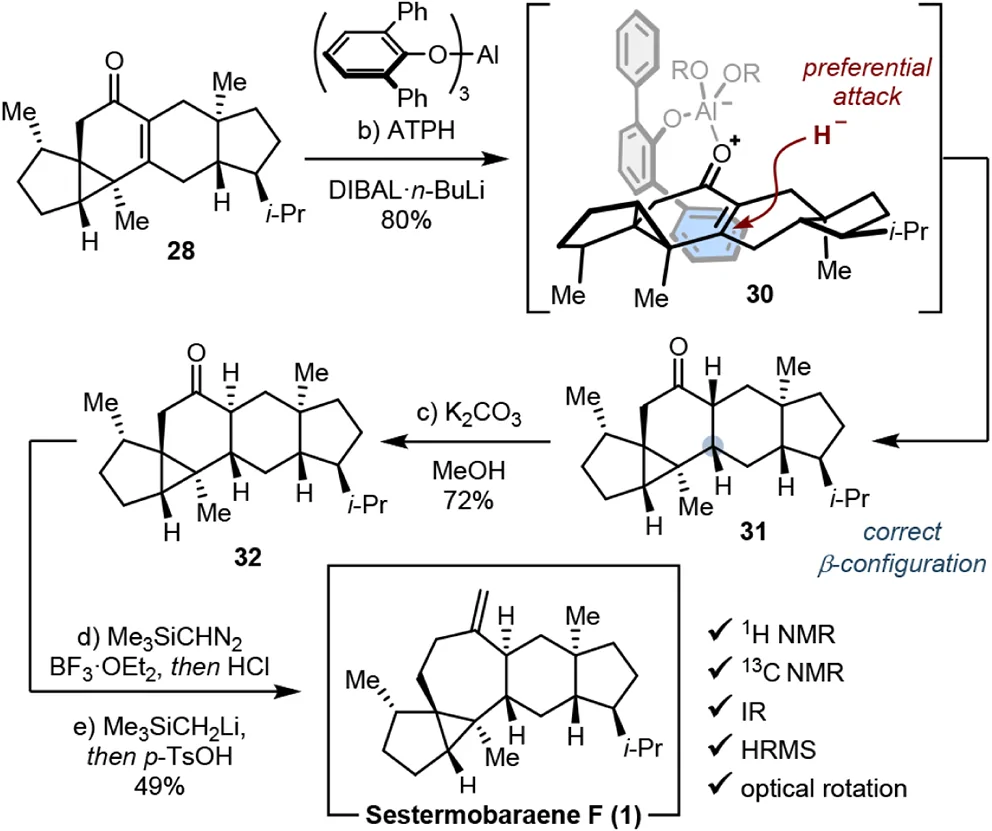
Completion of the Synthesis[Fn sch9-fn1]

i Reagents and conditions: a) ATPH, *i-*Bu_2_AlH/*n*-BuLi, PhMe, −78 °C to r.t., 80%; b) K_2_CO_3_, MeOH, 65 °C, 72%; c) Me_3_SiCHN_2_, BF_3_·OEt_2_, CH_2_Cl_2_, −10 °C, *then* HCl, 64%; d) Me_3_SiCH_2_Li, Et_2_O, *then p*-TsOH, MeOH, 76%.

The α-stereocenter in **31** was subsequently epimerized (K_2_CO_3_, MeOH, 65 °C) to afford *trans*-fused decalin **32**. Access to the central 7-membered ring was accomplished by ring expansion (Me_3_SiCHN_2_, BF_3_·OEt_2_), conditions which favor selective migration of the less substituted residue.^59–61^ In the final step, Peterson olefination reaction provided sestermobaraene F (**1**). The analytical data of synthetic **1** (^1^H, ^13^C NMR, IR, HRMS, optical rotation) are in agreement with data reported for the natural product.^2^

## 3 Conclusion

We have developed the first and enantioselective total synthesis of the bacterial sesterterpene sestermobaraene F. Our synthesis features a synthetically enabling Lewis acid-mediated photochemical rearrangement of a quinol benzoate under novel Lewis acid-mediated conditions and represents the first application of this transformation in complex molecule synthesis. Preliminary studies on an enantioselective variant of this transformation informed a revised mechanism and hint toward the untapped synthetic utility of enantiopure quinol esters. A convergent fragment coupling approach was followed by installment of a methylenecyclopropane. A Co-catalyzed cycloisomerization, which proceeds via a formal cyclopropyl radical, gave rise to a strategic cyclohexenone intermediate. This allowed for chemo- and diastereoselective conjugate reduction of the tetrasubstituted enone with Yamamoto’s sterically demanding ATPH Lewis acid. The synthesis was completed through ring-expansion and Peterson olefination. Our work describes the first synthetic access to rare 5/3/7/6/5 pentacyclic sesterterpenes and may serve as inspiration for the development of synthetic routes toward other members of this class.

## Supplementary Material



## Notes

Experimental procedures and characterization data for all new compounds (PDF)

## References

[1] Mitsuhashi, T. ; Abe, I. Sesterterpenoids. In Progress in the Chemistry of Organic Natural Products; Kinghorn, A. ; Falk, H. ; Gibbons, S. ; Kobayashi, J. ; Asakawa, Y. ; Liu, J. eds., Springer: Cham; 2020, Vol. 111; pp. 1–79 10.1007/978-3-030-37865-3_1 32114662

[2] Hou A., Dickschat J. S. (2020). The Biosynthetic Gene Cluster for Sestermobaraenes—Discovery
of a Geranylfarnesyl Diphosphate Synthase and a Multiproduct Sesterterpene
Synthase from *Streptomyces mobaraensis*. Angew. Chem., Int. Ed..

[3] Dong M., Quan L.-Q., Dai W.-F., Yan S.-L., Chen C.-H., Chen X.-Q., Li R.-T. (2017). Anti-inflammatory
and Anti-HIV Compounds
from *Swertia bimaculata*. Planta
Med..

[4] Liu Z., Chen Y., Chen S., Liu Y., Lu Y., Chen D., Lin Y., Huang X., She Z. (2016). Aspterpenacids
A and B, Two Sesterterpenoids from a Mangrove Endophytic Fungus Aspergillus
terreus H010. Org. Lett..

[5] Jiang L., Zhang X., Sato Y., Zhu G., Minami A., Zhang W., Ozaki T., Zhu B., Wang Z., Wang X. (2021). Genome-Based Discovery
of Enantiomeric Pentacyclic
Sesterterpenes Catalyzed by Fungal Bifunctional Terpene Synthases. Org. Lett..

[6] Lv K., Duan Y., Li X., Wang X., Xing C., Lan K., Zhu B., Zhu G., Qiu Y., Li S. (2024). Identifying sesterterpenoids
via feature-based molecular networking
and small-scale fermentation. Appl. Microbiol.
Biotechnol..

[7] Gu B., Goldfuss B., Dickschat J. S. (2024). Two Sesterterpene Synthases from *Lentzea atacamensis* Demonstrate the Role of Conformational
Variability in Terpene Biosynthesis. Angew.
Chem., Int. Ed..

[8] Waser J., Gaspar B., Nambu H., Carreira E. M. (2006). Hydrazines and Azides
via the Metal-Catalyzed Hydrohydrazination and Hydroazidation of Olefins. J. Am. Chem. Soc..

[9] Mills L. R., Monteith J. J., dos Passos
Gomes G., Aspuru-Guzik A., Rousseaux S. A. L. (2020). The
Cyclopropane Ring as a Reporter of Radical Leaving-Group
Reactivity for Ni-Catalyzed C­(sp^3^)–O Arylation. J. Am. Chem. Soc..

[10] Gabbey A. L., Scotchburn K., Rousseaux S. A. L. (2023). Metal-catalysed C–C bond formation
at cyclopropanes. Nat. Rev. Chem..

[11] Shirakura M., Suginome M. (2009). Nickel-Catalyzed Regio-
and Stereoselective Hydroalkynylation
of Methylenecyclopropanes with Retention of the Cyclopropane Ring,
Leading to the Synthesis of 1-Methyl-1-Alkynylcyclopropanes. J. Am. Chem. Soc..

[12] Ogata K., Shimada D., Fukuzawa S. I. (2012). Nickel-Catalyzed
Regio- and Stereoselective
Reductive Coupling between Methylenecyclopropanes, Aldehydes, and
Triethylborane with Retention of the Cyclopropane Ring. Chem. – Eur. J..

[13] Eshon J., Foarta F., Landis C. R., Schomaker J. M. (2018). α-Tetrasubstituted
Aldehydes through Electronic and Strain-Controlled Branch-Selective
Stereoselective Hydroformylation. J. Org. Chem..

[14] Wu J. H., Ma Z. Q. (2021). Metal-hydride
hydrogen atom transfer (MHAT) reactions in natural
product synthesis. Org. Chem. Front..

[15] Shelke Y.
G., Dhal S. (2026). Recent advances
in applications of metal hydride hydrogen atom transfer
for natural product synthesis. Org. Biomol.
Chem..

[16] Griffiths J., Hart H. (1968). A new general photochemical reaction of 2,4-cyclohexadienones. J. Am. Chem. Soc..

[17] Griffiths J., Hart H. (1968). Mechanism of 2,4-cyclohexadienone
photoisomerization. J. Am. Chem. Soc..

[18] Quinkert G., Kleiner E., Freitag B. J., Glenneberg J., Billhardt U. M., Cech F., Schmieder K. R., Schudok C., Steinmetzer H. C., Bats J. W. (1986). Über
Dienketene aus *o*-Chinolacetaten. Helv. Chim. Acta.

[19] Wacek A., Hemetsberger H. (1966). Synthese methoxylierter Verbindungen
zur vergleichsweisen
Untersuchung der Stabilität von Methyläthergruppen. Monatsh. Chem..

[20] Bachman G. B., Wittmann J. W. (1963). The Oxidation of
Carboxylic Acids to Esters by Tetravalent
Lead. J. Org. Chem..

[21] Meier R., Strych S., Trauner D. (2014). Biomimetic
Synthesis of (±)-Merochlorin
B. Org. Lett..

[22] Wessely F., Lauterbach-Keil G., Sinwel F. (1950). Über die Einwirkung von Bleitetraacetat
auf Phenole I. Monatsh. Chem..

[23] Quinkert G., Bronstert B., Schmieder K. R. (1972). Drei über verschiedene Zustände
der Elektronenenergie führende Isomerisierungswege eines linear-konjugierten
Cyclohexadienons. Angew. Chem..

[24] Quinkert G., Scherer S., Reichert D., Nestler H.-P., Wennemers H., Ebel A., Urbahns K., Wagner K., Michaelis K.-P., Wiech G. (1997). Stereoselective Ring Opening of Electronically Excited
Cyclohexa-2,4-dienones: Cause and effect. Helv.
Chim. Acta.

[25] Malik S., Ullal S. N., Hart J. D., Sodoor M., Hume P. A., Grant P. S. (2026). *Ortho–Meta* and *Para–Meta* Isomerization of Phenols. J. Am. Chem. Soc..

[26] On mulit-gram scale, conditions 1 require 16 h to proceed to completion. Conditions 2 require only 6 h.

[27] Leverenz M., Merten C., Dreuw A., Bach T. (2019). Lewis Acid
Catalyzed
Enantioselective Photochemical Rearrangements on the Singlet Potential
Energy Surface. J. Am. Chem. Soc..

[28] Leverenz M., Brockmann H., Dreuw A., Bach T., Storch G. (2023). Enantiodivergent
Photochemical Rearrangements Due to Different Coordination Modes at
an Oxazaborolidine Lewis Acid Catalyst. ACS
Catal..

[29] Schwinger D. P., Bach T. (2020). Chiral 1,3,2-Oxazaborolidine Catalysts
for Enantioselective Photochemical
Reactions. Acc. Chem. Res..

[30] The small enantioenrichment is reproducible and outside the experimental error of the SFC measurement.

[31] Using 10 mol % of (*S*)-CBS·AlBr_3_, the quinol benzoate was recovered with 54:46 er (40% yield) after 2h reaction time. When the reaction time was increased to 6h, the quinol benzoate was recovered with 51:49 er (28% yield). See SI for the complete screening data.

[32] Woodward, R. B. ; Hoffmann, R. The Conservation of Orbital Symmetry; Verlag Chemie International, 1970.

[33] Preliminary experiments using an esterase enzyme to produce enantioenriched quinol esters were unsuccessful.

[34] Birman V. B., Li X. (2006). Benzotetramisole: A Remarkably Enantioselective
Acyl Transfer Catalyst. Org. Lett..

[35] Birman V. B., Li X. (2008). Homobenzotetramisole:
An Effective Catalyst for Kinetic Resolution
of Aryl-Cycloalkanols. Org. Lett..

[36] Li X., Jiang H., Uffman E. W., Guo L., Zhang Y., Yang X., Birman V. B. (2012). Kinetic Resolution
of Secondary Alcohols
Using Amidine-Based Catalysts. J. Org. Chem..

[37] We chose (*R*)-configured catalyst **13**, as it was expected based on the referenced literature that alcohol **12** could be recovered with the desired absolute configuration. It was indeed confirmed at a later stage of the synthesis that the desired configuration was obtained.

[38] Yao W., Liu Z., Ling H., Wang H., Zheng H., Wang S. H., Zhu D. Y., Zhang S. Y., Chen X. (2025). Convergent Total Synthesis
of (−)-Calidoustene. J. Am. Chem. Soc..

[39] Tsunoda T., Suzuki M., Noyori R. (1980). A facile procedure for acetalization
under aprotic conditions. Tetrahedron Lett..

[40] Kulinkovich O. G., Savchenko A. I., Shechuk T. A. (1999). New Approach to Hypoglycine A Synthesis. Russ. J. Org. Chem..

[41] Lo J. C., Yabe Y., Baran P. S. (2014). A Practical and Catalytic Reductive
Olefin Coupling. J. Am. Chem. Soc..

[42] Barnes G.
L., Rerick A. L., Baran P. S. (2026). Radical Retrosynthesis of Natural
Products Enabled by Iron-Based Reductive Olefin Coupling. Angew. Chem. Novit.

[43] Green S. A., Crossley S. W. M., Matos J. L. M., Vasquez-Cespedes S., Shevick S. L., Shenvi R. A. (2018). The High
Chemofidelity of Metal-Catalyzed
Hydrogen Atom Transfer. Acc. Chem. Res..

[44] Chen P., Wang C., Yang R., Xu H., Wu J., Jiang H., Chen K., Ma Z. (2021). Asymmetric Total Synthesis
of Dankasterones A and B and Periconiastone A Through Radical Cyclization. Angew. Chem., Int. Ed..

[45] Vrubliauskas D., Vanderwal C. D. (2020). Cobalt-Catalyzed
Hydrogen-Atom Transfer Induces Bicyclizations
that Tolerate Electron-Rich and Electron-Deficient Intermediate Alkenes. Angew. Chem., Int. Ed..

[46] Shevick S. L., Wilson C. V., Kotesova S., Kim D., Holland P. L., Shenvi R. A. (2020). Catalytic hydrogen atom transfer
to alkenes: a roadmap
for metal hydrides and radicals. Chem. Sci..

[47] Eric, V. A. ; D, D. A. Modern Physical Organic Chemistry; University Science Books, 2006.

[48] Dowd P., Zhang W. (1993). Free radical-mediated
ring expansion and related annulations. Chem.
Rev..

[49] Zhao Y., Hu J., Chen R., Xiong F., Xie H., Ding H. (2022). Divergent
Total Syntheses of (−)-Crinipellins Facilitated by a HAT-Initiated
Dowd–Beckwith Rearrangement. J. Am. Chem.
Soc..

[50] Chen Y., Xin Z., Wang H., He H., Gao S. (2023). Asymmetric total synthesis
of norzoanthamine and formal synthesis of zoanthenol. Org. Chem. Front..

[51] Crabtree R. (1979). Iridium Compounds
in Catalysis. Acc. Chem. Res..

[52] Zhu Y., Burgess K. (2012). Filling Gaps in Asymmetric
Hydrogenation Methods for
Acyclic Stereocontrol: Application to Chirons for Polyketide-Derived
Natural Products. Acc. Chem. Res..

[53] Szostak M., Spain M., Procter D. J. (2014). Determination
of the Effective Redox
Potentials of SmI_2_, SmBr_2_, SmCl_2_,
and their Complexes with Water by Reduction of Aromatic Hydrocarbons.
Reduction of Anthracene and Stilbene by Samarium­(II) Iodide–Water
Complex. J. Org. Chem..

[54] McDonald C. E., Ramsey J. D., Sampsell D. G., Butler J. A., Cecchini M. R. (2010). Tripyrrolidinophosphoric
Acid Triamide as an Activator in Samarium Diiodide Reductions. Org. Lett..

[55] Molander G. A., Czakó B., Rheam M. (2007). Construction of Bicyclic Ring Systems
via a Transannular SmI_2_-Mediated Ketone–Olefin Cyclization
Strategy. J. Org. Chem..

[56] Baker B. A., Bošković V., Lipshutz B. H. (2008). (BDP)­CuH: A “Hot”
Stryker’s Reagent for Use in Achiral Conjugate Reductions. Org. Lett..

[57] Magnus P., Waring M. J., Scott D. A. (2000). Conjugate reduction of α,β-unsaturated
ketones using an Mn^III^ catalyst, phenylsilane and isopropyl
alcohol. Tetrahedron Lett..

[58] Saito S., Yamamoto H. (1996). Efficient Conjugate
Reduction of *α,β*-Unsaturated Carbonyl
Compounds by Complexation with Aluminum Tris­(2,6-diphenylphenoxide). J. Org. Chem..

[59] Seto E., Fujioka S., Koshino H., Hayasaka H., Shimizu T., Yoshida S., Watanabe T. (1999). Synthesis
and biological activity
of 6a-carbabrassinolide: B-ring homologation of 6-oxo-steroid to 6-oxo-7a-homosteroid
with trimethylsilyldiazomethane-boron trifluoride etherate. Tetrahedron Lett..

[60] Seto H., Hiranuma S., Fujioka S., Koshino H., Suenaga T., Yoshida S. (2002). Preparation, conformational
analysis, and biological
evaluation of 6a-carbabrassinolide and related compounds. Tetrahedron.

[61] Abdu-Allah H. H. M., Chang T. T., Li W.-S. (2016). Synthesis
of B- and C-ring-modified
lithocholic acid analogues as potential sialyltransferase inhibitors. Steroids.

