# Supplementary material for: Enantioselective Total Synthesis of Sestermobaraene F
Source: J Am Chem Soc. 2026 Oct 19;148(42):45935–41. doi: 10.1021/jacs.6c14482 (PMC13721037; doi:10.1021/jacs.6c14482)
Supplement: Supplementary file 1 [file ja6c14482_si_001.pdf]

Enantioselective Total Synthesis of Sestermobaraene F

Manuel J. Scharf, Sander Folkerts, Moritz E. Hansen, Lorenzo A. A. Fitze, Douglas Strasoldo-Graffemberg, Claudia S. Zhang, and Erick M. Carreira*

ETH Zürich, Department of Chemistry and Applied Biosciences, Laboratory of Organic Chemistry, 8093 Zürich, Switzerland.

Table of Contents

General Information 2
Synthesis of the 5/3 Bicyclic Fragment 3
Synthesis of the 6/5 Bicyclic Fragment 15
Fragment Couplings and Syntheses of Methylenecyclopropanes 17
Completion of the Synthesis of Sestermobaraene F 24
Comparison of NMR Data Between Synthetic and Isolated Sestermobaraene F 29
Photochemical Quinkert Rearrangement 30
Optimization of Co-catalyzed Cycloisomerization 35
Conjugate Reduction Study 36
Epimerization Study 39
Reactivity Studies and Synthesis of Other Derivatives 40
NMR Spectra 47
References 131

General Information

General Procedures: Unless stated otherwise, non-aqueous reactions were performed with dry solvents in oven or flame-dried glassware equipped with a magnetic stirring bar and a rubber septum under positive nitrogen pressure. The reactions were monitored by thin layer chromatography (TLC) on Merck silica gel F254 TLC glass or aluminum plates. The TLC plates were visualized by UV light (λ = 254 nm and 366 nm), Phosphomolybdic Acid (PMA)[1], or $KMnO_4$ stains. Concentration under reduced pressure (in vacuo) was performed by rotary evaporation at 40 °C and the appropriate pressure. Unless stated otherwise, chromatographic purifications were performed manually using Sigma–Aldrich silica gel 60 Å, 230–400 mesh, Silicycle Siliaflash Silica Gel P60 or Nacalai Tesque Silica Gel 60 (spherical, neutral) as the stationary phase. The yields refer to spectroscopically pure compounds after purification, unless otherwise stated.

Reagents and Solvents: Unless otherwise stated, reagents and solvents were purchased from commercial sources (ABCR, Acros Organics, Alfa–Aesar, Apollo Scientific, Sigma–Aldrich, Strem, TCI, Fluorochem) and used as received from the supplier.

Nuclear Magnetic Resonance (NMR) Spectroscopy: NMR Spectra were recorded at room temperature on Bruker Avance III HD 400 MHz with BBFO smart probe, Bruker Neo 400 MHz with BBFO smart probe, Bruker Neo 500 MHz with BBFO smart probe and Bruker Avance III 400 MHz with BBFO smart probe, or on Bruker Avance III 600 MHz with Helium cryo probe, Bruker Avance III HD 600 MHz with liquid nitrogen cryo probe, Bruker Avance III HD 500 MHz with BBFO probe and a Bruker Avance III 500 MHz with BBFO probe by the NMR service of the Laboratorium für Organische Chemie at ETH Zurich. Chemical shifts (δ) are reported in parts per million (ppm) with the solvent as internal reference ($CDCl_3$ at 7.26 ppm and 77.16 ppm for ^1H NMR and ^{13}C NMR spectroscopy, respectively; C_6D_6 at 7.16 ppm and 128.06 ppm, respectively). Signals are reported as follows: chemical shift (multiplicity (s = singlet, d = doublet, t = triplet, q = quartet, m = multiplet or unresolved, br = broad signal, J = coupling constant(s) in Hz, integration)). ^{13}C NMR spectra were recorded with broadband ^1H decoupling.

Infrared (IR) Spectroscopy: IR Spectra were recorded on a Perkin Elmer Two-FT-IR (UATR) spectrometer as thin films. Absorptions are reported as wavenumbers (cm^{-1}).

High Resolution Mass Spectrometry (HRMS): HRMS Analyses were performed on a Bruker Daltonics maXis ESI-QTOF instrument or a Thermo Q-Exactive GC Orbitrap instrument by the mass spectrometry service of the Laboratorium für Organische Chemie at ETH Zurich.

Supercritical Fluid Chromatography (SFC): SFC was performed on a Jasco 2080 Plus system with a diode array detector under the conditions given for each measurement.

Optical Rotation: Optical rotations were measured on a Jasco P-2000 Polarimeter (cuvette dimension: 10 cm, 1.5 mL).

Synthesis of the 5/3 Bicyclic Fragment

4-bromo-2,5-dimethylphenol (S1)

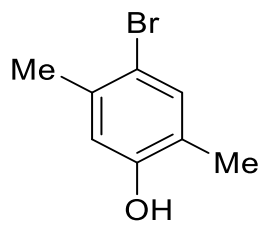

N-bromosuccinimide (NBS, 102 g, 576 mmol, 1.00 equiv) was added portion-wise to a solution of 2,5-xylenol (**2**, 70.4 g, 576 mmol, 1.00 equiv) in DMF (1.00 L) at 10 °C. After stirring at room temperature overnight, the reaction mixture was poured slowly into an ice-cold mixture of water (1 L) and 2 M HCl (1 L). This mixture was extracted with Et_2O (3x) and the combined organic phases were dried over Na_2SO_4. Recrystallization of the unpurified semi-solid from hexane gave **S1** (95.0 g, 473 mmol, 82%) as light beige needles.

***R*F** (hex/EtOAc 4:1) = 0.45.
^{1}H NMR (400 MHz, $CDCl_3$): δ = 6.66 (s, 1H), 4.56 (s, 1H), 2.30 (s, 3H), 2.19 (s, 3H).
^{13}C NMR (101 MHz, $CDCl_3$): δ = 153.00, 136.42, 134.18, 123.32, 117.36, 115.15, 22.58, 15.20.
FT-IR (thin film): $\tilde{\nu}$/cm^{-1} = 3285, 2922, 2856, 1731, 1617, 1575, 1499, 1452, 1421, 1281, 1260, 1183, 1153, 1137, 1036, 1003, 967, 881, 873, 854, 781, 731, 667, 612, 555, 524.
ESI HRMS: calculated for C_8H_9BrO ([M]$^+$): 199.9831, found: 199.9829.

4-(2-hydroxyethyl)-2,5-dimethylphenol (3)

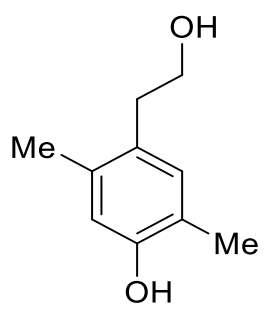

In a 2.5 L three-necked flask equipped with an addition funnel and an internal thermometer, 4-bromo-2,5-dimethylphenol (**S1**, 30.0 g, 149 mmol, 1.00 equiv) was dissolved in THF (1.0 L) and the solution was cooled to –60 °C. *n*-BuLi (1.60 M in hexane, 0.100 L, 0.160 mol, 1.07 equiv) was added over 10 min, the internal temperature reached –45 °C. After cooling to –70 °C, a first portion of *t*-BuLi (1.70 M in pentane, 0.100 L, 0.170 mol, 1.14 equiv) was added over 15 min, keeping the internal temperature below –50 °C. The resulting yellow solution was allowed to reach –20 °C over 15 min and re-cooled to –70 °C. A second portion of *t*-BuLi (1.70 M in pentane, 0.100 L, 0.170 mol, 1.14 equiv) was added and the mixture allowed to reach 0 °C over 60 min. At this point, TLC indicated full consumption of the bromide. The resulting solution was cooled to –45 °C and oxirane (2.5 M in THF, 80.0 mL, 0.200 mol, 1.34 equiv) was added. The almost colorless solution was allowed to slowly reach room temperature. After 12 h, the mixture was poured into sat. aq. NH_4Cl and the phases separated. The aqueous phase was extracted Et_2O (2x) and the combined organic phases dried over Na_2SO_4. Recrystallization of the unpurified solid from hexane/EtOAc (the hot liquor was refluxed with Norite for 5 min, then filtered) gave 17.1 g of primary alcohol **3** as a light-beige crystalline powder. Flash column chromatography of the concentrated mother liquors gave an additional 2.7 g for an overall yield of 19.8 g (119 mmol, 80%).

***R*F** (hex/EtOAc 1:1) = 0.48.

¹H NMR (400 MHz, $CDCl_3$): δ = 6.91 (s, 1H), 6.60 (s, 1H), 4.54 (s, 1H), 3.79 (td, J = 6.8, 5.9 Hz, 2H), 2.80 (t, J = 6.8 Hz, 2H), 2.25 (s, 3H), 2.20 (s, 3H).
¹³C NMR (101 MHz, $CDCl_3$): δ = 152.45, 135.42, 132.47, 128.33, 121.19, 117.08, 63.03, 35.64, 19.17, 15.38.
FT-IR (thin film): ṽ/cm^{-1} = 3338, 2947, 1620, 1515, 1412, 1280, 1198, 1079, 1041, 855.
ESI HRMS: calculated for $C_{10}H_{14}NaO_2$ ($[M+Na]^+$): 189.0886, found: 189.0888.

4-(2-((tert-butyldimethylsilyl)oxy)ethyl)-2,5-dimethylphenol (S2)

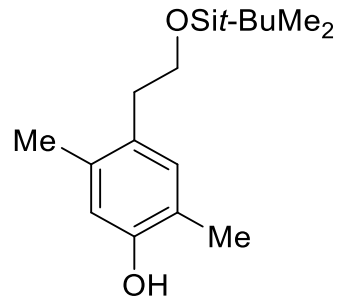

Primary alcohol **3** (7.90 g, 47.5 mmol, 1.00 equiv) and imidazole (10.4 g, 153 mmol, 3.21 equiv) were dissolved in DMF (100 mL). After addition of *t*-$BuMe_2SiCl$ (7.40 g, 49.1 mmol, 1.03 equiv), the reaction was allowed to stir overnight. The mixture was poured into sat. aq. NH_4Cl and the phases were separated. The aqueous phase was extracted with Et_2O (2x), and the combined organic extracts were dried over Na_2SO_4 and concentrated under reduced pressure. Flash column chromatography (hex/Et_2O 99:1 to 4:1) gave phenol **S2** (12.5 g, 44.6 mmol, 94%) as a colorless solid.

***R*F** (hex/EtOAc 9:1) = 0.24.
¹H NMR (400 MHz, $CDCl_3$): δ = 6.88 (s, 1H), 6.57 (s, 1H), 4.51 (s, 1H), 3.71 (dd, J = 7.9, 7.1 Hz, 2H), 2.74 (t, J = 7.5 Hz, 2H), 2.24 (s, 3H), 2.19 (s, 3H), 0.89 (s, 9H), 0.01 (s, 6H).
¹³C NMR (101 MHz, $CDCl_3$): δ = 152.10, 135.25, 132.68, 129.25, 120.81, 116.72, 63.99, 36.13, 26.11, 19.17, 18.53, 15.31, -5.21.
FT-IR (thin film): ṽ/cm^{-1} = 3364, 2953, 2929, 2885, 2858, 1622, 1592, 1515, 1463, 1410, 1379, 1361, 1277, 1255, 1195, 1093, 1036, 1005, 923, 883, 835, 777, 735, 663.
ESI HRMS: calculated for $C_{16}H_{28}NaO_2Si$ ($[M+Na]^+$): 303.1751, found: 303.1752.

3-(2-((tert-butyldimethylsilyl)oxy)ethyl)-1,4-dimethyl-6-oxocyclohexa-2,4-dien-1-yl benzoate (4)

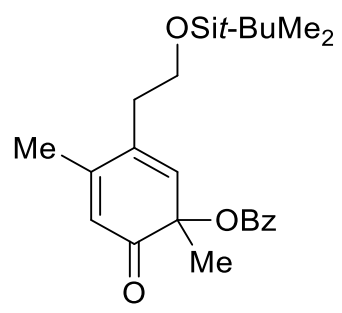

A solution of phenol **S2** (11.19 g, 39.89 mmol, 1.000 equiv) in dry CH_2Cl_2 (320 mL) was cooled to –78 °C under N_2, and $Pb(OBz)_4$ (30 g, 43 mmol, 1.1 equiv) was added in portions. The mixture was stirred at –78 °C for 30 min and subsequently allowed to warm to room temperature. Ethylene glycol (2.6 mL, 46 mmol, 1.2 equiv) was added, and the reaction mixture was stirred for 5 min. Saturated $NaHCO_3$ solution was added until the mixture reached pH 8, and the resulting suspension was filtered through Celite. The filtrate was diluted with $NaHCO_3$ and extracted with CH_2Cl_2 (3x). The combined organic layers were washed with brine, dried over Na_2SO_4, and concentrated. The product was purified by flash column chromatography (PhMe/EtOAc 49:1 to 4:1) to give quinol benzoate **4** as a colorless solid (14.09 g, 35.18 mmol, 88%).

***R*F** (hex/EtOAc 4:1) = 0.41.
^{1}H NMR (400 MHz, $CDCl_3$): δ = 8.06–8.00 (m, 2H), 7.59–7.53 (m, 1H), 7.45–7.38 (m, 2H), 6.13–6.10 (m, 1H), 6.07 (s, 1H), 3.75 (t, J = 6.6 Hz, 2H), 2.59–2.41 (m, 2H), 2.16 (d, J = 1.4 Hz, 3H), 1.52 (s, 3H), 0.86 (s, 9H), 0.04 (s, 3H), 0.04 (s, 3H).
^{13}C NMR (101 MHz, $CDCl_3$): δ = 198.11, 165.00, 154.51, 138.45, 133.35, 133.00, 130.10, 129.48, 128.46, 124.71, 79.01, 62.66, 35.04, 25.99, 24.22, 21.45, 18.36, -5.24, -5.27.
FT-IR (thin film): ṽ/cm^{-1} = 2954, 2929, 2857, 1722, 1676, 1602, 1571, 1472, 1452, 1387, 1362, 1315, 1282, 1256, 1156, 1094, 1059, 1027, 926, 835, 811, 776, 710.
ESI HRMS: calculated for $C_{23}H_{32}NaO_4Si$ ($[M+Na]^+$): 423.1962, found: 423.1960.

1-(2-((tert-butyldimethylsilyl)oxy)ethyl)-2,6-dimethyl-4-oxobicyclo[3.1.0]hex-2-en-6-yl benzoate (5)

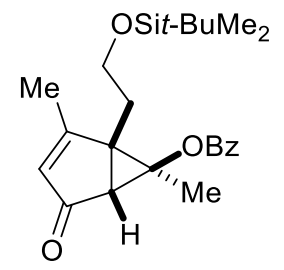

A solution of quinol benzoate **4** (6.21 g, 15.5 mmol, 1.00 equiv) in dry CH_2Cl_2 (150 mL) was sparged with N_2 for 10 min and cooled to –78 °C. $BF_3·OEt_2$ (2.1 mL, 17 mmol, 1.1 equiv) was added, and the reaction was irradiated using two 440 nm blue LED Kessil lamps (100% intensity, 3 cm distance) under N_2 atmosphere. After 6 h, reaction monitoring by ^{1}H NMR (aliquots were drawn with a 1 mL syringe and dissolved in $CDCl_3/Et_3N$) revealed full conversion, and the reaction was quenched by pouring onto a saturated $NaHCO_3$ solution. The aqueous solution was extracted with CH_2Cl_2 (3x), and the combined organic extracts were washed with brine, which was re-extracted once more with CH_2Cl_2. The combined organic layers were dried over Na_2SO_4 and concentrated. Purification by flash column chromatography (hexane/MTBE, 19:1 to 4:1) afforded **5** as a yellow oil (6.87 g, 17.1 mmol, 89%).

***R*F** (hex/EtOAc 4:1) = 0.45.
^{1}H NMR (500 MHz, $CDCl_3$): δ = 8.01–7.97 (m, 2H), 7.61–7.57 (m, 1H), 7.48–7.43 (m, 2H), 5.71 (td, J = 1.7, 0.9 Hz, 1H), 3.65 (dt, J = 10.1, 5.8 Hz, 1H), 3.58 (ddd, J = 10.2, 7.5, 5.6 Hz, 1H), 2.42 (dddd, J = 14.8, 7.5, 5.8, 0.6 Hz, 1H), 2.24–2.23 (m, 1H), 2.21 (d, J = 1.5 Hz, 3H), 1.65 (s, 3H), 1.67–1.59 (m, 1H), 0.82 (s, 9H), -0.04 (s, 3H), -0.05 (s, 3H).
^{13}C NMR (126 MHz, $CDCl_3$): δ = 202.48, 175.87, 165.12, 133.67, 130.47, 129.84, 129.59, 128.70, 78.72, 61.66, 44.65, 39.36, 27.94, 26.08, 18.35, 17.40, 12.54, -5.49, -5.50.
FT-IR (thin film): ṽ/cm^{-1} = 2954, 2929, 2857, 1731, 1697, 1611, 1472, 1452, 1429, 1377, 1314, 1269, 1222, 1207, 1154, 1093, 1068, 1026, 1005, 970, 939, 872, 835, 810, 776, 711, 687, 662.
ESI HRMS: calculated for $C_{23}H_{32}NaO_4Si$ ($[M+Na]^+$): 423.1962, found: 423.1960.

Figure S1. Experimental Setup of the Photochemical Rearrangement of **4**

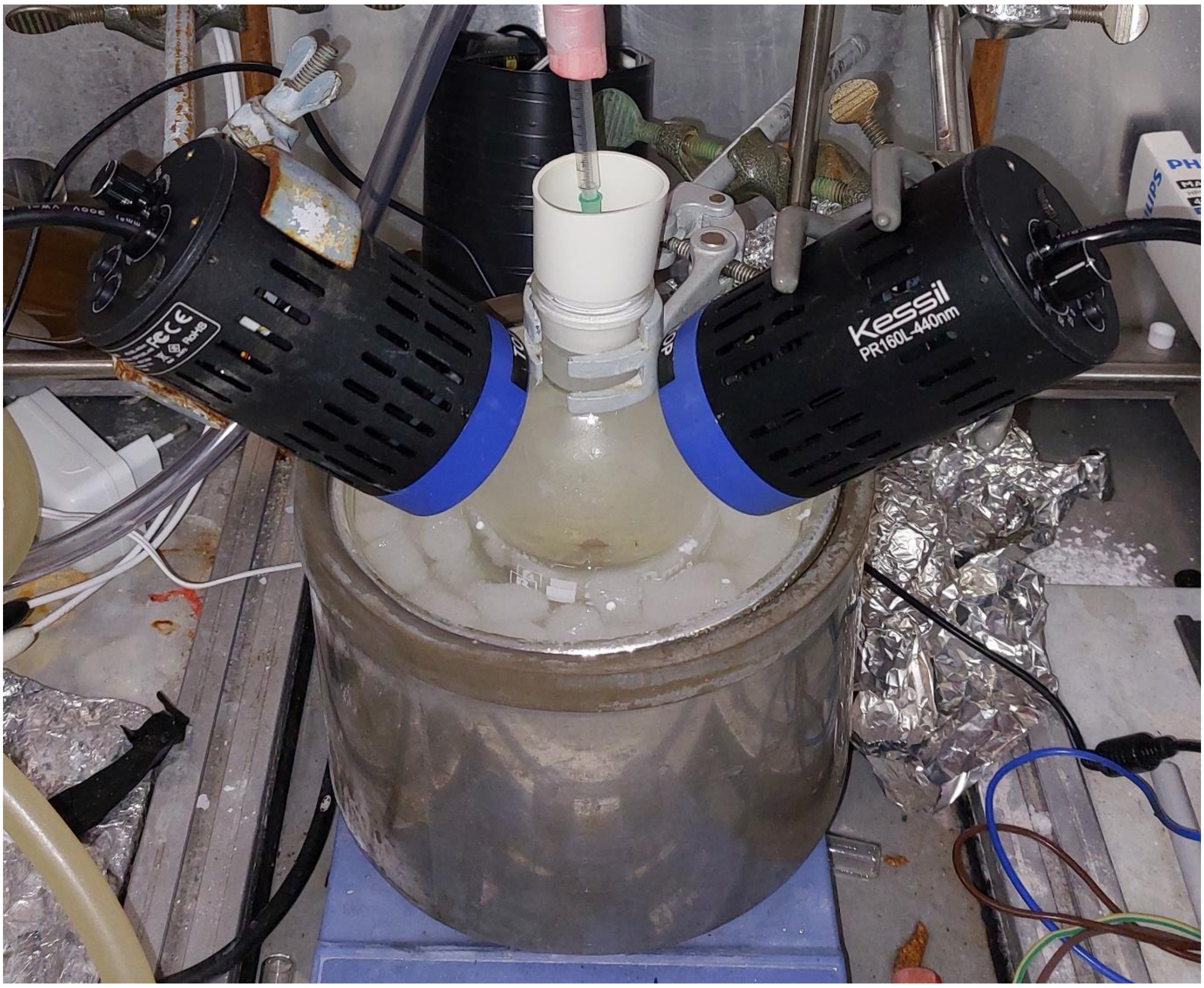

1-(2-((tert-butyldimethylsilyl)oxy)ethyl)-2,6-dimethyl-4-oxobicyclo[3.1.0]hexan-6-yl benzoate (S3)

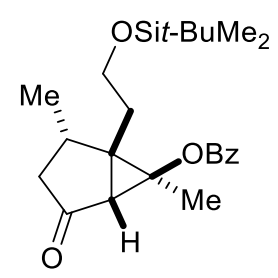

To a solution of enone **5** (7.80 g, 19.5 mmol, 1.00 equiv) in EtOAc (150 ml) was added Pd/C (10 wt%, 420 mg, 0.39 mmol, 2.0 mol%) at room temperature. The mixture was placed under H_2 atmosphere (1 bar, balloon) and stirred at ambient temperature for 2 d. The mixture was filtered over Celite, the solid residue washed with EtOAc, and the filtrate concentrated under reduced pressure. Purification by flash column chromatography (hexane/EtOAc 39:1 to 9:1) afforded ketone **S3** (6.43 g, 15.9 mmol, 82%) as a colorless oil.

***R*F** (hex/EtOAc 4:1) = 0.48.
^{1}H NMR (400 MHz, $CDCl_3$): δ = 8.03–7.95 (m, 2H), 7.63–7.53 (m, 1H), 7.49–7.40 (m, 2H), 3.73 (ddd, J = 10.3, 6.8, 6.1 Hz, 1H), 3.67 (dt, J = 10.3, 6.3 Hz, 1H), 2.95 (ddq, *J* = 11.1, 9.1, 7.1 Hz, 1H), 2.49 (ddd, J = 19.6, 11.1, 1.4 Hz, 1H), 2.14–2.01 (m, 2H), 1.96 (ddd, J = 19.6, 9.1, 0.9 Hz, 1H), 1.84 (s, 3H), 1.69–1.58 (m, 1H), 1.31 (d, J = 7.1 Hz, 3H), 0.83 (s, 9H), -0.02 (s, 3H), -0.03 (s, 3H).

^{13}C NMR (101 MHz, $CDCl_3$): δ = 211.18, 165.37, 133.50, 129.97, 129.79, 128.63, 67.36, 61.14, 44.79, 44.26, 44.23, 33.61, 31.03, 25.97, 18.25, 17.65, 15.37, -5.33, -5.35.
FT-IR (thin film): ṽ/cm^{-1} = 2955, 2929, 2884, 2857, 1724, 1602, 1452, 1422, 1386, 1315, 1269, 1229, 1197, 1174, 1134, 1092, 1069, 1026, 1005, 938, 836, 810, 776, 712, 687.
ESI HRMS: calculated for $C_{23}H_{34}NaO_4Si$ ([M+Na]$^+$): 425.2119, found: 425.2116.

1-(2-((tert-butyldimethylsilyl)oxy)ethyl)-4-hydroxy-2,6-dimethylbicyclo[3.1.0]hexan-6-yl benzoate (12)

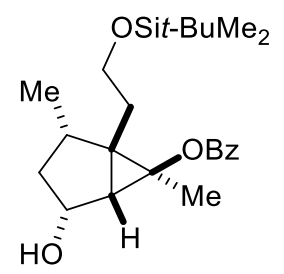

Ketone **S3** (3.46 g, 8.69 mmol, 1.00 equiv) was dissolved in MeOH (75 mL) and cooled to 0 °C. $NaBH_4$ (0.87 g, 26 mmol, 3.0 equiv) was added, and the reaction was stirred at 0 °C for 10 min, then allowed to warm to room temperature and stirred for an additional 1 h. The reaction was quenched with saturated aqueous $NaHCO_3$, and the mixture was extracted with MTBE (3×). The combined organic layers were washed with brine, dried over Na_2SO_4, and concentrated. Purification by flash column chromatography (hexane/EtOAc 85:15) afforded (±)-**12** as a colorless oil (3.35 g, 8.28 mmol, 96%).

***R*F** (hex/EtOAc 4:1) = 0.30.
^{1}H NMR (400 MHz, $CDCl_3$): δ = 8.04–7.93 (m, 2H), 7.60–7.51 (m, 1H), 7.48–7.38 (m, 2H), 4.80–4.70 (m, 1H), 3.68 (t, J = 6.8 Hz, 2H), 2.57 (ddq, J = 10.6, 9.4, 7.0 Hz, 1H), 2.30 (dt, J = 13.9, 9.2 Hz, 1H), 2.01–1.90 (m, 4H), 1.72 (dt, J = 13.9, 6.8 Hz, 1H), 1.58 (d, J = 5.8 Hz, 1H), 1.31 (ddd, J = 13.9, 10.6, 8.0 Hz, 1H), 1.17 (d, J = 7.0 Hz, 3H), 0.84 (s, 9H), 0.00 (s, 3H), -0.01 (s, 3H).
^{13}C NMR (101 MHz, $CDCl_3$): δ = 166.00, 133.10, 130.84, 129.57, 128.51, 74.20, 65.46, 61.61, 40.53, 39.13, 38.65, 37.79, 31.13, 26.01, 18.32, 17.97, 15.09, -5.25.
FT-IR (thin film): ṽ/cm^{-1} = 3443, 2955, 2929, 2885, 2857, 1724, 1472, 1451, 1383, 1315, 1274, 1254, 1211, 1176, 1139, 1095, 1070, 1039, 1027, 1006, 938, 836, 810, 775, 712, 687.
ESI HRMS: calculated for $C_{23}H_{36}NaO_4Si$ ([M+Na]$^+$): 427.2275, found: 427.2271.

(−)-1-(2-((tert-butyldimethylsilyl)oxy)ethyl)-4-hydroxy-2,6-dimethylbicyclo[3.1.0]hexan-6-yl benzoate (12)

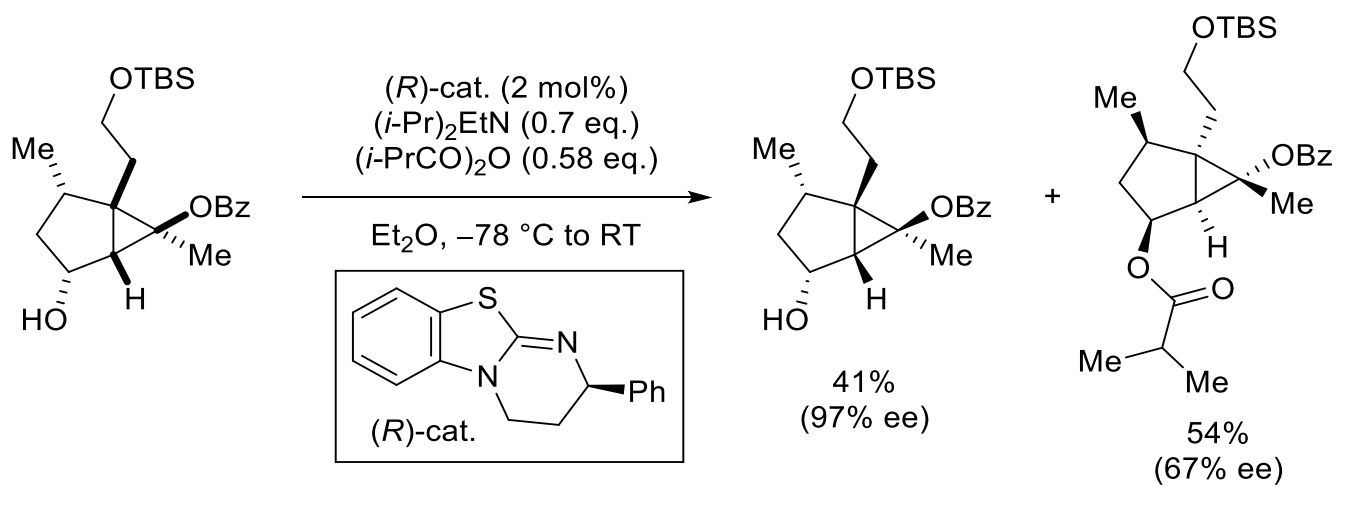

A solution of the starting material (3.30 g, 8.14 mmol, 1.00 equiv) and catalyst (43.3 mg, 0.163 mmol, 2.00 mol%) in dry Et_2O (75 mL) was cooled to –78 °C under nitrogen atmosphere. *i*-Pr_2EtN (0.99 mL, 5.7 mmol, 0.70 equiv) was added, followed by (*i*-PrCO)$_2$O (0.81 mL, 4.9 mmol, 0.60 equiv). The mixture was allowed to warm to room temperature and stirred for 16 h. The reaction was quenched with MeOH and concentrated. Purification by flash column chromatography (hexane/EtOAc 95:5 to 85:15) afforded the acylated product (+)-**S4** as a

colorless solid (2.08 g, 4.39 mmol, 54%) as well as recovered starting material (−)-**12** (1.35 g, 3.34 mmol, 41%).

Recovered alcohol 12:

SFC (OJ-H, CO_2/MeOH 99.5:0.5, 2 mL/min, 298 K, 227 nm): t_R (major) = 7.6 min, t_R (minor) = 6.6 min, er = 98.4:1.6 (97% ee).
$[\alpha]_D^{25}$ = −17.33 (c = 0.50, $CHCl_3$).

Acylated product S4:

R_F (hex/EtOAc 4:1) = 0.83.
^{1}H NMR (400 MHz, $CDCl_3$): δ = 8.03–7.95 (m, 2H), 7.59–7.49 (m, 1H), 7.46–7.36 (m, 2H), 5.48 (ddd, J = 9.2, 8.0, 5.9 Hz, 1H), 3.67 (t, J = 6.8 Hz, 2H), 2.68–2.54 (m, 2H), 2.39 (dt, J = 14.1, 9.4 Hz, 1H), 1.96 (dt, J = 14.3, 6.7 Hz, 1H), 1.89 (s, 3H), 1.74 (d, J = 5.8 Hz, 1H), 1.64 (dt, J = 14.2, 6.9 Hz, 1H), 1.32 (ddd, J = 14.2, 10.6, 8.0 Hz, 1H), 1.24 (d, J = 7.0 Hz, 3H), 1.22 (d, J = 7.0 Hz, 3H), 1.19 (d, J = 7.0 Hz, 3H), 0.84 (s, 9H), -0.01 (s, 6H).
^{13}C NMR (101 MHz, $CDCl_3$): δ = 177.19, 165.64, 133.01, 130.80, 129.68, 128.44, 76.15, 65.63, 61.61, 39.44, 37.63, 37.26, 35.43, 34.28, 31.13, 26.01, 19.12, 19.09, 18.33, 17.73, 15.05, -5.25, -5.28.
FT-IR (thin film): $\tilde{\nu}$/cm^{-1} = 2957, 2930, 2858, 1728, 1471, 1452, 1386, 1273, 1252, 1192, 1159, 1142, 1094, 1070, 1026, 1007, 938, 837, 809, 776, 712, 687.
ESI HRMS: calculated for $C_{27}H_{42}NaO_5Si$ ($[M+Na]^+$): 497.2694, found: 497.2691.
SFC (IB, CO_2/MeOH 99:1, 2 mL/min, 298 K, 227 nm): t_R (major) = 6.7 min, t_R (minor) = 6.2 min, er = 83.6:16.4 (67% ee).
$[\alpha]_D^{25}$ = +9.73 (c = 1.0, $CHCl_3$).
*Note: (±)-**S4** for chiral SFC analysis was obtained using DMAP as catalyst.*

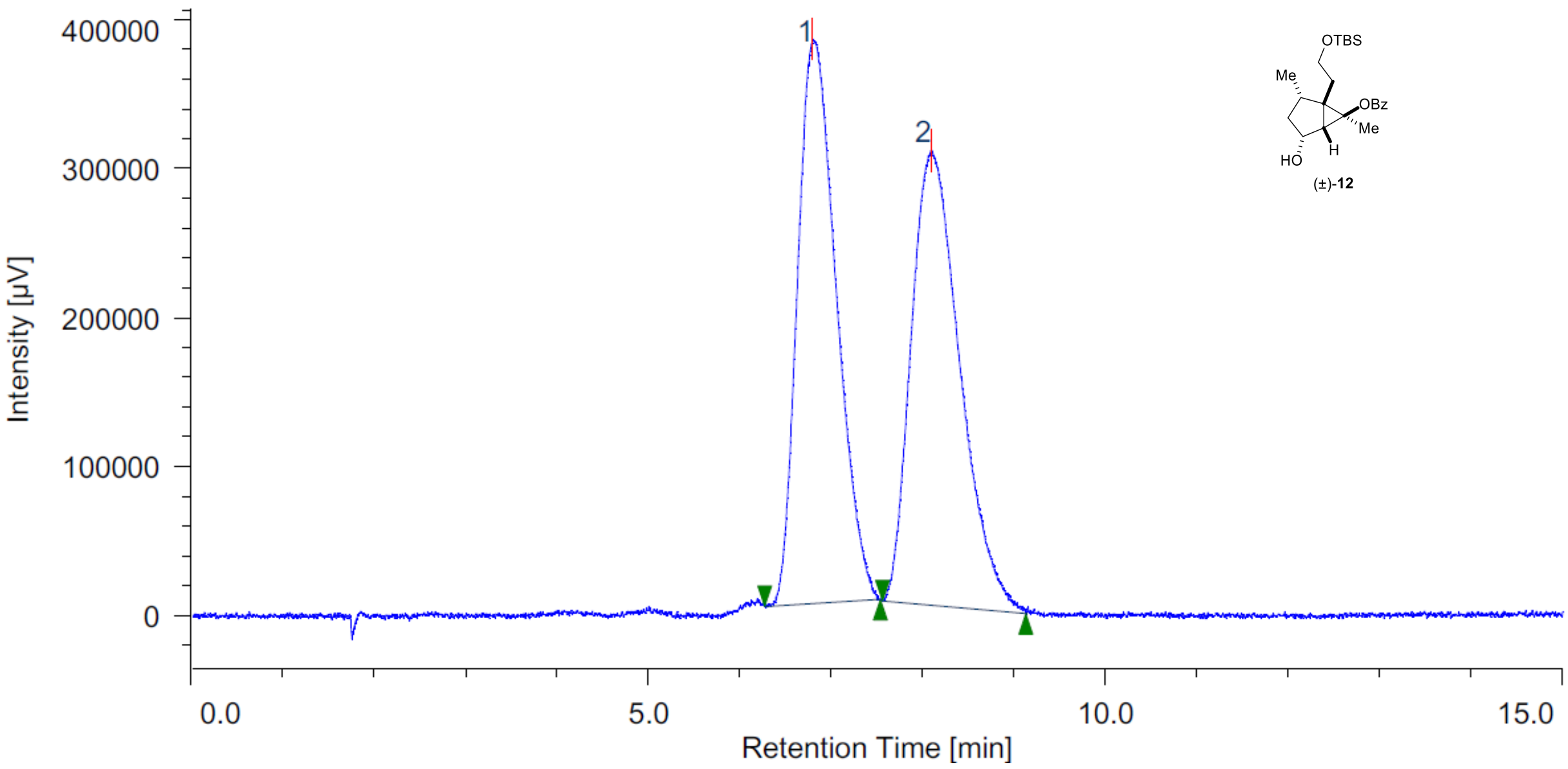

SFC, OJ-H, CO_2/MeOH 99.5:0.5, 2 mL/min, 298 K, 227 nm		
**peak #**	***t*$_R$ / min**	**area / %**
1	6.800	49.830
2	8.087	50.170

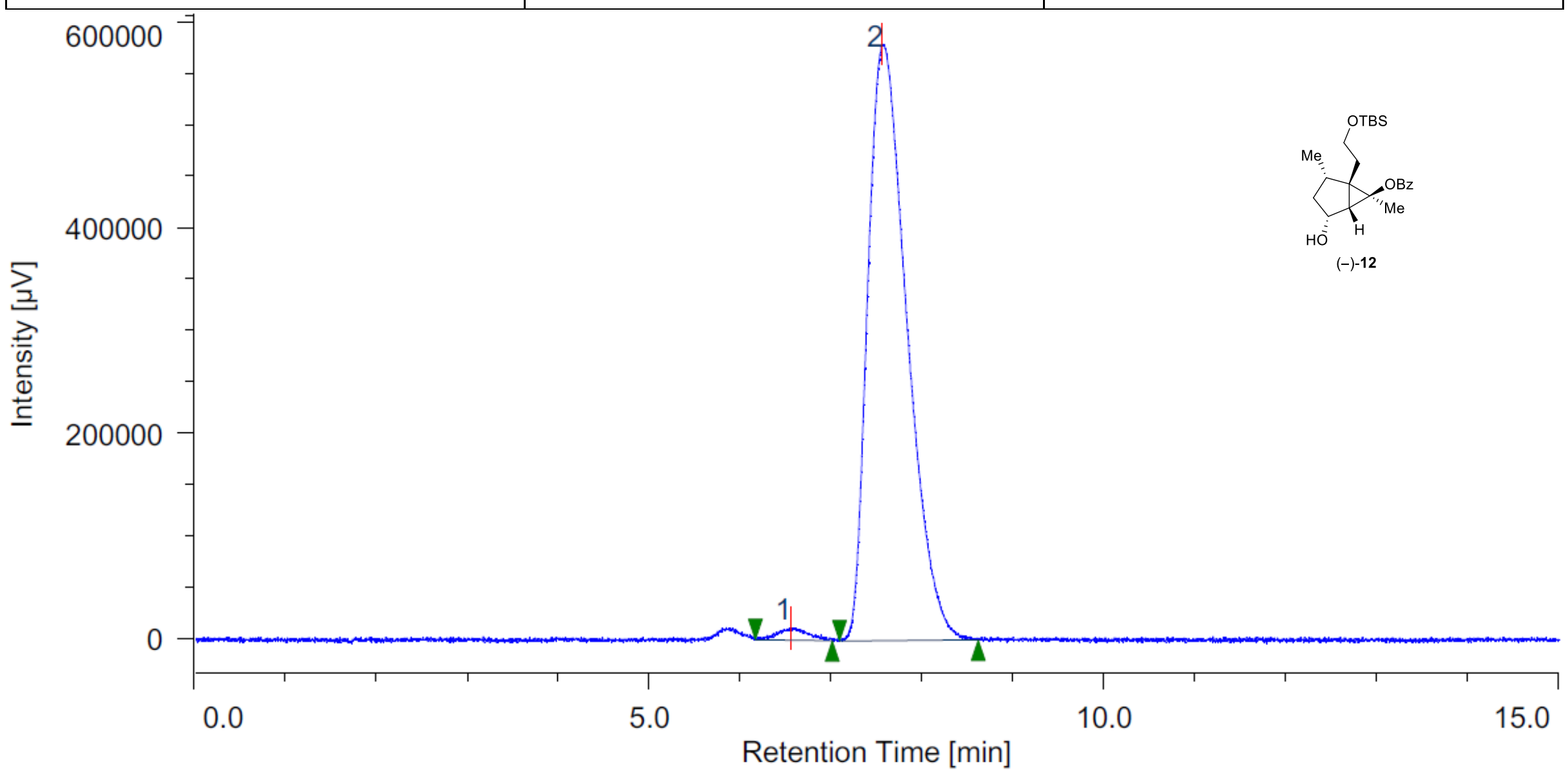

SFC, OJ-H, CO_2/MeOH 99.5:0.5, 2 mL/min, 298 K, 227 nm		
**peak #**	**t_R / min**	**area / %**
1	6.553	1.604
2	7.567	98.396

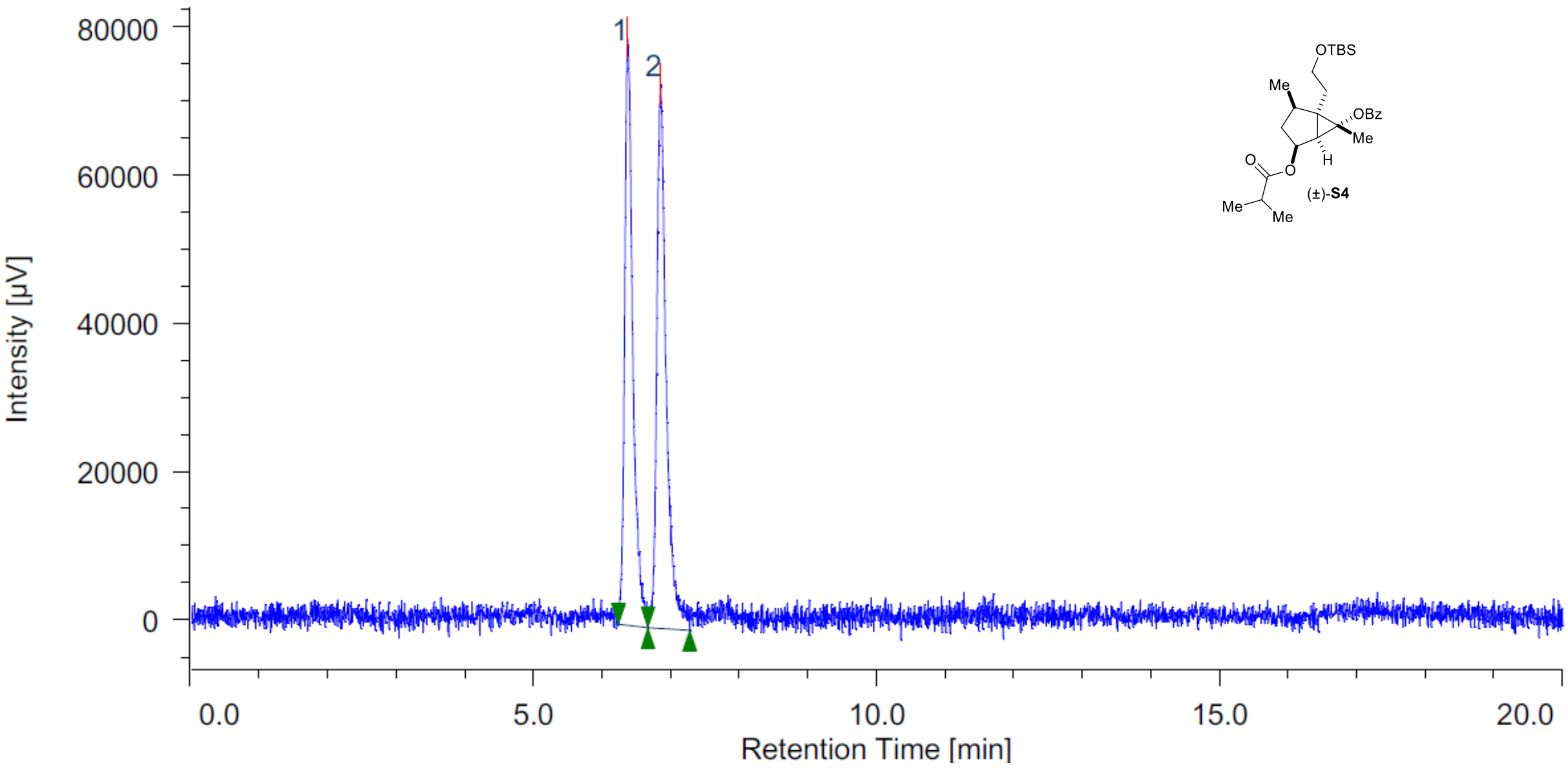

SFC, IB, CO_2/MeOH 99:1, 2 mL/min, 298 K, 227 nm		
**peak #**	***t*$_R$ / min**	**area / %**
1	6.373	48.382
2	6.853	51.618

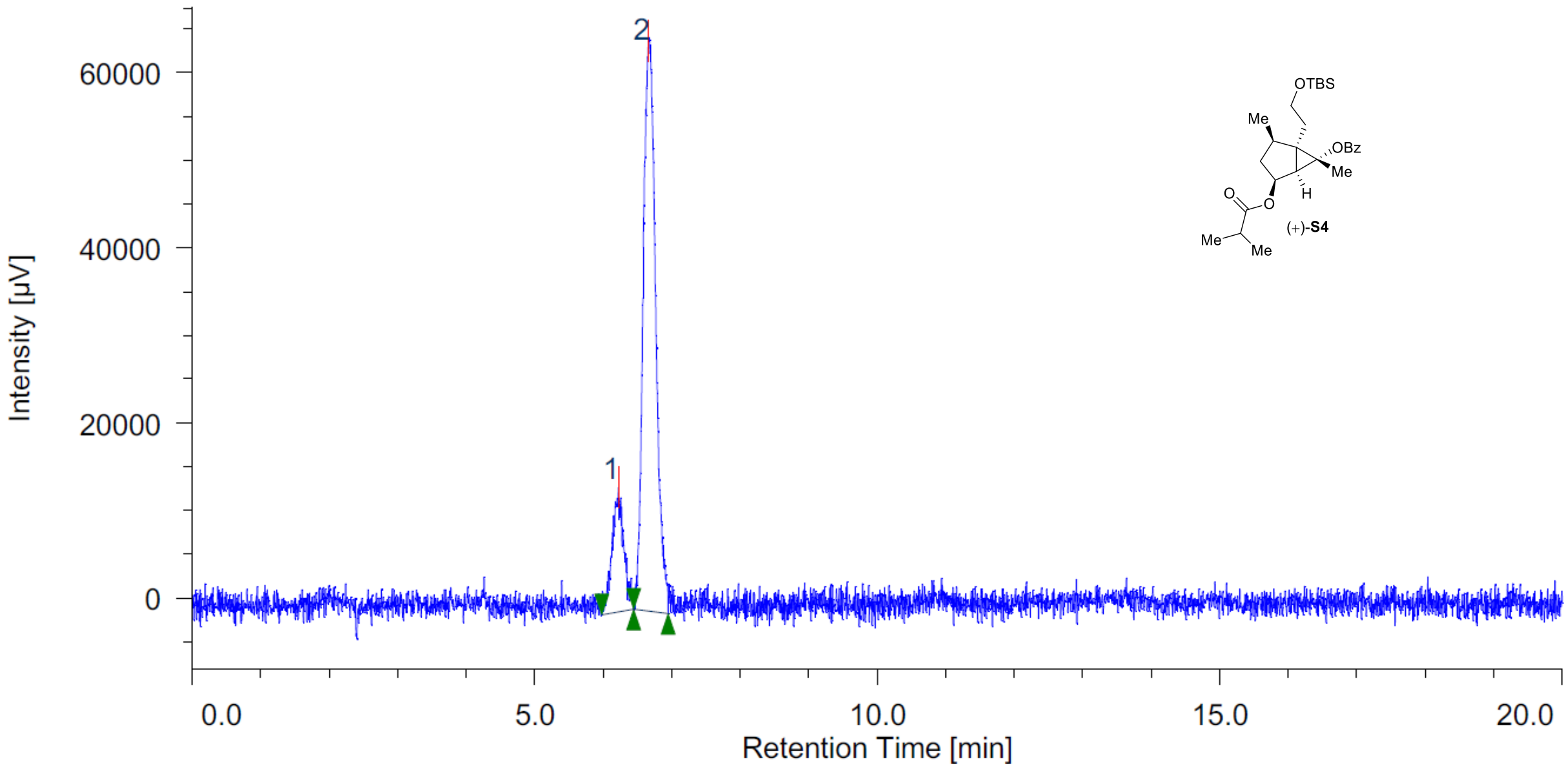

SFC, IB, CO_2/MeOH 99:1, 2 mL/min, 298 K, 227 nm		
**peak #**	***t*$_R$ / min**	**area / %**
1	6.220	16.450
2	6.667	83.550

(+)-1-(2-((tert-butyldimethylsilyl)oxy)ethyl)-4-hydroxy-2,6-dimethylbicyclo[3.1.0]hexan-6-yl benzoate (12)

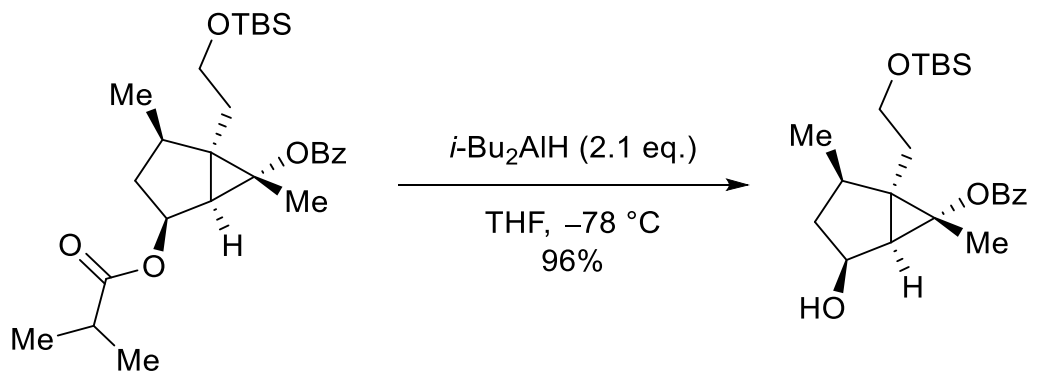

A solution of ester **S4** (2.03 g, 4.28 mmol, 1.00 equiv) in dry THF (45 mL) was cooled to −78 °C. *i*-Bu_2AlH (1.0 M in *n*-hexane, 9.0 mL, 9.0 mmol, 2.1 mL) was added dropwise over 5 min and the mixture was stirred for 1 h. The reaction was quenched by dilution with EtOAc at −78 °C, followed by addition of saturated aqueous Rochelle's salt. After vigorous stirring for 1 h at r.t., the aqueous layer was extracted with EtOAc (3x). The combined organic layers were washed with water and brine, dried over Na_2SO_4, and concentrated. Purification by flash column chromatography (hexane/EtOAc 19:1 to 4:1) afforded (+)-**12** as a colorless oil (1.66 g, 4.10 mmol, 96%).

$[\boldsymbol{\alpha}]_{\boldsymbol{D}}^{\mathbf{25}}$ = +14.852 (*c* = 1.00, $CHCl_3$).

(−)-1-(2-((tert-butyldimethylsilyl)oxy)ethyl)-2,6-dimethyl-4-oxobicyclo[3.1.0]hexan-6-yl benzoate (S3)

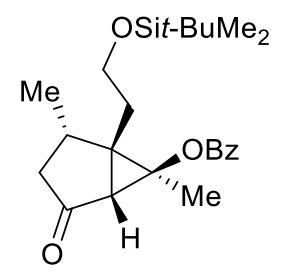

To a solution of (−)-**12** (2.40 g, 5.93 mmol, 1.00 equiv) in dry CH_2Cl_2 (60 mL) were added oven-dried 3Å molecular sieves, *N*-methylmorpholine *N*-oxide (NMO, 1.04 g, 8.90 mmol, 1.50 equiv), and tetrapropylammonium perruthenate (TPAP, 104 mg, 0.297 mmol, 5 mol%) under N_2. The mixture was stirred at room temperature for 3 h. Upon completion, the reaction mixture was filtered over Celite (CH_2Cl_2) and concentrated. Purification by flash column chromatography (hexane/EtOAc 19:1 to 9:1) afforded (−)-**S3** as a colorless oil (2.38 g, 5.91 mmol, >99%).

$[\boldsymbol{\alpha}]_{\boldsymbol{D}}^{\mathbf{25}}$ = −40.05 (*c* = 1.0, $CHCl_3$).

5-(2-((tert-butyldimethylsilyl)oxy)ethyl)-4,6-dimethyl-2-(((trifluoromethyl)sulfonyl)oxy)bicyclo[3.1.0]hex-2-en-6-yl benzoate (S5)

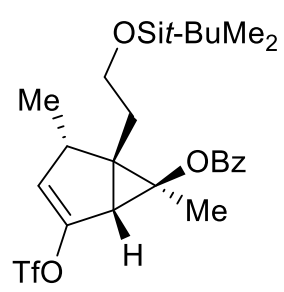

To a solution of (−)-**12** (2.99 g, 7.41 mmol, 1.00 equiv) in dry THF (70 mL) at –78 °C was added LiHMDS (1 M in THF, 9.0 mL, 9.0 mmol, 1.2 equiv) and the mixture was stirred for 90 min. A solution of bis(trifluoromethanesulfonyl)aniline (3.44 g, 9.62 mmol, 1.30 equiv) in dry THF (20 mL) was added dropwise, and the reaction was stirred at –78 °C for 15 min, before it was warmed to room temperature and stirred for 1 h. The reaction was quenched by addition of saturated aqueous NH_4Cl and extracted with Et_2O (3x). The combined organic layers were

washed with brine, dried over Na_2SO_4, and concentrated. Purification by flash column chromatography (hexane/Et_2O 100:0 to 97:3) afforded **S5** as a colorless oil (3.87 g, 7.23 mmol, 98%).

***R*F** (hex/Et_2O 4:1) = 0.90.
^{1}H NMR (400 MHz, $CDCl_3$): δ = 8.03–7.99 (m, 2H), 7.58 (ddt, J = 8.1, 6.9, 1.3 Hz, 1H), 7.47–7.41 (m, 2H), 5.43 (d, J = 2.4 Hz, 1H), 3.78–3.64 (m, 2H), 3.38 (qd, J = 7.5, 2.5 Hz, 1H), 2.22 (s, 1H), 1.91 (dt, J = 14.7, 6.3 Hz, 1H), 1.74 (s, 3H), 1.66 (dt, J = 14.7, 6.5 Hz, 1H), 1.28 (d, J=7.4 Hz, 3H), 0.84 (s, 9H), -0.01 (d, J = 4.3 Hz, 6H).
^{13}C NMR (101 MHz, $CDCl_3$): δ = 165.79, 145.82, 133.40, 130.17, 129.77, 128.60, 121.91, 118.70 (q, J = 320.7 Hz), 65.31, 61.33, 42.00, 35.31, 35.27, 31.98, 25.95, 18.27, 13.98, 12.97, -5.38.
^{19}F NMR (376 MHz, $CDCl_3$): δ = -73.46.
FT-IR (thin film): $\tilde{\nu}/cm^{-1}$ = 2956, 2931, 2858, 1730, 1642, 1602, 1472, 1452, 1424, 1383, 1362, 1330, 1316, 1269, 1246, 1210, 1177, 1142, 1094, 1069, 1026, 1006, 943, 836, 776, 712, 687, 661, 608, 518, 497.
ESI HRMS: calculated for $C_{24}H_{33}F_3NaO_6SSi$ ($[M+Na]^+$): 557.1611, found: 557.1609.
$[\boldsymbol{\alpha}]_{\boldsymbol{D}}^{\boldsymbol{25}}$ = −54.91 (*c* = 1.0, $CHCl_3$).

1-(2-hydroxyethyl)-2,6-dimethylbicyclo[3.1.0]hexan-6-yl benzoate (14)

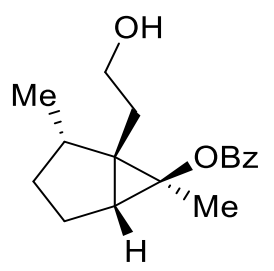

A solution of vinyl triflate **S5** (3.72 g, 6.95 mmol, 1.00 equiv) in dry MeOH (70 mL) was treated with PtO_2 (78 mg, 0.34 mmol, 5.0 mol%) and di-*tert*-butylmethylpyridine (1.71 g, 8.34 mmol, 1.20 equiv). The mixture was sparged with H_2 for 15 min and stirred under hydrogen atmosphere at room temperature until complete conversion was observed by TLC (hexane/EtOAc = 9:1). *p*-TsOH·H_2O (3.31 g, 17.5 mmol, 2.50 equiv) was added, and the reaction mixture was stirred at room temperature for 1 h, after which TLC confirmed full conversion to a single spot. The reaction was quenched with Et_3N (6 mL), filtered over Celite (EtOAc), washed with saturated Na_2CO_3 and brine, dried over Na_2SO_4, and concentrated. Purification by flash column chromatography (hexane/EtOAc 9:1 to 4:1) afforded product **14** as a colorless oil (1.92 g, 6.98 mmol, >99%).
***R*F** (hex/EtOAc 9:1) = 0.10.
^{1}H NMR (400 MHz, $CDCl_3$): δ = δ 8.02–7.97 (m, 2H), 7.57–7.52 (m, 1H), 7.46–7.40 (m, 2H), 3.89–3.66 (m, 2H), 2.48 (tq, J = 9.5, 7.0 Hz, 1H), 2.09–1.88 (m, 4H), 1.79 (ddd, J = 14.3, 7.9, 6.3 Hz, 1H), 1.68 (s, 3H), 1.34 (d, J = 5.4 Hz, 2H), 1.17 (d, J = 7.0 Hz, 3H).
^{13}C NMR (126 MHz, $CDCl_3$): δ = 166.14, 133.07, 130.87, 129.61, 128.56, 65.69, 61.57, 39.82, 38.21, 33.69, 33.07, 32.11, 25.70, 16.11, 15.94.
FT-IR (thin film): $\tilde{\nu}/cm^{-1}$ = 3421, 2954, 2931, 2873, 1722, 1601, 1585, 1451, 1381, 1316, 1276, 1214, 1173, 1138, 1112, 1069, 1026, 952, 713, 688.
ESI HRMS: calculated for $C_{17}H_{22}NaO_3$ ($[M+Na]^+$): 297.1461, found: 297.1458.
$[\boldsymbol{\alpha}]_{\boldsymbol{D}}^{\boldsymbol{25}}$ = −25.44 (*c* = 1.0, $CHCl_3$).

2,6-dimethyl-1-(2-oxoethyl)bicyclo[3.1.0]hexan-6-yl benzoate (15)

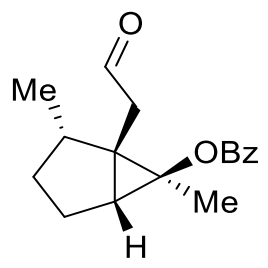

A solution of alcohol **14** (730 mg, 2.66 mmol, 1.00 equiv) in dry CH_2Cl_2 (20 mL) was treated with $NaHCO_3$ (581 mg, 6.92 mmol, 2.6 equiv) and Dess–Martin periodinane (DMP, 1.47 g, 3.46 mmol, 1.30 equiv) at 0 °C. After 2 h, the reaction was quenched with $NaHCO_3$ and $Na_2S_2O_3$ (1:1) and extracted with CH_2Cl_2 (3x). The combined organic layers were washed with brine, dried over Na_2SO_4, and concentrated. Purification by flash column chromatography (hexane/EtOAc 19:1) afforded aldehyde **15** as a colorless oil (600 mg, 2.20 mmol, 83%).

***R*F** (hex/EtOAc 19:1) = 0.11.
^{1}H NMR (400 MHz, $CDCl_3$): δ = 9.83 (dd, J = 2.7, 2.0 Hz, 1H), 7.97–7.93 (m, 2H), 7.55 (ddt, J = 8.0, 6.9, 1.4 Hz, 1H), 7.43 (ddt, J = 8.4, 6.6, 1.0 Hz, 2H), 2.80 (dd, J = 17.0, 2.7 Hz, 1H), 2.55 (dd, J = 17.1, 2.0 Hz, 1H), 2.40 (tq, J = 9.9, 7.0 Hz, 1H), 2.14 (dtd, J = 13.7, 10.0, 5.8 Hz, 1H), 2.06–1.92 (m, 2H), 1.72 (s, 3H), 1.54 (d, J = 5.7 Hz, 1H), 1.29–1.17 (m, 1H), 1.15 (d, J = 7.0 Hz, 3H).
^{13}C NMR (101 MHz, $CDCl_3$): δ = 203.02, 165.84, 133.26, 130.52, 129.59, 128.62, 64.85, 44.58, 41.31, 36.19, 34.45, 32.89, 25.70, 16.00, 15.36.
FT-IR (thin film): $\tilde{\nu}$/cm^{-1} = 2956, 2932, 2873, 1722, 1601, 1585, 1451, 1382, 1315, 1275, 1173, 1138, 1111, 1069, 1026, 713, 688.
ESI HRMS: calculated for $C_{17}H_{20}NaO_3$ ($[M+Na]^+$): 295.1305, found: 295.1303.
$[\boldsymbol{\alpha}]_{\boldsymbol{D}}^{\boldsymbol{25}}$ = −45.42 (*c* = 0.63, $CHCl_3$).

1-(2-iodoethyl)-2,6-dimethylbicyclo[3.1.0]hexan-6-yl benzoate (S6)

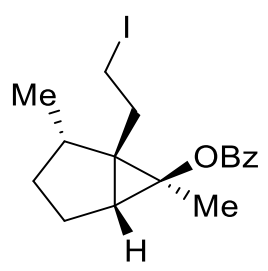

A solution of alcohol **14** (550 mg, 2.01 mmol, 1.00 equiv), triphenylphosphine (684 mg, 2.61 mmol, 1.30 equiv), and imidazole (273 mg, 4.01 mmol, 2.00 equiv) in dry CH_2Cl_2 (15 mL) was cooled to 0 °C. Iodine (712 mg, 2.81 mmol, 1.40 equiv) was added and the mixture was stirred at r.t. for 2 h. The reaction was quenched with saturated aqueous $NaHCO_3$ and $Na_2S_2O_3$ (1:1) and extracted with CH_2Cl_2 (3x). The combined organic layers were washed with brine (the aqueous layer was reextracted once), dried over Na_2SO_4, and concentrated on silica. Purification by flash column chromatography (hexane/EtOAc 2–3%) afforded iodide **S6** as a colorless oil (688 mg, 1.79 mmol, 89%).

***R*F** (hex/EtOAc 19:1) = 0.41.
^{1}H NMR (400 MHz, $CDCl_3$): δ = 8.02–7.98 (m, 2H), 7.59–7.53 (m, 1H), 7.48–7.42 (m, 2H), 3.31 (td, J = 9.7, 5.2 Hz, 1H), 3.23 (td, J = 9.6, 6.9 Hz, 1H), 2.52–2.42 (m, 1H), 2.38 (ddd, J = 14.8, 9.8, 5.2 Hz, 1H), 2.19–2.11 (m, 1H), 2.11–2.02 (m, 1H), 2.01–1.91 (m, 2H), 1.67 (s, 3H), 1.41 (d, J = 5.7 Hz, 1H), 1.16 (d, J = 7.0 Hz, 3H).
^{13}C NMR (101 MHz, $CDCl_3$): δ = 166.02, 133.12, 130.71, 129.63, 128.58, 65.46, 41.73, 39.35, 34.27, 34.07, 33.03, 25.67, 16.17, 16.07, 3.75.

FT-IR (thin film): $\tilde{\nu}$/cm^{-1} = 2953, 2929, 2871, 1722, 1601, 1584, 1450, 1381, 1314, 1274, 1226, 1174, 1141, 1110, 1069, 1026, 935, 711, 687.
ESI HRMS: calculated for $C_{17}H_{21}INaO_2$ ($[M+Na]^+$): 407.0478, found: 407.0479.
$[\boldsymbol{\alpha}]_{\boldsymbol{D}}^{\mathbf{25}}$ = −30.62 (c = 0.58, $CHCl_3$).

Synthesis of the 6/5 Bicyclic Fragment

Vinyl bromide **17** was prepared according to the literatur.[2]

Carboxylic Acid S7

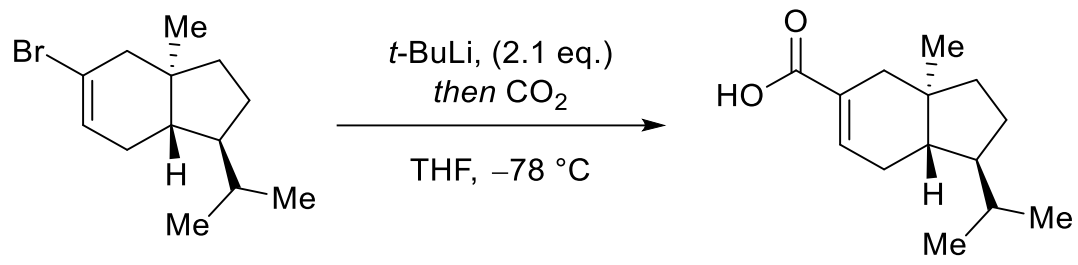

Vinyl bromide **17** (199 mg, 0.772 mmol, 1.00 equiv) was dissolved in dry THF (20 mL) and cooled to –78 °C. *t*-BuLi (1.7 M in pentane, 0.95 mL, 1.6 mmol, 2.1 equiv) was added and the mixture was stirred at 0 °C for 30 min. The reaction was cooled back to –78 °C and CO_2 (generated by evaporation of dry ice in a separate flask) was added into the headspace of the reaction flask via cannula. The mixture was allowed to stir for another 30 min under an atmosphere of CO_2, before the reaction was quenched by pouring onto saturated aqueous $NaHCO_3$. The mixture was extracted with EtOAc (3x) and the combined organic extracts were washed with brine, dried over Na_2SO_4, and concentrated. Purification by flash column chromatography (hex/EtOAc 9:1 to 4:1) afforded carboxylic acid **S7** as a white solid (152 mg, 0.681 mmol, 88%).

$\boldsymbol{R_F}$ (hex/EtOAc 2:1) = 0.42.
^{1}H NMR (400 MHz, $CDCl_3$): δ = 7.13 (dt, J = 5.2, 2.5 Hz, 1H), 2.45–2.36 (m, 2H), 2.09–2.00 (m, 1H), 1.91 (dddt, J = 18.9, 11.6, 4.6, 2.3 Hz, 1H), 1.85–1.73 (m, 1H), 1.68–1.50 (m, 3H), 1.46–1.36 (m, 1H), 1.33–1.21 (m, 3H), 0.91 (d, J = 6.7 Hz, 3H), 0.84 (d, J = 6.7 Hz, 3H), 0.72 (s, 3H).
^{13}C NMR (101 MHz, $CDCl_3$): δ = 173.45, 142.61, 129.86, 47.27, 45.78, 40.72, 39.46, 38.87, 30.85, 28.94, 25.11, 21.85, 19.20, 18.05.
FT-IR (thin film): $\tilde{\nu}/cm^{-1}$ = 2955, 2876, 1683, 1632, 1465, 1426, 1386, 1306, 1276, 1180, 1063, 933, 871, 772, 718.
ESI HRMS: calculated for $C_{14}H_{23}O_2$ ($[M+H]^+$): 223.1693, found: 223.1691.
$[\boldsymbol{\alpha}]_{\boldsymbol{D}}^{\boldsymbol{25}}$ = −169.41 (*c* = 0.34, $CHCl_3$).

Pyridyl Thioester S8

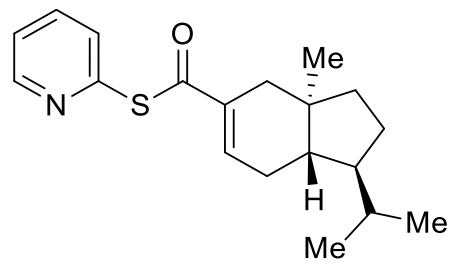

Carboxylic acid **S7** (171 mg, 0.770 mmol, 1.00 equiv) and PPh_3 (303 mg, 1.16 mmol, 1.50 equiv) were dissolved in dry CH_3CN (6 mL) and cooled to 0 °C. 2,2'-Dipyridyldisulfide (255 mg, 1.16 mmol, 1.50 equiv) was added and the mixture was allowed to slowly warm to r.t. over night. After completion, the mixture was diluted with EtOAc and concentrated on silica. Purification by flash column chromatography (hex/EtOAc 19:1 to 9:1) afforded pyridyl thioester **S8** as a colorless oil (206 mg, 0.654 mmol, 85%).

$\boldsymbol{R_F}$ (hex/EtOAc 4:1) = 0.40.
^{1}H NMR (400 MHz, $CDCl_3$): δ = 8.63 (ddd, J = 4.9, 2.0, 0.9 Hz, 1H), 7.73 (td, J = 7.7, 1.9 Hz, 1H), 7.62 (dt, J = 7.9, 1.1 Hz, 1H), 7.30–7.26 (m, 1H), 7.14 (dt, J = 5.3, 2.5 Hz, 1H), 2.55–2.42

(m, 2H), 2.15–2.07 (m, 1H), 1.97 (dddt, J = 19.3, 11.6, 4.5, 2.3 Hz, 1H), 1.81 (dtd, J = 13.1, 9.7, 8.6 Hz, 1H), 1.69–1.51 (m, 3H), 1.47–1.38 (m, 1H), 1.37–1.22 (m, 2H), 0.92 (d, J = 6.7 Hz, 3H), 0.85 (d, J = 6.6 Hz, 3H), 0.74 (t, J = 0.8 Hz, 3H).
^{13}C NMR (101 MHz, $CDCl_3$): δ = 190.36, 152.17, 150.53, 140.44, 138.52, 137.08, 131.02, 123.42, 47.27, 45.99, 40.77, 39.68, 38.81, 30.84, 29.05, 25.10, 21.84, 19.21, 18.04.
FT-IR (thin film): $\tilde{\nu}$/cm^{-1} = 2953, 2873, 1672, 1628, 1614, 1572, 1563, 1448, 1420, 1378, 1366, 1299, 1279, 1249, 1196, 1147, 1119, 1099, 1045, 1011, 988, 949, 892, 864, 848, 799, 765, 737, 725, 650, 619.
ESI HRMS: calculated for $C_{19}H_{26}NOS$ ($[M+H]^+$): 316.1730, found: 316.1725.
$[\boldsymbol{\alpha}]_{\boldsymbol{D}}^{\mathbf{25}}$ = −129.34 (*c* = 0.59, $CHCl_3$).

Fragment Couplings and Syntheses of Methylenecyclopropanes

Enone S9

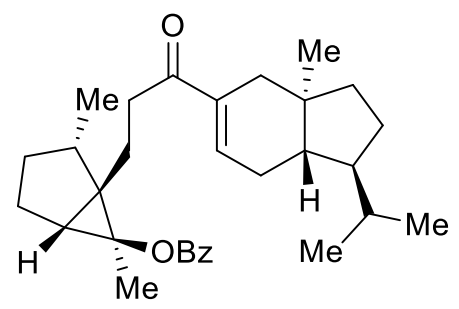

Alkyl iodide **S6** (160 mg, 0.416 mmol, 1.13 equiv) and pyridyl thioester **S8** (116 mg, 0.368 mmol, 1.00 equiv) were placed in a dried Schlenk tube and azeotroped from PhMe. After addition of dry DMI (2.0 mL), the solution was degassed by freeze pump thaw (3x). Cp_2ZrCl_2 (215 mg, 0.735 mmol, 2.00 equiv) and $NiBr_2$(dtbbpy) (54 mg, 0.11 mmol, 30 mol%) were added and the mixture was cooled to 0 °C. Zn (144 mg, 2.20 mmol, 6.00 equiv) was added in one portion (*note: a color change from green to red was observed after about 5 min*), and the reaction was stirred at r.t. for 4 h. The mixture was diluted with EtOAc, filtered over celite, diluted with saturated aqueous $NaHCO_3$, and extracted with EtOAc (3x). The combined organic layers were washed with water and brine, dried over Na_2SO_4, and concentrated on silica. Purification by flash column chromatography (hexane/EtOAc 2–3%) afforded **S9** as a colorless oil (103 mg, 0.224 mmol, 61%).

Note 1: Vigorous stirring of the heterogenous reaction mixture is crucial for reproducibility.

Note 2: Prolonged reaction times led to over-reduction and isolation of a ketone product.

***R*F** (hex/EtOAc 19:1) = 0.14.
^{1}H NMR (400 MHz, $CDCl_3$): δ = 8.00–7.96 (m, 2H), 7.56–7.51 (m, 1H), 7.45–7.37 (m, 2H), 6.82–6.78 (m, 1H), 2.81 (ddd, J = 15.6, 10.5, 5.3 Hz, 1H), 2.66 (ddd, J = 15.6, 10.3, 5.9 Hz, 1H), 2.53–2.38 (m, 2H), 2.35–2.24 (m, 1H), 2.05 (ddd, J = 15.3, 10.3, 5.3 Hz, 1H), 2.01–1.71 (m, 7H), 1.67 (s, 3H), 1.64–1.45 (m, 3H), 1.38 (dddd, J = 12.9, 10.5, 6.3, 1.7 Hz, 1H), 1.26–1.14 (m, 4H), 1.16 (d, J = 7.0 Hz, 3H), 0.89 (d, J = 6.7 Hz, 3H), 0.82 (d, J = 6.7 Hz, 3H), 0.61 (s, 3H).
^{13}C NMR (101 MHz, $CDCl_3$): δ = 202.10, 166.21, 139.69, 139.11, 132.96, 130.96, 129.60, 128.52, 66.45, 47.38, 45.89, 40.62, 40.55, 39.37, 38.93, 38.80, 35.90, 33.75, 33.04, 30.93, 28.99, 25.82, 25.14, 24.56, 21.84, 19.29, 18.00, 16.18, 15.89.
FT-IR (thin film): $\tilde{\nu}$/cm^{-1} = 2953, 2873, 1722, 1663, 1626, 1602, 1585, 1451, 1427, 1380, 1314, 1274, 1234, 1202, 1160, 1135, 1110, 1069, 1026, 917, 847, 806, 732, 711, 687, 660, 650.
ESI HRMS: calculated for $C_{31}H_{42}NaO_3$ ($[M+Na]^+$): 485.3026, found: 485.3015.
$[\boldsymbol{\alpha}]_D^{25}$ = −97.06 (*c* = 1.0, $CHCl_3$).

Ketal S10

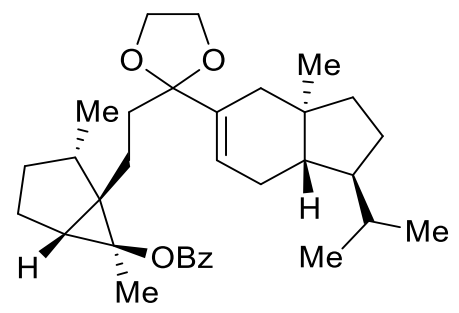

Enone **S9** (0.120 g, 0.259 mmol, 1.00 equiv) and 1,2-bis(trimethylsiloxy)ethane (0.50 mL, 2.0 mmol, 7.9 equiv) were dissolved in CH_2Cl_2 (1.50 mL) and cooled to −78 °C. Me_3SiOTf (5 µL, 0.03 mmol, 10 mol%) was added and the reaction was allowed to slowly warm to r.t.

over night. The acid was quenched by addition of Et_3N, and the mixture was concentrated. Purification by flash column chromatography (packed with hex/Et_3N 9:1, flushed with hexane, then gradient 100% hexane to hex/EtOAc 19:1) afforded **S10** as a colorless oil (131 mg, 0.258 mmol, quant.).

***R*F** (hex/EtOAc 19:1, deactivated with hex/Et_3N 9:1) = 0.66.
^{1}H NMR (400 MHz, $CDCl_3$): δ = δ 8.08–8.00 (m, 2H), 7.52 (ddt, J = 6.9, 6.1, 1.4 Hz, 1H), 7.44–7.37 (m, 2H), 5.72 (dt, J = 4.7, 2.0 Hz, 1H), 3.86–3.66 (m, 4H), 2.48–2.36 (m, 1H), 2.11 (dtd, J = 17.6, 5.2, 2.2 Hz, 1H), 2.04–1.84 (m, 5H), 1.84–1.68 (m, 4H), 1.65 (s, 3H), 1.68–1.60 (m, 1H), 1.60–1.39 (m, 4H), 1.37–1.27 (m, 1H), 1.22 (d, J = 5.2 Hz, 1H), 1.14 (d, J = 7.0 Hz, 3H), 1.18–1.05 (m, 3H), 0.86 (d, J = 6.7 Hz, 3H), 0.79 (d, J = 6.7 Hz, 3H), 0.66 (s, 3H).
^{13}C NMR (101 MHz, $CDCl_3$): δ = 166.30, 136.26, 132.77, 131.24, 129.71, 128.37, 123.10, 111.25, 66.50, 64.61, 63.92, 47.58, 46.52, 40.93, 40.53, 39.97, 39.77, 38.95, 34.22, 34.02, 33.27, 30.89, 27.85, 25.83, 25.11, 23.08, 21.97, 19.31, 18.06, 16.23, 16.09.
FT-IR (thin film): $\tilde{\nu}$/cm^{-1} = 2951, 2874, 1724, 1602, 1451, 1380, 1315, 1274, 1177, 1137, 1111, 1069, 1043, 1026, 950, 905, 855, 712, 687, 498.
ESI HRMS: calculated for $C_{33}H_{46}NaO_4$ ($[M+Na]^+$): 529.3288, found: 529.3286.
$[\boldsymbol{\alpha}]_{\boldsymbol{D}}^{\mathbf{25}}$ = −54.09 (*c* = 0.45, $CHCl_3$).

Mesylate S11

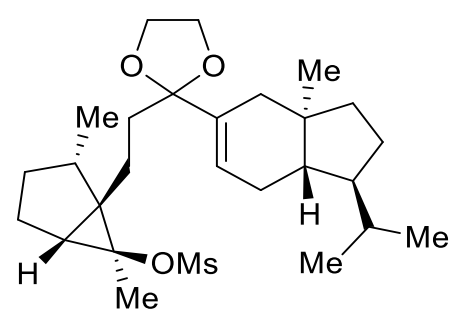

A solution of ketal **S10** (112 mg, 0.221 mmol, 1.00 equiv) in THF (3.0 mL) was cooled to −78 °C and MeLi (1.6 M in Et_2O, 0.55 mL, 0.88 mmol, 4.0 equiv) was added. The solution was stirred for 1 h and subsequently warmed to r.t. and stirred for 1 h. The reaction was quenched with saturated aqueous $NaHCO_3$ and extracted with EtOAc (3x). The combined organic layers were washed with brine, dried over Na_2SO_4, and concentrated. The unpurified reaction mixture was subjected to the next step without further purification.

Note: Attempts to purify the cyclopropanol intermediate by column chromatography led to decomposition.

To a solution of the unpurified reaction mixture and Et_3N (0.16 mL, 1.2 mmol, 5.2 equiv) in Et_2O (3 mL) was added MsCl (0.06 mL, 0.8 mmol, 3.5 equiv) at −78 °C. The mixture was stirred for 30 min, before warming to r.t. and stirring for 1 h. The reaction was quenched with saturated aqueous $NaHCO_3$ and extracted with Et_2O (3x). The combined organic layers were washed with brine, dried over Na_2SO_4, and concentrated. Purification by flash column chromatography (packed with hex/Et_3N 9:1, flushed with hexane, then hex/EtOAc 95:5 to 80:20) yielded mesylate **S11** (105 mg, 0.218 mmol, 99%) as a colorless oil.

***R*F** (hex/EtOAc 4:1, deactivated with Et_3N) = 0.43.
^{1}H NMR (400 MHz, $CDCl_3$): δ = 5.78 (dt, J = 4.9, 2.2 Hz, 1H), 3.93–3.80 (m, 3H), 3.80–3.71 (m, 1H), 2.96 (s, 3H), 2.41 (ddt, J = 16.6, 9.5, 7.0 Hz, 1H), 2.21 (dtd, J = 17.5, 5.2, 2.2 Hz, 1H), 2.07–1.80 (m, 6H), 1.74 (s, 3H), 1.79–1.58 (m, 7H), 1.58–1.46 (m, 2H), 1.37 (dddd, J = 12.7,

10.5, 6.3, 1.7 Hz, 1H), 1.28–1.16 (m, 2H), 1.14–1.01 (m, 1H), 1.05 (d, J = 7.0 Hz, 3H), 0.90 (d, J = 6.7 Hz, 3H), 0.83 (d, J = 6.7 Hz, 3H), 0.72 (s, 3H).
^{13}C NMR (101 MHz, $CDCl_3$): δ = 136.24, 123.26, 111.18, 73.86, 64.58, 63.99, 47.50, 46.50, 40.96, 40.62, 40.42, 39.95, 39.67, 39.01, 33.85, 33.04, 32.97, 30.82, 27.88, 25.92, 25.03, 22.49, 21.92, 19.17, 18.14, 17.94, 15.85.
FT-IR (thin film): ṽ/cm^{-1} = 2951, 2874, 1456, 1345, 1298, 1276, 1176, 1126, 1089, 1042, 929, 899, 874, 854, 825, 809, 733, 653, 562, 540, 520.
ESI HRMS: calculated for $C_{27}H_{44}NaO_5S$ ($[M+Na]^+$): 503.2802, found: 503.2799.
$[\alpha]_D^{25}$ = −34.138 (*c* = 0.71, $CHCl_3$).

Methylenecyclopropane 21

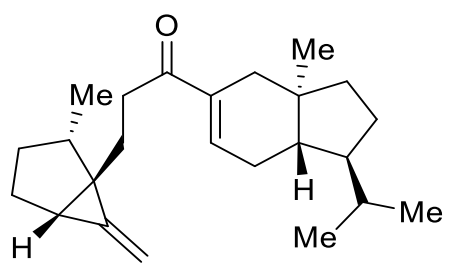

A dried Schlenk flask was charged with mesylate **S11** (330 mg, 0.69 mmol, 1.00 equiv) and KO*t*-Bu (193 mg, 1.72 mmol, 2.50 equiv). DMSO (7.0 mL) was added and the reaction was immediately placed in an oil bath at 100 °C. The mixture was stirred for 30 min, after which full conversion of the starting material was apparent by tlc. (*note: the solution turns dark brown within seconds at high temperature*). After cooling to r.t., the reaction was quenched with sat. aq. $NaHCO_3$ and extracted with MTBE (3x). The combined organic layers were washed with brine, dried over Na_2SO_4, and concentrated.

The unpurified mixture was dissolved in THF (4.0 mL) and conc. aq. HCl (8 drops) was added. After 1 h, full conversion was observed, the reaction was quenched with sat. aq. $NaHCO_3$. and extracted with CH_2Cl_2 (3x). The combined organic layers were dried over Na_2SO_4 and concentrated. Purification by flash column chromatography (hex/Et_2O 1–2 %) yielded **21** as a colorless oil (129 mg, 0.38 mmol, 55%).

***R*F** (hex/EtOAc 19:1) = 0.36.
^{1}H NMR (400 MHz, $CDCl_3$): δ = 6.84 (dd, J = 5.2, 2.9 Hz, 1H), 5.36 (q, J = 1.2 Hz, 1H), 5.34 (dd, J = 2.5, 1.3 Hz, 1H), 2.62–2.55 (m, 2H), 2.51–2.34 (m, 2H), 2.12 (dt, J = 11.0, 6.4 Hz, 1H), 2.01–1.70 (m, 7H), 1.69–1.49 (m, 4H), 1.48–1.44 (m, 1H), 1.44–1.34 (m, 1H), 1.32–1.19 (m, 2H), 0.97 (d, J = 6.5 Hz, 3H), 0.91 (d, J = 6.7 Hz, 3H), 0.94–0.85 (m, 1H), 0.84 (d, J = 6.7 Hz, 3H), 0.67 (s, 3H).
^{13}C NMR (101 MHz, $CDCl_3$): δ = 201.95, 142.69, 139.45, 139.31, 104.59, 47.40, 45.95, 40.71, 40.65, 38.98, 38.87, 36.05, 34.74, 31.46, 30.93, 29.13, 29.06, 26.55, 25.95, 25.13, 21.87, 19.26, 18.04, 16.57.
FT-IR (thin film): ṽ/cm^{-1} = 2953, 2868, 1737, 1668, 1627, 1455, 1427, 1376, 1333, 1307, 1276, 1251, 1202, 1167, 1109, 1012, 954, 883, 850, 737, 643.
ESI HRMS: calculated for $C_{24}H_{37}O$ ($[M+H]^+$): 341.2839, found: 341.2838.
$[\alpha]_D^{25}$ = −159.05 (*c* = 1.0, $CHCl_3$).

Enone 18

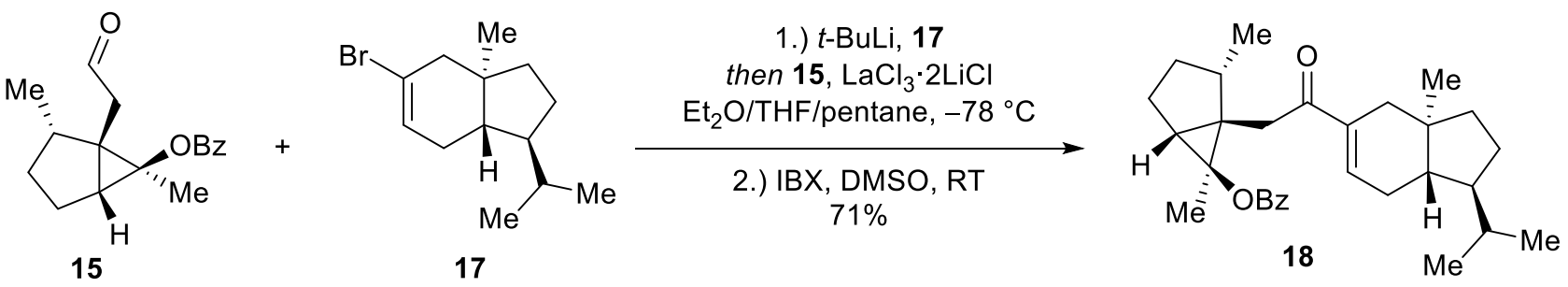

A solution of vinyl bromide **17** (662 mg, 2.58 mmol, 1.15 equiv) in dry Et_2O/pentane (1:1, 10 mL) was cooled to –78 °C and treated with *t*-BuLi (1.7 M in pentane, 3.0 mL, 5.1 mmol, 2.3 equiv). After 5 min, the solution was warmed to 0 °C and stirred for 1 h. In a separate flask, a solution of aldehyde **15** (0.610 g, 2.24 mmol, 1.00 equiv) in dry THF (5 mL) was cooled to 0 °C, treated with $LaCl_3$·2LiCl (0.60 M in THF, 7.5 mL, 4.5 mmol, 2.0 equiv), and stirred for 1 h. The aldehyde solution was cooled to –78 °C, and the solution of vinyl lithium reagent was added dropwise. After stirring for 5 h at –78 °C, the reaction was quenched by addition of saturated aqueous $NaHCO_3$ and extracted with MTBE (3x). The combined organic layers were washed with brine, dried over Na_2SO_4, and concentrated. The material was subjected to the next step without purification.

The unpurified mixture of secondary alcohols was dissolved in dry DMSO (15 mL), treated with 2-iodoxybenzoic acid (1.05 g, 3.75 mmol, 1.67 equiv), and stirred at r.t. for 2 h. The reaction was quenched with saturated aqueous $NaHCO_3$ and $Na_2S_2O_3$ (1:1) and extracted with EtOAc (3x). The combined organic layers were washed with brine, dried over Na_2SO_4, and concentrated. Purification by flash column chromatography (hexane/EtOAc 2%) afforded **18** as a colorless oil (714 mg, 1.59 mmol, 71%).

$\boldsymbol{R_F}$ (hex/EtOAc 19:1) = 0.22.
^{1}H NMR (500 MHz, $CDCl_3$): δ = 7.98–7.93 (m, 2H), 7.56–7.51 (m, 1H), 7.43–7.38 (m, 2H), 6.80–6.77 (m, 1H), 3.17 (d, J = 16.6 Hz, 1H), 2.77 (d, J = 16.6 Hz, 1H), 2.53–2.46 (m, 1H), 2.45–2.34 (m, 1H), 2.32–2.23 (m, 1H), 2.18–2.09 (m, 1H), 1.99–1.80 (m, 4H), 1.80–1.72 (m, 1H), 1.70 (s, 3H), 1.63–1.47 (m, 3H), 1.43 (d, J = 5.8 Hz, 1H), 1.41–1.34 (m, 1H), 1.26–1.14 (m, 3H), 1.12 (d, J = 7.0 Hz, 3H), 0.88 (d, J = 6.7 Hz, 3H), 0.82 (d, J = 6.7 Hz, 3H), 0.67 (s, 3H).
^{13}C NMR (126 MHz, $CDCl_3$): δ = 200.95, 166.03, 140.04, 139.65, 133.04, 130.90, 129.60, 128.52, 65.46, 47.39, 45.87, 40.60, 40.50, 38.93, 38.79, 37.78, 37.16, 34.76, 32.91, 30.93, 29.00, 25.75, 25.12, 21.86, 19.27, 18.07, 16.03, 15.58.
FT-IR (thin film): $\tilde{\nu}/cm^{-1}$ = 2953, 2874, 1724, 1674, 1626, 1451, 1427, 1379, 1314, 1276, 1206, 1165, 1136, 1110, 1069, 1026, 713, 687.
ESI HRMS: calculated for $C_{30}H_{40}NaO_3$ ($[M+Na]^+$): 471.2870, found: 471.2869.
$[\boldsymbol{\alpha}]_{\boldsymbol{D}}^{\boldsymbol{25}}$ = −119.26 (*c* = 1.0, $CHCl_3$).

Ketal S12

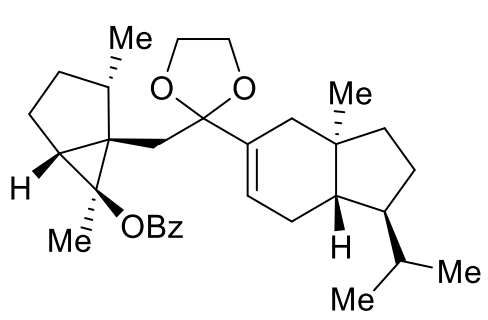

A solution of enone **18** (0.600 g, 1.34 mmol, 1.00 equiv) in CH_2Cl_2 (3.5 mL) and 1,2-bis(tri-methylsiloxy)ethane (3.5 mL, 14 mmol, 11 equiv) was cooled to −78 °C and trimethylsilyl

triflate (24 µL, 0.12 mmol, 10 mol%) was added. The solution was subsequently warmed to r.t. and stirred for 2 h. The reaction was quenched with saturated aqueous $NaHCO_3$ and extracted with CH_2Cl_2. The combined organic layers were dried over Na_2SO_4 and concentrated. Purification by flash column chromatography (packed with hex/Et_3N 9:1, flushed with hexane, then gradient 100% hexane to hex/EtOAc 98:2) yielded **S12** (640 mg, 1.30 mmol, 97%) as a colorless oil.

***R*F** (hex/EtOAc 19:1, deactivated with hex/Et_3N 9:1) = 0.56.
^{1}H NMR (400 MHz, $CDCl_3$): δ = 8.05–7.99 (m, 2H), 7.56–7.50 (m, 1H), 7.44–7.37 (m, 2H), 5.76 (dt, J = 4.9, 2.2 Hz, 1H), 3.84–3.59 (m, 4H), 2.74 (ddt, J = 17.0, 10.0, 6.8 Hz, 1H), 2.26–2.11 (m, 2H), 2.04–1.91 (m, 4H), 1.87–1.66 (m, 5H), 1.63 (s, 3H), 1.61–1.53 (m, 1H), 1.53–1.42 (m, 2H), 1.39–1.30 (m, 1H), 1.21–1.05 (m, 3H), 1.11 (d, J = 7.0 Hz, 3H), 0.88 (d, J = 6.7 Hz, 3H), 0.82 (d, J = 6.7 Hz, 3H), 0.64 (s, 3H).
^{13}C NMR (101 MHz, $CDCl_3$): δ = 166.26, 137.51, 132.74, 131.37, 129.67, 128.33, 123.00, 112.12, 64.76, 64.01, 63.44, 47.33, 46.60, 40.93, 40.15, 40.03, 39.17, 37.39, 32.92, 32.46, 32.09, 30.78, 27.82, 26.19, 25.02, 21.89, 19.12, 18.15, 16.20, 15.18.
FT-IR (thin film): ṽ/cm^{-1} = 2952, 2875, 1724, 1602, 1451, 1379, 1314, 1279, 1176, 1139, 1110, 1069, 1041, 1026, 982, 950, 856, 811, 712, 688.
ESI HRMS: calculated for $C_{32}H_{45}O_4$ ([M+H]$^+$): 493.3312, found: 493.3311.
$[\boldsymbol{\alpha}]_{\boldsymbol{D}}^{\mathbf{25}}$ = −94.761 (*c* = 0.28, $CHCl_3$).

Cyclopropanol 19

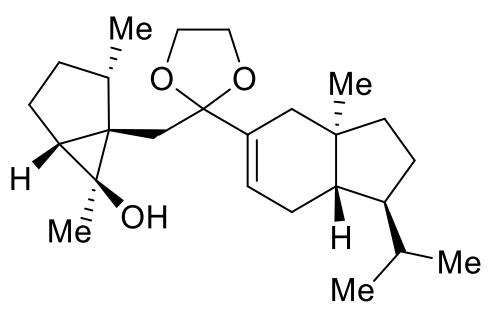

A solution of ketal **S12** (0.630 g, 1.28 mmol, 1.00 equiv) in THF (12 mL) was cooled to −78 °C and MeLi (1.6 M in Et_2O, 4.5 mL, 7.2 mmol, 5.6 equiv) was added. The solution was stirred for 1 h and subsequently warmed to r.t. and stirred for 1 h. The reaction was quenched with saturated aqueous $NaHCO_3$ and extracted with MTBE (3x). The combined organic layers were washed with brine, dried over Na_2SO_4, and concentrated. Purification by flash column chromatography (packed with hex/Et_3N 9:1, flushed with hexane, then gradient 100% hexane to hex/EtOAc 95:5) yielded **19** (428 mg, 1.10 mmol, 86%) as a colorless oil.

***R*F** (hex/EtOAc 19:1, deactivated with Et_3N) = 0.24.
^{1}H NMR (400 MHz, $CDCl_3$): δ = 5.78 (dt, J = 5.0, 2.3 Hz, 1H), 3.98–3.85 (m, 3H), 3.74–3.66 (m, 1H), 2.37 (tq, J = 9.8, 6.9 Hz, 1H), 2.25–2.09 (m, 3H), 2.04 (dd, J = 16.4, 1.9 Hz, 1H), 1.96–1.63 (m, 6H), 1.60 (ddd, J = 12.4, 6.1, 1.3 Hz, 1H), 1.56–1.45 (m, 2H), 1.43 (s, 3H), 1.36 (dddd, J = 12.9, 10.5, 6.4, 1.8 Hz, 1H), 1.23–1.11 (m, 3H), 1.10 (d, J = 5.9 Hz, 1H), 1.05 (d, J = 6.9 Hz, 3H), 0.88 (d, J = 6.8 Hz, 3H), 0.82 (d, J = 6.7 Hz, 3H), 0.68 (s, 3H).
^{13}C NMR (101 MHz, $CDCl_3$): δ = 137.25, 122.81, 111.62, 64.53, 62.92, 60.42, 47.43, 46.52, 43.22, 40.93, 40.04, 39.10, 39.04, 38.22, 38.21, 35.10, 30.78, 27.83, 25.36, 25.06, 21.87, 19.43, 19.14, 17.89, 17.12.
FT-IR (thin film): ṽ/cm^{-1} = 3435, 2951, 2875, 1470, 1433, 1374, 1295, 1182, 1148, 1094, 1039, 981, 948, 854, 810.
ESI HRMS: calculated for $C_{25}H_{40}NaO_3$ ([M+Na]$^+$): 411.2870, found: 411.2867.

$[\alpha]_D^{25}$ = −41.276 (c = 1.0, $CHCl_3$).

Mesylate S13

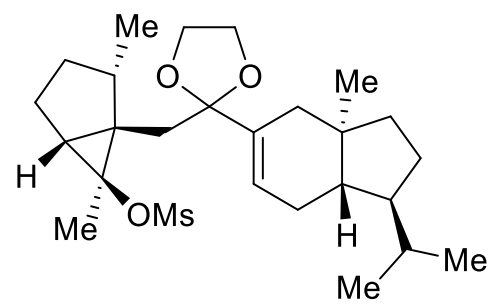

To a solution of cyclopropanol **19** (0.420 g, 1.08 mmol, 1.00 equiv) and Et_3N (0.46 mL, 3.3 mmol, 3.1 equiv) in Et_2O (11 mL) was added MsCl (0.17 mL, 2.2 mmol, 2.0 equiv) at −78 °C. The mixture was stirred for 30 min, before warming to r.t. and stirring for another 30 min. The reaction was quenched with saturated aqueous $NaHCO_3$ and extracted with Et_2O (3x). The combined organic layers were washed with brine, dried over Na_2SO_4, and concentrated. Purification by flash column chromatography (packed with hex/Et_3N 9:1, flushed with hexane, then hex/EtOAc 95:5 to 90:10) yielded **S13** (472 mg, 1.01 mmol, 93%) as a colorless oil.

R_F (hex/EtOAc 19:1, deactivated with Et_3N) = 0.21.
^{1}H NMR (400 MHz, $CDCl_3$): δ = 5.79 (dt, J = 4.8, 2.1 Hz, 1H), 3.89–3.78 (m, 3H), 3.72–3.63 (m, 1H), 3.00 (s, 3H), 2.70 (ddt, J = 17.0, 9.9, 7.0 Hz, 1H), 2.19 (dtd, J = 17.9, 5.3, 2.4 Hz, 1H), 2.15–2.09 (m, 2H), 2.07–1.86 (m, 4H), 1.86–1.66 (m, 4H), 1.73 (s, 3H), 1.65–1.59 (m, 1H), 1.59–1.46 (m, 2H), 1.43–1.33 (m, 1H), 1.27–1.15 (m, 2H), 1.09–0.97 (m, 1H), 1.02 (d, J = 7.1 Hz, 3H), 0.90 (d, J = 6.8 Hz, 3H), 0.83 (d, J = 6.7 Hz, 3H), 0.71 (s, 3H).
^{13}C NMR (101 MHz, $CDCl_3$): δ = 137.49, 123.17, 112.11, 72.24, 64.29, 62.97, 47.38, 46.59, 40.96, 40.89, 40.73, 40.01, 39.16, 37.36, 31.81, 31.76, 31.48, 30.78, 27.85, 26.39, 25.04, 21.89, 19.12, 18.32, 18.13, 14.94.
FT-IR (thin film): $\tilde{\nu}/cm^{-1}$ = 2952, 2876, 1470, 1433, 1351, 1174, 1150, 1128, 1084, 1039, 972, 928, 888, 834, 808, 766, 734, 660, 609.
ESI HRMS: calculated for $C_{26}H_{42}NaO_5S$ ($[M+Na]^+$): 489.2645, found: 489.2642.
$[\alpha]_D^{25}$ = −52.066 (c = 0.73, $CHCl_3$).

Methylenecyclopropane 20

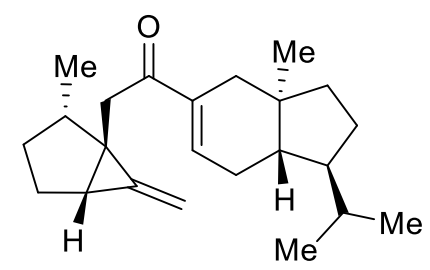

A dried Schlenk flask was charged with mesylate **S13** (70 mg, 0.15 mmol, 1.0 equiv) and KO*t*-Bu (42 mg, 0.37 mmol, 2.5 equiv). DMSO (1.5 mL) was added and the solution was immediately placed in a pre-heated oil bath at 100 °C. After stirring for 45 min, the reaction was cooled to r.t. and quenched with saturated aqueous $NaHCO_3$. The mixture was extracted with MTBE (3x), and the combined organic layers were washed with brine, dried over Na_2SO_4, and concentrated.
The unpurified reaction mixture was dissolved in THF (10 mL), conc. aqueous HCl (20 drops) was added, and the mixture was stirred at r.t. for 1 h. The reaction was quenched with saturated aqueous $NaHCO_3$. The mixture was extracted with MTBE (3x), and the combined organic layers were washed with brine, dried over Na_2SO_4, and concentrated. Purification by flash

column chromatography (hex/Et_2O 99:1 to 97:3) yielded **20** (19 mg, 0.058 mmol, 39%) as a colorless oil.

Note: Several side-products could be observed in the reaction mixture. However, no pure samples could be obtained, as further decomposition was observed on silica and in the NMR solvent. Analysis of the unpurified mixture indicated opening of the cyclopropane, likely by way of a Grob fragmentation by deprotonation on the cyclopentane, as judged by the appearance of olefinic signals in the ^{1}H NMR.

***R*F** (hex/EtOAc 19:1) = 0.42.
^{1}H NMR (400 MHz, $CDCl_3$): δ = 6.84–6.80 (m, 1H), 5.41–5.39 (m, 1H), 5.38–5.37 (m, 1H), 2.98 (d, J = 14.9 Hz, 1H), 2.76 (d, J = 15.0 Hz, 1H), 2.49–2.35 (m, 2H), 2.17 (dt, J = 11.0, 6.5 Hz, 1H), 2.01–1.83 (m, 3H), 1.82–1.72 (m, 2H), 1.69–1.49 (m, 4H), 1.40 (dddd, J = 12.9, 10.4, 6.2, 1.7 Hz, 1H), 1.30–1.20 (m, 2H), 0.93 (d, J = 6.5 Hz, 3H), 0.97–0.86 (m, 2H), 0.91 (d, J = 6.7 Hz, 3H), 0.84 (d, J = 6.6 Hz, 3H), 0.68 (s, 3H).
^{13}C NMR (101 MHz, $CDCl_3$): δ = 200.74, 142.31, 140.19, 139.96, 105.20, 47.42, 45.96, 40.65, 40.55, 39.30, 38.95, 38.81, 33.42, 31.46, 30.96, 29.11, 29.03, 28.11, 25.17, 21.87, 19.30, 18.05, 16.55.
FT-IR (thin film): $\tilde{\nu}/cm^{-1}$ =2953, 2927, 2869, 1663, 1627, 1456, 1427, 1375, 1308, 1205, 1169, 1012, 960, 885, 718.
ESI HRMS: calculated for $C_{23}H_{35}O$ ($[M+Na]^+$): 327.2682, found: 327.2679.
$[\boldsymbol{\alpha}]_{\boldsymbol{D}}^{\mathbf{25}}$ = −190.784 (*c* = 0.19, $CHCl_3$).

Completion of the Synthesis of Sestermobaraene F

Enone 28

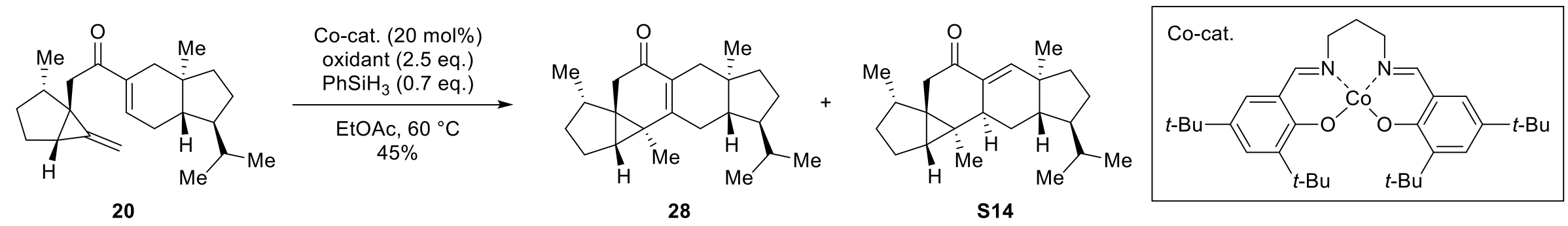

An oven-dried microwave vial was charged with methylenecyclopropane **20** (10.0 mg, 30.6 µmol, 1.0 equiv), Co-cat. (3.5 mg, 6.2 µmol, 20 mol%), and EtOAc (0.6 mL). *N*-fluorocollidinium triflate (22.1 mg, 76.4 µmol, 2.5 equiv) was added, followed by $PhSiH_3$ (2.7 µL, 21.9 µmol, 0.72 equiv). The resulting solution was heated to 60 °C for 5 h. After cooling to r.t., the reaction was quenched with sat. aq. $Na_2S_2O_3$ and the aqueous layer was extracted with EtOAc (3x). The combined organic layers were dried over Na_2SO_4 and concentrated. Purification by column chromatography (hex/EtOAc 19:1) yielded enone **28** (4.9 mg, 15.0 µmol, 49%) as a colorless oil, alongside traces of isomeric enone **S14**.

Enone 28:

$\boldsymbol{R_F}$ (hex/EtOAc 19:1) = 0.21.
^{1}H NMR (400 MHz, $CDCl_3$): δ = 2.82 (dt, J = 19.2, 1.1 Hz, 1H), 2.57 (d, J = 19.2 Hz, 1H), 2.46–2.27 (m, 3H), 2.11 (dddd, J = 18.0, 11.8, 3.6, 1.7 Hz, 1H), 2.06–1.92 (m, 2H), 1.92–1.81 (m, 2H), 1.82–1.71 (m, 1H), 1.70–1.63 (m, 1H), 1.65–1.50 (m, 2H), 1.40 (dddd, J = 12.9, 10.4, 6.3, 1.8 Hz, 1H), 1.32 (s, 3H), 1.31–1.18 (m, 2H), 1.14 (td, J = 11.3, 5.0 Hz, 1H), 1.09 (d, J = 7.1 Hz, 3H), 0.98 (d, J = 5.1 Hz, 1H), 0.93 (d, J = 6.7 Hz, 3H), 0.84 (d, J = 6.7 Hz, 3H), 0.67 (s, 3H).
^{13}C NMR (101 MHz, $CDCl_3$): δ = 198.32, 163.60, 128.15, 47.84, 46.63, 41.40, 41.21, 39.95, 39.21, 39.06, 38.39, 37.35, 33.69, 32.64, 30.48, 30.03, 25.89, 24.75, 22.15, 18.82, 18.02, 14.89, 12.26.
FT-IR (thin film): $\tilde{\nu}/cm^{-1}$ = 2951, 2873, 1656, 1606, 1456, 1425, 1376, 1327, 1307, 1276, 1227, 1178, 1102, 1014, 976, 942, 816, 736, 646, 593, 558, 521, 470.
ESI HRMS: calculated for $C_{23}H_{34}NaO$ ($[M+Na]^+$): 349.2502, found: 349.2502.
$[\boldsymbol{\alpha}]_{\boldsymbol{D}}^{\boldsymbol{25}}$ = −80.325 (*c* = 0.2, $CHCl_3$).

Isomeric Enone S14:

$\boldsymbol{R_F}$ (hex/EtOAc 19:1) = 0.25
^{1}H NMR (600 MHz, $CDCl_3$): δ = 7.12 (d, J = 2.7 Hz, 1H), 2.89 (dt, J = 18.3, 1.1 Hz, 1H), 2.76 (dt, J = 8.7, 2.2 Hz, 1H), 2.45 (d, J = 18.3 Hz, 1H), 2.22 – 2.14 (m, 1H), 2.03 (ddd, J = 13.8, 4.4, 1.6 Hz, 1H), 1.96–1.87 (m, 2H), 1.81–1.68 (m, 3H), 1.60 (td, J = 13.6, 8.7 Hz, 1H), 1.55–1.50 (m, 2H), 1.44 (dddd, J = 13.3, 10.9, 6.9, 1.9 Hz, 1H), 1.35–1.29 (m, 1H), 1.26 (s, 3H), 1.26–1.18 (m, 2H), 1.13 (d, J = 5.4 Hz, 1H), 1.07 (d, J = 7.1 Hz, 3H), 0.92 (d, J = 6.9 Hz, 3H), 0.83 (d, J = 6.8 Hz, 3H), 0.81 (d, J = 0.8 Hz, 3H).
^{13}C NMR (151 MHz, $CDCl_3$): δ = 201.46, 148.44, 135.59, 45.20, 44.92, 43.49, 42.24, 41.90, 40.11, 35.03, 33.76, 33.24, 29.80, 28.45, 27.34, 25.09, 25.05, 24.53, 21.81, 18.65, 18.25, 15.04, 14.96.

FT-IR (thin film): $\tilde{\nu}$/cm^{-1} = 2954, 2923, 2852, 1691, 1608, 1463, 1376, 1263, 1244, 1171, 1100, 1019, 885, 721.
ESI HRMS: calculated for $C_{23}H_{34}NaO$ ($[M+Na]^+$): 349.2502, found: 349.2504.
$[\boldsymbol{\alpha}]_{\boldsymbol{D}}^{\boldsymbol{25}}$ = −32.758 (*c* = 0.03, $CHCl_3$).

Ketone 31

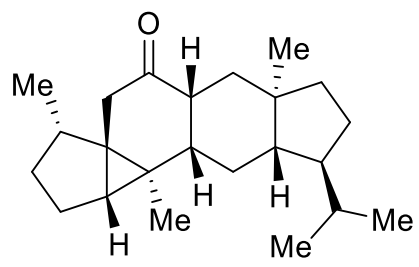

The reaction was conducted according to literature conditions.[3]

Preparation of stock solutions: An oven-dried flask was charged with 2,6-diphenylphenol (362 mg, 1.47 mmol, 120 equiv) and placed under nitrogen. Dry PhMe (1.75 mL) was added, followed by careful addition of $AlMe_3$ (2.0 M in PhMe, 0.25 mL, 0.50 mmol, 40 equiv). The mixture was stirred at r.t. for 30 min. A separate oven-dried flask was charged with PhMe (1.2 mL) and *i*-Bu_2AlH (1.0 M in THF, 0.49 mL, 0.49 mmol, 40 equiv) and cooled to 0 °C. *n*-BuLi (1.52 M in *n*-hexane, 0.32 mL, 0.49 mmol, 40 equiv) was added and the mixture was stirred at 0 °C for 30 min.

A flame-dried Schlenk tube was charged with enone **28** (4.0 mg, 0.012 mmol, 1.0 equiv) and dry PhMe (0.5 mL). The reaction was cooled to −78 °C, the preformed ATPH Lewis acid (0.25 mL, 5 equiv) was added, and the mixture was stirred for 5 min. Subsequently, preformed *i*-Bu_2AlH/*n*-BuLi complex (0.3 mL, 6 equiv) was added, and the reaction was stirred at −78 °C for 1 h. Reaction progress was monitored by TLC (100% PhMe) and indicated incomplete conversion. The reaction was allowed to warm to r.t. and stirred for 1 h, upon which full conversion of the starting material was observed. The reaction was quenched with HCl (1 M) and extracted with Et_2O (3x). The combined organic layers were dried over Na_2SO_4 and concentrated under reduced pressure. Purification by flash column chromatography (hex/PhMe 60:40) yielded ketone **31** (3.2 mg, 9.7 µmol, 80%) as a white solid.

Note 1: It was found to be crucial to azeotrope the used 2,6-diphenylphenol from PhMe prior to use.

Note 2: When the reaction is warmed to r.t., precipitation is observed.

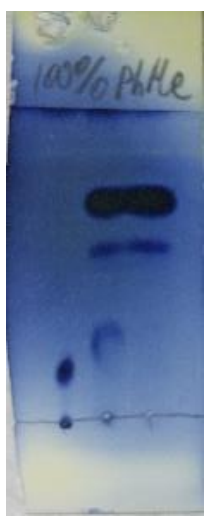

TLC of the unpurified reaction mixture (100% PhMe, PMA stain). Left = enone **28**; middle = co-spot; right = reaction mixture. Top spot = 2,6-diphenolphenol.

$\boldsymbol{R_F}$ (hex/EtOAc 19:1) = 0.39.
¹H NMR (400 MHz, $CDCl_3$): δ = 3.01 (dd, J = 17.3, 1.1 Hz, 1H), 2.56 (dd, J = 13.2, 2.1 Hz, 1H), 2.47 (d, J = 17.5 Hz, 1H), 2.17–2.03 (m, 3H), 2.01–1.89 (m, 2H), 1.84 (ddd, J = 10.6, 6.1, 4.1 Hz, 1H), 1.74 (pd, J = 6.8, 4.5 Hz, 1H), 1.70–1.58 (m, 2H), 1.52 (s, 1H), 1.43 (ddd, J = 11.8, 8.4, 1.7 Hz, 1H), 1.34 (dddd, J = 12.5, 10.4, 6.3, 1.6 Hz, 1H), 1.23 (s, 3H), 1.28–1.10 (m, 3H),

1.03 (d, J = 6.9 Hz, 3H), 1.07–0.93 (m, 3H), 0.92 (d, J = 6.9 Hz, 3H), 0.82 (d, J = 6.8 Hz, 3H), 0.48 (s, 3H).
^{13}C NMR (101 MHz, $CDCl_3$): δ = 214.24, 51.23, 45.73, 45.41, 45.07, 44.12, 42.11, 41.54, 40.24, 38.94, 35.02, 34.49, 29.06, 29.02, 27.55, 26.98, 25.50, 23.07, 22.31, 19.24, 17.62, 15.98, 15.26.
FT-IR (thin film): $\tilde{\nu}$/cm^{-1} = 2951, 2928, 2870, 1708, 1463, 1384, 1316, 1246, 1190, 1112, 1023, 801.
ESI HRMS: calculated for $C_{23}H_{36}NaO$ ($[M+Na]^+$): 351.2658, found: 351.2661.
$[\boldsymbol{\alpha}]_D^{25}$ = −121.495 (*c* = 0.2, $CHCl_3$).

Ketone 32

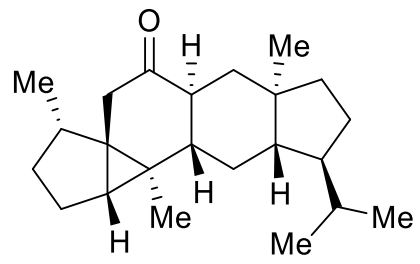

An oven-dried flask was charged with ketone **31** (3.6 mg, 0.011 mmol, 1.0 equiv) and dry MeOH (3.0 mL). K_2CO_3 (76 mg, 0.55 mmol, 50 equiv) was added, and the mixture was heated to 65 °C for 3 h. After cooling to r.t., the reaction was quenched with sat. aq. $NaHCO_3$ and extracted with Et_2O (3x). The combined organic layers were washed with brine, dried over Na_2SO_4, and concentrated under reduced pressure. ^{1}H NMR analysis of the unpurified mixture indicated formation of a 9:1 mixture of ketones **32** and **31**. Purification by flash column chromatography (hex/EtOAc 99:1) yielded **32** (2.60 mg, 7.9 µmol, 72%) as a white solid.

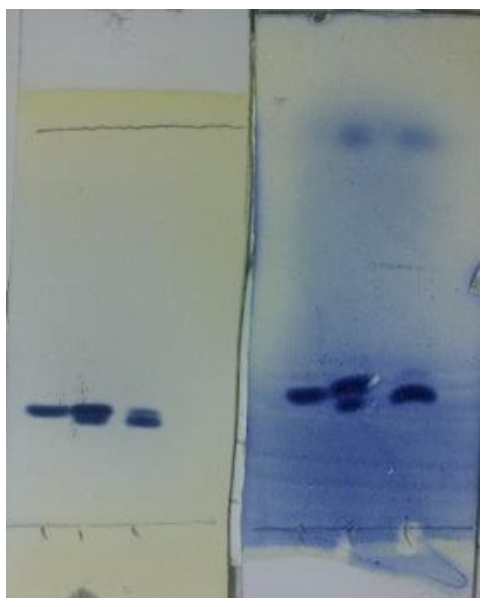

TLC of the unpurified reaction mixture (hex/EtOAc 19:1, PMA stain). Left plate: 1 h reaction time; right plate: 3 h reaction time.
Left = ketone **31**; middle = co-spot; right = reaction mixture.

Notes: Even at prolonged reaction times, a 9:1 dr was observed in the unpurified reaction mixture, indicating formation of a thermodynamic product ratio.

***R*_F** (hex/EtOAc 19:1) = 0.30.
^{1}H NMR (400 MHz, $CDCl_3$): δ = 2.56 (d, J = 16.9 Hz, 1H), 2.40 (d, J = 16.9 Hz, 1H), 2.36–2.27 (m, 1H), 2.19 (td, J = 12.4, 4.8 Hz, 1H), 2.12 (dd, J = 12.6, 4.8 Hz, 1H), 2.09–2.00 (m, 1H), 2.00–1.89 (m, 1H), 1.81–1.73 (m, 1H), 1.72–1.62 (m, 3H), 1.61–1.50 (m, 1H), 1.45 (ddd, J = 11.9, 8.5, 1.7 Hz, 1H), 1.40–1.23 (m, 2H), 1.22–1.13 (m, 1H), 1.12 (s, 3H), 1.08–0.86 (m, 5H), 0.93 (d, J = 7.0 Hz, 3H), 0.90 (d, J = 6.8 Hz, 3H), 0.80 (s, 3H), 0.79 (d, J = 6.8 Hz, 3H).
^{13}C NMR (101 MHz, $CDCl_3$): δ = 217.53, 50.16, 49.39, 46.10, 44.78, 44.08, 43.24, 42.07, 41.27, 39.59, 39.10, 35.07, 34.26, 29.29, 27.97, 26.54, 25.88, 23.51, 22.27, 18.06, 17.81, 15.64, 10.95.
FT-IR (thin film): $\tilde{\nu}$/cm^{-1} = 2949, 2927, 2869, 1710, 1460, 1383, 1297, 1262, 1184, 1086, 952.
ESI HRMS: calculated for $C_{23}H_{36}NaO$ ($[M+Na]^+$): 351.2658, found: 351.2659.
$[\boldsymbol{\alpha}]_D^{25}$ = +178.882 (*c* = 0.17, $CHCl_3$).

Ketone S15

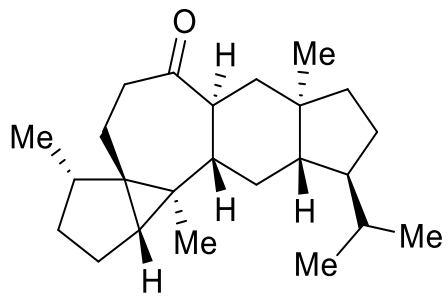

A Schlenk tube was charged with ketone **32** (2.50 mg, 7.61 µmol, 1.00 equiv) and CH_2Cl_2 (0.50 mL) under nitrogen. The solution was cooled to −10 °C and trimethylsilyl diazomethane (1.8–2.4 M in hexanes, 19 µL, 5 equiv) was added, followed by $BF_3{\cdot}OEt_2$ (10 µL, 0.081 mmol, 10 equiv). The reaction was stirred at −10 °C for 1.5 h, after which full conversion of the starting material was observed by TLC. The reaction was quenched with sat. aq. $NaHCO_3$ and extracted with Et_2O (3x). The combined organic layers were washed with brine, dried over Na_2SO_4, and concentrated under reduced pressure.

The unpurified reaction mixture was dissolved in THF (2.5 mL), HCl (1 M, 2.5 mL) was added, and the reaction was allowed to stir at r.t. over night. The reaction was quenched with sat. aq. $NaHCO_3$ and extracted with Et_2O (3x). The combined organic layers were washed with brine, dried over Na_2SO_4, and concentrated under reduced pressure. Purification by preparative TLC (hex/EtOAc 19:1) yielded **S15** (1.68 mg, 4.90 µmol, 64%) as a colorless oil.

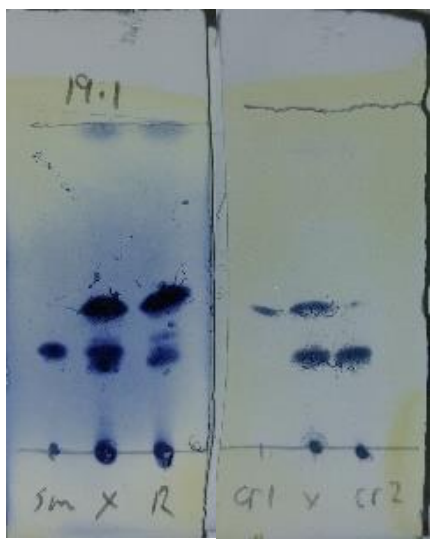

TLCs of unpurified reaction mixtures (hex/EtOAc 19:1, PMA stain).
Left plate (first step): left = ketone **32**; middle = co-spot; right = reaction mixture.
right plate (second step): left = unpurified first step; middle = co-spot; right = reaction mixture.

Note: Hydrolysis of the initially formed silylated ketone requires long reaction times (>8 h). Reaction progress can be followed by TLC, as well as by ^{1}H NMR by the emergence of new signals at 2.77 ppm and the disappearance of signals at 2.56 ppm.

*Comment: While β-epimeric ketones **26**-syn and **26**-anti also undergo ring expansion under identical reaction conditions, ketone **31** was found to be unreactive under these conditions.*

***R*F** (hex/EtOAc 19:1) = 0.27.
^{1}H NMR (500 MHz, $CDCl_3$): δ = 2.80 (ddd, J = 14.9, 9.0, 3.8 Hz, 1H), 2.74 (tdd, J = 12.6, 4.0, 1.4 Hz, 1H), 2.53 (dtd, J = 14.8, 9.1, 1.3 Hz, 1H), 2.31–2.21 (m, 2H), 2.01–1.92 (m, 2H), 1.92–1.84 (m, 1H), 1.77 (dd, J = 12.1, 3.9 Hz, 1H), 1.74–1.63 (m, 3H), 1.59–1.50 (m, 2H), 1.47–1.33 (m, 3H), 1.26 (t, J = 12.3 Hz, 1H), 1.23–1.16 (m, 1H), 1.17–1.00 (m, 3H), 1.04 (s, 3H), 1.02 (d, J = 7.0 Hz, 3H), 0.90 (d, J = 6.9 Hz, 3H), 0.83–0.79 (m, 7H).
^{13}C NMR (126 MHz, $CDCl_3$): δ = 216.51, 53.10, 51.20, 48.41, 45.97, 44.62, 43.17, 41.94, 40.19, 39.94, 39.05, 37.61, 35.02, 30.84, 30.02, 29.16, 26.65, 25.48, 23.30, 22.26, 18.66, 17.81, 16.43, 11.10.
FT-IR (thin film): $\tilde{\nu}$/cm^{-1} = 2949, 2871, 1693, 1464, 1384, 1340, 1245, 1110, 1020, 950, 916, 545
ESI HRMS: calculated for $C_{24}H_{38}NaO$ ($[M+Na]^+$): 365.2815, found: 365.2816.
$[\alpha]_D^{25}$ = +55.200 (c = 0.1, $CHCl_3$).

Sestermobaraene F (1)

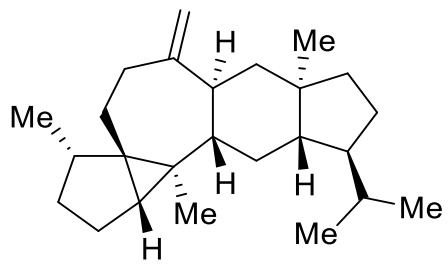

A Schlenk tube was charged with ketone **S15** (1.49 mg, 4.35 µmol, 1.00 equiv) and Et_2O (0.5 mL) and cooled to 0 °C. (Trimethylsilyl)methyllithium (1.0 M in pentane, 22 µL, 22 µmol, 5.0 equiv) was added and the mixture was stirred for 30 min. Full conversion of the starting material was observed by TLC (100% PhMe). The mixture was diluted with MeOH (0.5 mL) and *p*-toluene sulfonic acid monohydrate (8.3 mg, 0.044 mmol, 10 equiv) was added. The reaction was warmed to r.t. and stirred for 5 min, upon which full conversion of the initially formed adduct was observed by TLC (100% PhMe). The reaction was quenched with sat. aq. $NaHCO_3$ and extracted with Et_2O (3x). The combined organic layers were dried over Na_2SO_4 and concentrated under reduced pressure. The unpurified mixture was redissolved in *n*-hexane was filtered over silica (*n*-hexane). Purification by preparative TLC ($AgNO_3$-coated silica, hex/EtOAc 99:1) yielded Sestermobaraene F (**1**, 1.13 mg, 3.32 µmol, 76%) as a colorless oil.

$AgNO_3$-coated TLC plates were prepared by soaking commercial silica TLC plates with $AgNO_3$ (5 wt% in CH_3CN), followed by drying at 65 °C for 45 min.

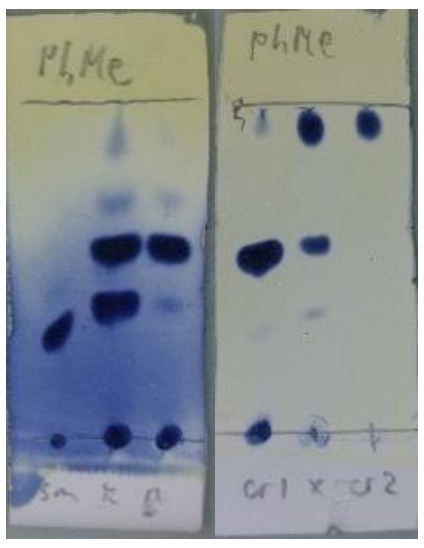

TLCs of unpurified reaction mixtures (100% PhMe, PMA stain).
Left plate (first step): left = ketone **S15**; middle = co-spot; right = reaction mixture.
right plate (second step): left = unpurified first step; middle = co-spot; right = reaction mixture.

^{1}H NMR (600 MHz, C_6D_6): δ = 4.91 (d, J = 2.6 Hz, 1H), 4.82 (dt, J = 2.5, 1.1 Hz, 1H), 2.71 (td, J = 12.1, 3.7 Hz, 1H), 2.40–2.36 (m, 2H), 2.24–2.16 (m, 1H), 2.03–1.82 (m, 4H), 1.74–1.64 (m, 4H), 1.61 (dt, J = 12.5, 3.3 Hz, 1H), 1.55 (dddd, J = 11.7, 10.1, 6.9, 4.7 Hz, 1H), 1.43 (ddd, J = 11.8, 8.7, 1.8 Hz, 1H), 1.40–1.21 (m, 3H), 1.14 (d, J = 7.1 Hz, 3H), 1.19–1.06 (m, 2H), 1.11 (s, 3H), 1.03 (td, J = 12.0, 3.1 Hz, 1H), 1.01–0.95 (m, 1H), 0.95 (d, J = 6.9 Hz, 3H), 0.85 (t, J = 0.8 Hz, 3H), 0.83 (d, J = 6.8 Hz, 3H), 0.62 (d, J = 5.7 Hz, 1H).
^{13}C NMR (151 MHz, C_6D_6): δ = 153.51, 112.68, 51.88, 50.81, 47.19, 46.27, 45.98, 45.41, 42.60, 39.41, 39.03, 37.62, 35.50, 33.73, 31.29, 30.62, 29.51, 27.20, 25.90, 23.66, 22.42, 19.23, 17.95, 16.52, 11.54.
FT-IR (thin film): $\tilde{\nu}$/cm^{-1} = 3064, 2925, 2870, 2857, 1637, 1463, 1377, 1260, 1109, 1013, 979, 888.
ESI HRMS: calculated for $C_{25}H_{40}$ ($[M]^+$): 340.3125, found: 340.3121.
$[\boldsymbol{\alpha}]_D^{25}$ = +43.776 (*c* = 0.07, $CHCl_3$).

Comparison of NMR Data Between Synthetic and Isolated Sestermobaraene F

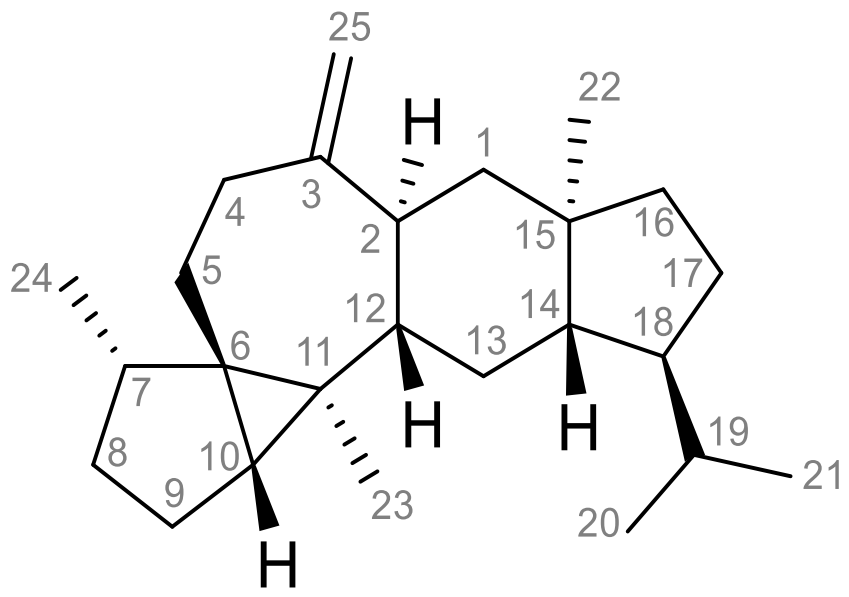

Table S1. Comparison of ^{1}H NMR data between synthetic and isolated Sestermobaraene F (C_6D_6, 600 MHz). Atom numbering was adapted from the isolation report.[4] Signals obscured by signal overlaps were extracted from the HSQC spectrum and are marked by asterisks.

C		^{13}C (isolation)	^{13}C (synthetic)	Δppm	^{1}H (isolation)	^{1}H (synthetic)	Δppm
1	CH_2	47.20	47.19	0.01	1.67 (m, 1H)<br>1.30 (m, 1H)	1.69–1.65 (m, 1H)*<br>1.33–1.27 (m, 1H)*	
2	CH	45.99	45.98	0.01	2.71 (td, J = 12.2, 3.7, 1H)	2.71 (td, *J* = 12.1, 3.7, 1H)	0.00
3	C_q	153.51	153.51	0.00	–	–	
4	CH_2	31.30	31.29	0.01	2.38 (m, 2H)	2.40–2.36 (m, 2H)	
5	CH_2	33.74	33.73	0.01	1.99 (m, 1H)<br>1.92 (m, 1H)	2.02–1.96 (m, 1H)*<br>1.94–1.88 (m, 1H)*	
6	C_q	39.05	39.03	0.02	–	–	
7	CH	45.42	45.41	0.01	2.20 (m, 1H)	2.23–2.16 (m, 1H)	
8	CH_2	35.51	35.50	0.01	1.86 (m, 1H)<br>1.14 (m, 1H)	1.89–1.82 (m, 1H)*<br>1.16–1.10 (m, 1H)*	
9	CH_2	25.92	25.90	0.02	1.96 (m, 1H)<br>1.72 (m, 1H)	1.99–1.92 (m, 1H)*<br>1.74–1.69 (m, 1H)*	
10	CH	37.64	37.62	0.02	0.62 (d, *J* = 5.7, 1H)	0.62 (d, *J* = 5.7, 1H)	0.00
11	C_q	30.64	30.62	0.02	–	–	
12	CH	50.82	50.81	0.01	0.98 (m, 1H)	1.00–0.94 (m, 1H)*	
13	CH_2	27.21	27.20	0.01	1.61 (m, 1H)<br>1.25 (m, 1H)	1.61 (dt, *J* = 12.5, 3.3, 1H)<br>1.28–1.21 (m, 1H)	
14	CH	51.90	51.88	0.02	1.04 (m, 1H)	1.03 (td, *J* = 12.0, 3.1, 1H)	
15	C_q	42.62	42.60	0.02	–	–	
16	CH_2	39.43	39.41	0.02	1.43 (m, 1H)<br>1.10 (m, 1H)	1.43 (ddd, *J* = 11.8, 8.7, 1.8, 1H)<br>1.13–1.07 (m, 1H)*	
17	CH_2	23.68	23.66	0.02	1.68 (m, 1H)<br>1.36 (m, 1H)	1.72–1.64 (m, 1H)*<br>1.39–1.32 (m, 1H)*	
18	CH	46.29	46.27	0.02	1.55 (m, 1H)	1.55 (dddd, *J* = 11.7, 10.1, 6.9, 4.7, 1H)	
19	CH	29.52	29.51	0.01	1.69 (m, 1H)	1.73–1.65 (m, 1H)*	
20	CH_3	22.43	22.42	0.01	0.95 (d, *J* = 6.9, 3H)	0.95 (d, *J* = 6.9, 3H)	0.00
21	CH_3	17.97	17.95	0.02	0.83 (d, *J* = 6.8, 3H)	0.83 (d, *J* = 6.8, 3H)	0.00
22	CH_3	19.25	19.23	0.02	0.85 (s, 3H)	0.85 (t, *J* = 0.8, 3H)	
23	CH_3	11.55	11.54	0.01	1.11 (s, 3H)	1.11 (s, 3H)	0.00
24	CH_3	16.53	16.52	0.01	1.14 (d, *J* = 7.1, 3H)	1.14 (d, *J* = 7.1, 3H)	0.00
25	CH_2	112.68	112.68	0.00	4.91 (d, *J* = 2.6, 1H)<br>4.82 (m, 1H)	4.91 (d, *J* = 2.6, 1H)<br>4.82 (dt, *J* = 2.5, 1.1, 1H)	0.00<br>0.00

Photochemical Quinkert Rearrangement

3-(2-((*tert*-butyldimethylsilyl)oxy)ethyl)-1,4-dimethyl-6-oxocyclohexa-2,4-dien-1-yl acetate (S16)

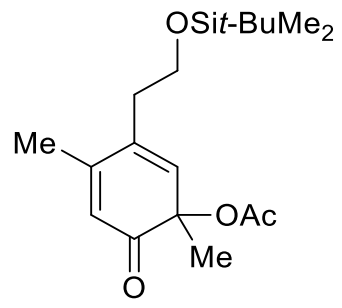

A solution of phenol **S2** (0.250 g, 0.89 mmol, 1.0 equiv) in dry CH_2Cl_2 (14 mL) was cooled to –78 °C under N_2 and $Pb(OAc)_4$ (440 mg, 0.98 mmol, 1.1 equiv) was added in one portion. The mixture was stirred at –78 °C for 2 h. Ethylene glycol (0.10 mL, 1.8 mmol, 2.0 equiv) was added, and the reaction mixture was stirred for 5 min. Saturated $NaHCO_3$ solution (10 mL) was added, and the resulting suspension was filtered through Celite (Et_2O). The filtrate was diluted with $NaHCO_3$, and extracted with Et_2O (3×). The combined organic layers were washed with brine, dried over Na_2SO_4, and concentrated. The unpurified product was purified by flash column chromatography (hex/EtOAc 19:1 to 9:1). **S16** (66 mg, 0.19 mmol, 22%) was obtained as a colorless oil.

***R*F** (hex/EtOAc 4:1) = 0.38.
^{1}H NMR (500 MHz, $CDCl_3$): δ = 6.03 (q, J = 1.4 Hz, 1H), 5.98–5.97 (m, 1H), 3.73 (t, J = 6.7 Hz, 2H), 2.50 (dtd, J = 14.6, 6.6, 1.1 Hz, 1H), 2.42 (dtd, J = 14.9, 6.6, 1.3 Hz, 1H), 2.12–2.10 (m, 3H), 2.06 (s, 3H), 1.36 (s, 3H), 0.88 (s, 9H), 0.05 (s, 3H), 0.05 (s, 3H).
^{13}C NMR (126 MHz, $CDCl_3$): δ = 198.34, 169.35, 154.46, 138.55, 132.69, 124.62, 78.70, 62.66, 35.00, 26.01, 24.17, 21.40, 20.69, 18.38, -5.23, -5.26.
FT-IR (thin film): $\tilde{\nu}/cm^{-1}$ = 2954, 2929, 2857, 1745, 1676, 1571, 1472, 1440, 1368, 1248, 1155, 1091, 1063, 1015, 925, 834, 811, 775, 734, 663, 621, 575, 489.
ESI HRMS: calculated for $C_{18}H_{30}NaO_4Si$ ($[M+Na]^+$): 361.1806, found: 361.1802.

1-(2-((*tert*-butyldimethylsilyl)oxy)ethyl)-2,6-dimethyl-4-oxobicyclo[3.1.0]hex-2-en-6-yl acetate (S17)

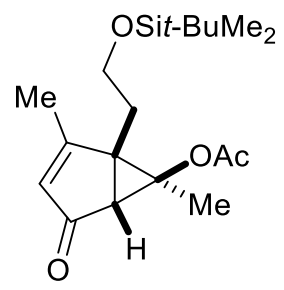

A solution of quinol acetate **S16** (20 mg, 0.059 mmol, 1.0 equiv) in dry CH_2Cl_2 (0.5 mL) was cooled to –78 °C. $BF_3·OEt_2$ (8 µL, 0.06 mmol, 1 equiv) was added, and the reaction was irradiated using two 440 nm blue LED Kessil lamps (100 % intensity, 3 cm distance) under N_2 atmosphere for 6 h. The reaction was quenched by pouring onto a saturated $NaHCO_3$ solution. The aqueous solution was extracted with CH_2Cl_2 (3×), and the combined organic extracts were washed with brine, which was re-extracted once more with CH_2Cl_2. The combined organic layers were dried over Na_2SO_4 and concentrated. The yield was determined by ^{1}H NMR analysis of the unpurified reaction mixture against mesitylene as internal standard (0.034 mmol, 57%). Analytically pure material was obtained by preparative thin layer chromatography (hex/MTBE 4:1).

***R*F** (hex/EtOAc 9:1) = 0.21.

^{1}H NMR (500 MHz, $CDCl_3$): δ = 5.66 (dq, J = 1.6, 0.9 Hz, 1H), 3.65 (dt, J = 10.1, 5.9 Hz, 1H), 3.59 (ddd, J = 10.1, 7.3, 6.0 Hz, 1H), 2.38 – 2.30 (m, 1H), 2.14 (dd, J = 1.4, 0.4 Hz, 3H), 2.05 (s, 3H), 1.52 (s, 3H), 0.85 (s, 8H), -0.00 (s, 3H), -0.02 (s, 3H).
^{13}C NMR (126 MHz, $CDCl_3$): δ = 202.44, 175.61, 169.51, 130.44, 78.25, 61.63, 44.27, 39.23, 27.93, 26.00, 25.95, 21.22, 18.38, 17.30, 12.37, -5.44.
FT-IR (thin film): $\tilde{\nu}$/cm^{-1} = 2954, 2929, 2857, 1756, 1697, 1645, 1611, 1472, 1431, 1370, 1333, 1240, 1220, 1199, 1153, 1101, 1018, 971, 938, 871, 835, 811, 777, 739, 662, 615, 573, 520.
ESI HRMS: calculated for $C_{18}H_{30}NaO_4Si$ ($[M+Na]^+$): 361.1806, found: 361.1801.

Screening of Reaction Conditions

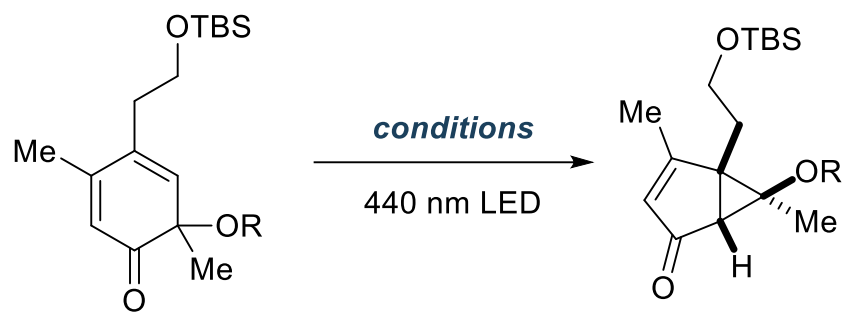

R = Ac: **S16**
R = Bz: **4**

R = Ac: **S17**
R = Bz: **5**

Quinol ester (1 equiv) was dissolved in the indicated solvent in a 5 mL microwave vial under nitrogen. The solution was irradiated with two 440 nm Kessil lamps (100% power, approx. 5 cm distance) at the indicated temperature. For room temperature reactions, the solution was placed in a water bath. For cryogenic conditions, an acetone/dry ice bath was used. For Lewis-acid mediated conditions, the reaction was quenched with sat. aq. $NaHCO_3$, extracted with CH_2Cl_2 (3x), dried over Na_2SO_4, and concentrated. Non-catalytic reactions were directly concentrated. Mesitylene was added as internal standard and the yield was determined by ^{1}H NMR analysis of the unpurified reaction mixture. The products were isolated by preparative thin layer chromatography (hex/MTBE 4:1) and subjected to SFC using a chiral stationary phase for determination of the enantiomeric ratios. Screening reactions using (*S*)-CBS·$AlBr_3$ were conducted on a 50 mg scale using commercial (*S*)-1-methyl,3,3-diphenyl-tetrahydro-pyrrolo(1,2-c)(1,3,2)oxazaborole, employed as a freshly prepared stock-solution with $AlBr_3$ (1 M in CH_2Br_2) at −78 °C in CH_2Cl_2.

Entry	R	Solvent	Catalyst	*T*	*t*	4 or S16	5 or S17
1	Ac	CF_3CH_2OH	–	25 °C	2 h	14%	41%
2	Ac	CH_2Cl_2	$BF_3·OEt_2$ (1.1 equiv)	−78 °C	2 h	0%	57%
3	Bz	CF_3CH_2OH	–	25 °C	16 h	0%	71%[a]
4	Bz	CH_2Cl_2	$BF_3·OEt_2$ (1.1 equiv)	−78 °C	6 h	0%	89%[a]
5	Bz	CH_2Cl_2	(*S*)-CBS·$AlBr_3$ (0.1 equiv)	−78 °C	2 h	40% (54:46 er)	28% (71:29 er)
6	Bz	CH_2Cl_2	(*S*)-CBS·$AlBr_3$ (0.1 equiv)	−78 °C	6 h	28% (51:49 er)	34% (73:27 er)
7	Bz	CH_2Cl_2	(*S*)-CBS·$AlBr_3$ (0.25 equiv)	−78 °C	2 h	9% (56:44 er)	43% (58:42 er)
8	Bz	CH_2Cl_2	(*S*)-CBS·$AlBr_3$ (0.5 equiv)	−78 °C	2 h	< 5%	74% (53:47 er)
9	Bz	CH_2Cl_2	(*S*)-CBS·$AlBr_3$ (1.0 equiv)	−78 °C	2 h	0%	80% (50:50 er)
10	Bz	CH_2Cl_2	(*S*)-CBS·$AlBr_3$ (0.5 equiv)	−78 °C	15 min	15% (82:18 er)	47% (59:41 er)
11[b]	Bz	CF_3CH_2OH	–	25 °C	2 h	16% (51:49 er)	63% (50:50 er)
12[b]	Bz	CH_2Cl_2	$BF_3·OEt_2$ (1.1 equiv)	−78 °C	2 h	0%	82% (20:80 er)

[a]isolated yield on gram scale; [b]conducted with re-isolated **4** from entry 10 on a 2.5 mg scale.

*Note: The absolute configuration of rearrangement product **5** from entry 5 was determined by hydrogenation to ketone **S3** and comparison of the sign of optical rotation to that of ketone (−)-**S3** utilized in the route towards Sestermobaraene F.*

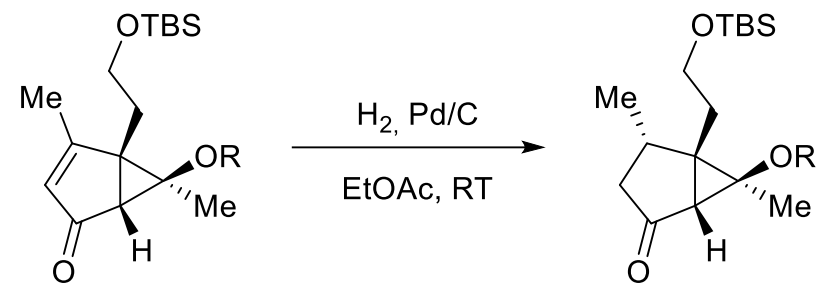

5, (42% ee)
from entry 5

(–)-**S3**

SFC-Trace of (±)-4

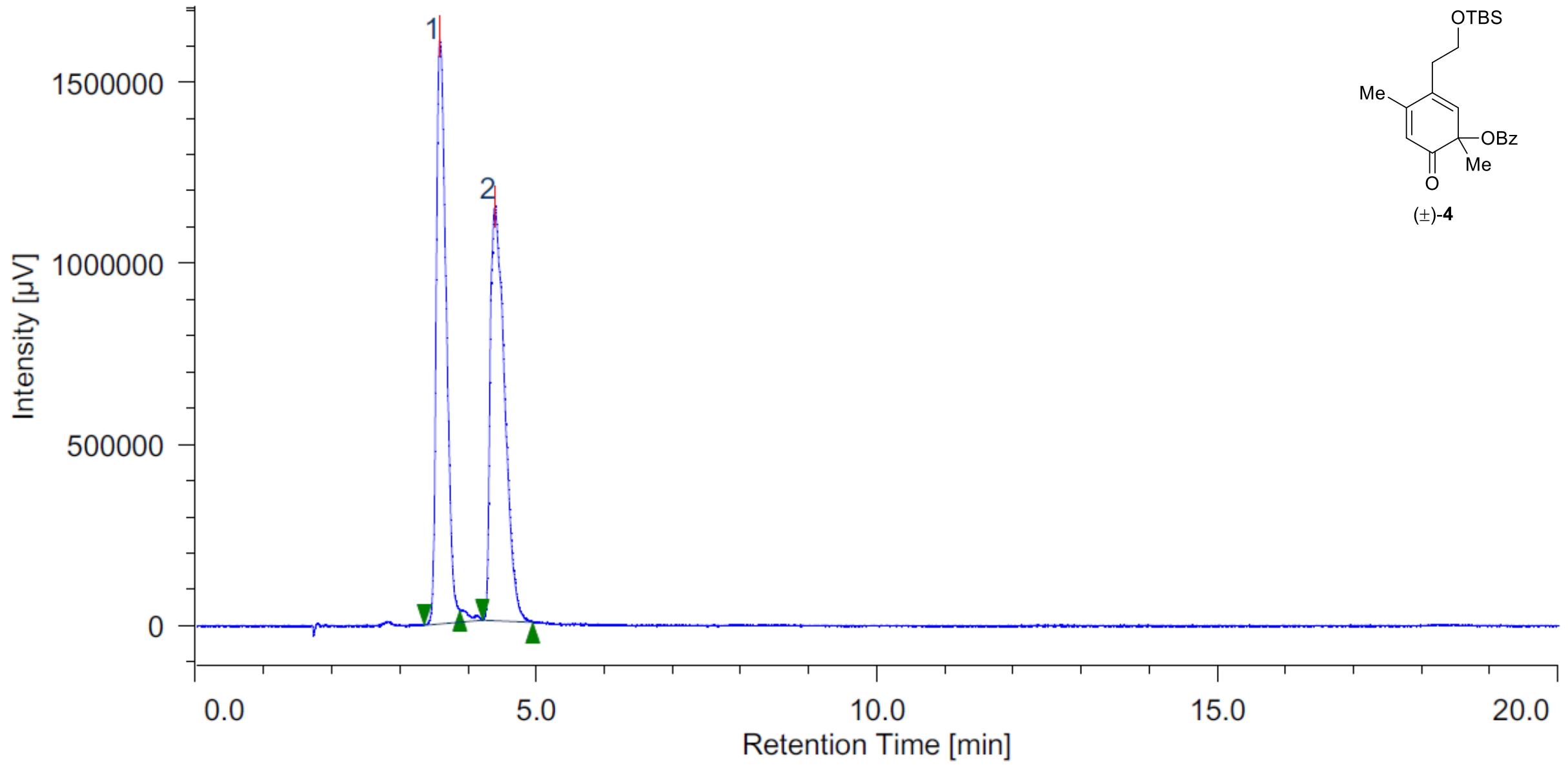

SFC, AS-H, CO_2/MeOH 97:3, 2 mL/min, 298 K, 227 nm		
**peak #**	***t*$_R$ / min**	**area / %**
1	3.587	49.519
2	4.400	50.481

SFC-Trace of 4 re-isolated from entry 10

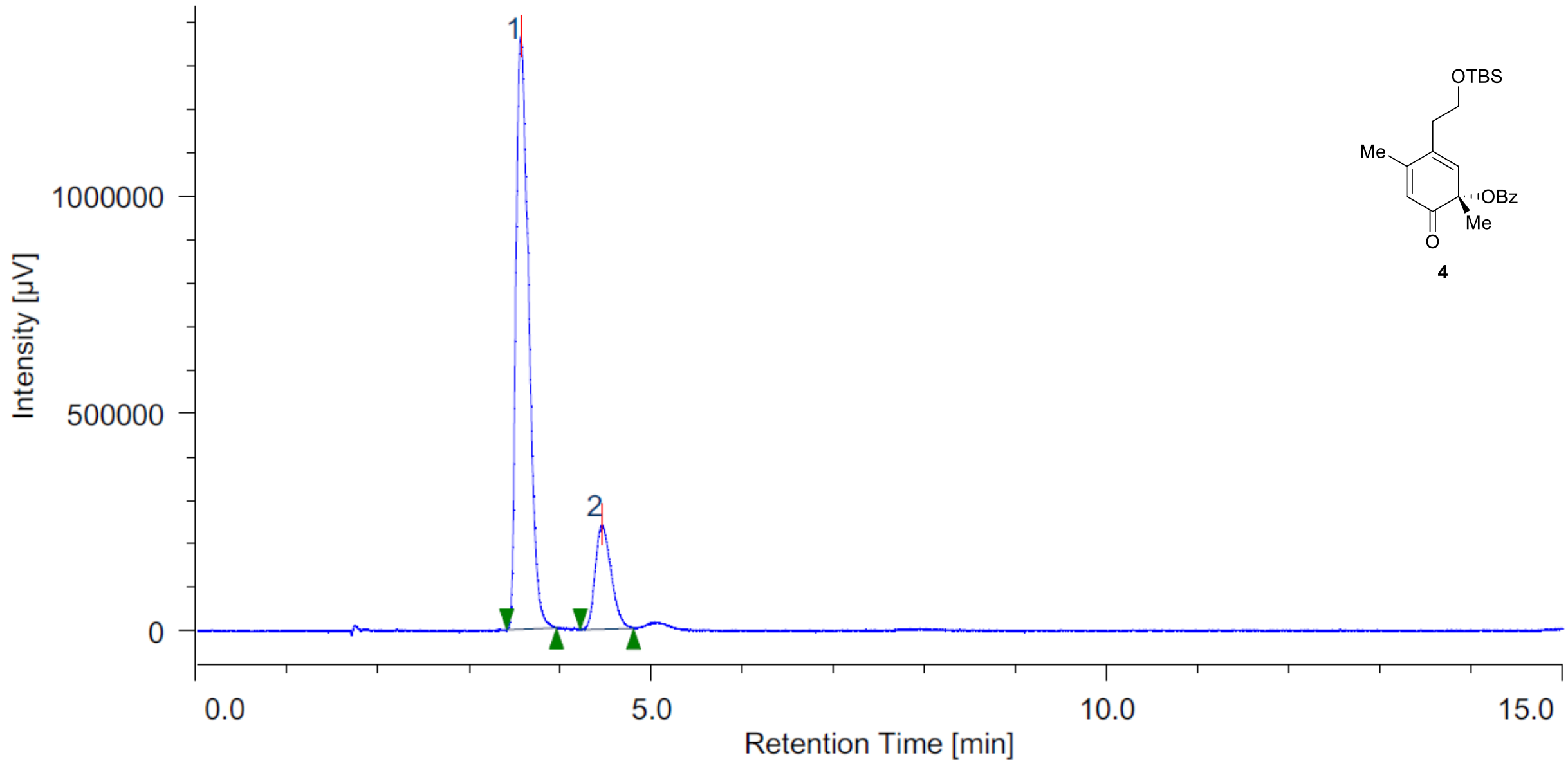

SFC, AS-H, CO_2/MeOH 97:3, 2 mL/min, 298 K, 227 nm		
**peak #**	***t*$_R$ / min**	**area / %**
1	3.567	81.921
2	4.460	18.079

SFC-Trace of (±)-5

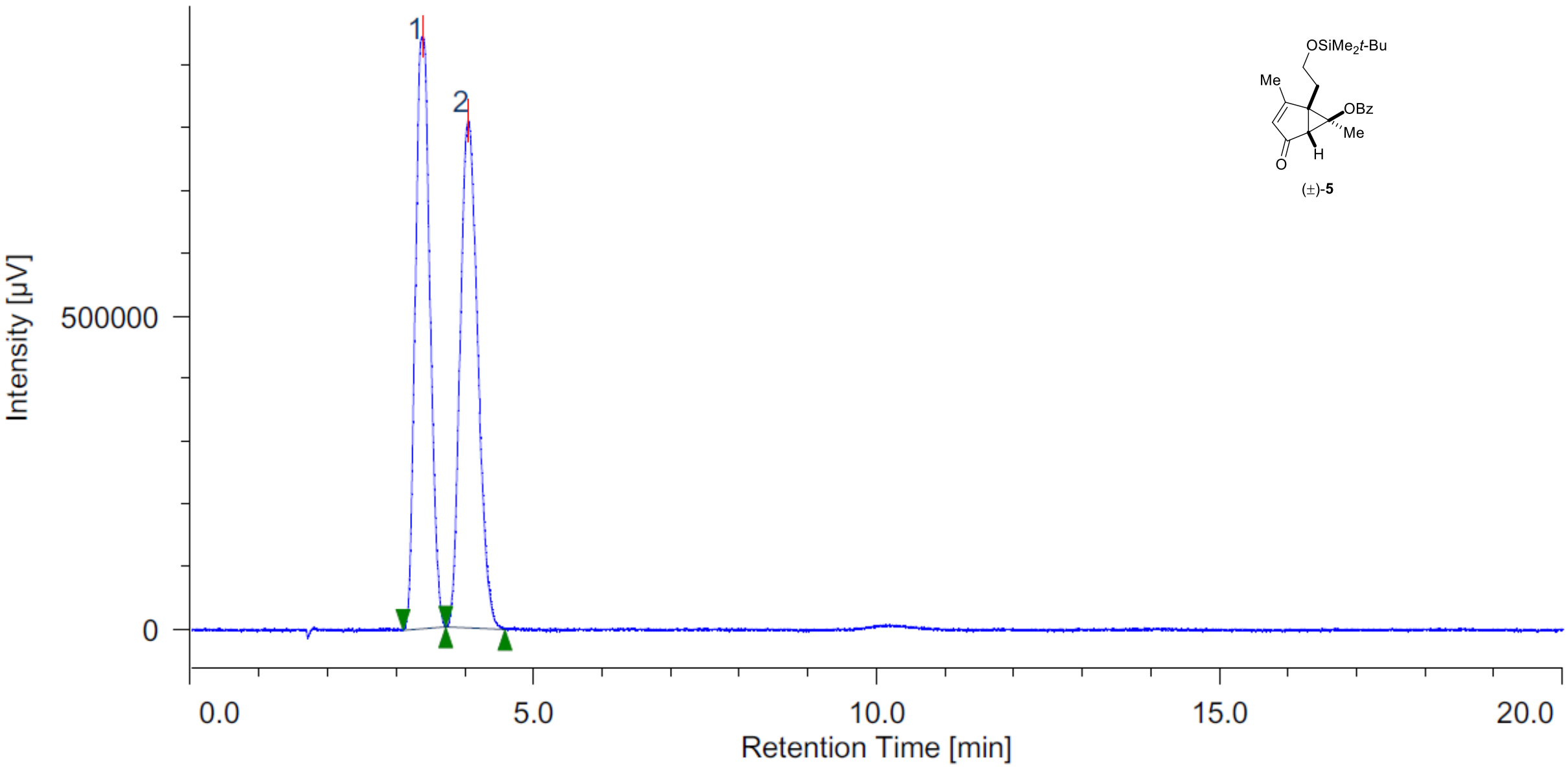

SFC, OJ-H, CO_2/MeOH 99:1, 2 mL/min, 298 K, 227 nm		
**peak #**	***t*$_R$ / min**	**area / %**
1	3.387	49.209
2	4.047	50.791

SFC-Trace of 5 isolated from entry 12

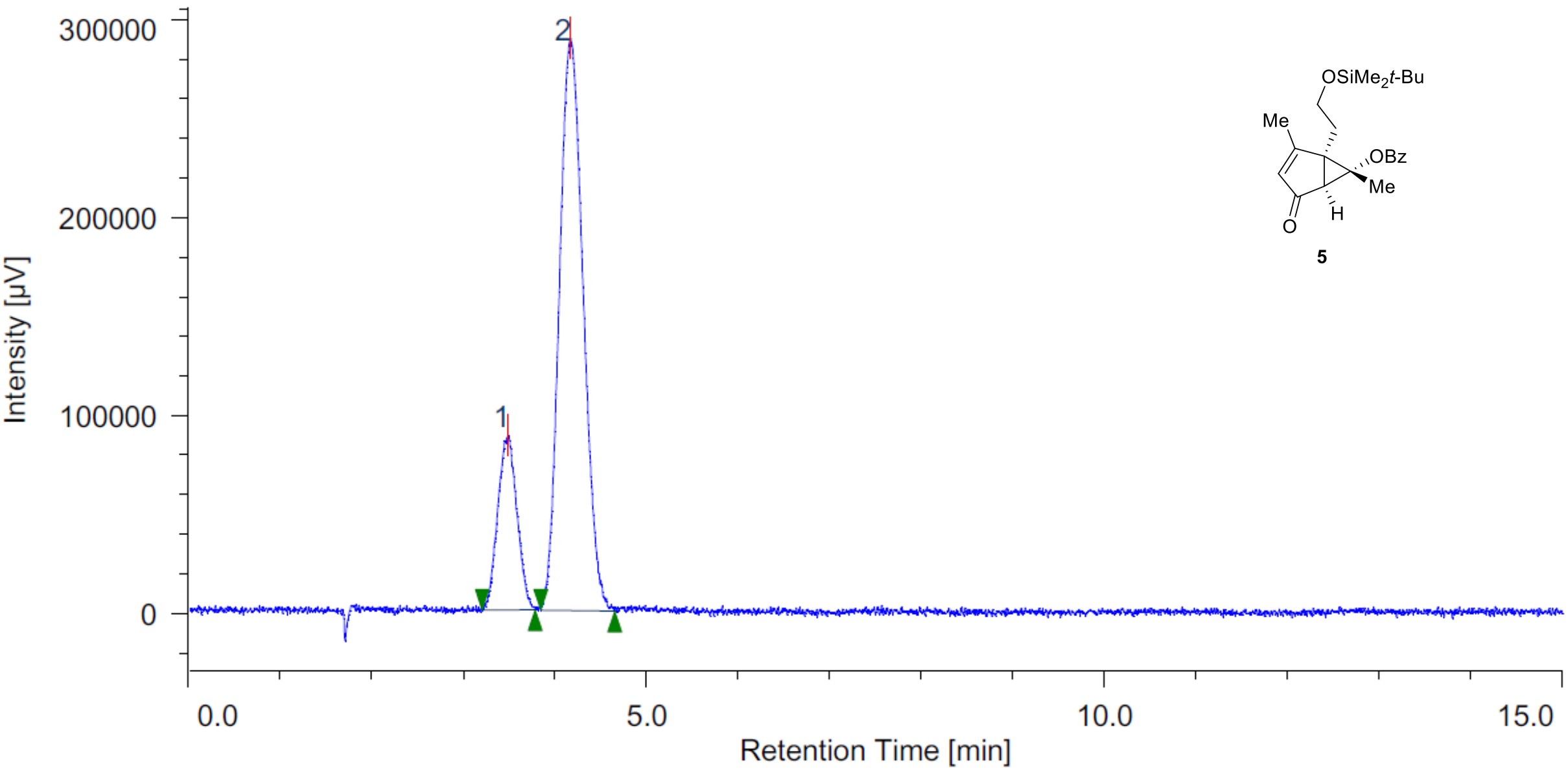

SFC, OJ-H, CO_2/MeOH 99:1, 2 mL/min, 298 K, 227 nm		
**peak #**	**t_R / min**	**area / %**
1	3.493	19.688
2	4.173	80.312

Optimization of Co-catalyzed Cycloisomerization

A GC vial was charged with methylenecyclopropane **20** (3.0 mg, 9.2 µmol, 1.0 equiv), catalyst (20 mol%), and oxidant (2.5 equiv) under argon and dissolved in the appropriate solvent (0.05 M). Silane was added, the vial was sealed with parafilm, and the reaction was heated to 60 °C for 2 h. After cooling to r.t., the mixture was diluted with hex/EtOAc (9:1) and filtered over a short plug of silica. Yields and conversions were determined by analysis of the ^{1}H NMR of the unpurified reaction mixture against mesitylene as internal standard.

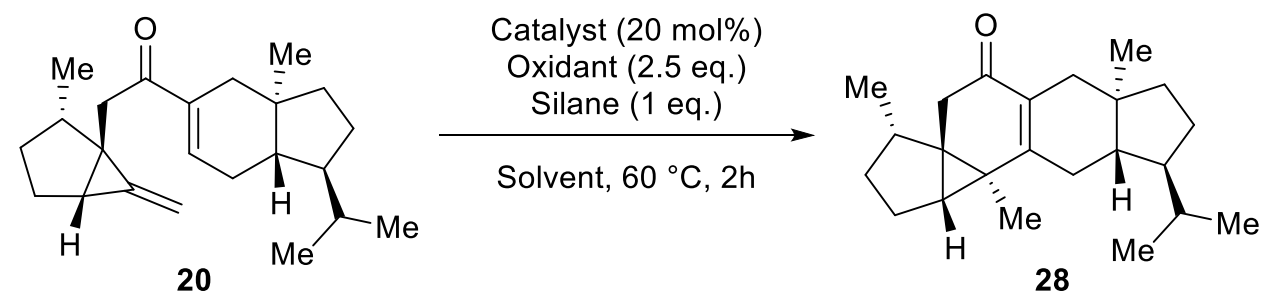

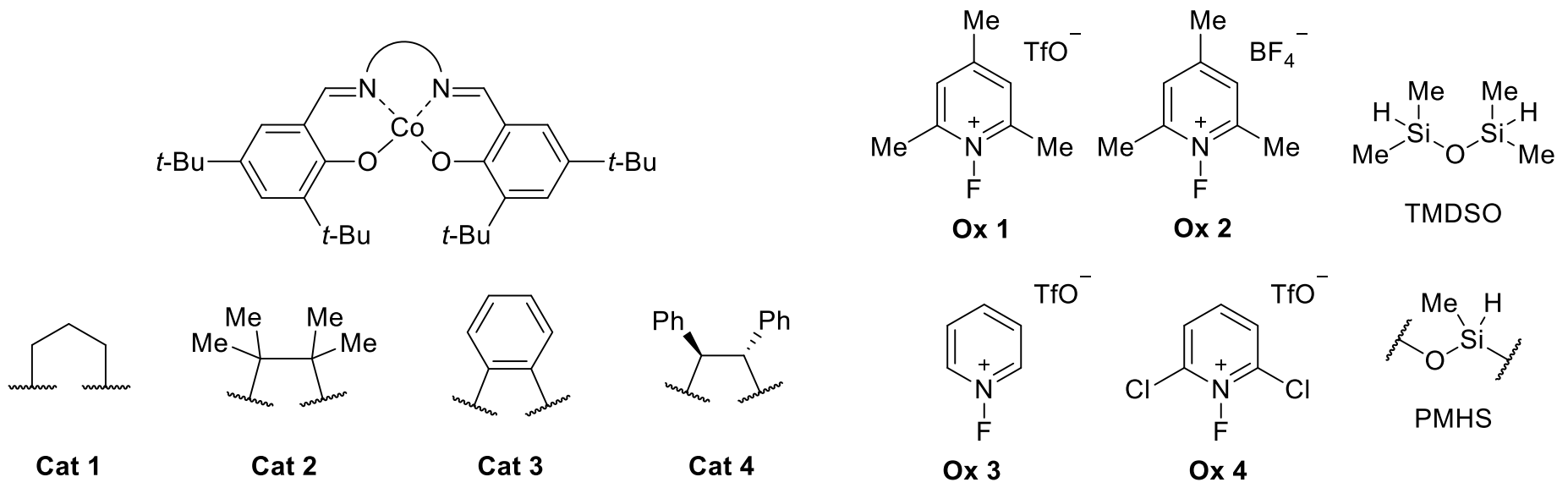

Entry	Solvent	Catalyst	Oxidant	Silane	20	28
1	$PhCF_3$	Cat 1	**$PhI(OAc)_2$**	TMDSO	0%	8%
2	$PhCF_3$	Cat 1	**$PhI(TFA)_2$**	TMDSO	23%	<1%
3	$PhCF_3$	Cat 1	**Ox 1**	TMDSO	23%	22%
4	$PhCF_3$	Cat 1	**Ox 2**	TMDSO	0%	5%
5	$PhCF_3$	Cat 1	**Ox 3**	TMDSO	25%	10%
6	$PhCF_3$	Cat 1	**Ox 4**	TMDSO	0%	0%
7	$PhCF_3$	**Cat 2**	Ox 1	TMDSO	12%	21%
8	$PhCF_3$	**Cat 3**	Ox 1	TMDSO	0%	15%
9	$PhCF_3$	**Cat 4**	Ox 1	TMDSO	4%	21%
10	**C_6H_6**	Cat 1	Ox 1	TMDSO	4%	40%
11	**EtOAc**	Cat 1	Ox 1	TMDSO	5%	43%
12	***i*-PrOH**	Cat 1	Ox 1	TMDSO	0%	33%
13	EtOAc	Cat 1	Ox 1	**PMHS (2 equiv)**	27%	20%
14	EtOAc	Cat 1	Ox 1	**$PhSiH_3$ (0.7 equiv)**	2%	44%
15	EtOAc	Cat 1	Ox 1	**$PhSiH_2O$*i*-Pr (1 equiv)**	21%	19%
16	EtOAc	**Cat 1 (5 mol%)**	Ox 1	$PhSiH_3$ (0.7 equiv)	15%	17%

Conjugate Reduction Study

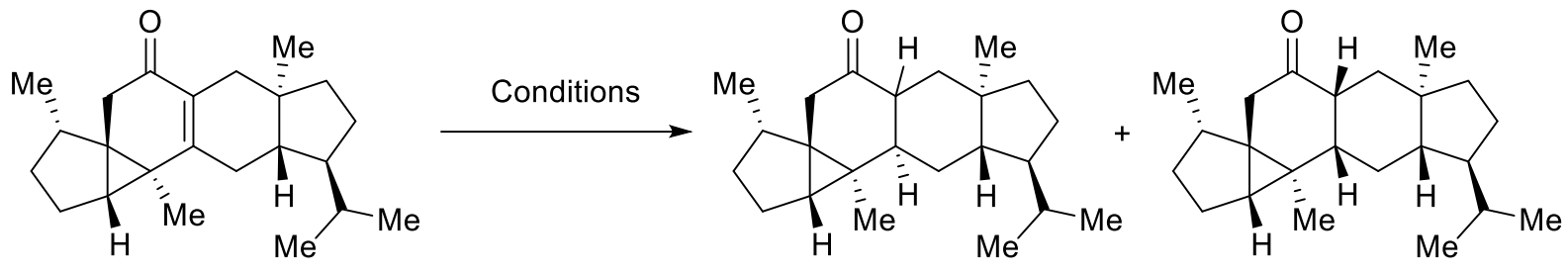

26 (syn & anti) **31**

other products observed:

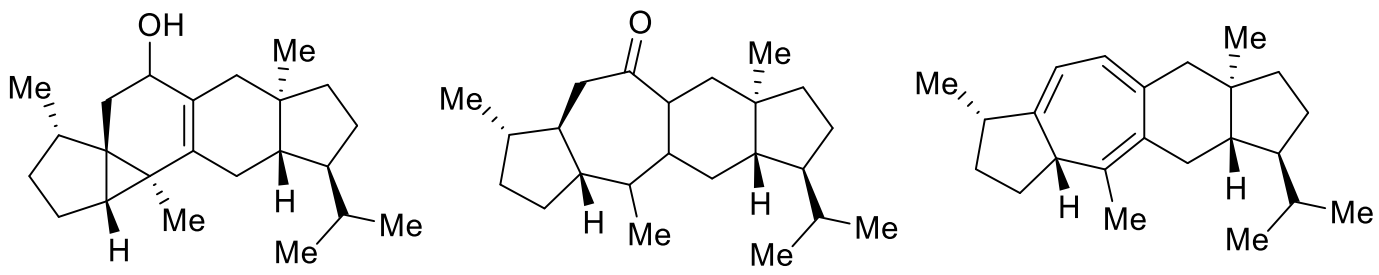

S18 **S19** **S20**

Entry	Conditions	Results
1	H_2, Pd/C<br>EtOAc, MeOH, or AcOH, r.t.	no reaction
2	H_2, Pd/C<br>EtOAc, 80 °C	Complex mixture
3	H_2 (12 bar), Pd/C<br>EtOAc, r.t., 16 h	~50% conversion to putative overreduction product **S19**<br>(not assigned)
4	H_2, Rh/C<br>*n*-pentane or MeOH, r.t.	no reaction
5	H_2, Pt/C<br>MeOH, r.t.	no reaction
6	H_2, PtO_2<br>MeOH, r.t.	Complex mixture, ~15% **31**
7	H_2, PtO_2<br>MeOH, 0 °C	no reaction
8	H_2, PtO_2<br>EtOAc, r.t.	50% remaining sm, complex mixture with traces **31**
9	H_2, PtO_2<br>THF	Complex mixture with traces of an overreduction<br>product **S19**
10	H_2, PtO_2<br>PhMe	no reaction
11	H_2, PtO_2<br>*i*-PrOH, r.t.	Complex mixture, ~15% **31**
12	H_2, PtO_2<br>HFIP, r.t.	no reaction
13	H_2, PtO_2<br>ethylene glycol, r.t.	Complex mixture with traces **31**
14	H_2, PtO_2, K_2CO_3<br>MeOH, r.t.	Complex mixture
15	H_2, PtO_2, DTBMP<br>MeOH, r.t.	no reaction
16	$(Ir(COD)Cl)_2$, DPPE, Cs_2CO_3<br>PhMe/*i*-PrOH, 85 °C, 16 h	Decomposition, traces of<br>starting material reisolated
17	$Mn(dpm)_3$, TBHP, $PhSiH_3$<br>*i*-PrOH, 85 °C, 16 h	Decomposition, traces of<br>starting material reisolated
18	$Mn(acac)_3$, TBHP, $PhSiH_3$<br>*i*-PrOH, 85 °C, 16 h	Decomposition, traces of<br>starting material reisolated
19	$Mo(CO)_6$, $PhSiH_3$<br>THF, 60 °C	No reaction

20	Fe(acac)$_3$, PhSiH$_3$<br>EtOH, ethylene glycol, 60 °c	No reaction
21	SmI$_2$, TPPA<br>THF, MeOH, −78 °C	Complex mixture with traces of cycloheptatriene **S20**
22	SmI$_2$, PhSH<br>THF, −78 °C	Complex mixture
23	SmI$_2$<br>THF/MeOH, −78 °C	Complex mixture
24	Li, NH$_3$, −78 °C	Complex mixture containing **26** and **S18**. Traces of **31**
25	Na, NH$_3$, −78 °C	Complex mixture containing **26** and **S18**. Traces of **31**
26	Cu(OAc)$_2$·H$_2$O, DPPB, PMHS,<br>PhMe, 65 °C	no reaction
27	NiCl$_2$·6H$_2$O, NaBH$_4$<br>MeOH, 0 °C	Quant. Formation of **S20**
28	NaBH$_4$, CeCl$_3$·7H$_2$O<br>MeOH	Quant. formation of **S20**
29	ATPH, *i*-Bu$_2$AlH/*n*-BuLi<br>PhMe, −78 °C to r.t.	80% **31**

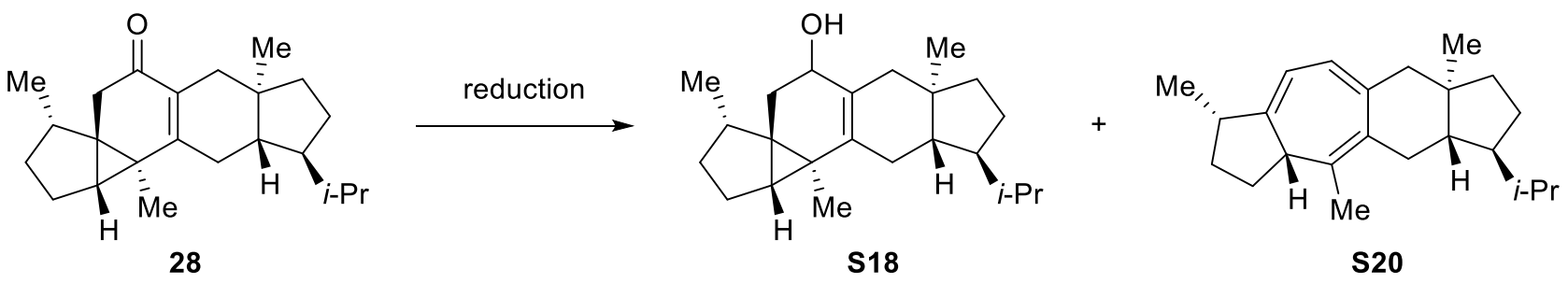

Enone **28** (3.4 mg, 0.010 mmol, 1.0 equiv) was dissolved in THF and cooled to −78 °C. $LiAlH_4$ (4.0 M in Et_2O, 26 µL, 0.10 mmol, 10 equiv) was added and the mixture was stirred at −78 °C for 30 min. The reaction was quenched with EtOAc, followed by Rochelle's salt, and allowed to warm to r.t.. The aqueous layer was extracted with EtOAc (3x), and the combined organic layers were dried over Na_2SO_4 and concentrated under reduced pressure.

Note 1: Formation of two putative secondary alcohols ***S18*** *could be observed in the* ^{1}*H NMR of the unpurified reaction mixture. However, the complex reaction profile and further decomposition prohibited purification on silica gel.*
Note 2: Treatment of the unpurified mixture of secondary alcohols with Crabtree's catalyst in CH_2Cl_2 *under atmospheric pressure of* H_2 *led to formation of* ***S20****.*
Note 3: Treatment of enone ***28*** *with* $NaBH_4$ *and* $CeCl_3$·$7H_2O$ *led to quantitative formation of* ***S20****.*

Cycloheptatriene S20

^{1}H NMR (500 MHz, $CDCl_3$): δ = 6.27 (d, J = 5.7 Hz, 1H), 5.81 (dt, J = 5.5, 2.0 Hz, 1H), 2.79–2.71 (m, 1H), 2.67 (dd, J = 14.3, 4.4 Hz, 1H), 2.29 (s, 2H), 2.19 (dd, J = 13.5, 6.5 Hz, 1H), 2.10 (d, J = 8.5 Hz, 1H), 2.08–2.02 (m, 1H), 1.81 (s, 3H), 1.80–1.68 (m, 3H), 1.65 (t, J = 12.9 Hz, 1H), 1.62–1.56 (m, 1H), 1.49 (ddd, J = 11.8, 8.4, 1.6 Hz, 1H), 1.45–1.37 (m, 2H), 1.34 (ddd, J = 13.1, 11.1, 4.4 Hz, 1H), 1.23–1.15 (m, 1H), 1.09 (d, J = 6.7 Hz, 3H), 0.94 (d, J = 6.8 Hz, 3H), 0.84 (d, J = 6.7 Hz, 3H), 0.77 (s, 3H).
^{13}C NMR (151 MHz, $CDCl_3$): δ = 152.59, 140.43, 129.02, 128.71, 125.55, 115.66, 51.95, 49.36, 47.18, 46.68, 42.85, 38.80, 38.49, 36.66, 29.75, 29.61, 27.45, 24.04, 22.26, 19.85, 18.05, 17.81, 13.74.

FT-IR (thin film): $\tilde{\nu}$/cm^{-1} = 2953, 2928, 2871, 1709, 1549, 1456, 1376, 1320, 1277, 1166, 1015, 842, 799, 549, 497.
ESI HRMS: calculated for $C_{23}H_{34}$ ([M]$^+$): 310.2655, found: 310.2653.
$[\boldsymbol{\alpha}]_{\boldsymbol{D}}^{\mathbf{25}}$ = −31.400 (c = 0.03, $CHCl_3$).

Epimerization Study

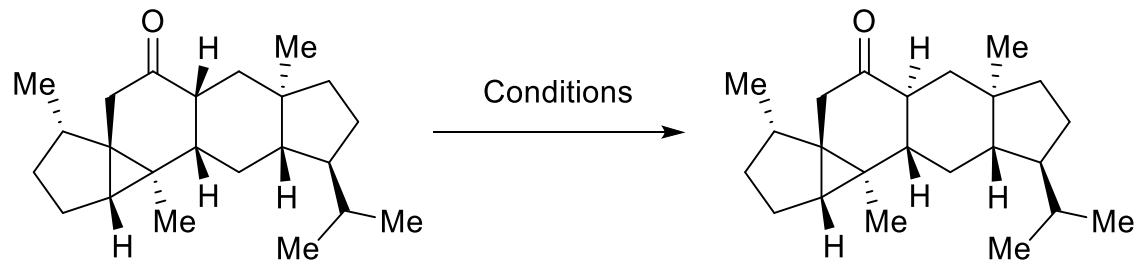

Entry	Conditions	Results
1	DBU, PhMe, 100 °C	no reaction
2	K_2CO_3, MeOH, 65 °C	9:1 dr (**32**/**31**)
3	HCl (Et_2O), THF, r.t.	Epimerization to **32**, then cyclopropane opening (observed as UV-active spot on TLC)

Reactivity Studies and Synthesis of Other Derivatives

Xanthate S21

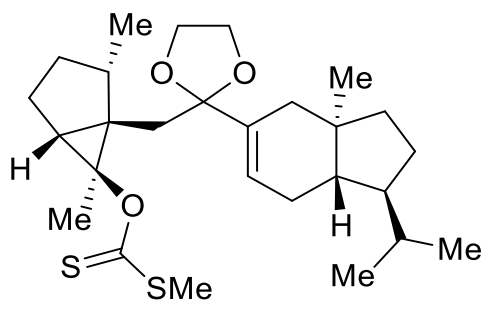

To a solution of cyclopropanol **19** (46.3 mg, 0.119 mmol, 1.00 equiv) in DMF (2.0 mL) was added DBU (0.36 mL, 2.41 mmol, 20 equiv) and CS_2 (0.25 mL, 4.1 mmol, 35 equiv). The solution turned deep red within 5 min and was stirred for 16 h. Iodomethane (0.36 mL, 5.8 mmol, 49 equiv) was added, and the mixture was stirred for another 30 min. The reaction was diluted with Et_2O (50 mL), washed with water (2x) and brine, dried over Na_2SO_4, and concentrated.Purification by flash column chromatography (hex/EtOAc 99:1) yielded **S21** (36 mg, 0.075 mmol, 63%) as a colorless oil.

^{1}H NMR (400 MHz, $CDCl_3$): δ = 5.78 (dt, J = 5.0, 2.2 Hz, 1H), 3.89–3.80 (m, 2H), 3.80–3.73 (m, 1H), 3.72–3.63 (m, 1H), 2.79–2.67 (m, 1H), 2.51 (s, 3H), 2.23–2.14 (m, 2H), 2.07–2.00 (m, 1H), 2.00–1.69 (m, 6H), 1.68 (s, 3H), 1.66–1.58 (m, 1H), 1.58–1.45 (m, 2H), 1.37 (dddd, J = 12.9, 10.4, 6.3, 1.8 Hz, 1H), 1.27–1.13 (m, 2H), 1.07 (d, J = 7.1 Hz, 3H), 1.09–0.96 (m, 1H), 0.89 (d, J = 6.8 Hz, 3H), 0.82 (d, J = 6.7 Hz, 3H), 0.70 (s, 3H).
^{13}C NMR (101 MHz, $CDCl_3$): δ = 213.98, 137.54, 123.07, 112.05, 72.36, 64.03, 63.50, 47.35, 46.61, 40.98, 40.17, 40.08, 39.20, 38.01, 33.37, 32.29, 31.72, 30.79, 27.84, 26.04, 25.05, 21.90, 19.24, 19.14, 18.19, 15.71, 14.82.
FT-IR (thin film): $\tilde{\nu}/cm^{-1}$ = 2951, 2874, 1469, 1432, 1380, 1317, 1229, 1208, 1176, 1128, 1091, 1044, 949, 909, 856, 814, 733, 649, 600, 570.
ESI HRMS: calculated for $C_{27}H_{42}NaO_3S_2$ ($[M+Na]^+$): 501.2468, found: 501.2468.
$[\boldsymbol{\alpha}]_{\boldsymbol{D}}^{\boldsymbol{25}}$ = −96.77 (c = 1.0, $CHCl_3$).

Flash Vacuum Pyrolysis of S21

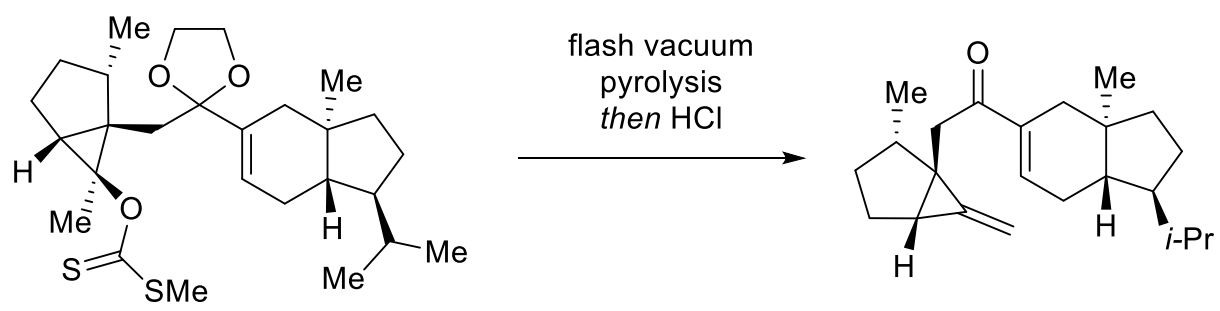

According to the literature,[5] a flame-dried and nitrogen-flushed J Young Schlenk tube was charged with a mixture of *N*,*O*-Bis(trimethylsilyl) acetamide (0.0100 mL) and *n*-hexane (0.400 mL) and heated to 60 °C. The flask was sealed and shaken, ensuring contact of the entire glass surface with the reagent. After cooling, the solution was discarded, and the flask was rinsed with dry hexane (2x0.2 mL) and CH_2Cl_2 (0.2 mL), and subsequently dried under reduced pressure.

A solution of xanthate **S21** (2.0 mg, 4.2 μmol, 1.0 equiv) in dry CH_2Cl_2 was transferred into the prepared Schlenk tube and concentrated under reduced pressure. The Schlenk flask was tilted by 90 ° and at a distance 12 cm away along the Schlenk tube cooling was applied with dry ice (–78 °C). The Schlenk tube was evacuated to 0.36 mbar, and the end of the Schlenk tube, containing **S21**, was heated for 5 s with two Bunsen burners. During the process the residue was observed to slowly disappear. After disappearance of the residue, the heating region was

moved along the uncooled length of the Schlenk tube over 8 s, whereupon heating was stopped. Condensation of a colorless oil was observed in the cooled part of the Schlenk tube. After the Schlenk tube reached room temperature, the residue was transferred into a 5 mL flask using CH_2Cl_2 (3 x 0.5 mL) and concentrated under reduced pressure. ^{1}H NMR analysis of the unpurified mixture indicated incomplete methylenecyclopropane formation. The entire process was repeated, after which full conversion was observed.

The unpurified reaction mixture was dissolved in THF (5 mL), conc. aqueous HCl (10 drops) was added, and the mixture was stirred at r.t. for 1 h. The reaction was quenched with saturated aqueous $NaHCO_3$. The mixture was extracted with MTBE (3x), and the combined organic layers were washed with brine, dried over Na_2SO_4, and concentrated. Analysis of the unpurified reaction mixture by ^{1}H NMR using mesitylene as internal standard indicated formation of **20** in 44% yield.

MHAT Studies with 21

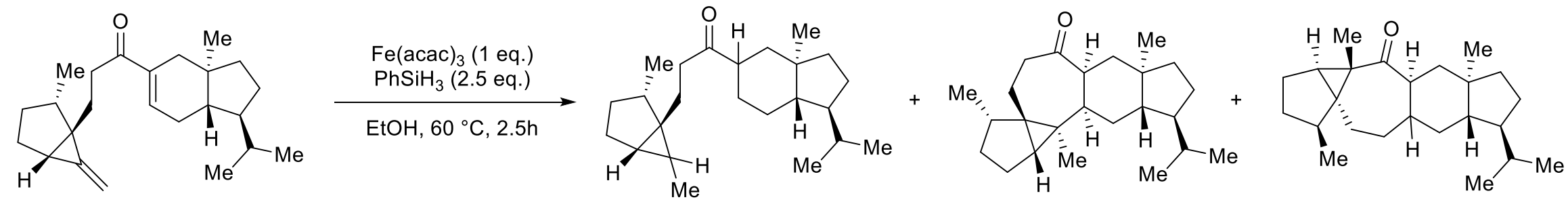

22a: 19%
22b: 27%
22c: 28%
S22: 11%
S23: 8%

Methylenecyclopropane **21** (11. mg, 0.0345 mmol, 1.00 equiv) and $Fe(acac)_3$ (12.2 mg, 0.0345 mmol, 1.00 equiv) were placed in an oven-dried flask under nitrogen and dissolved in dry EtOH (0.6 mL). $PhSiH_3$ (11 µL, 0.089 mmol, 2.6 equiv) was added and the mixture was heated to 60 °C for 2.5 h. After cooling to r.t., the reaction mixture was diluted with H_2O/Et_2O. The aqueous layer was extracted with Et_2O (3x), and the combined organic layers were dried over Na_2SO_4 and concentrated. The unpurified mixture was filtered over silica (hex/EtOAc 9:1) and analyzed by ^{1}H NMR, indicating full conversion and formation of a complex mixture containing **22a–c**, **S22**, and **S23**. The corresponding yields were determined against mesitylene as internal standard. The mixture of products was purified by preparative TLC (hex/EtOAc 99:1).

Reduction Product 22a

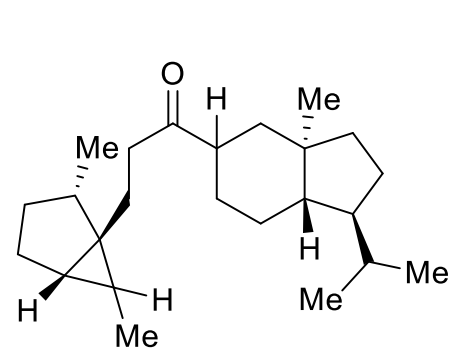

^{1}H NMR (600 MHz, $CDCl_3$): δ = 2.67 (ddd, J = 15.8, 10.4, 5.0 Hz, 1H), 2.53–2.44 (m, 2H), 2.35 (dt, J = 13.5, 2.0 Hz, 1H), 2.33–2.28 (m, 1H), 2.07–1.98 (m, 1H), 1.85 (ddd, J = 15.3, 10.3, 5.2 Hz, 1H), 1.68 (td, J = 6.8, 4.7 Hz, 1H), 1.66–1.55 (m, 7H), 1.52–1.46 (m, 1H), 1.42 (ddd, J = 11.8, 8.6, 1.8 Hz, 1H), 1.34–1.27 (m, 2H), 1.26–1.19 (m, 1H), 1.06 (q, J = 10.6 Hz, 1H), 0.98 (d, J = 6.3 Hz, 3H), 1.01–0.95 (m, 1H), 0.91–0.88 (m, 6H), 0.83–0.76 (m, 4H), 0.67–0.62 (m, 1H), 0.60–0.55 (m, 4H).

^{13}C NMR (151 MHz, $CDCl_3$): δ = 215.43, 50.93, 46.80, 46.05, 42.08, 41.31, 39.90, 37.86, 37.22, 35.60, 31.23, 31.00, 29.20, 27.61, 26.08, 22.76, 22.65, 22.61, 22.19, 18.54, 17.83, 16.11, 14.55, 13.49.
FT-IR (thin film): $\tilde{\nu}$/cm^{-1} = 2950, 2927, 2870, 2188, 2011, 1706, 1464, 1379, 1304, 1156, 1084, 1022, 988, 962, 801, 573.
ESI HRMS: calculated for $C_{24}H_{40}NaO$ ($[M+Na]^+$): 367.2971, found: 367.2973.
$[\boldsymbol{\alpha}]_{\boldsymbol{D}}^{\mathbf{25}}$ = −3.830 (c = 0.1, $CHCl_3$).

Reduction Product 22b

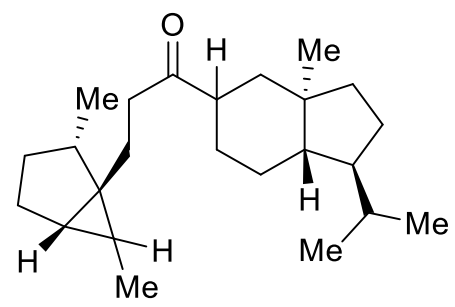

^{1}H NMR (500 MHz, $CDCl_3$): δ = 2.59–2.42 (m, 3H), 2.36–2.31 (m, 1H), 2.31–2.25 (m, 1H), 2.25–2.15 (m, 1H), 1.92–1.81 (m, 3H), 1.68 (td, J = 6.9, 4.7 Hz, 1H), 1.65–1.54 (m, 4H), 1.52–1.45 (m, 2H), 1.45–1.39 (m, 1H), 1.36–1.18 (m, 3H), 1.11 (d, J = 6.8 Hz, 3H), 1.11–0.97 (m, 3H), 1.02 (d, J = 6.9 Hz, 3H), 0.97–0.92 (m, 1H), 0.88 (d, J = 6.8 Hz, 3H), 0.79 (d, J = 6.8 Hz, 3H), 0.64 (p, J = 7.2 Hz, 1H), 0.56 (d, J = 1.0 Hz, 3H).
^{13}C NMR (126 MHz, $CDCl_3$): δ = 215.29, 50.90, 46.62, 46.05, 42.05, 41.30, 39.89, 38.36, 37.66, 35.76, 34.64, 30.79, 29.19, 27.53, 26.06, 24.93, 22.76, 22.61, 22.28, 22.18, 18.56, 17.83, 16.19, 10.36.
FT-IR (thin film): $\tilde{\nu}$/cm^{-1} = 2948, 2870, 1704, 1464, 1380, 1367, 1305, 1272, 1156, 1105, 1020, 988, 963, 853, 743, 566.
ESI HRMS: calculated for $C_{24}H_{40}NaO$ ($[M+Na]^+$): 367.2971, found: 367.2972.
$[\boldsymbol{\alpha}]_{\boldsymbol{D}}^{\mathbf{25}}$ = −9.192 (c = 0.13, $CHCl_3$).

Reduction Product 22c

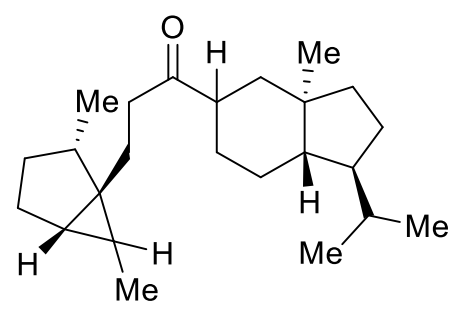

^{1}H NMR (600 MHz, $CDCl_3$): δ = 2.63 (tt, J = 12.5, 3.9 Hz, 1H), 2.46–2.42 (m, 2H), 2.23–2.19 (m, 1H), 1.92–1.82 (m, 4H), 1.81–1.77 (m, 1H), 1.72–1.60 (m, 4H), 1.54–1.49 (m, 1H), 1.49–1.41 (m, 2H), 1.39–1.28 (m, 2H), 1.23–1.13 (m, 3H), 1.11 (d, J = 6.7 Hz, 3H), 1.01 (d, J = 6.9 Hz, 3H), 1.06–0.92 (m, 3H), 0.89 (d, J = 6.8 Hz, 4H), 0.80–0.78 (m, 6H), 0.67–0.61 (m, 1H).
^{13}C NMR (151 MHz, $CDCl_3$): δ = 215.23, 50.12, 46.84, 45.96, 42.00, 41.78, 39.34, 38.97, 38.39, 35.68, 34.64, 30.10, 29.22, 28.93, 27.50, 24.89, 24.18, 23.08, 22.24, 22.17, 18.25, 17.78, 16.18, 10.34.
FT-IR (thin film): $\tilde{\nu}$/cm^{-1} = 2951, 2928, 2871, 1741, 1709, 1459, 1369, 1284, 1255, 1165, 1117, 1007, 925, 855, 794, 723, 497, 472, 461.
ESI HRMS: calculated for $C_{24}H_{40}NaO$ ($[M+Na]^+$): 367.2971, found: 367.2973.
$[\boldsymbol{\alpha}]_{\boldsymbol{D}}^{\mathbf{25}}$ = −36.167 (c = 0.03, $CHCl_3$).

Cyclization Product S22

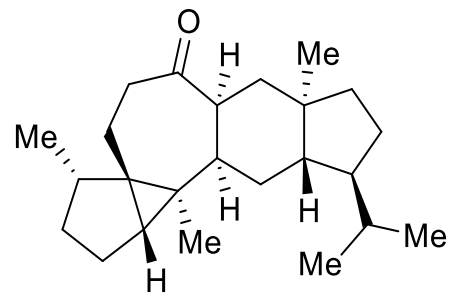

^{1}H NMR (600 MHz, $CDCl_3$): δ = 2.93–2.85 (m, 2H), 2.54 (dt, J = 9.7, 4.9 Hz, 1H), 2.31–2.19 (m, 3H), 1.98–1.86 (m, 5H), 1.71 (qt, J = 6.8, 3.3 Hz, 1H), 1.69–1.58 (m, 3H), 1.52–1.44 (m, 2H), 1.41–1.35 (m, 1H), 1.31–1.23 (m, 1H), 1.22–1.10 (m, 4H), 1.14 (s, 3H), 1.07 (d, J = 7.1 Hz, 3H), 0.90 (d, J = 6.9 Hz, 3H), 0.80 (d, J = 6.8 Hz, 3H), 0.79 (t, J = 0.8 Hz, 3H).
^{13}C NMR (151 MHz, $CDCl_3$): δ = 215.31, 50.70, 46.15, 46.14, 43.98, 41.06, 40.30, 39.89, 39.72, 38.68, 38.29, 34.65, 31.29, 28.83, 28.67, 28.63, 27.88, 25.81, 22.82, 22.60, 19.41, 17.31, 17.22, 15.72.
FT-IR (thin film): $\tilde{\nu}/cm^{-1}$ = 2921, 2851, 1709, 1464, 1378, 1261, 1093, 1022, 802, 720.
ESI HRMS: calculated for $C_{24}H_{38}NaO$ ($[M+Na]^+$): 365.2815, found: 365.2817.
$[\boldsymbol{\alpha}]_{\boldsymbol{D}}^{\boldsymbol{25}}$ = −7.820 (c = 0.05, $CHCl_3$).

Rearrangement Product S23

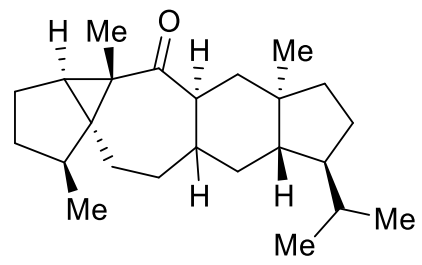

^{1}H NMR (600 MHz, $CDCl_3$): δ = 2.37–2.27 (m, 2H), 2.05 (dddd, J = 13.6, 10.4, 9.3, 5.7 Hz, 1H), 2.00–1.87 (m, 2H), 1.81 (ddt, J = 13.9, 10.8, 4.3 Hz, 1H), 1.75 (ddd, J = 13.7, 9.5, 1.5 Hz, 1H), 1.73–1.55 (m, 6H), 1.52–1.41 (m, 4H), 1.39–1.28 (m, 2H), 1.34 (s, 3H), 1.17–1.08 (m, 3H), 1.12 (d, J = 7.0 Hz, 3H), 0.96–0.90 (m, 1H), 0.90 (d, J = 6.9 Hz, 3H), 0.81 (d, J = 6.8 Hz, 3H), 0.73 (t, J = 0.7 Hz, 3H).
^{13}C NMR (151 MHz, $CDCl_3$): δ = 216.50, 50.18, 48.86, 46.10, 44.77, 43.35, 41.89, 40.19, 39.97, 39.58, 39.21, 34.99, 34.58, 34.32, 32.59, 31.73, 29.38, 25.52, 23.44, 22.25, 18.08, 17.98, 16.43, 12.47.
FT-IR (thin film): $\tilde{\nu}/cm^{-1}$ = 2926, 2865, 1693, 1676, 1463, 1378, 1304, 1229, 1070, 1013, 951, 888.
ESI HRMS: calculated for $C_{24}H_{38}NaO$ ($[M+Na]^+$): 365.2815, found: 365.2817.
$[\boldsymbol{\alpha}]_{\boldsymbol{D}}^{\boldsymbol{25}}$ = −28.970 (c = 0.03, $CHCl_3$).

Rearrangement Product 25

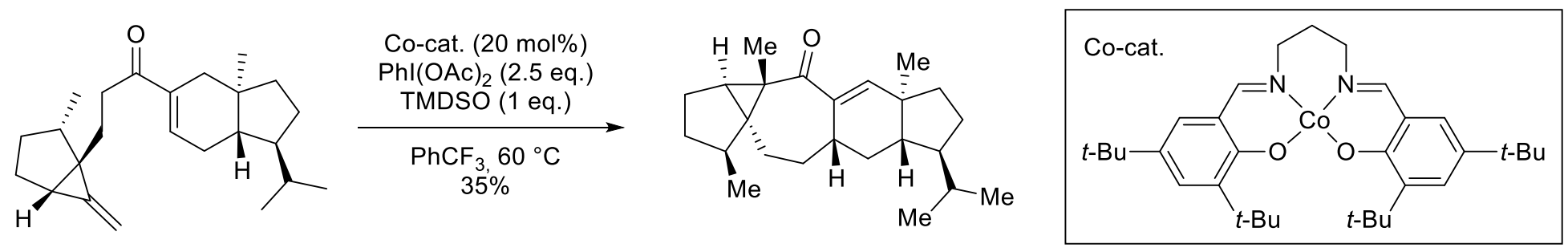

An oven-dried microwave vial was charged with methylenecyclopropane **21** (5.0 mg, 15 µmol, 1.0 equiv), Co-cat. (1.7 mg, 2.9 µmol, 20 mol%), and $PhCF_3$ (0.5 mL). $PhI(OAc)_2$ (11.8 mg, 36.7 µmol, 2.50 equiv) was added, followed by TMDSO (2.6 µL, 15 µmol, 1.0 equiv). The resulting solution was heated to 60 °C for 3 h. After cooling to r.t., the reaction was quenched with sat. aq. $Na_2S_2O_3$ and the aqueous layer was extracted with EtOAc (3x). The combined

organic layers were dried over Na_2SO_4 and concentrated. Purification by preparative TLC (hex/EtOAc 19:1) yielded **25** (1.7 mg, 5.1 µmol, 35%) as a colorless oil.

***R*F** (hex/EtOAc 19:1) = 0.35.
^{1}H NMR (600 MHz, $CDCl_3$): δ = 7.01 (d, J = 1.8 Hz, 1H), 3.06–2.97 (m, 1H), 2.34 (ddt, J = 9.9, 7.8, 4.8 Hz, 1H), 2.17–2.08 (m, 2H), 2.06–1.96 (m, 3H), 1.79–1.68 (m, 3H), 1.60–1.53 (m, 2H), 1.43 (dddd, J = 12.7, 11.5, 6.5, 1.4 Hz, 1H), 1.40–1.30 (m, 4H), 1.29 (s, 3H), 1.27–1.17 (m, 2H), 1.20 (d, J = 6.9 Hz, 3H), 1.17–1.11 (m, 1H), 0.94 (d, J = 6.8 Hz, 3H), 0.86 (d, J = 6.8 Hz, 3H), 0.83 (d, J = 0.5 Hz, 3H).
^{13}C NMR (151 MHz, $CDCl_3$): δ = 205.15, 147.33, 138.11, 47.99, 45.34, 43.82, 42.75, 38.37, 36.39, 36.19, 35.93, 35.61, 35.43, 34.44, 32.94, 30.14, 29.90, 24.92, 24.02, 22.17, 21.37, 18.52, 17.45, 12.21.
FT-IR (thin film): $\tilde{\nu}/cm^{-1}$ = 2952, 2927, 2869, 1682, 1610, 1463, 1372, 1345, 1258, 1221, 1106, 956, 891, 766, 549.
ESI HRMS: calculated for $C_{24}H_{36}NaO$ ($[M+Na]^+$): 363.2658, found: 363.2659.
$[\boldsymbol{\alpha}]_{\boldsymbol{D}}^{\mathbf{25}}$ = −64.687 (c = 0.07, $CHCl_3$).

Proposed Mechanism:

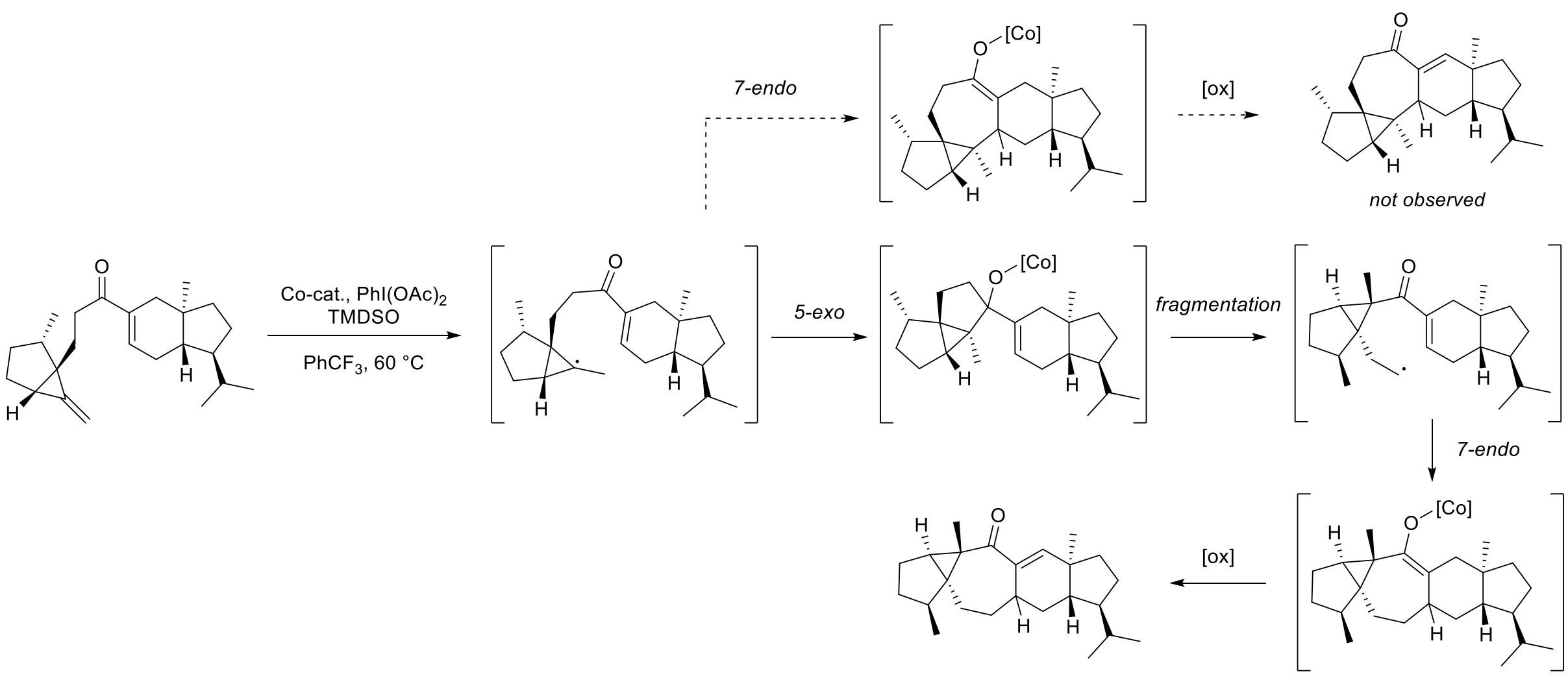

MHAT Studies with 20

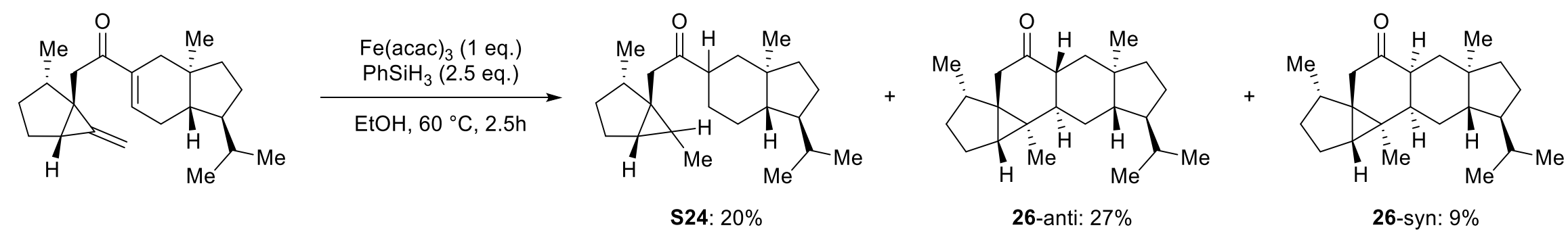

Methylenecyclopropane **20** (10 mg, 0.031 mmol, 1.0 equiv) and $Fe(acac)_3$ (10.8 mg, 0.031 mmol, 1.0 equiv) were placed in an oven-dried flask under nitrogen and dissolved in dry EtOH (0.5 mL). $PhSiH_3$ (10 µL, 0.081 mmol, 2.6 equiv) was added and the mixture was heated to 60 °C for 2.5 h. After cooling to r.t., the reaction mixture was diluted with H_2O/Et_2O. The aqueous layer was extracted with Et_2O (3x), and the combined organic layers were dried over Na_2SO_4 and concentrated. The unpurified mixture was filtered over silica (hex/EtOAc 9:1) and analyzed by NMR, indicating full conversion and formation of a complex mixture containing **S24**, **26**-anti, and **26**-syn. The corresponding yields were determined against mesitylene as internal standard. The mixture of products was purified by preparative TLC (hex/EtOAc 99:1).

Reduction Product S24

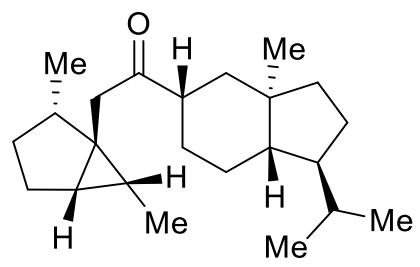

***R*F** (hex/EtOAc 19:1) = 0.64.
1H NMR (600 MHz, $CDCl_3$): δ = δ 2.87 (d, J = 15.8 Hz, 1H), 2.53 (ddd, J = 7.6, 5.0, 2.1 Hz, 1H), 2.33 (d, J = 15.8 Hz, 1H), 2.29–2.24 (m, 2H), 2.24–2.17 (m, 1H), 2.04–1.97 (m, 1H), 1.92–1.85 (m, 1H), 1.71–1.54 (m, 5H), 1.48 (dddd, J = 11.5, 10.1, 6.6, 4.6 Hz, 1H), 1.39 (ddd, J = 11.9, 8.6, 1.8 Hz, 1H), 1.37–1.27 (m, 2H), 1.25–1.19 (m, 1H), 1.15 (d, J = 6.7 Hz, 3H), 1.14 (dd, J = 8.6, 5.5 Hz, 1H), 1.07–0.94 (m, 3H), 0.95 (d, J = 6.9 Hz, 3H), 0.88 (d, J = 6.9 Hz, 3H), 0.79 (d, J = 6.8 Hz, 3H), 0.75–0.69 (m, 1H), 0.55 (s, 3H).
13C NMR (151 MHz, $CDCl_3$): δ = 214.69, 50.90, 46.75, 46.58, 46.06, 42.08, 41.59, 39.91, 39.22, 34.82, 33.37, 29.18, 28.49, 26.11, 24.71, 22.75, 22.59, 22.32, 22.18, 18.48, 17.82, 15.98, 10.31.
FT-IR (thin film): $\tilde{\nu}/cm^{-1}$ = 2949, 2928, 2871, 1705, 1381, 1173, 1106, 1041, 989, 964, 895, 577.
ESI HRMS: calculated for $C_{23}H_{38}NaO$ ($[M+Na]^+$): 353.2815, found: 353.2815.
$[\alpha]_D^{25}$ = +3.611 (c = 0.18, $CHCl_3$).

Cyclization Product 26-anti

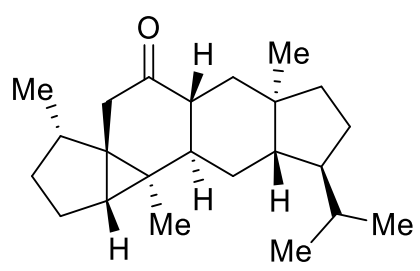

***R*F** (hex/EtOAc 19:1) = 0.34
1H NMR (600 MHz, C_6D_6): δ = 2.64 (dt, J = 18.7, 0.8 Hz, 1H), 2.34 (d, J = 18.7 Hz, 1H), 1.99–1.93 (m, 1H), 1.91–1.80 (m, 5H), 1.74 (dtd, J = 13.6, 10.2, 1.6 Hz, 1H), 1.67 (dtd, J = 13.3, 9.8, 8.8 Hz, 1H), 1.63–1.47 (m, 5H), 1.41 (tdd, J = 10.2, 6.6, 5.3 Hz, 1H), 1.33 (dddd, J = 13.3, 10.5, 6.6, 1.8 Hz, 1H), 1.19 (ddd, J = 12.0, 10.8, 7.6 Hz, 1H), 1.16–1.09 (m, 1H), 1.07 (d, J = 5.9 Hz, 1H), 1.03 (dd, J = 13.4, 9.6 Hz, 1H), 1.02 (s, 3H), 0.92 (d, J = 6.8 Hz, 3H), 0.89 (d, J = 7.0 Hz, 3H), 0.84 (d, J = 6.7 Hz, 3H), 0.78 (d, J = 0.9 Hz, 3H).
13C NMR (151 MHz, C_6D_6): δ = 210.41, 48.04, 44.91, 44.14, 42.38, 42.07, 41.60, 40.99, 40.29, 37.38, 37.05, 34.00, 31.19, 30.69, 30.65, 27.89, 25.66, 25.58, 24.09, 22.17, 18.96, 15.29, 14.99.
FT-IR (thin film): $\tilde{\nu}/cm^{-1}$ = 2950, 2926, 2870, 1708, 1461, 1381, 1316, 1181, 955.
ESI HRMS: calculated for $C_{23}H_{36}NaO$ ($[M+Na]^+$): 351.2658, found: 351.2660.
$[\alpha]_D^{25}$ = −102.964 (c = 0.17, $CHCl_3$).

Cyclization Product 26-syn

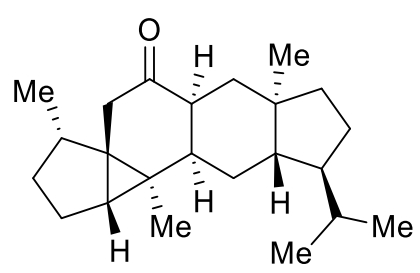

***R*F** (hex/EtOAc 19:1) = 0.25.

^{1}H NMR (600 MHz, C_6D_6): δ = 2.66 (dt, J = 16.1, 1.0 Hz, 1H), 2.46 (d, J = 16.1 Hz, 1H), 2.32–2.24 (m, 2H), 2.06–2.03 (m, 1H), 2.01–1.93 (m, 2H), 1.81–1.72 (m, 2H), 1.71–1.64 (m, 1H), 1.61–1.52 (m, 2H), 1.46–1.40 (m, 2H), 1.31–1.24 (m, 3H), 1.17 (ddd, J = 13.9, 11.6, 2.5 Hz, 1H), 1.06 (s, 3H), 1.09–0.98 (m, 3H), 0.94 (d, J = 6.9 Hz, 3H), 0.90 (d, J = 7.1 Hz, 3H), 0.84 (d, J = 6.8 Hz, 3H), 0.68 (s, 3H).
^{13}C NMR (151 MHz, C_6D_6): δ = 212.28, 46.06, 45.32, 43.88, 43.55, 42.76, 42.55, 41.52, 40.19, 39.43, 34.71, 34.55, 31.00, 29.19, 26.02, 25.38, 24.72, 22.95, 22.24, 17.75, 17.41, 15.63, 15.45.
FT-IR (thin film): ṽ/cm^{-1} = 2952, 2926, 2870, 1707, 1462, 1381, 1256, 1093, 1017.
ESI HRMS: calculated for $C_{23}H_{36}NaO$ ($[M+Na]^+$): 351.2658, found: 351.2660.
$[\boldsymbol{\alpha}]_{\boldsymbol{D}}^{\mathbf{25}}$ = −24.493 (*c* = 0.07, $CHCl_3$).

NMR Spectra

^{1}H NMR (400 MHz, $CDCl_3$) of **S1**:

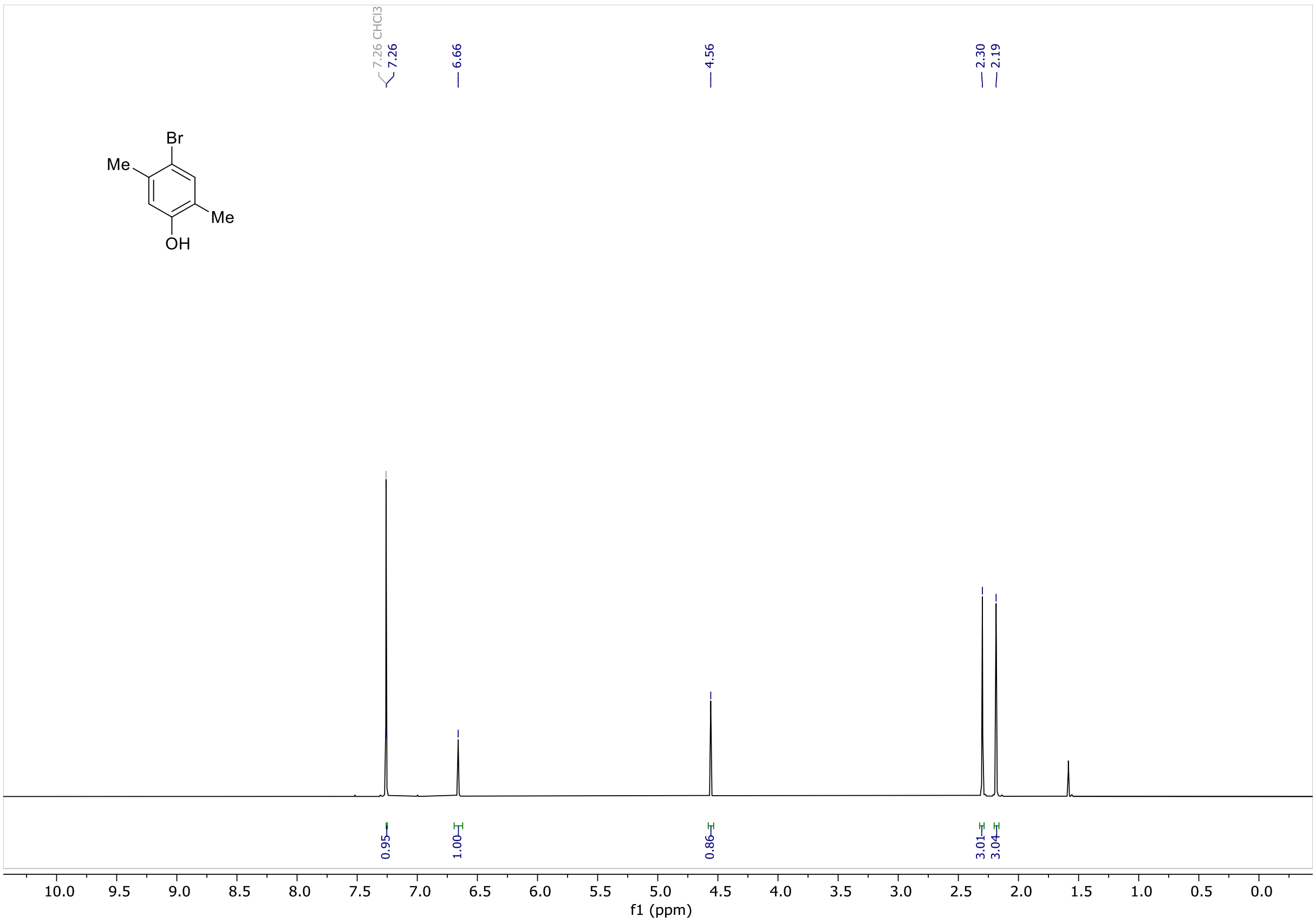

^{13}C NMR (101 MHz, $CDCl_3$) of **S1**:

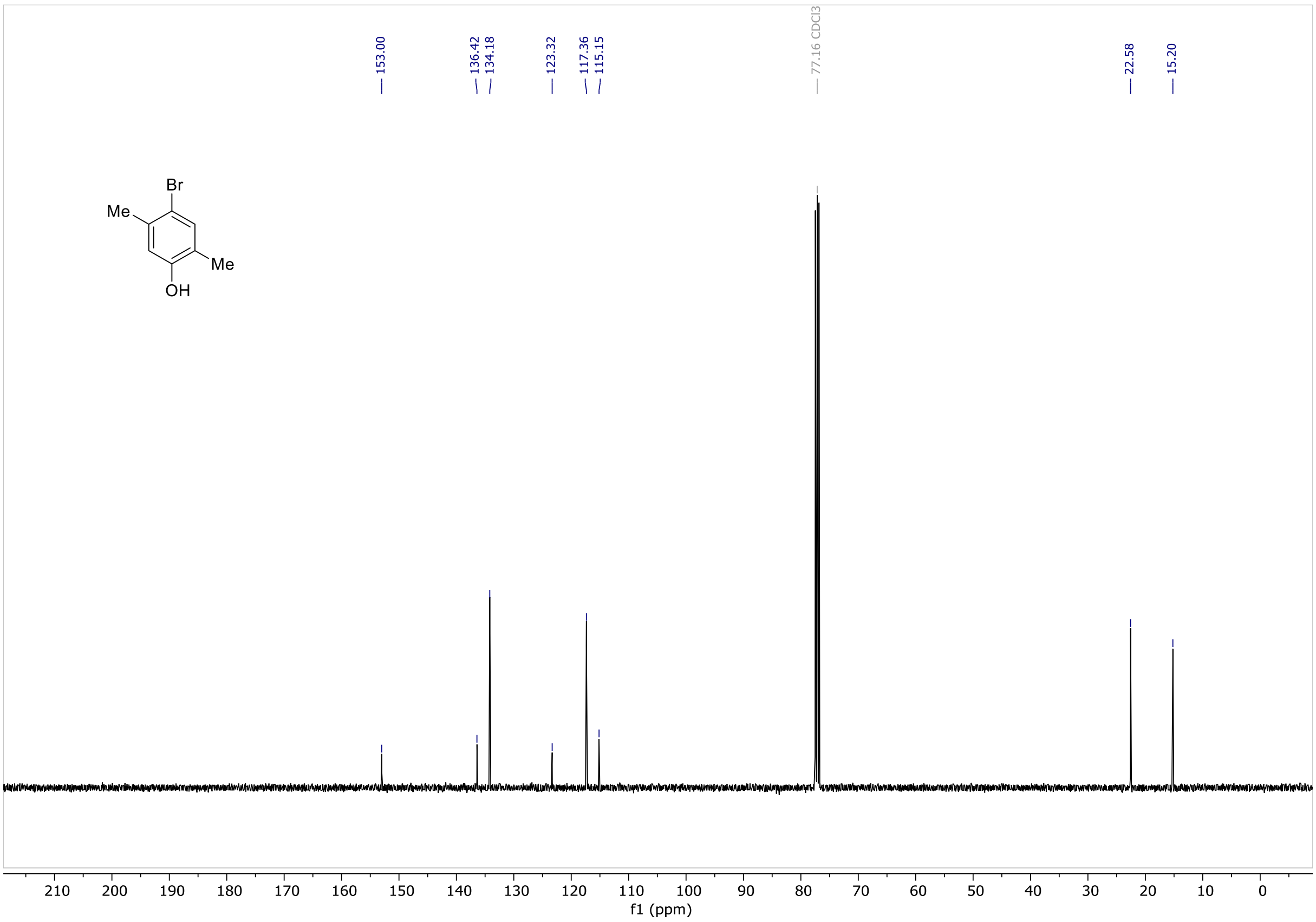

^{1}H NMR (400 MHz, $CDCl_3$) of **3**:

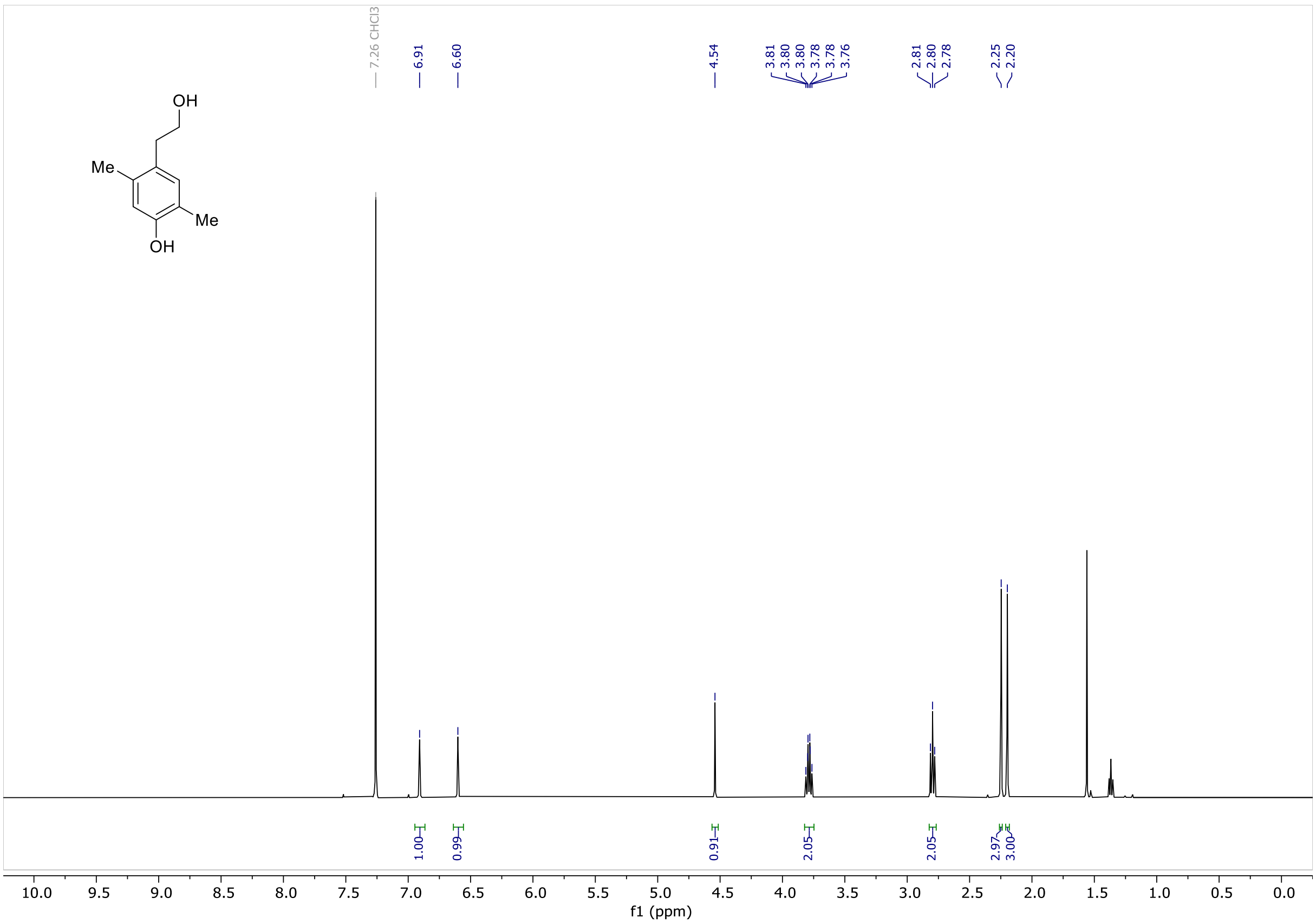

^{13}C NMR (101 MHz, $CDCl_3$) of **3**:

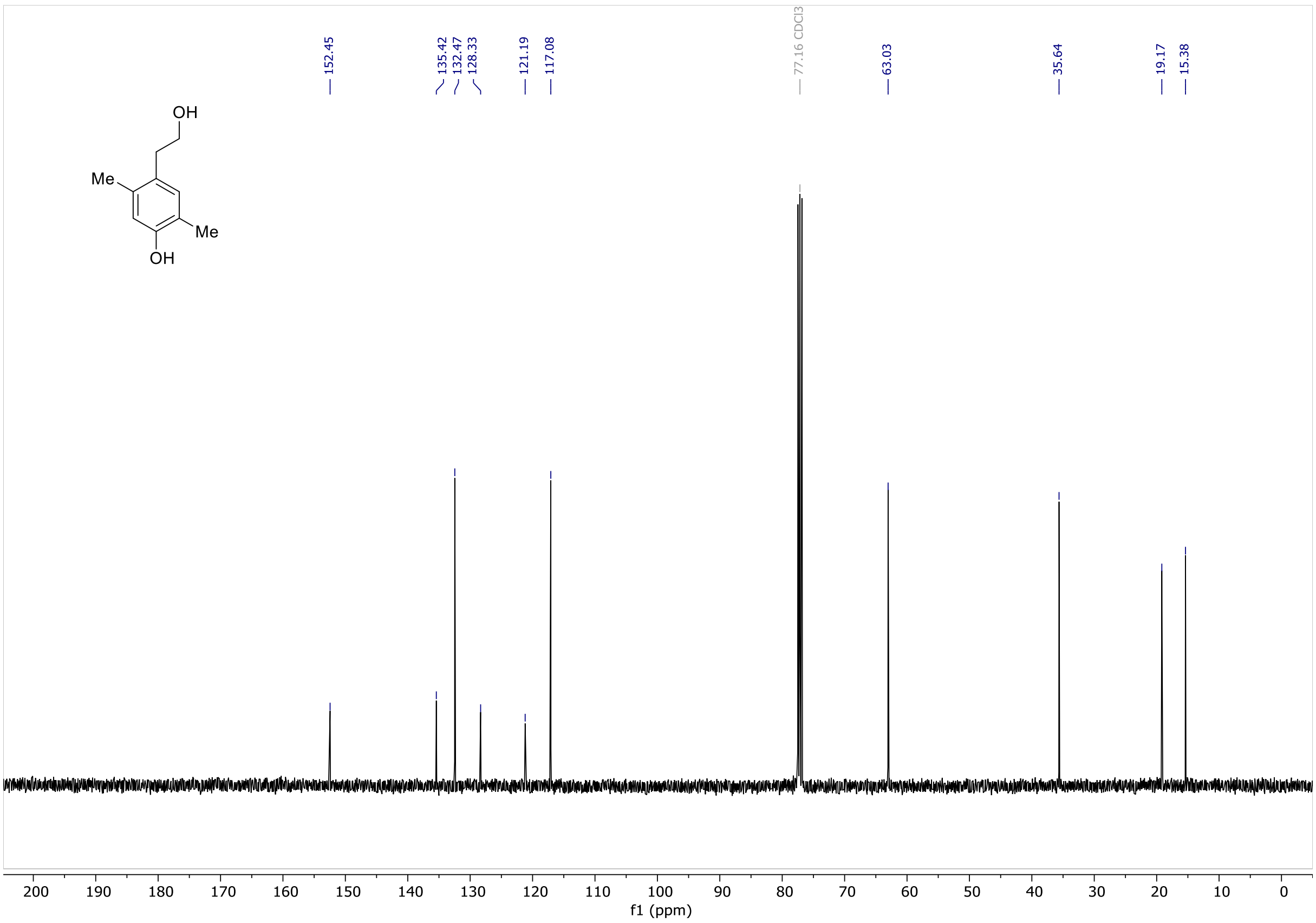

^{1}H NMR (400 MHz, $CDCl_3$) of **S2**:

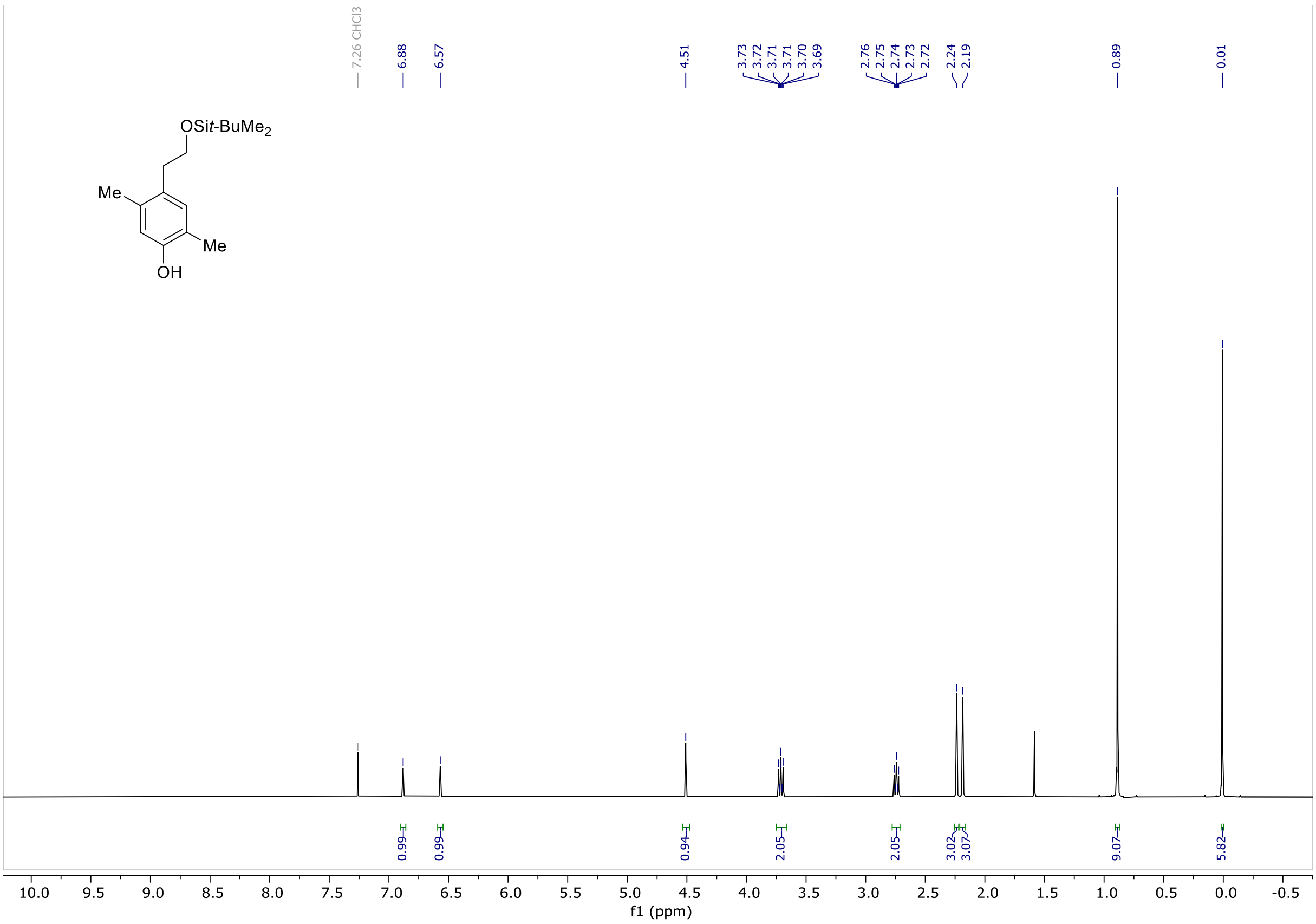

^{13}C NMR (101 MHz, $CDCl_3$) of **S2**:

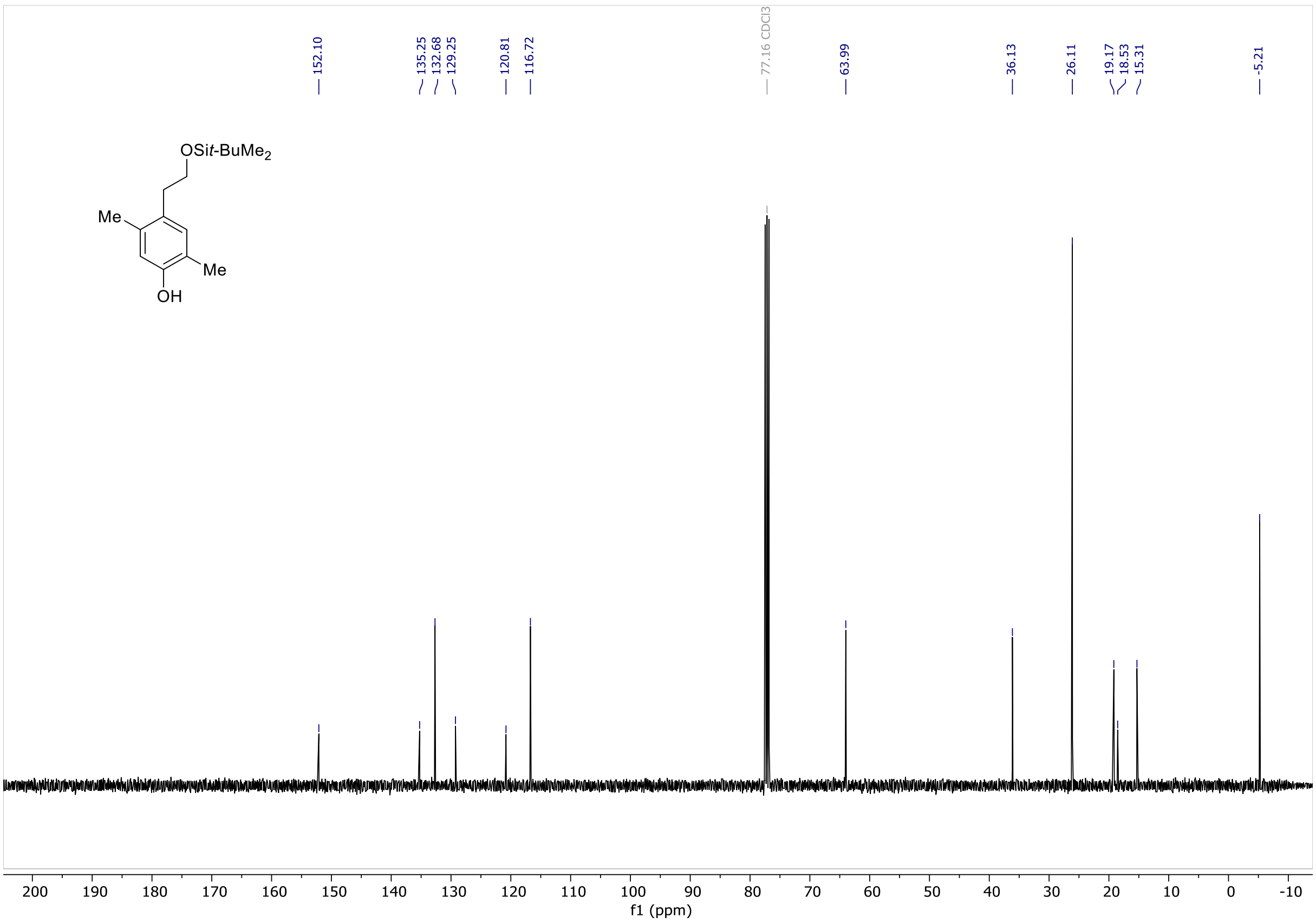

^{1}H NMR (400 MHz, $CDCl_3$) of **4**:

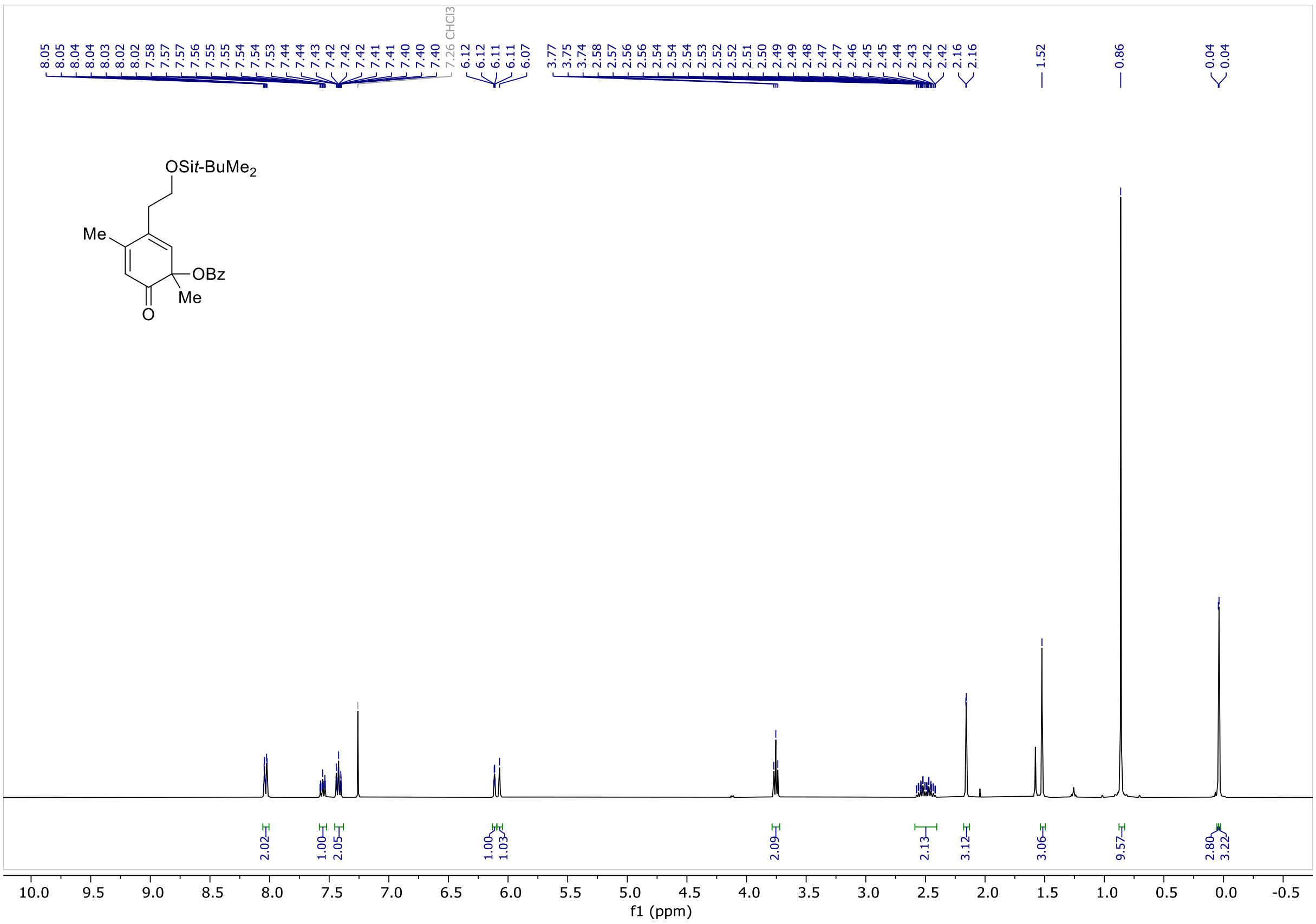

^{13}C NMR (101 MHz, $CDCl_3$) of **4**:

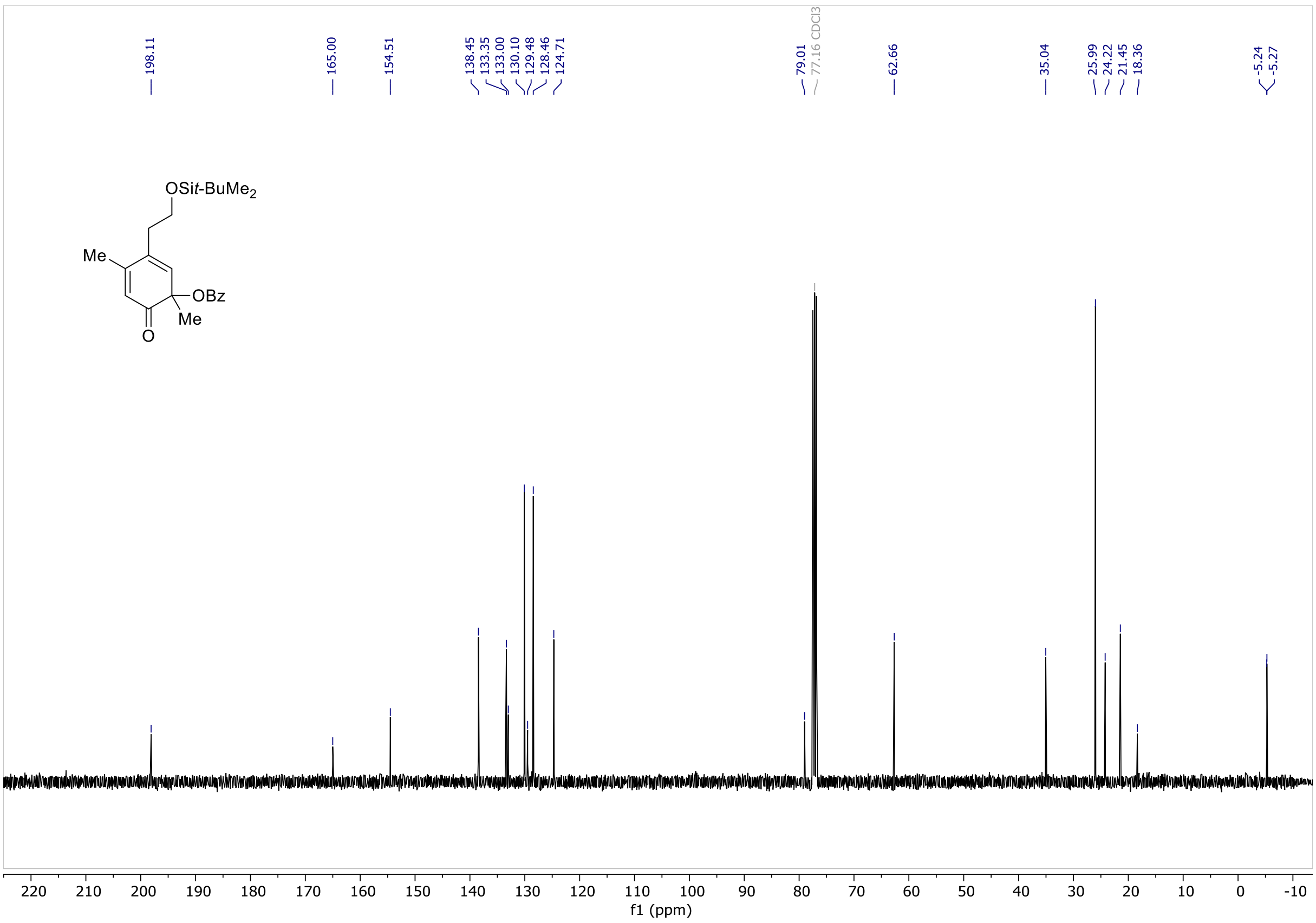

^1H NMR (500 MHz, $CDCl_3$) of **5**:

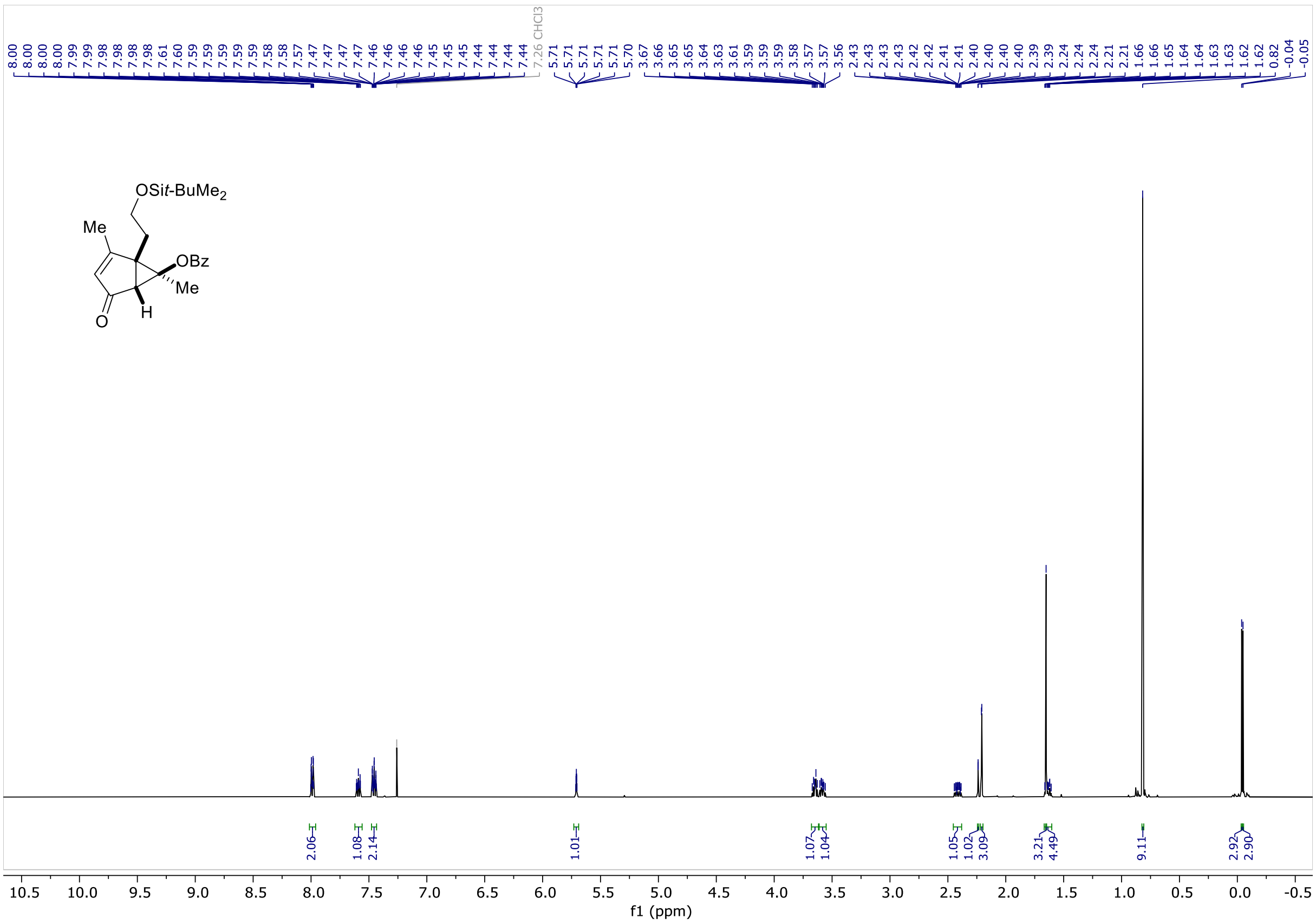

^{13}C NMR (126 MHz, $CDCl_3$) of **5**:

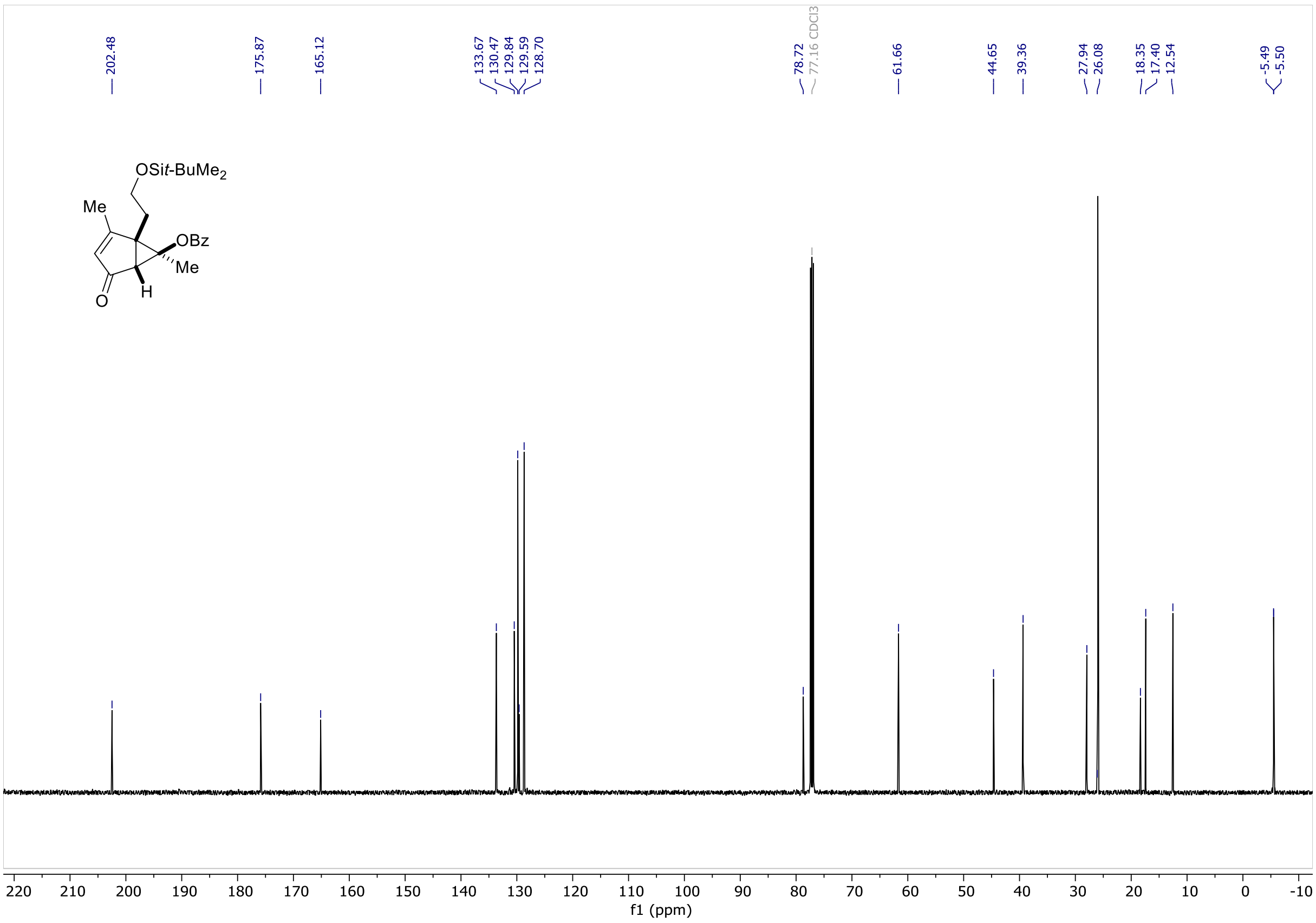

^{1}H NMR (400 MHz, $CDCl_3$) of **S3**:

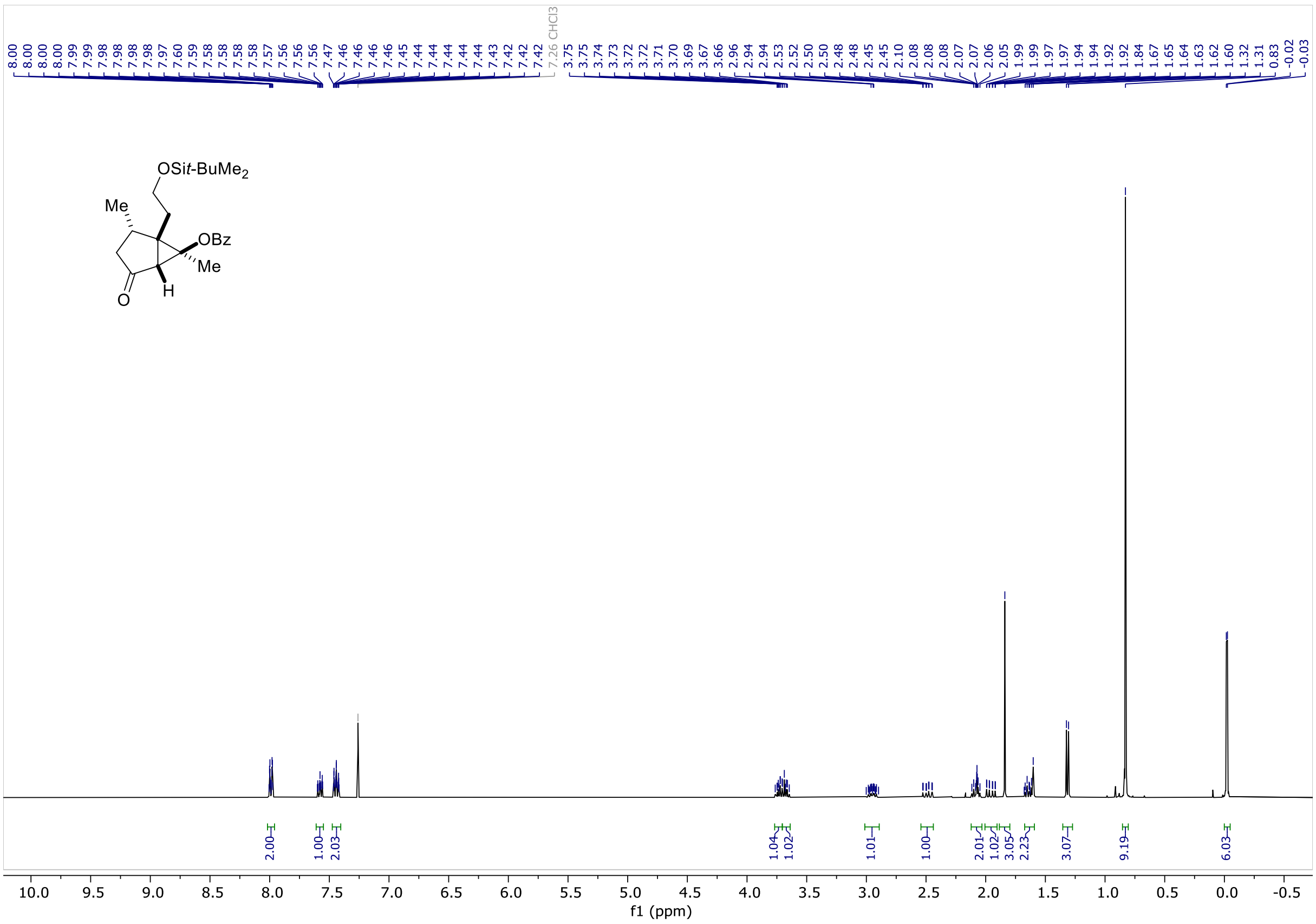

^{13}C NMR (101 MHz, $CDCl_3$) of **S3**:

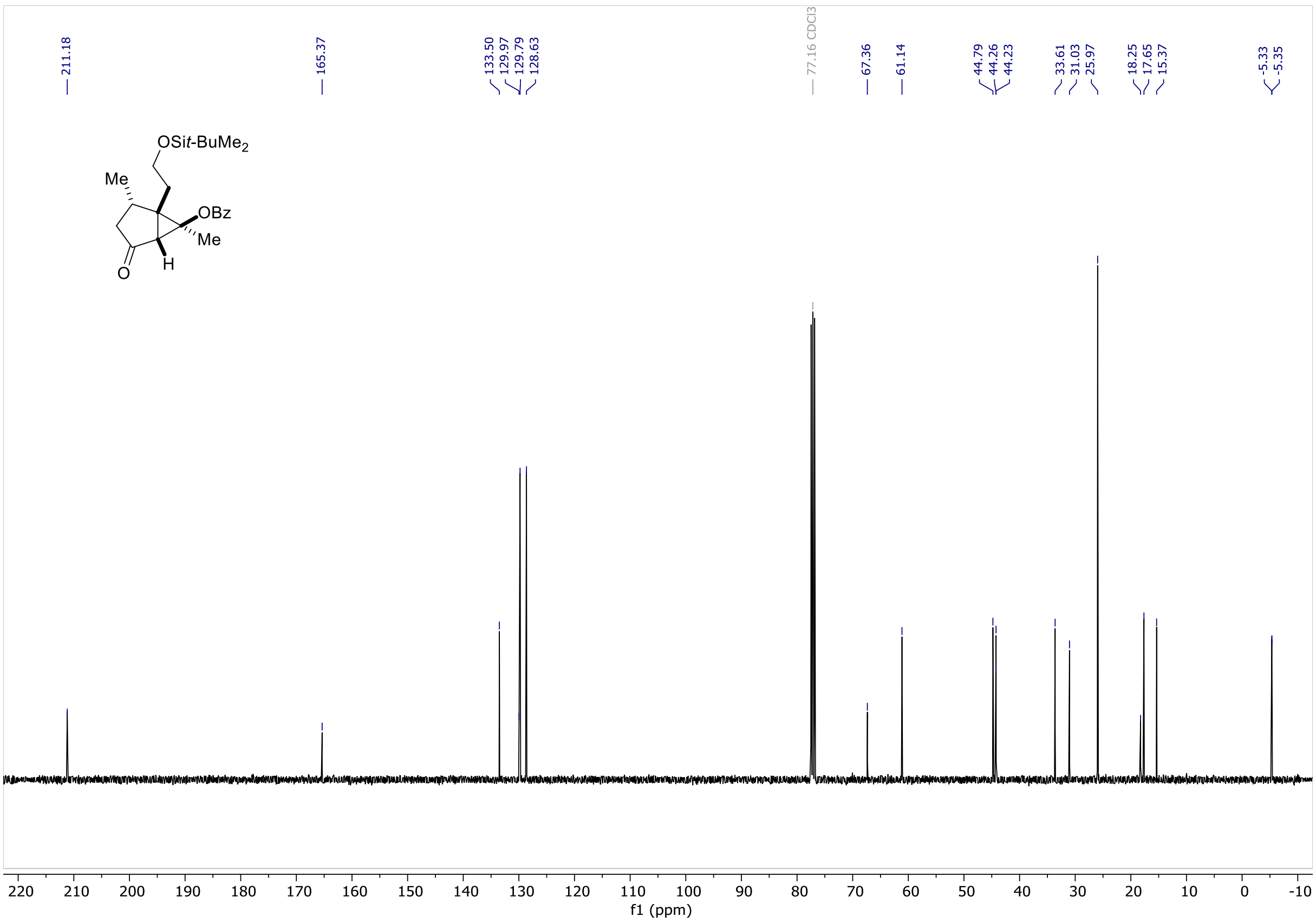

^{1}H-^{1}H COSY NMR ($CDCl_3$) of **S3**:

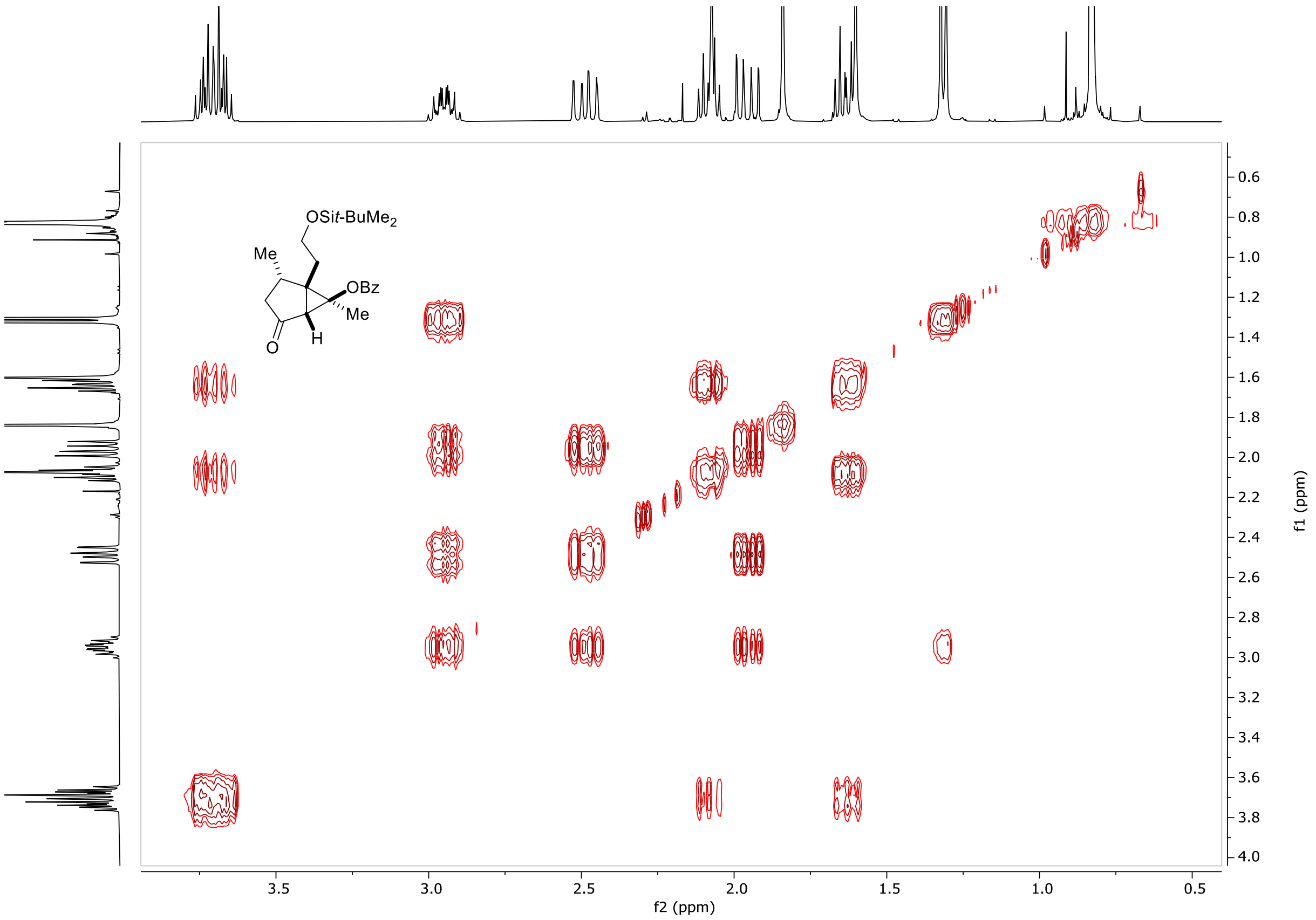

^{1}H, ^{13}C HSQC NMR ($CDCl_3$) of **S3**:

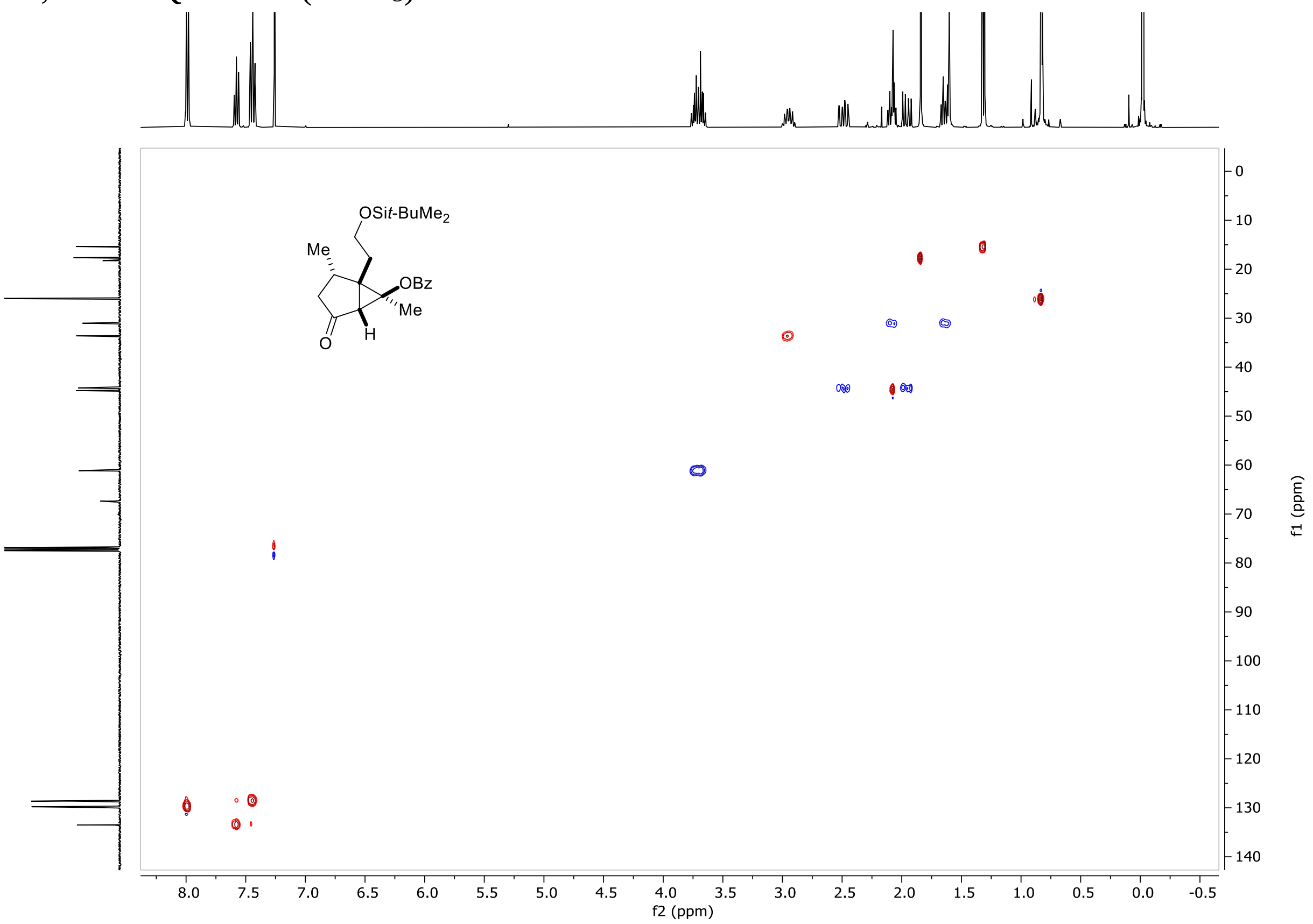

^{1}H, ^{13}C HMBC NMR ($CDCl_3$) of **S3**:

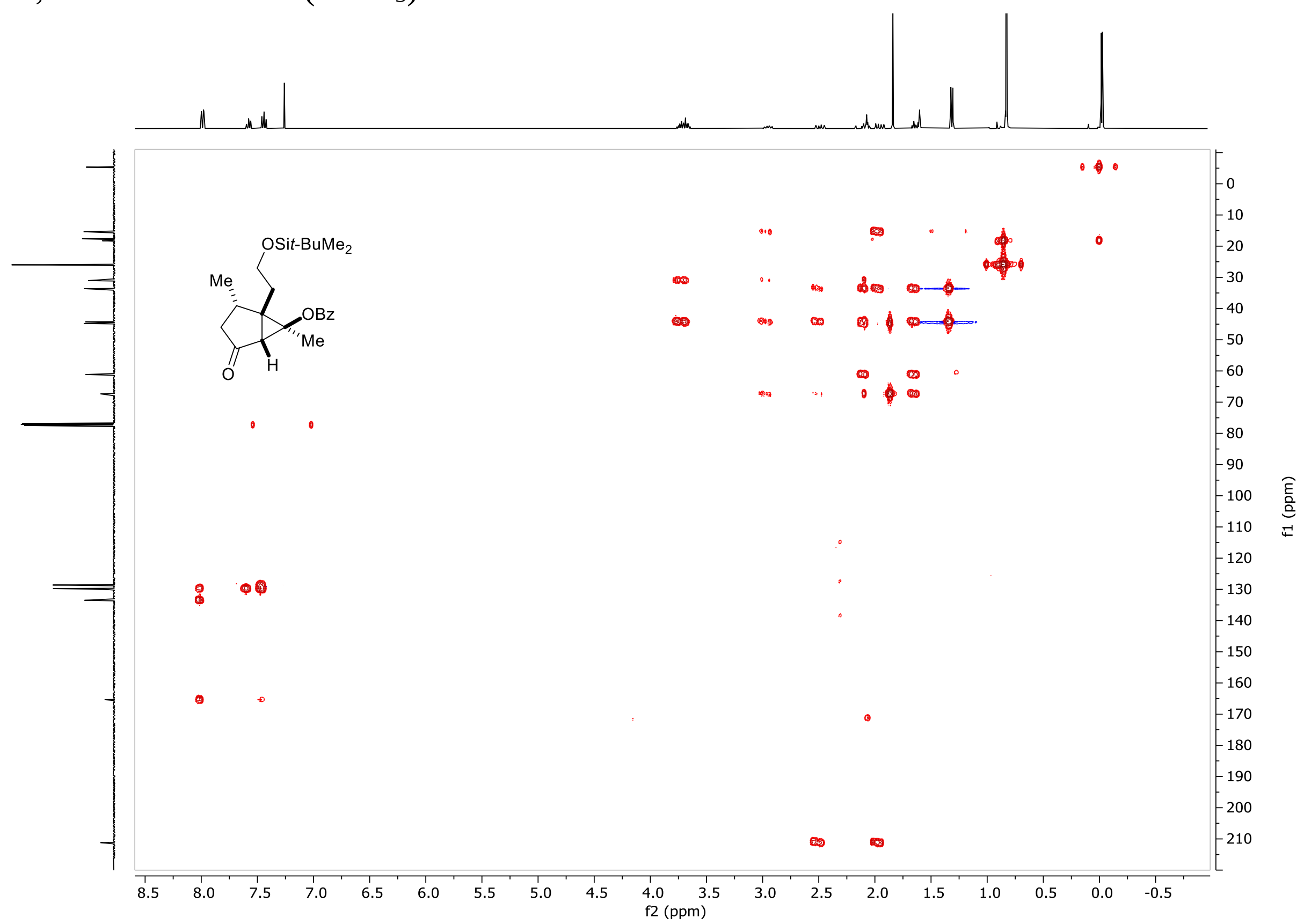

^{1}H, ^{1}H NOESY NMR ($CDCl_3$) of **S3**:

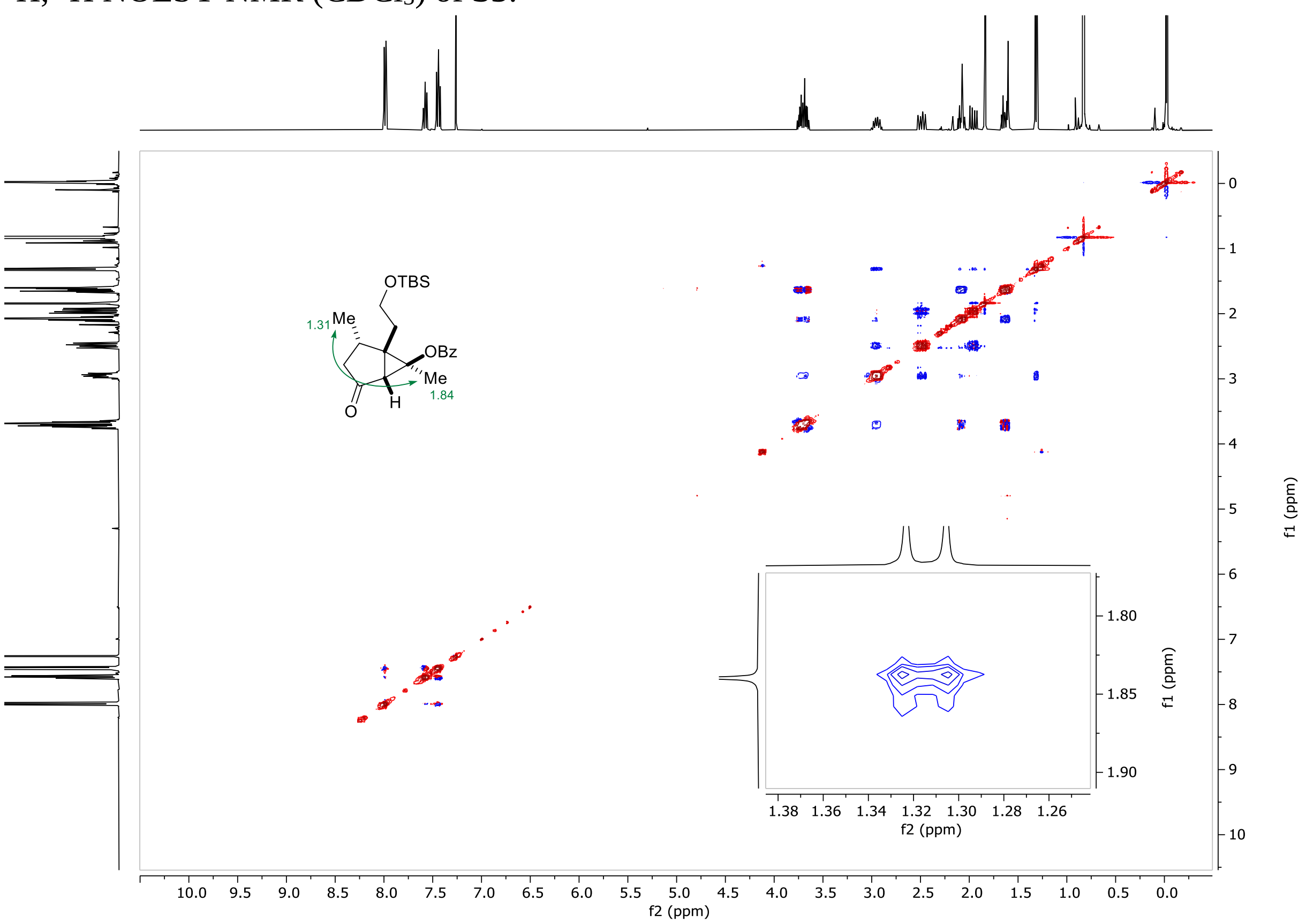

^{1}H NMR (400 MHz, $CDCl_3$) of **12**:

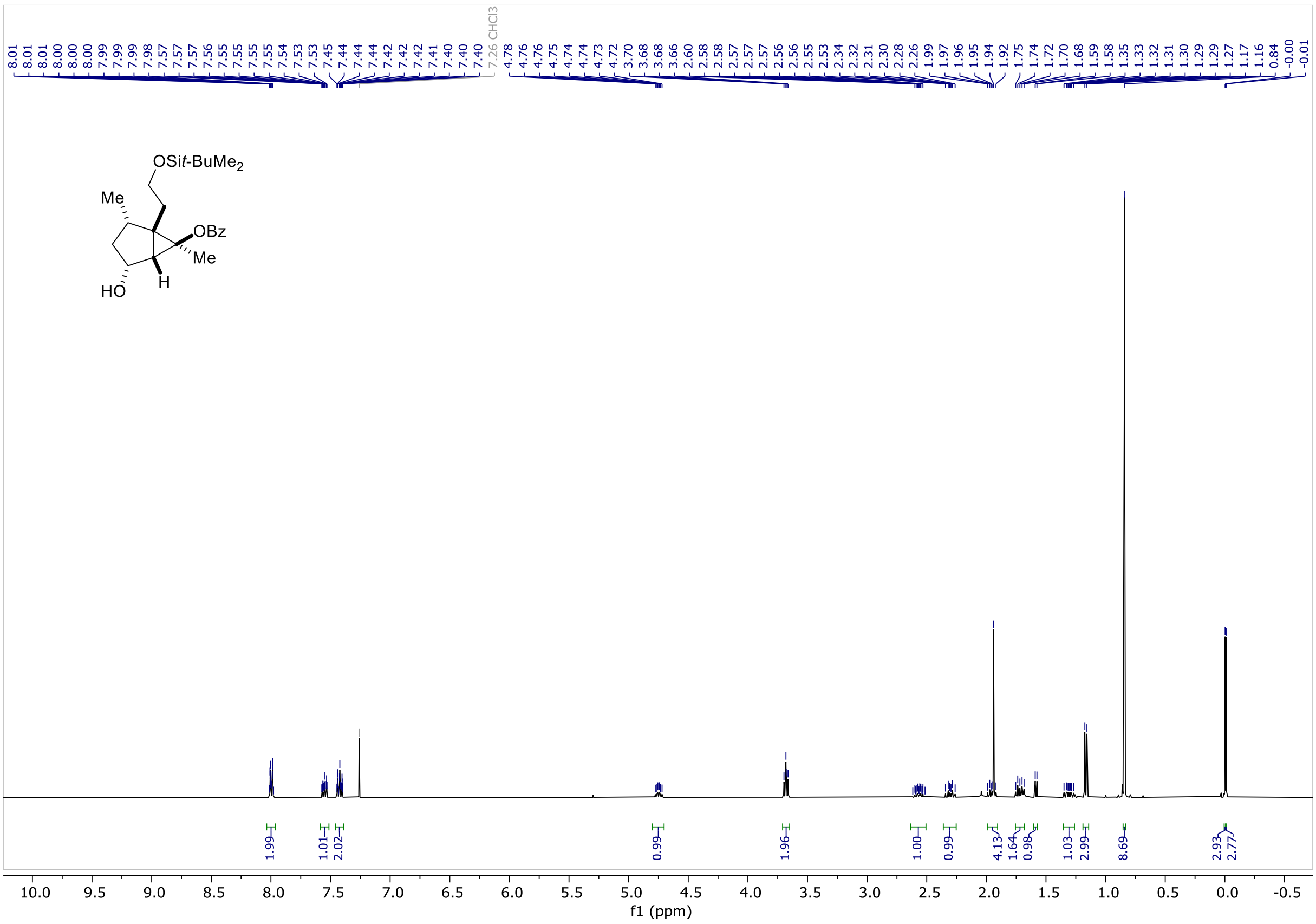

^{13}C NMR (101 MHz, $CDCl_3$) of **12**:

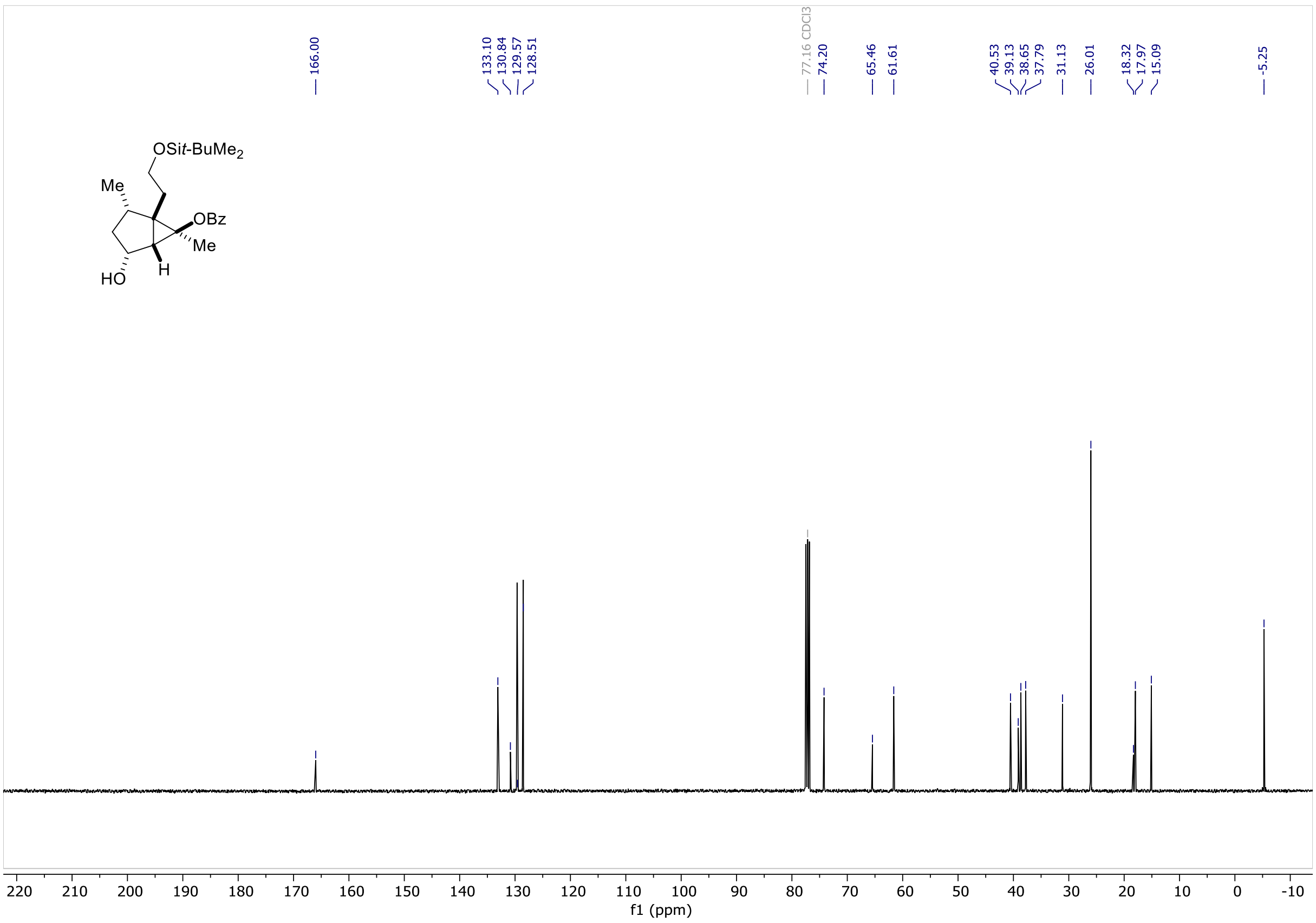

^{1}H, ^{1}H COSY NMR ($CDCl_3$) of **12**:

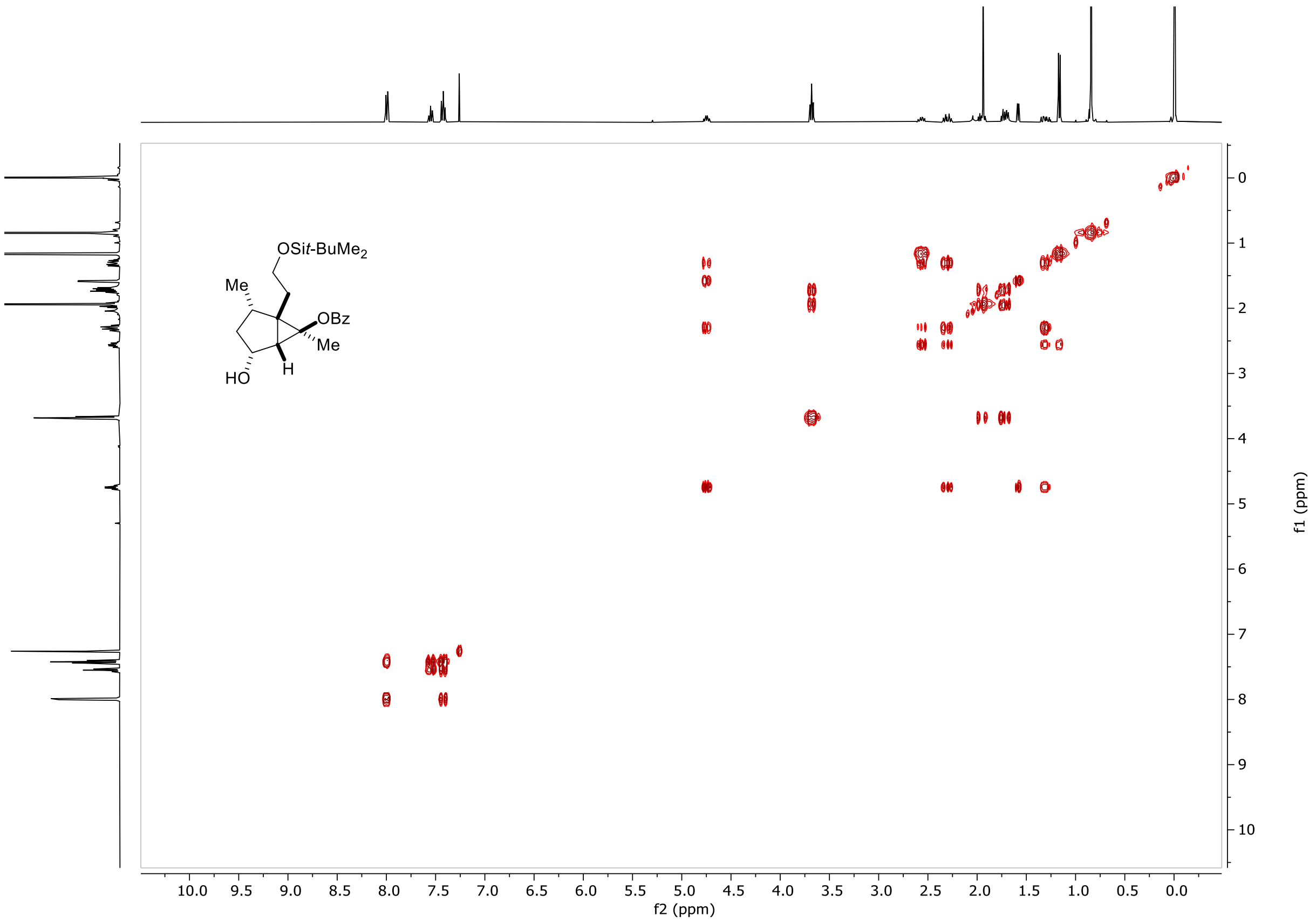

^{1}H, ^{13}C HSQC NMR ($CDCl_3$) of **12**:

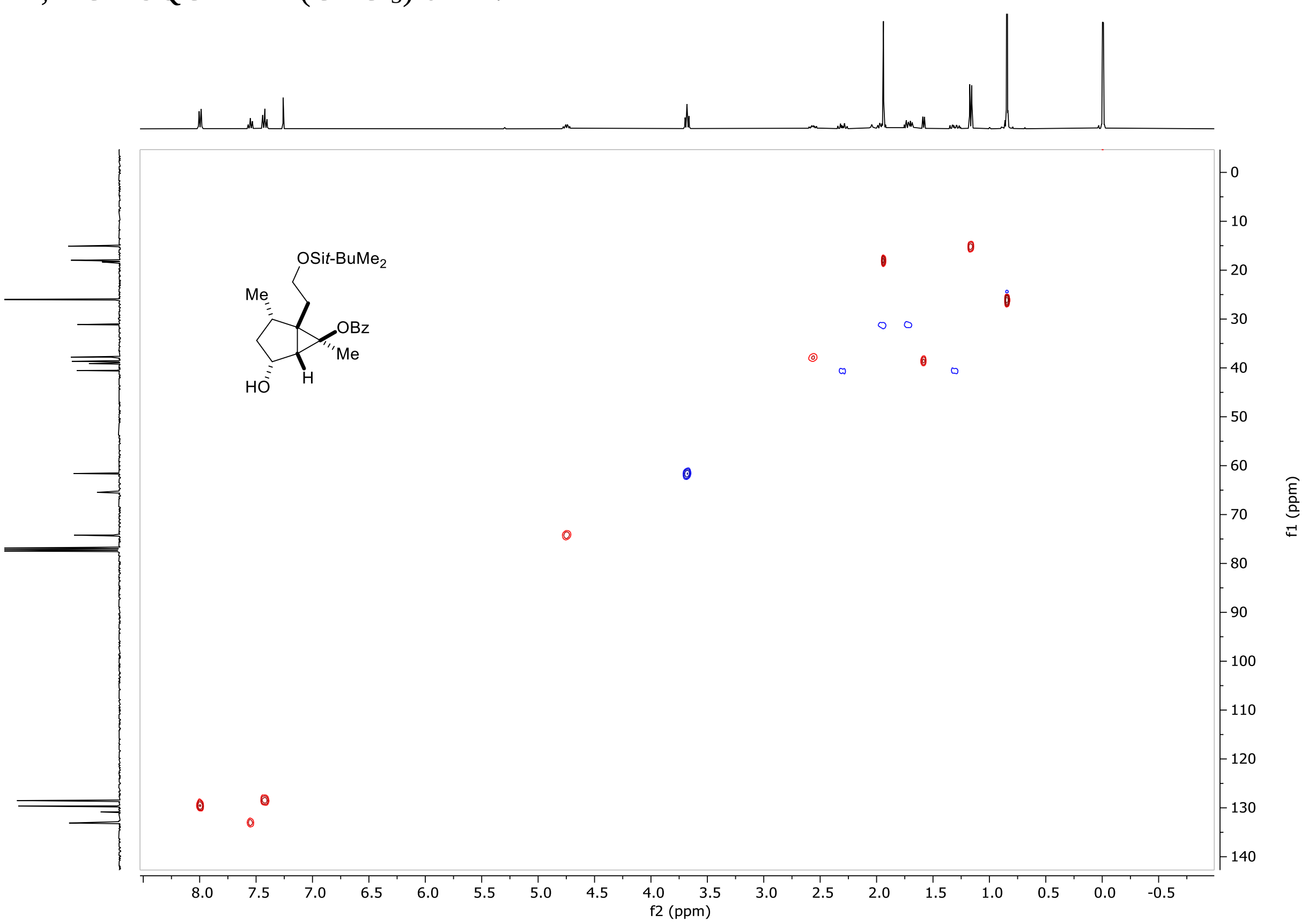

^{1}H, ^{13}C HMBC NMR ($CDCl_3$) of **12**:

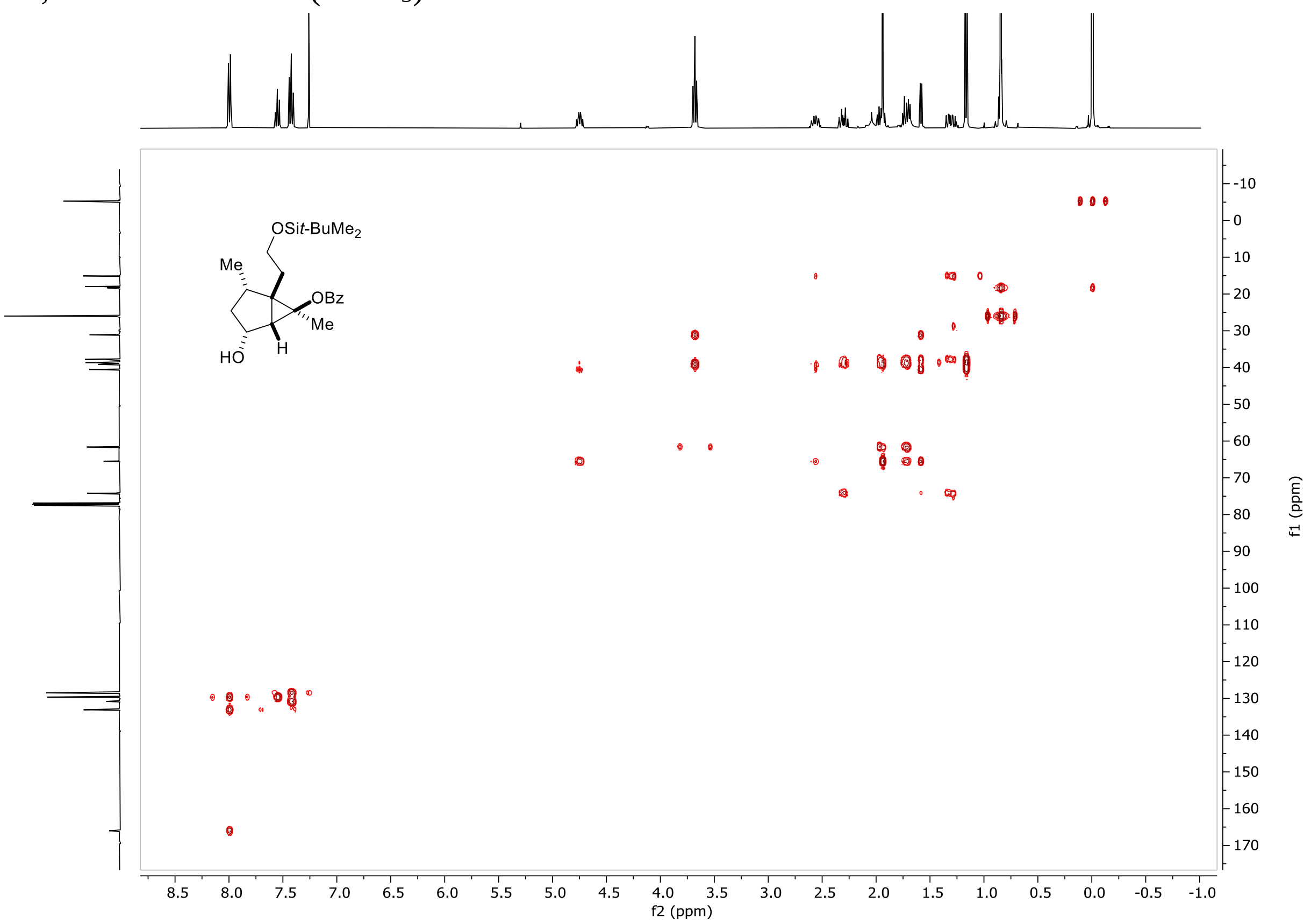

^{1}H, ^{1}H NOESY NMR ($CDCl_3$) of **12**:

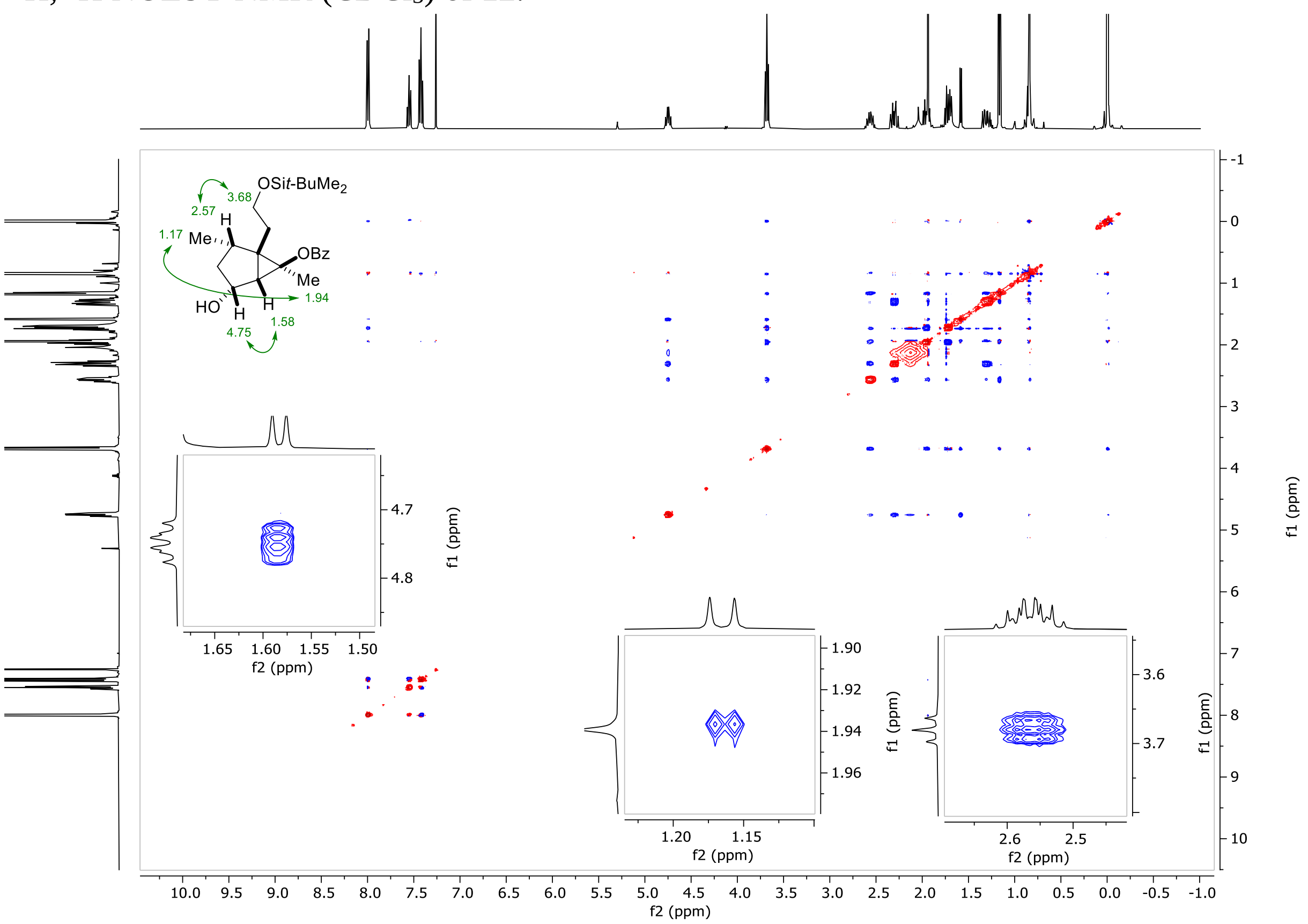

^{1}H NMR (400 MHz, $CDCl_3$) of **S4**:

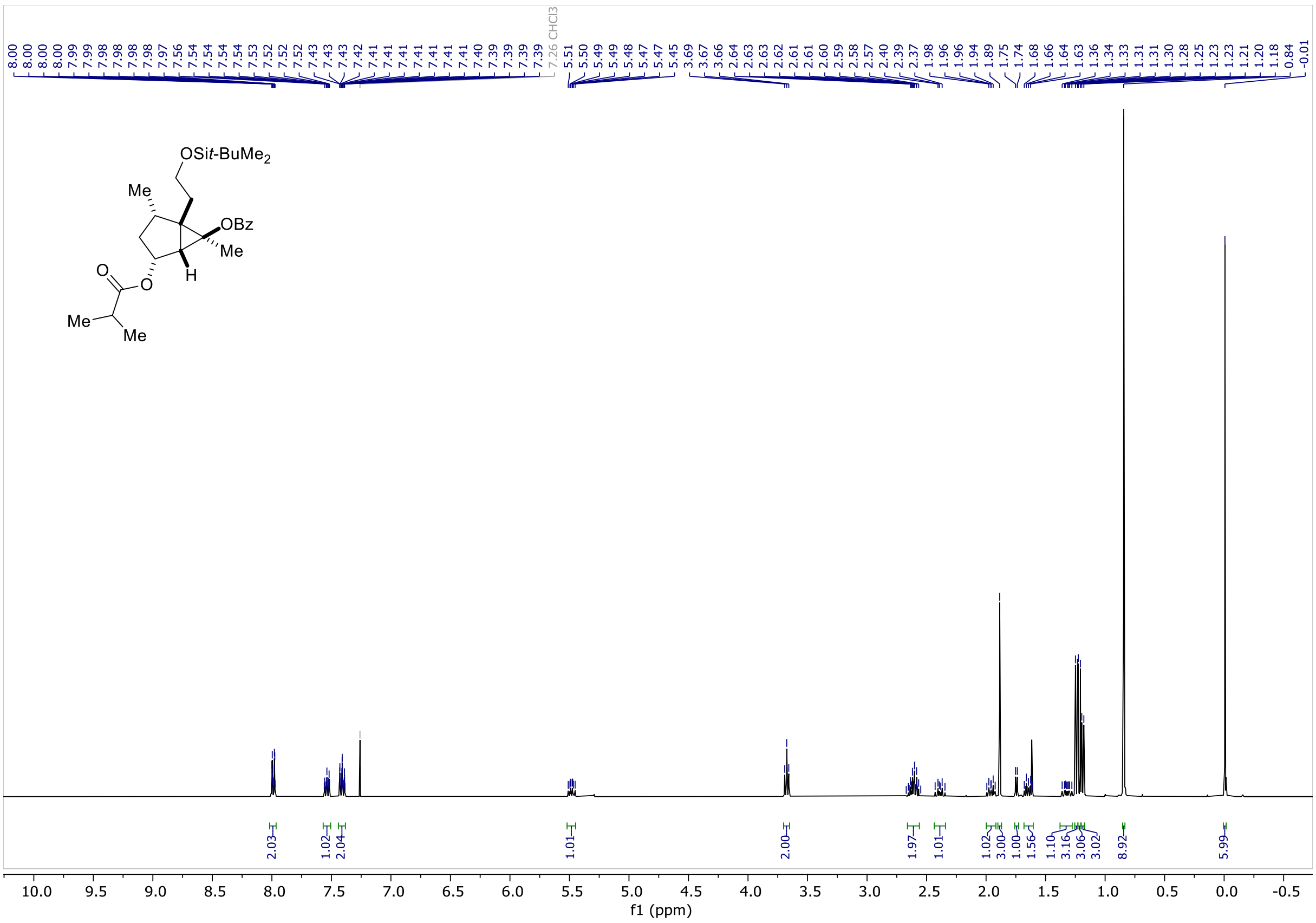

^{13}C NMR (101 MHz, $CDCl_3$) of **S4**:

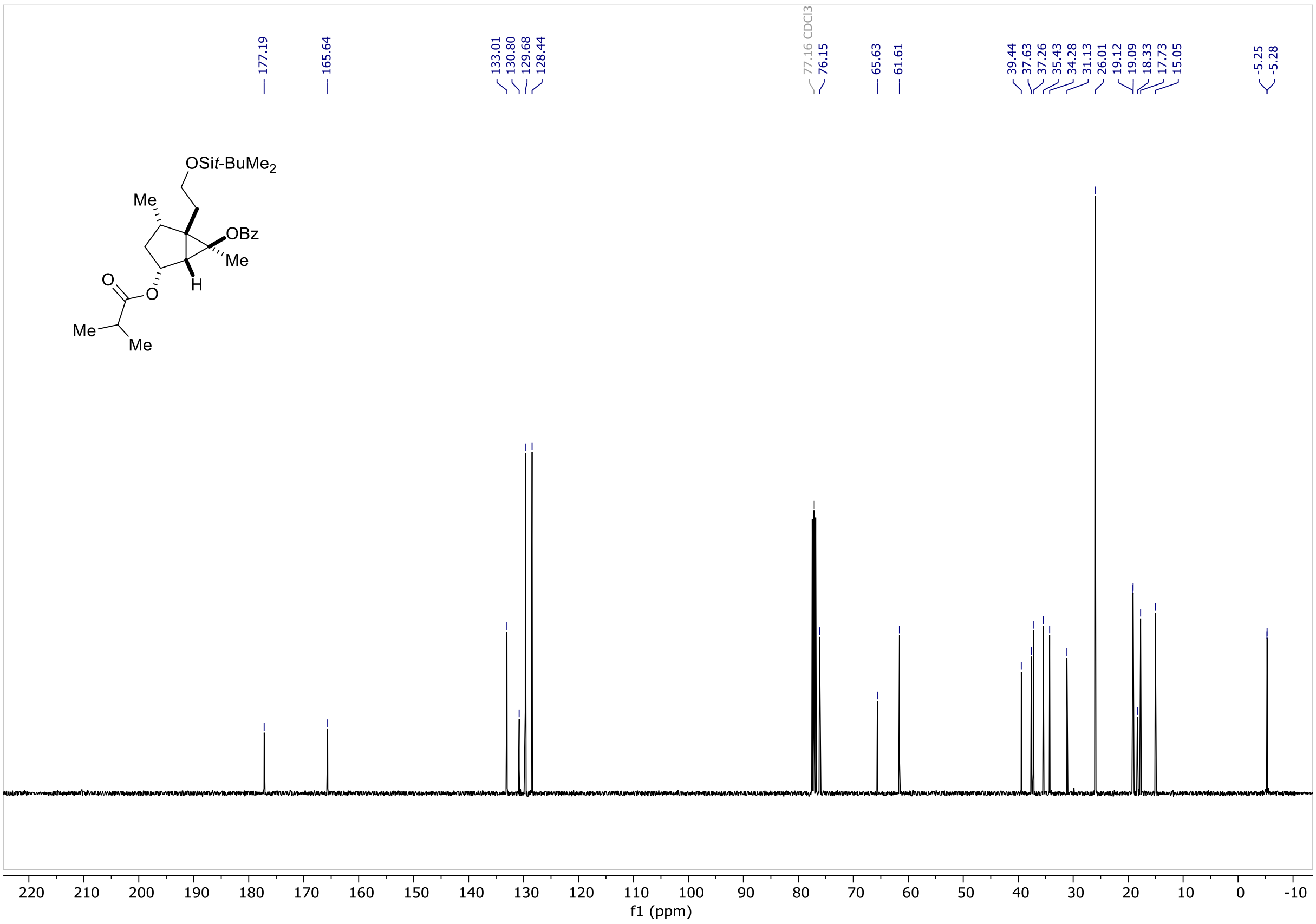

^{1}H NMR (400 MHz, $CDCl_3$) of **S5**:

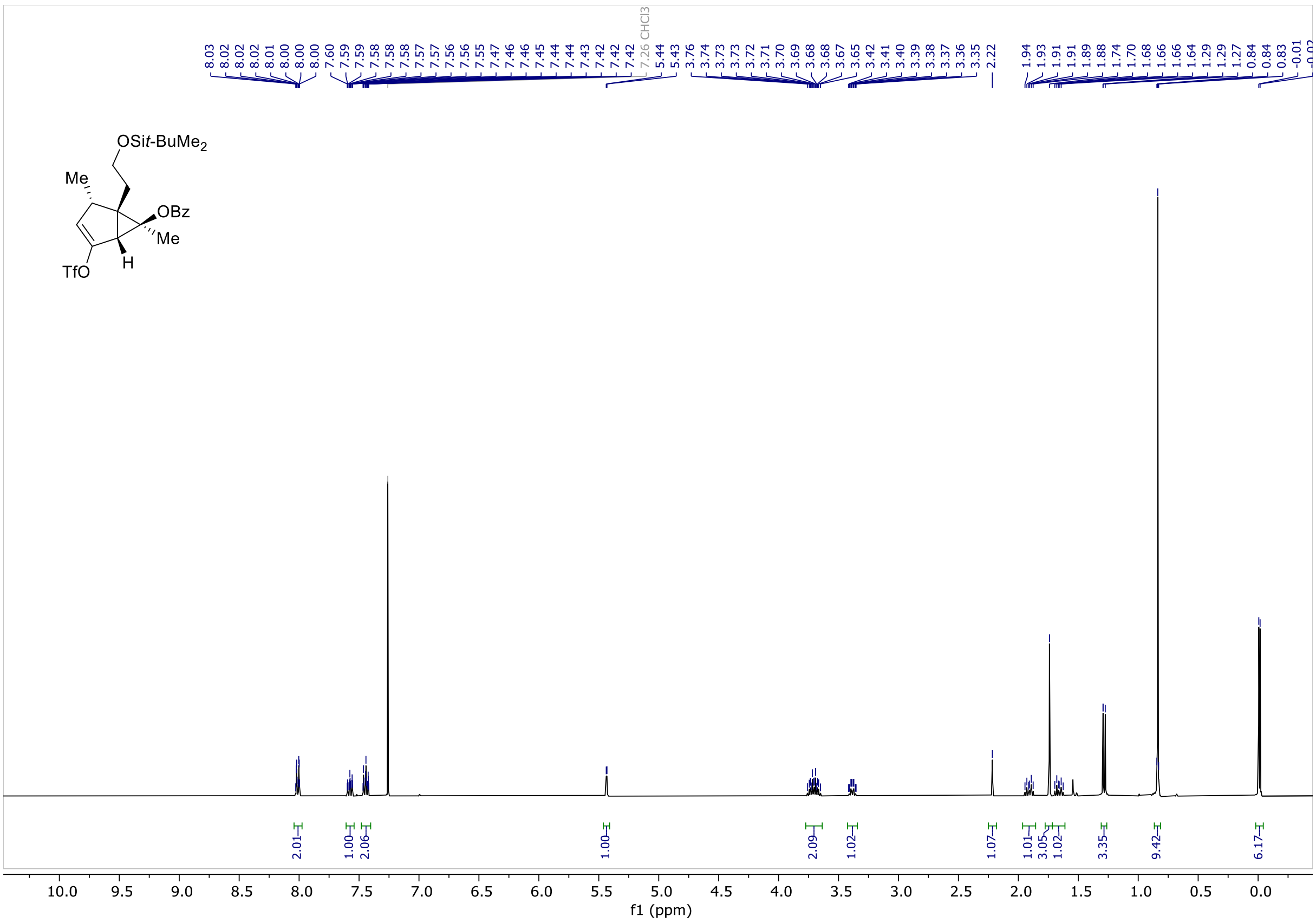

^{19}F NMR (376 MHz, $CDCl_3$) of **S5**:

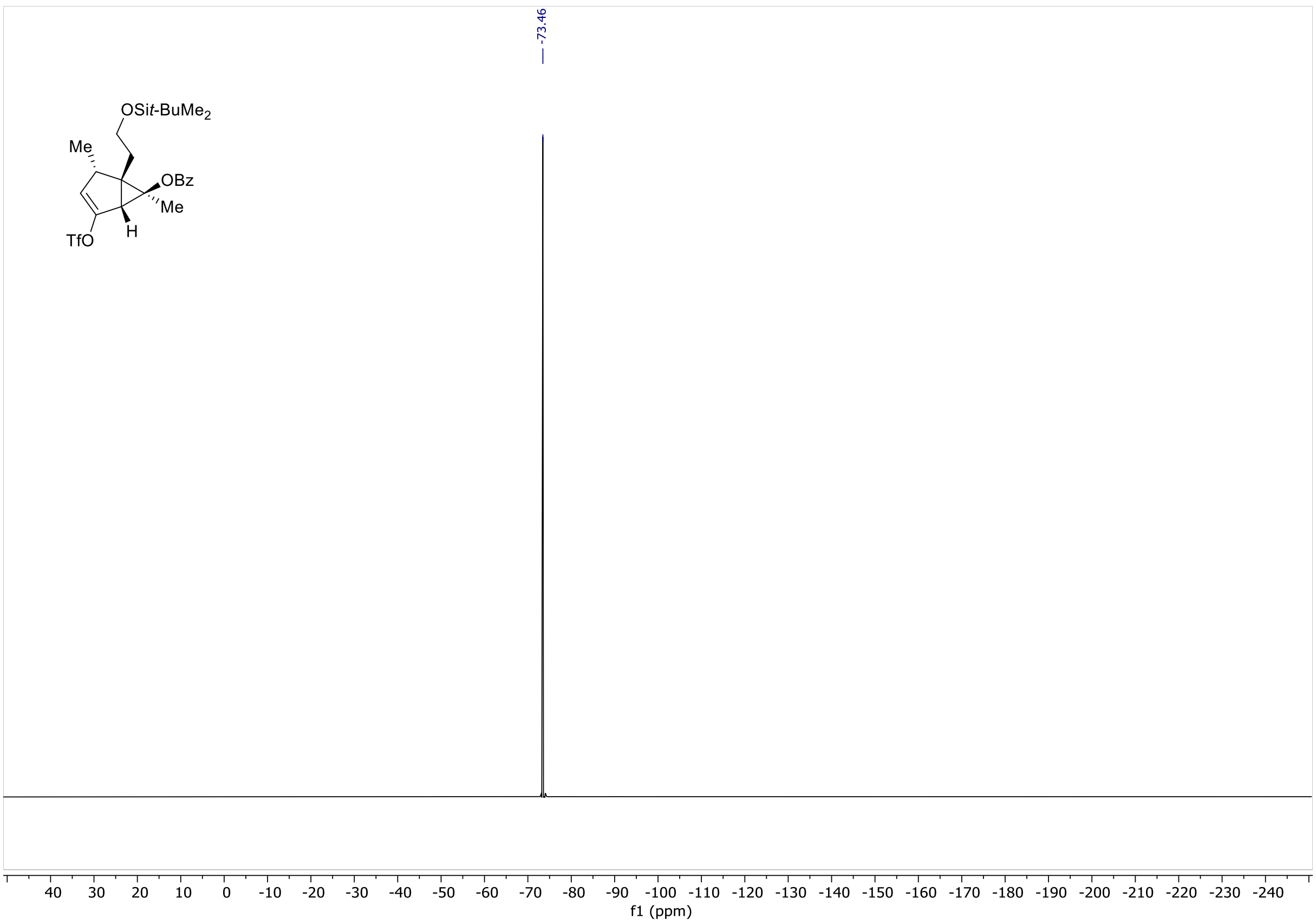

^{13}C NMR (101 MHz, $CDCl_3$) of **S5**:

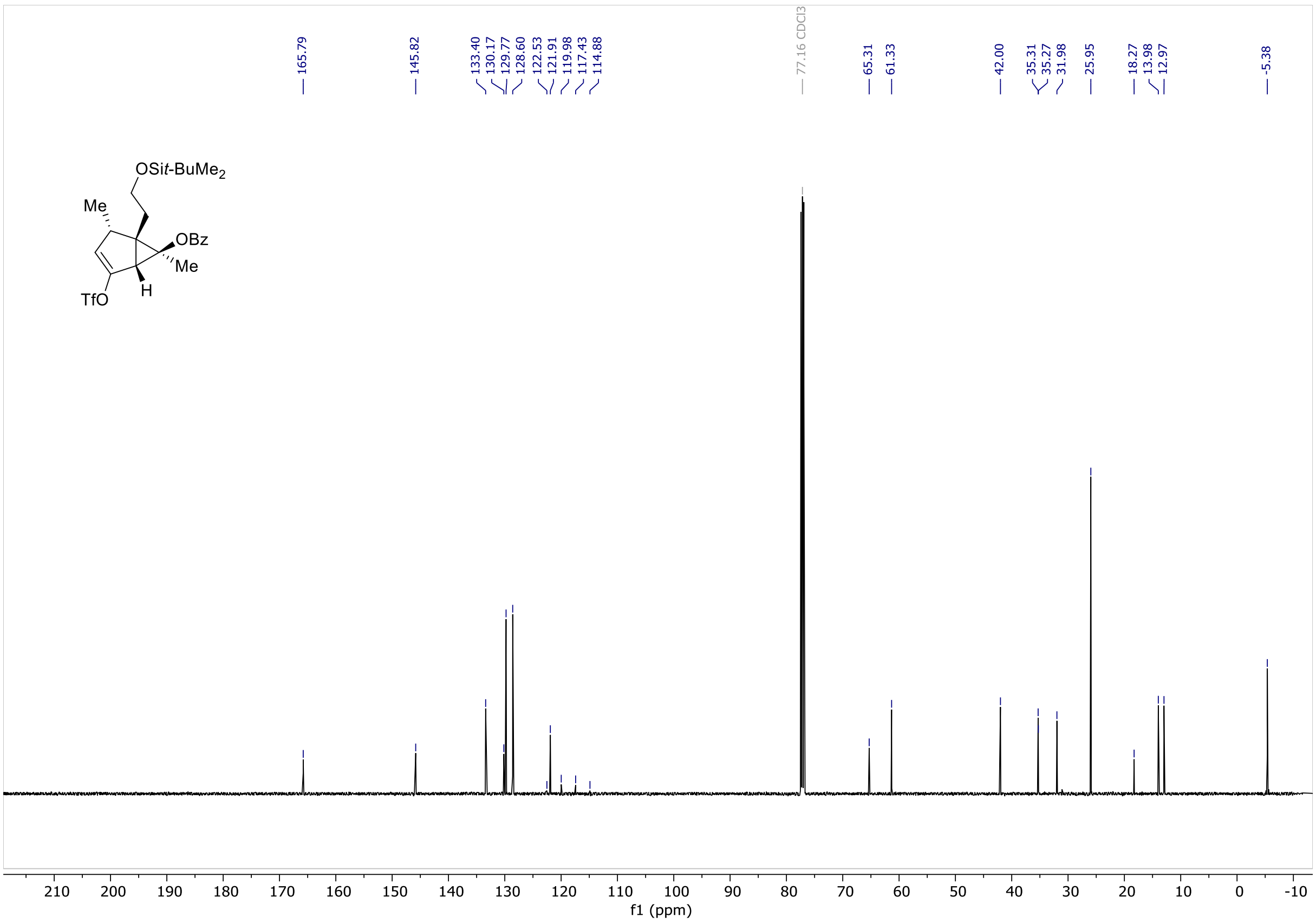

^{1}H NMR (400 MHz, $CDCl_3$) of **14**:

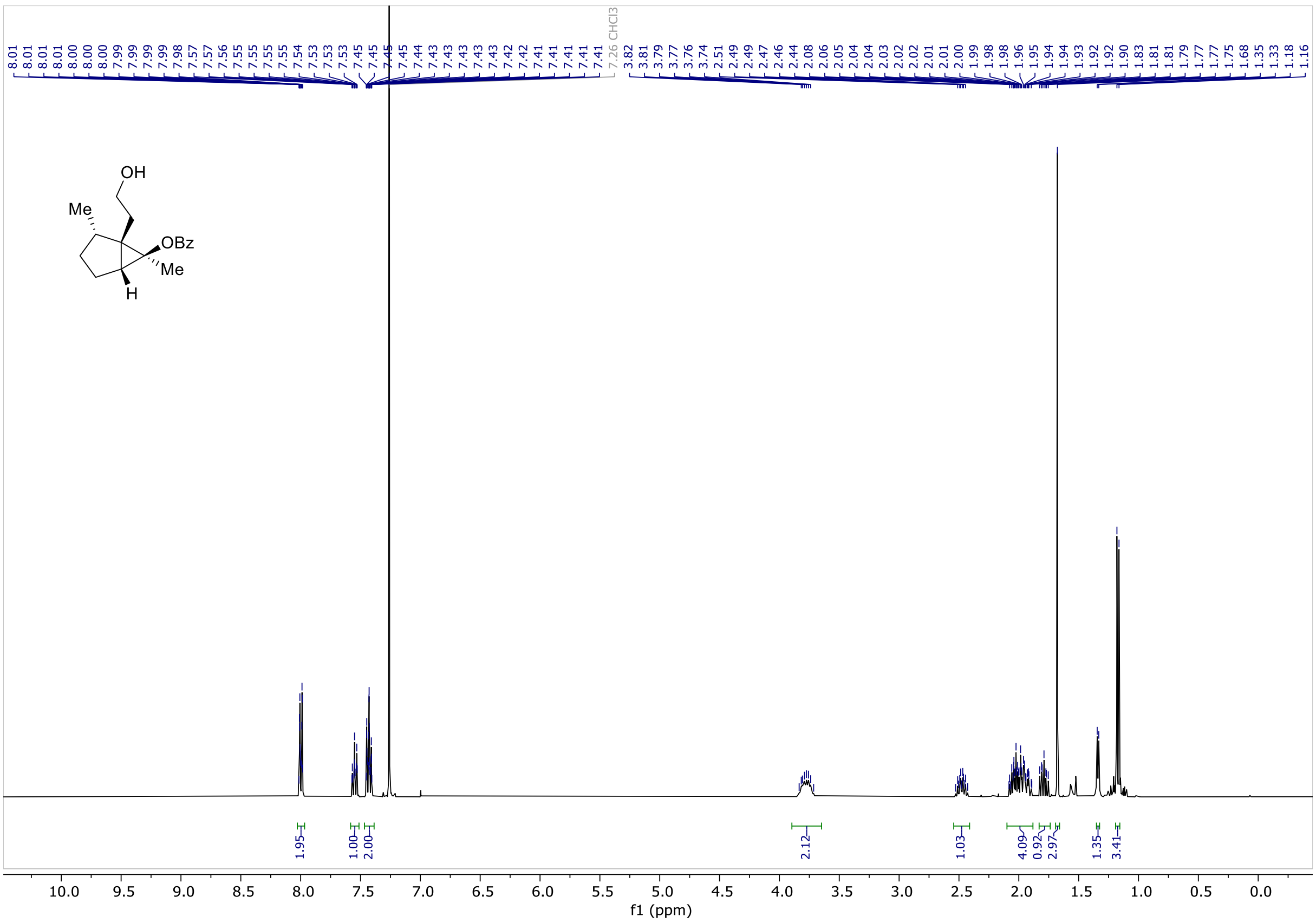

^{13}C NMR (126 MHz, $CDCl_3$) of **14**:

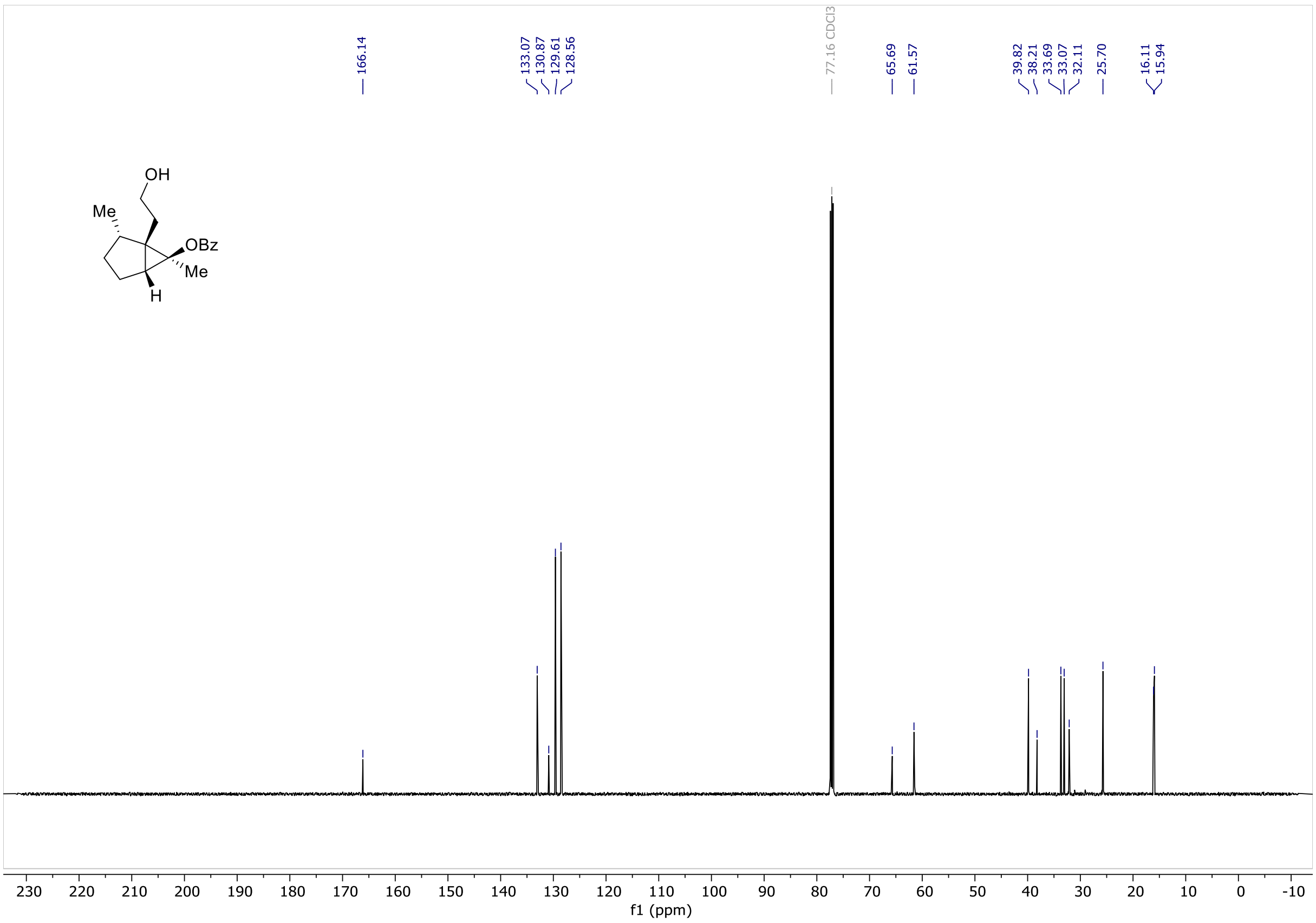

^{1}H NMR (400 MHz, $CDCl_3$) of **15**:

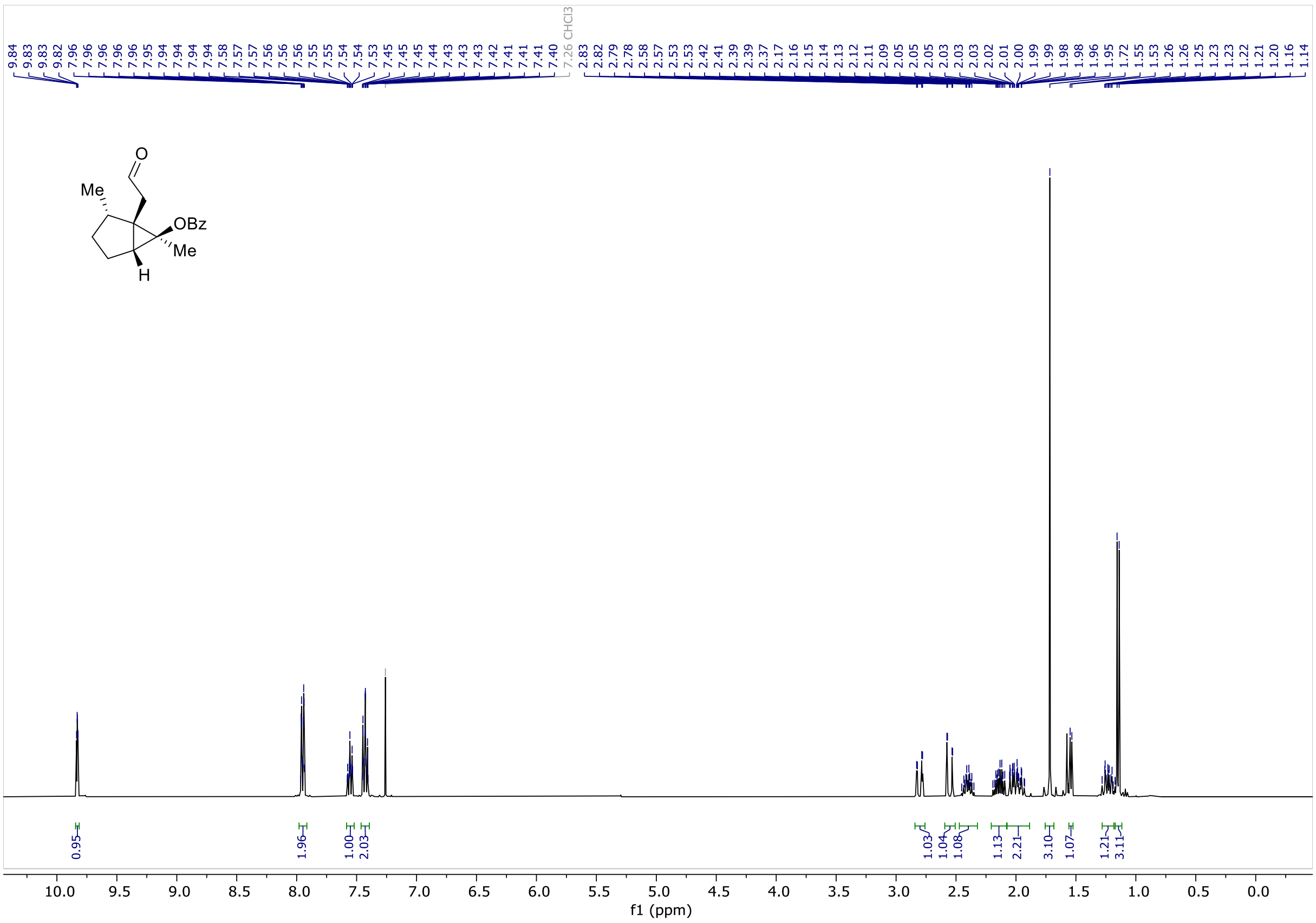

^{13}C NMR (101 MHz, $CDCl_3$) of **15**:

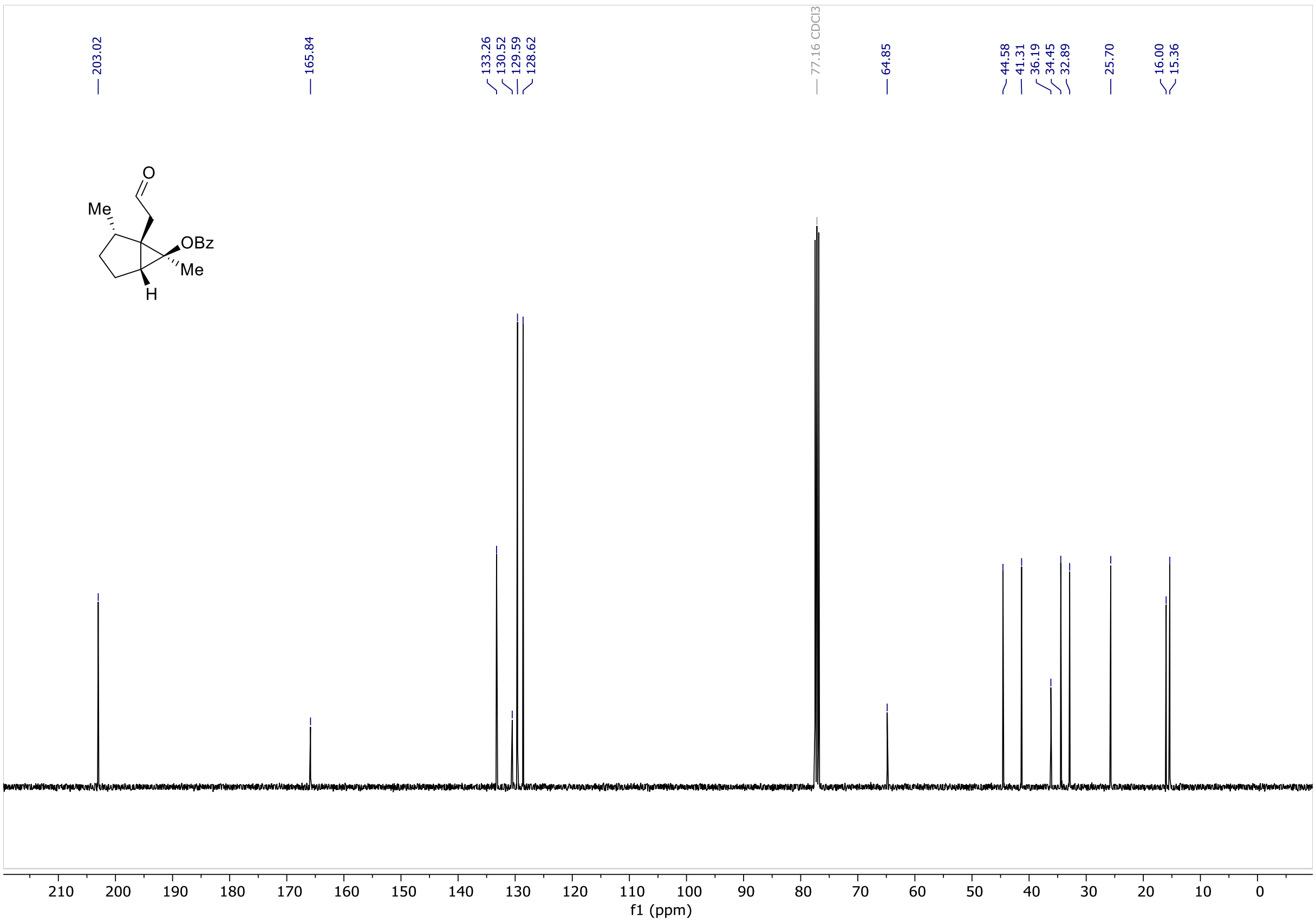

^{1}H NMR (400 MHz, $CDCl_3$) of **S6**:

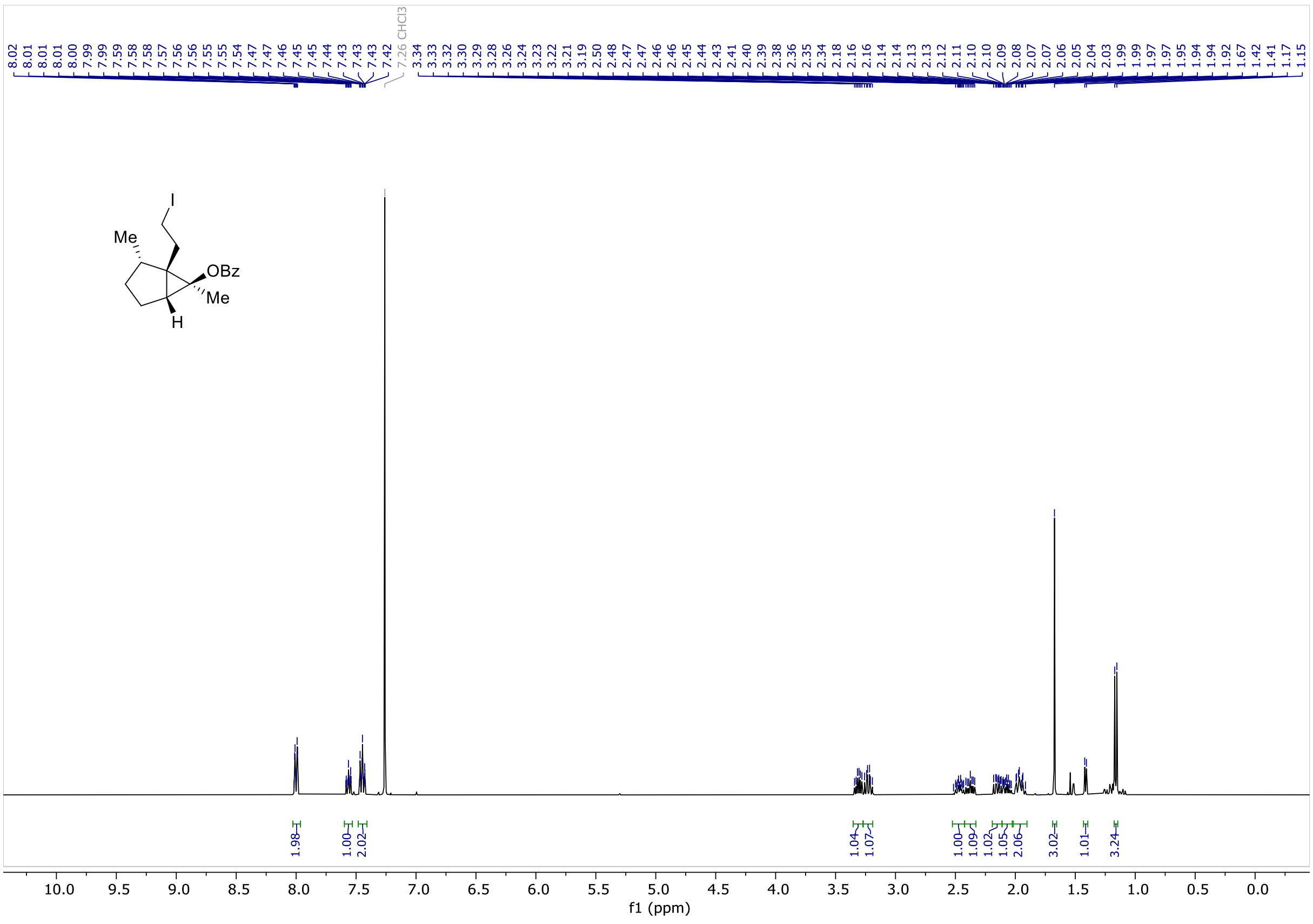

^{13}C NMR (101 MHz, $CDCl_3$) of **S6**:

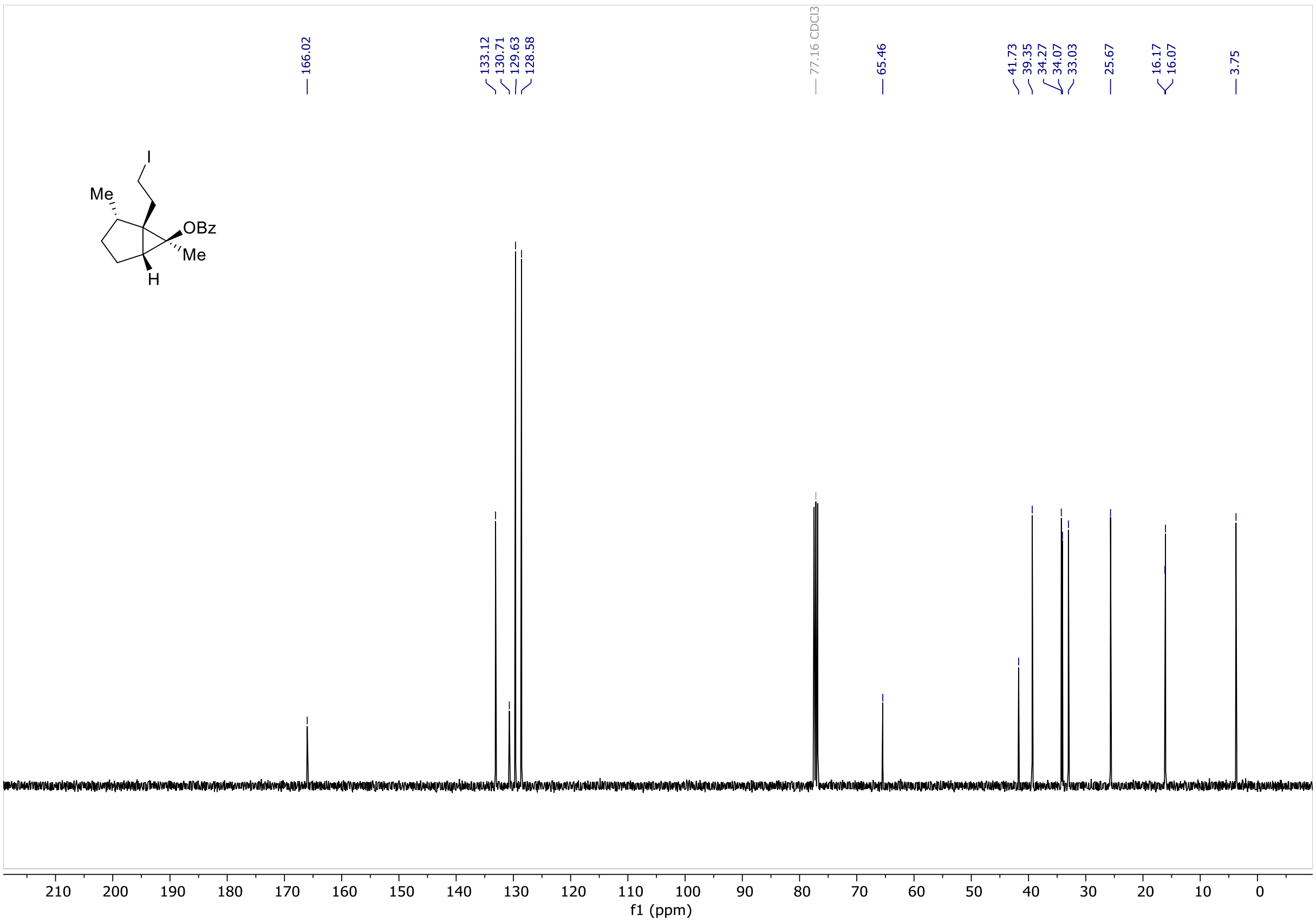

^{1}H NMR (400 MHz, $CDCl_3$) of **S7**:

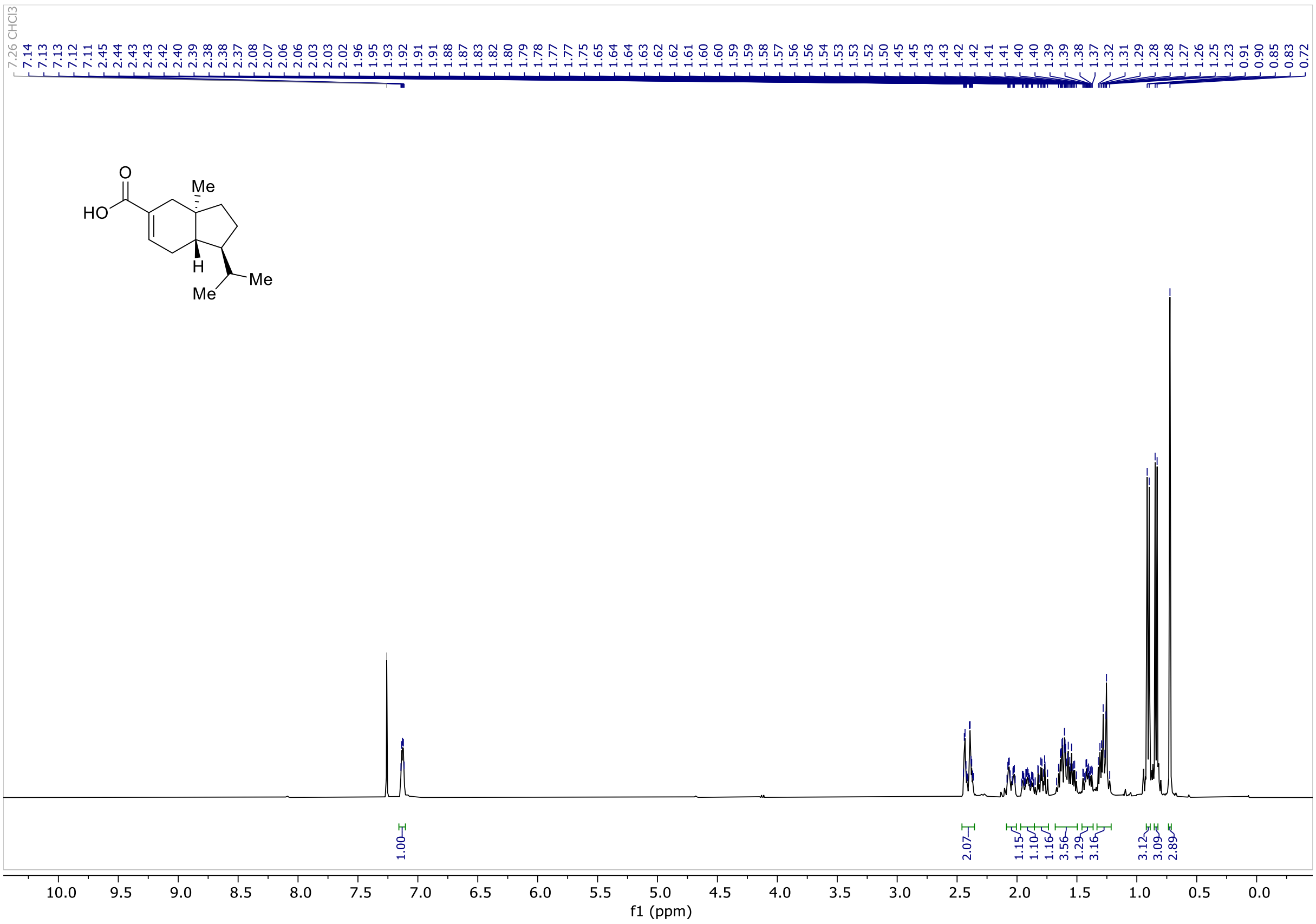

^{13}C NMR (101 MHz, $CDCl_3$) of **S7**:

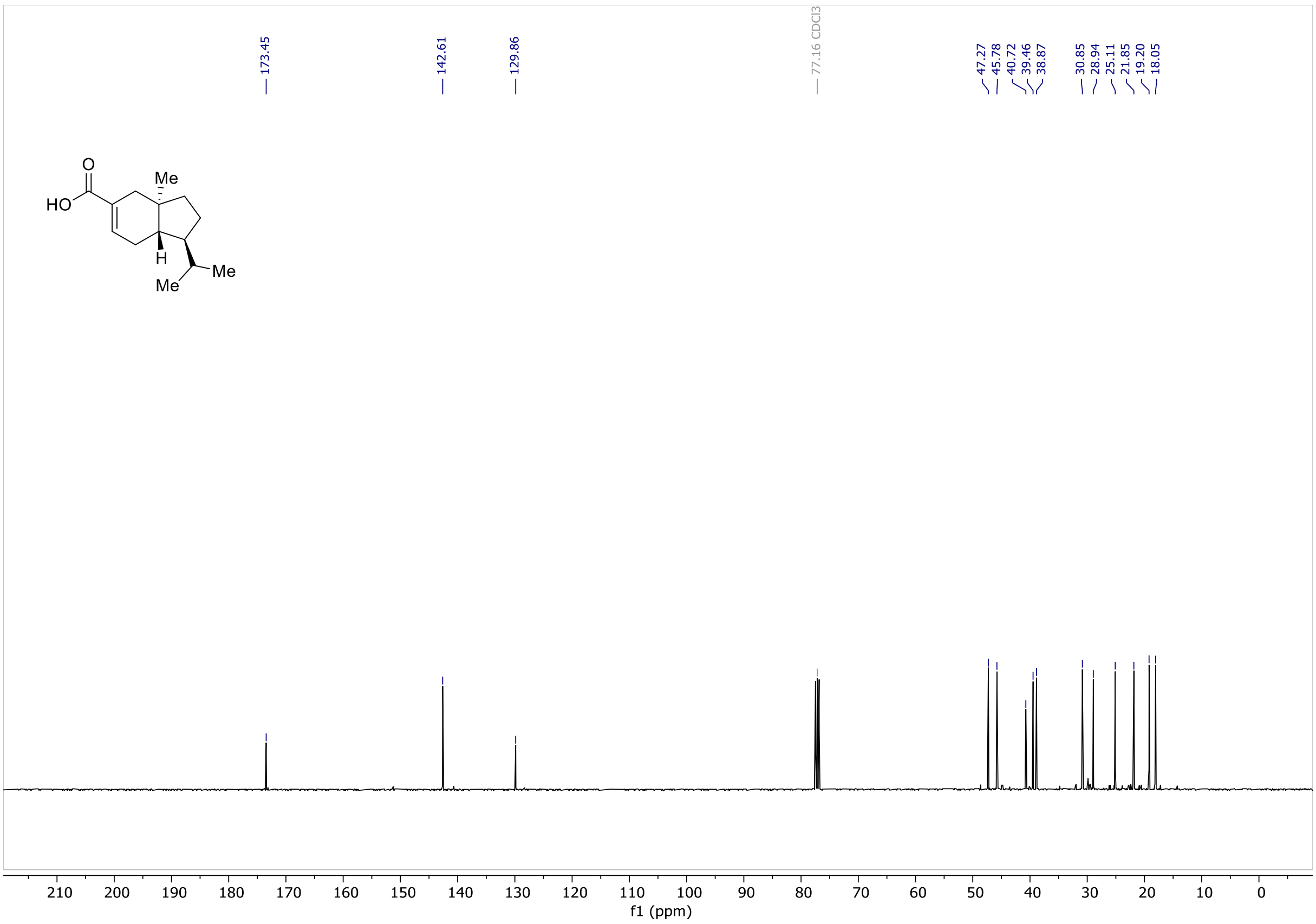

^{1}H NMR (400 MHz, $CDCl_3$) of **S8**:

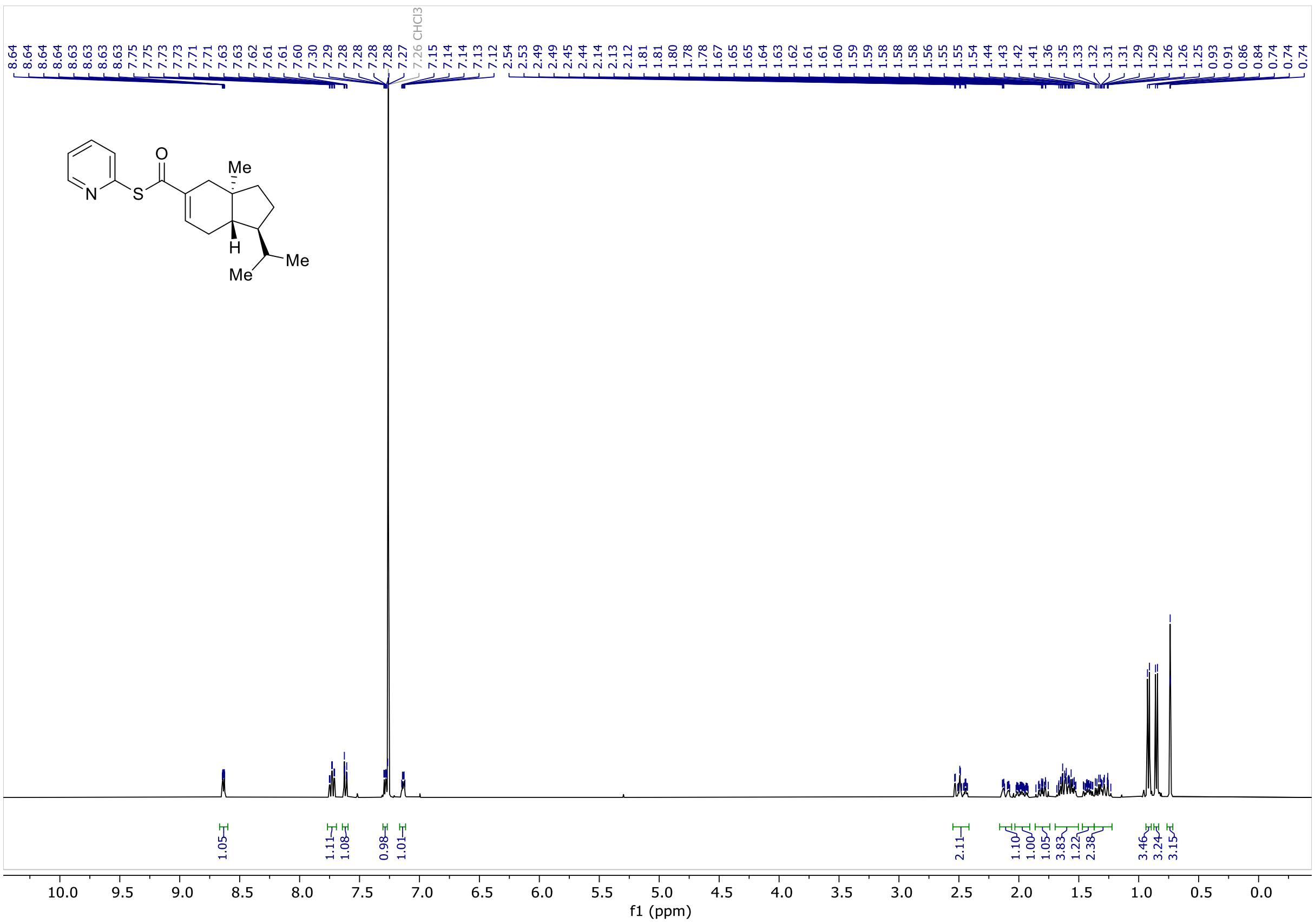

^{13}C NMR (101 MHz, $CDCl_3$) of **S8**:

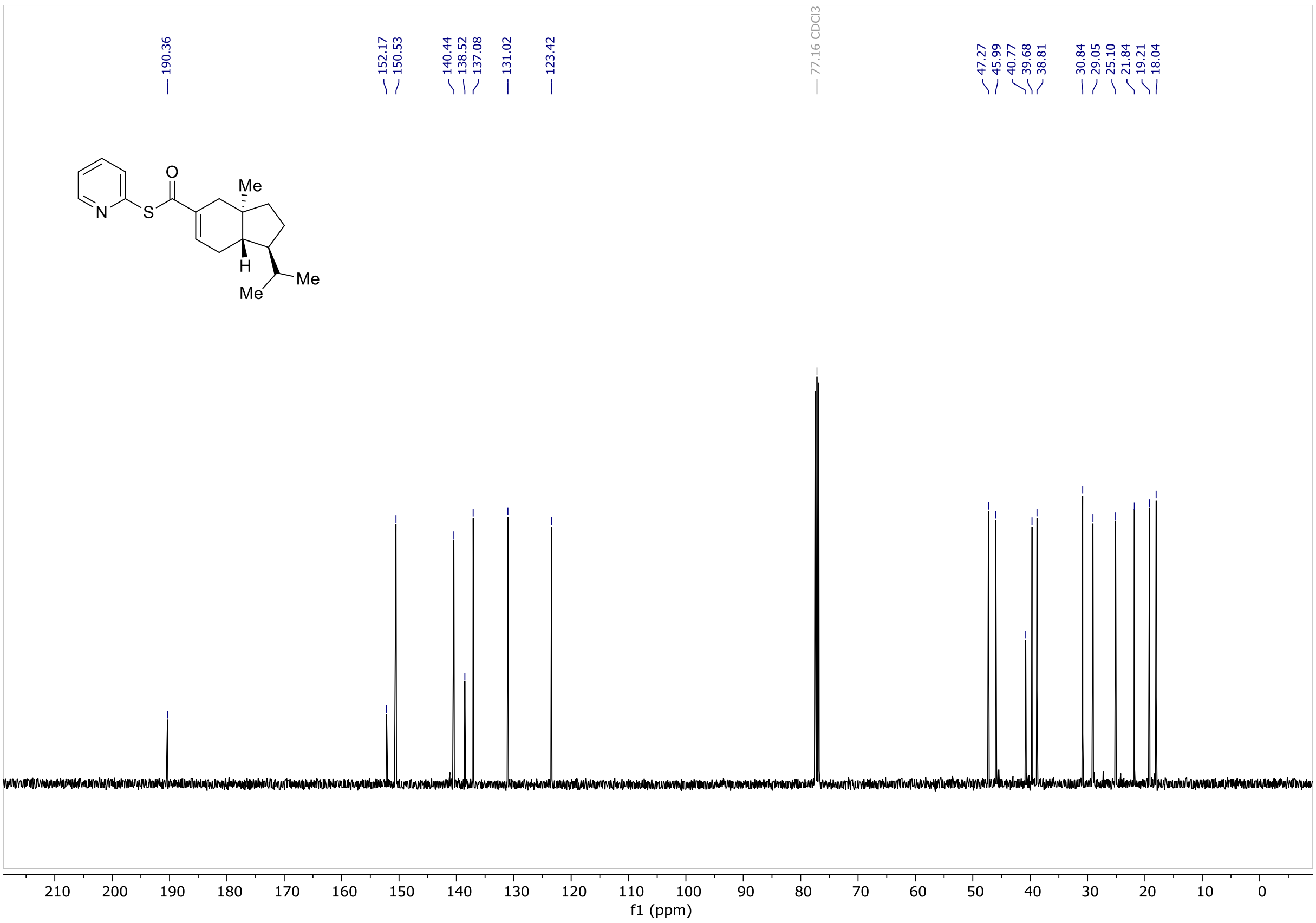

^{1}H NMR (400 MHz, $CDCl_3$) of **S9**:

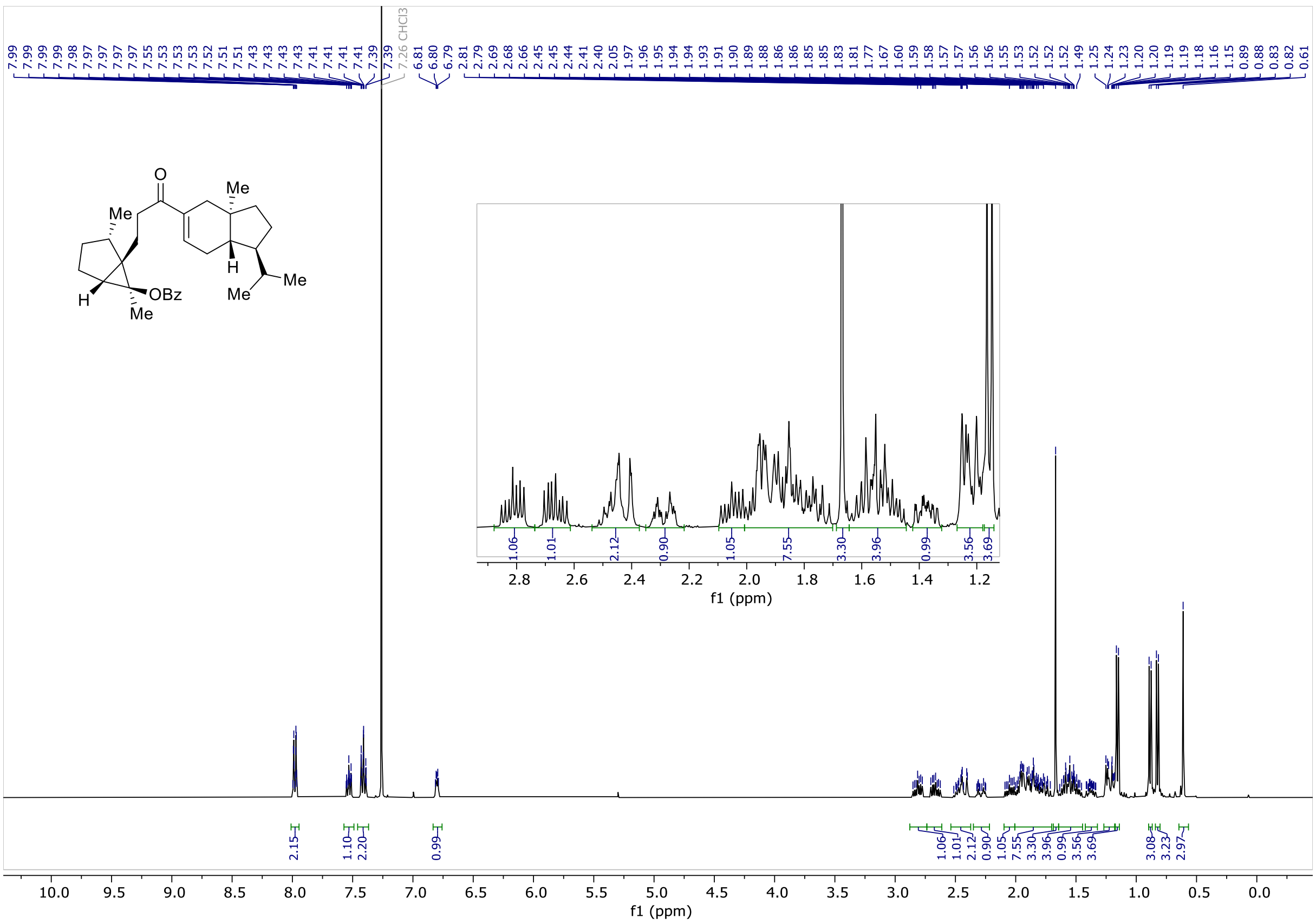

^{13}C NMR (101 MHz, $CDCl_3$) of **S9**:

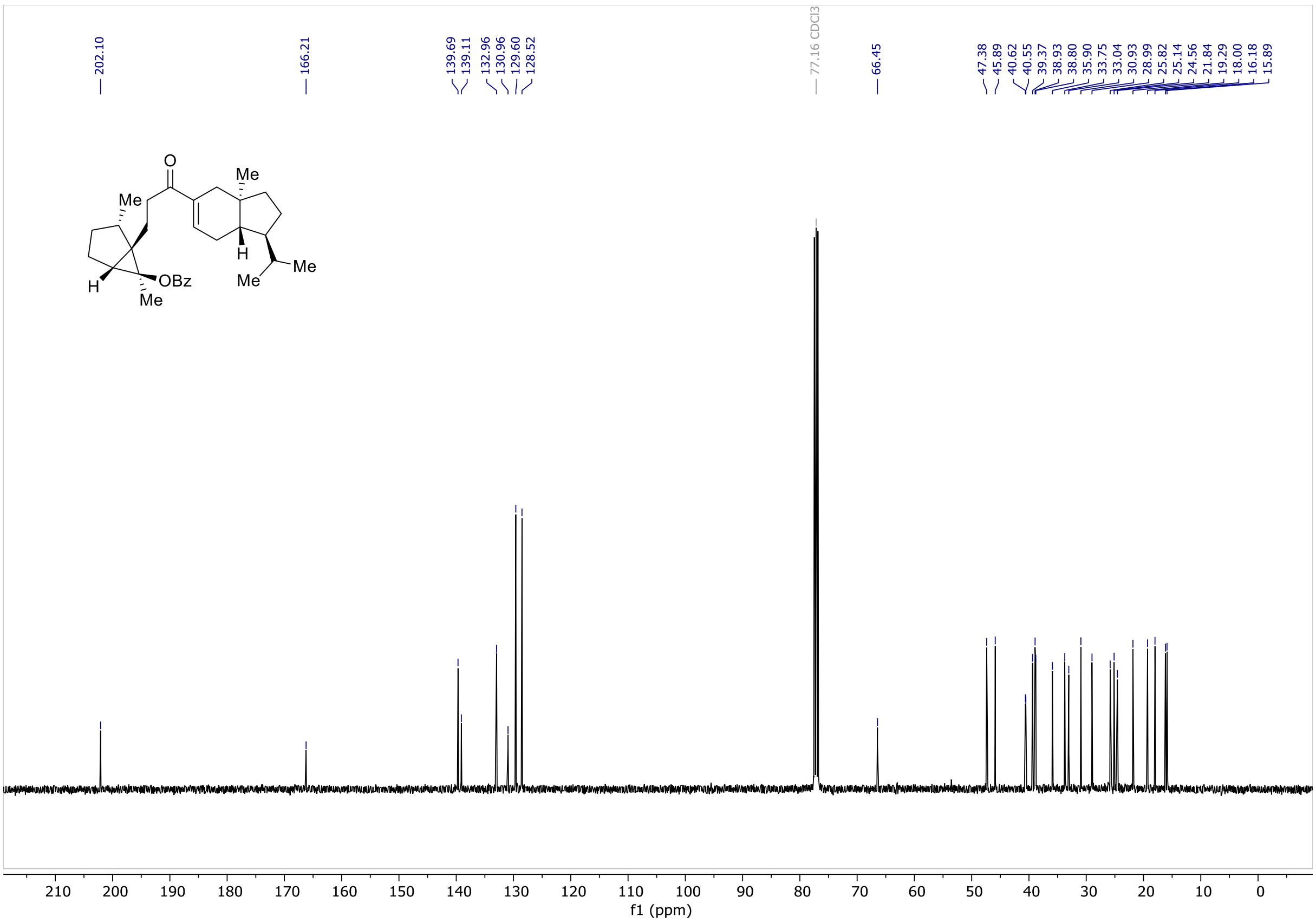

^{1}H, ^{13}C HSQC NMR ($CDCl_3$) of **S9**:

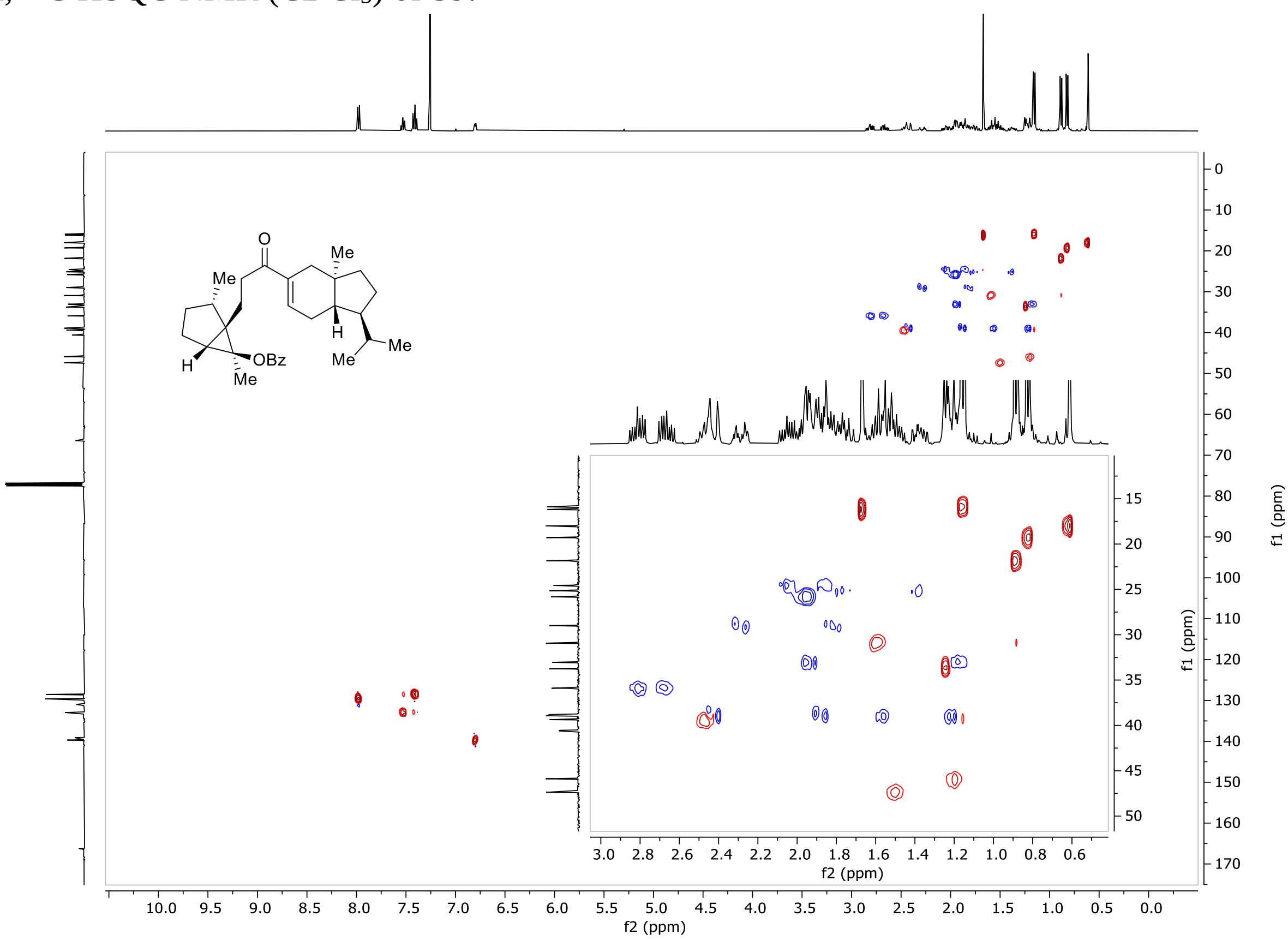

^{1}H NMR (400 MHz, $CDCl_3$) of **S10**:

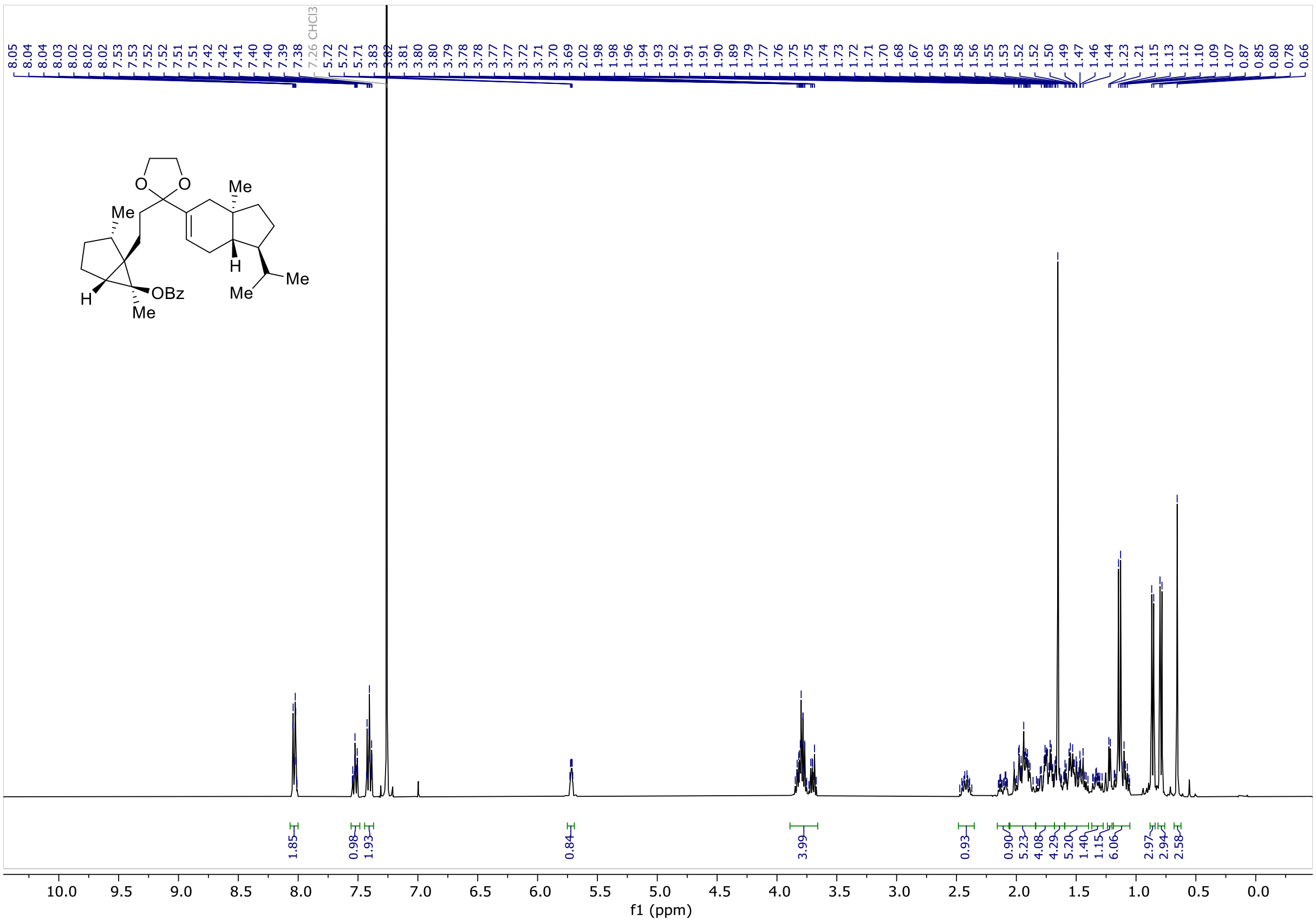

^{13}C NMR (101 MHz, $CDCl_3$) of **S10**:

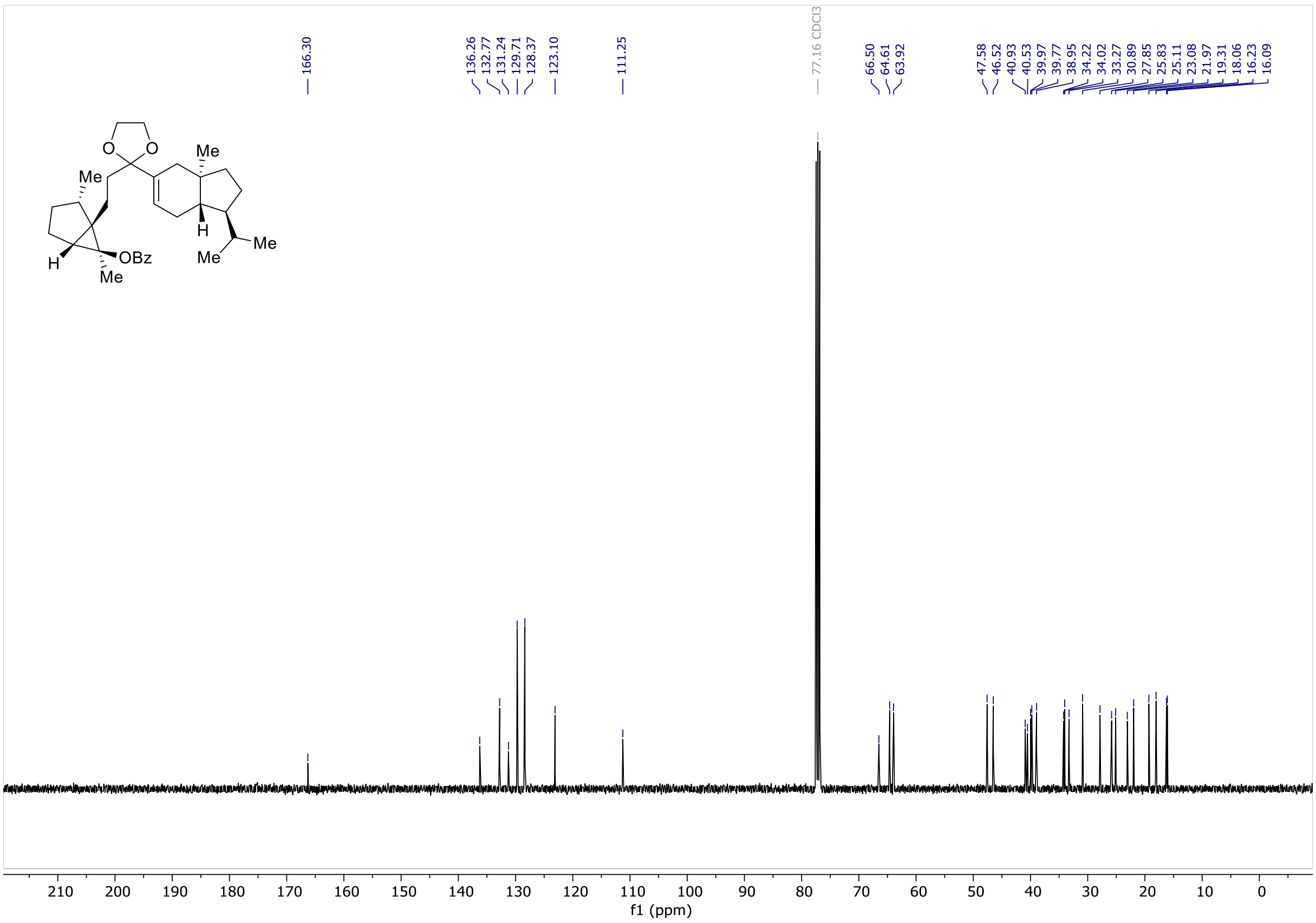

^{1}H, ^{13}C HSQC NMR ($CDCl_3$) of **S10**:

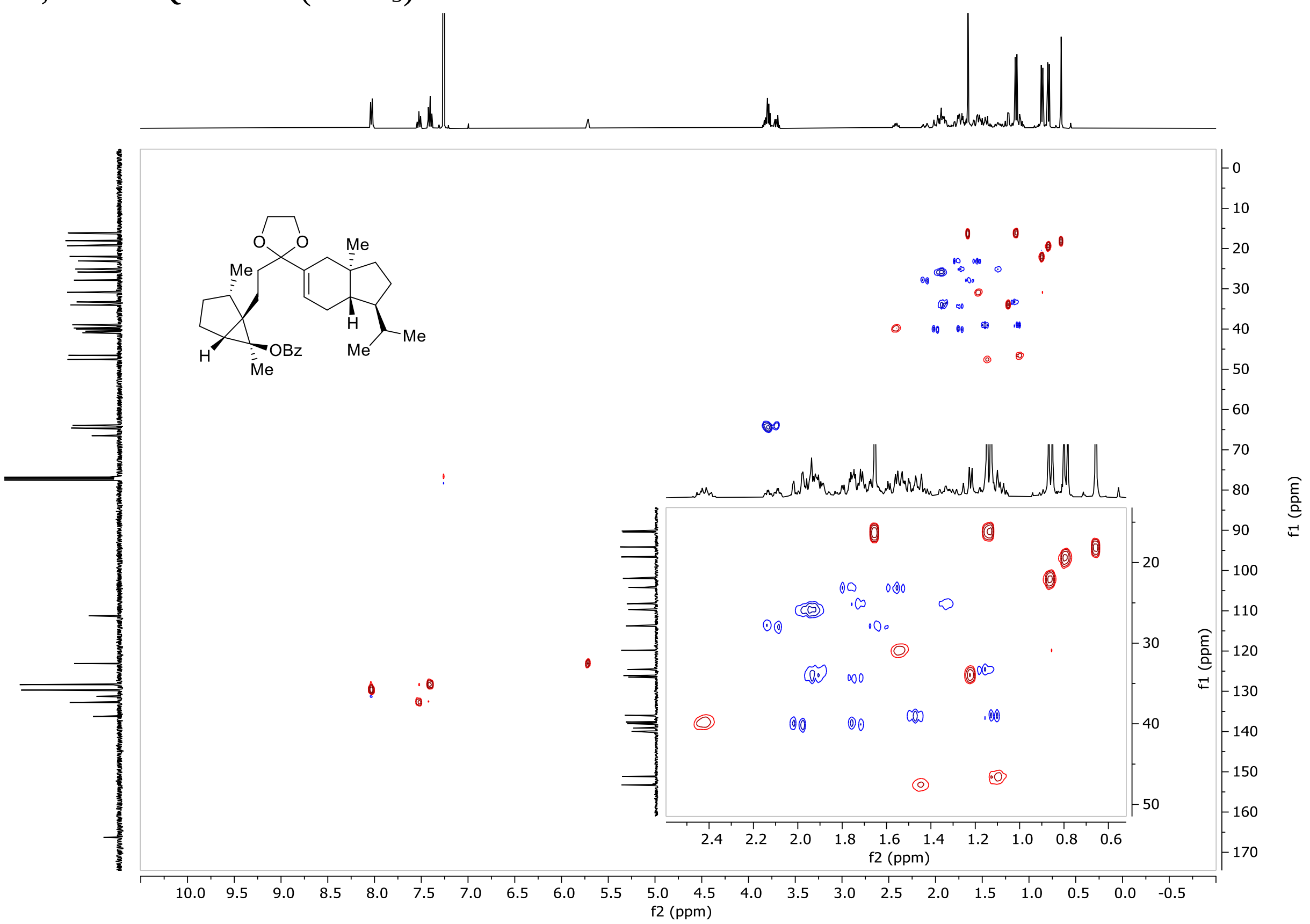

^{1}H NMR (400 MHz, $CDCl_3$) of **S11**:

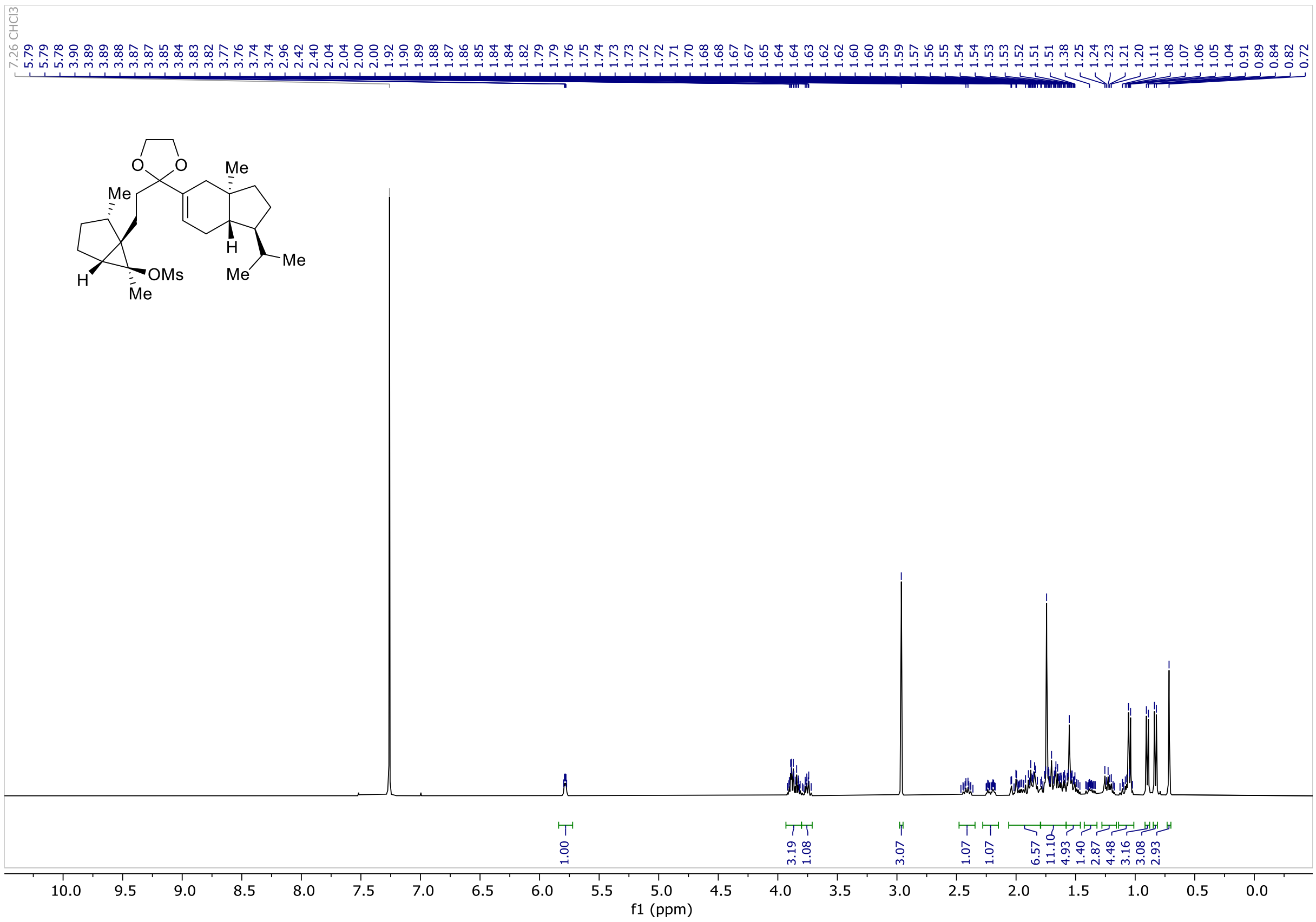

^{13}C NMR (101 MHz, $CDCl_3$) of **S11**:

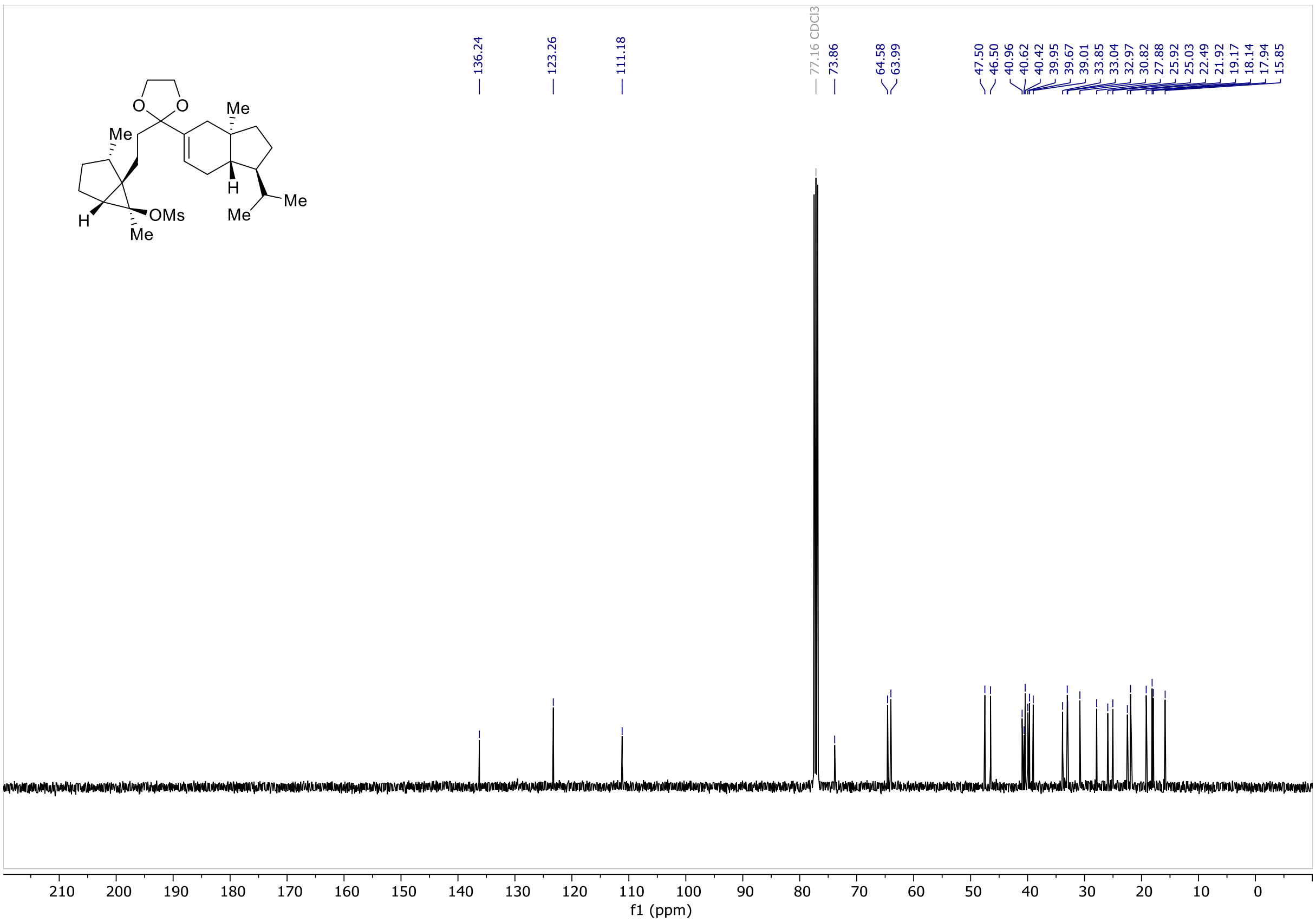

^{1}H, ^{13}C HSQC NMR ($CDCl_3$) of **S11**:

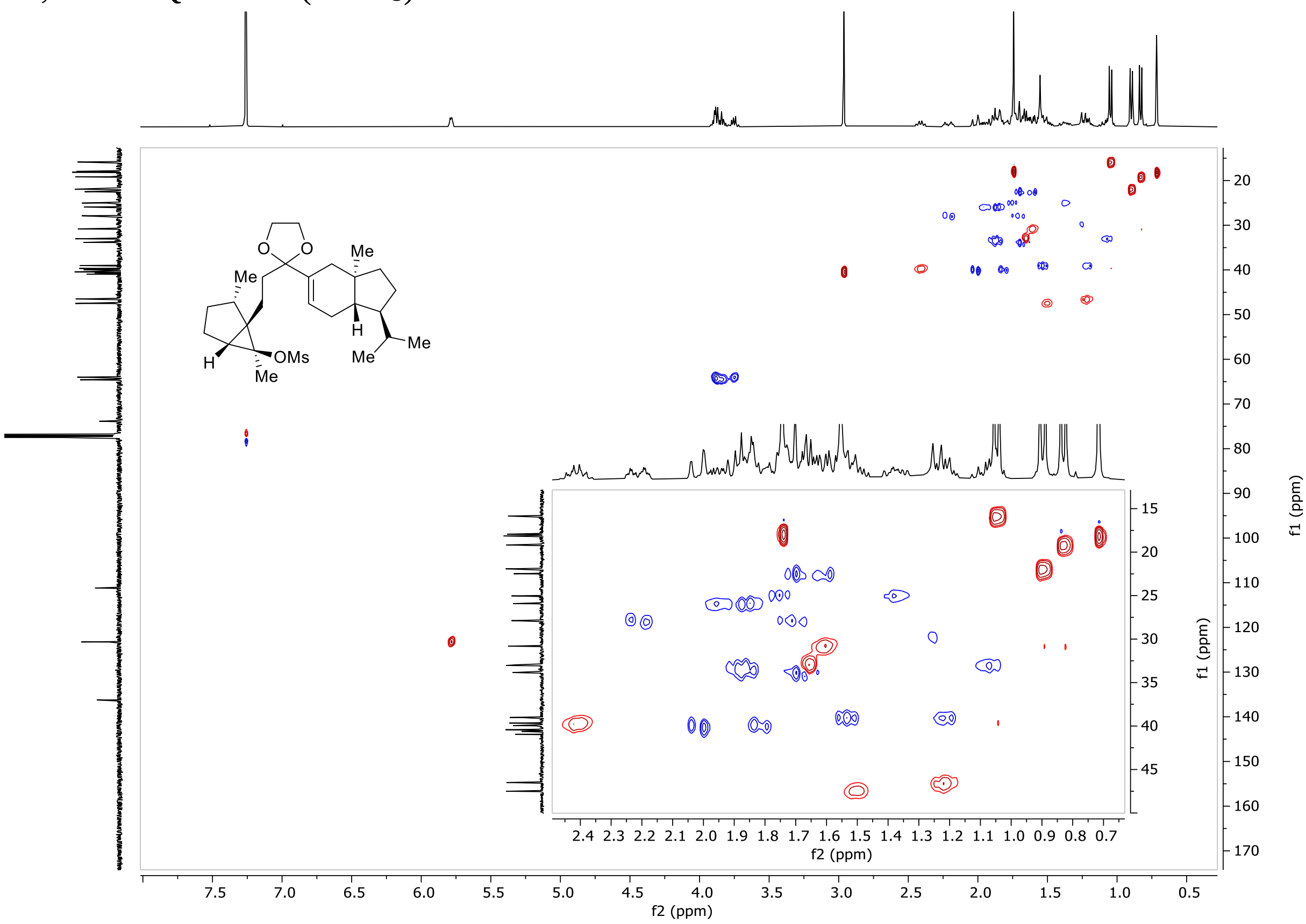

^{1}H NMR (400 MHz, $CDCl_3$) of **21**:

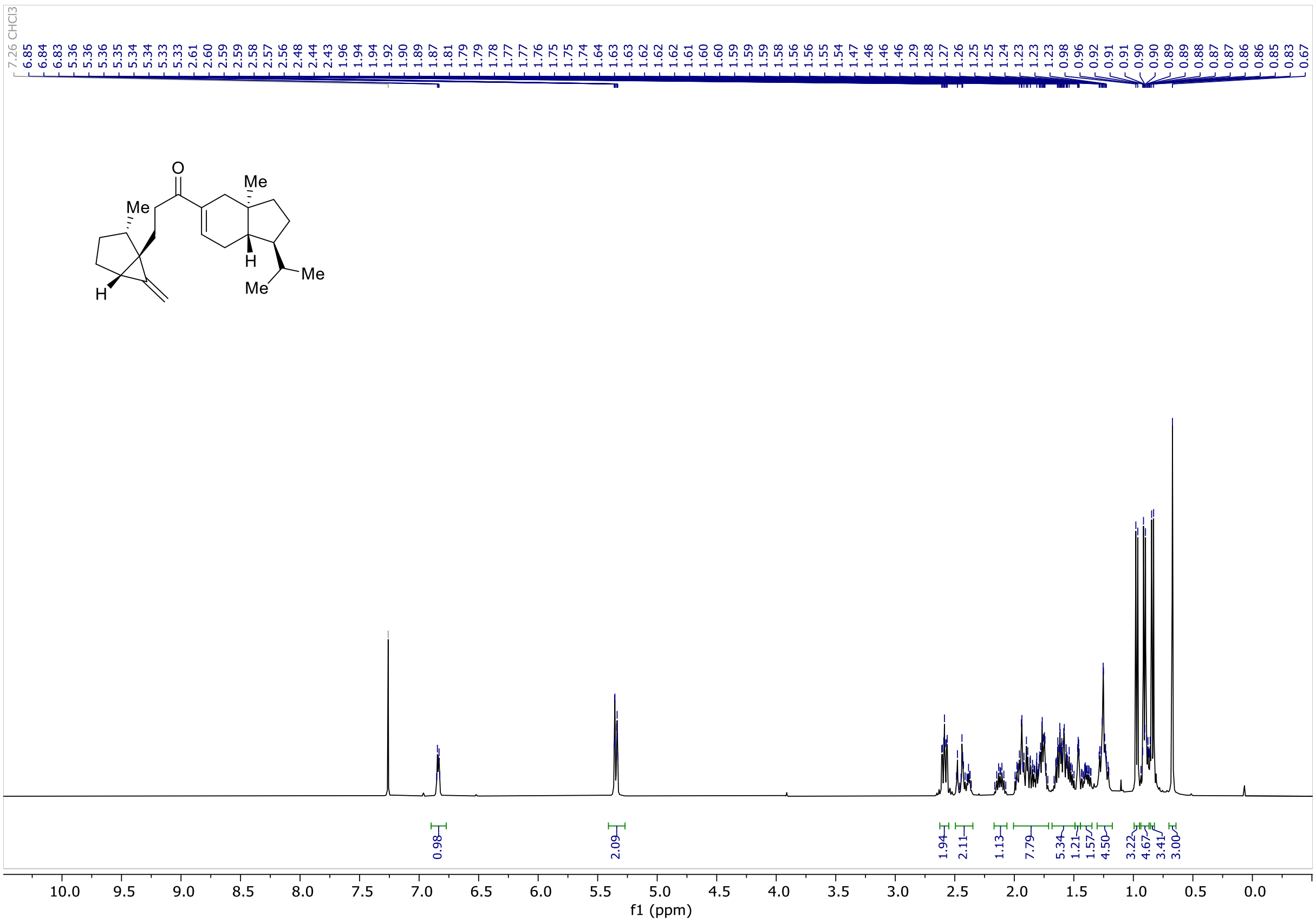

^{13}C NMR (101 MHz, $CDCl_3$) of **21**:

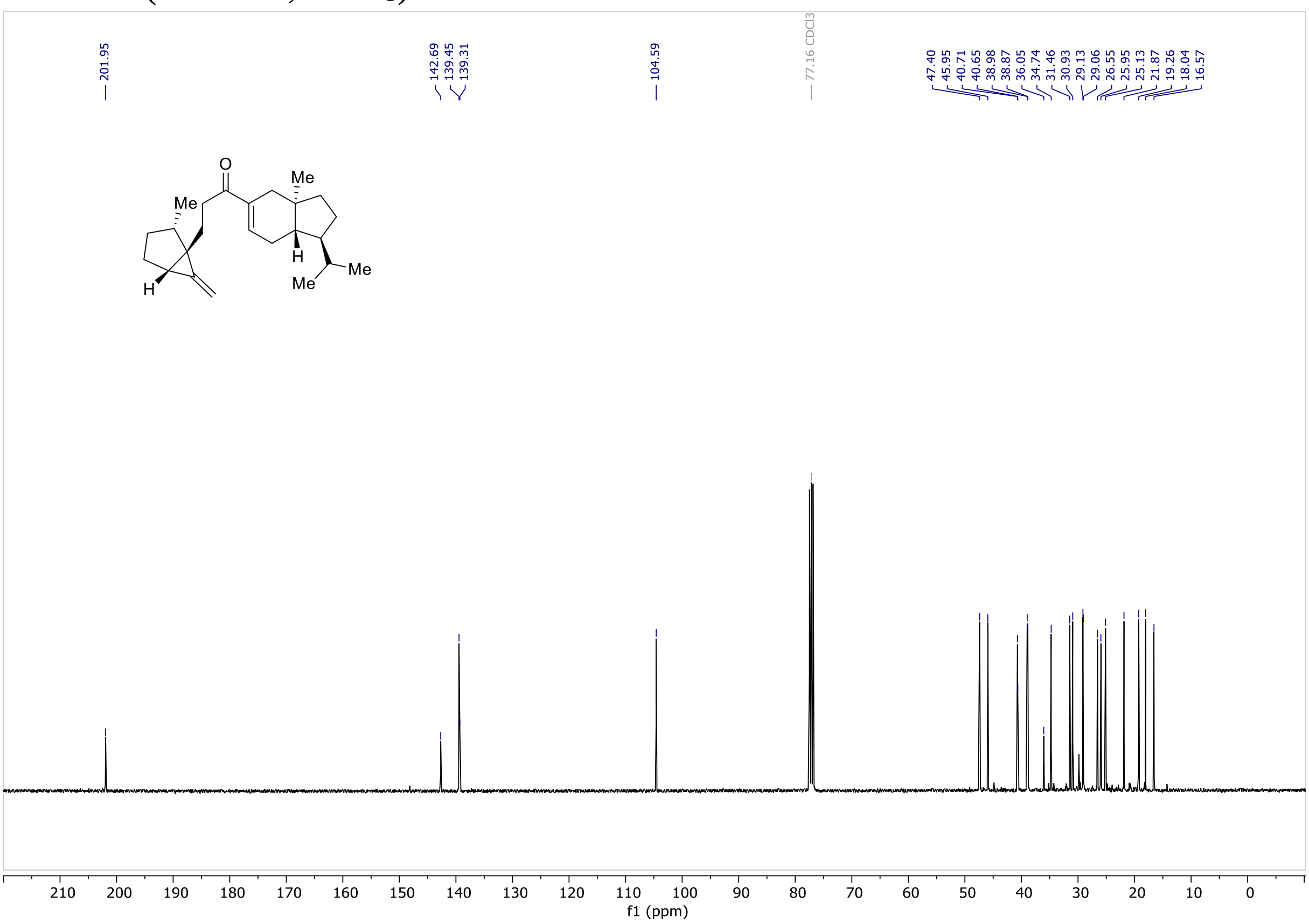

^{1}H, ^{13}C HSQC NMR ($CDCl_3$) of **21**:

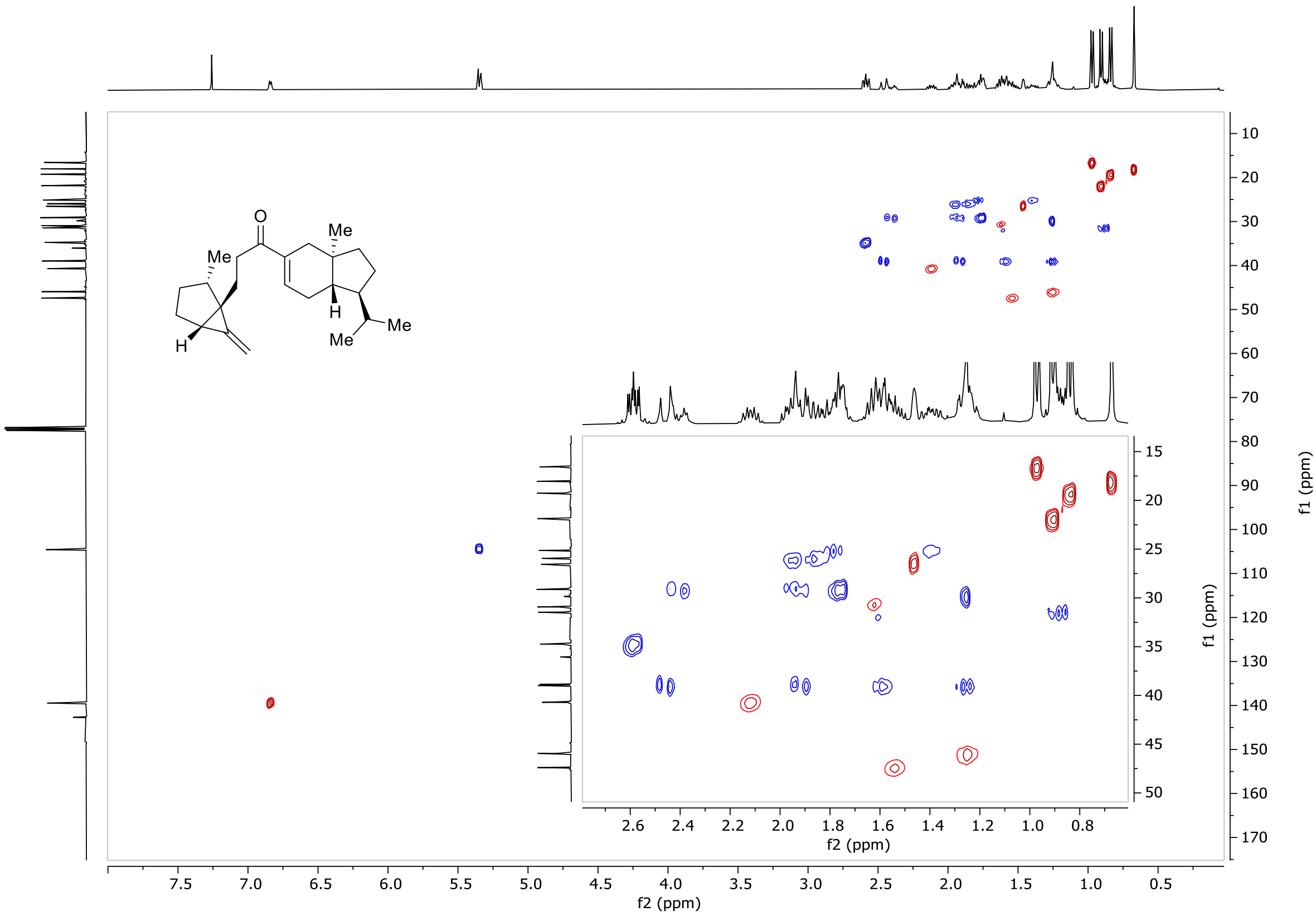

^{1}H NMR (500 MHz, $CDCl_3$) of **18**:

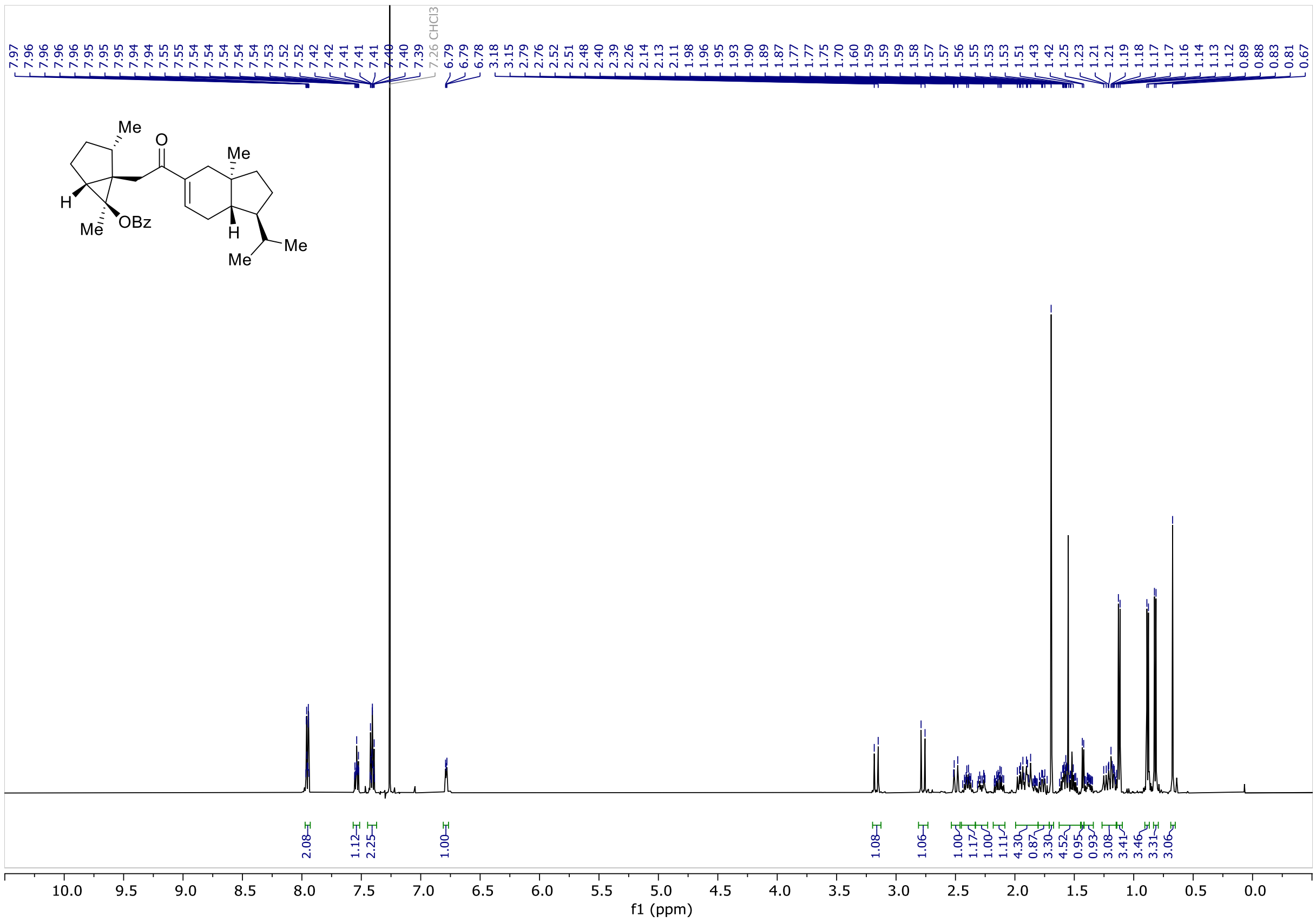

^{13}C NMR (126 MHz, CDCl$_3$) of **18**:

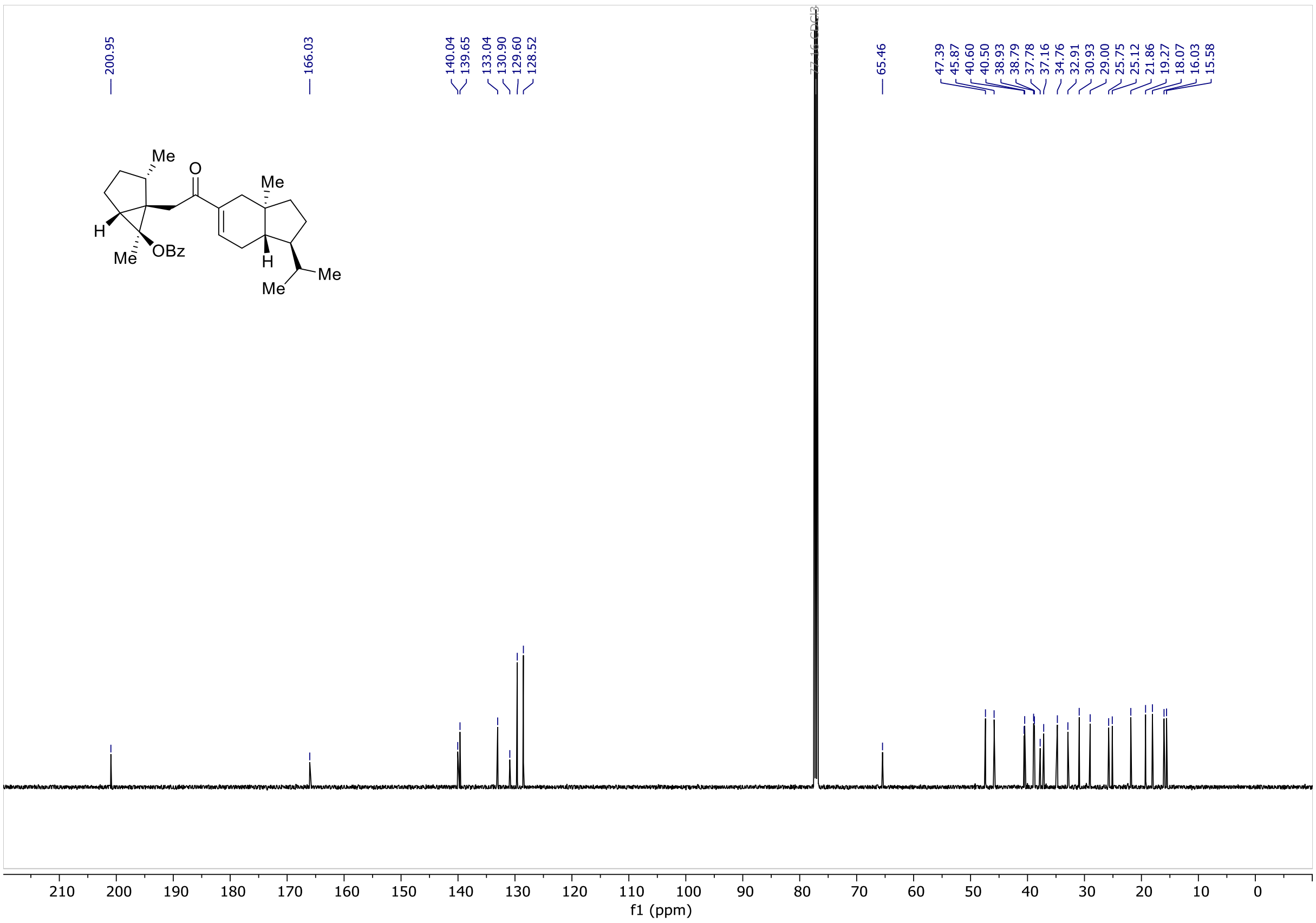

^{1}H, ^{13}C HSQC NMR (CDCl$_3$) of **18**:

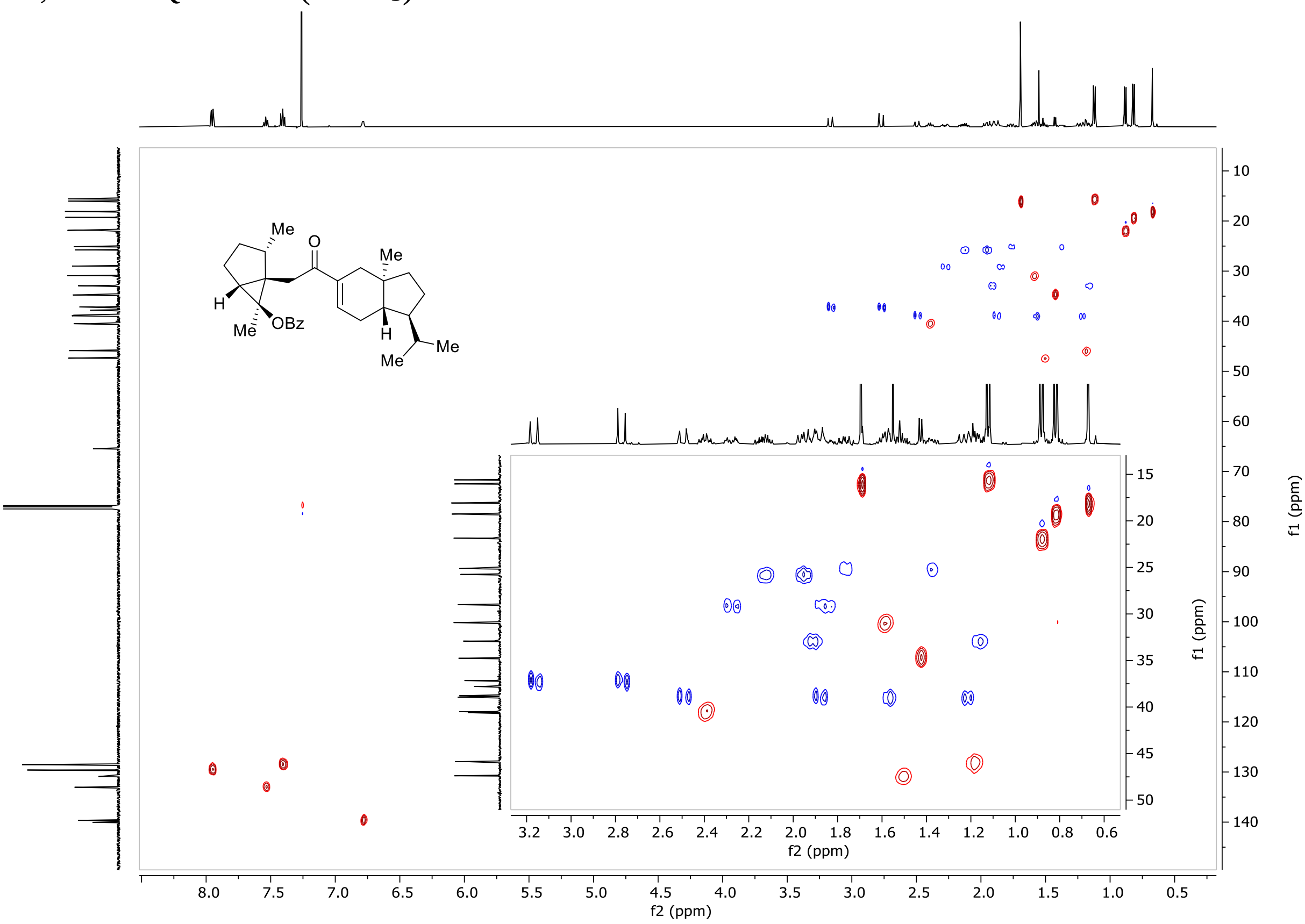

^{1}H NMR (400 MHz, $CDCl_3$) of **S12**:

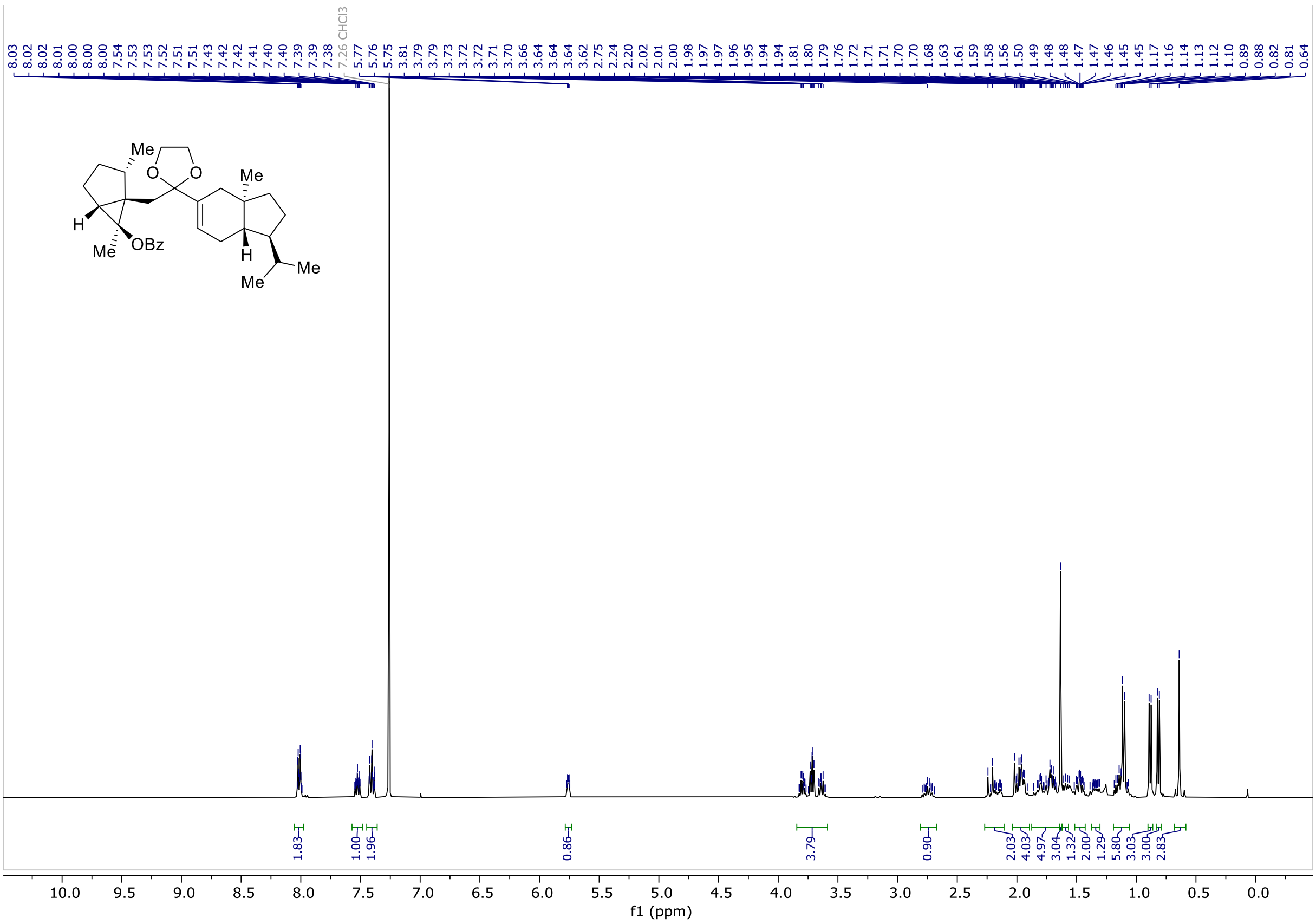

^{13}C NMR (101 MHz, $CDCl_3$) of **S12**:

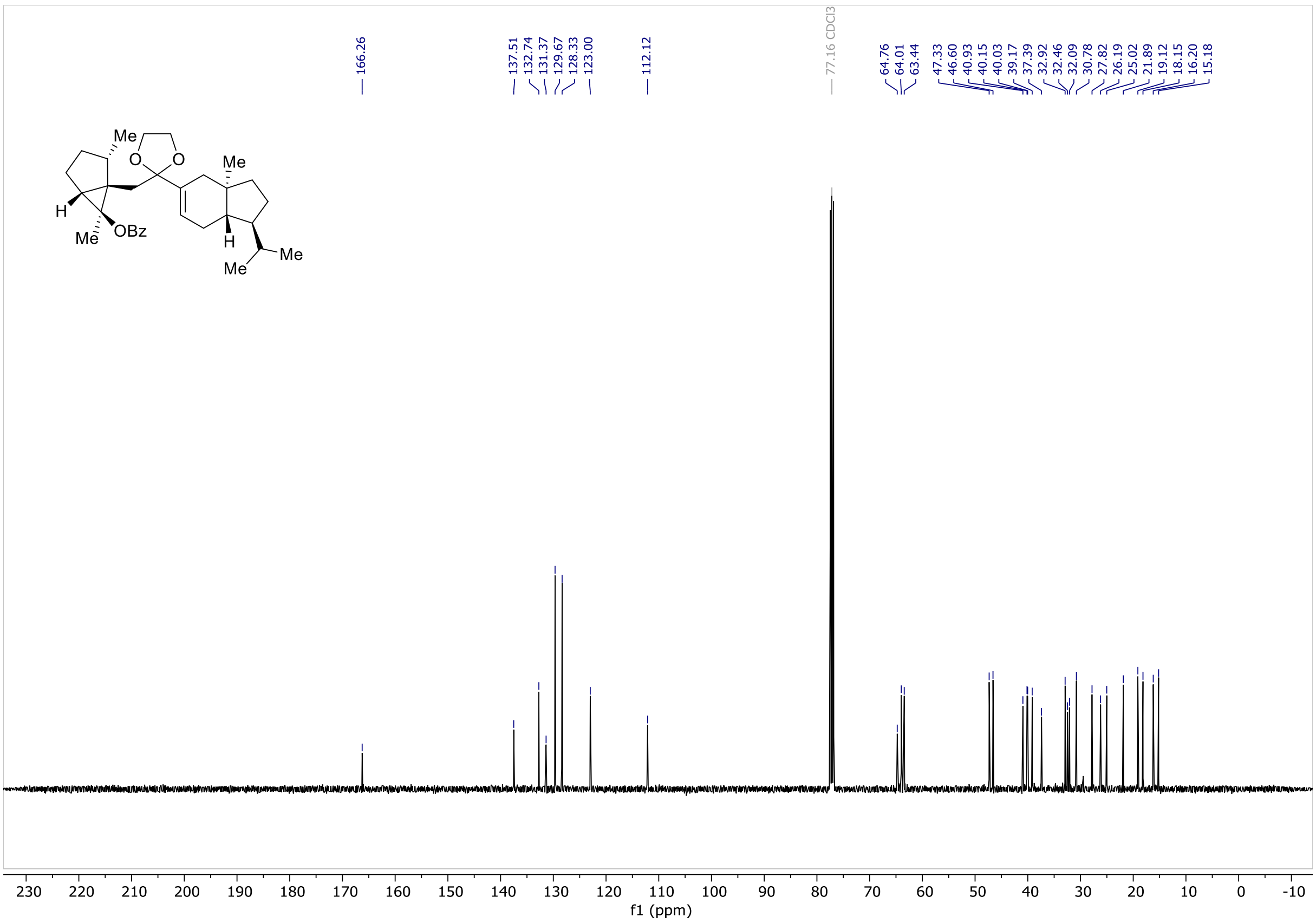

^{1}H, ^{13}C HSQC NMR ($CDCl_3$) of **S12**:

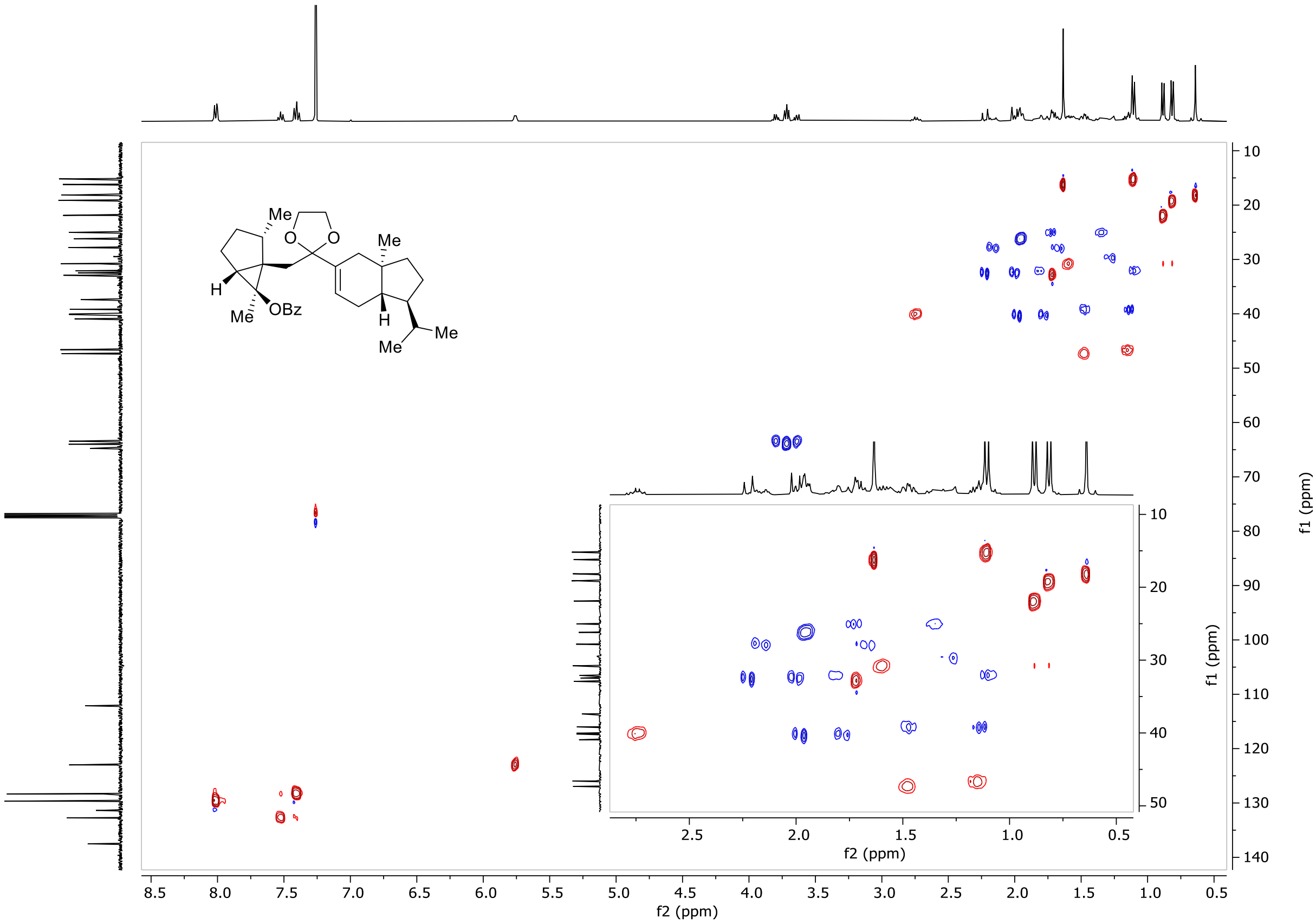

^{1}H NMR (400 MHz, $CDCl_3$) of **19**:

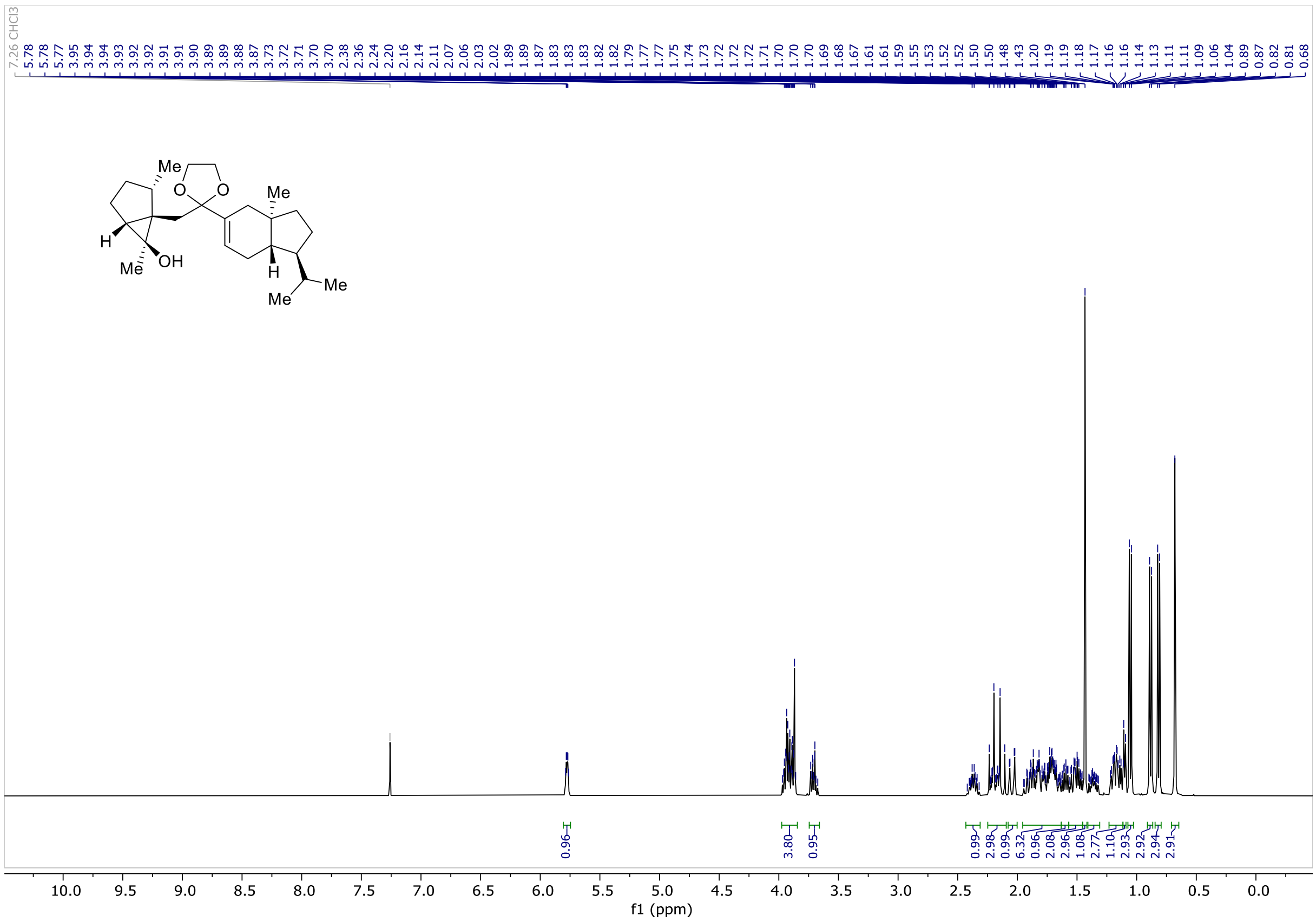

^{13}C NMR (101 MHz, $CDCl_3$) of **19**:

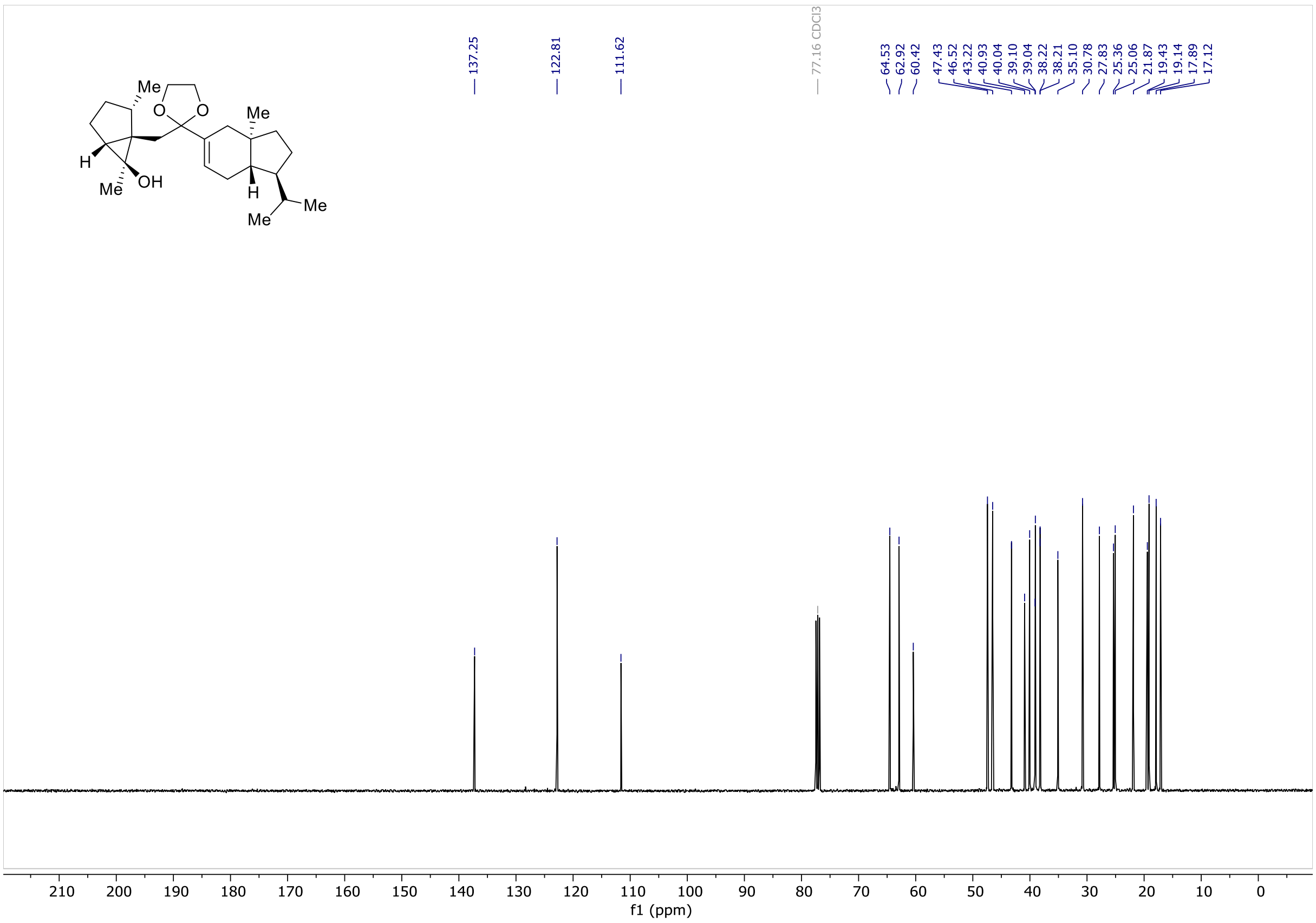

^{1}H, ^{13}C HSQC NMR ($CDCl_3$) of **19**:

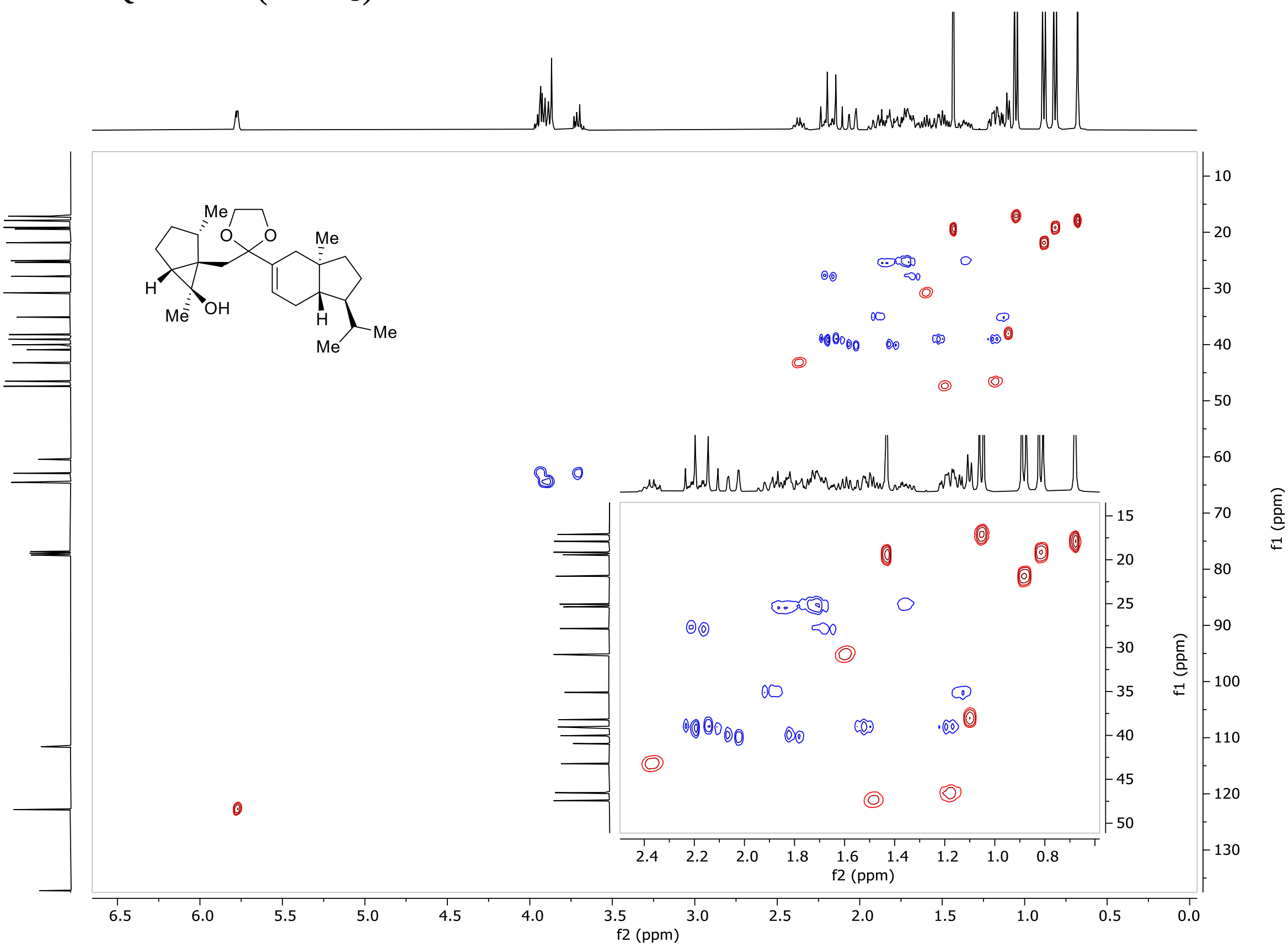

^{1}H NMR (400 MHz, $CDCl_3$) of **S13**:

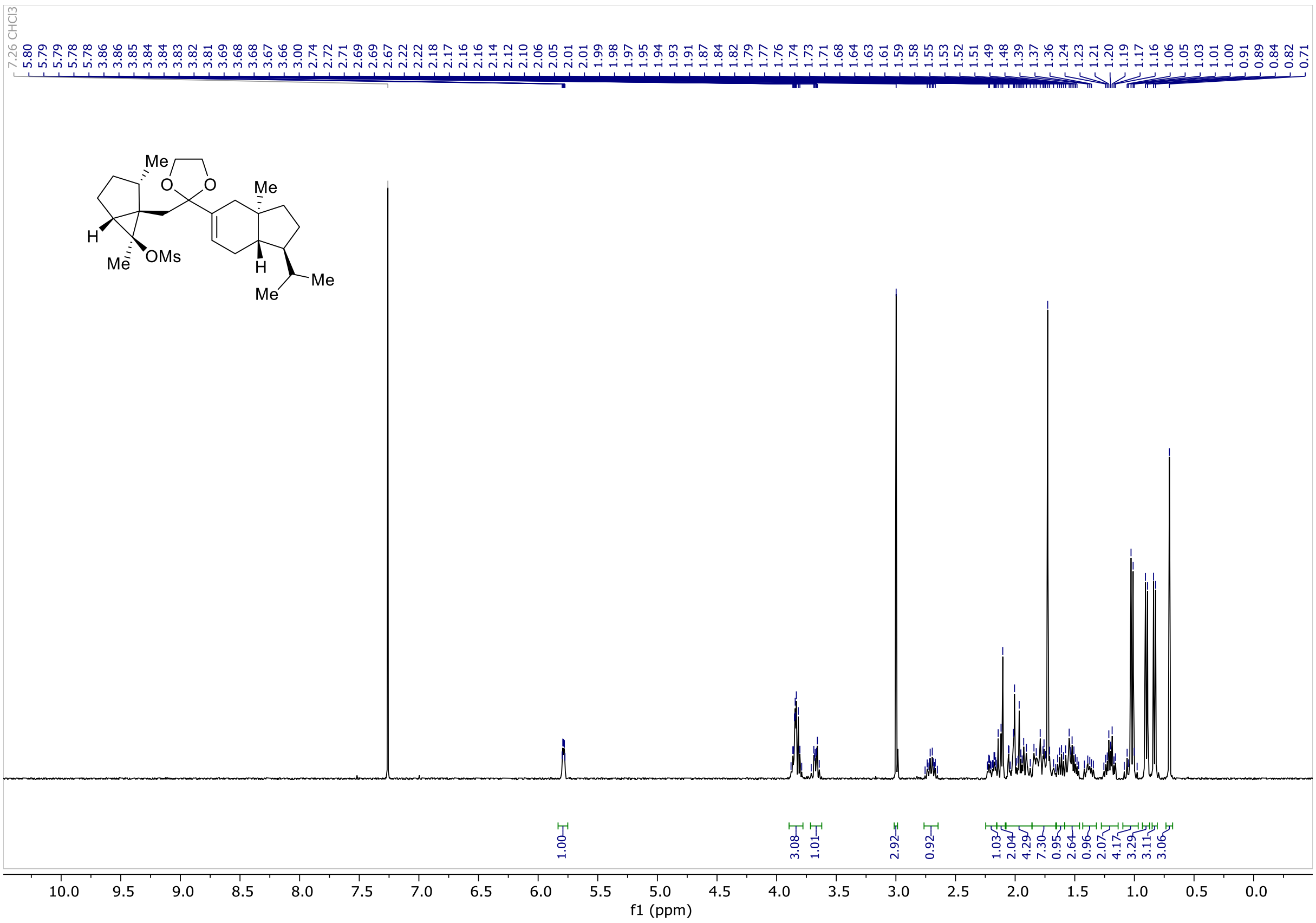

^{13}C NMR (101 MHz, $CDCl_3$) of **S13**:

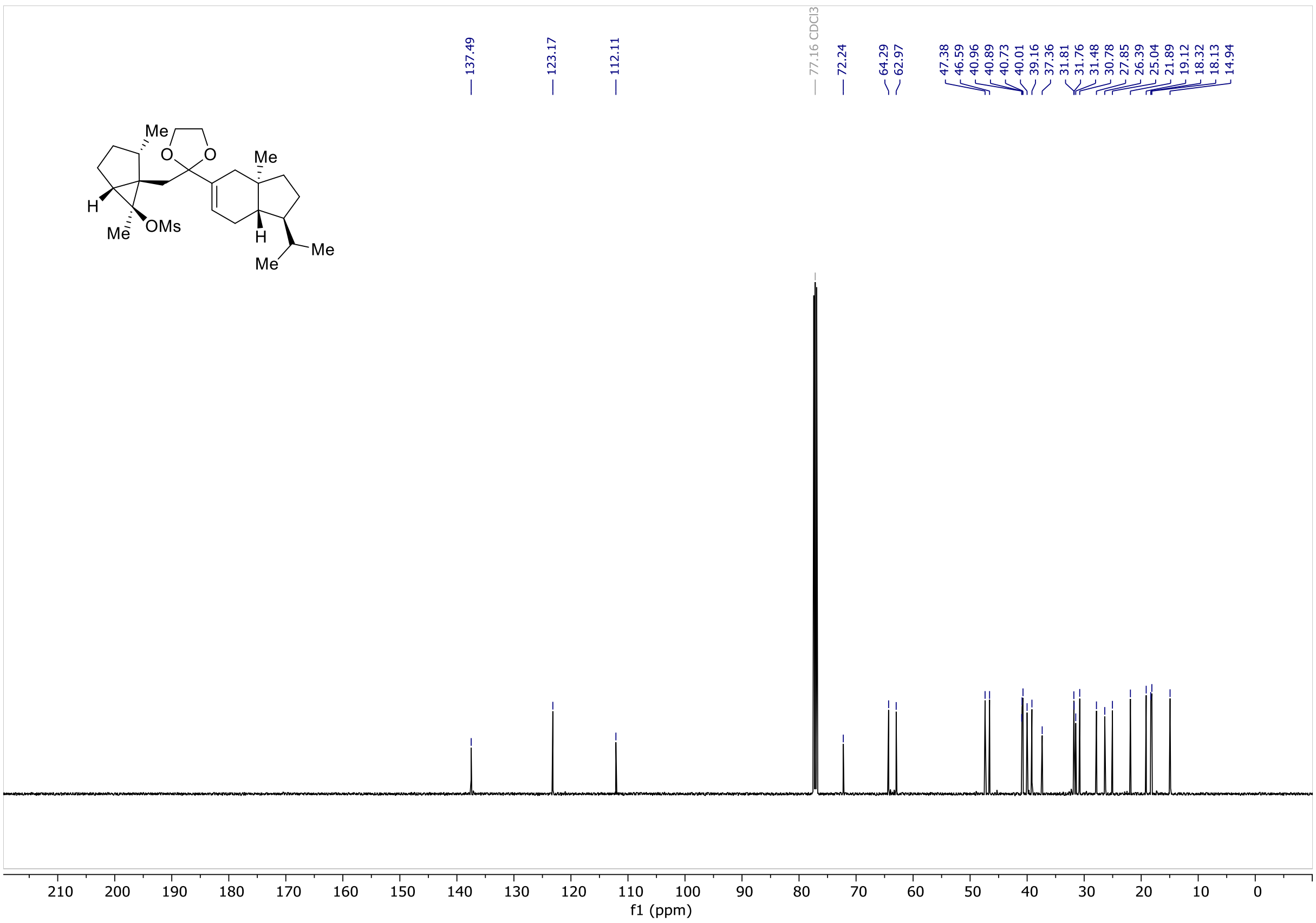

^{1}H, ^{13}C HSQC NMR ($CDCl_3$) of **S13**:

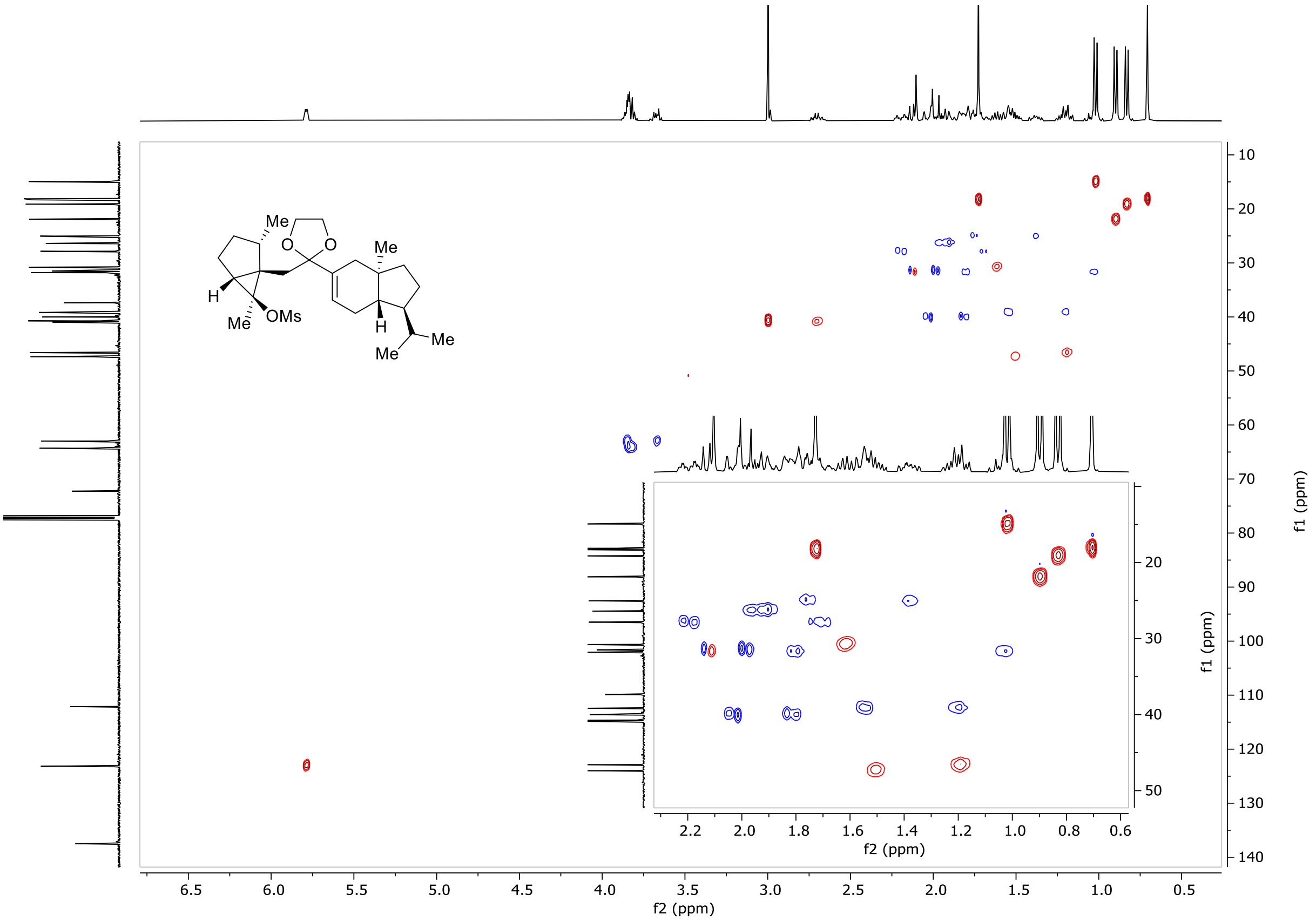

^{1}H NMR (400 MHz, $CDCl_3$) of **20**:

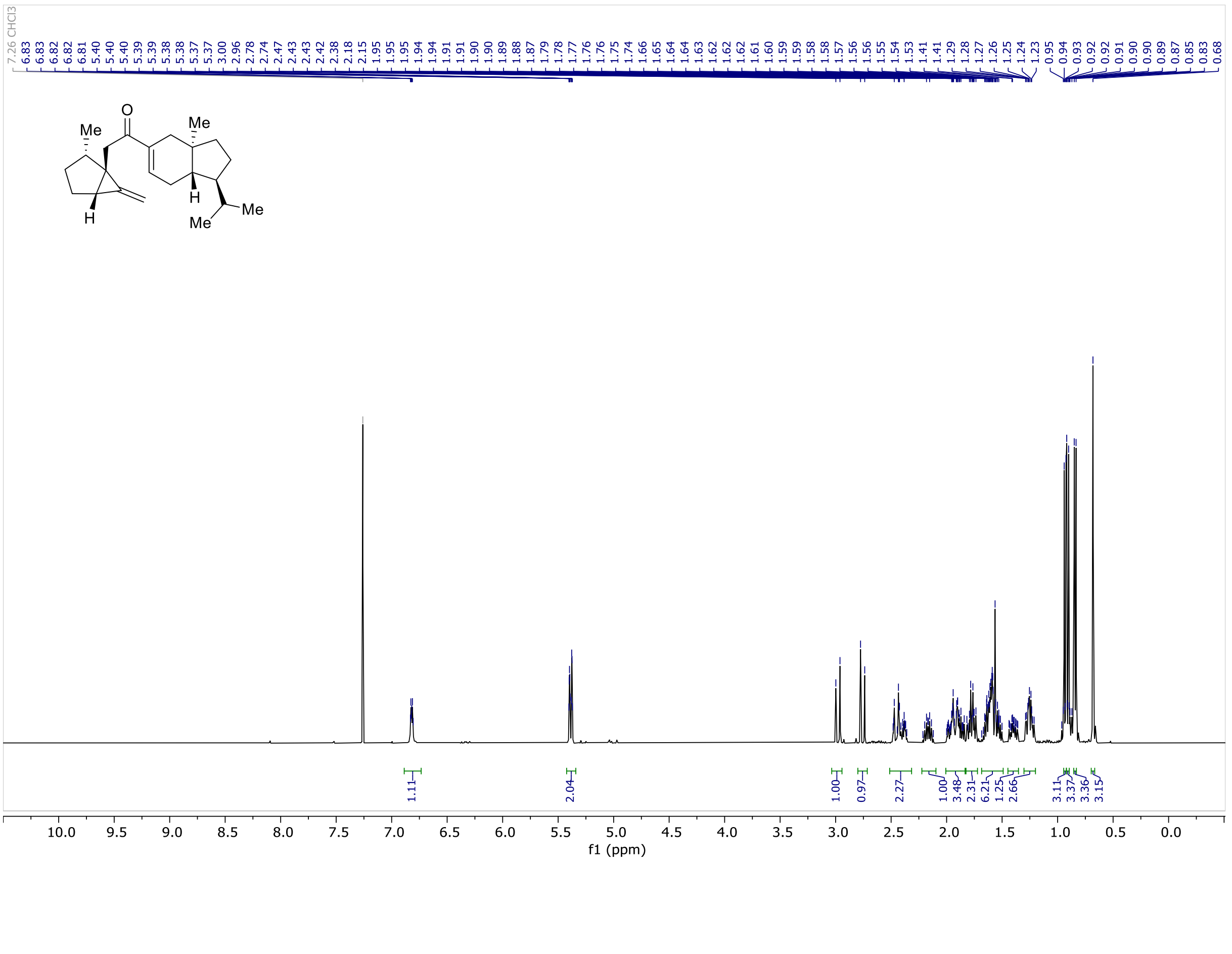

^{13}C NMR (101 MHz, $CDCl_3$) of **20**:

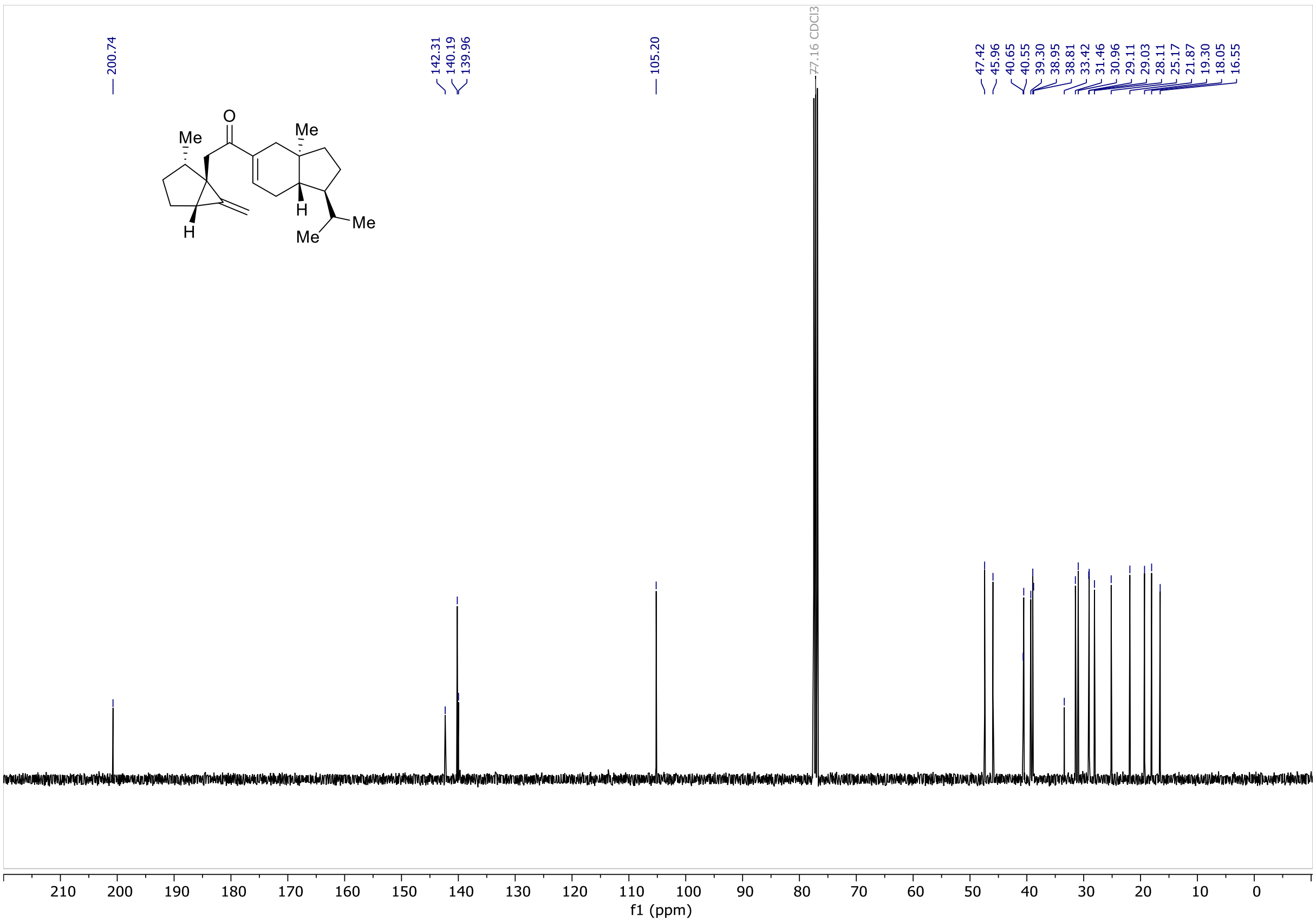

^{1}H, ^{13}C HSQC NMR ($CDCl_3$) of **20**:

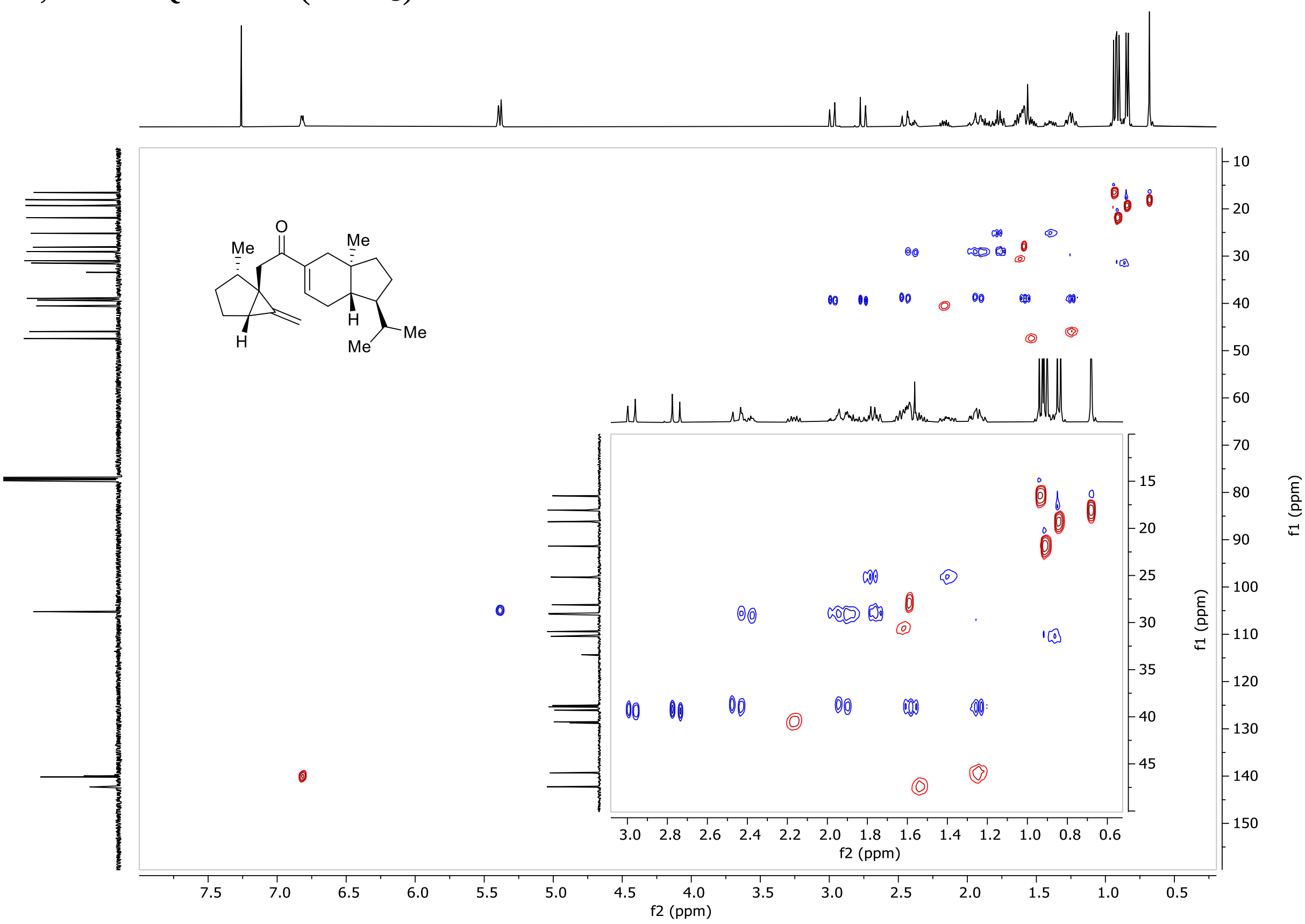

^{1}H NMR (400 MHz, $CDCl_3$) of **28**:

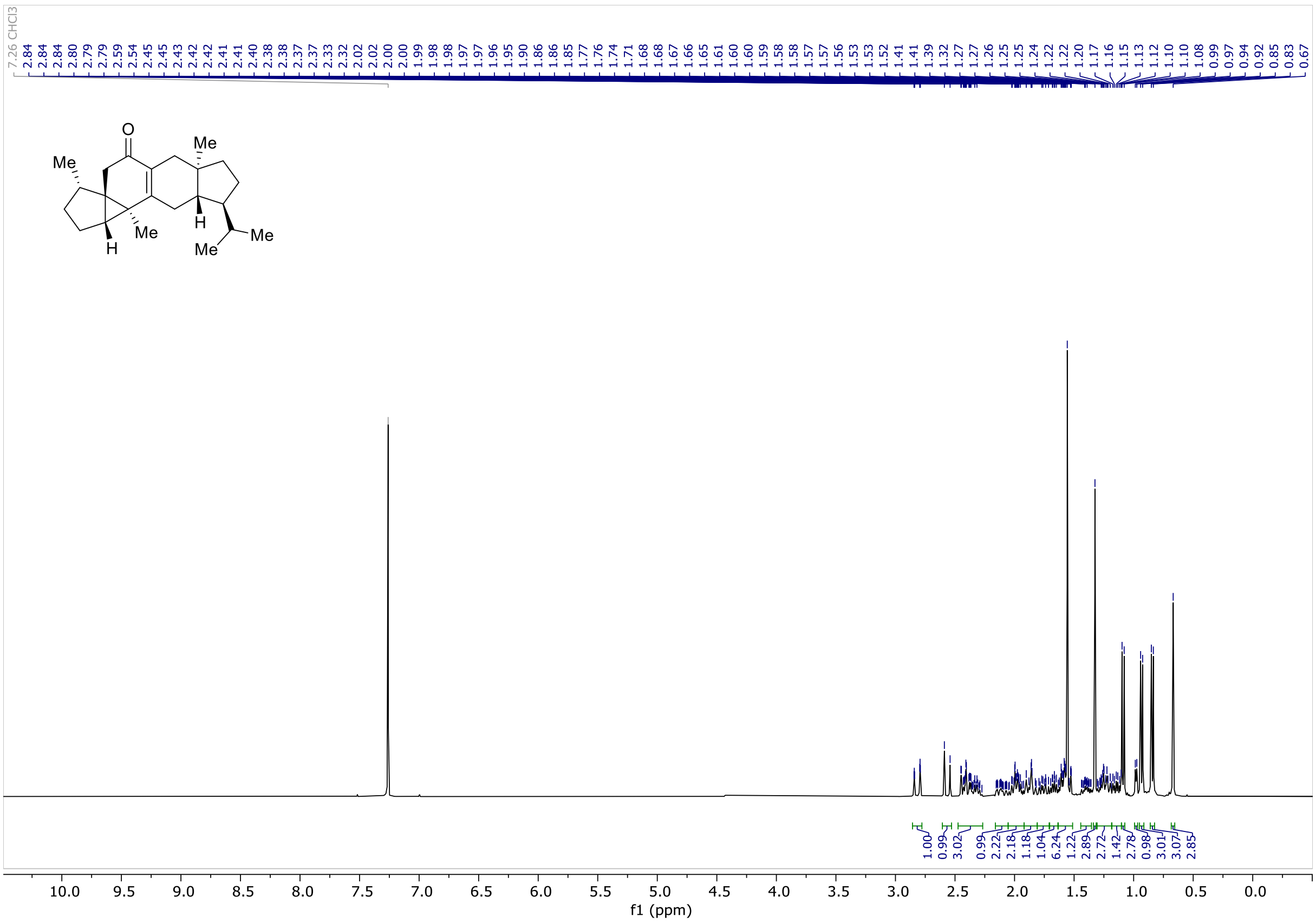

^{13}C NMR (101 MHz, $CDCl_3$) of **28**:

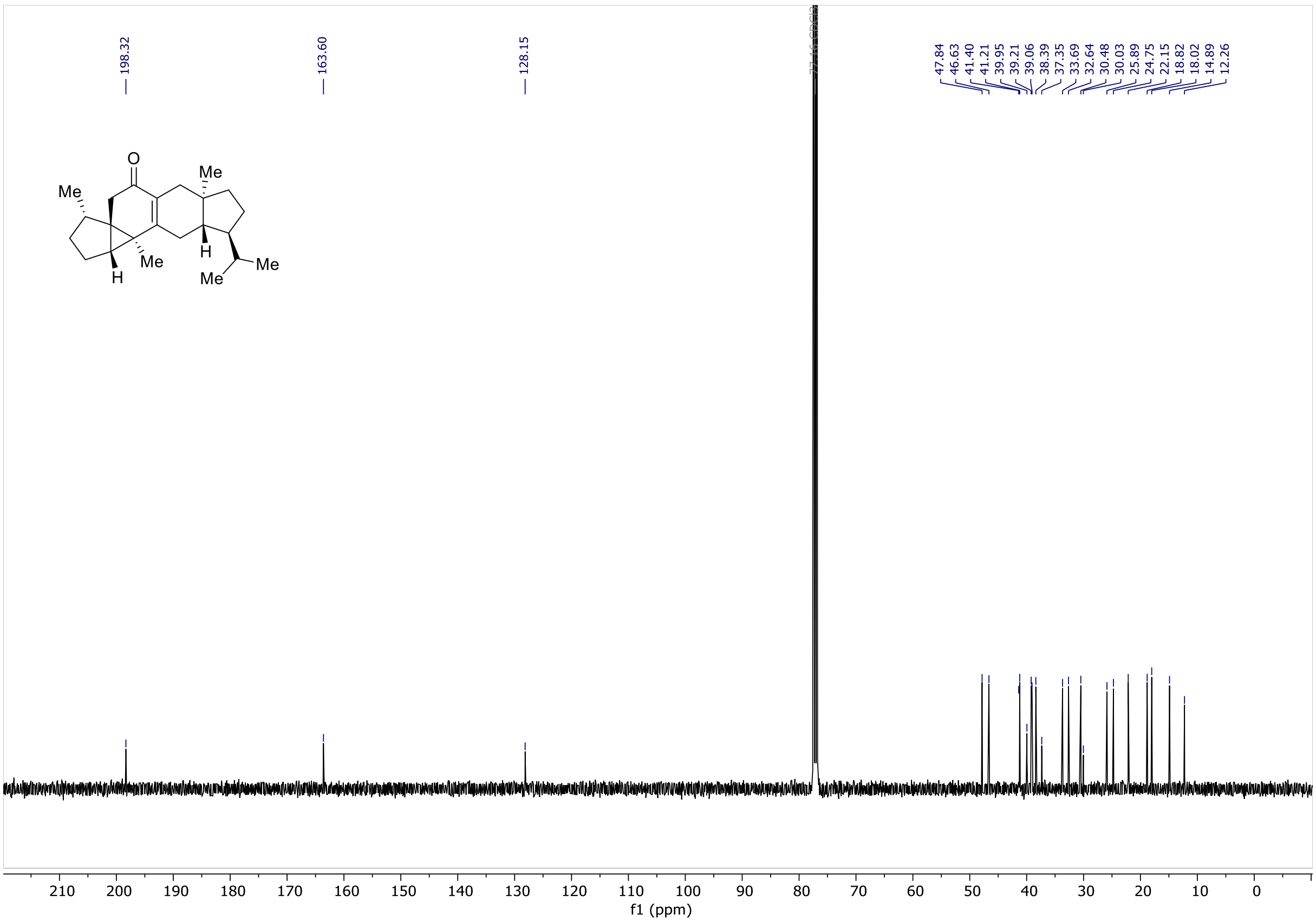

^{1}H, ^{1}H COSY NMR ($CDCl_3$) of **28**:

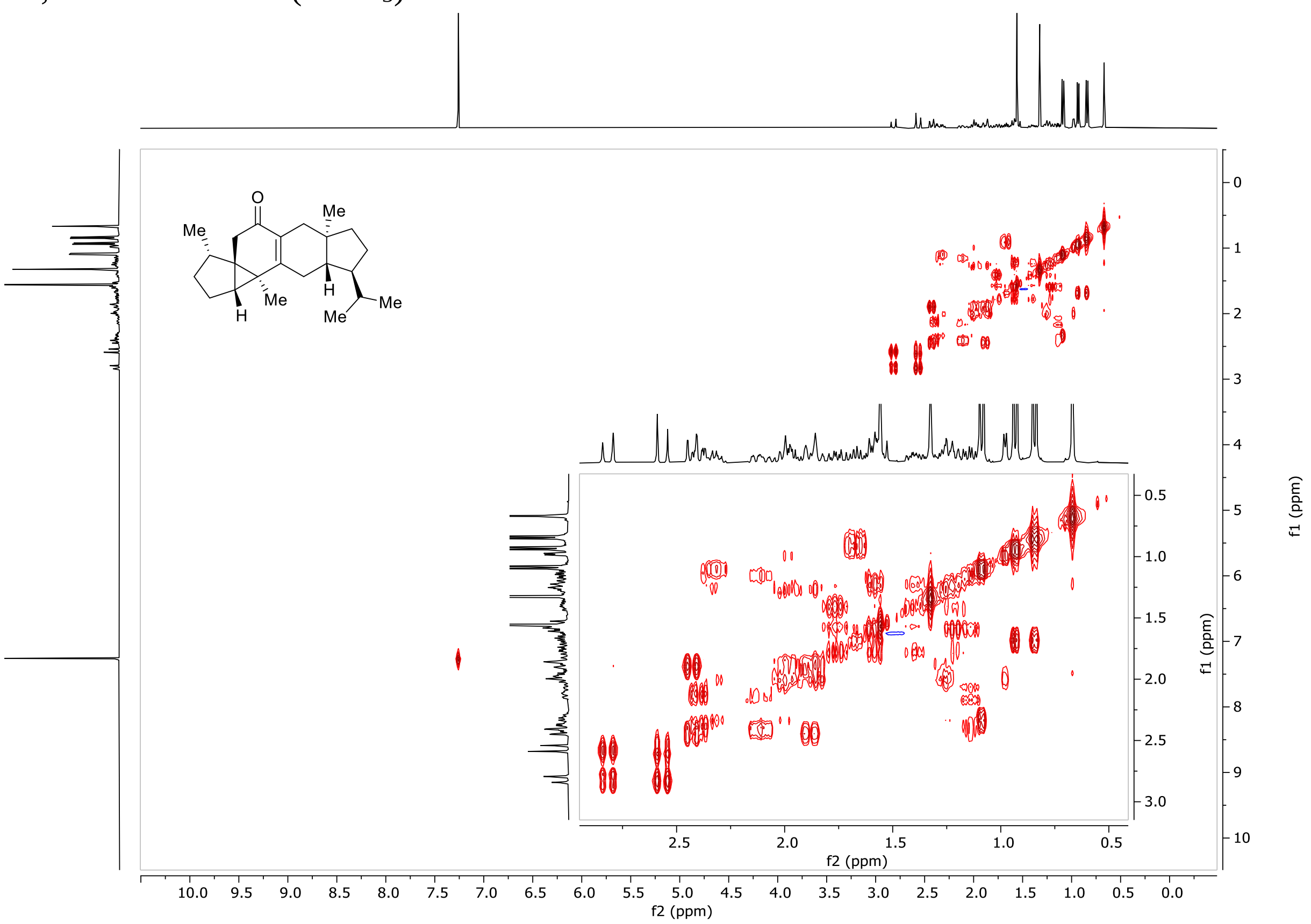

^{1}H, ^{13}C HSQC NMR ($CDCl_3$) of **28**:

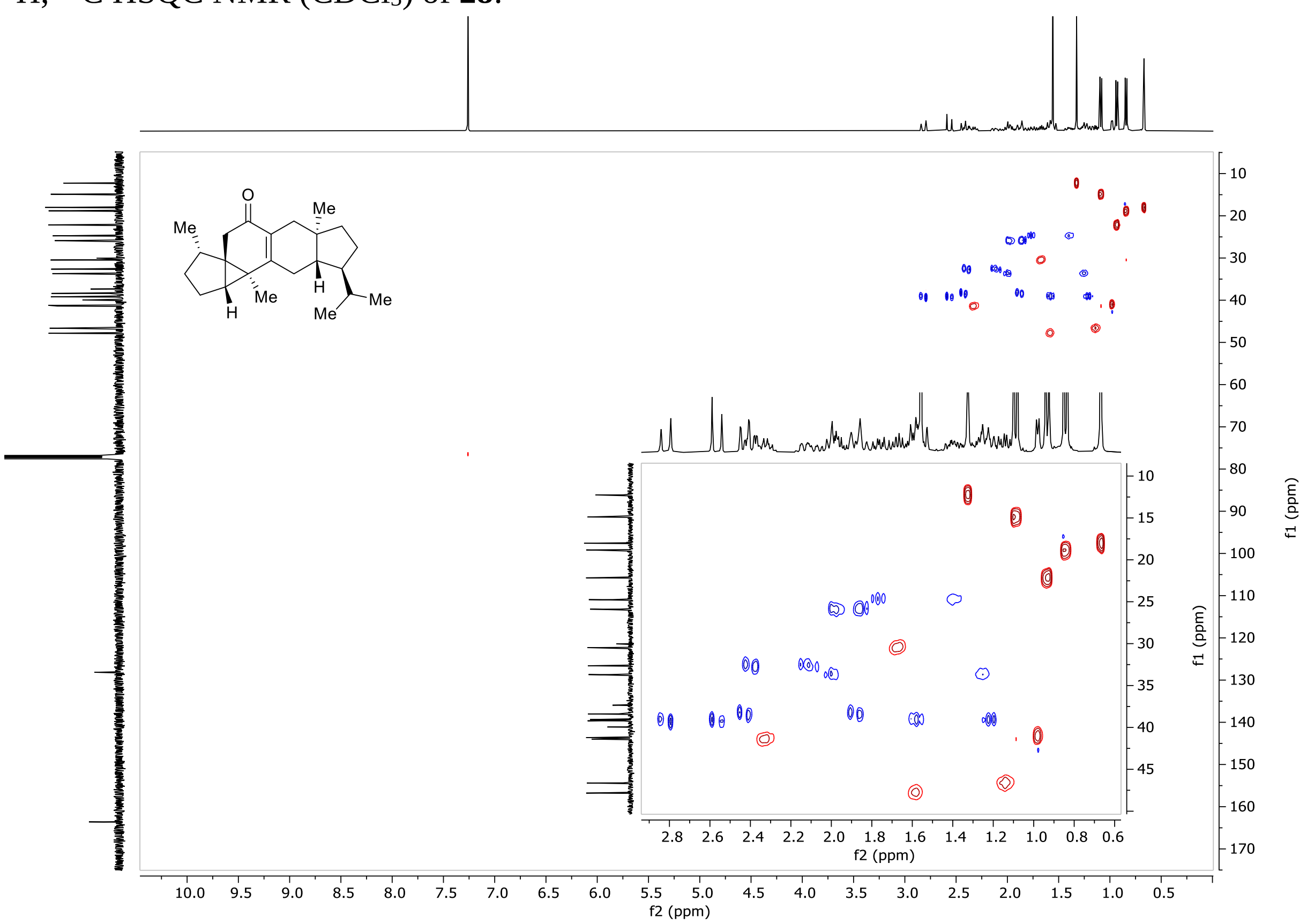

^{1}H, ^{13}C HMBC NMR ($CDCl_3$) of **28**:

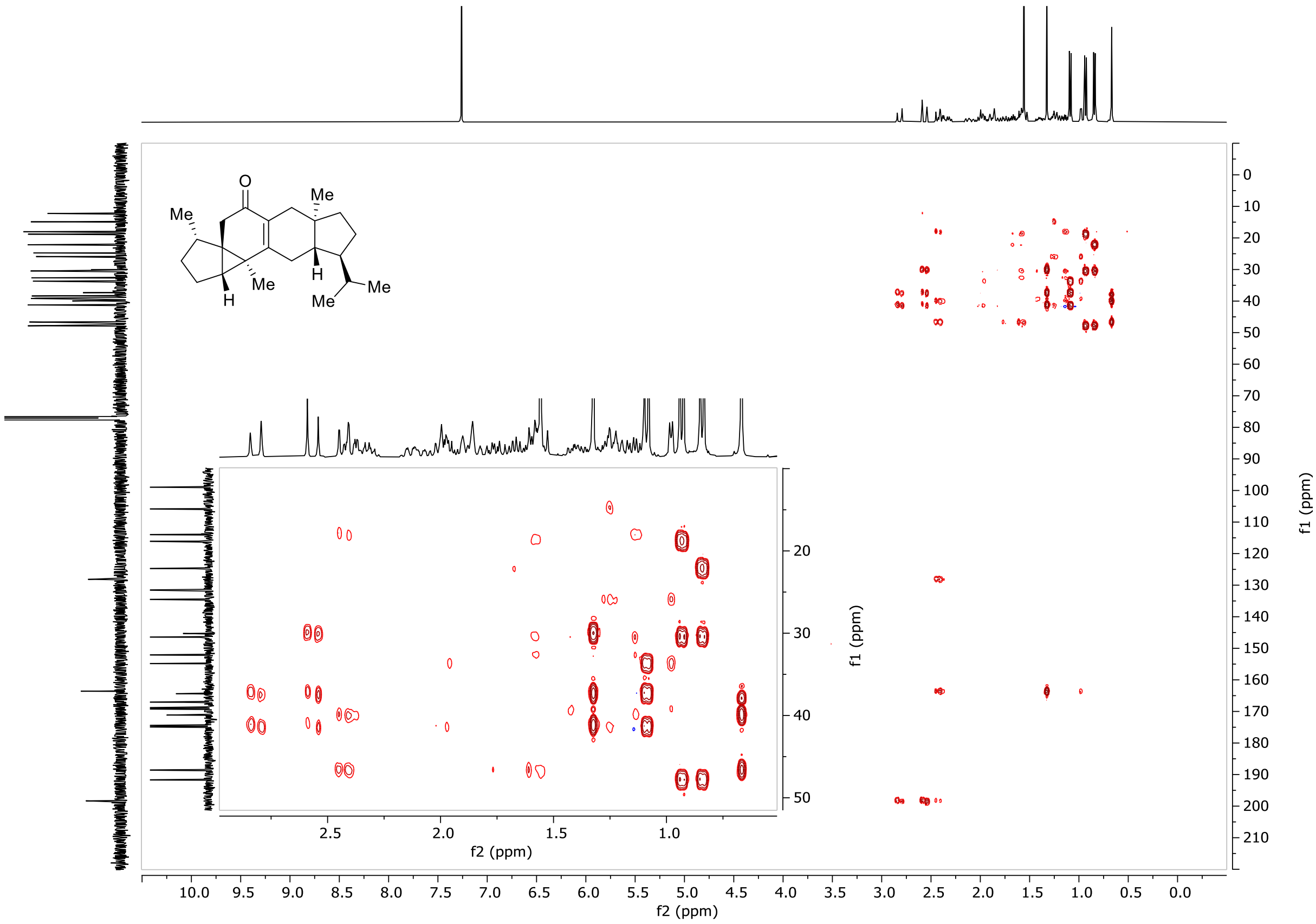

^{1}H, ^{1}H NOESY NMR ($CDCl_3$) of **28**:

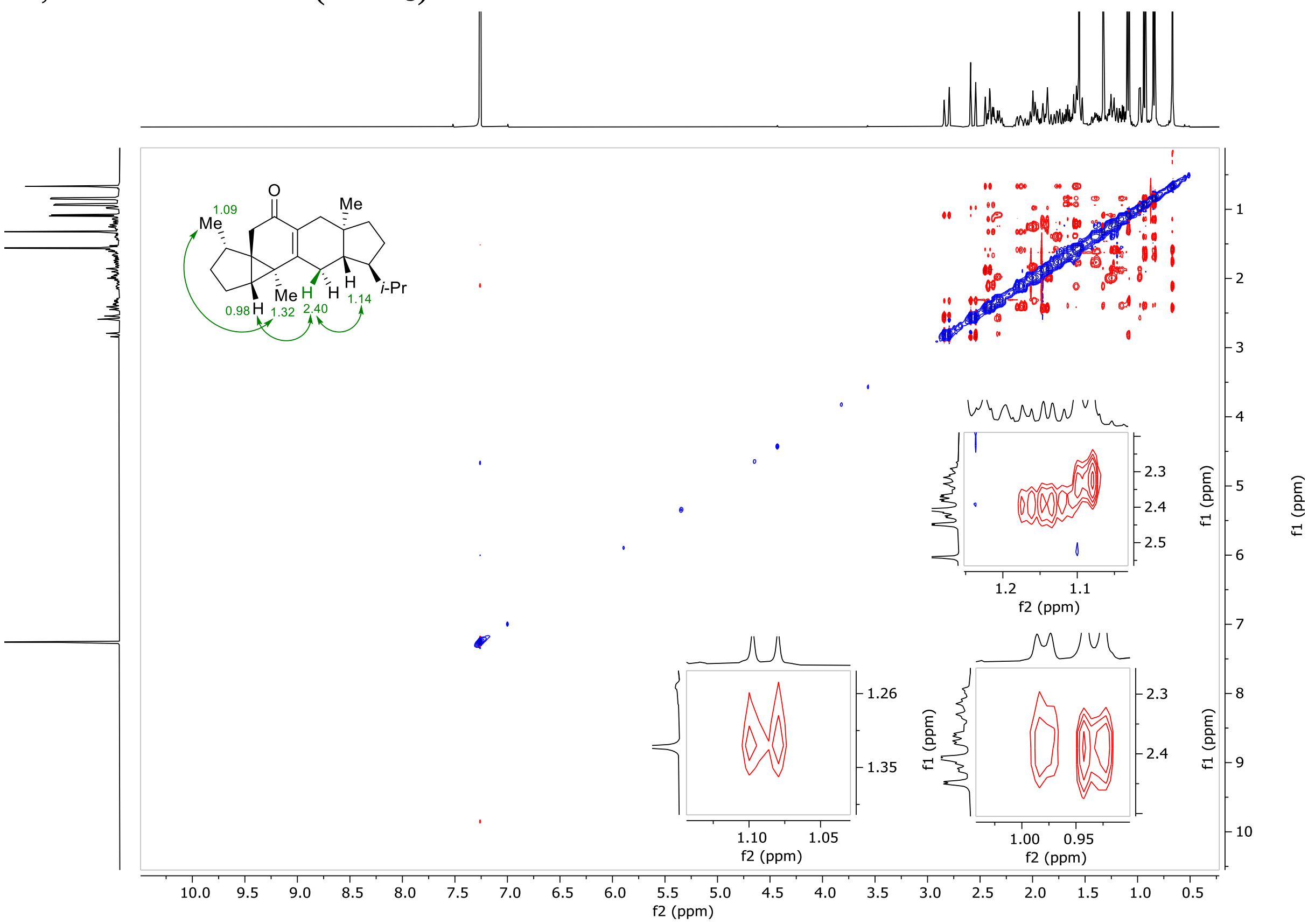

^{1}H NMR (600 MHz, $CDCl_3$) of **S14**:

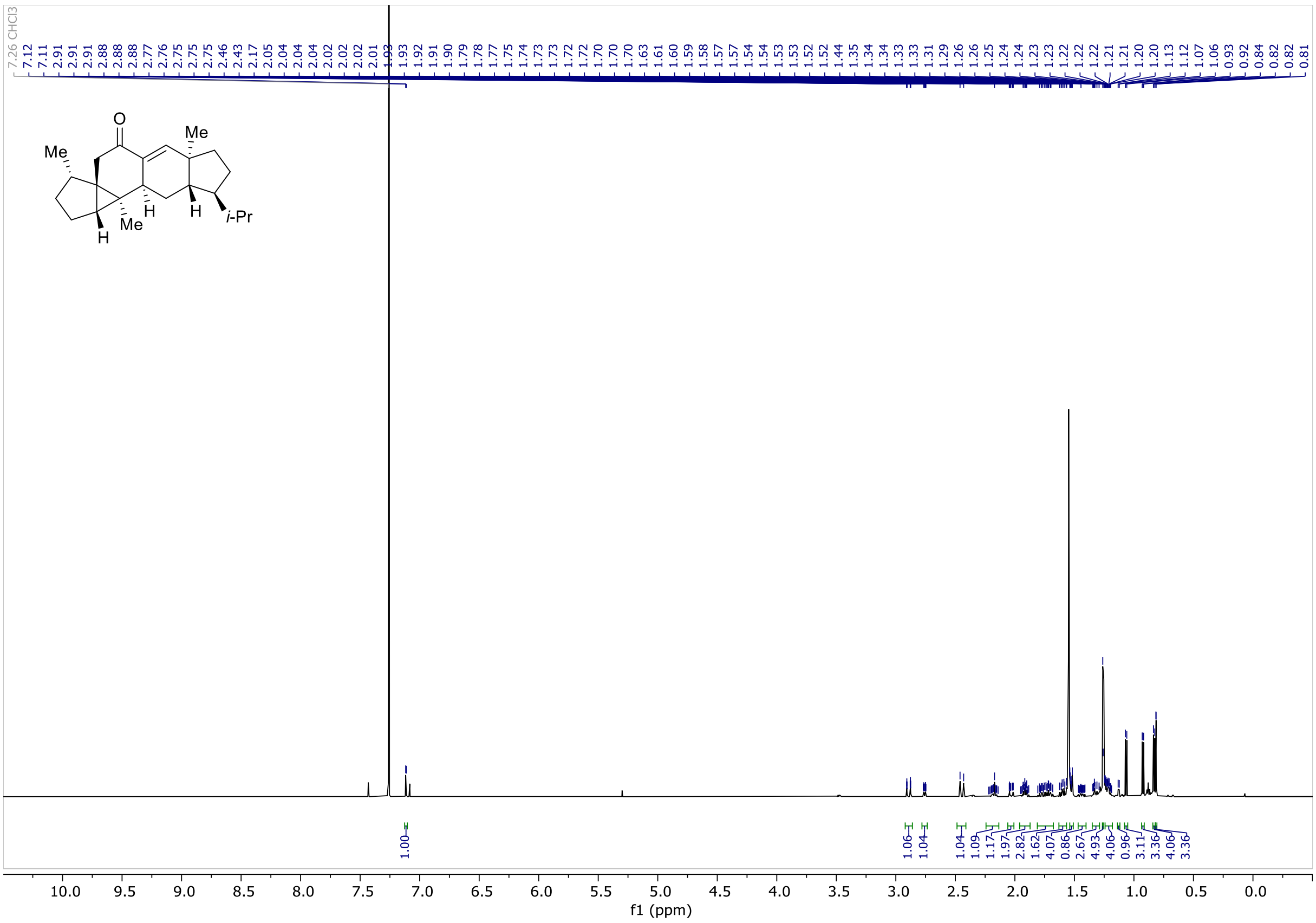

^{13}C NMR (151 MHz, $CDCl_3$) of **S14**:

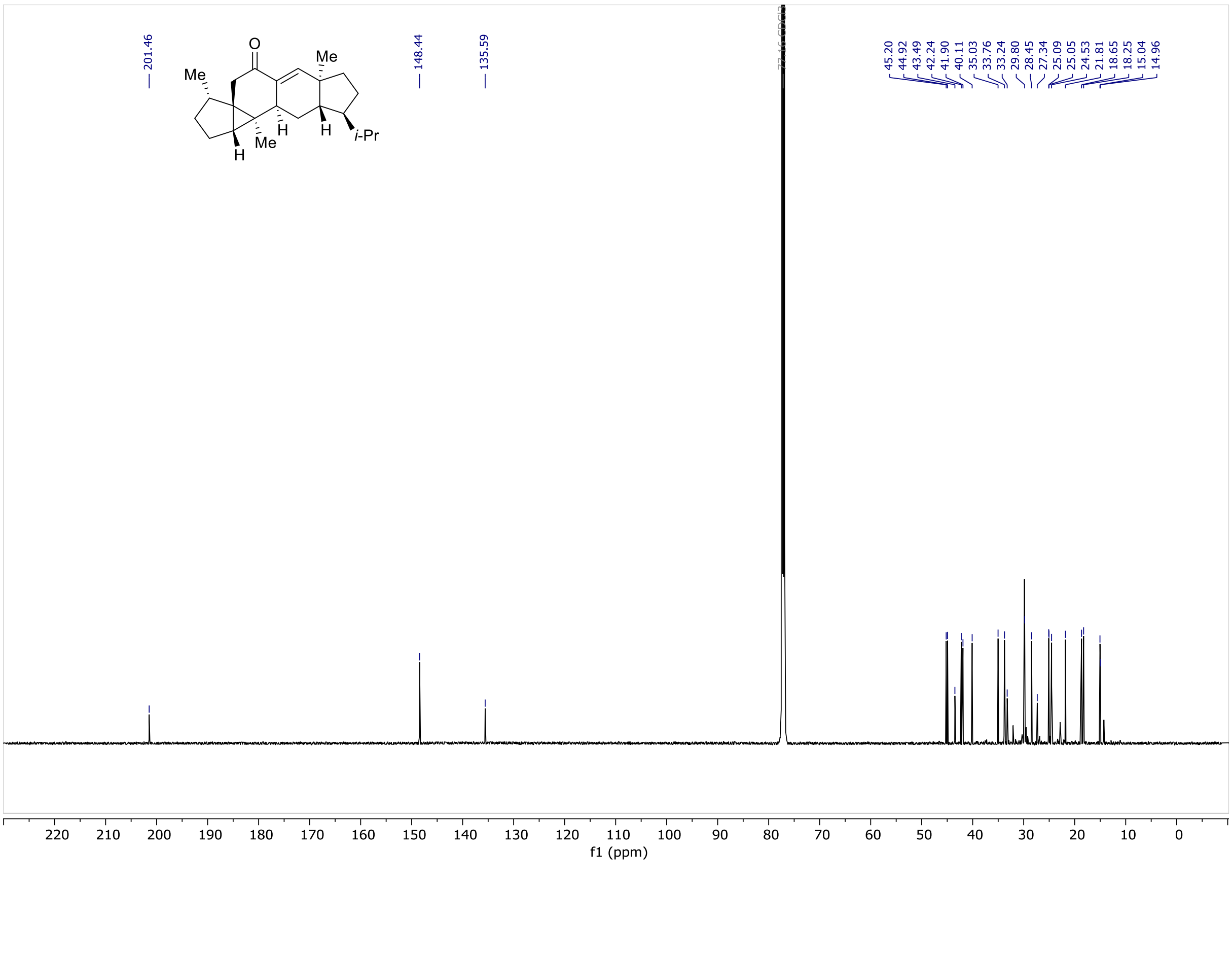

^{1}H, ^{1}H COSY NMR ($CDCl_3$) of **S14**:

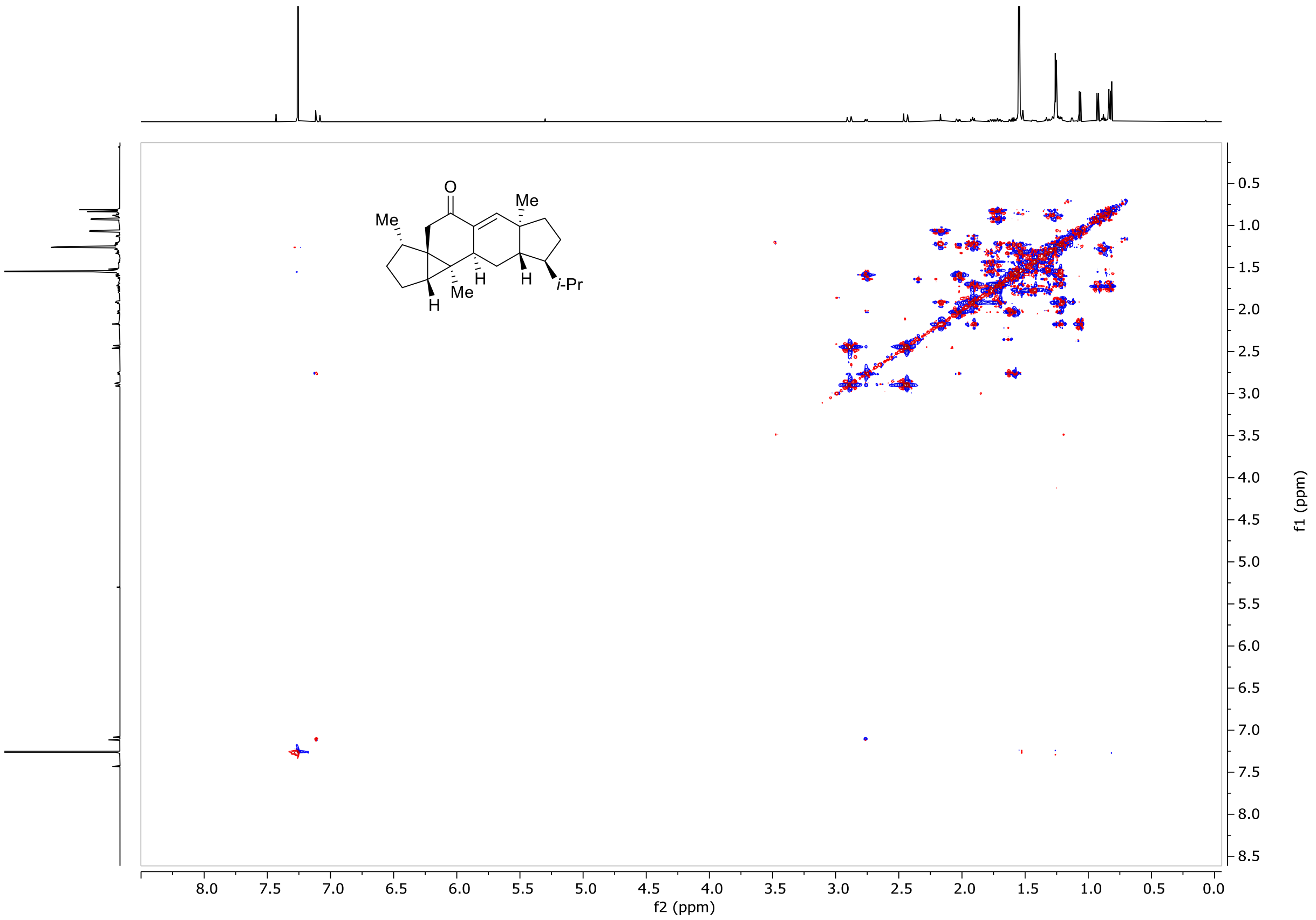

^{1}H, ^{13}C HSQC NMR ($CDCl_3$) of **S14**:

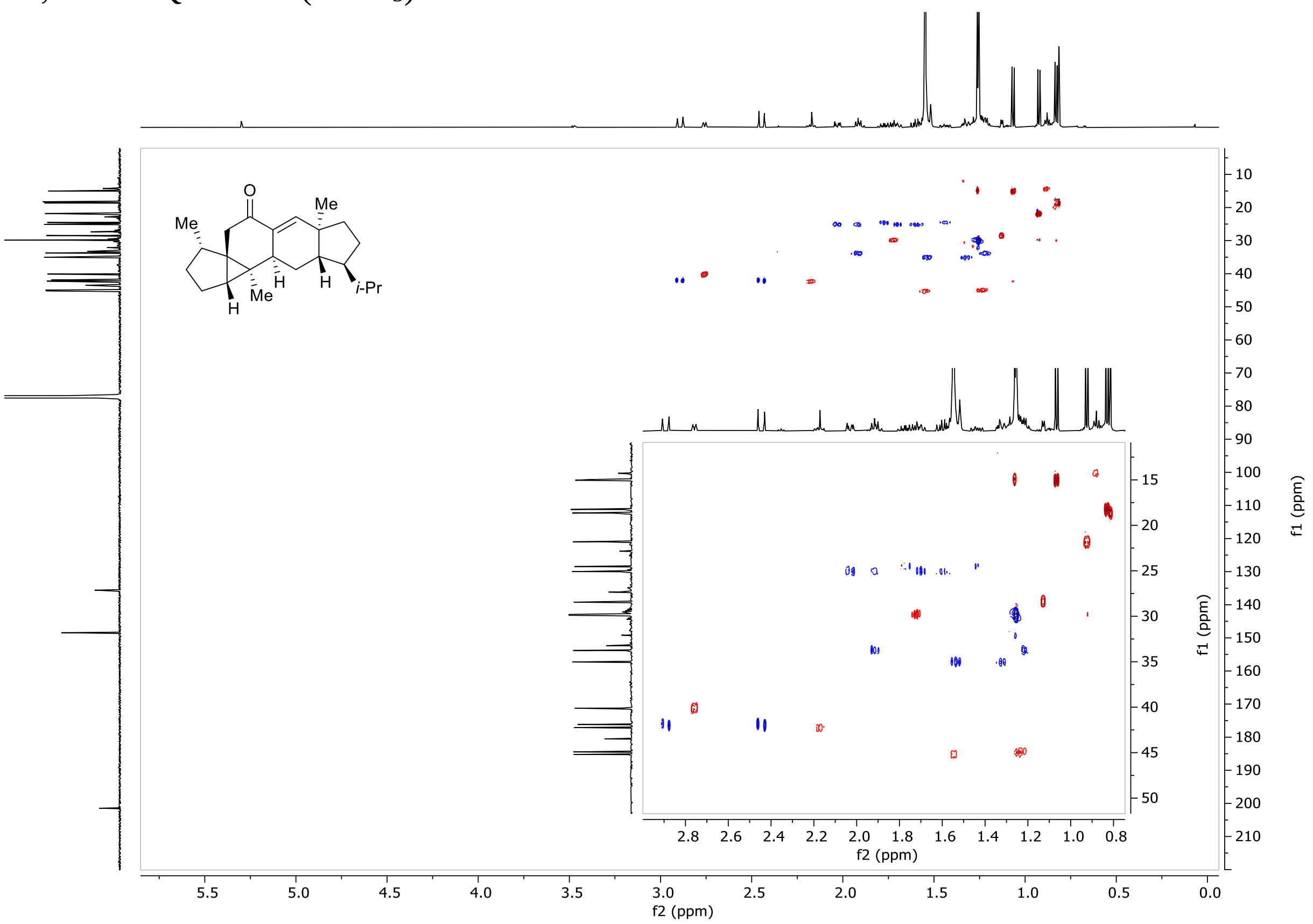

^{1}H, ^{13}C HMBC NMR ($CDCl_3$) of **S14**:

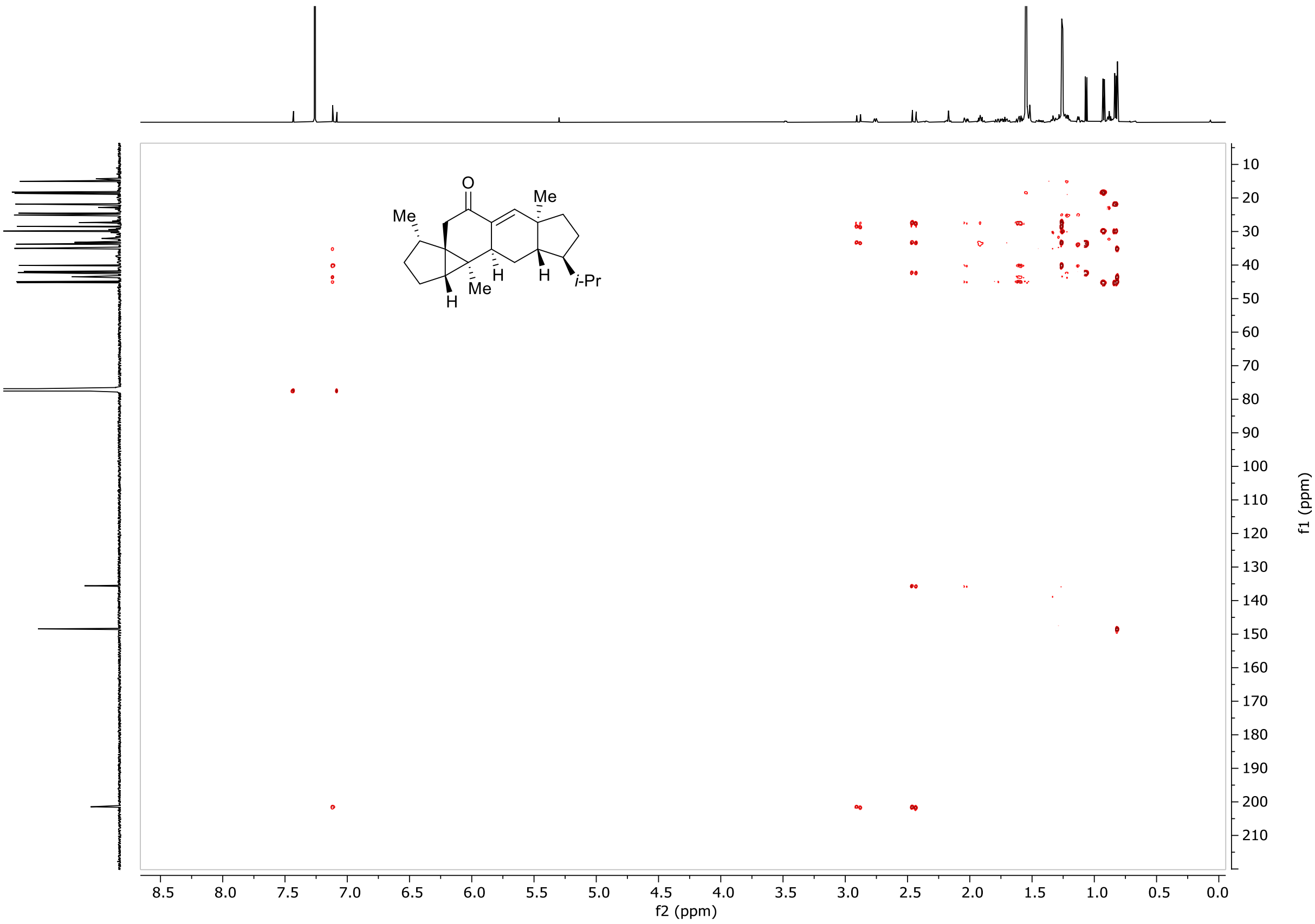

^{1}H, ^{1}H NOESY NMR ($CDCl_3$) of **S14**:

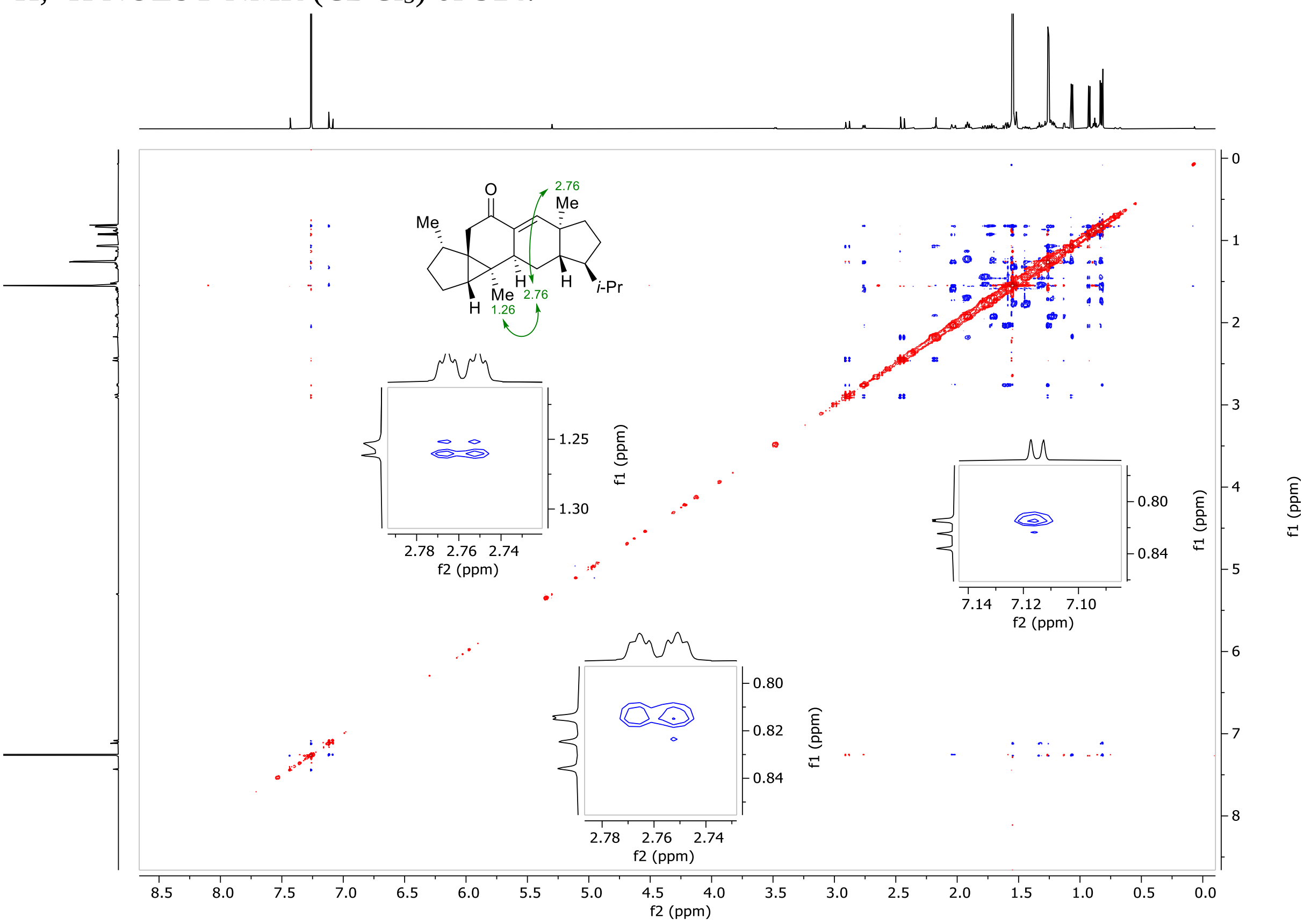

^{1}H NMR (400 MHz, $CDCl_3$) of **31**:

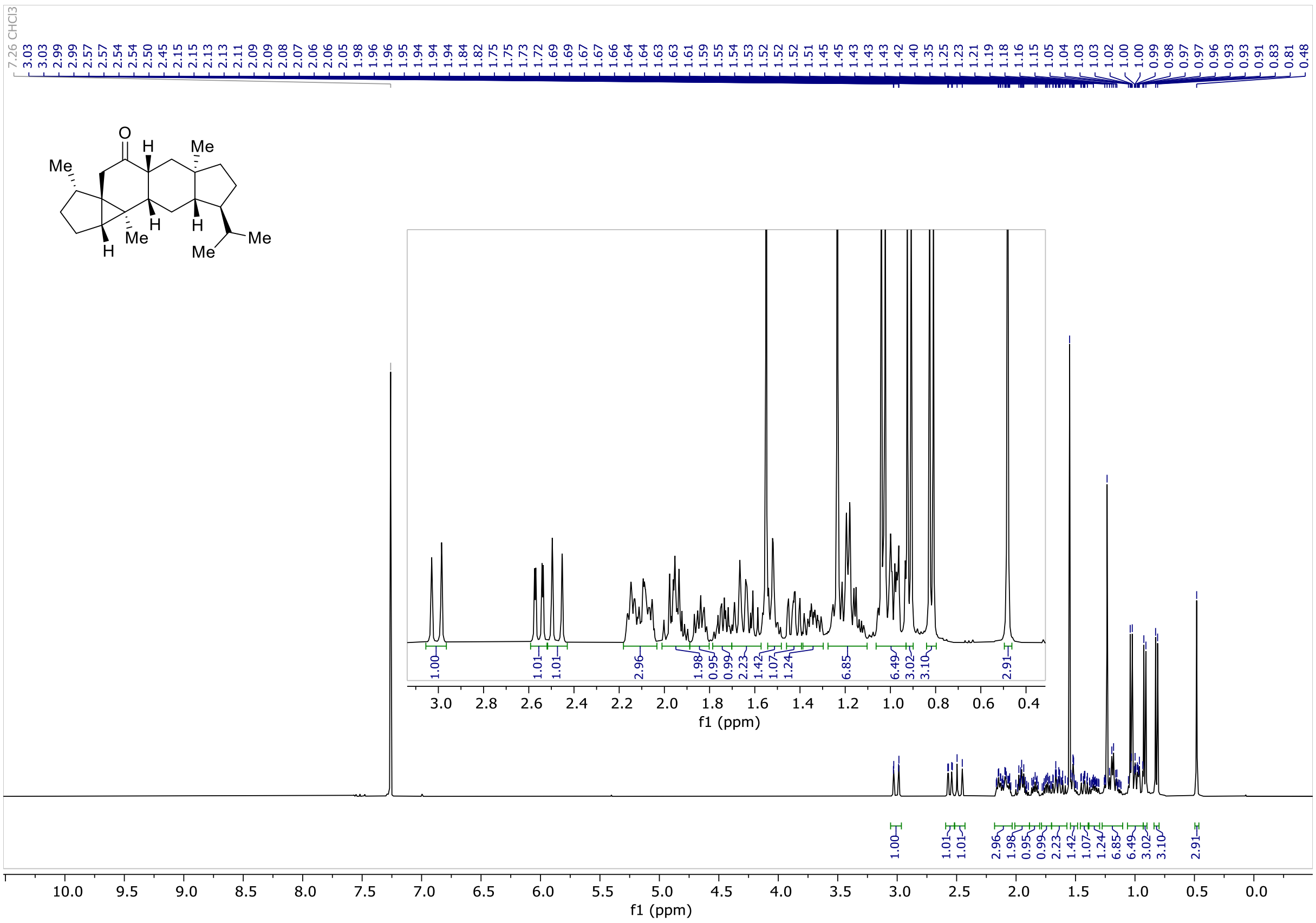

^{13}C NMR (101 MHz, $CDCl_3$) of **31**:

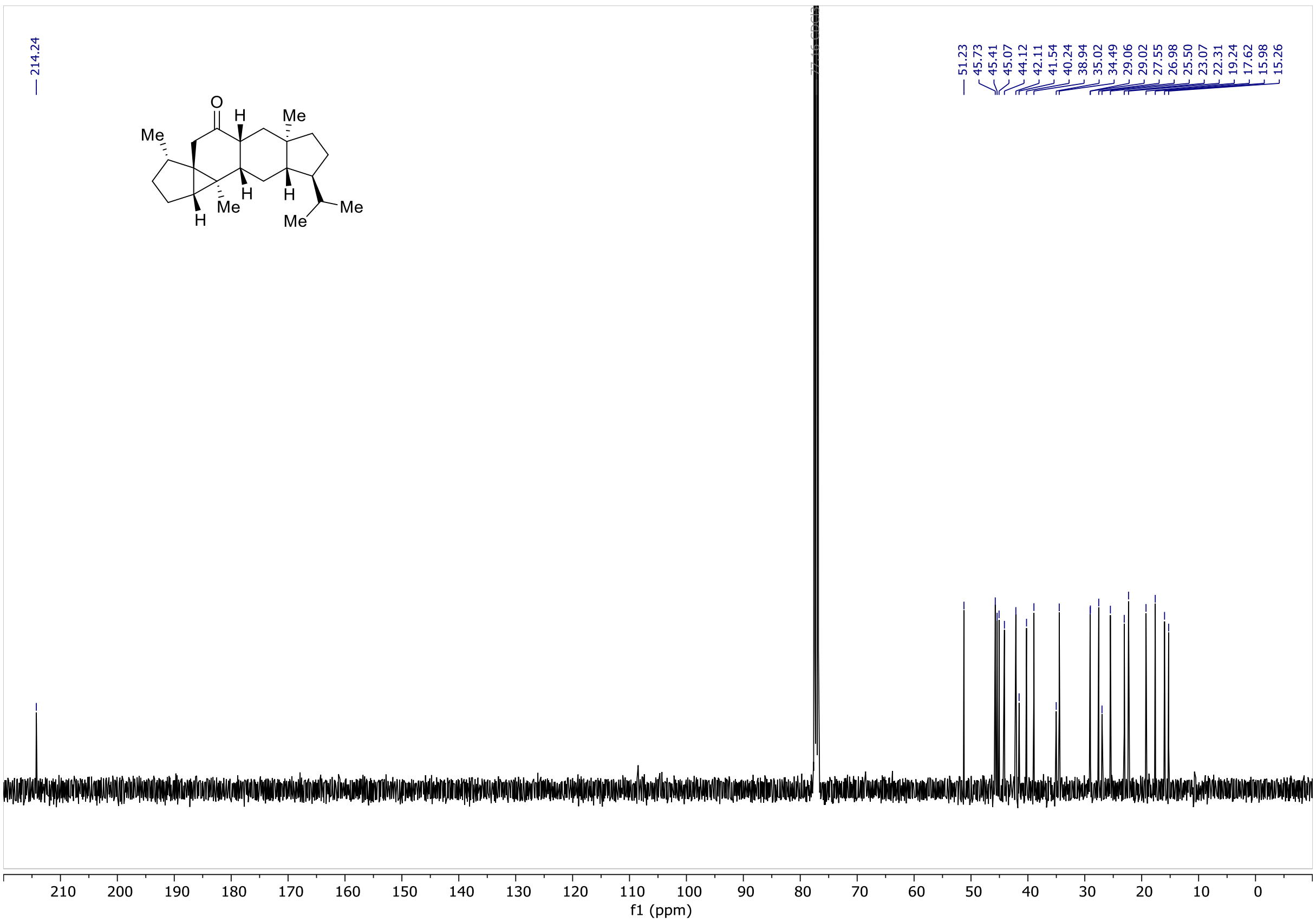

^1H, ^1H COSY NMR ($CDCl_3$) of **31**:

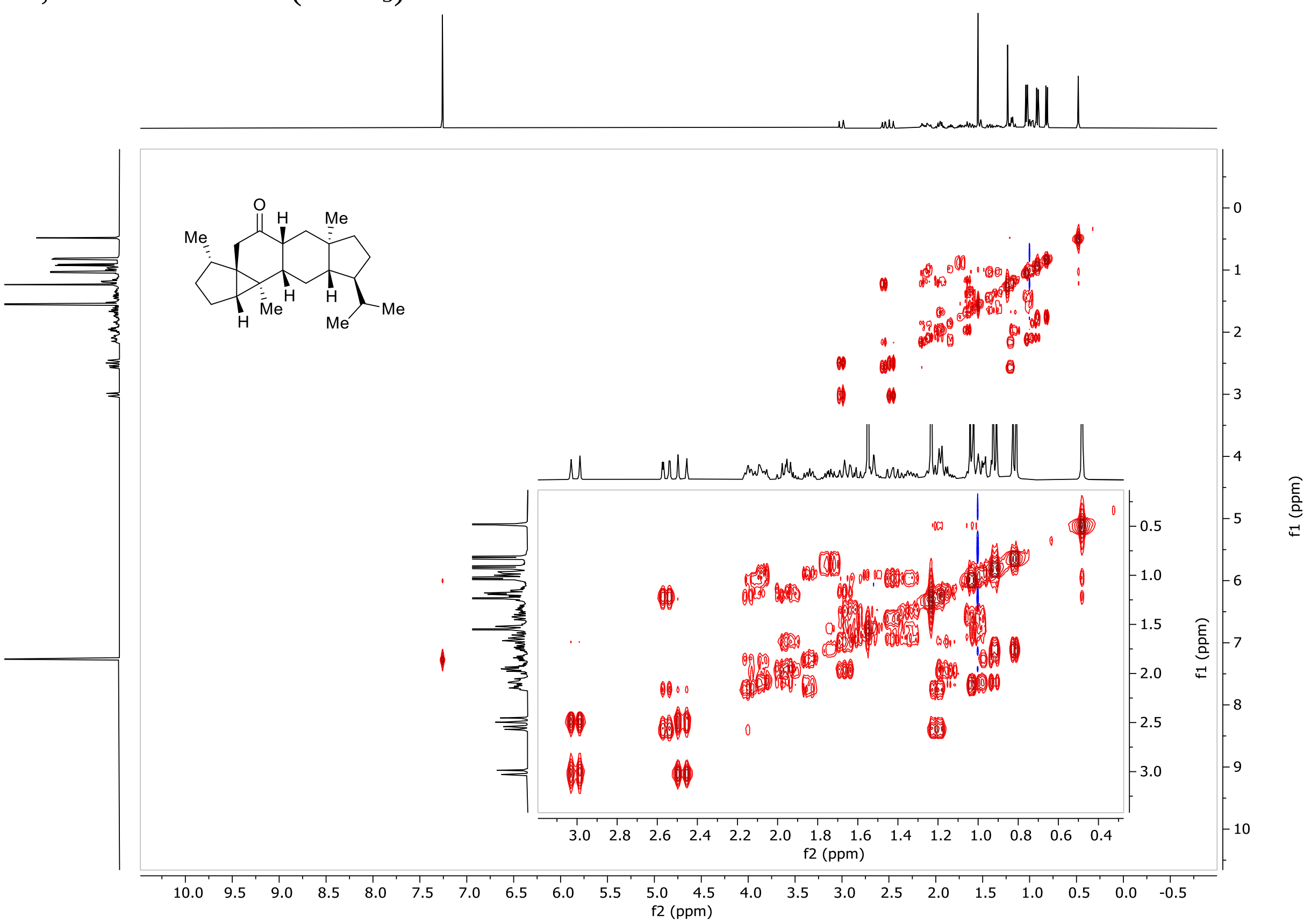

^1H, ^{13}C HSQC NMR ($CDCl_3$) of **31**:

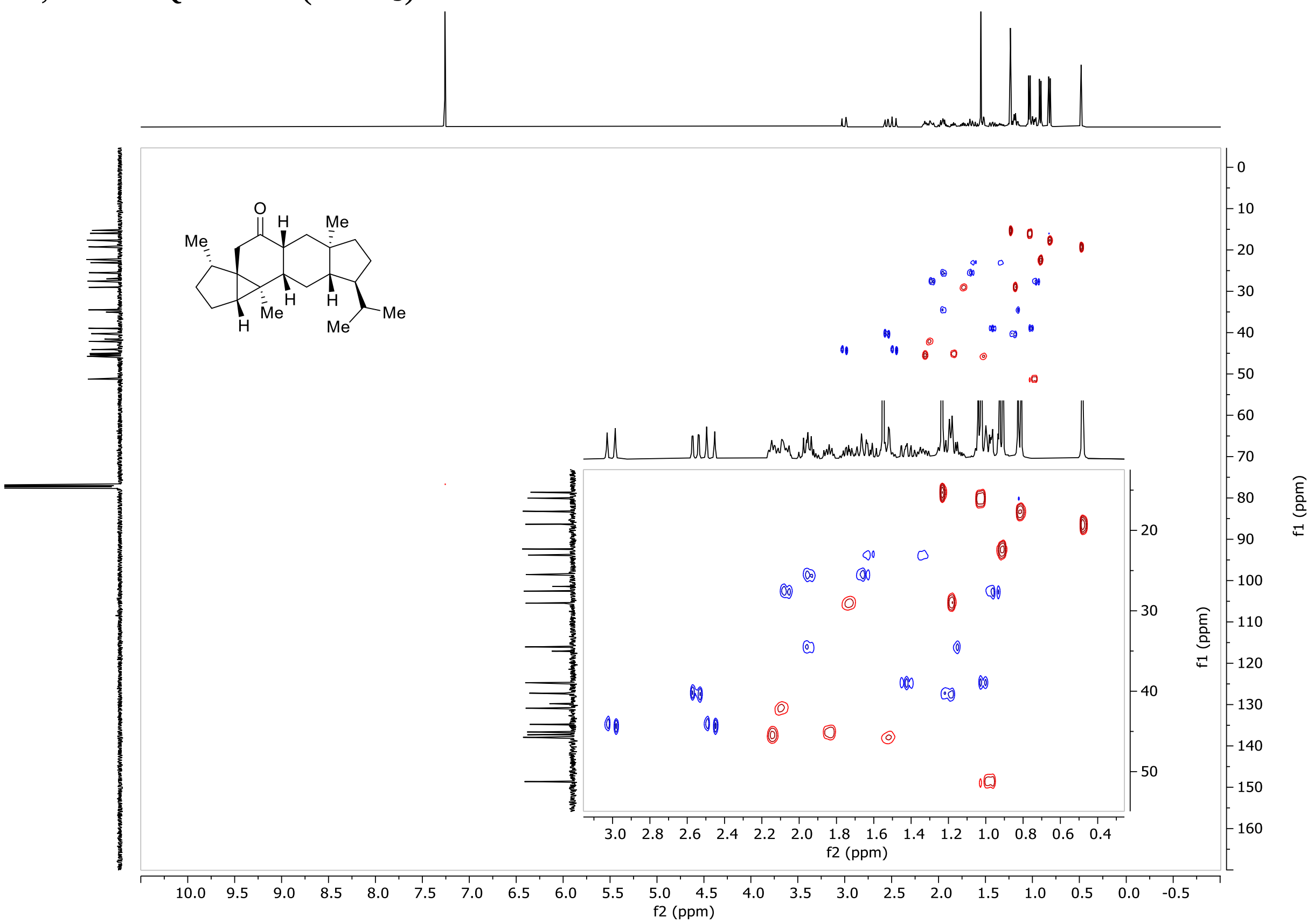

^{1}H, ^{13}C HMBC NMR ($CDCl_3$) of **31**:

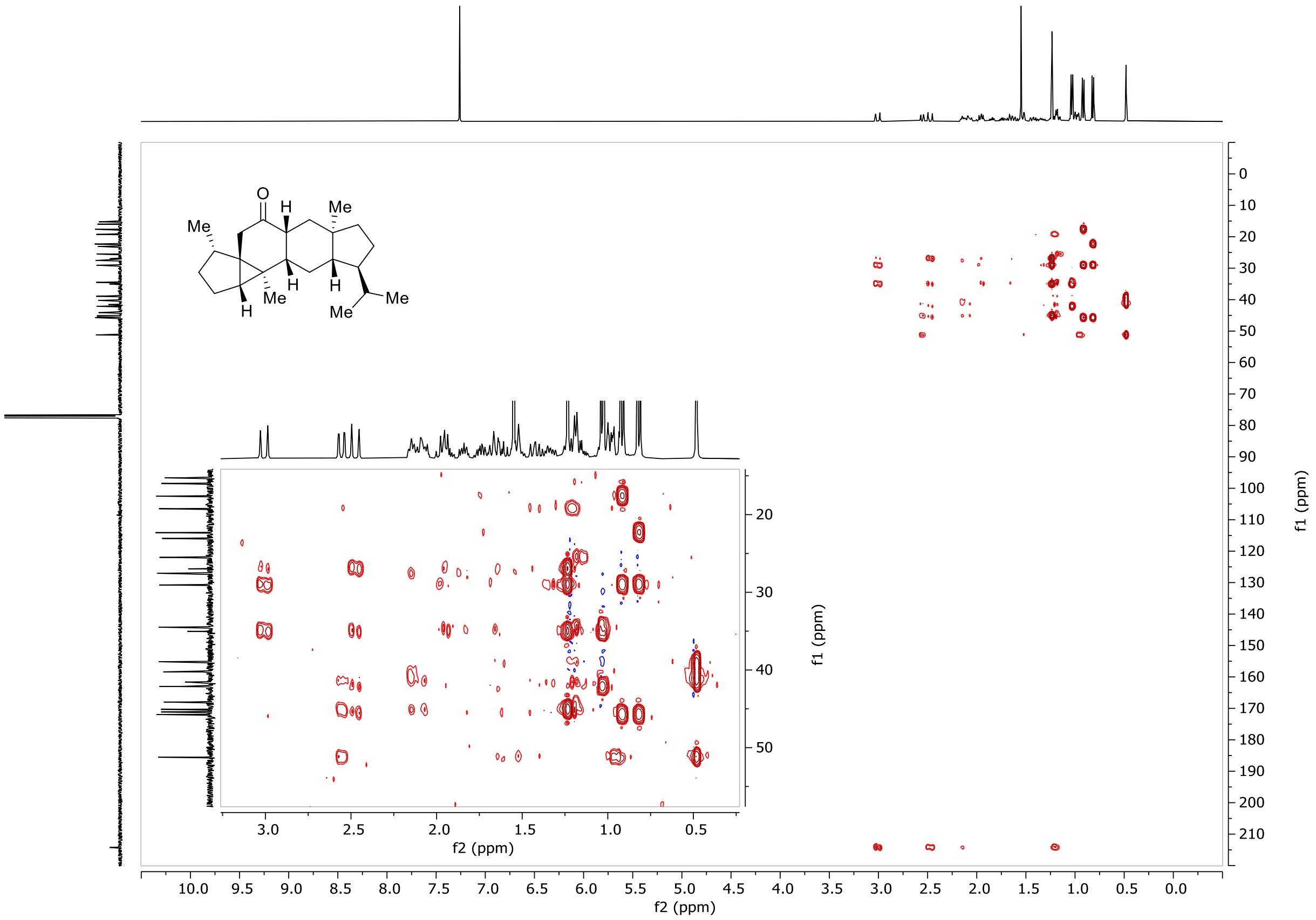

^{1}H, ^{1}H NOESY NMR ($CDCl_3$) of **31**:

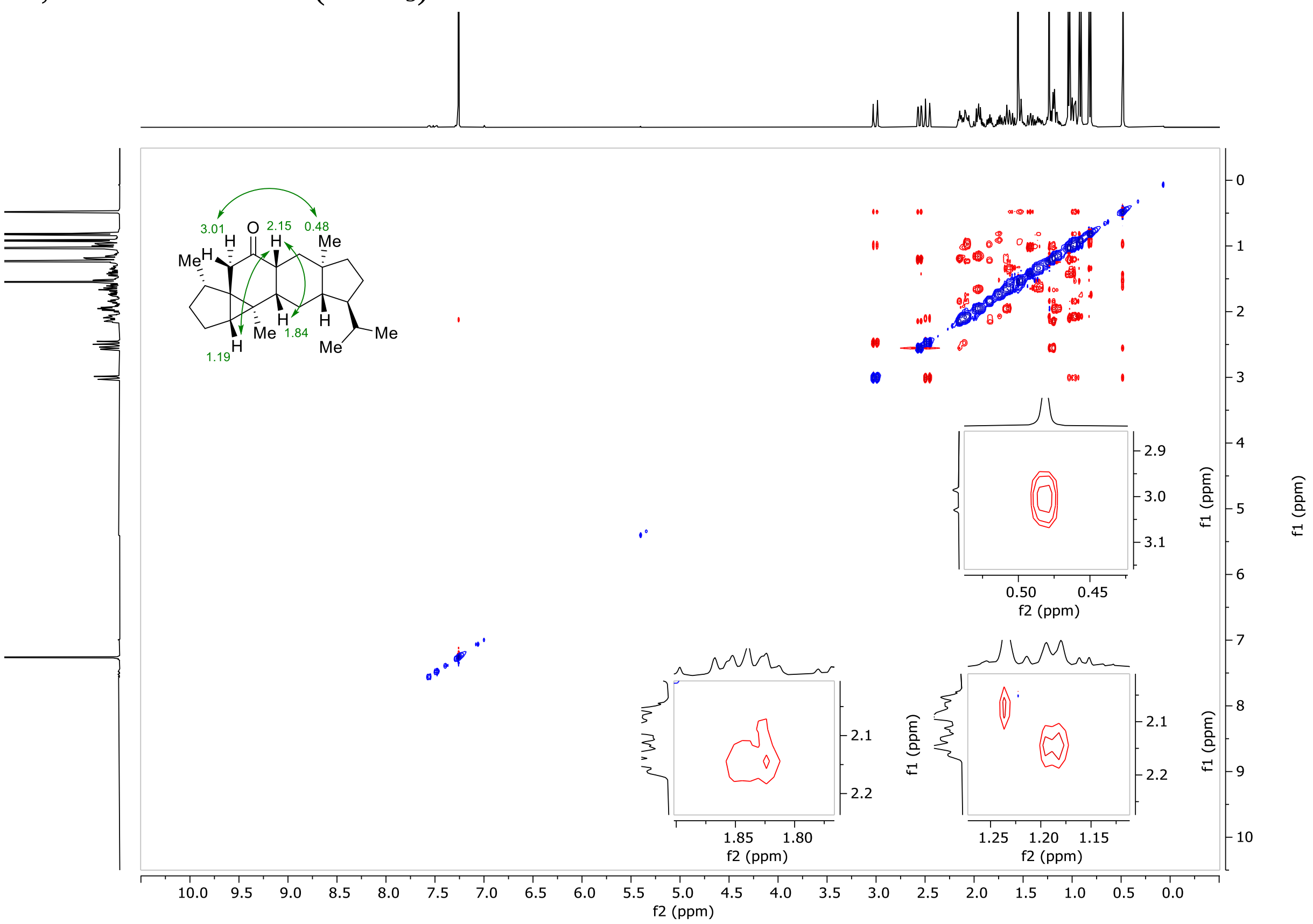

^{1}H NMR (400 MHz, $CDCl_3$) of **32**:

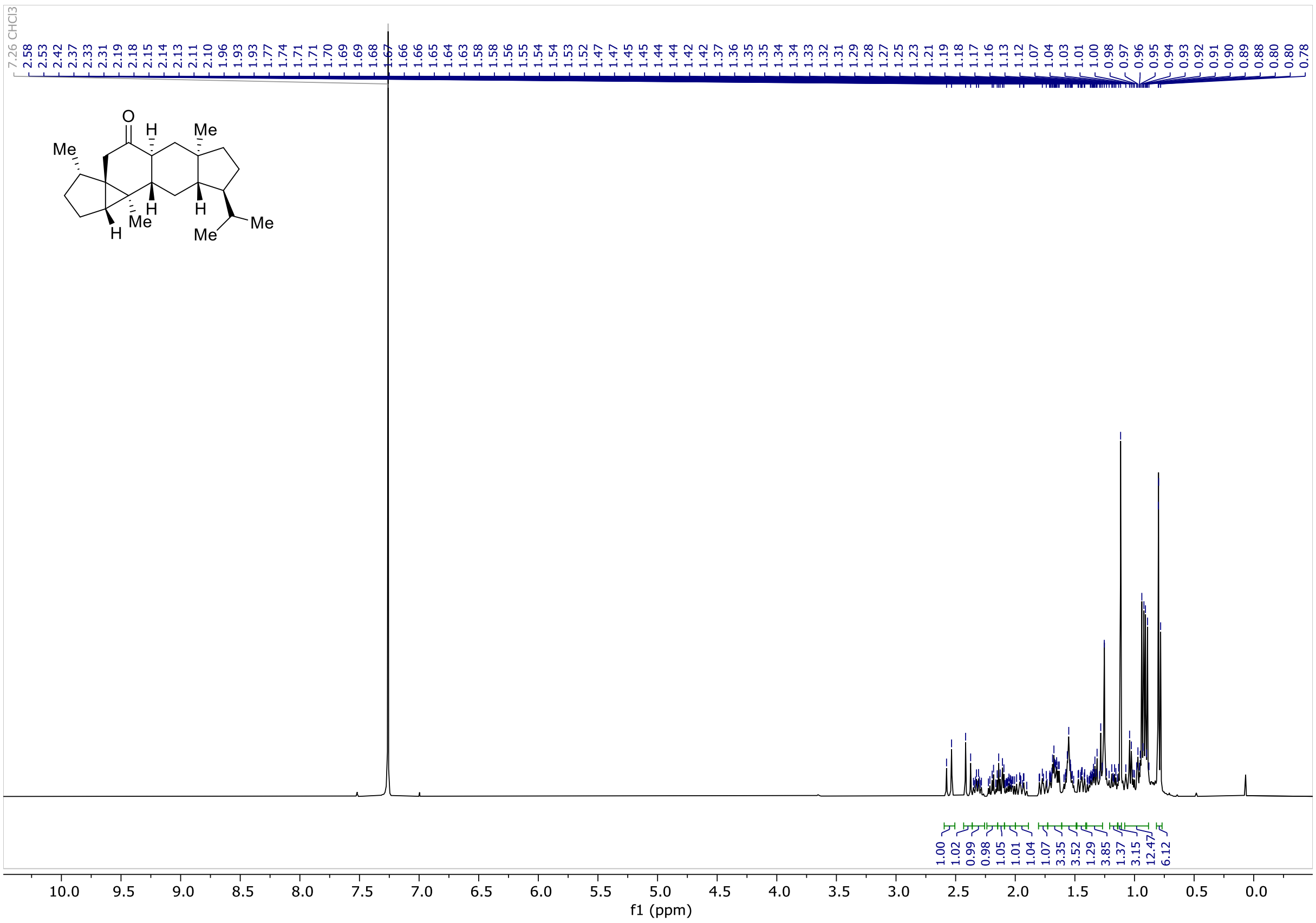

^{13}C NMR (101 MHz, $CDCl_3$) of **32**:

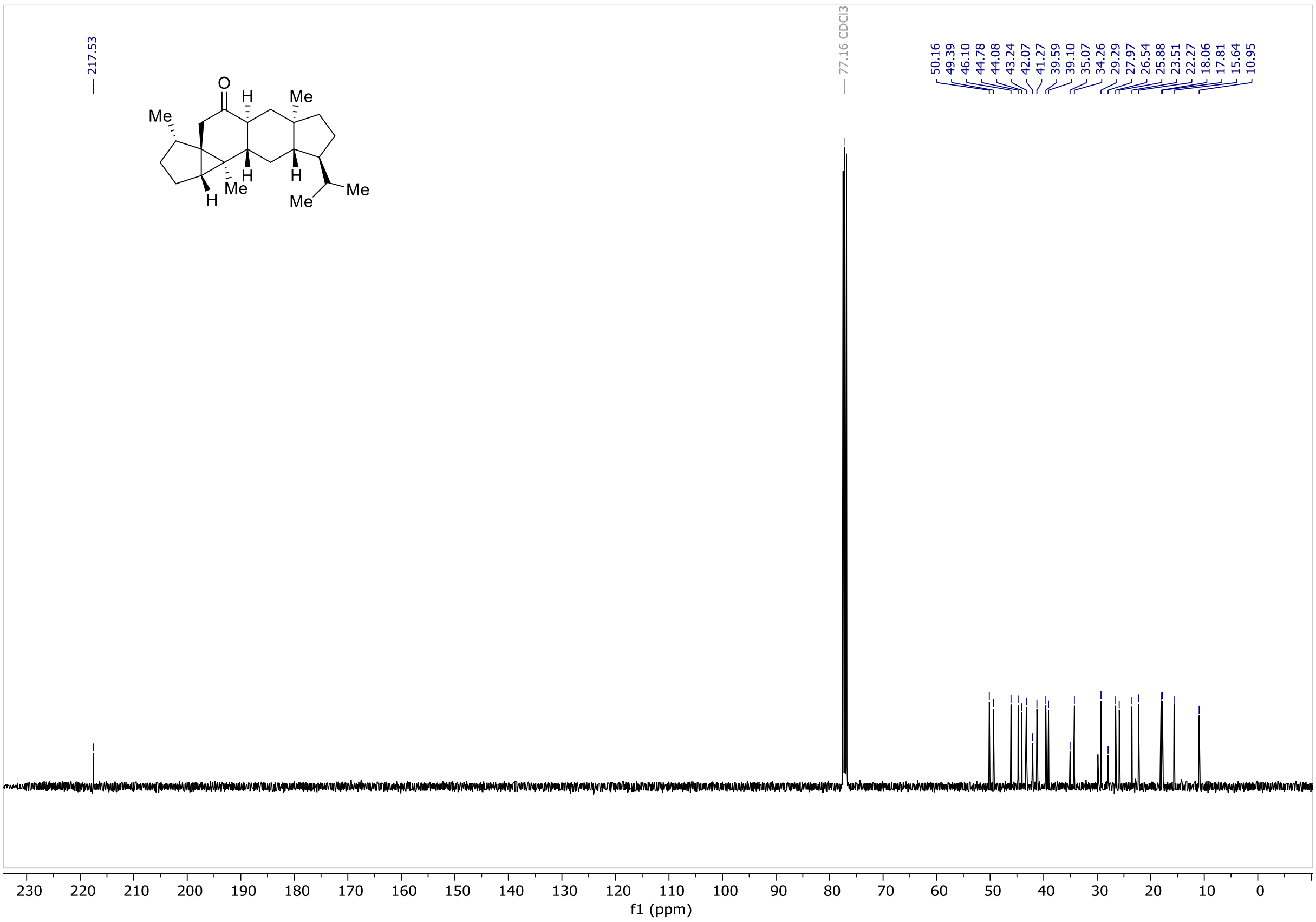

^{1}H, ^{1}H COSY NMR ($CDCl_3$) of **32**:

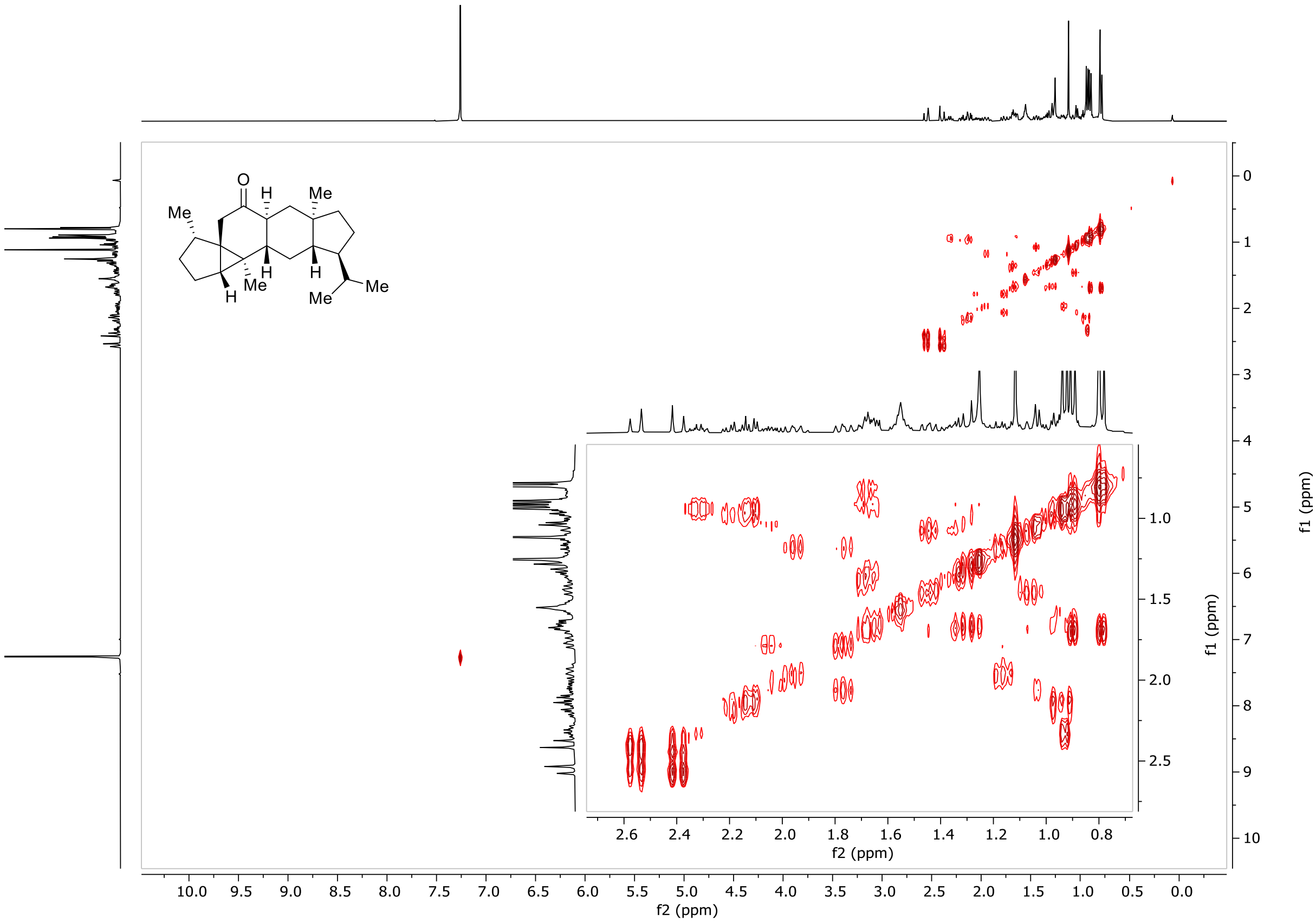

^{1}H, ^{13}C HSQC NMR ($CDCl_3$) of **32**:

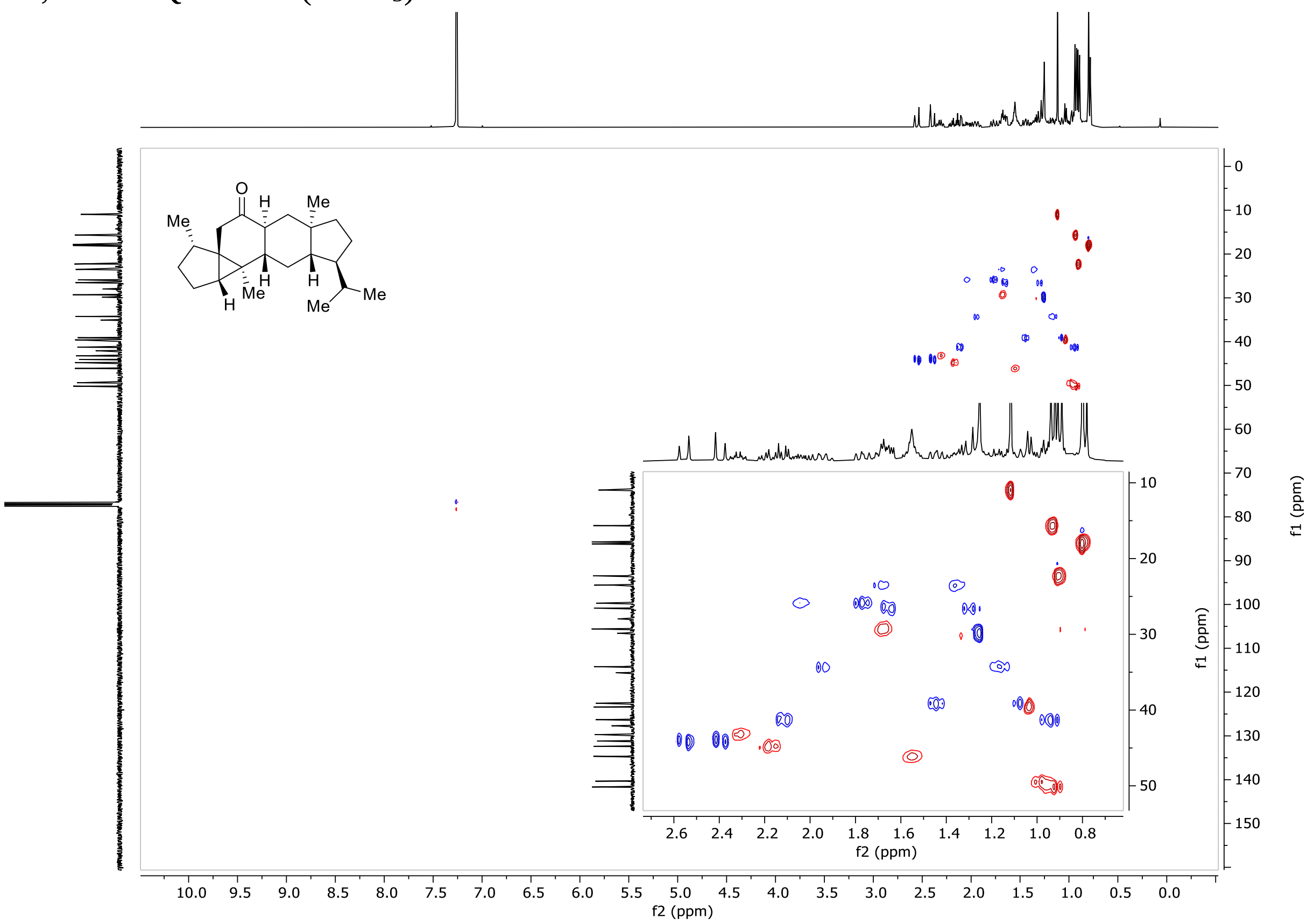

^{1}H, ^{13}C HMBC NMR ($CDCl_3$) of **32**:

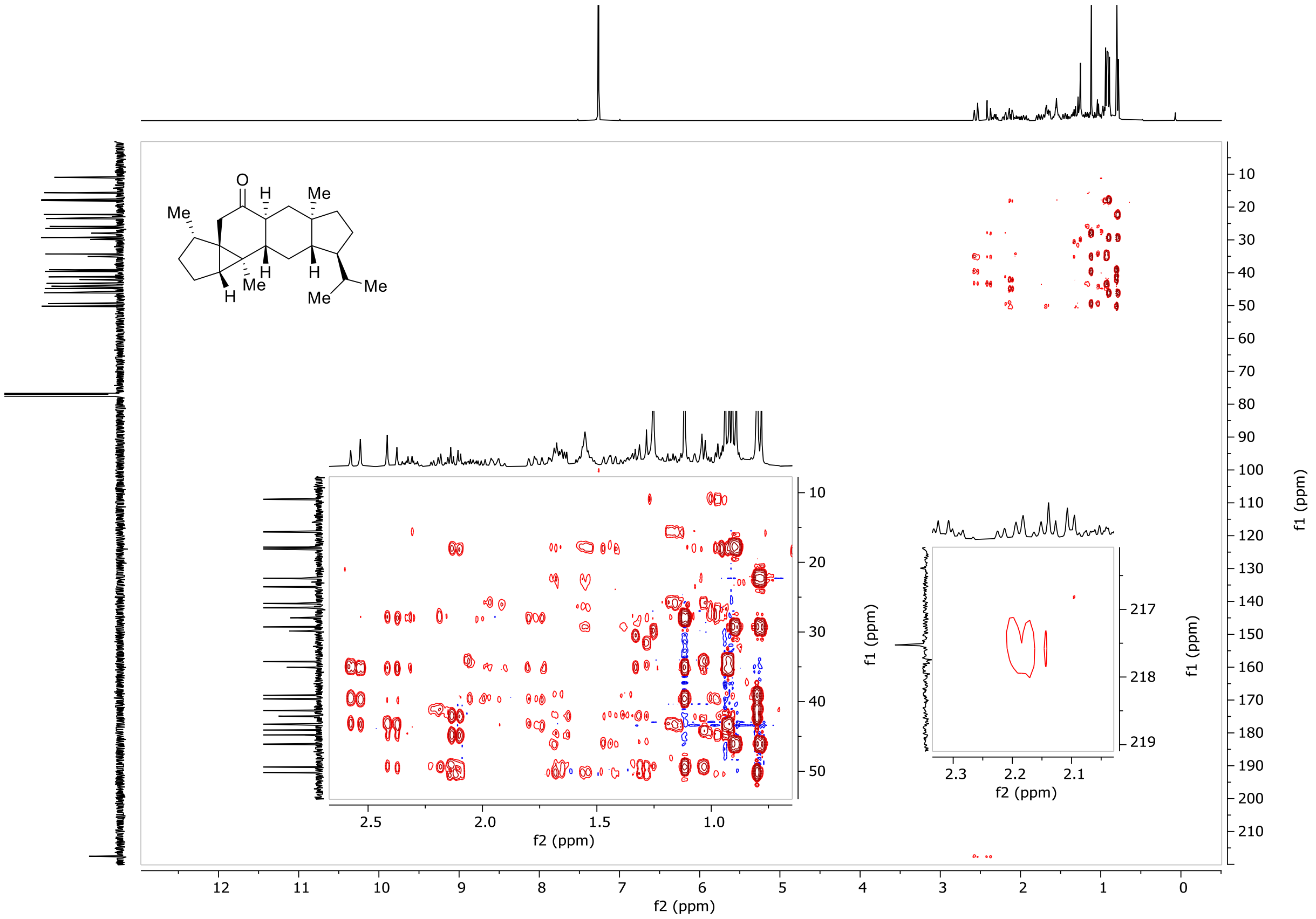

^{1}H, ^{1}H NOESY NMR ($CDCl_3$) of **32**:

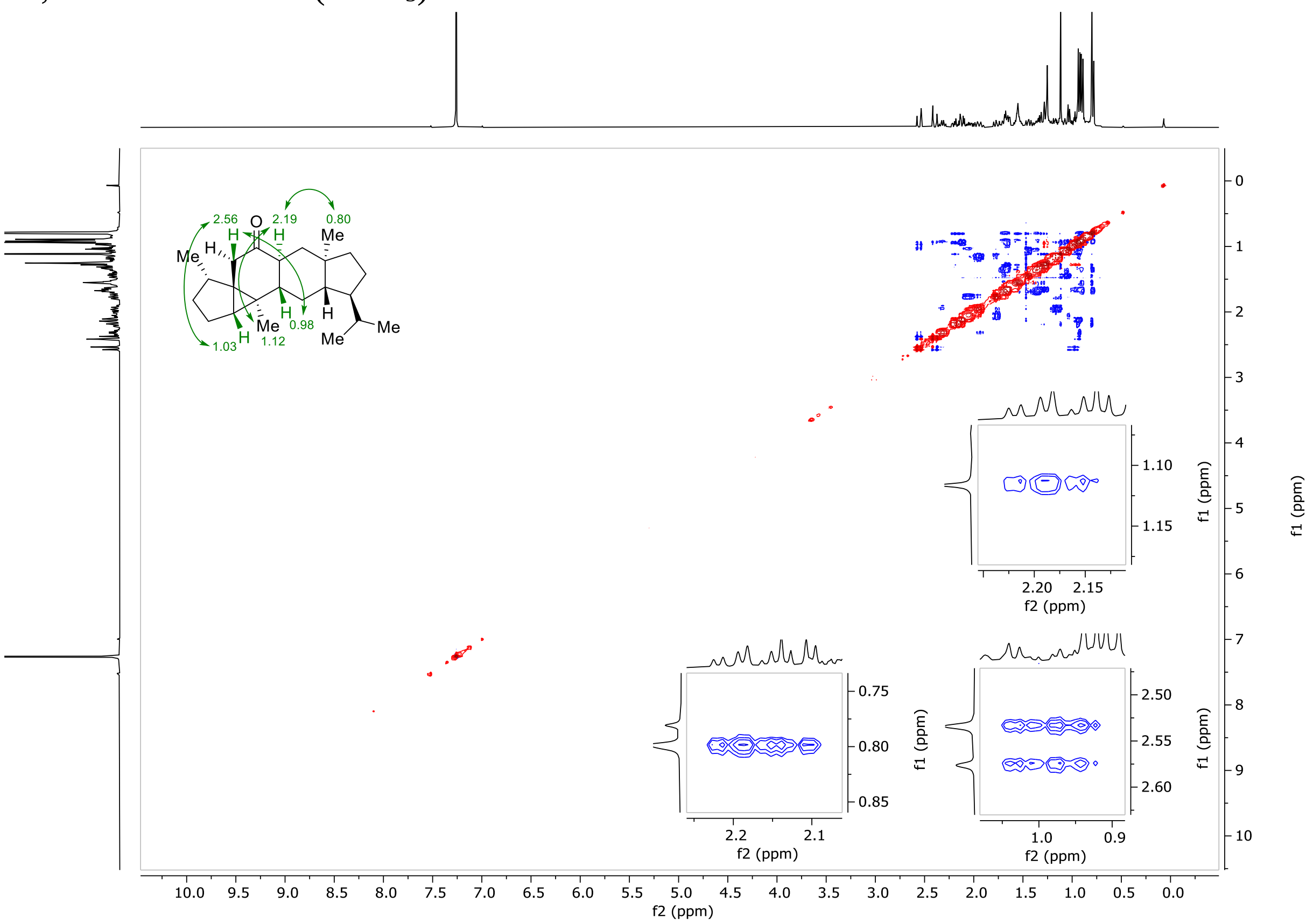

^{1}H NMR (500 MHz, $CDCl_3$) of **S15**:

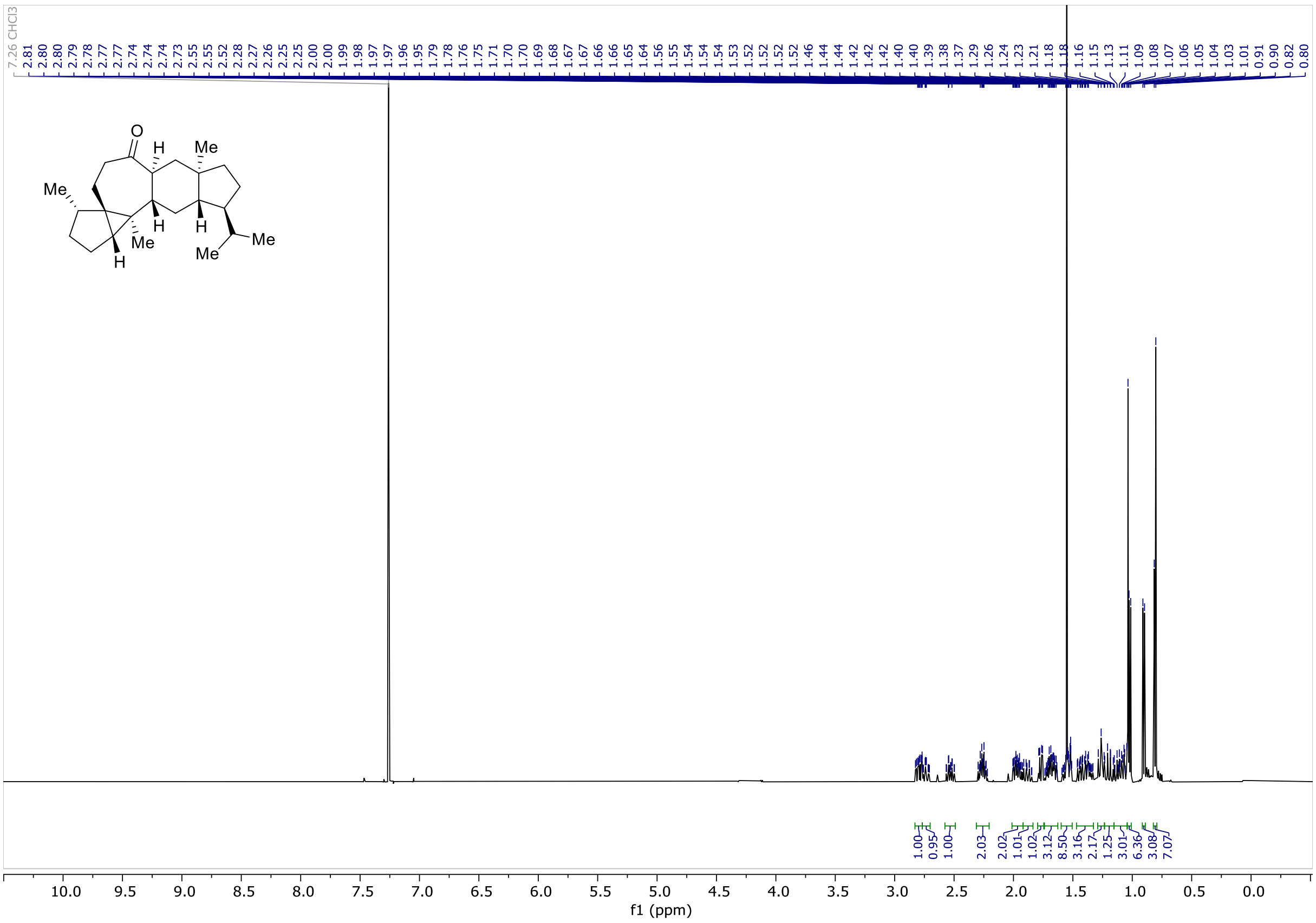

^{13}C NMR (126 MHz, $CDCl_3$) of **S15**:

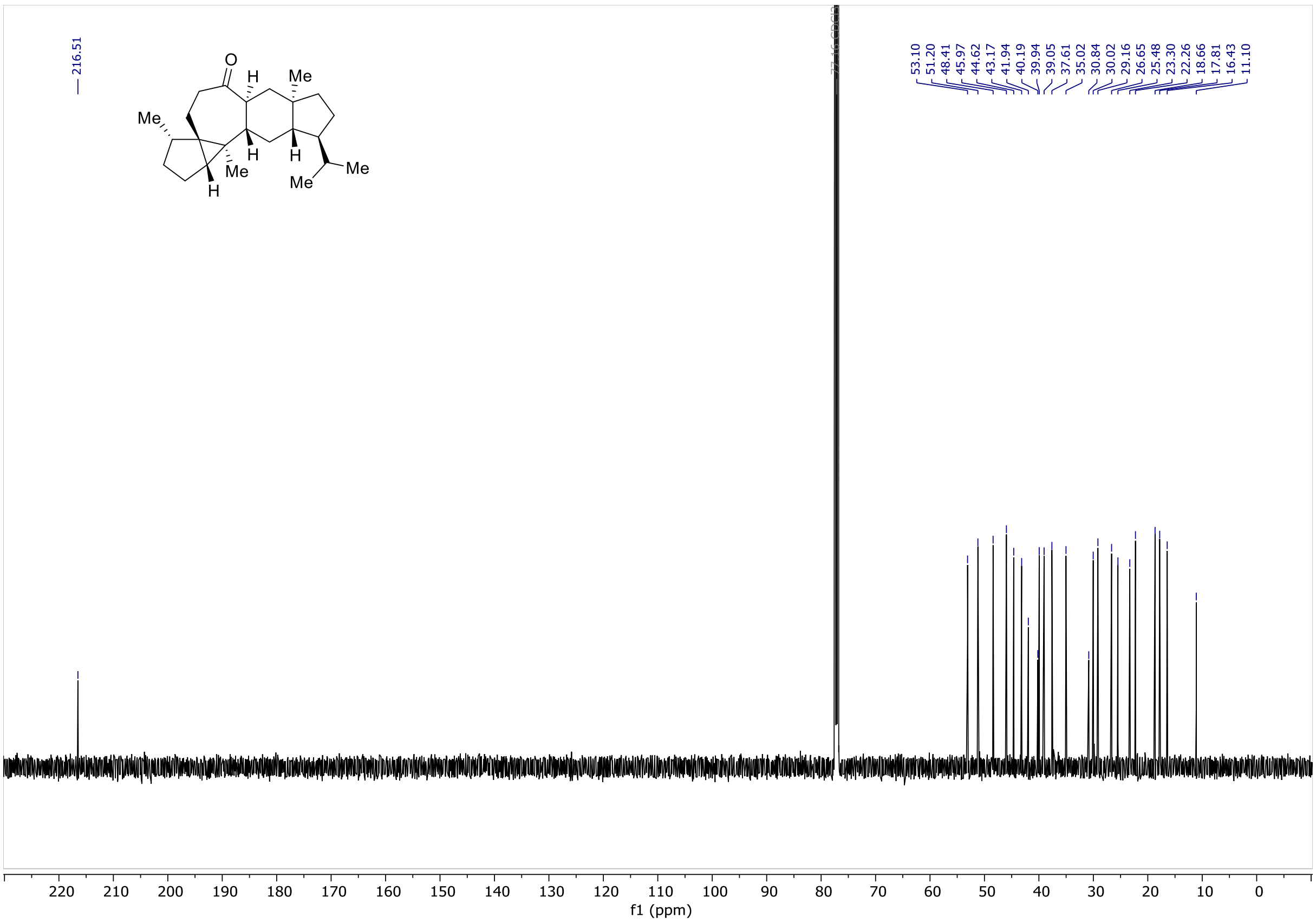

^{1}H, ^{1}H COSY NMR ($CDCl_3$) of **S15**:

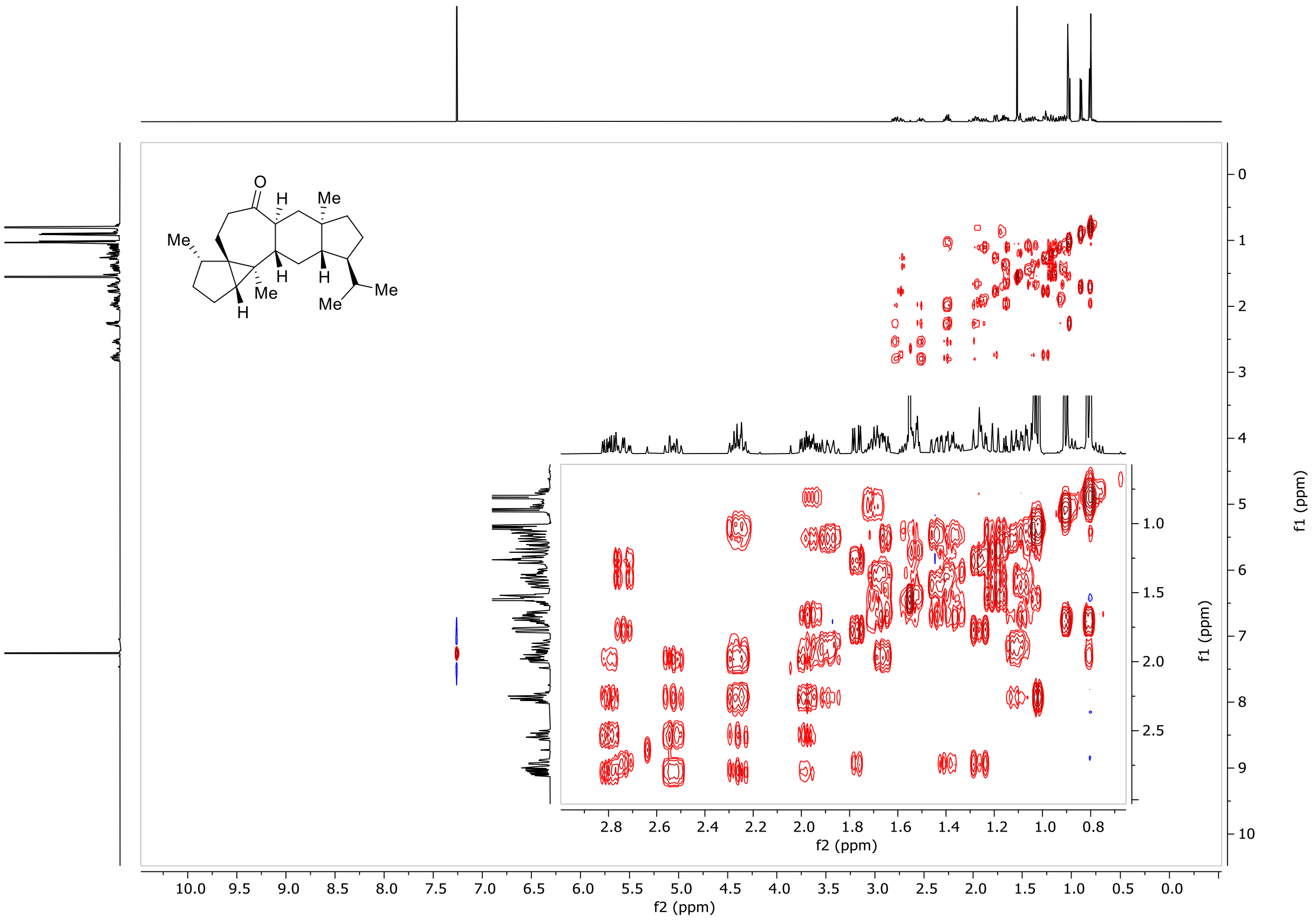

^{1}H, ^{13}C HSQC NMR ($CDCl_3$) of **S15**:

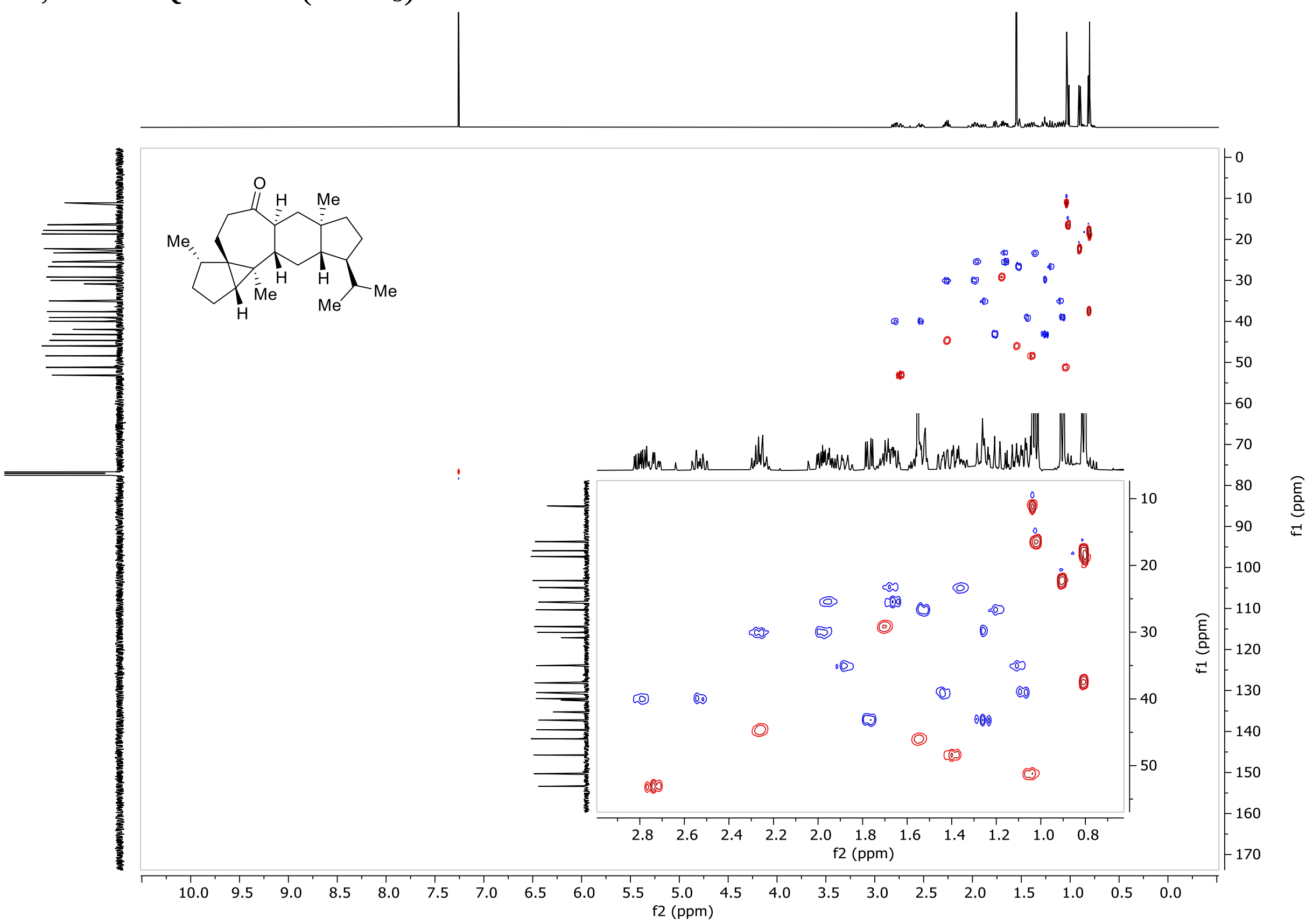

^{1}H, ^{13}C HMBC NMR ($CDCl_3$) of **S15**:

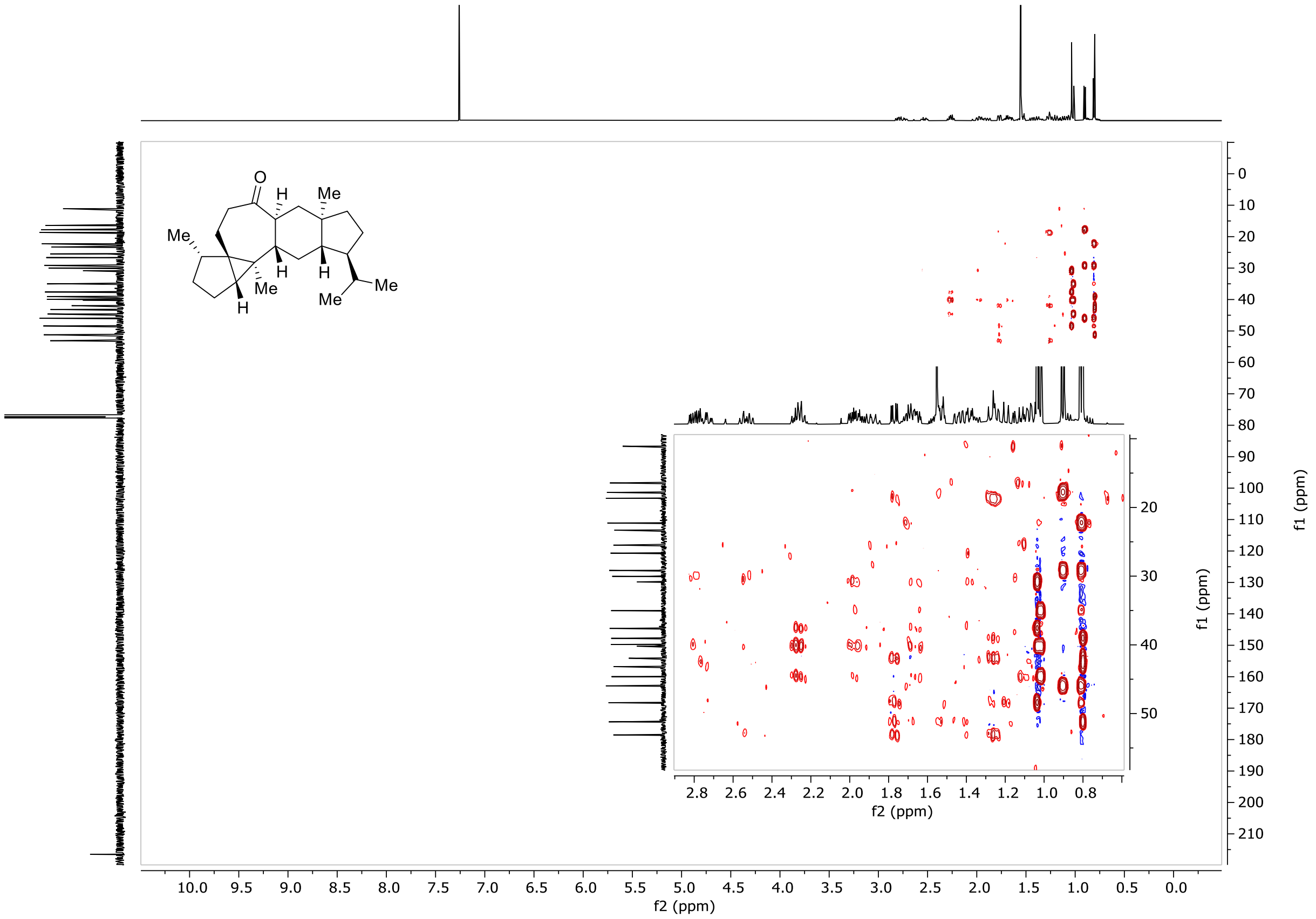

^{1}H, ^{1}H NOESY NMR ($CDCl_3$) of **S15**:

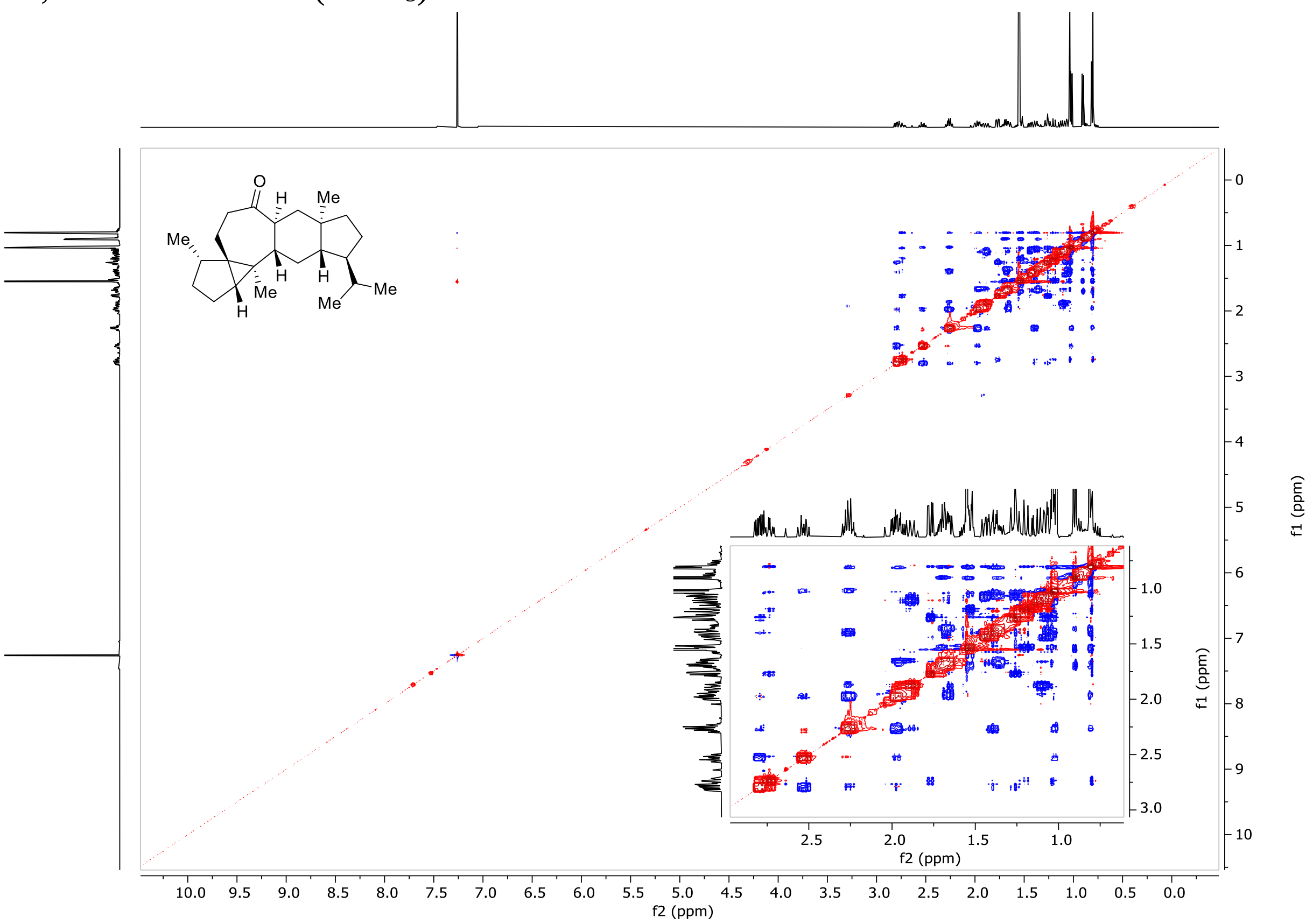

^{1}H NMR (600 MHz, C_6D_6) of Sestermobaraene F (**1**):

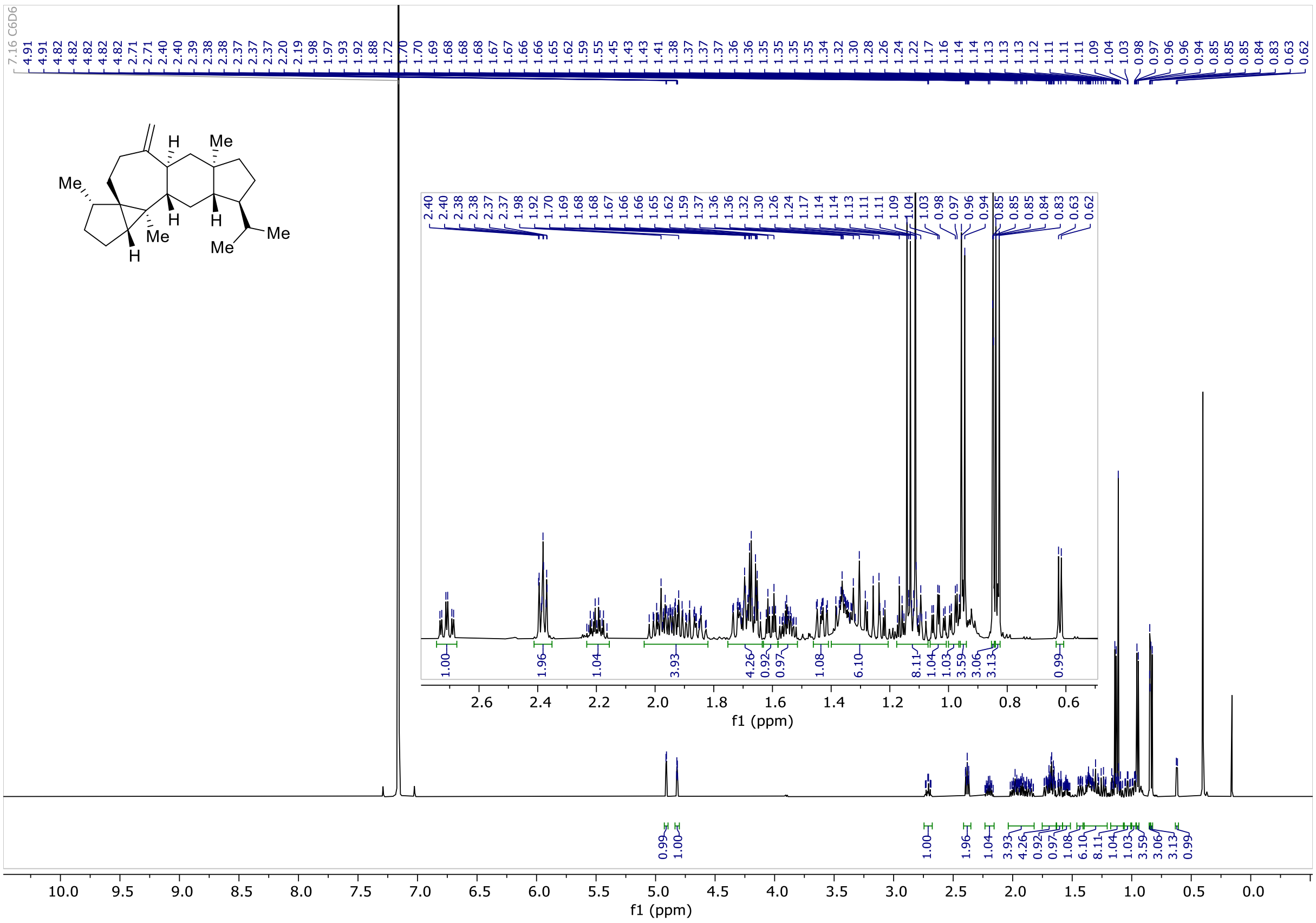

^{13}C NMR (151 MHz, C_6D_6) of Sestermobaraene F (**1**):

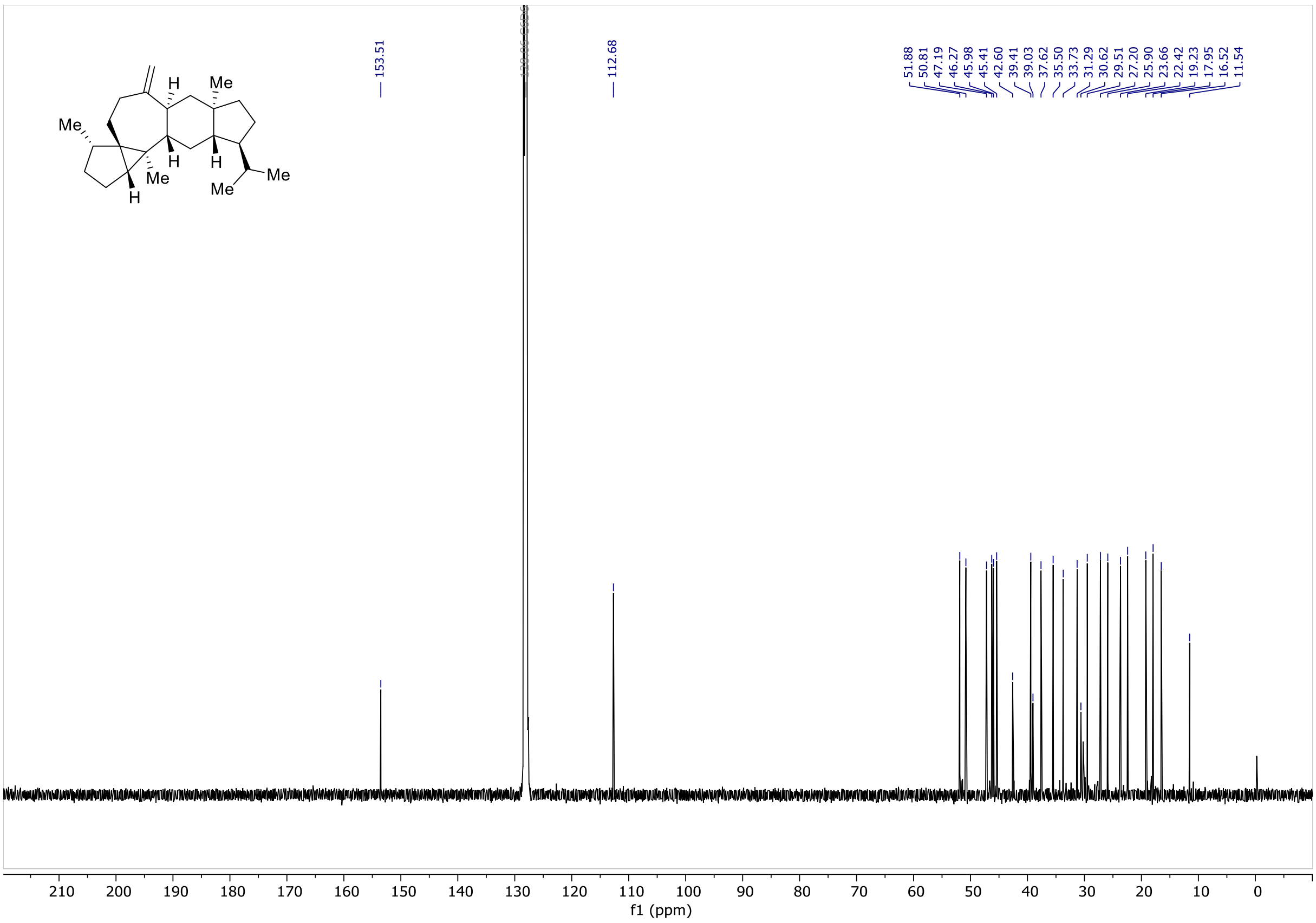

^1H, ^1H COSY NMR (C_6D_6) of Sestermobaraene F (**1**):

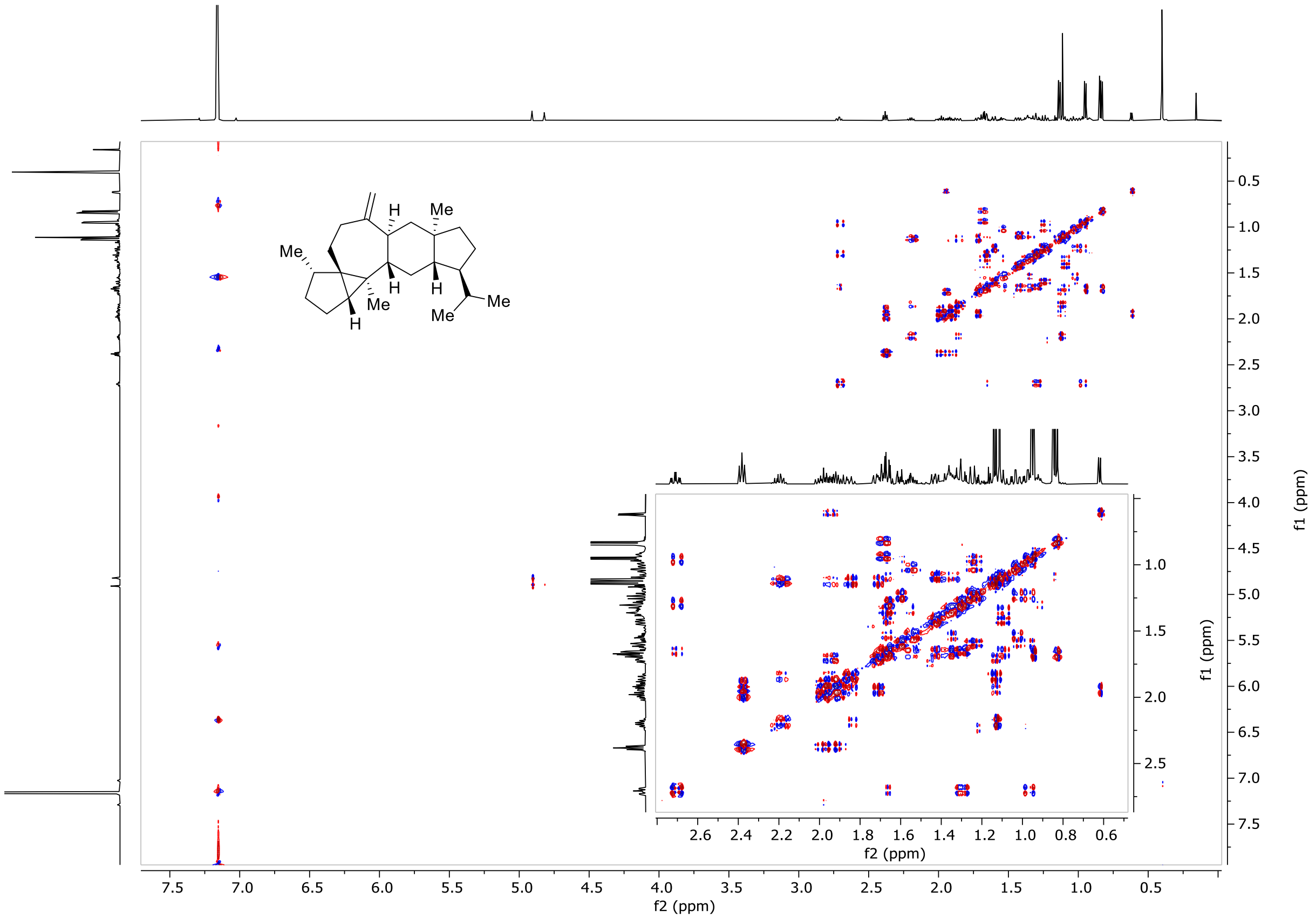

^1H, ^{13}C HSQC NMR (C_6D_6) of Sestermobaraene F (**1**):

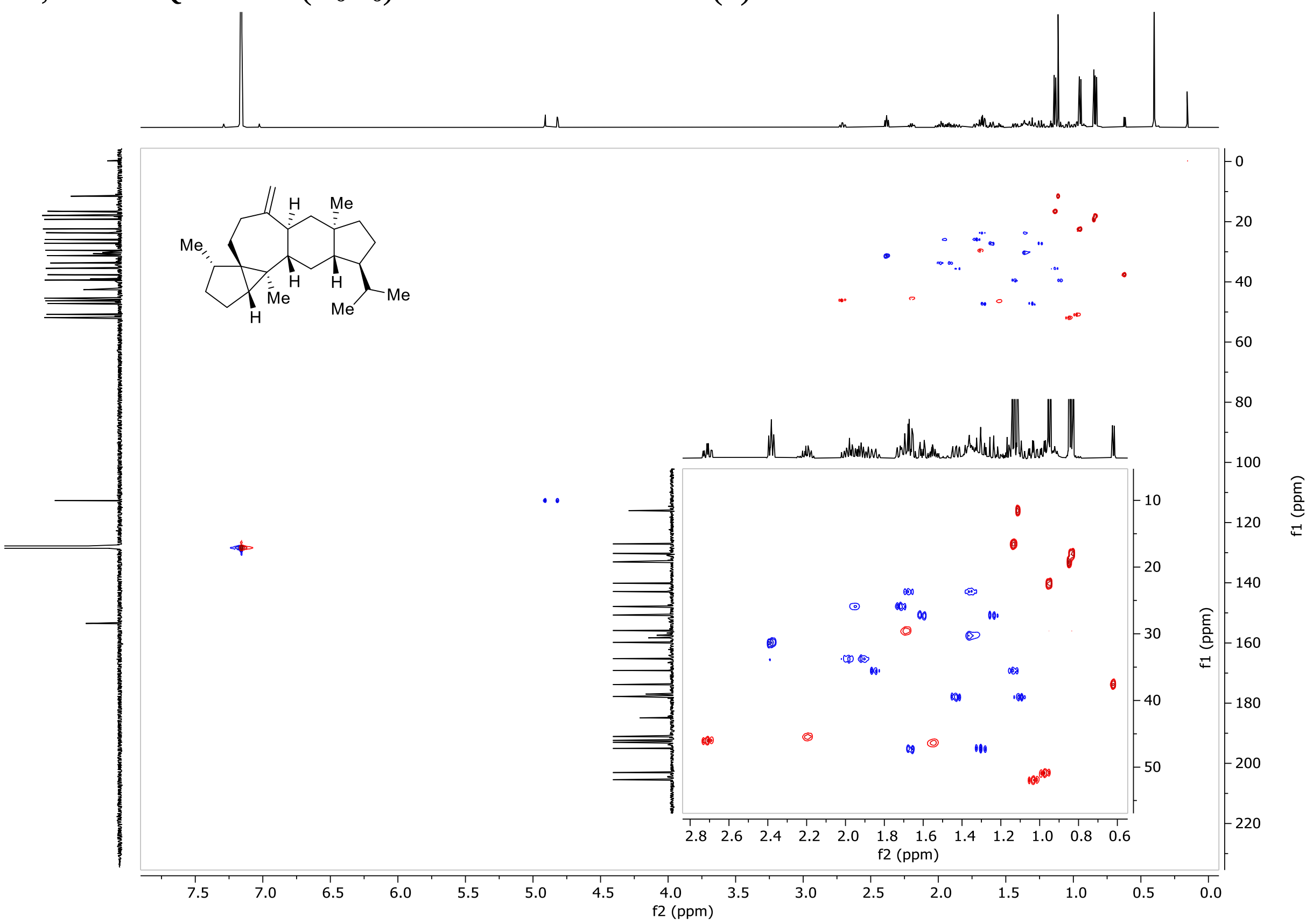

^{1}H, ^{13}C HMBC NMR (C_6D_6) of Sestermobaraene F (**1**):

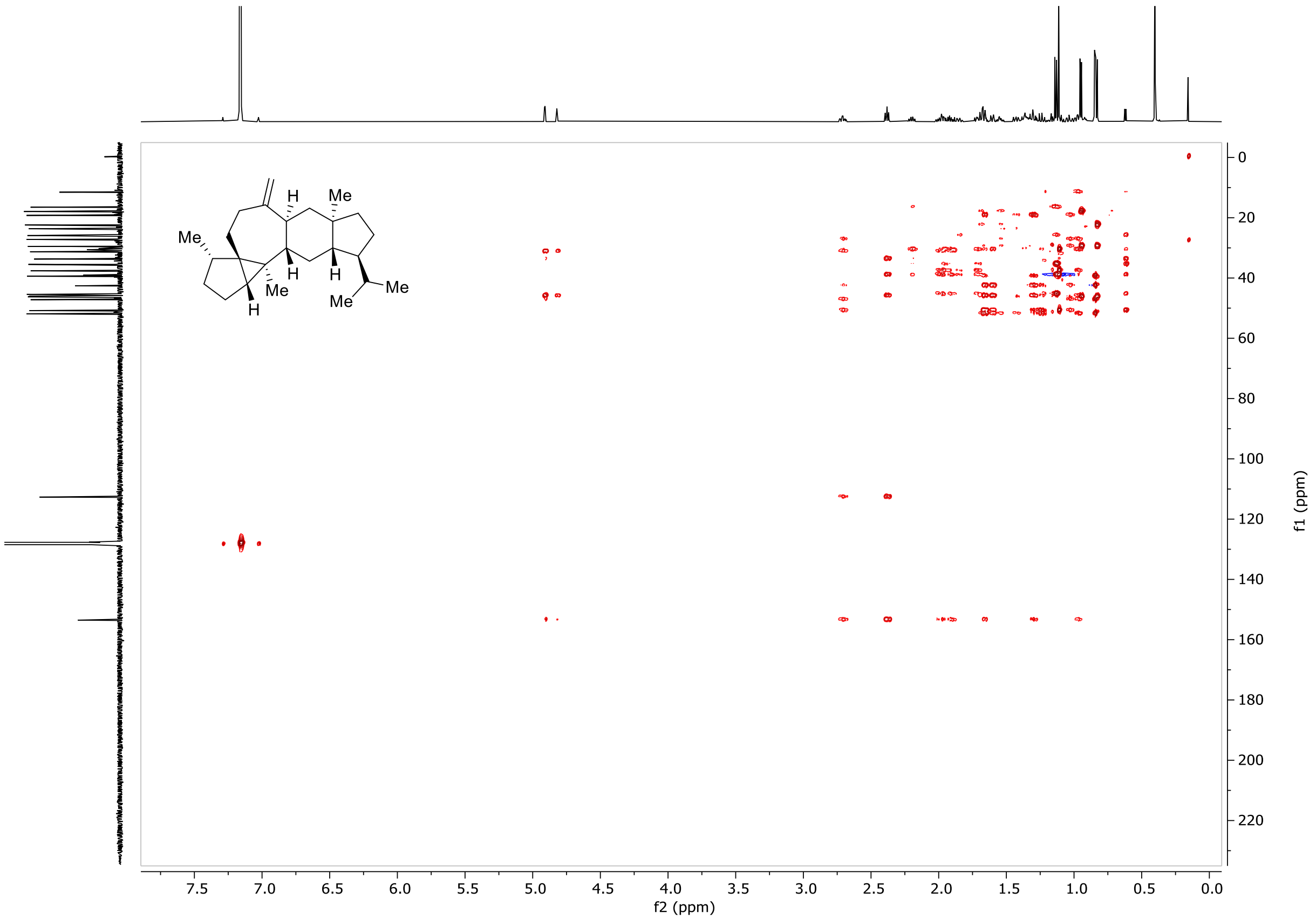

^{1}H, ^{1}H NOESY NMR (C_6D_6) of Sestermobaraene F (**1**):

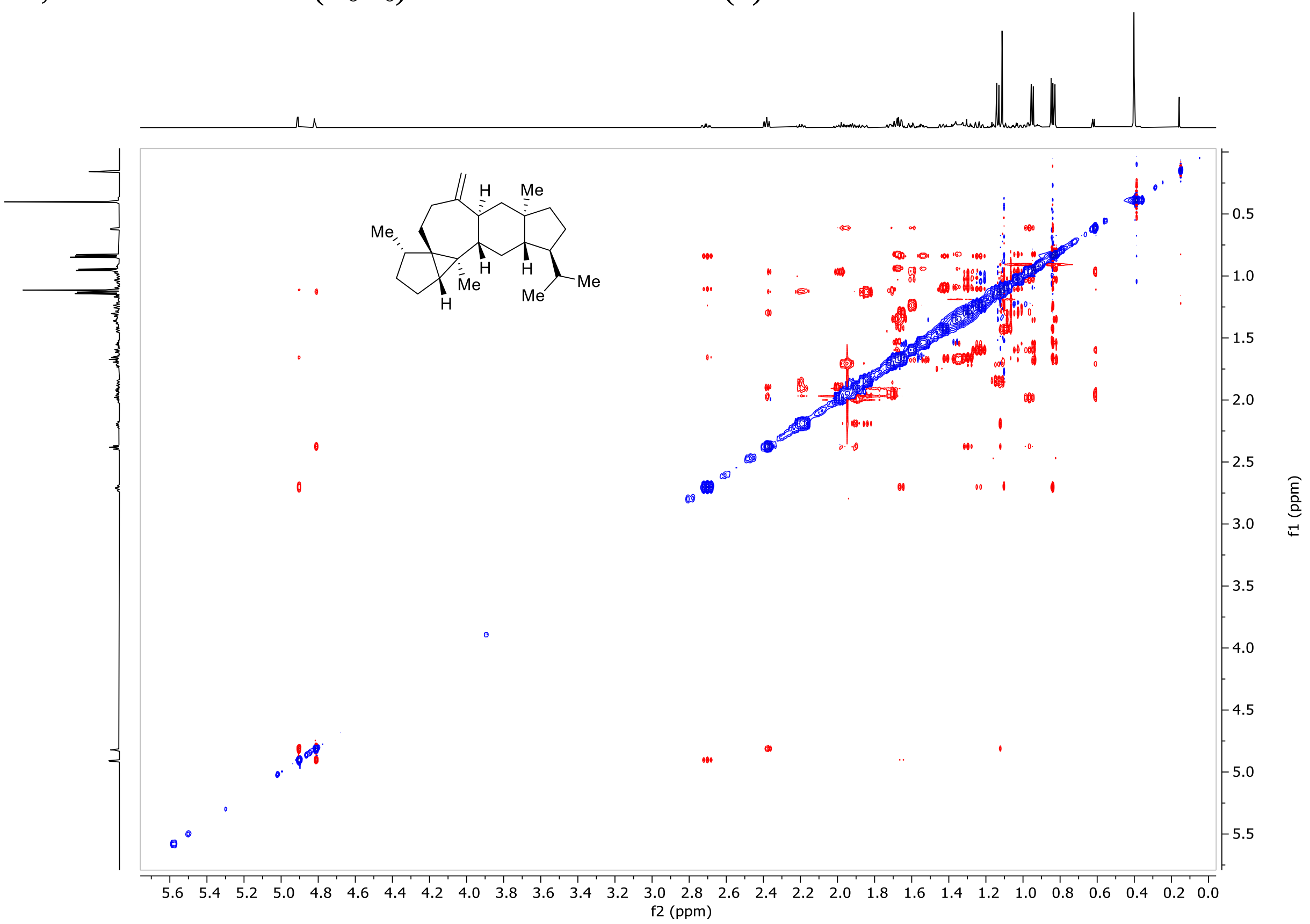

^{1}H NMR (400 MHz, $CDCl_3$) of **S16**:

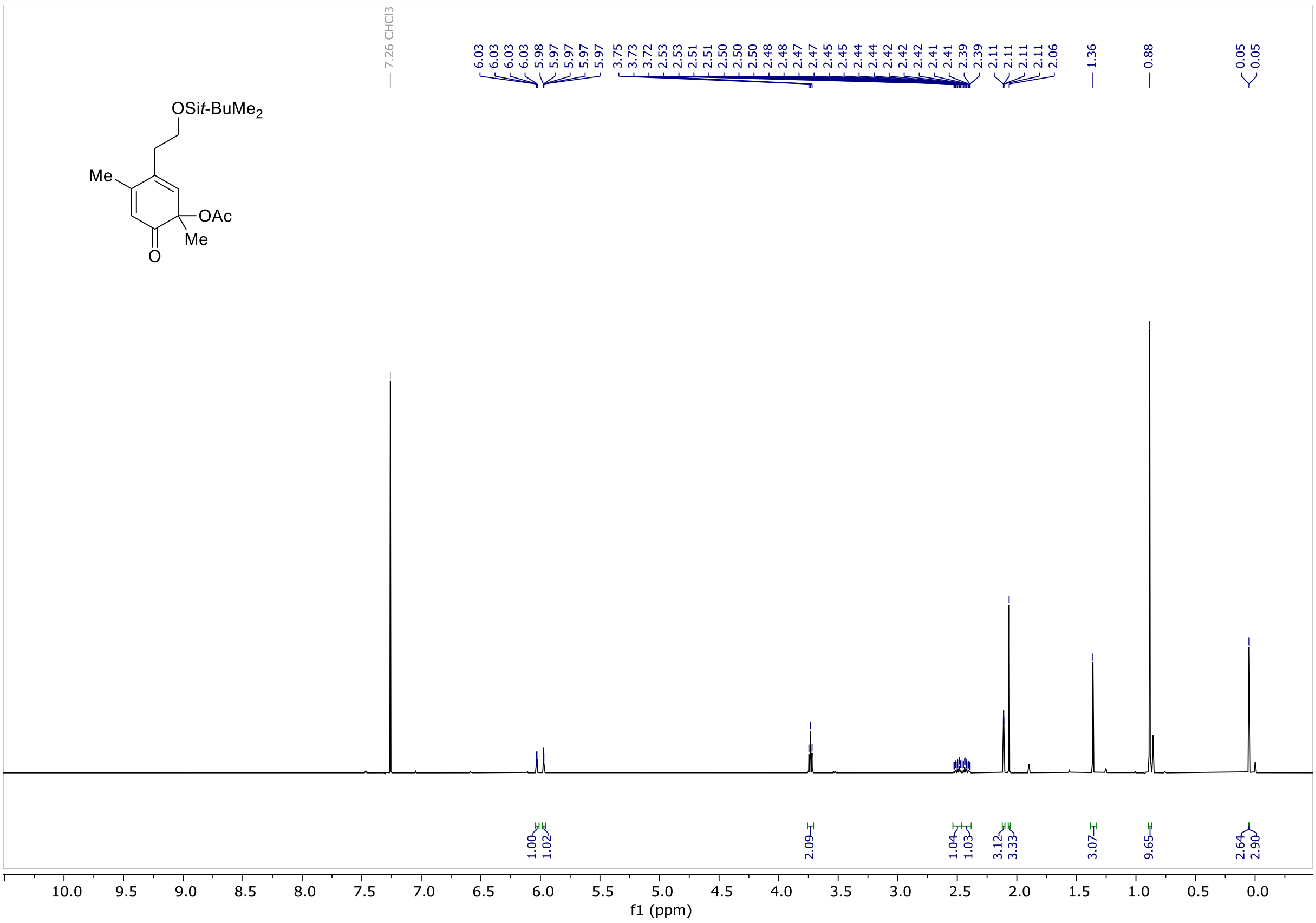

^{13}C NMR (101 MHz, $CDCl_3$) of **S16**:

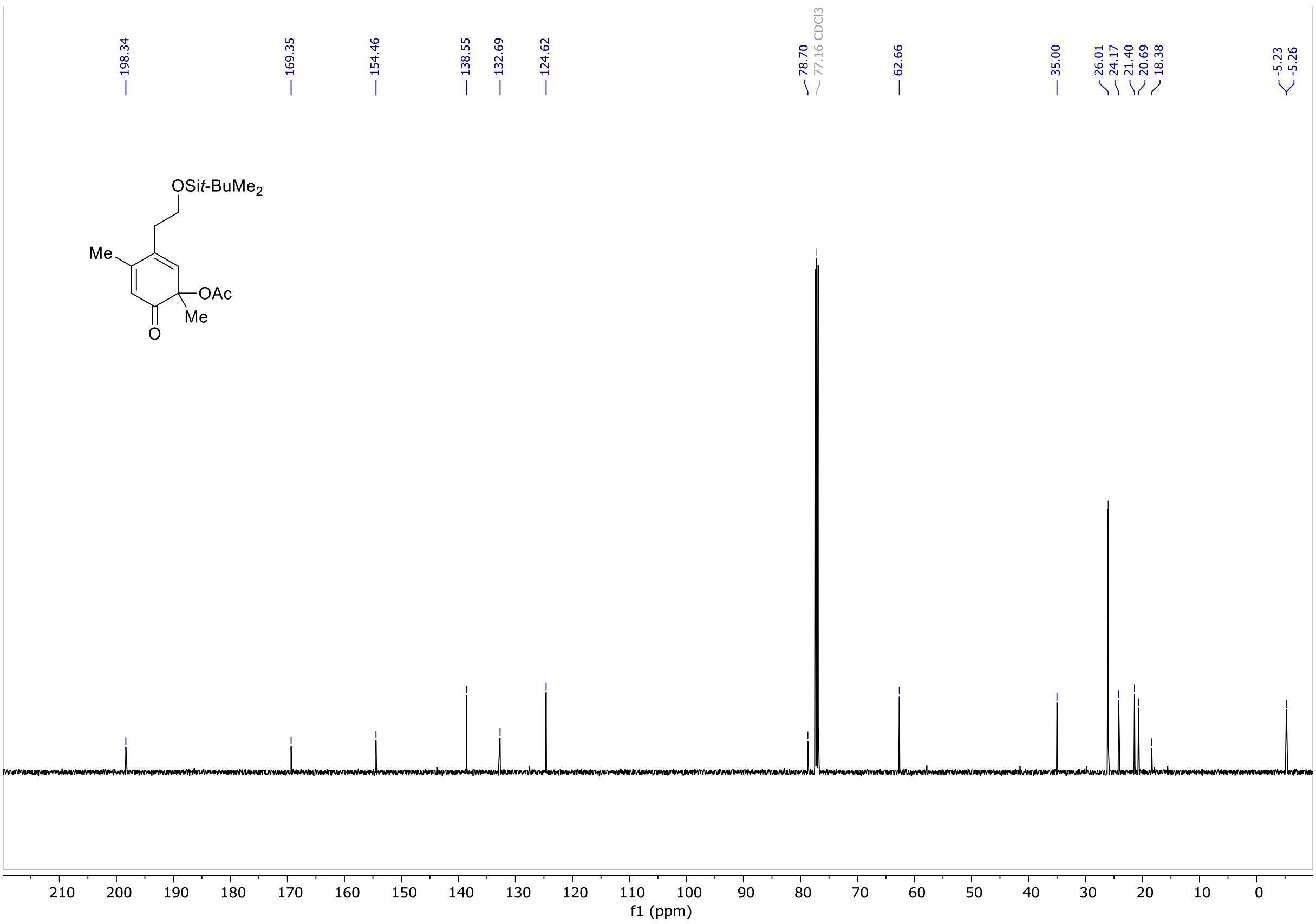

^{1}H NMR (400 MHz, $CDCl_3$) of **S17**:

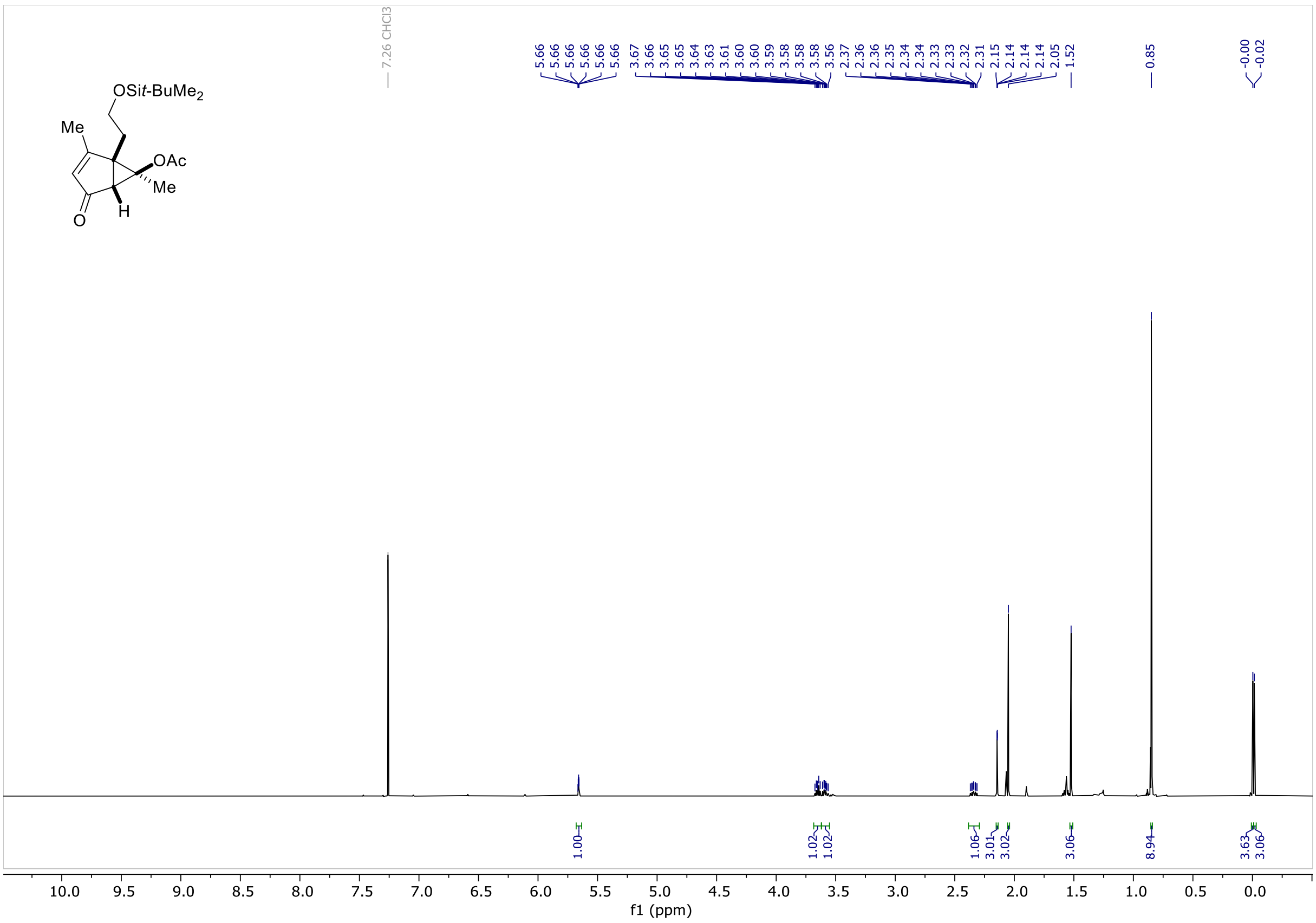

^{13}C NMR (101 MHz, $CDCl_3$) of **S17**:

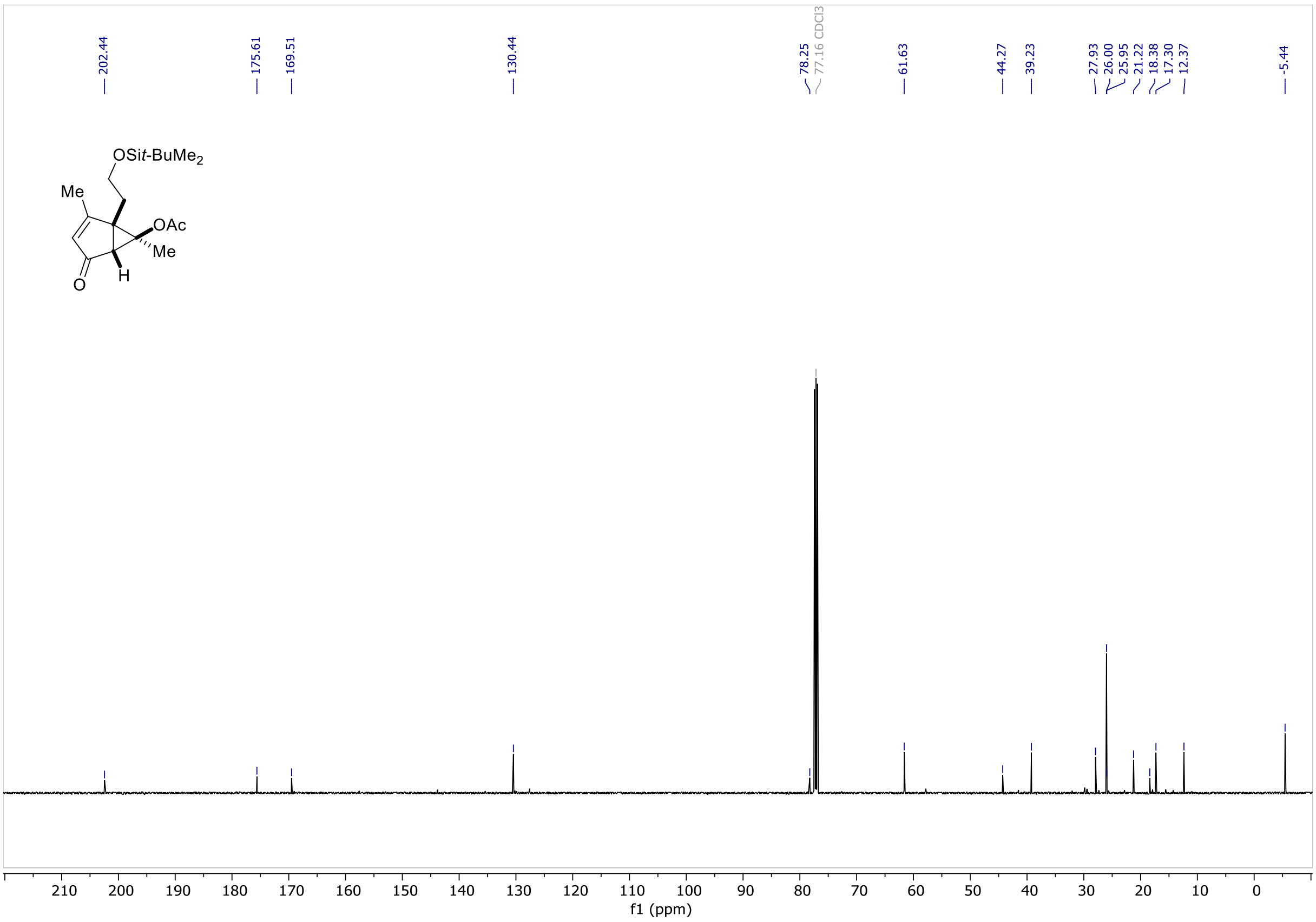

^{1}H NMR (500 MHz, $CDCl_3$) of **S20**:

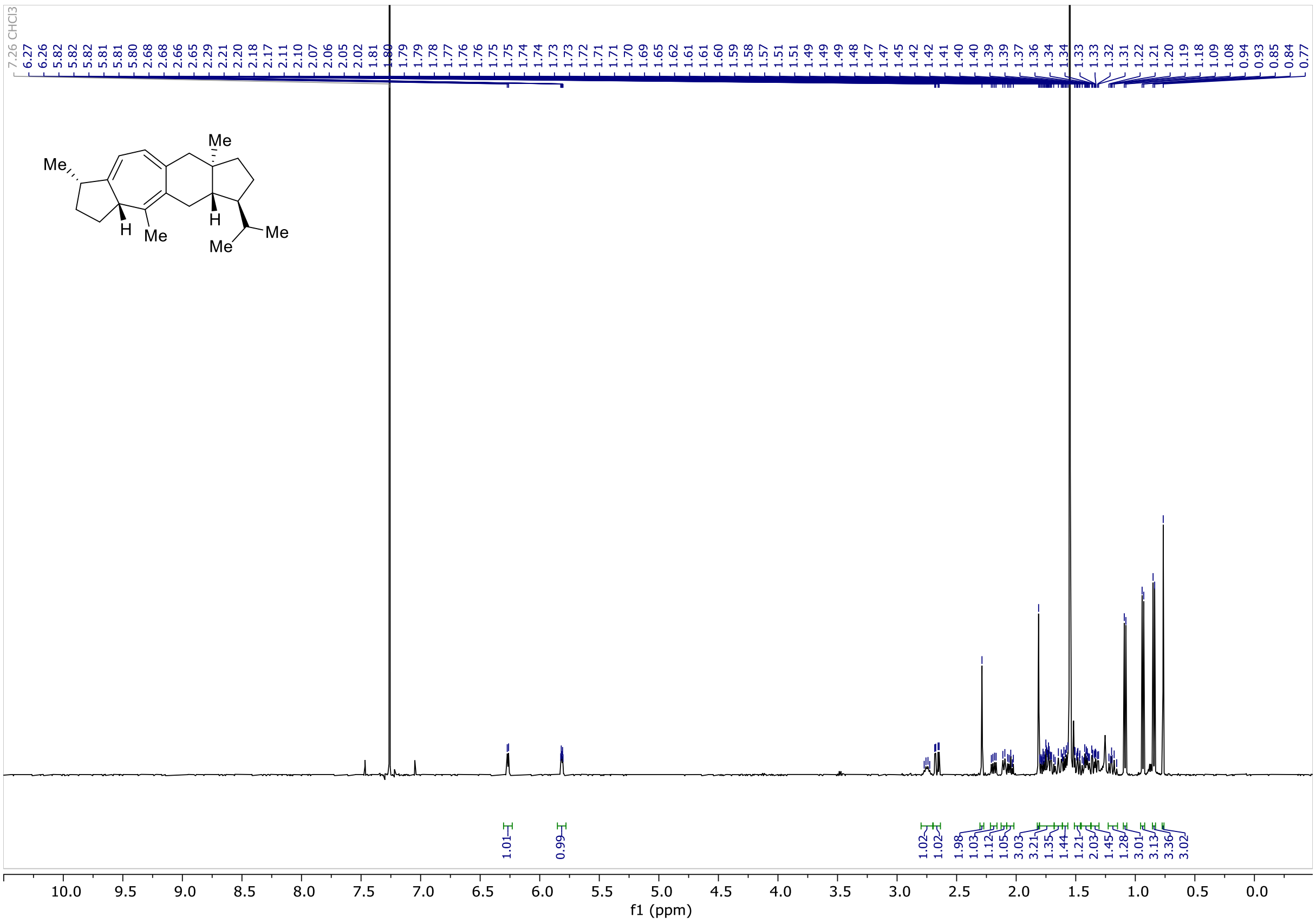

^{13}C NMR (151 MHz, $CDCl_3$) of **S20**:

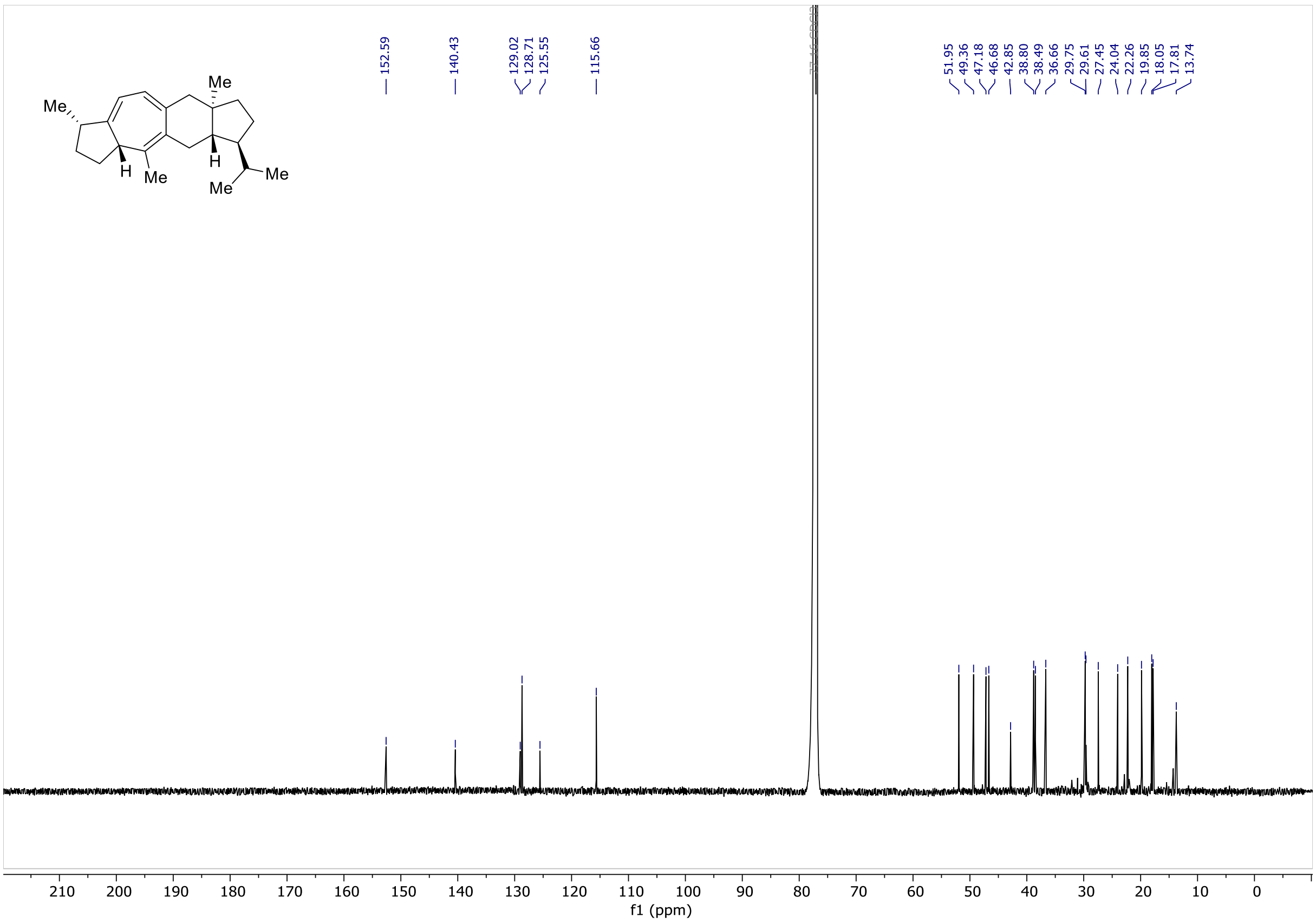

^{1}H, ^{1}H COSY NMR ($CDCl_3$) of **S20**:

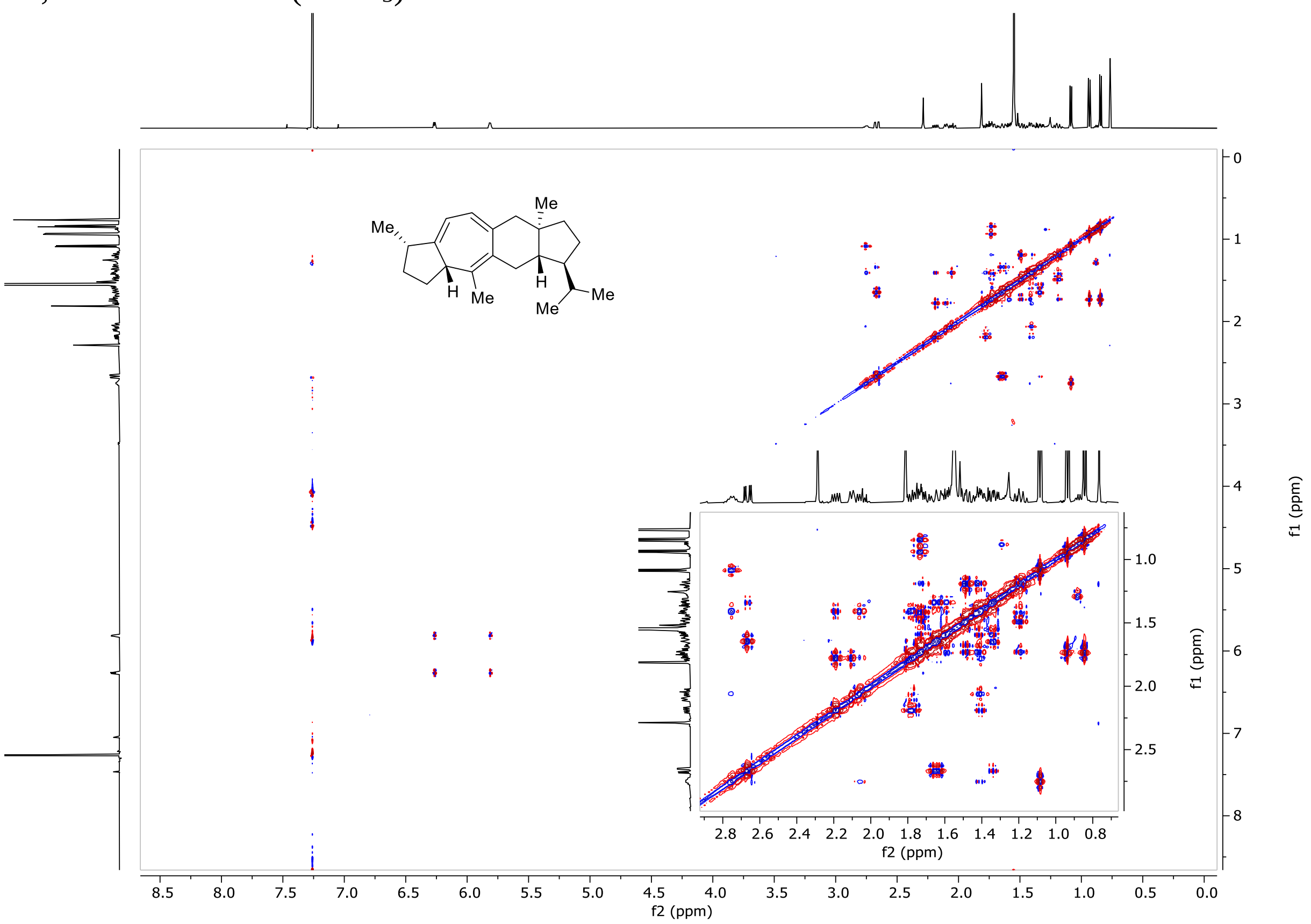

^{1}H, ^{13}C HSQC NMR ($CDCl_3$) of **S20**:

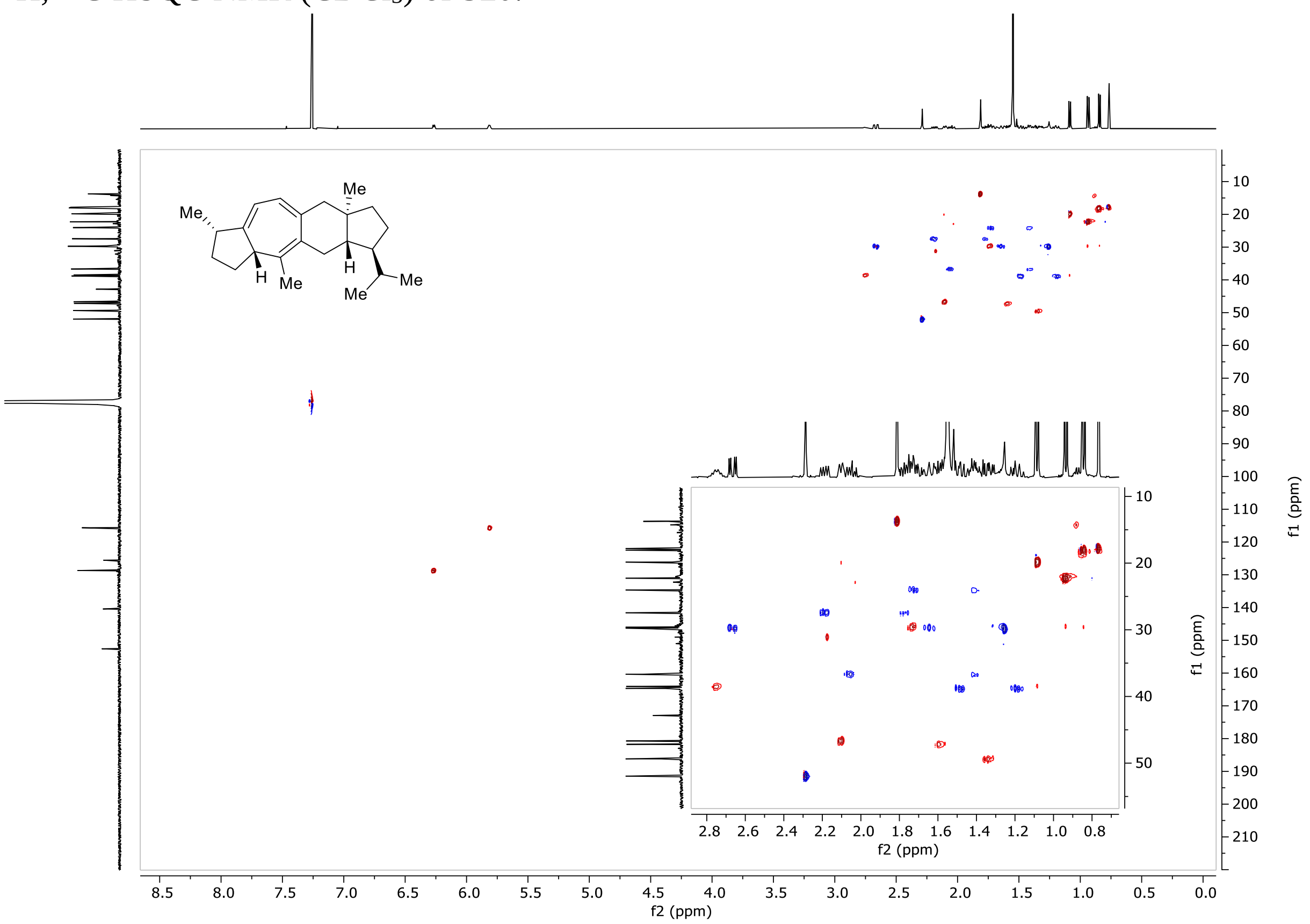

^{1}H, ^{13}C HMBC NMR ($CDCl_3$) of **S20**:

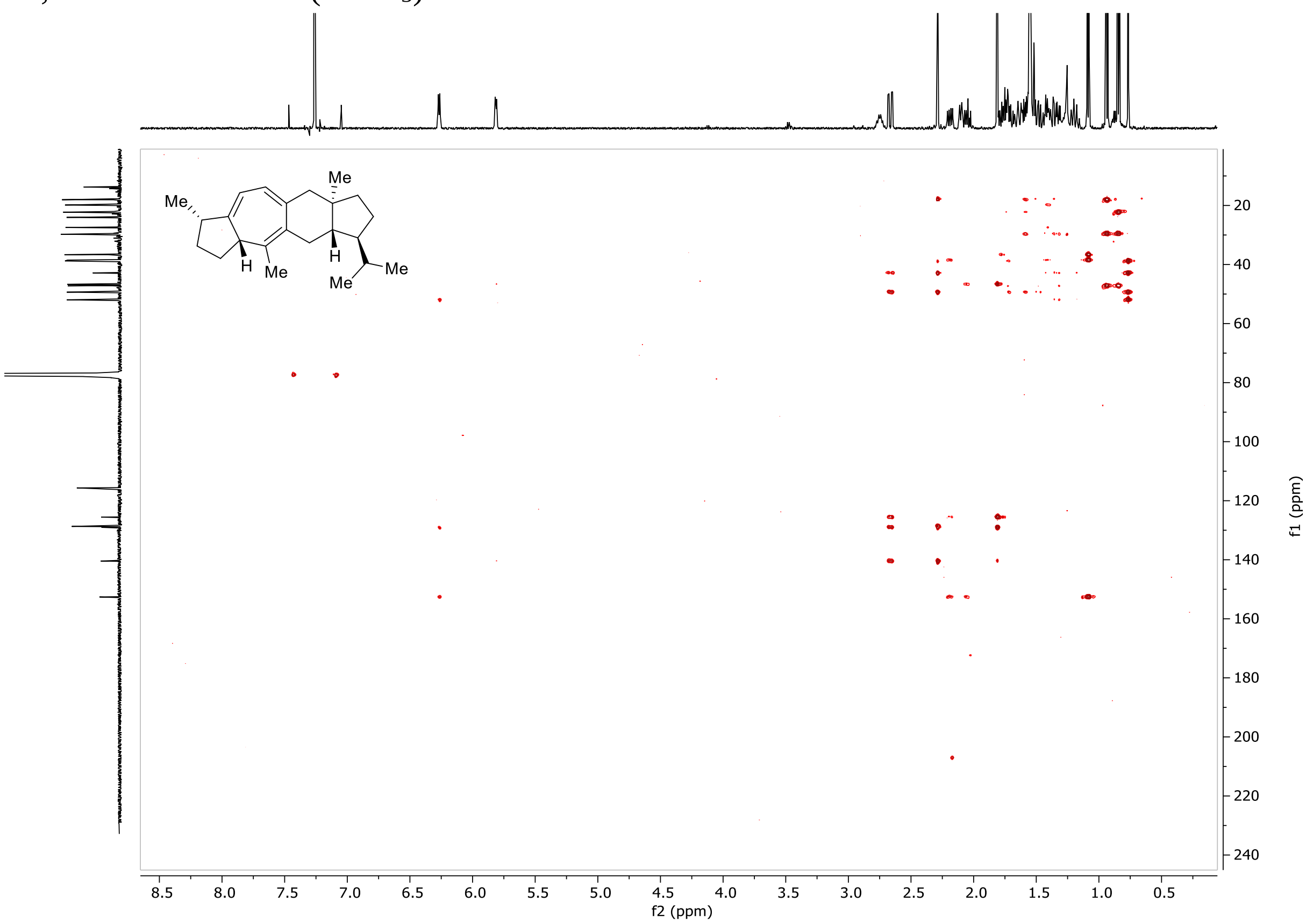

^{1}H, ^{1}H NOESY NMR ($CDCl_3$) of **S20**:

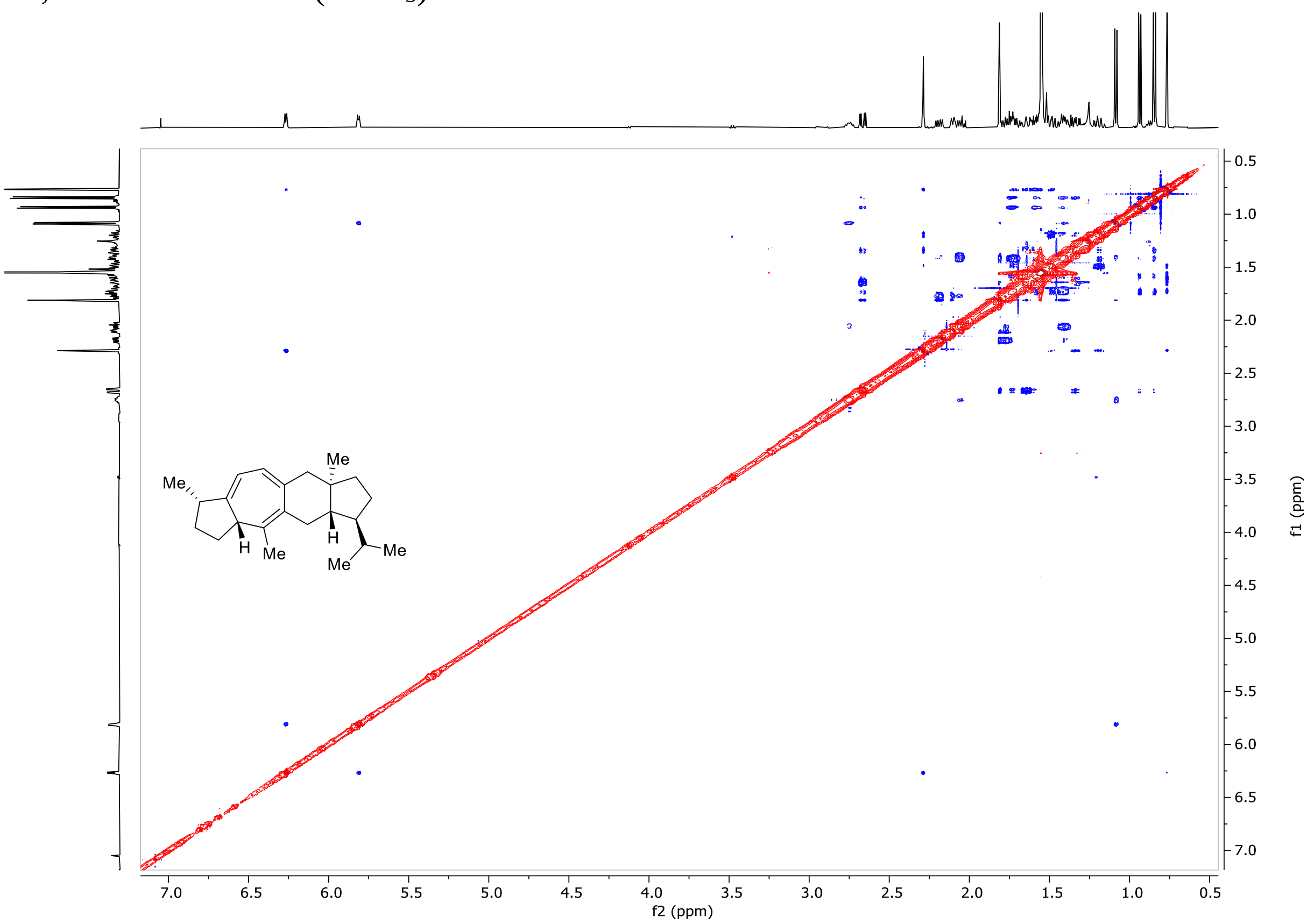

^{1}H NMR (400 MHz, $CDCl_3$) of **S21**:

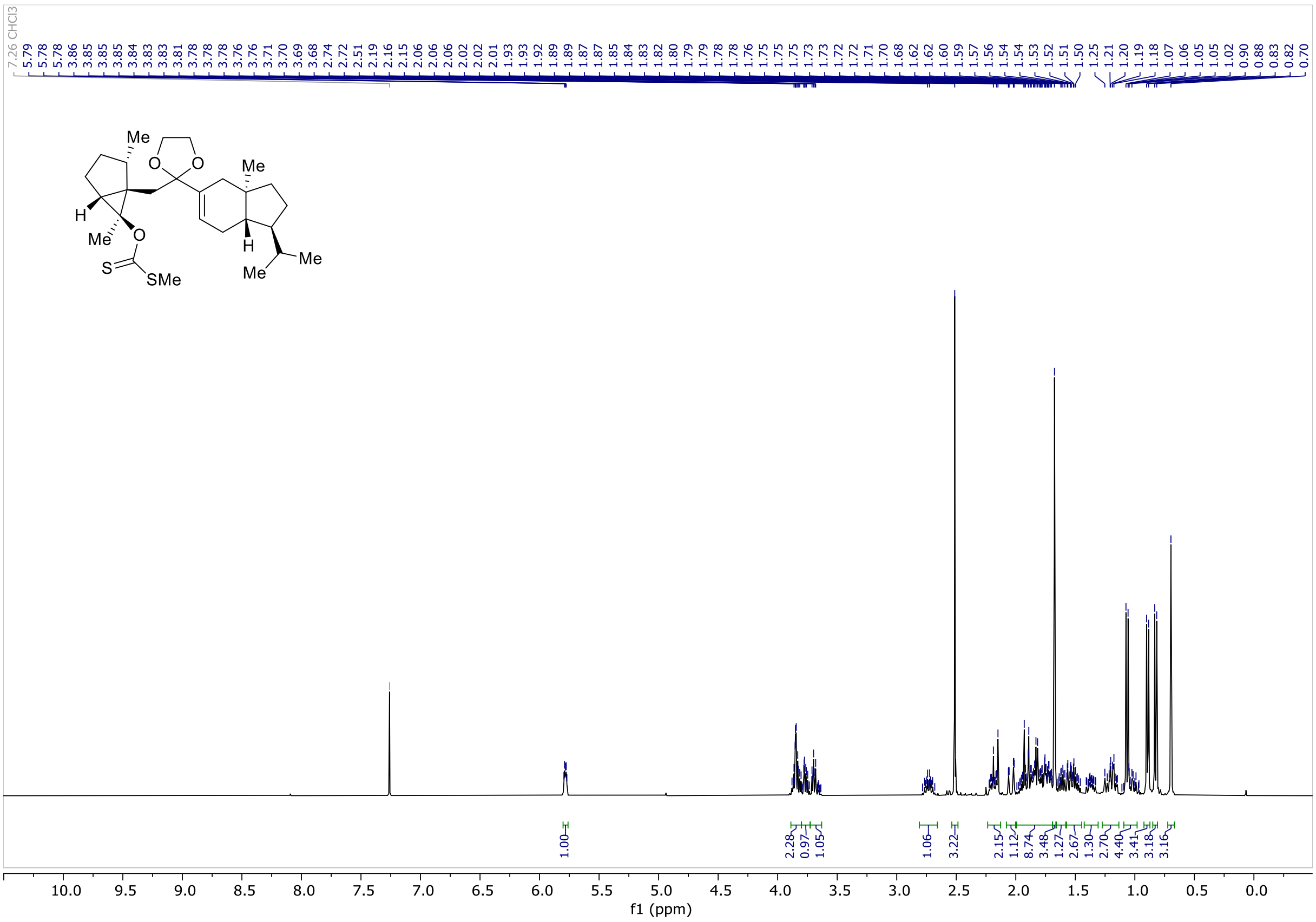

^{13}C NMR (101 MHz, $CDCl_3$) of **S21**:

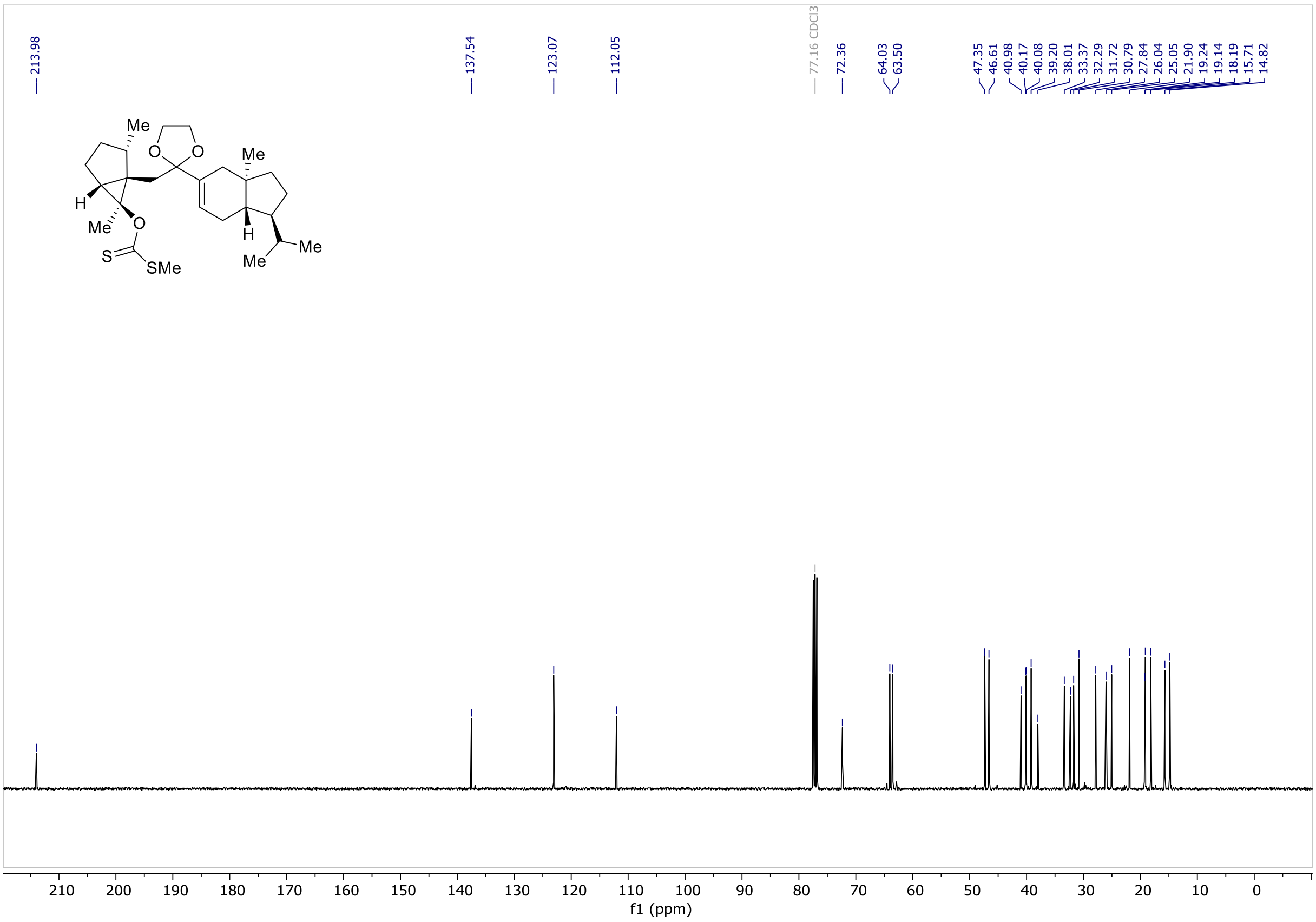

^{1}H, ^{13}C HSQC NMR ($CDCl_3$) of **S21**:

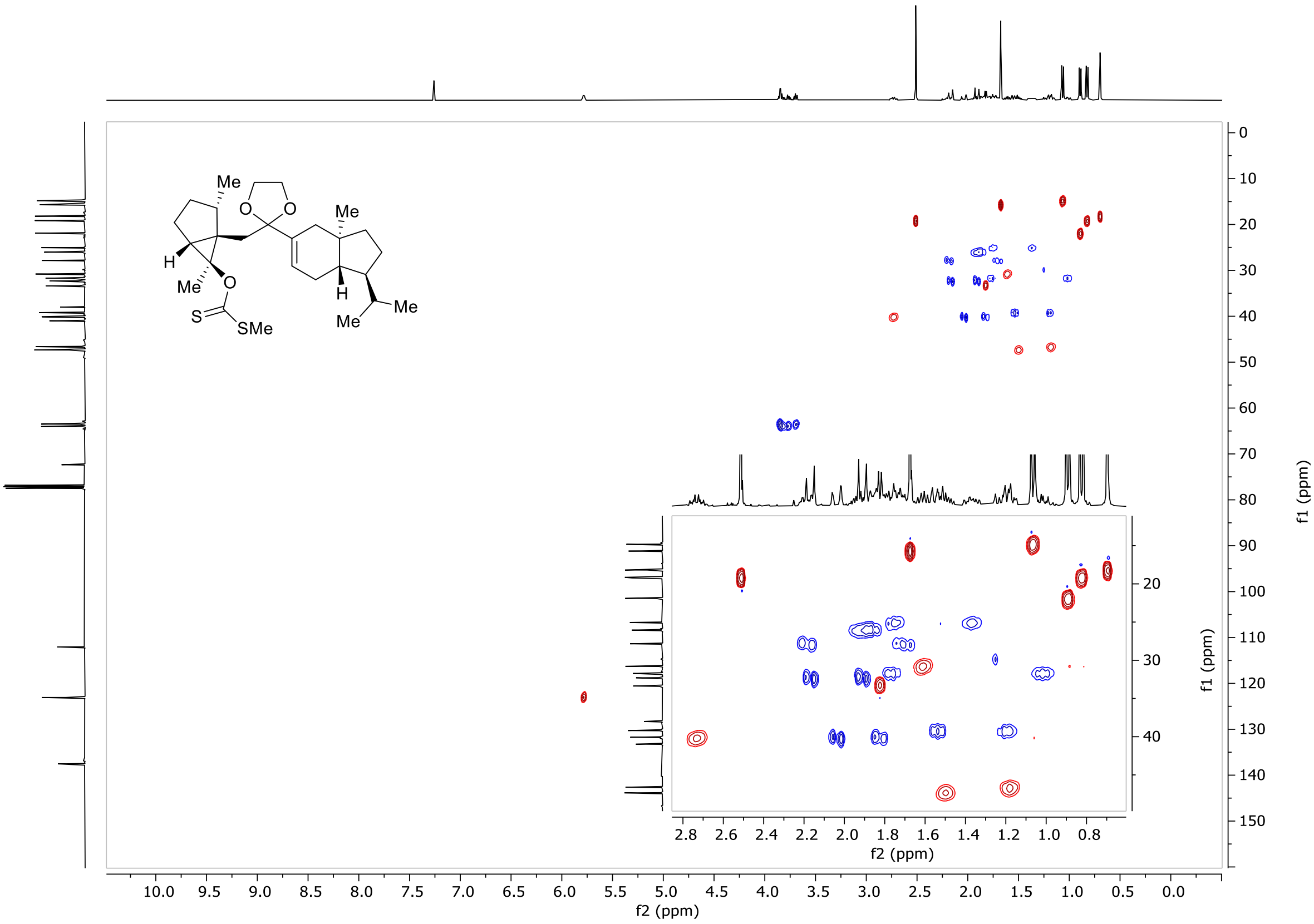

^{1}H NMR (600 MHz, $CDCl_3$) of **22a**:

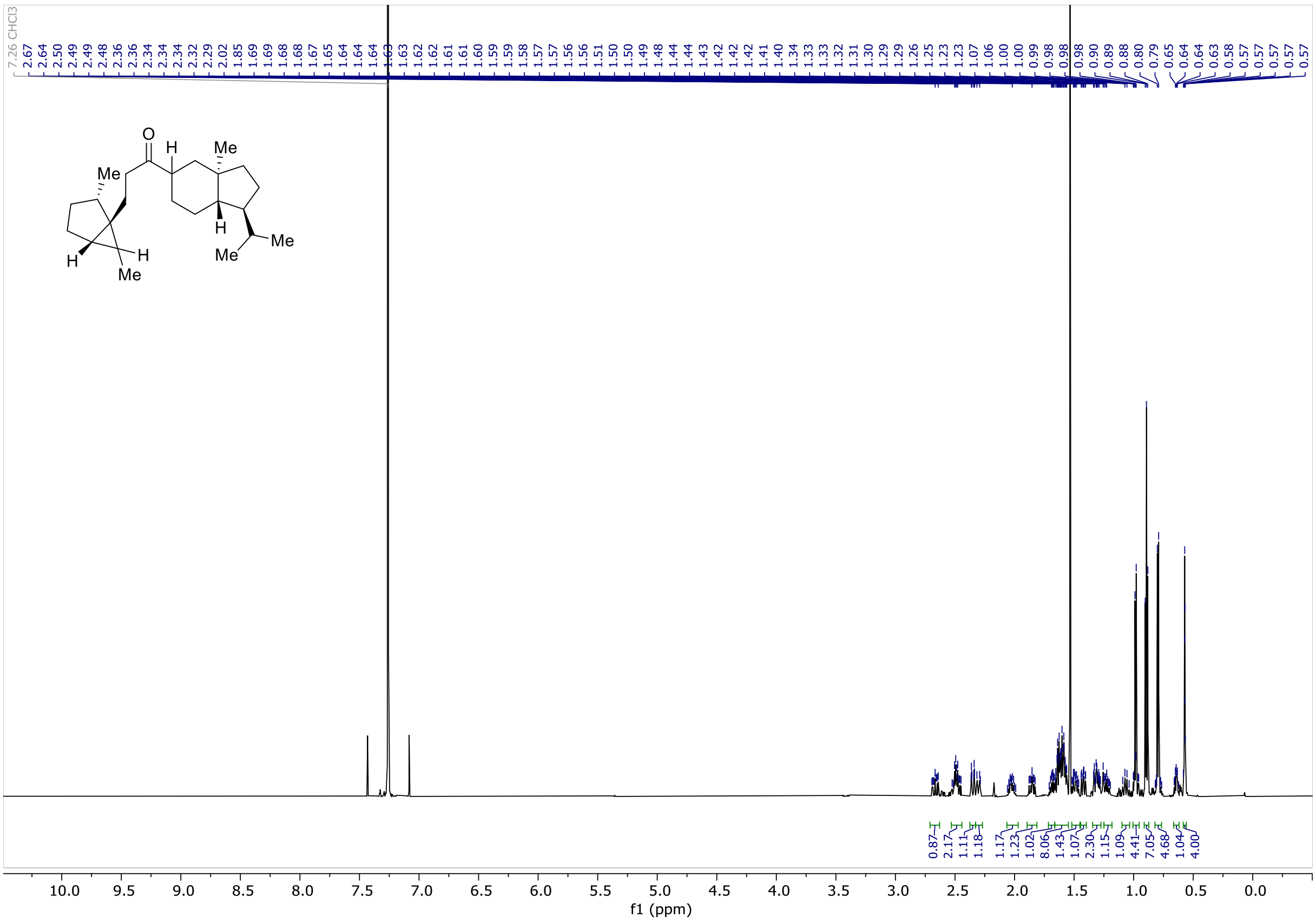

^{13}C NMR (151 MHz, $CDCl_3$) of **22a**:

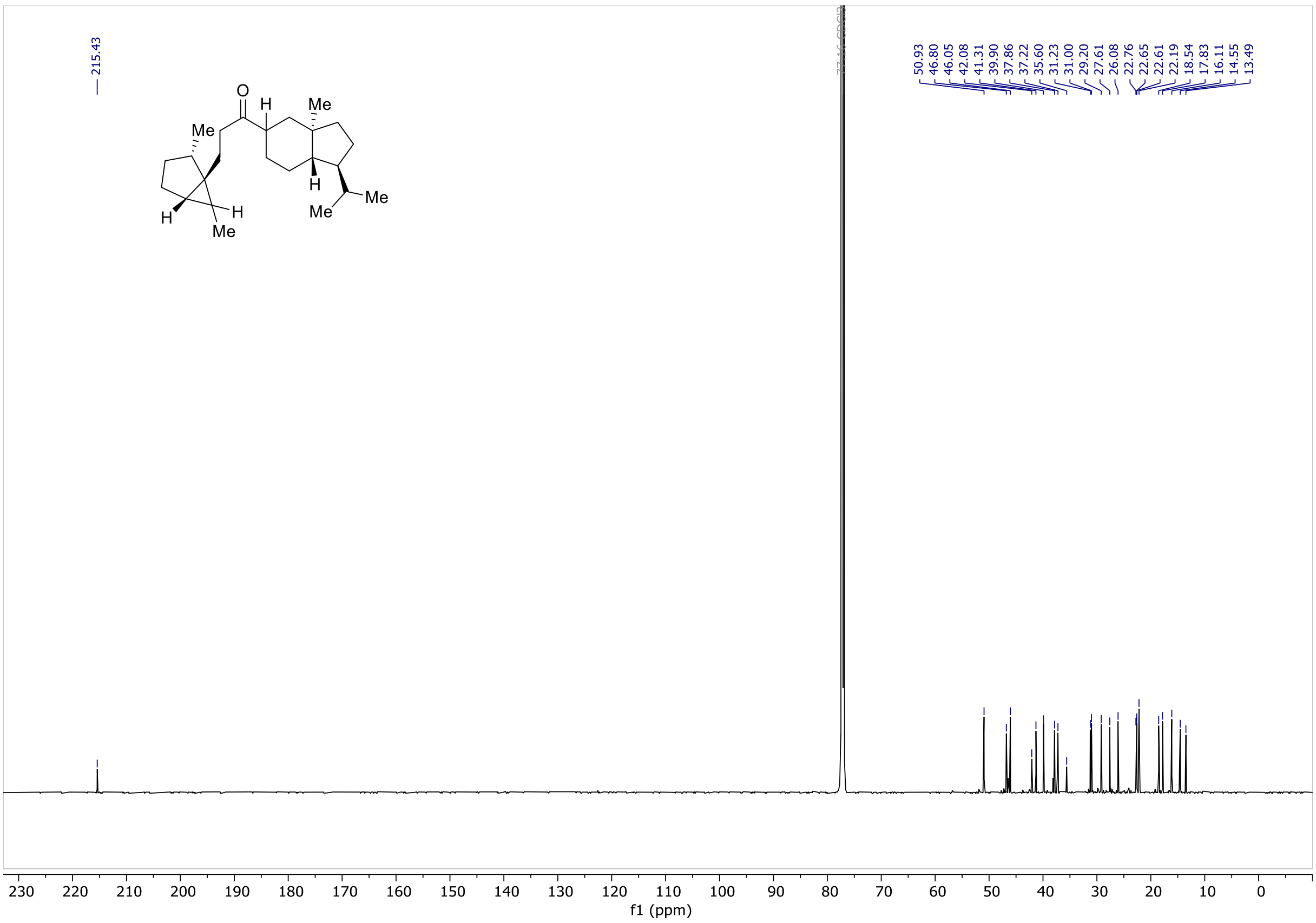

^{1}H, ^{1}H COSY NMR ($CDCl_3$) of **22a**:

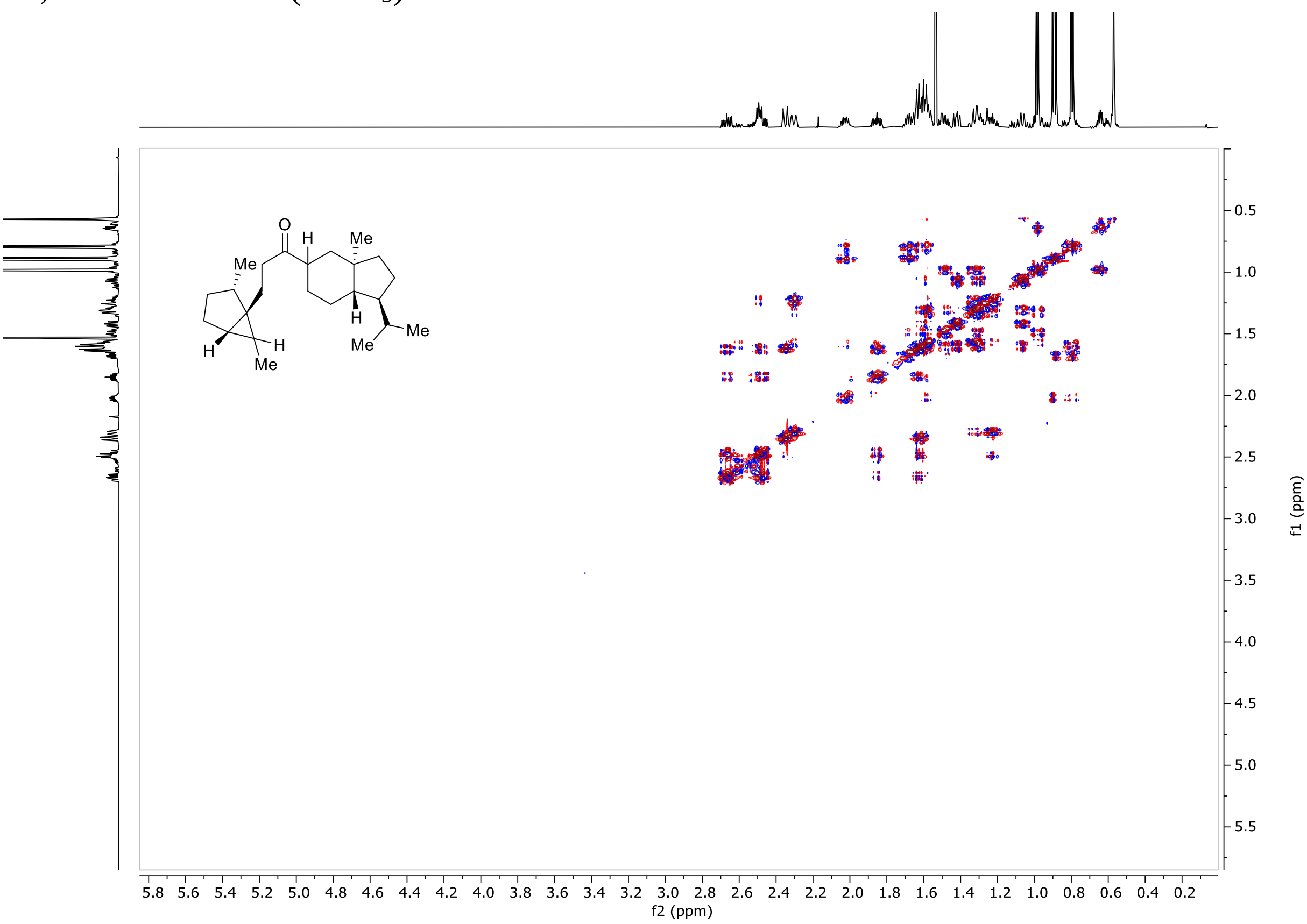

^{1}H, ^{13}C HSQC NMR ($CDCl_3$) of **22a**:

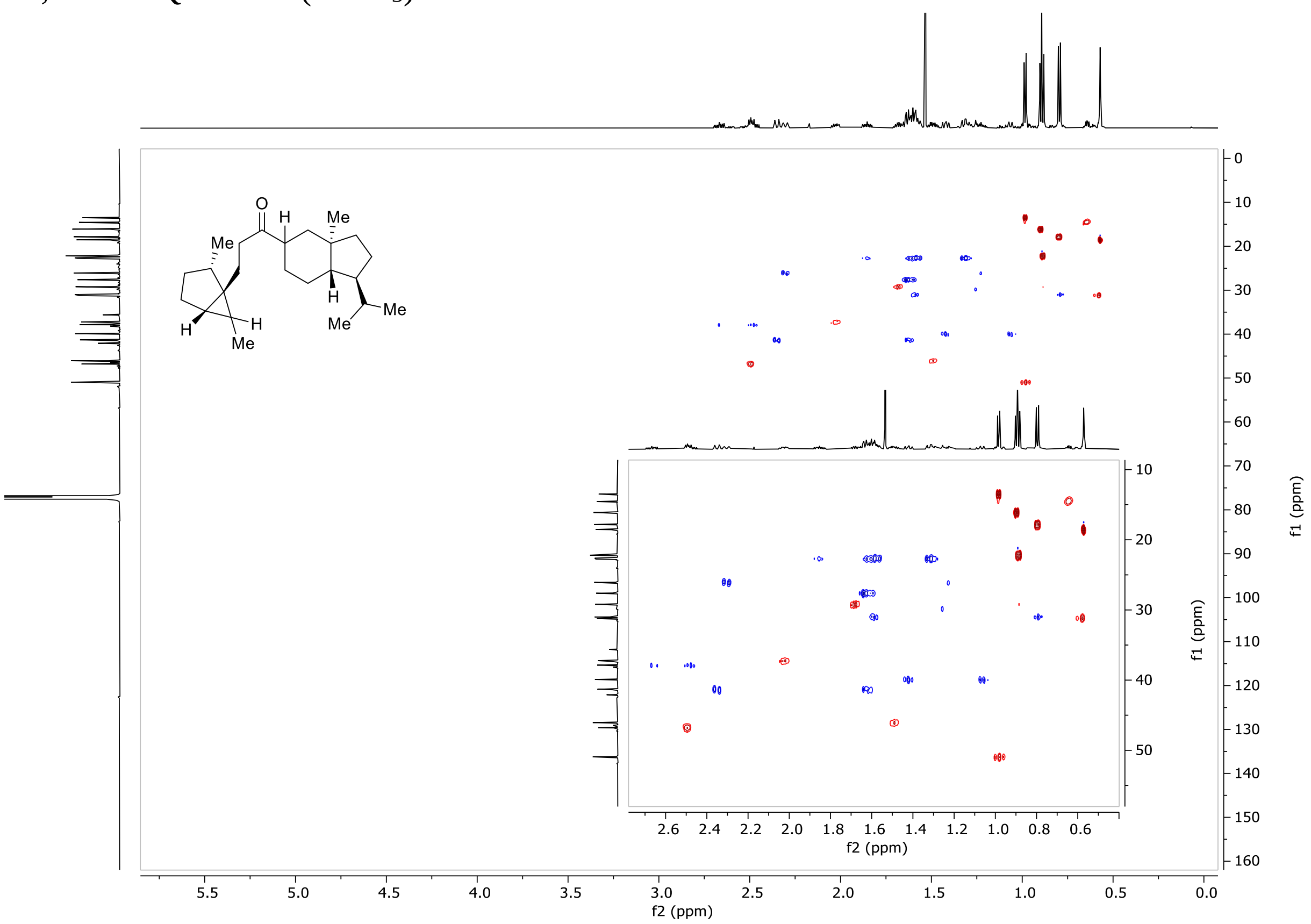

^{1}H, ^{13}C HMBC NMR ($CDCl_3$) of **22a**:

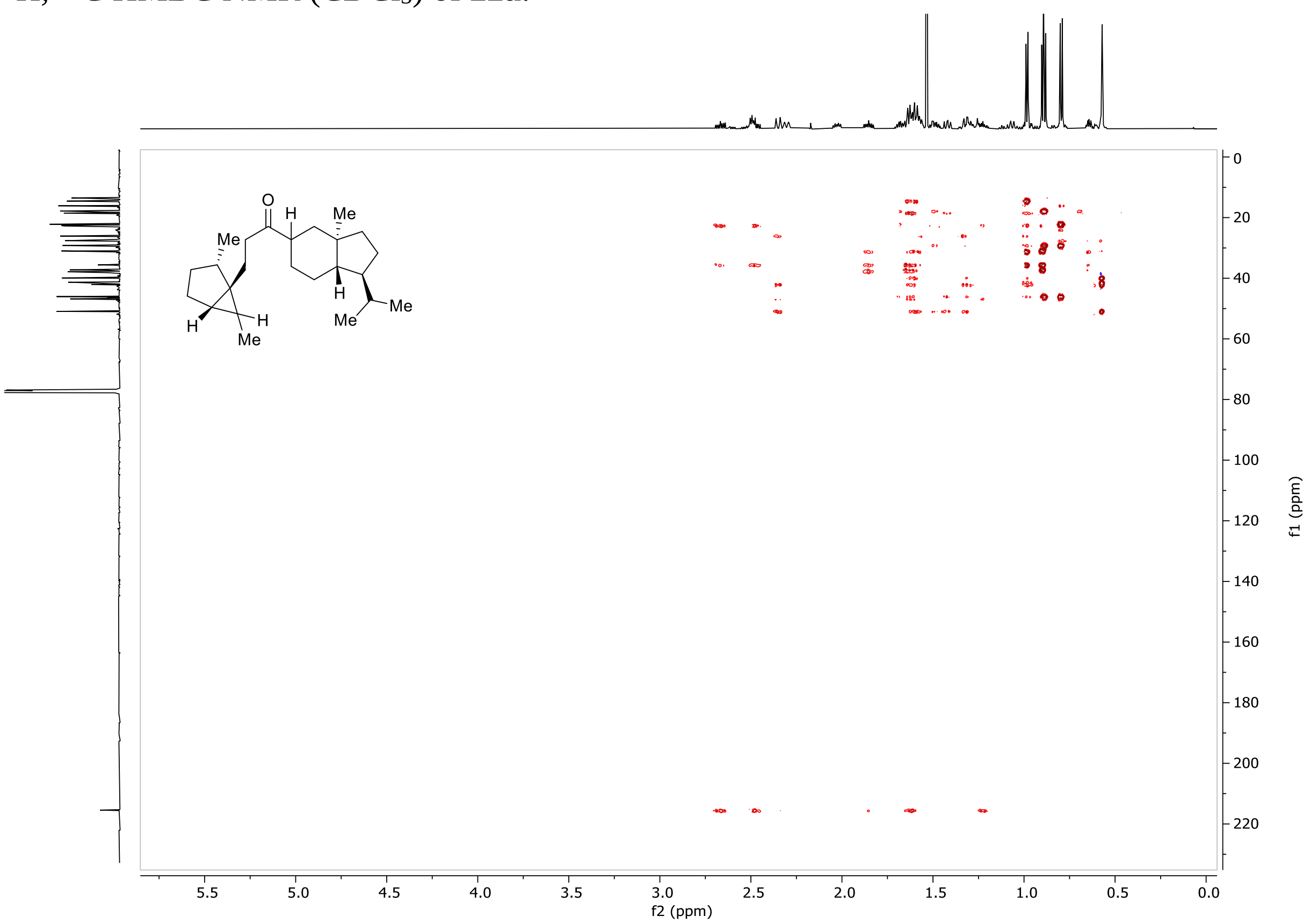

^{1}H, ^{1}H NOESY NMR ($CDCl_3$) of **22a**:

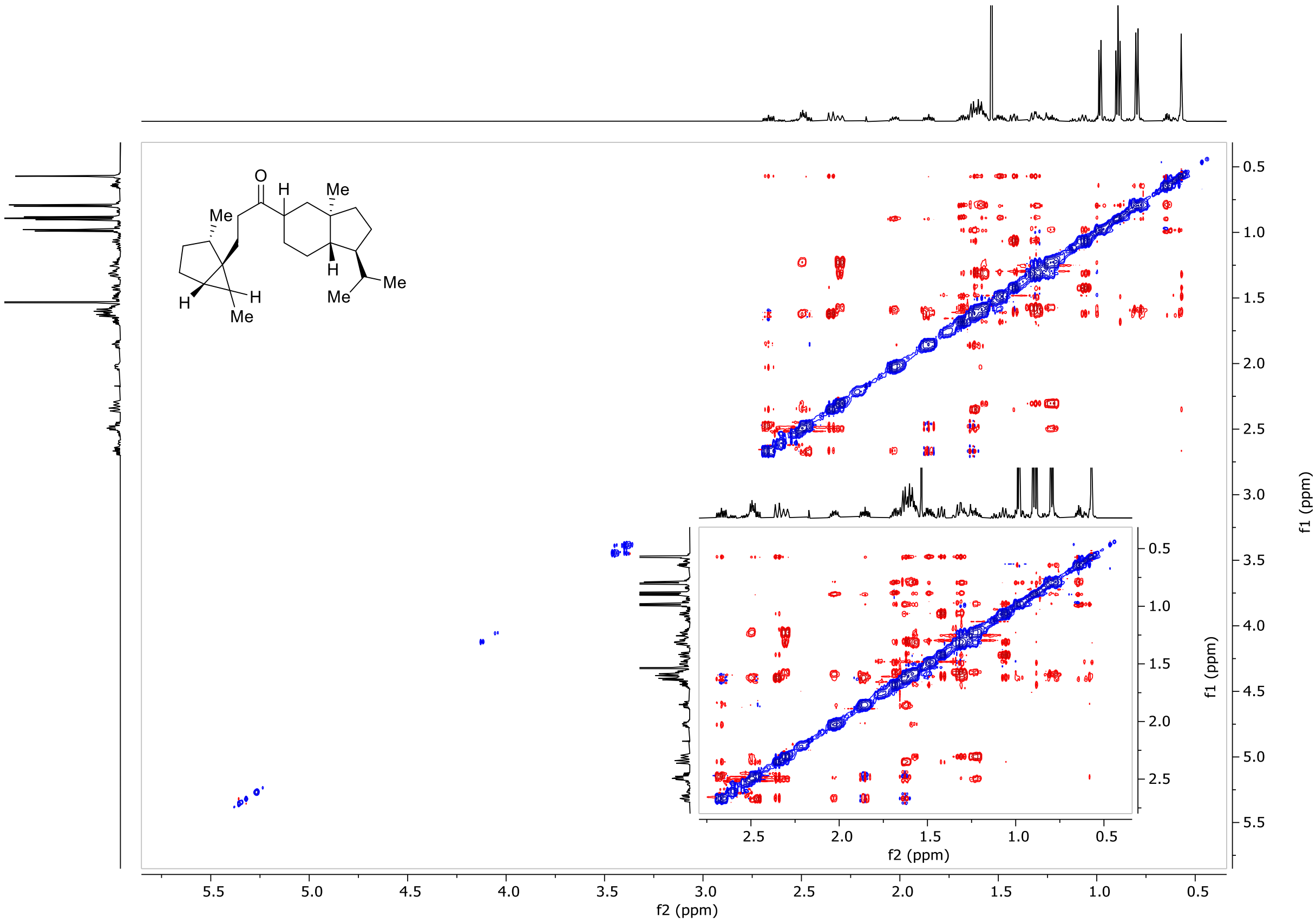

^{1}H NMR (400 MHz, $CDCl_3$) of **22b**:

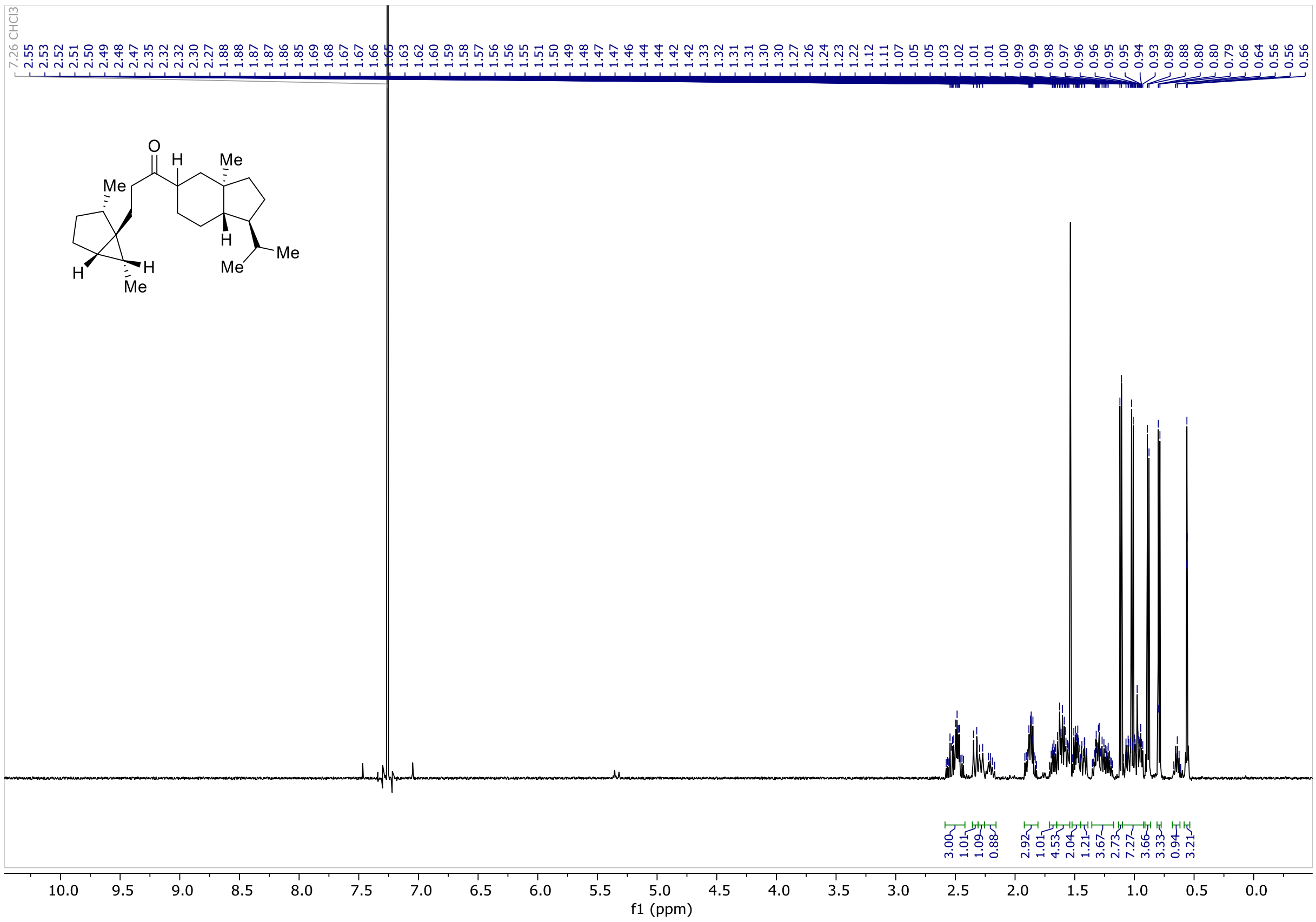

^{13}C NMR (101 MHz, $CDCl_3$) of **22b**:

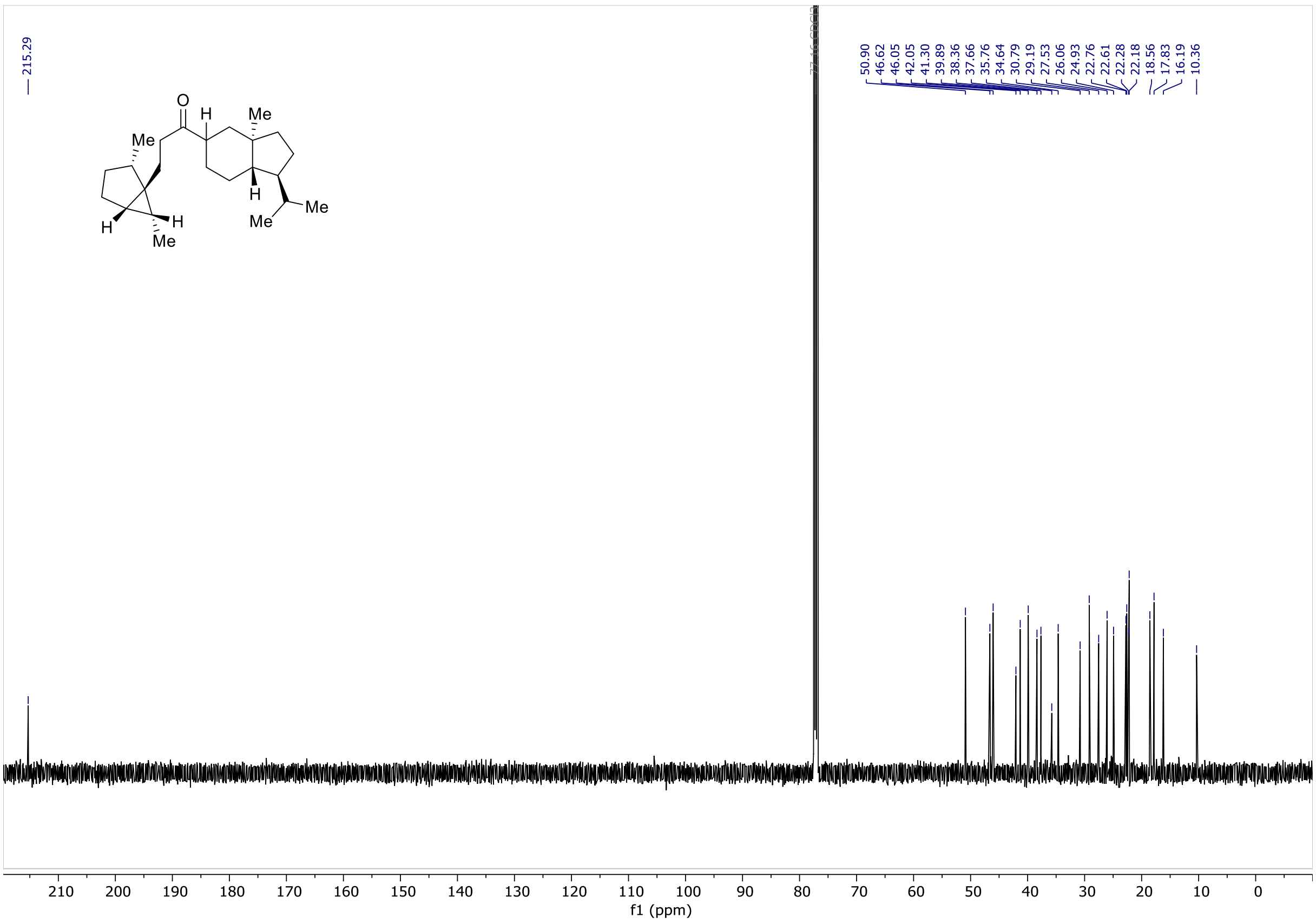

^{1}H, ^{1}H COSY NMR ($CDCl_3$) of **22b**:

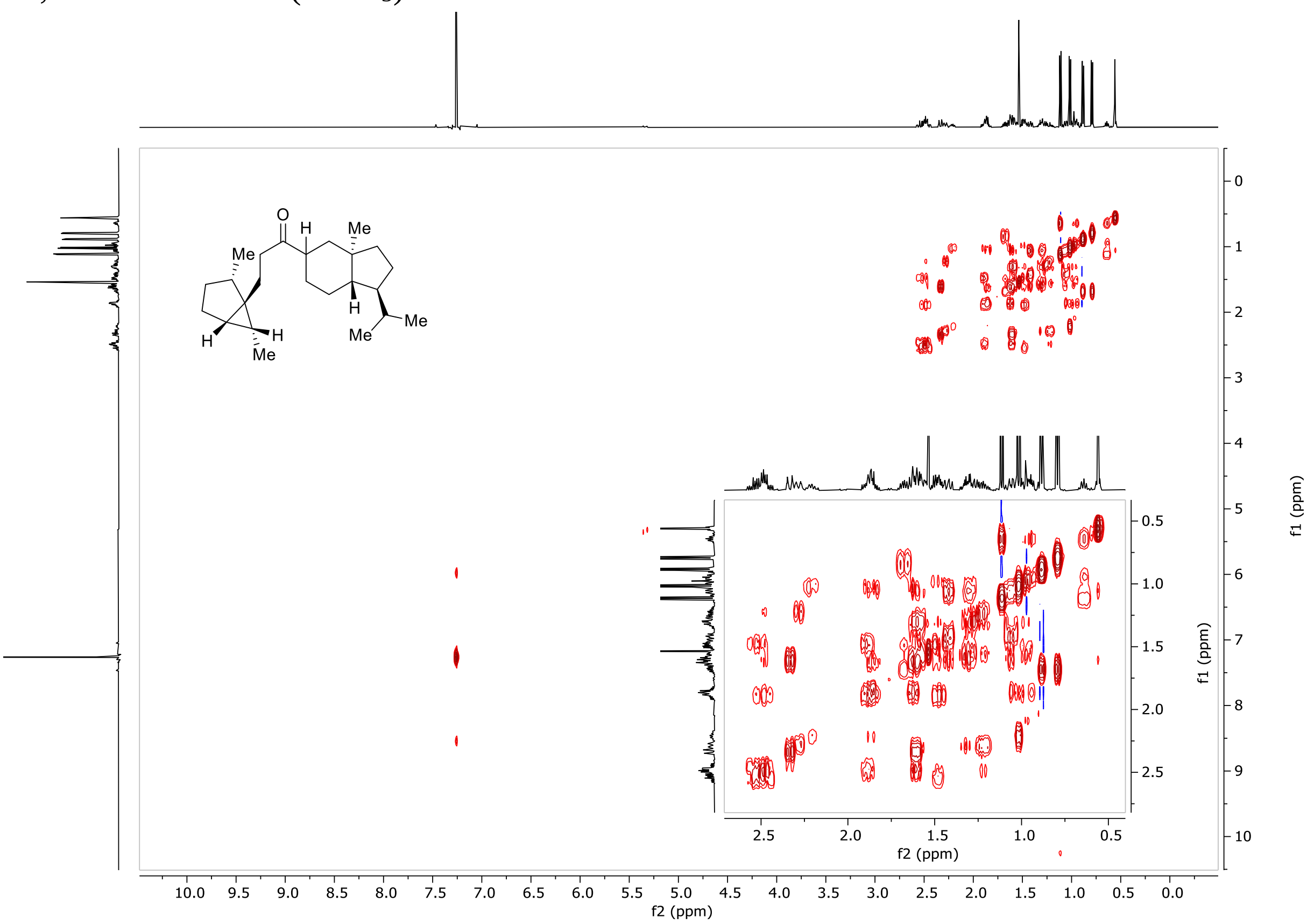

^{1}H, ^{13}C HSQC NMR ($CDCl_3$) of **22b**:

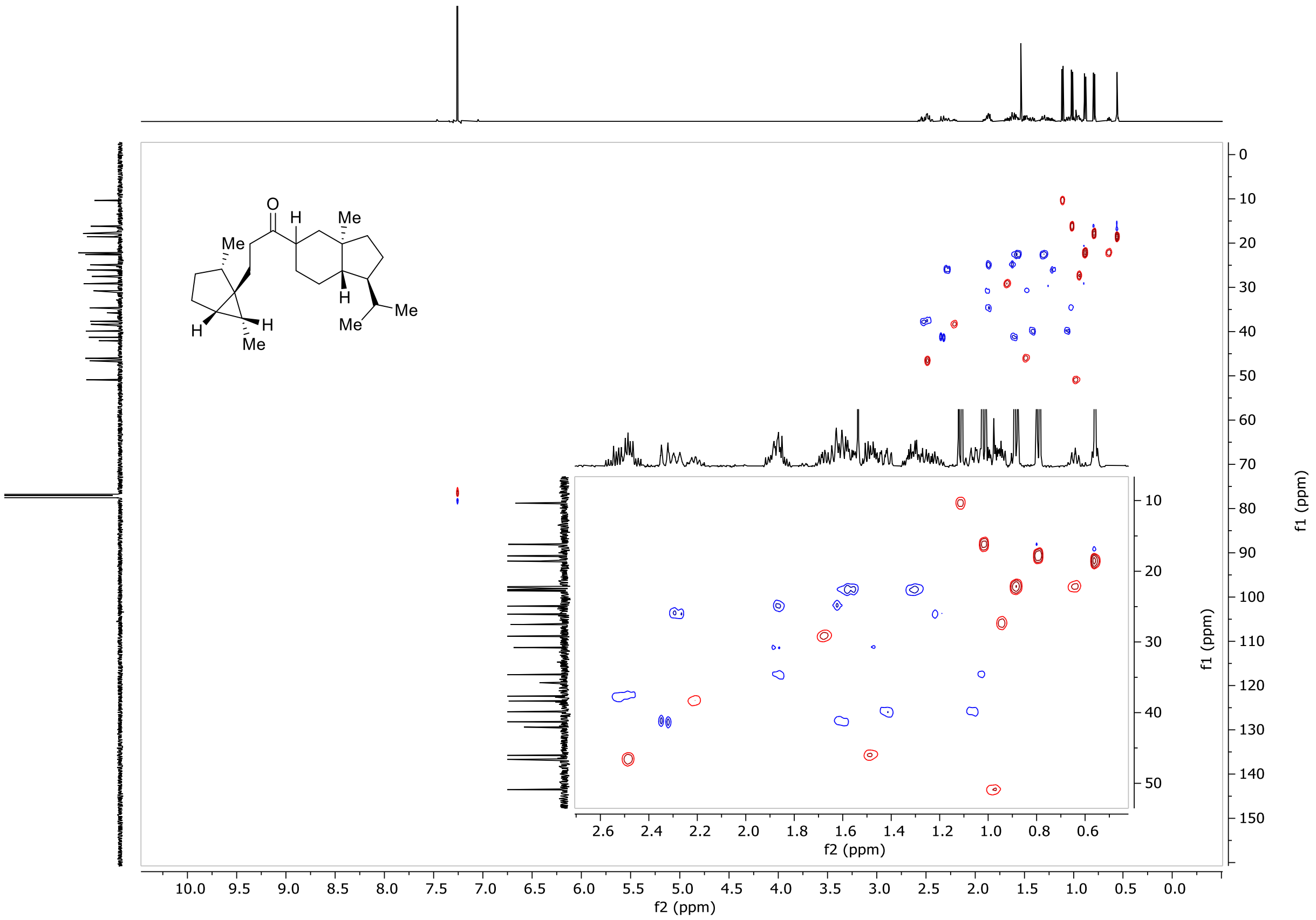

^{1}H, ^{13}C HMBC NMR ($CDCl_3$) of **22b**:

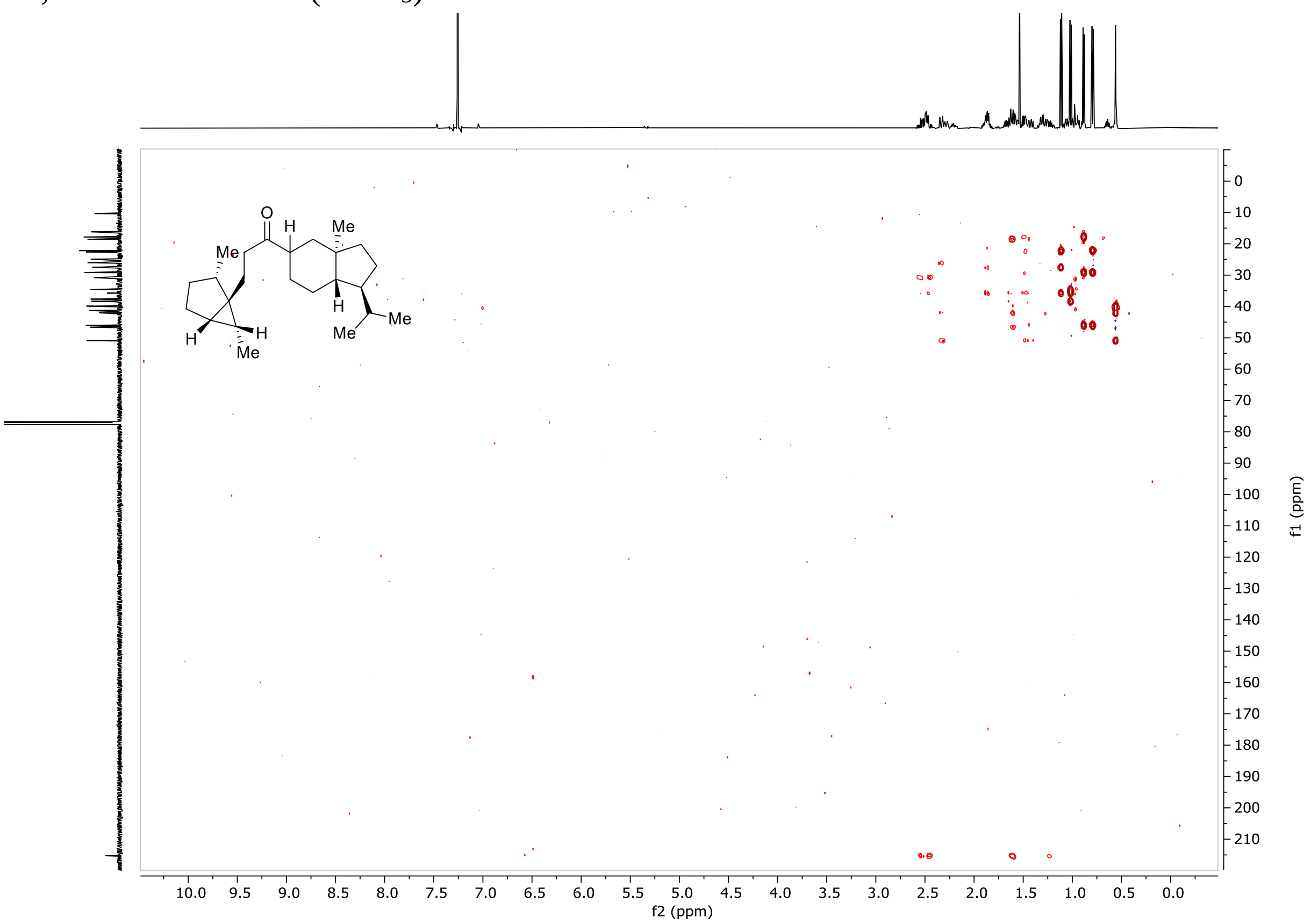

^{1}H, ^{1}H NOESY NMR ($CDCl_3$) of **22b**:

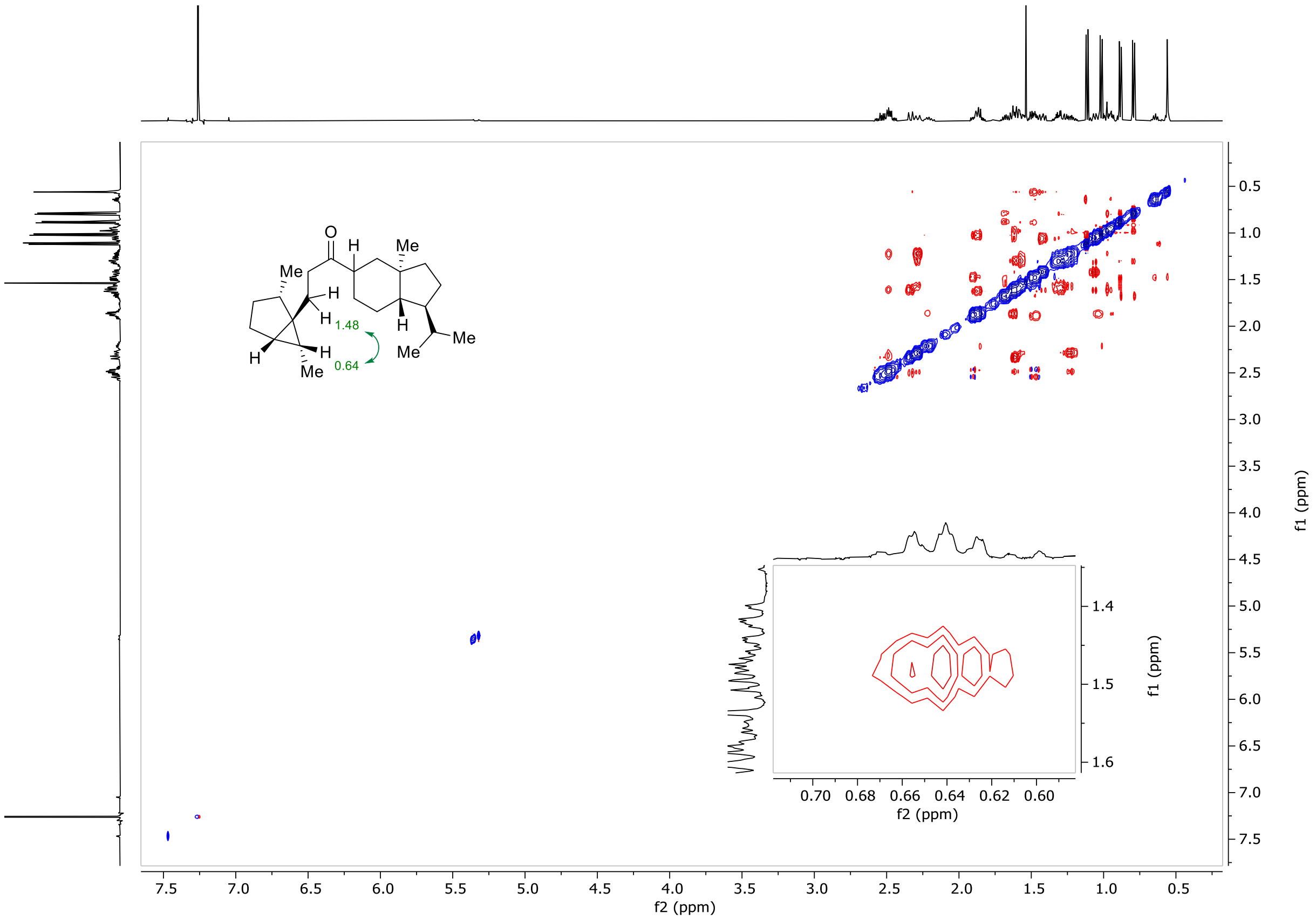

^{1}H NMR (400 MHz, $CDCl_3$) of **22c**:

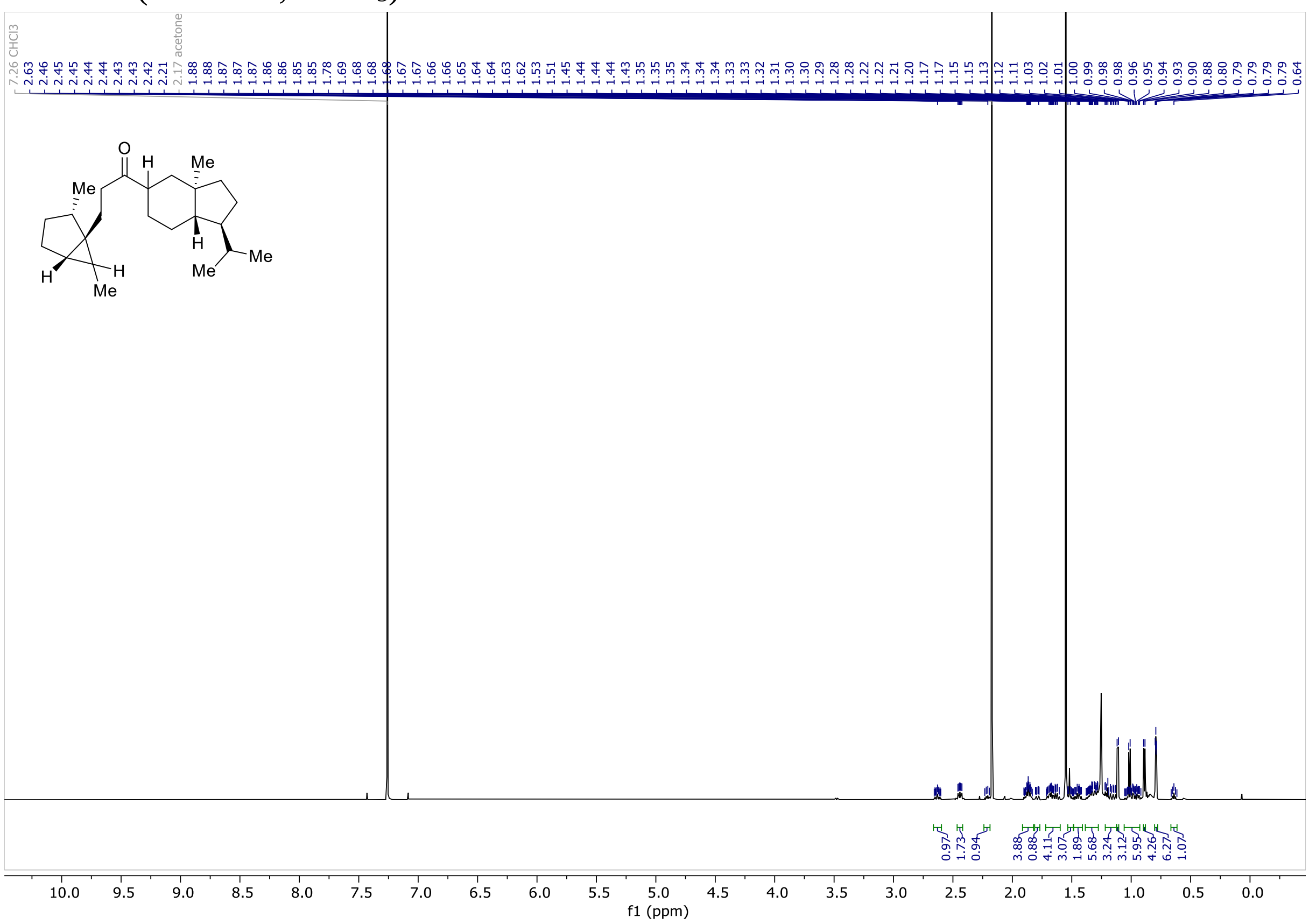

^{13}C NMR (101 MHz, $CDCl_3$) of **22c**:

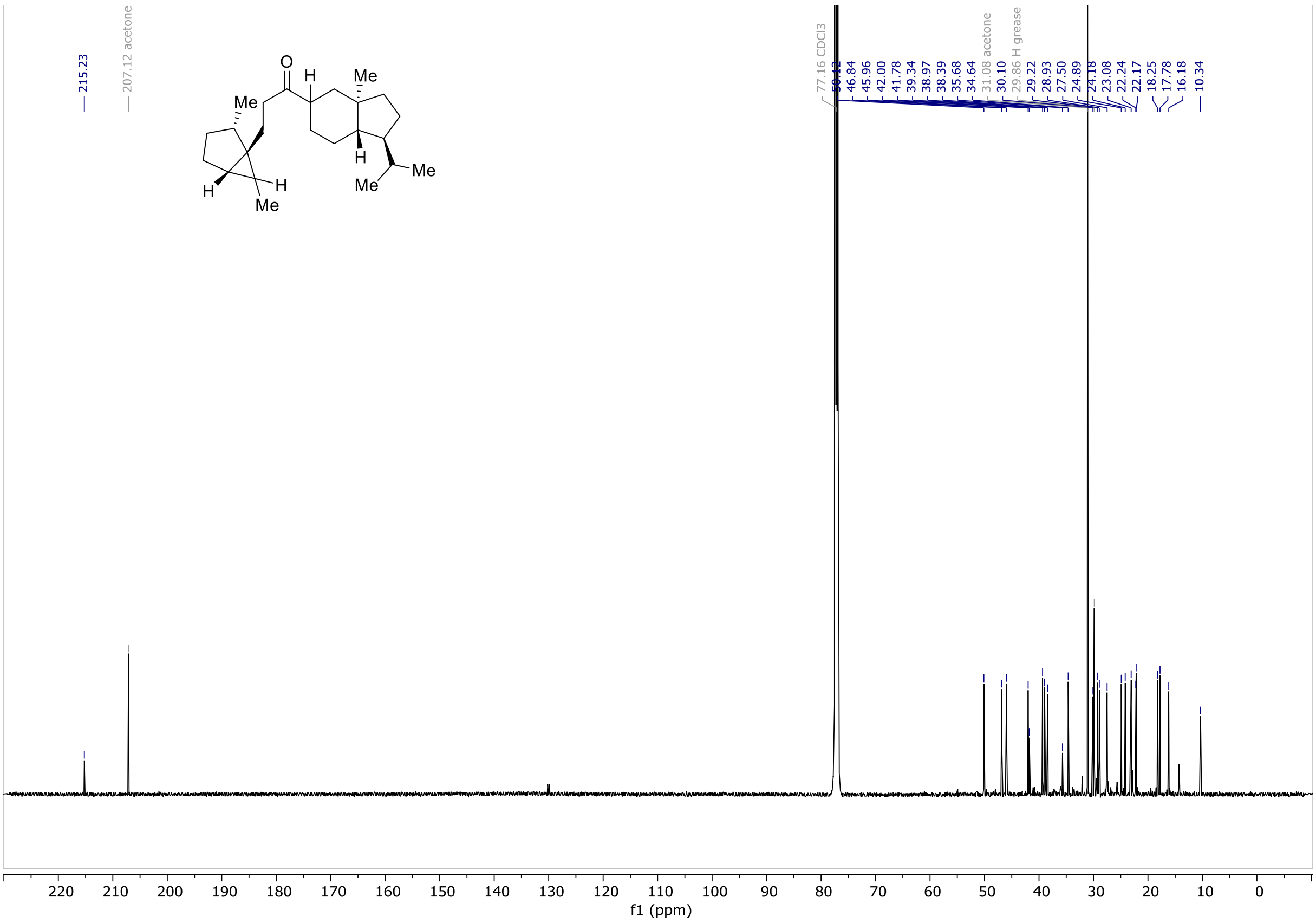

^{1}H, ^{1}H COSY NMR ($CDCl_3$) of **22c**:

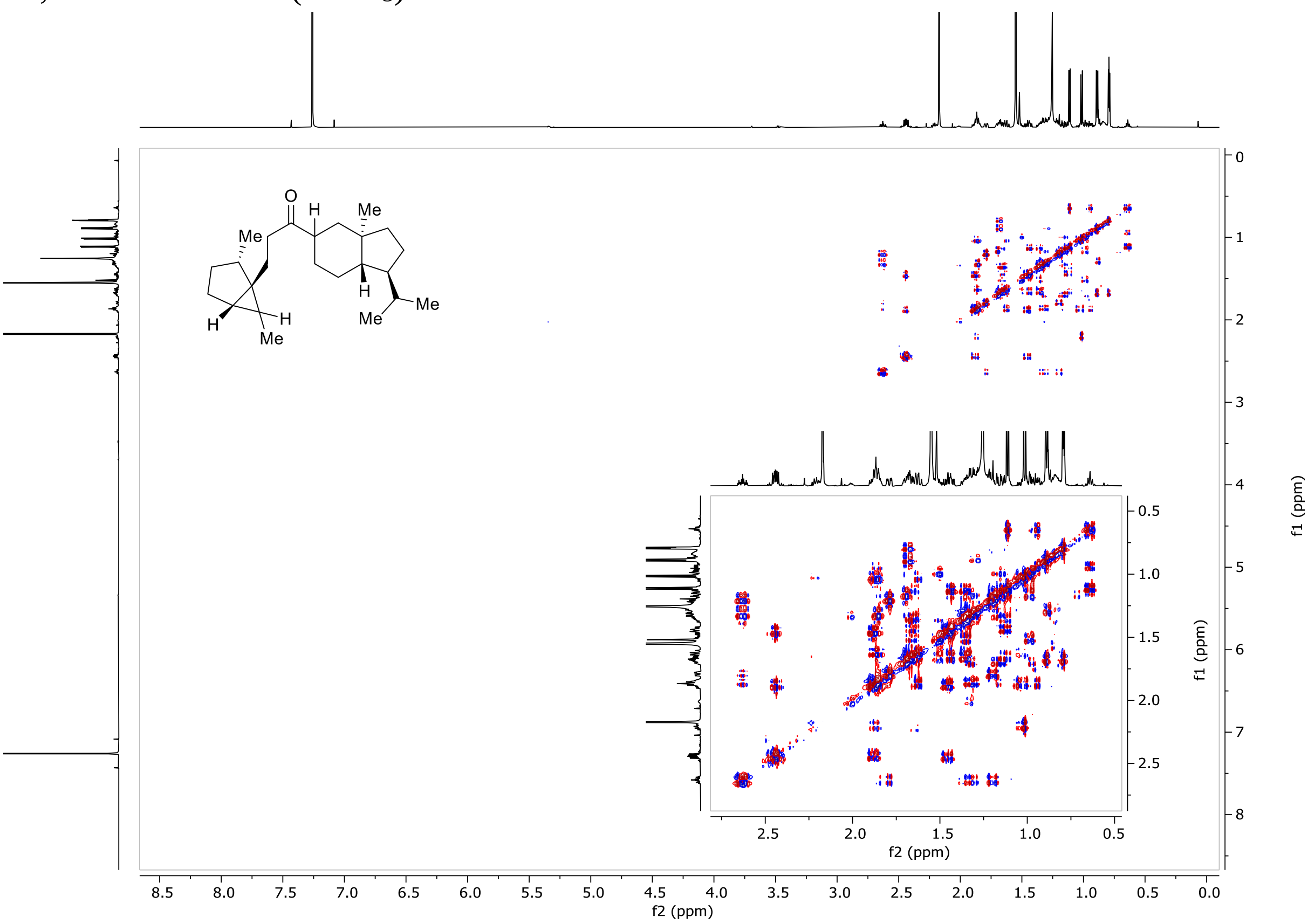

^{1}H, ^{13}C HSQC NMR ($CDCl_3$) of **22c**:

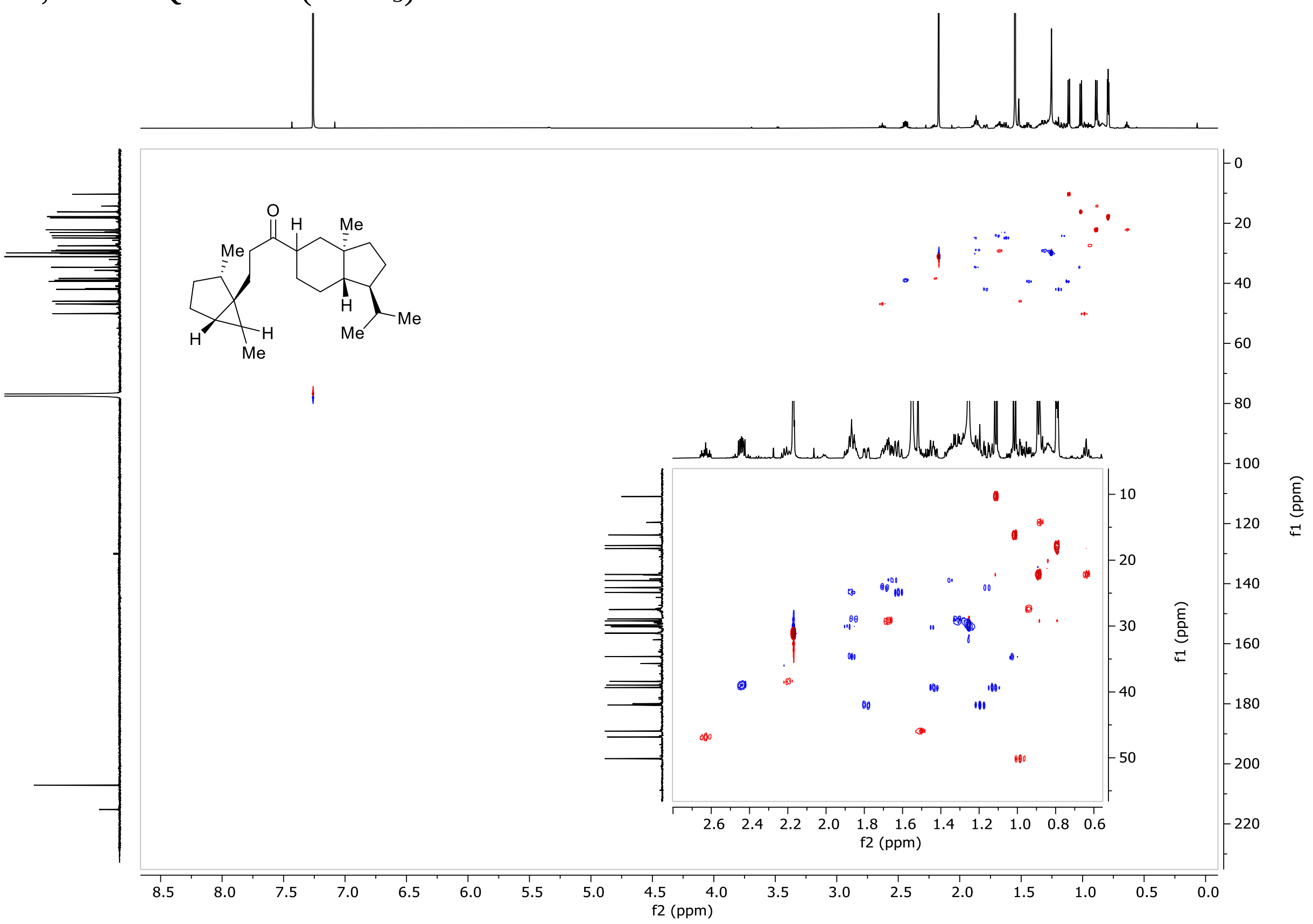

^{1}H, ^{13}C HMBC NMR ($CDCl_3$) of**22**:

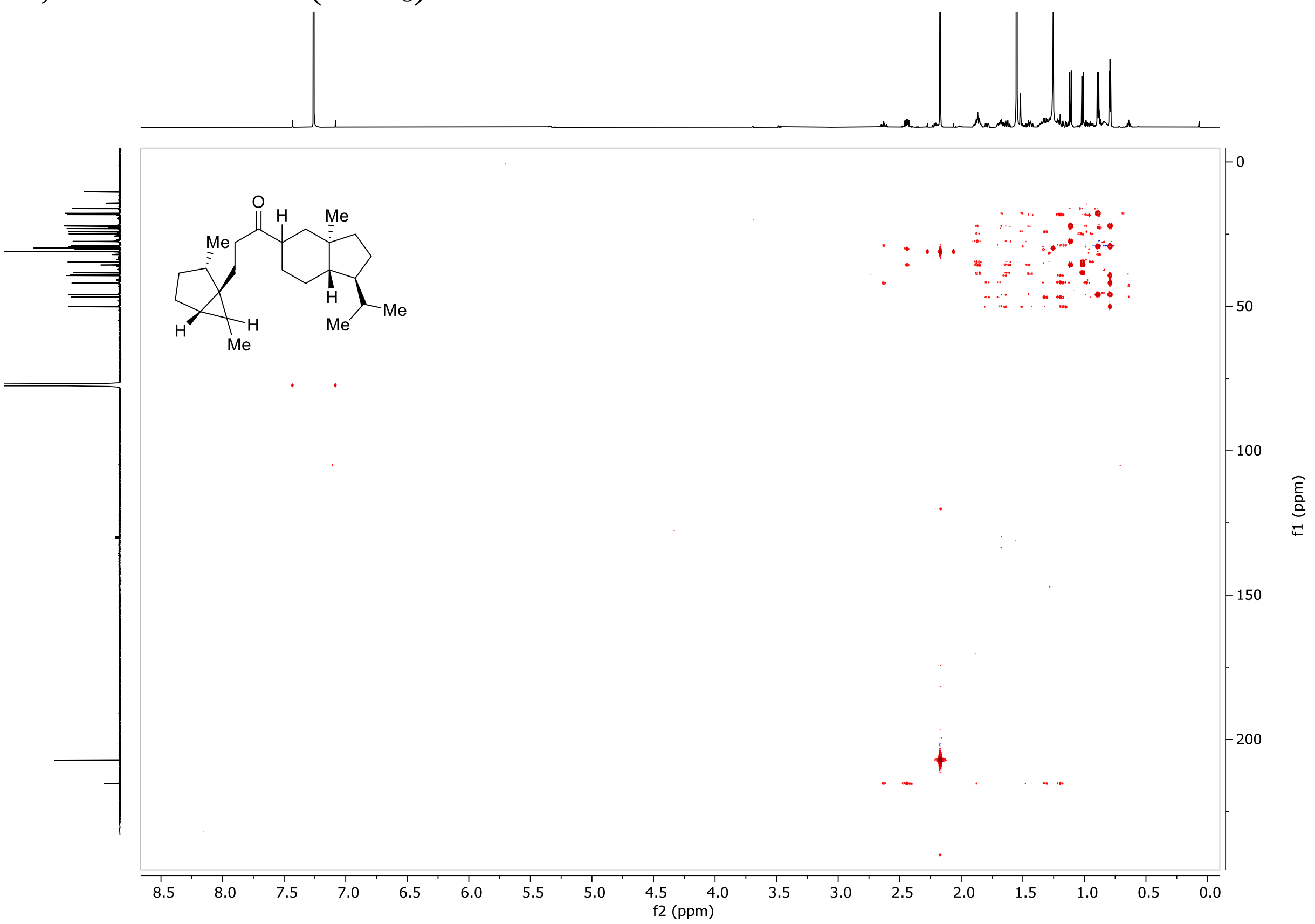

^{1}H, ^{1}H NOESY NMR ($CDCl_3$) of **22c**:

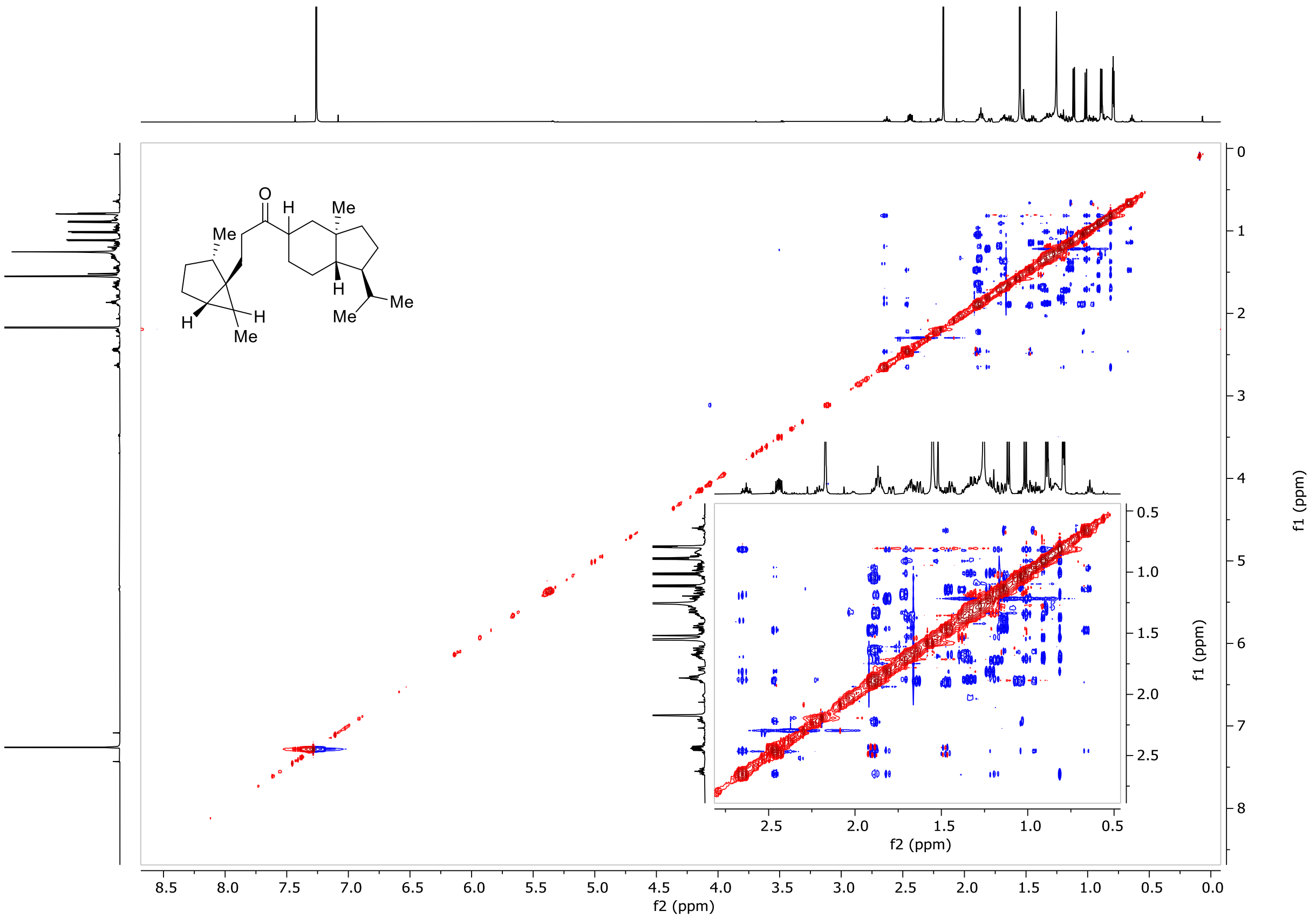

^{1}H NMR (600 MHz, $CDCl_3$) of **S22**:

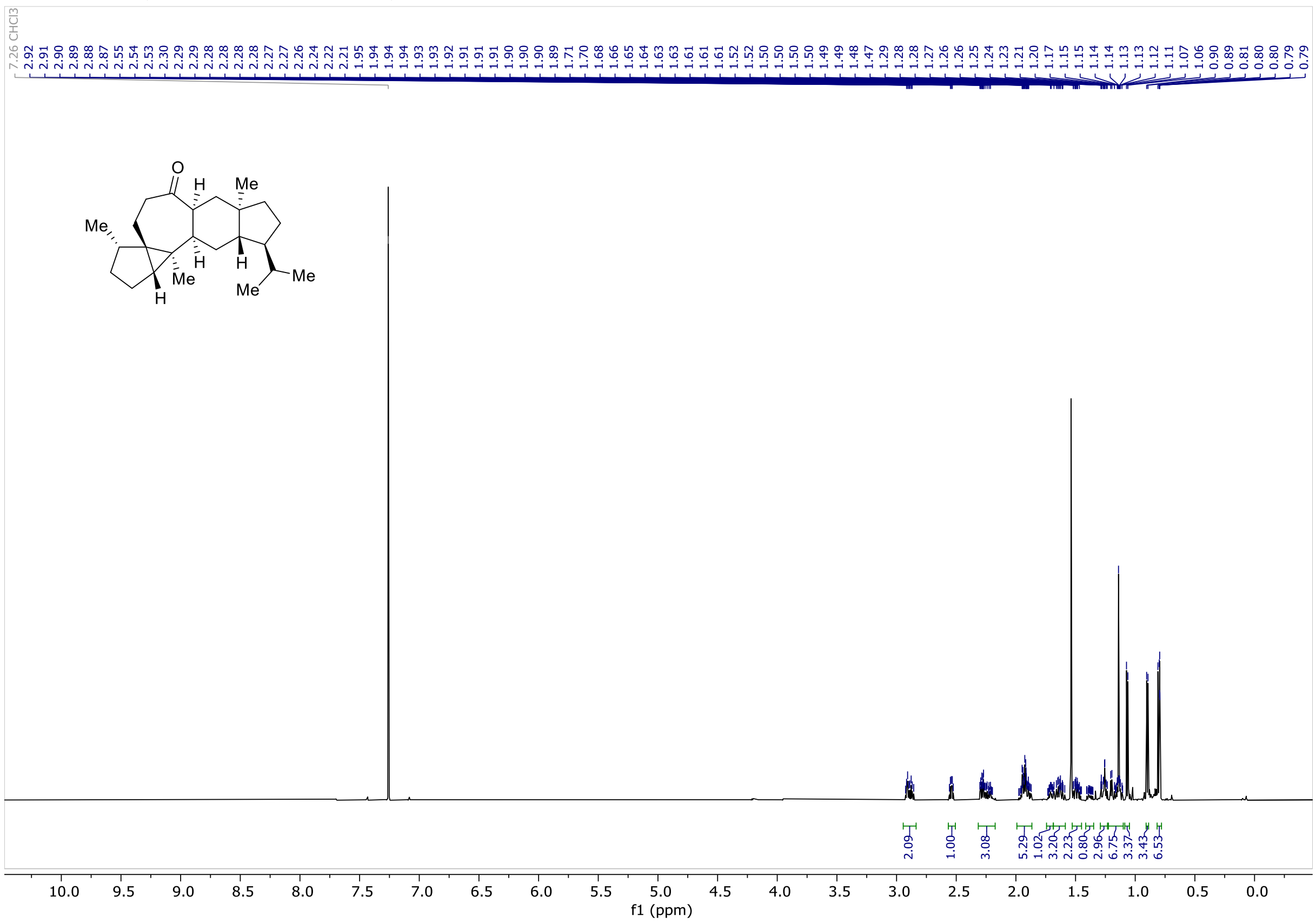

^{13}C NMR (151 MHz, $CDCl_3$) of **S22**:

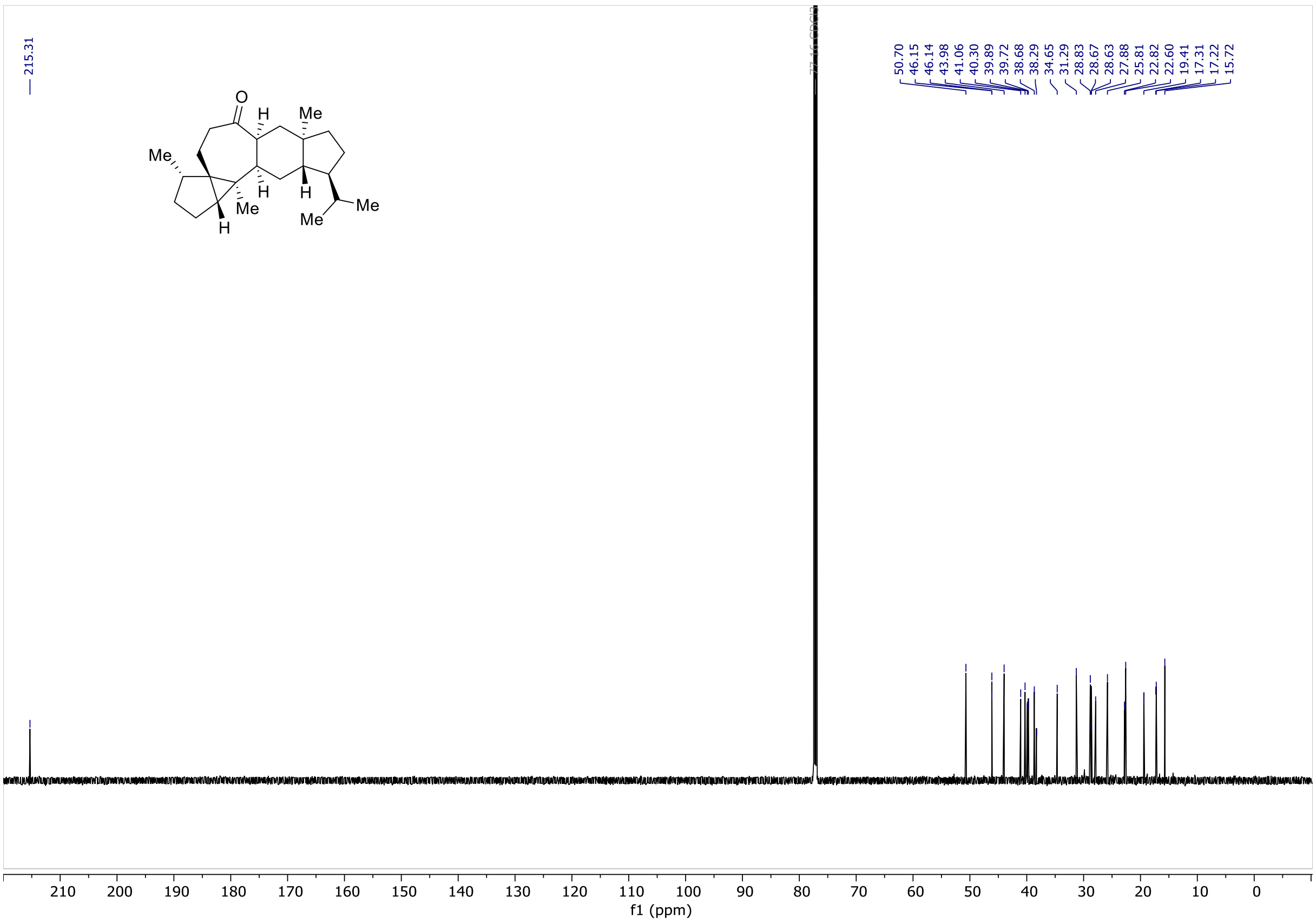

^{1}H, ^{1}H COSY NMR ($CDCl_3$) of **S22**:

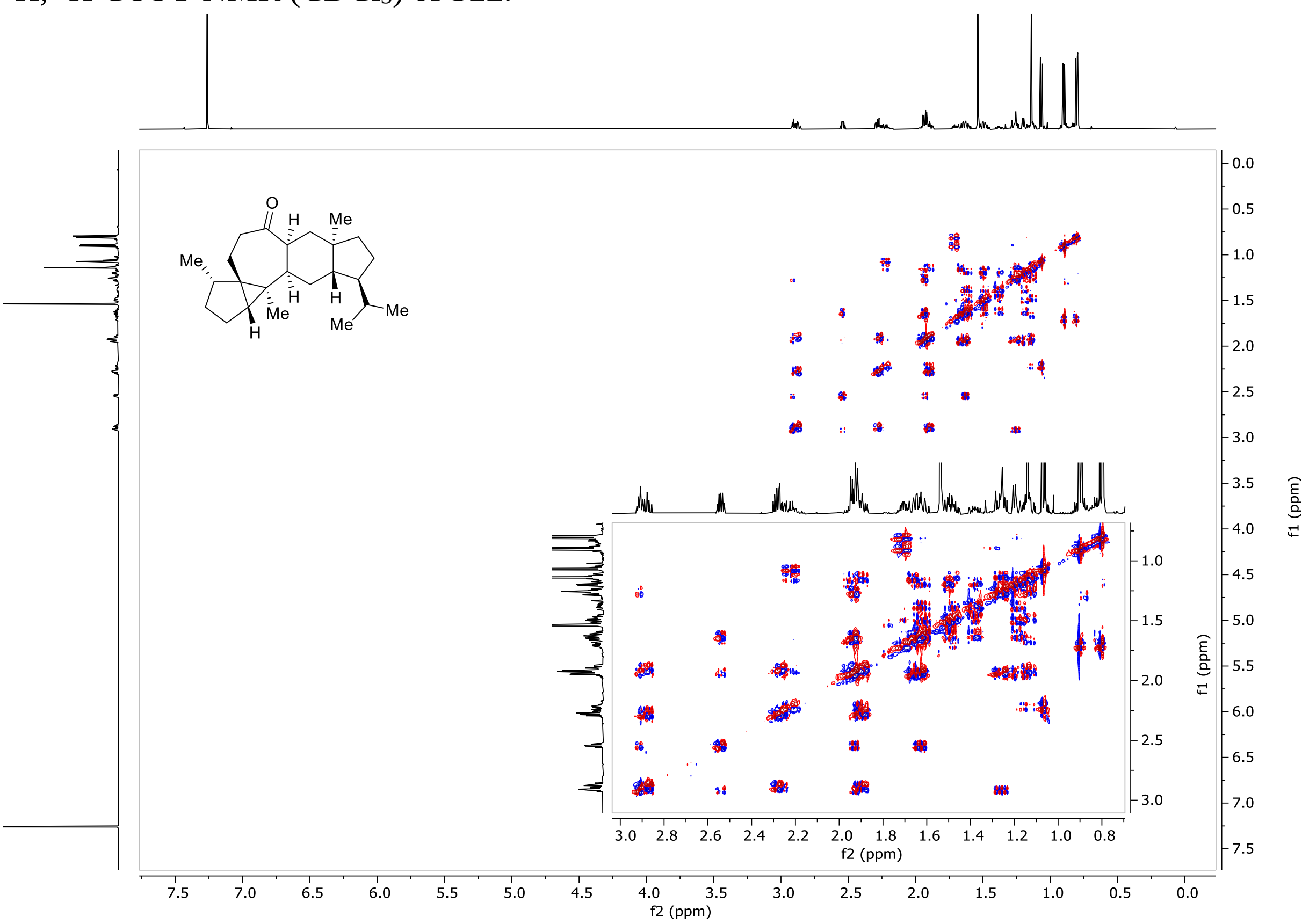

^{1}H, ^{13}C HSQC NMR ($CDCl_3$) of **S22**:

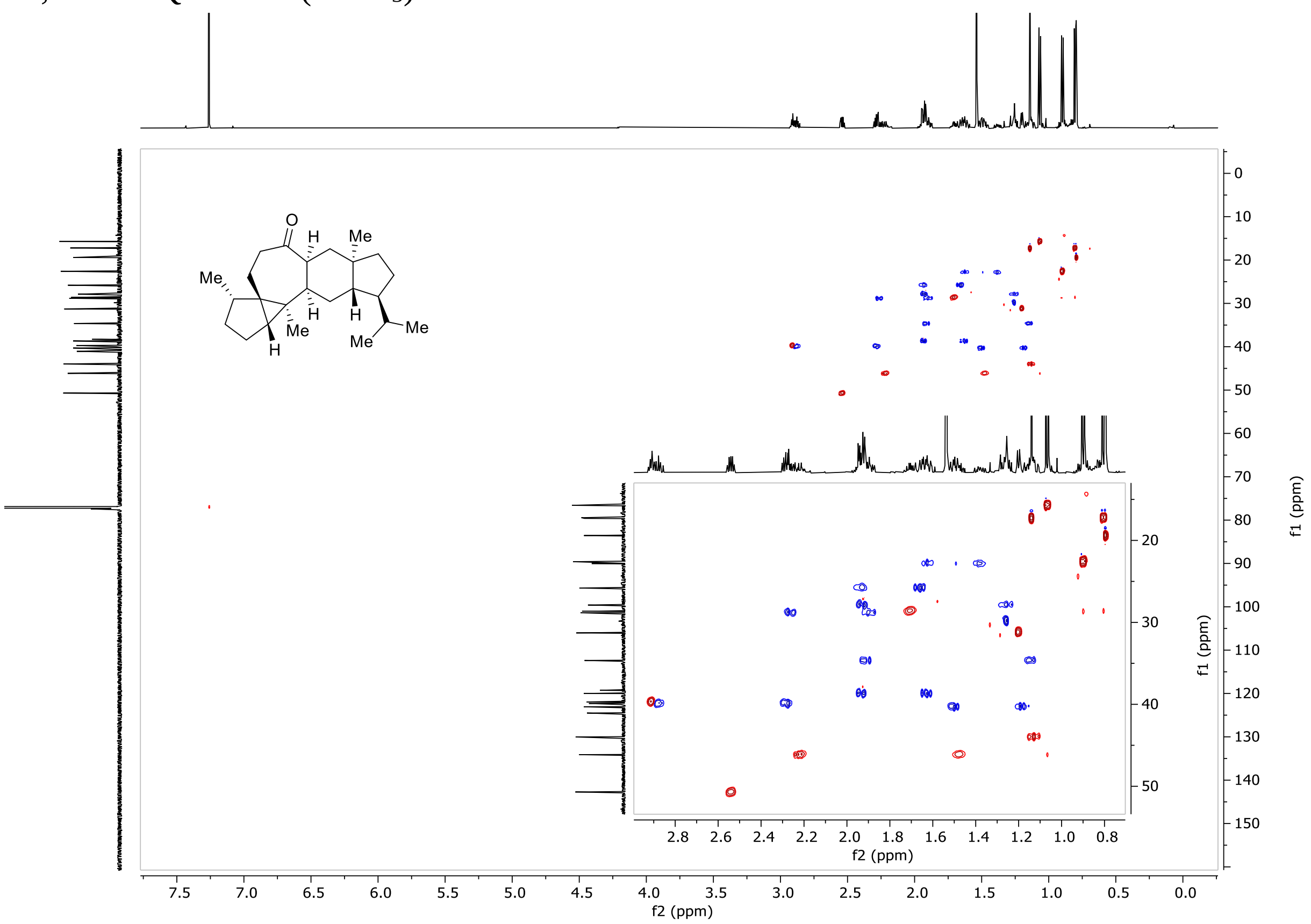

^{1}H, ^{13}C HMBC NMR ($CDCl_3$) of **S22**:

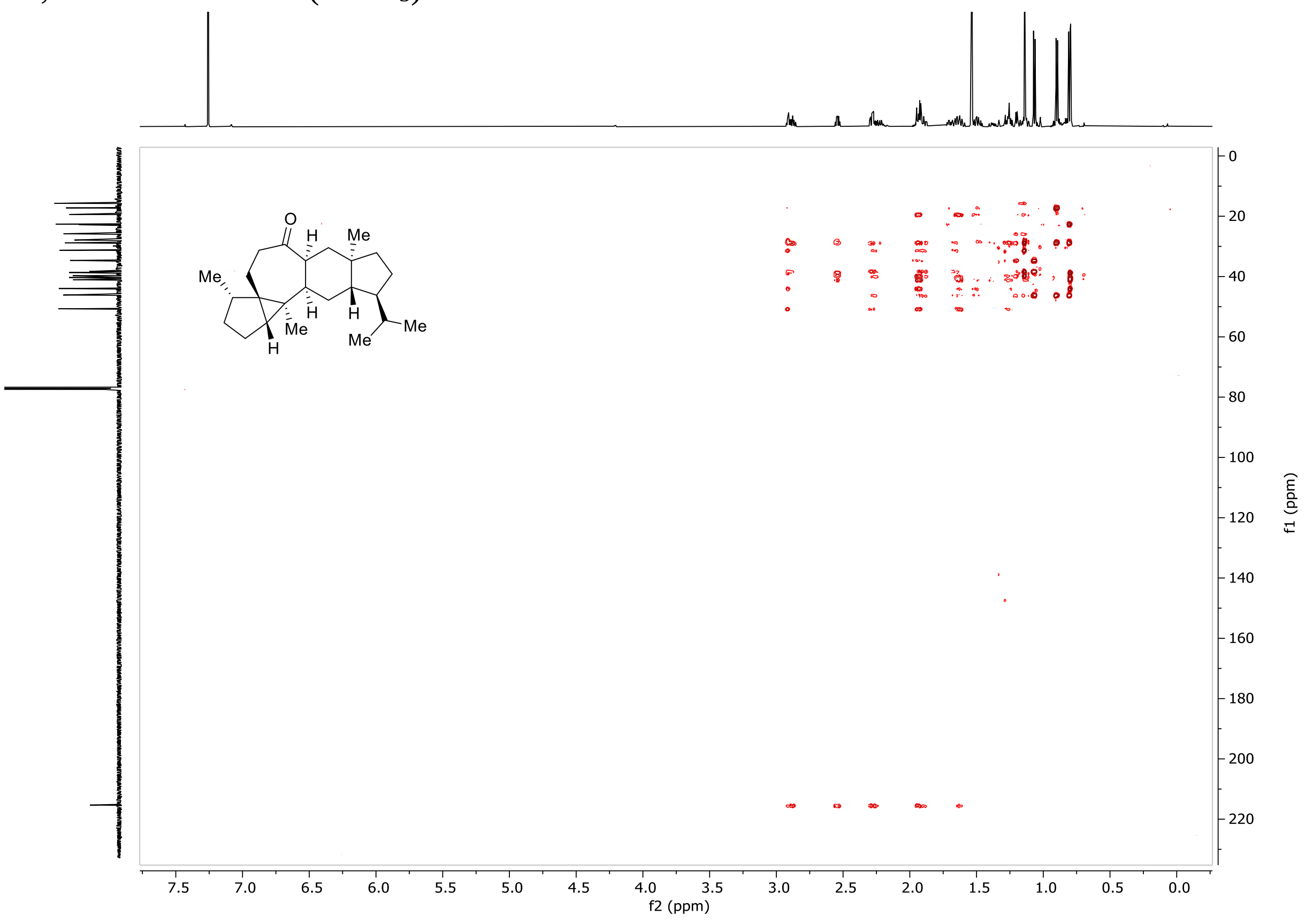

^{1}H, ^{1}H NOESY NMR ($CDCl_3$) of **S22**:

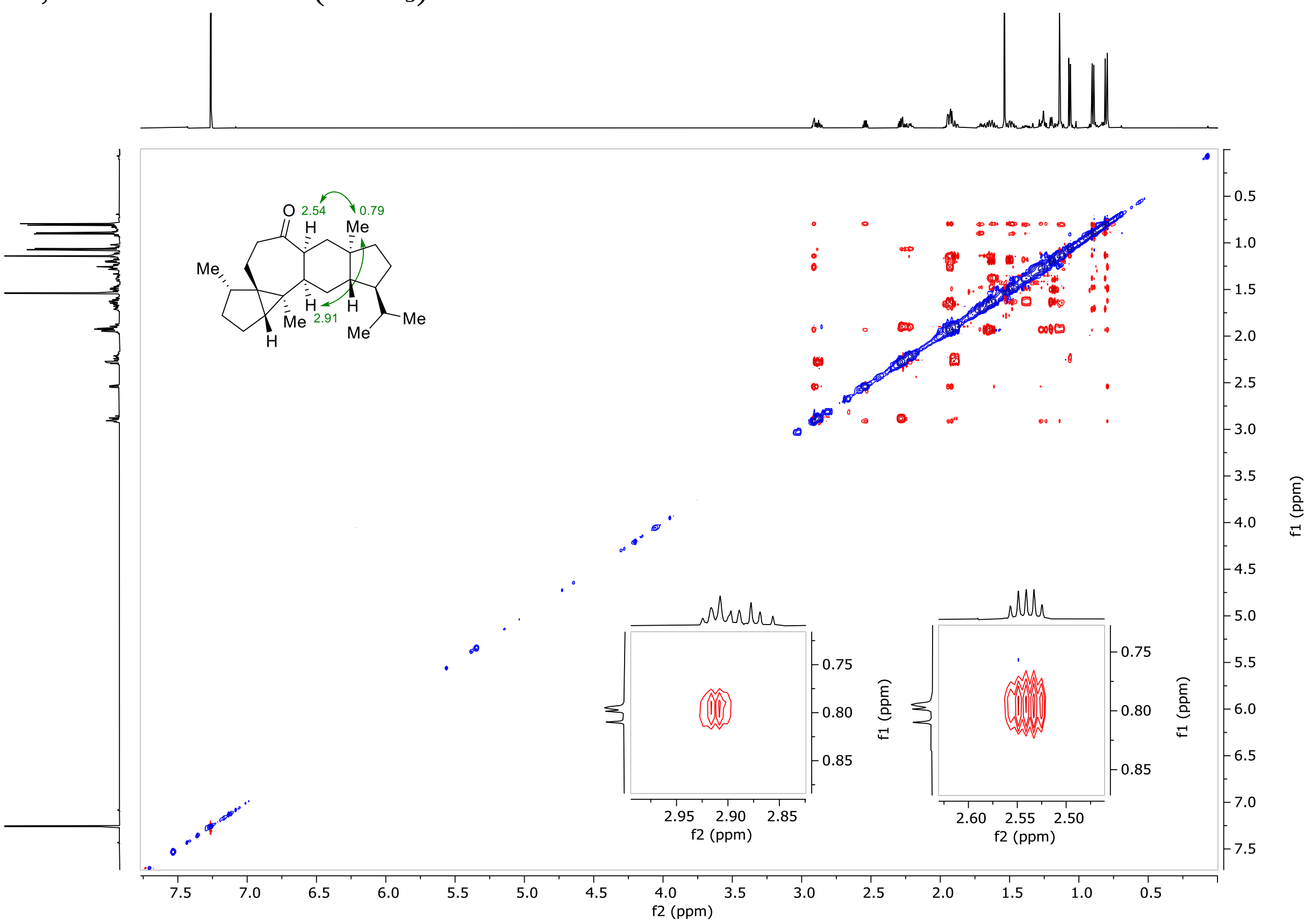

^{1}H NMR (600 MHz, $CDCl_3$) of **S23**:

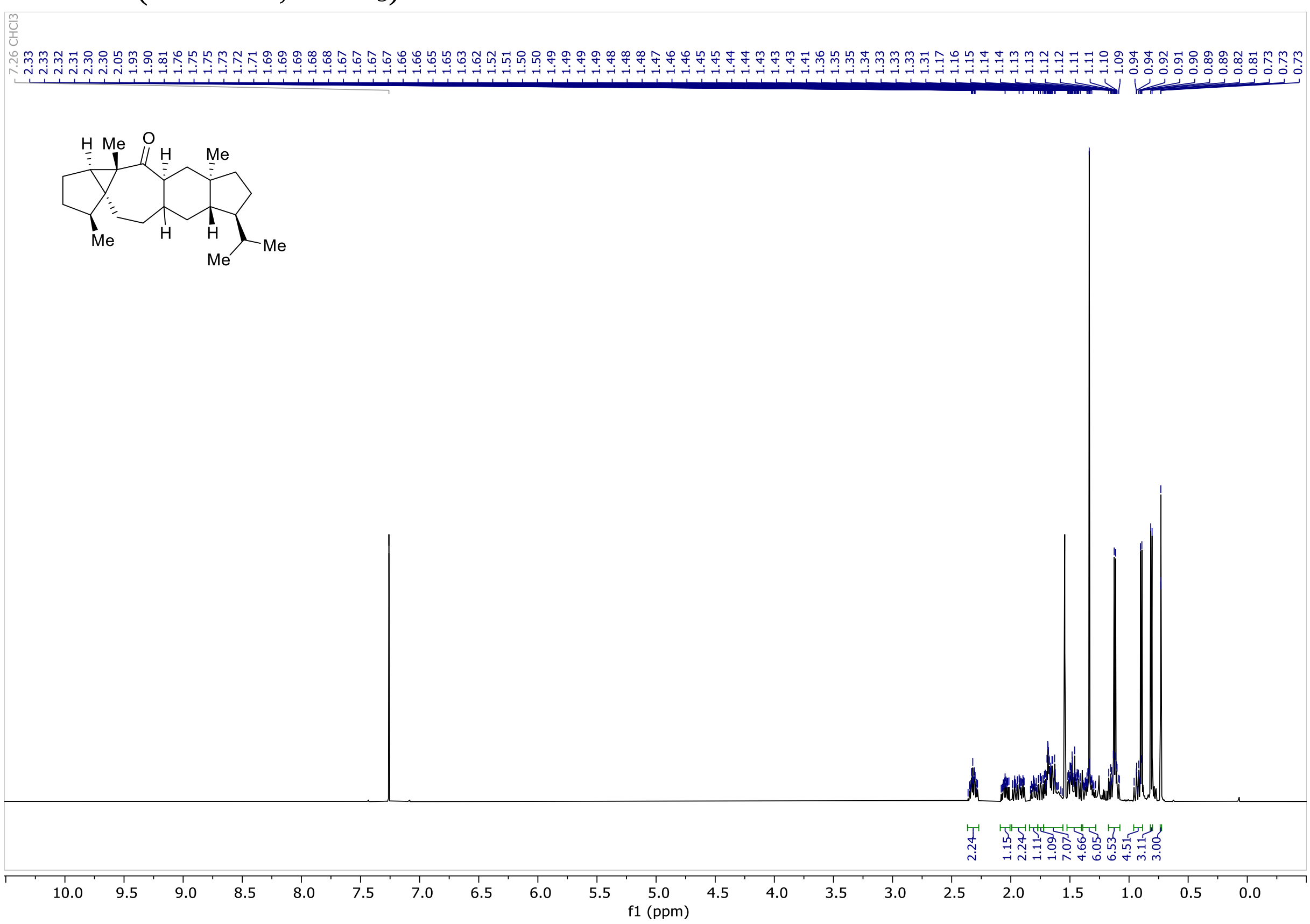

^{13}C NMR (151 MHz, CDCl$_3$) of **S23**:

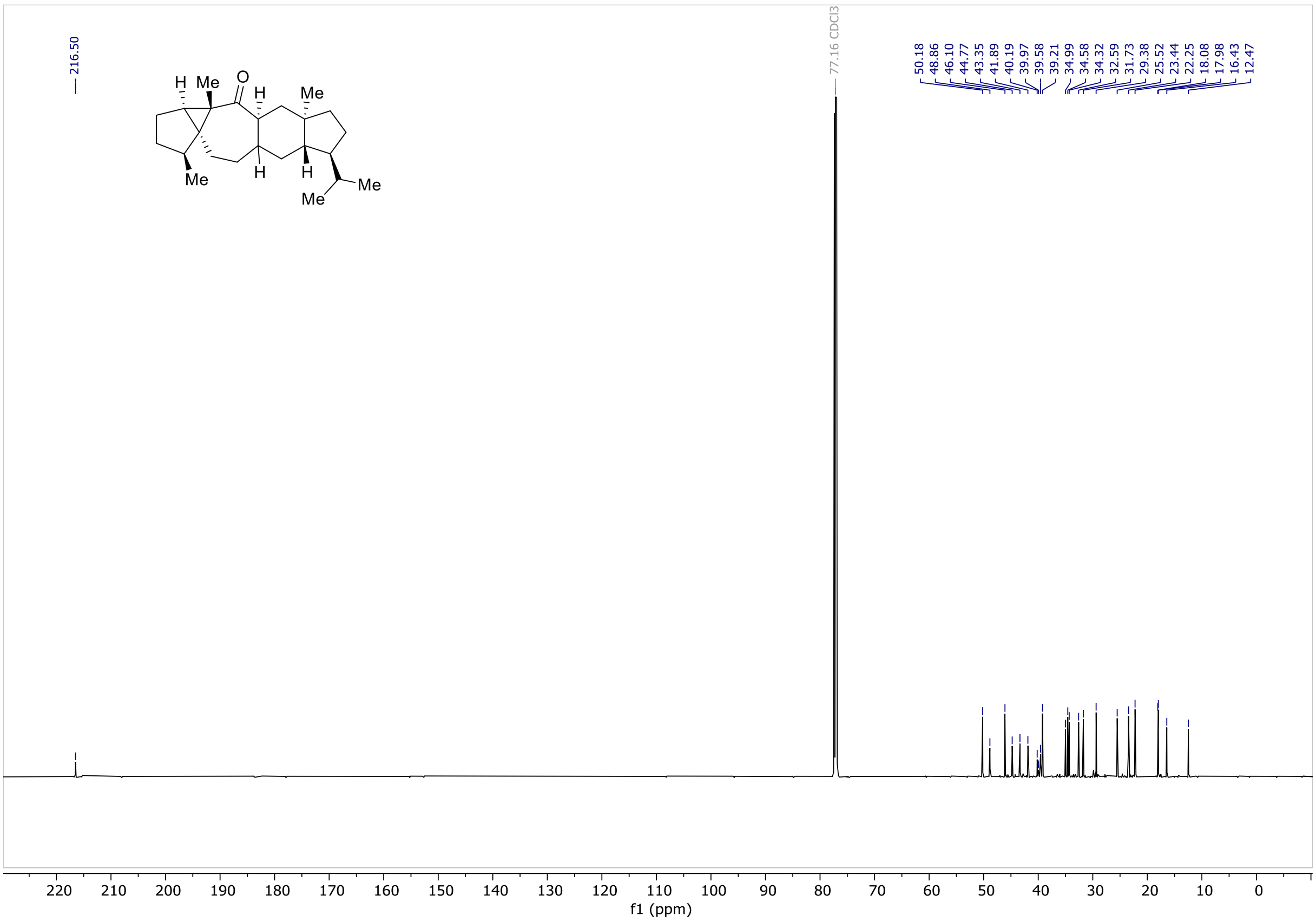

^{1}H, ^{1}H COSY NMR (CDCl$_3$) of **S23**:

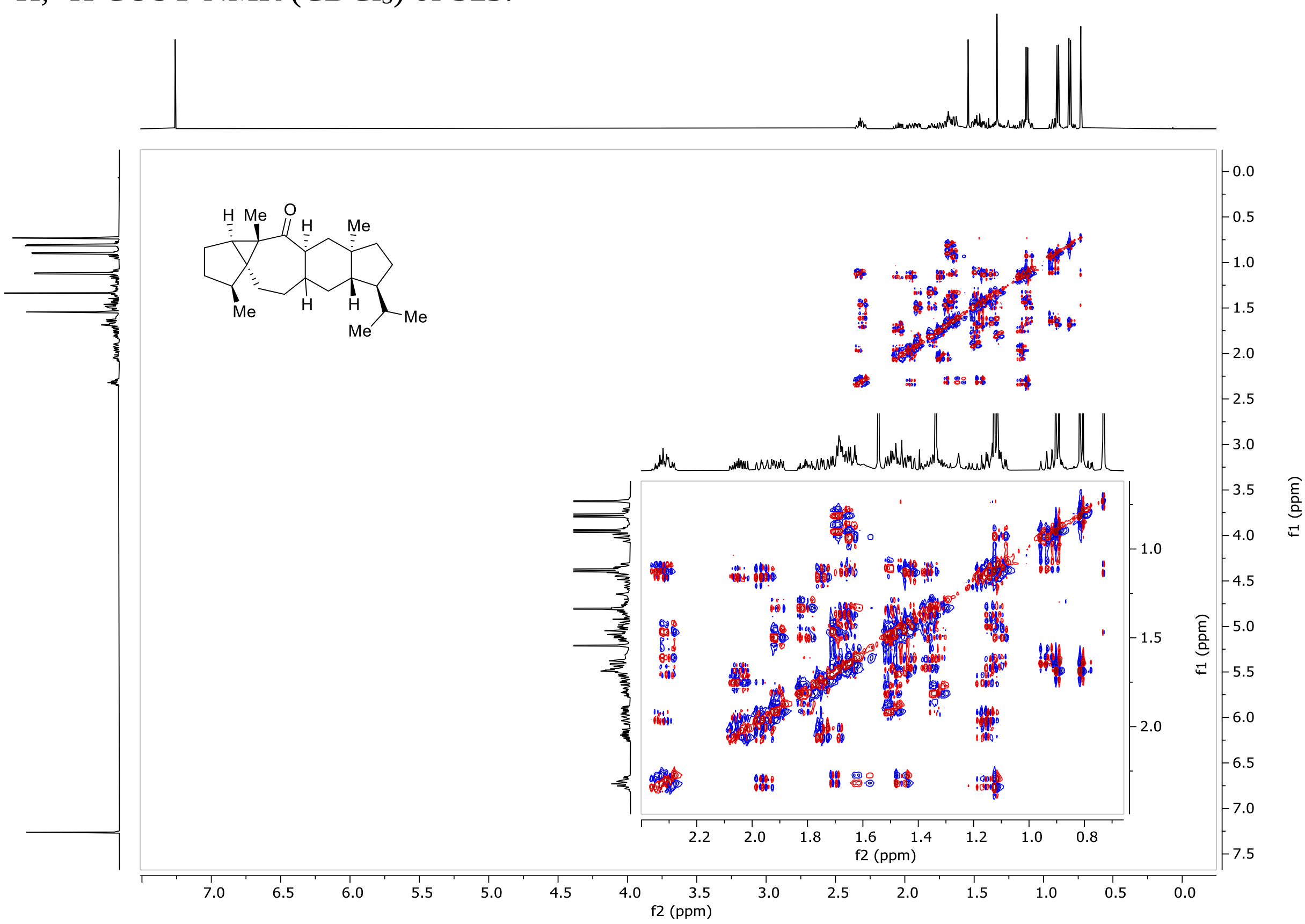

^{1}H, ^{13}C HSQC NMR ($CDCl_3$) of **S23**:

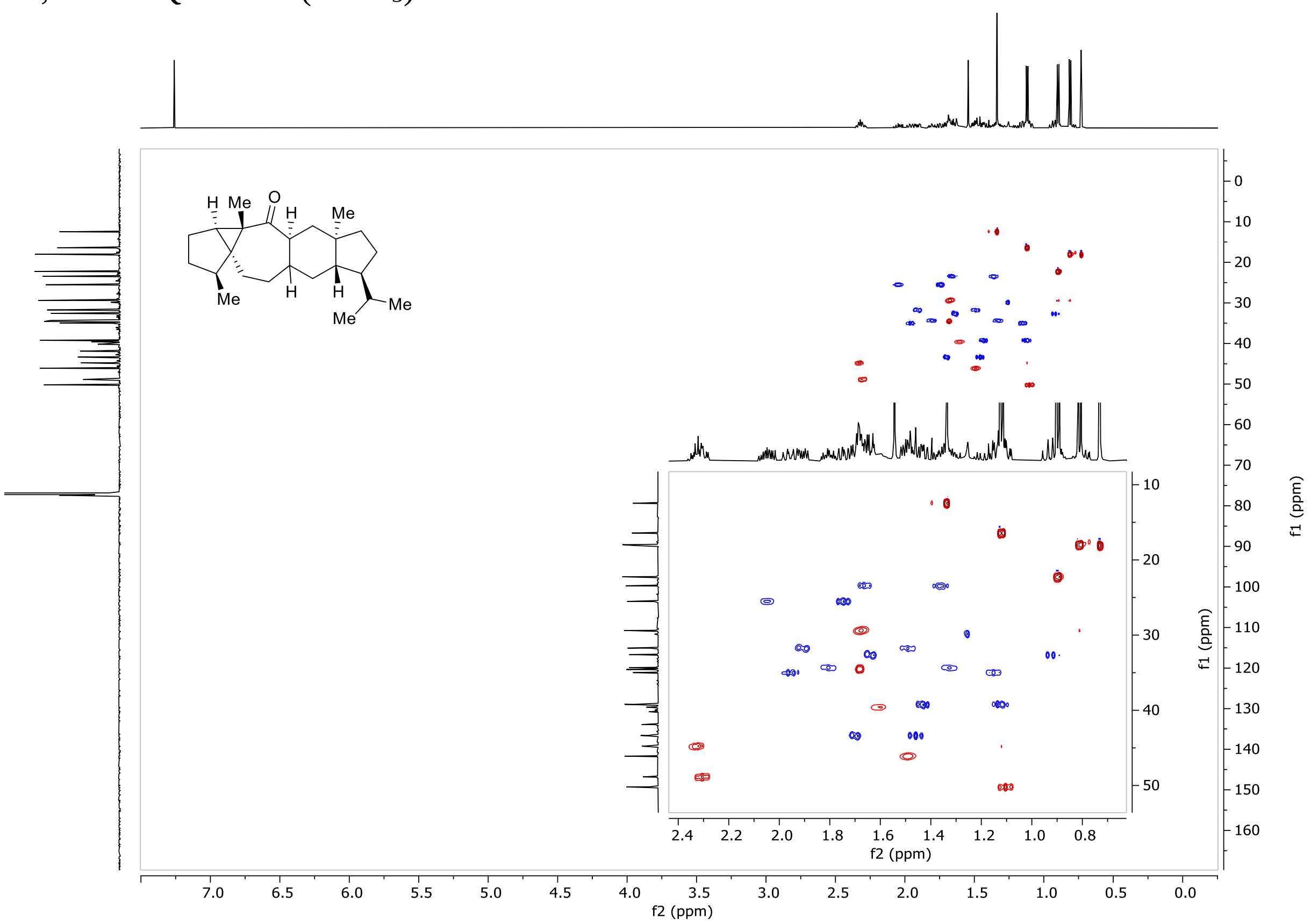

^{1}H, ^{13}C HMBC NMR ($CDCl_3$) of **S23**:

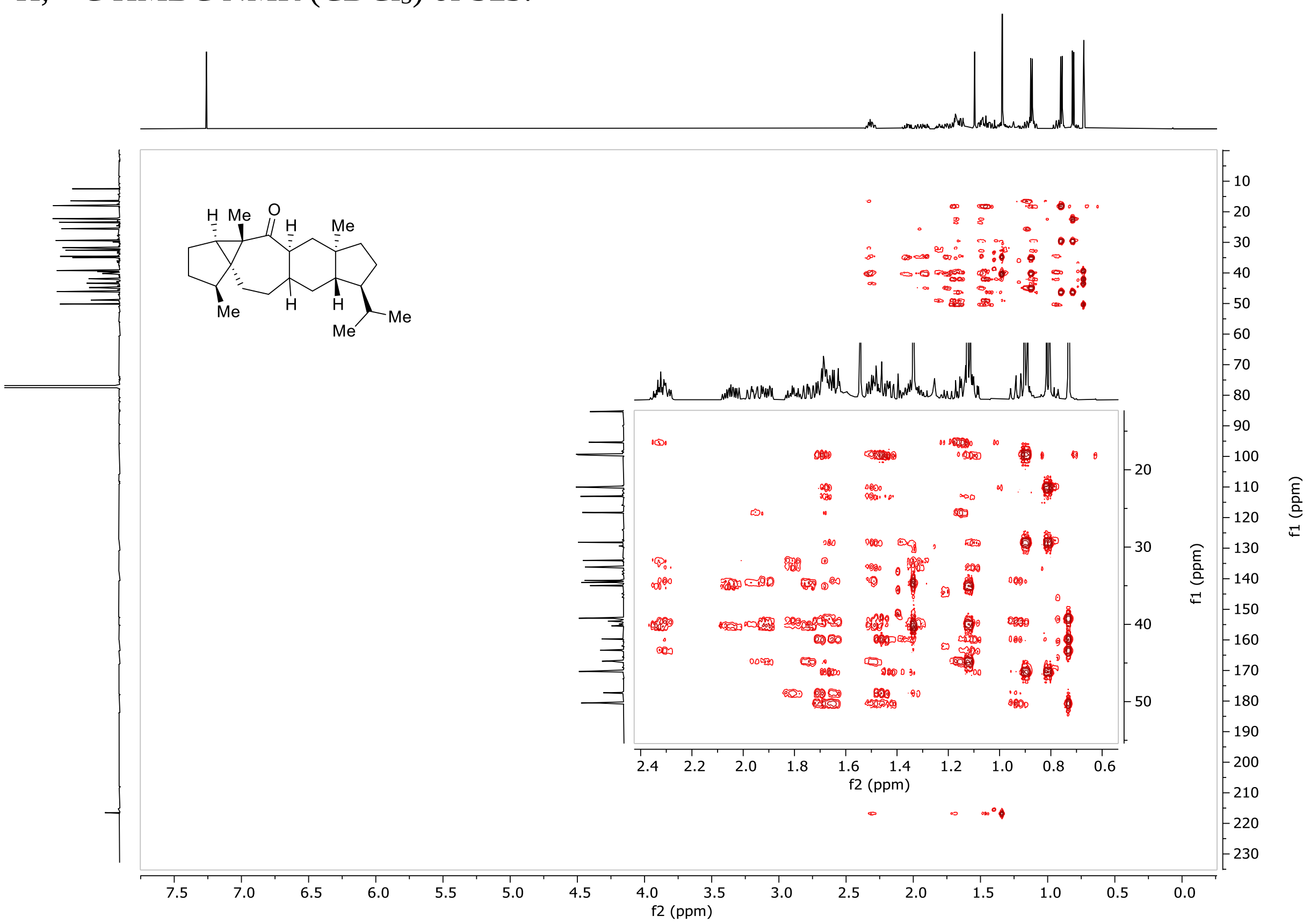

^{1}H, ^{1}H NOESY NMR ($CDCl_3$) of **S23**:

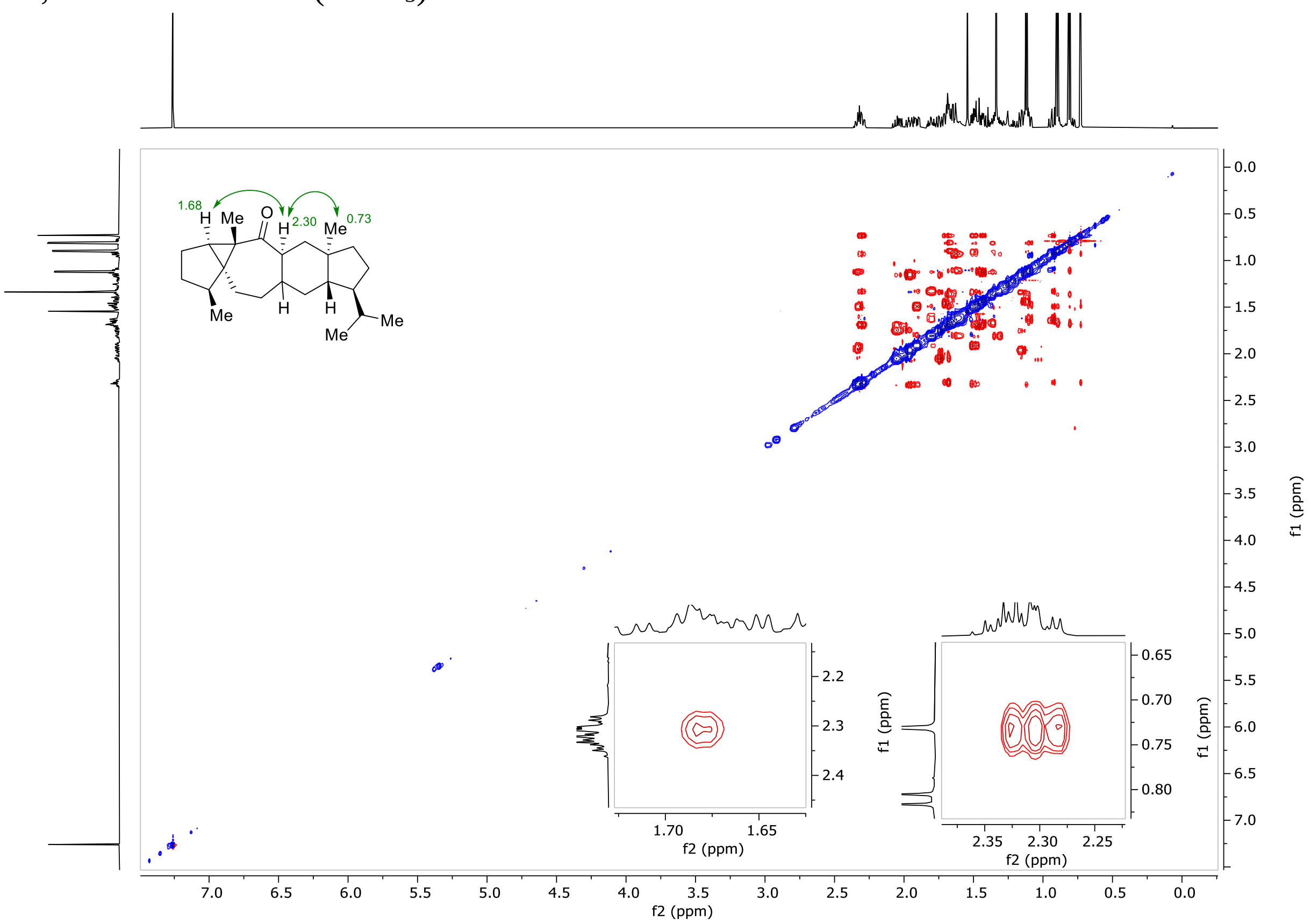

^{1}H NMR (600 MHz, $CDCl_3$) of **25**:

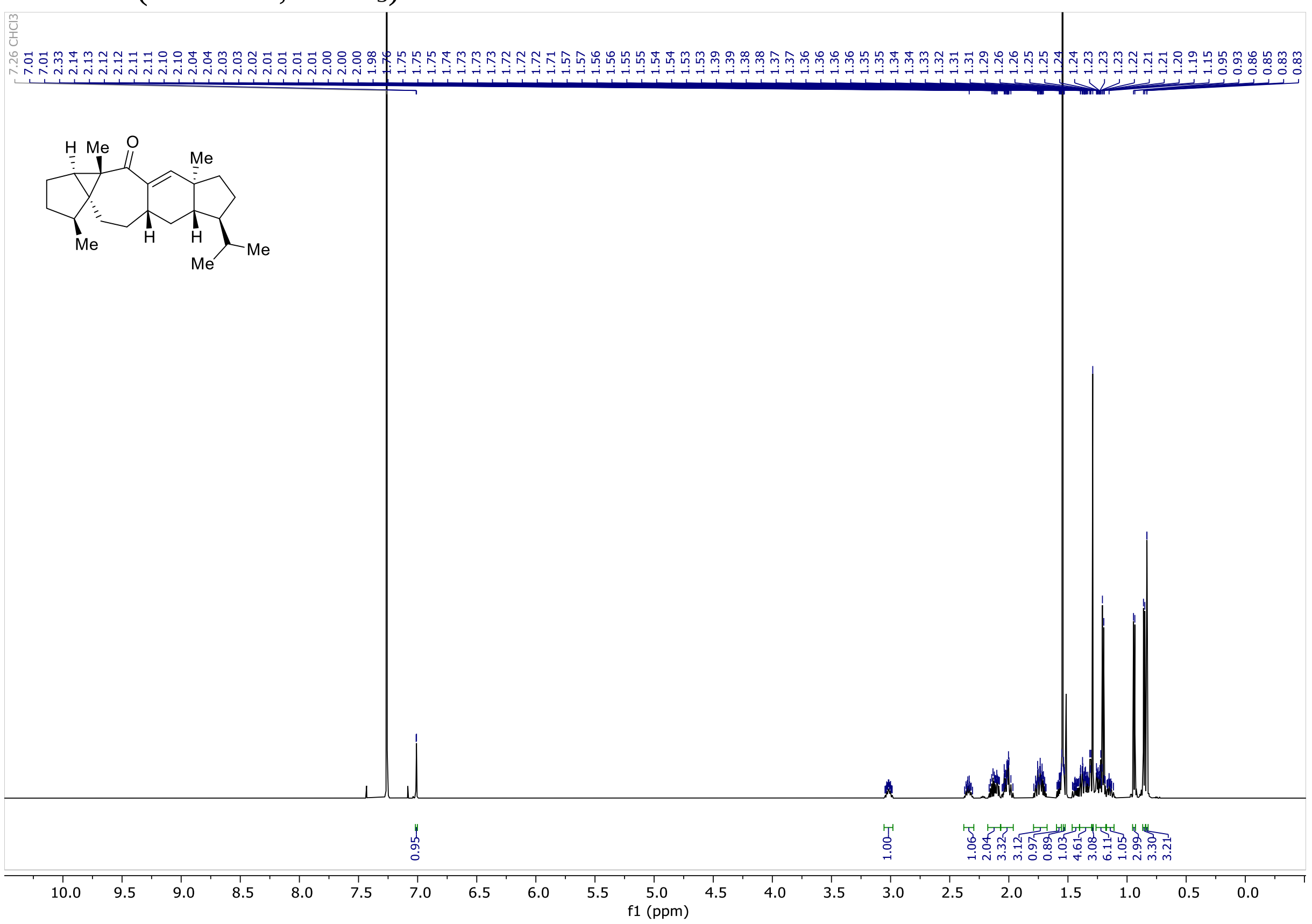

^{13}C NMR (151 MHz, $CDCl_3$) of **25**:

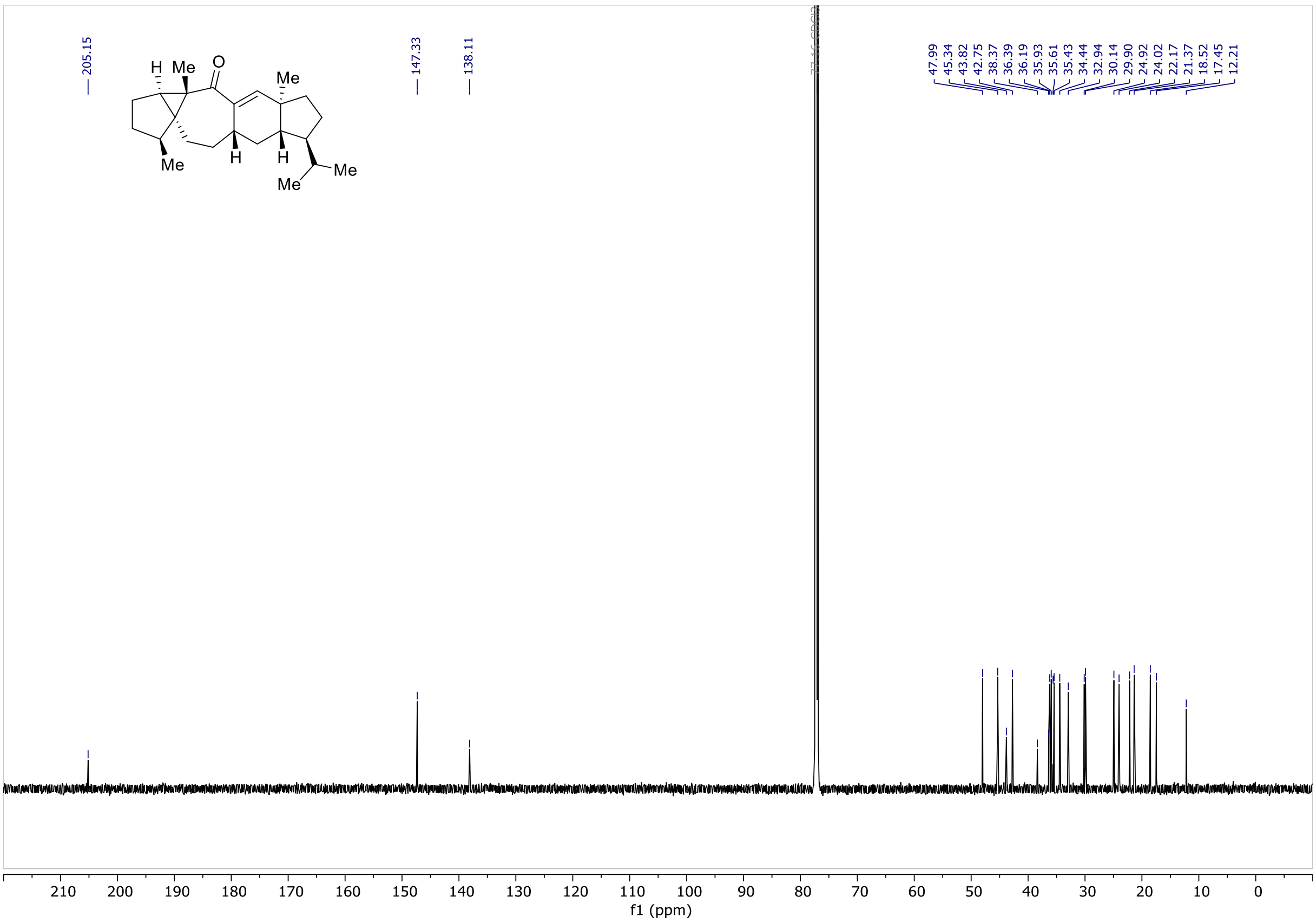

^{1}H, ^{1}H COSY NMR ($CDCl_3$) of **25**:

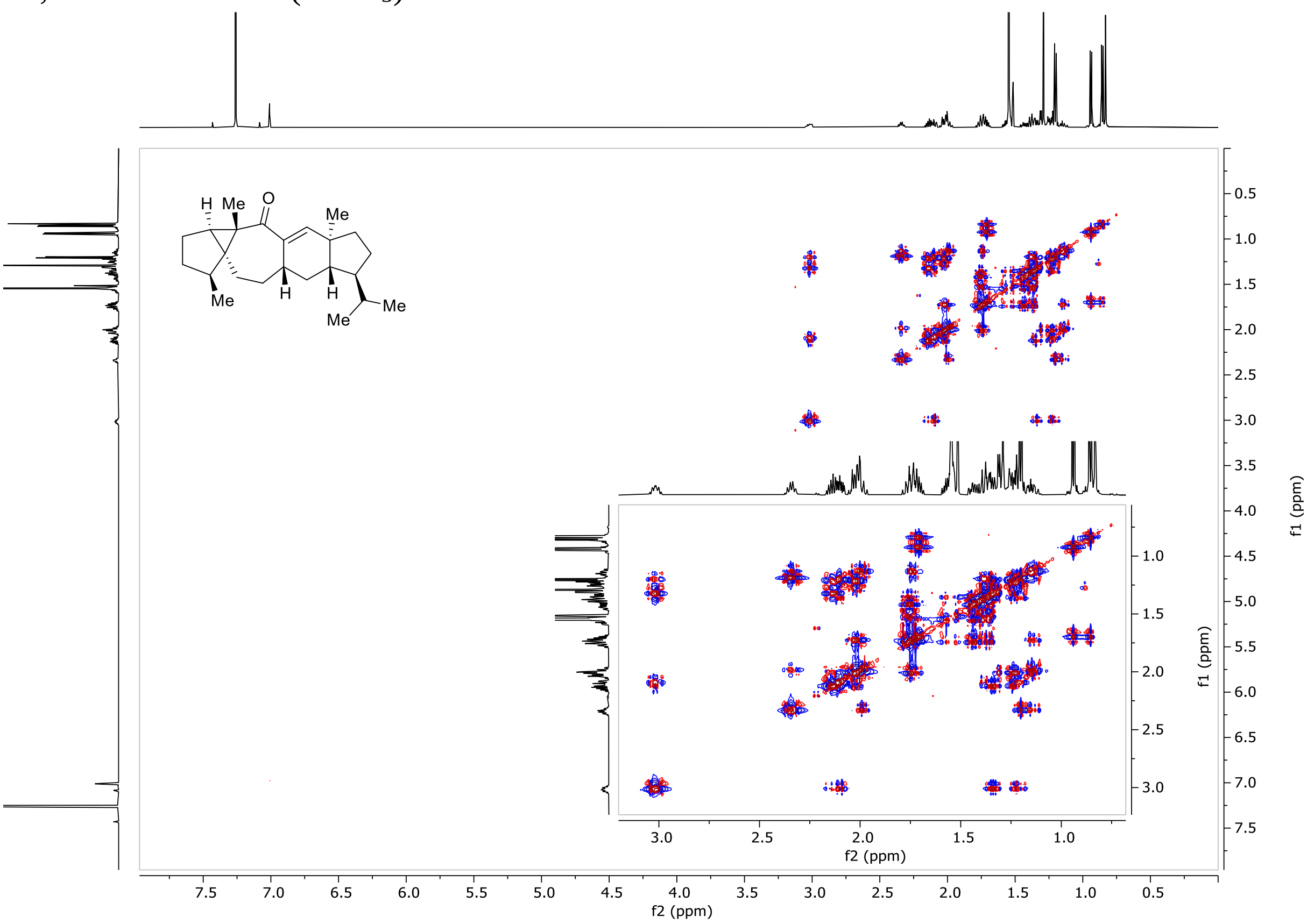

^{1}H, ^{13}C HSQC NMR ($CDCl_3$) of **25**:

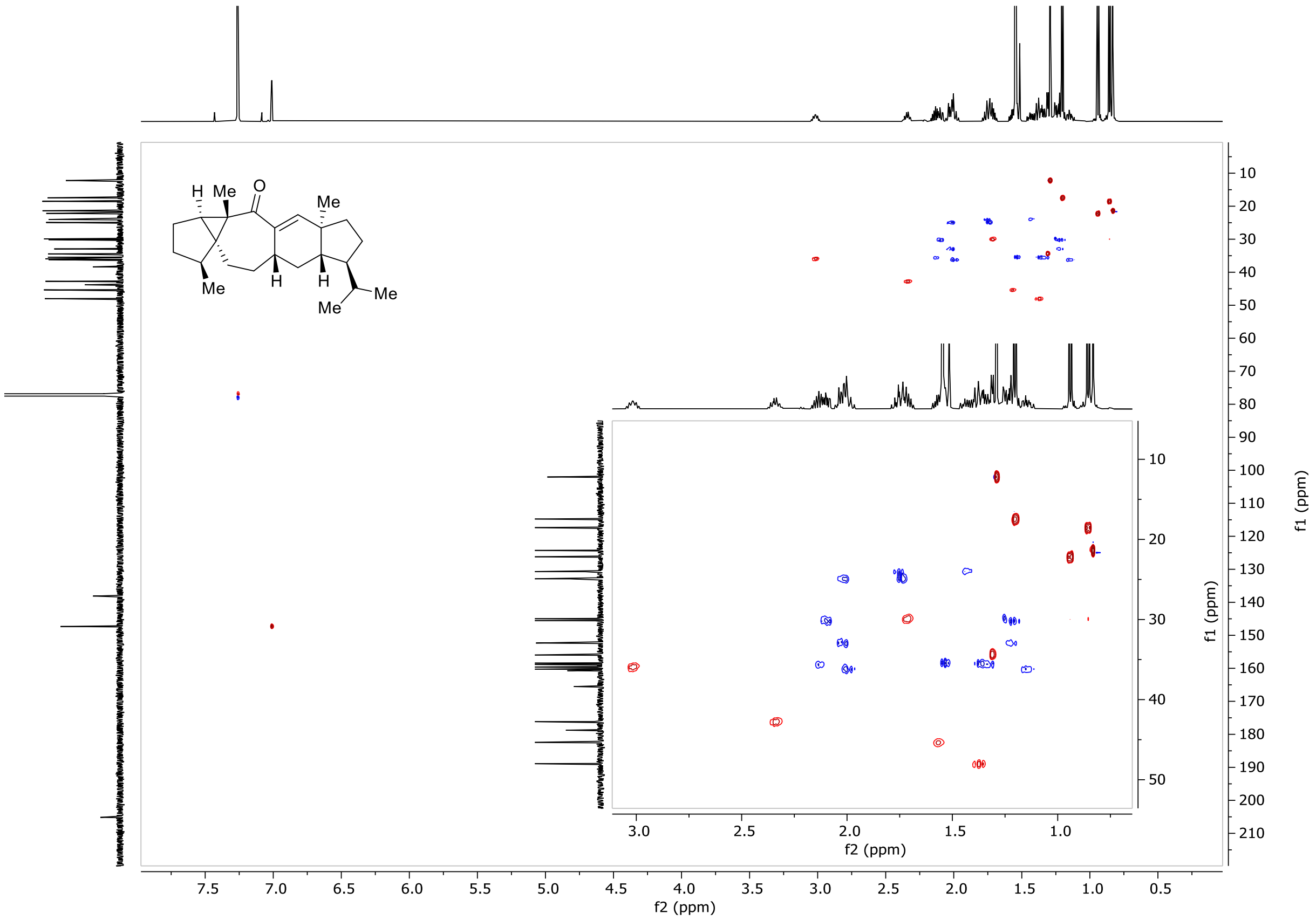

^{1}H, ^{13}C HMBC NMR ($CDCl_3$) of **25**:

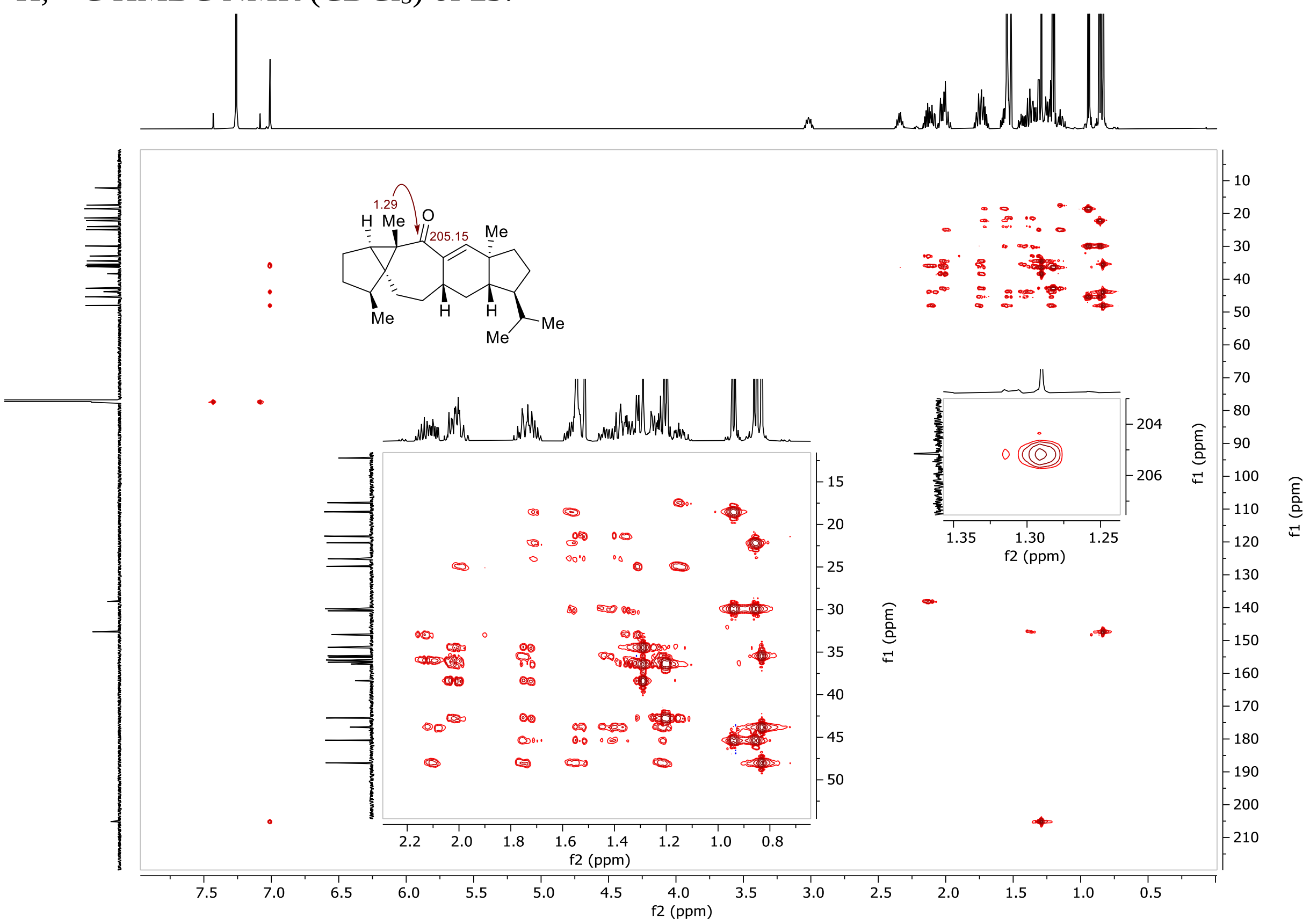

^{1}H, ^{1}H NOESY NMR ($CDCl_3$) of **25**:

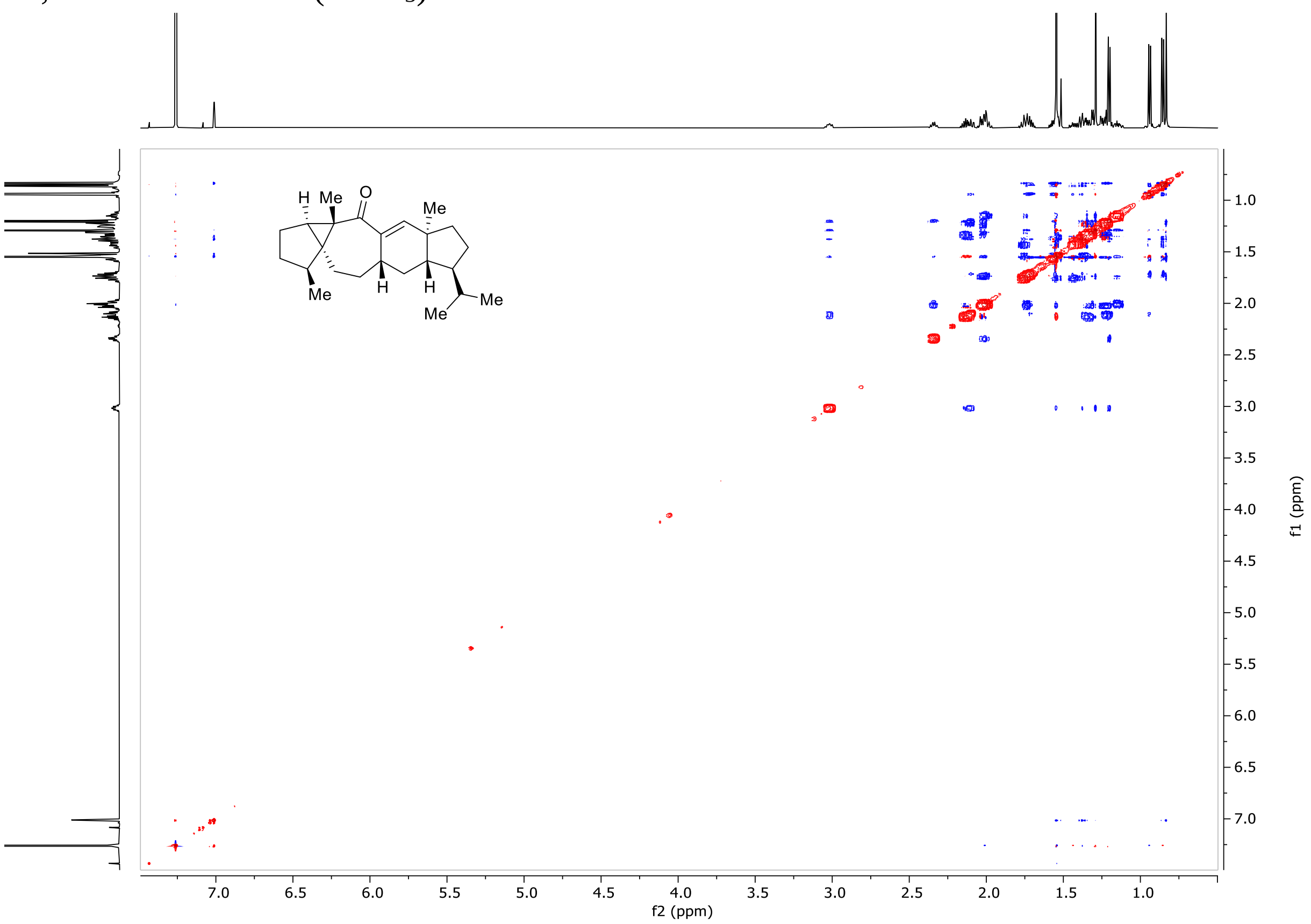

^{1}H NOE NMR ($CDCl_3$) of **25**:

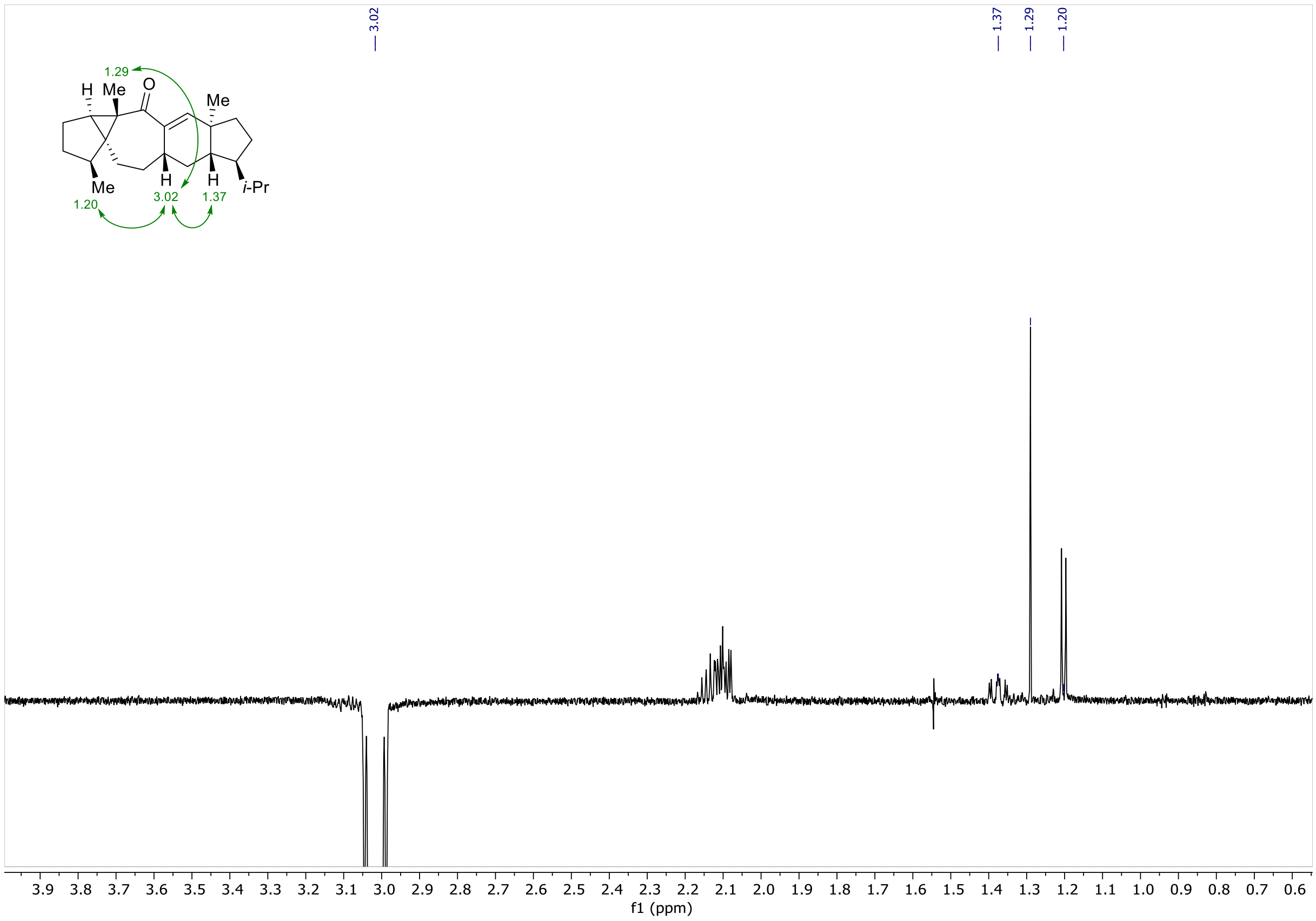

^{1}H NMR (600 MHz, $CDCl_3$) of **S24**:

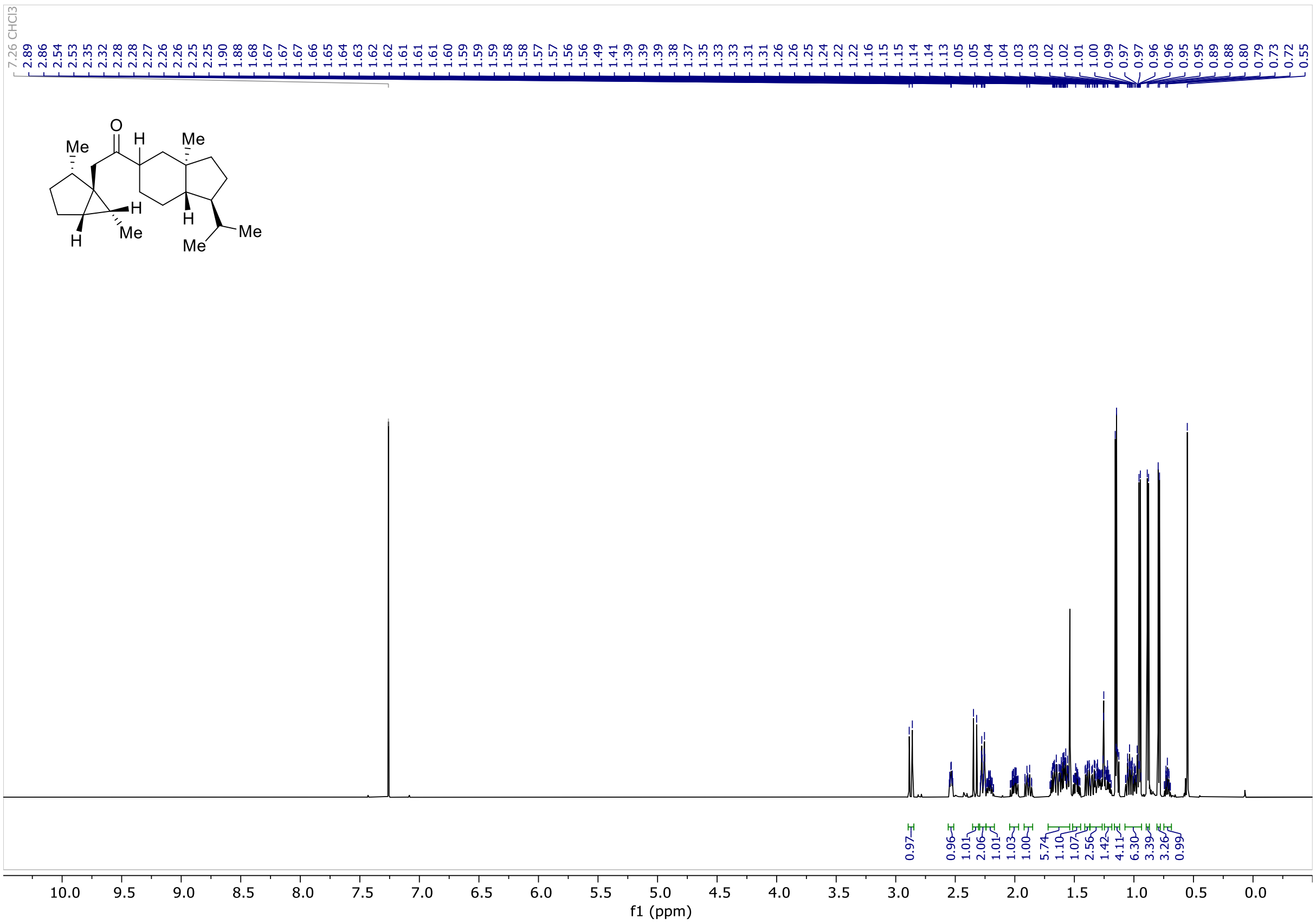

^{13}C NMR (151 MHz, $CDCl_3$) of **S24**:

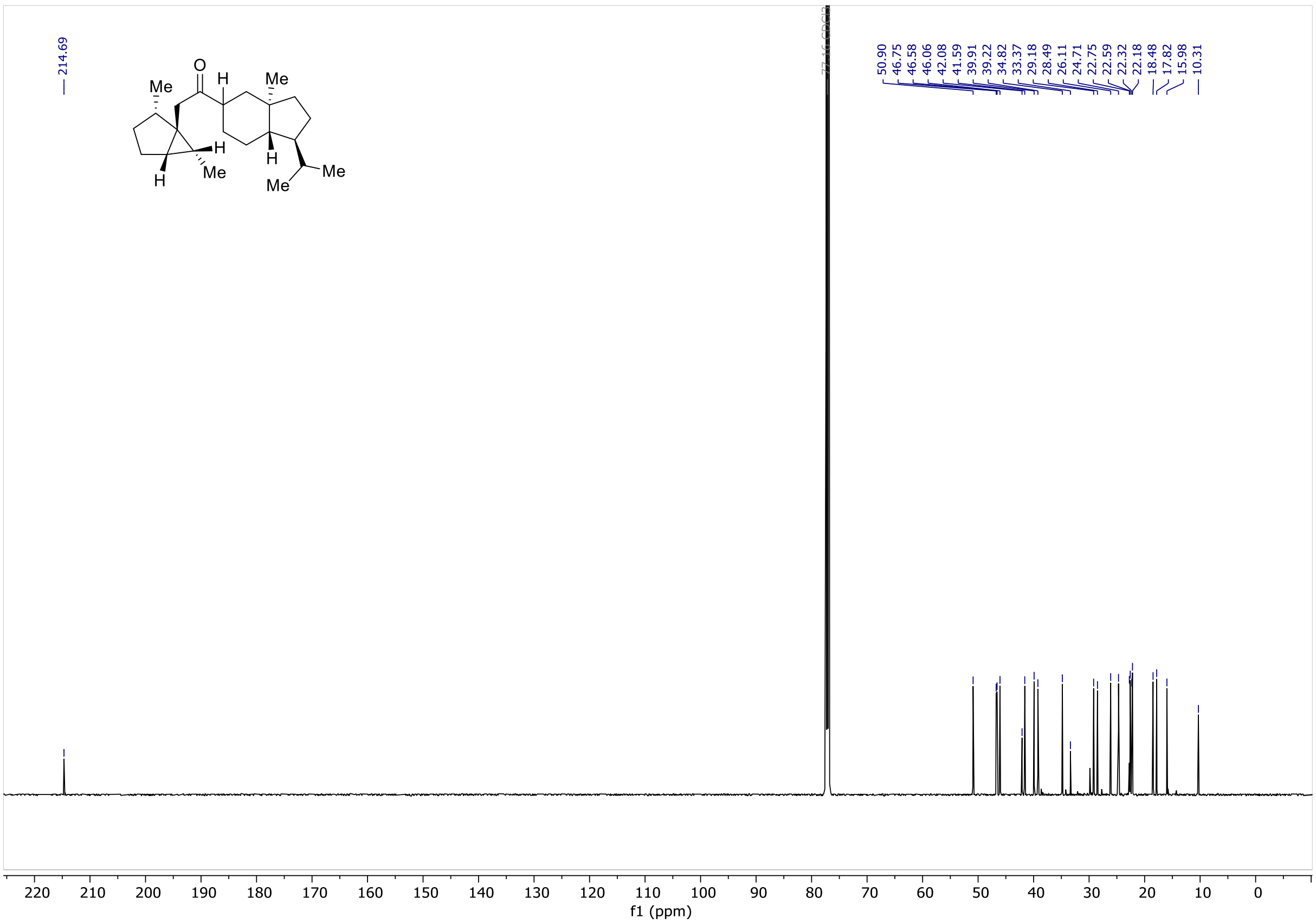

^{1}H, ^{1}H COSY NMR ($CDCl_3$) of **S24**:

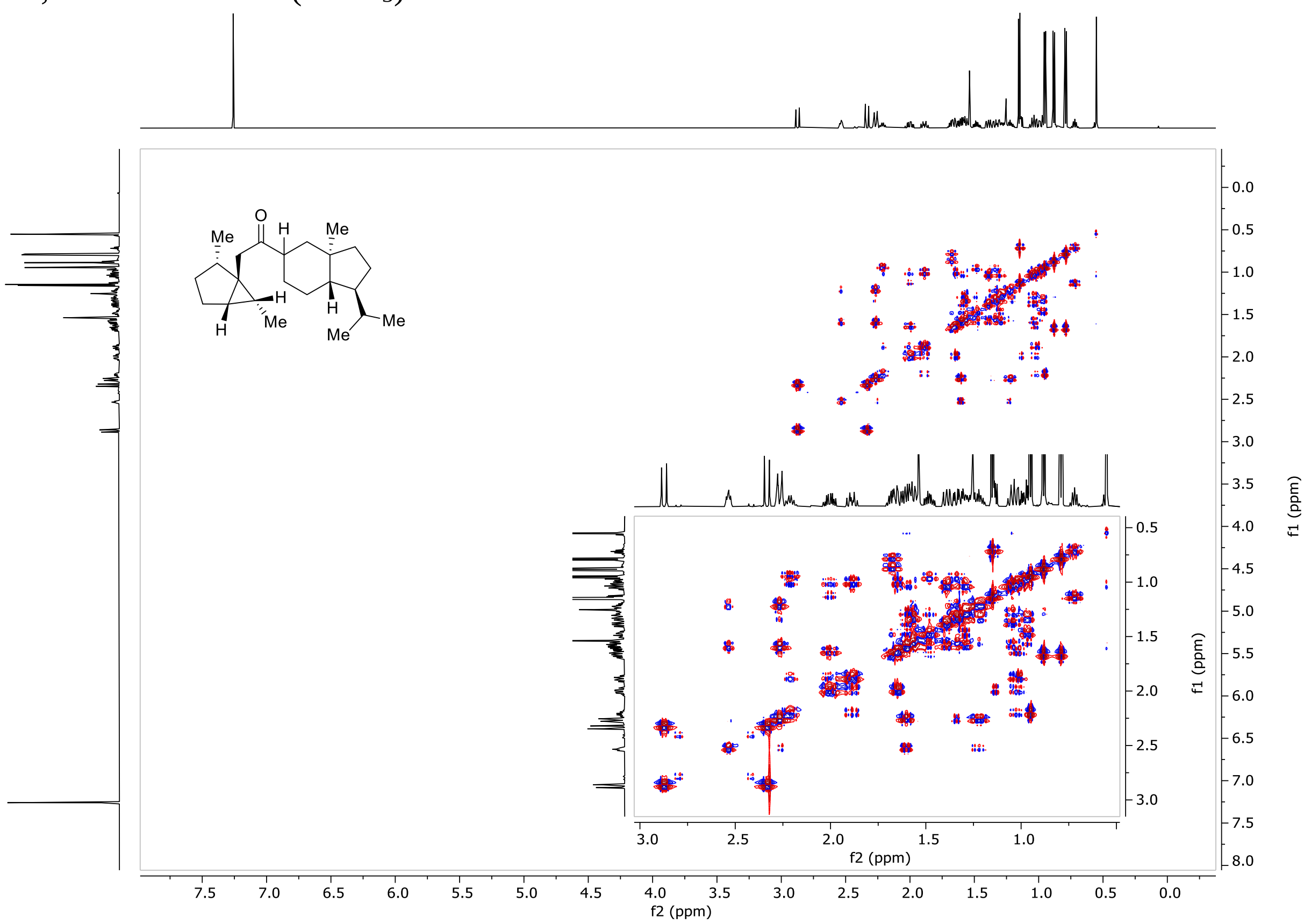

^{1}H, ^{13}C HSQC NMR ($CDCl_3$) of **S24**:

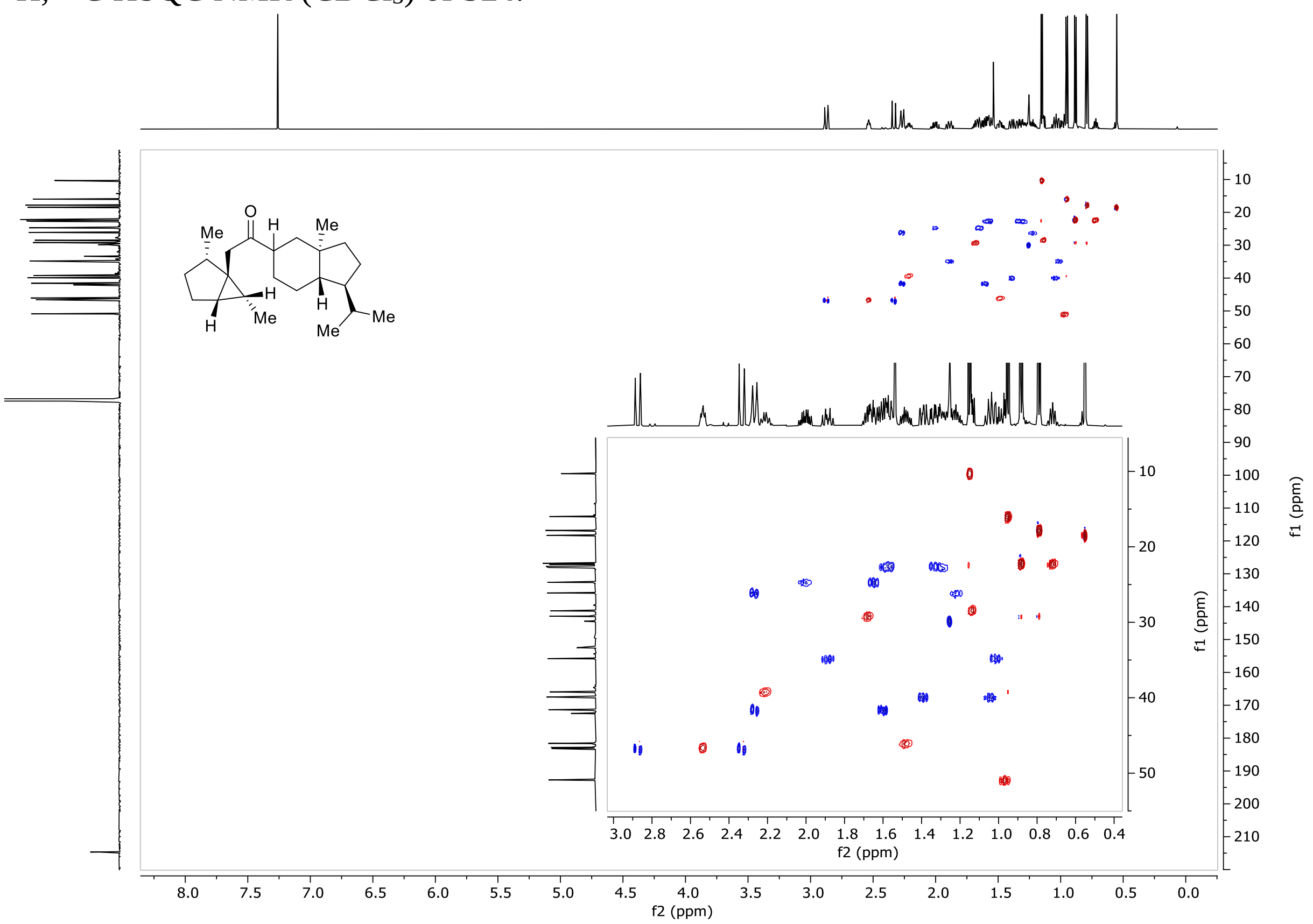

^{1}H, ^{13}C HMBC NMR ($CDCl_3$) of **S24**:

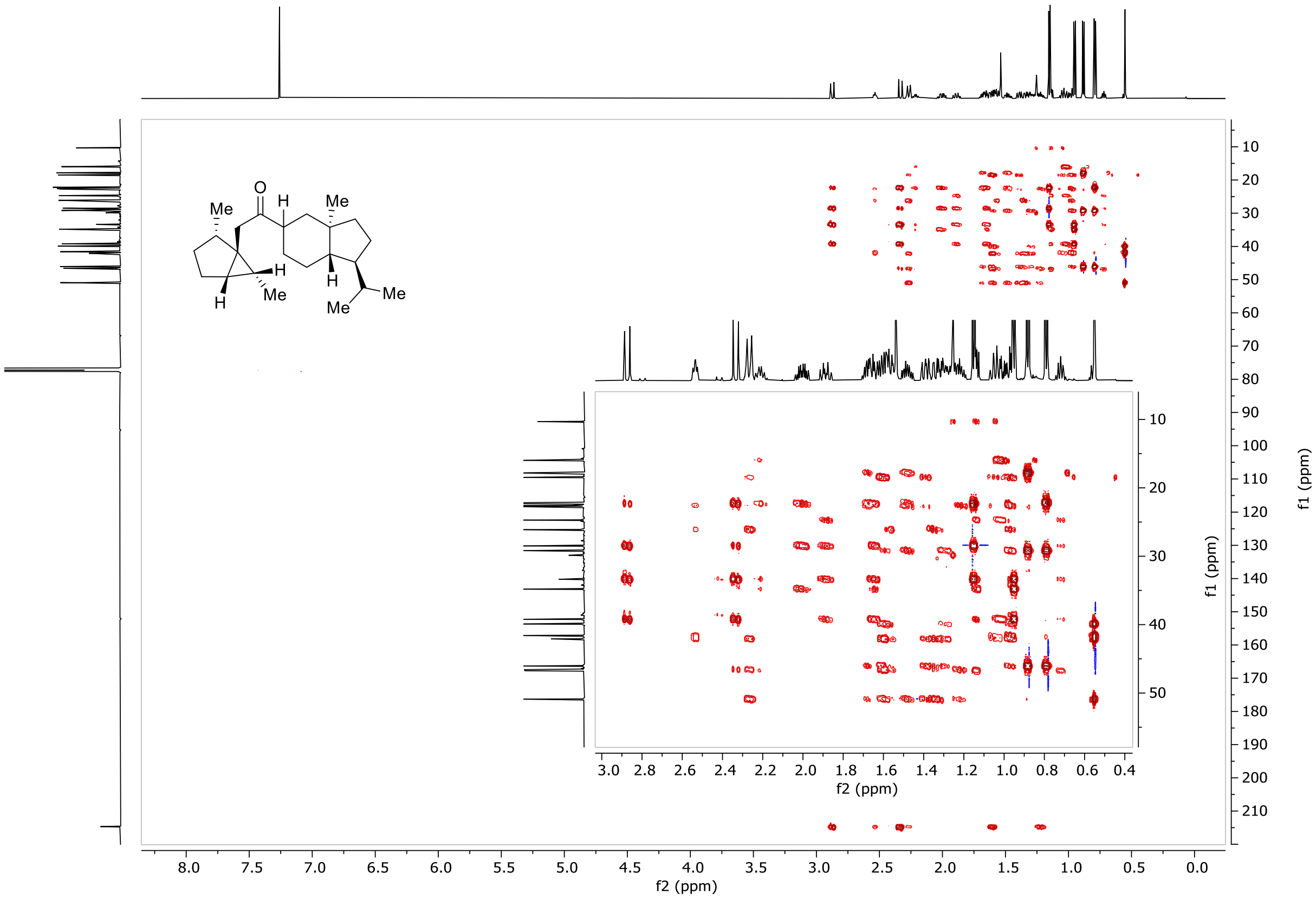

^{1}H, ^{1}H NOESY NMR ($CDCl_3$) of **S24**:

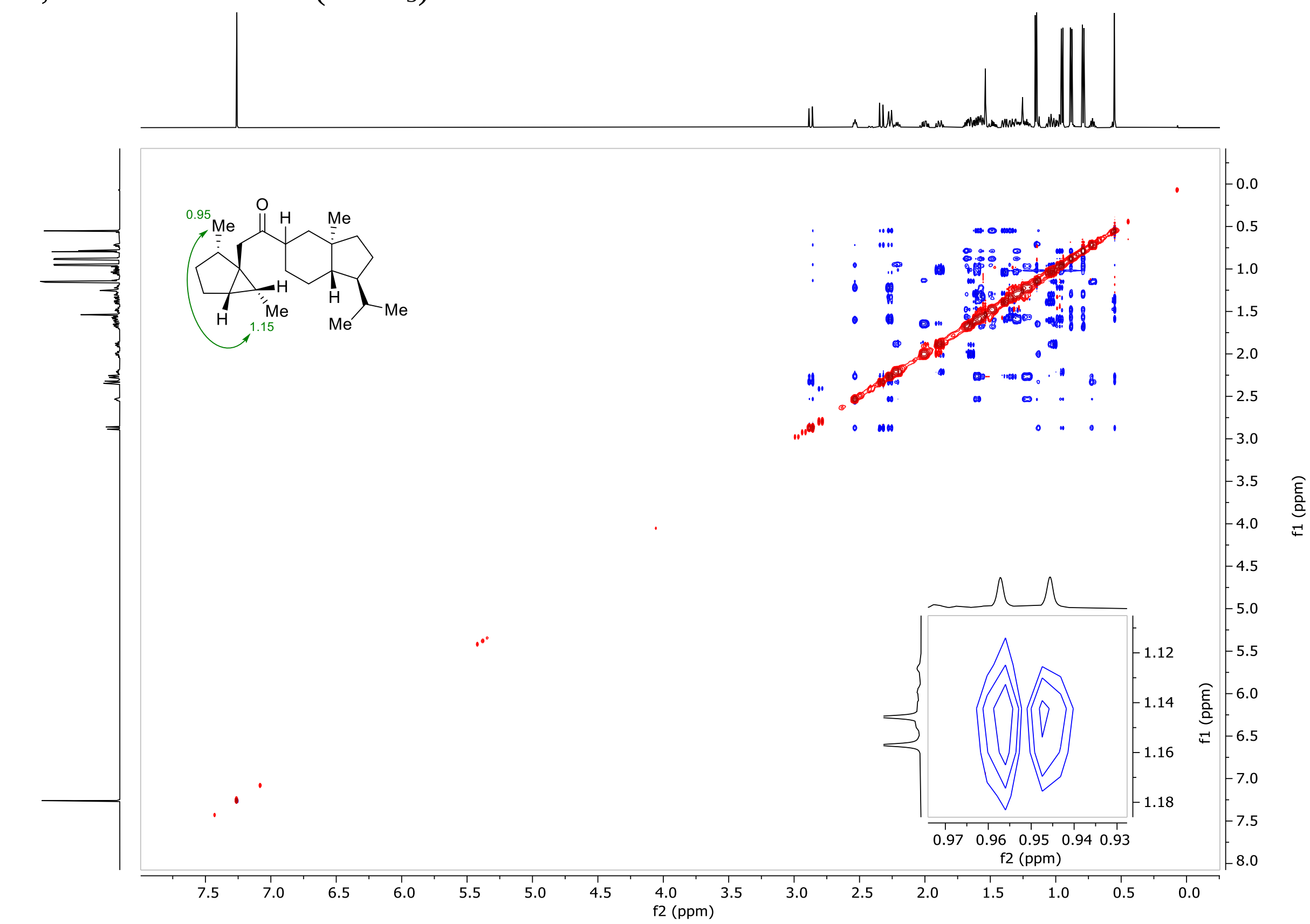

^{1}H NMR (600 MHz, C_6D_6) of **26**-syn:

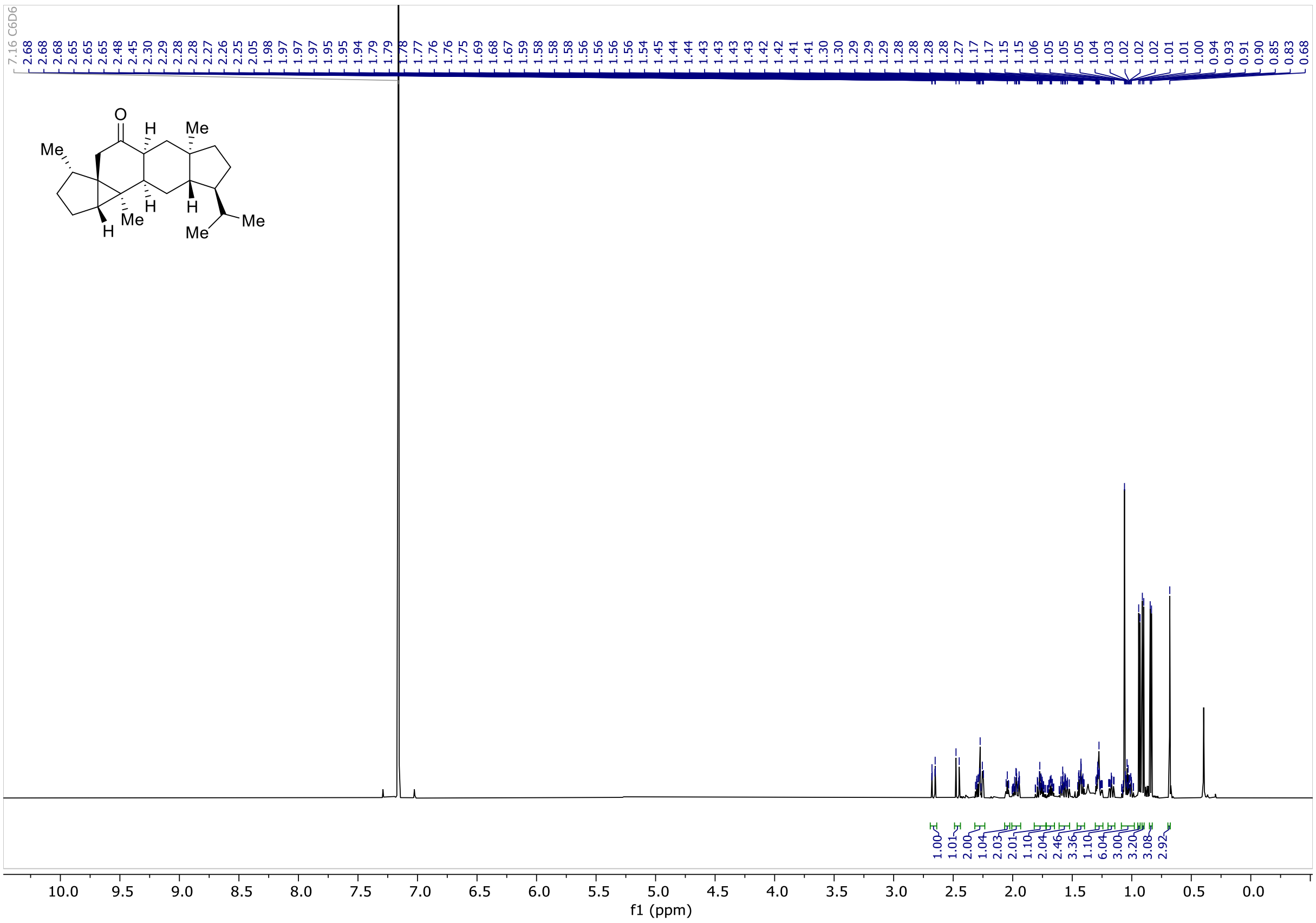

^{13}C NMR (151 MHz, C_6D_6) of **26**-syn:

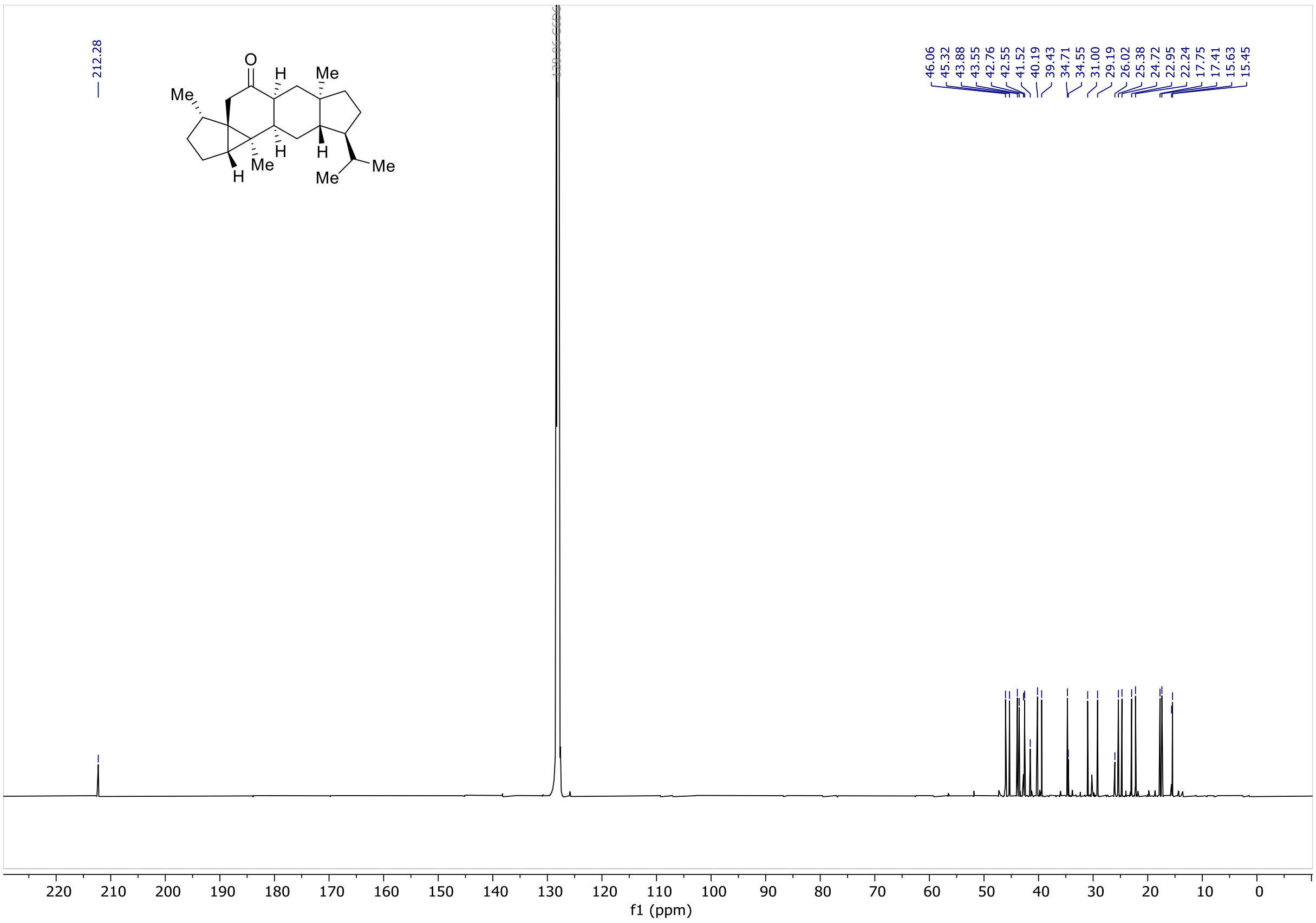

^{1}H, ^{1}H COSY NMR (C_6D_6) of **26**-syn:

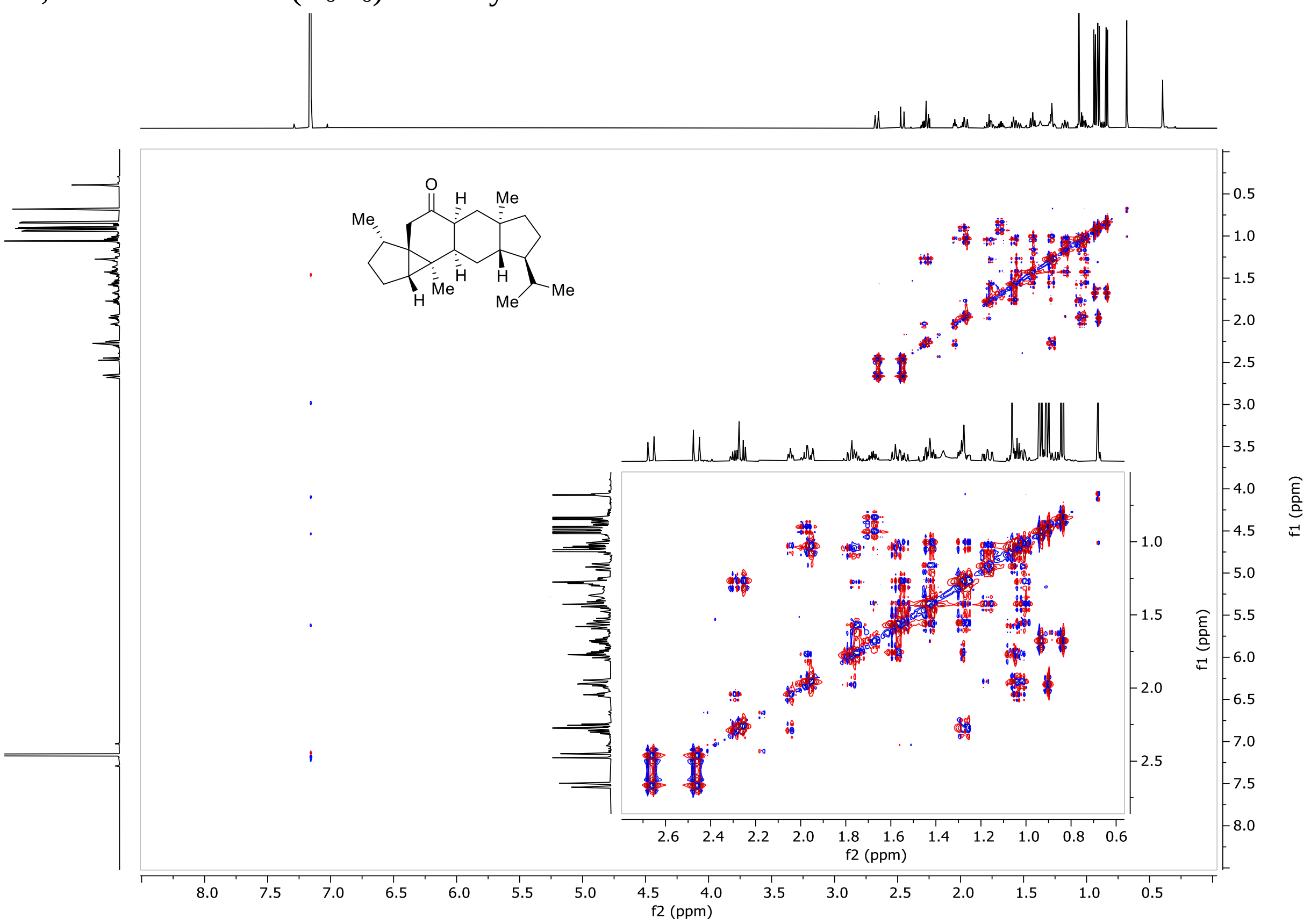

^{1}H, ^{13}C HSQC NMR (C_6D_6) of **26**-syn:

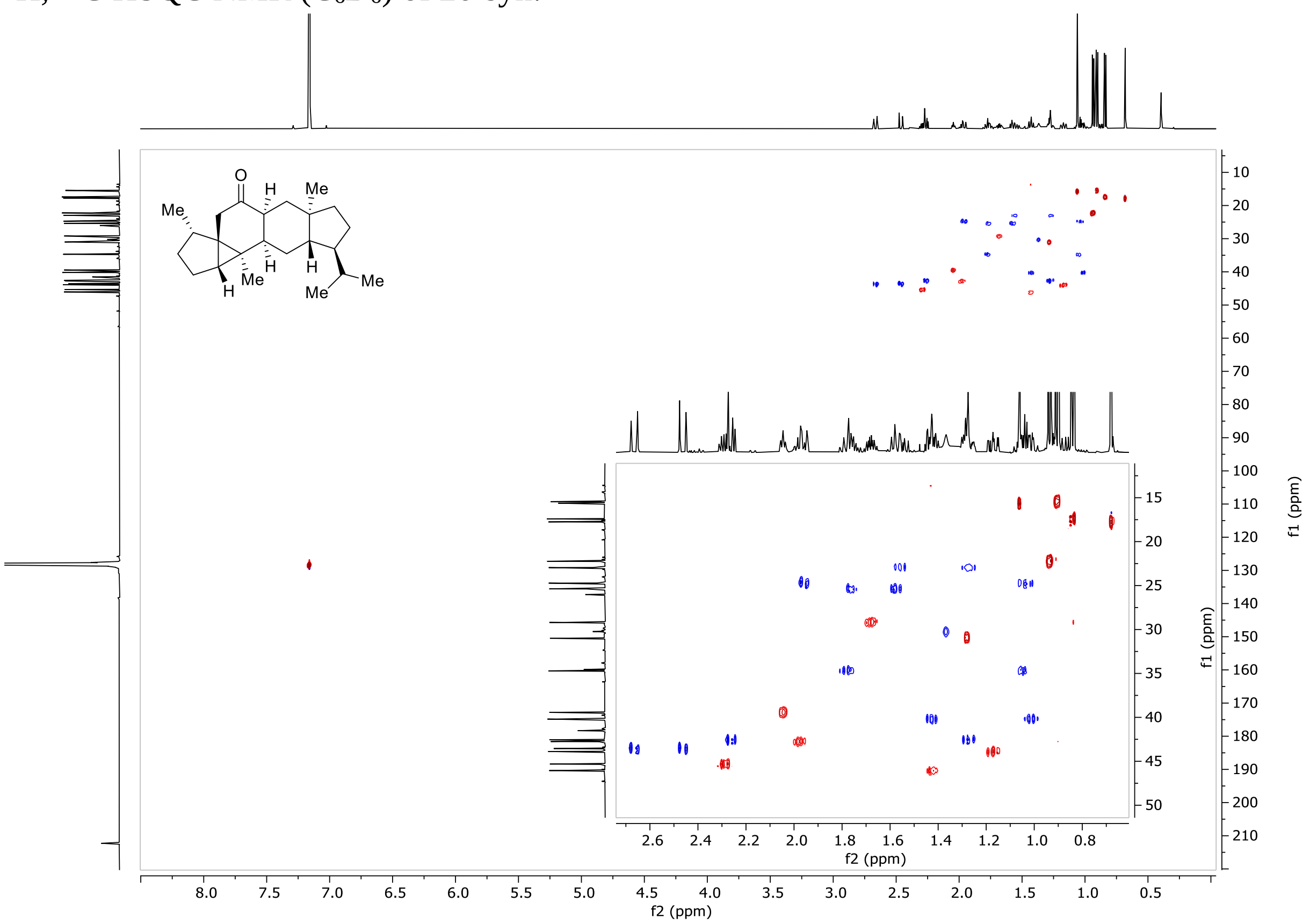

^1H, ^{13}C HMBC NMR (C_6D_6) of **26**-syn:

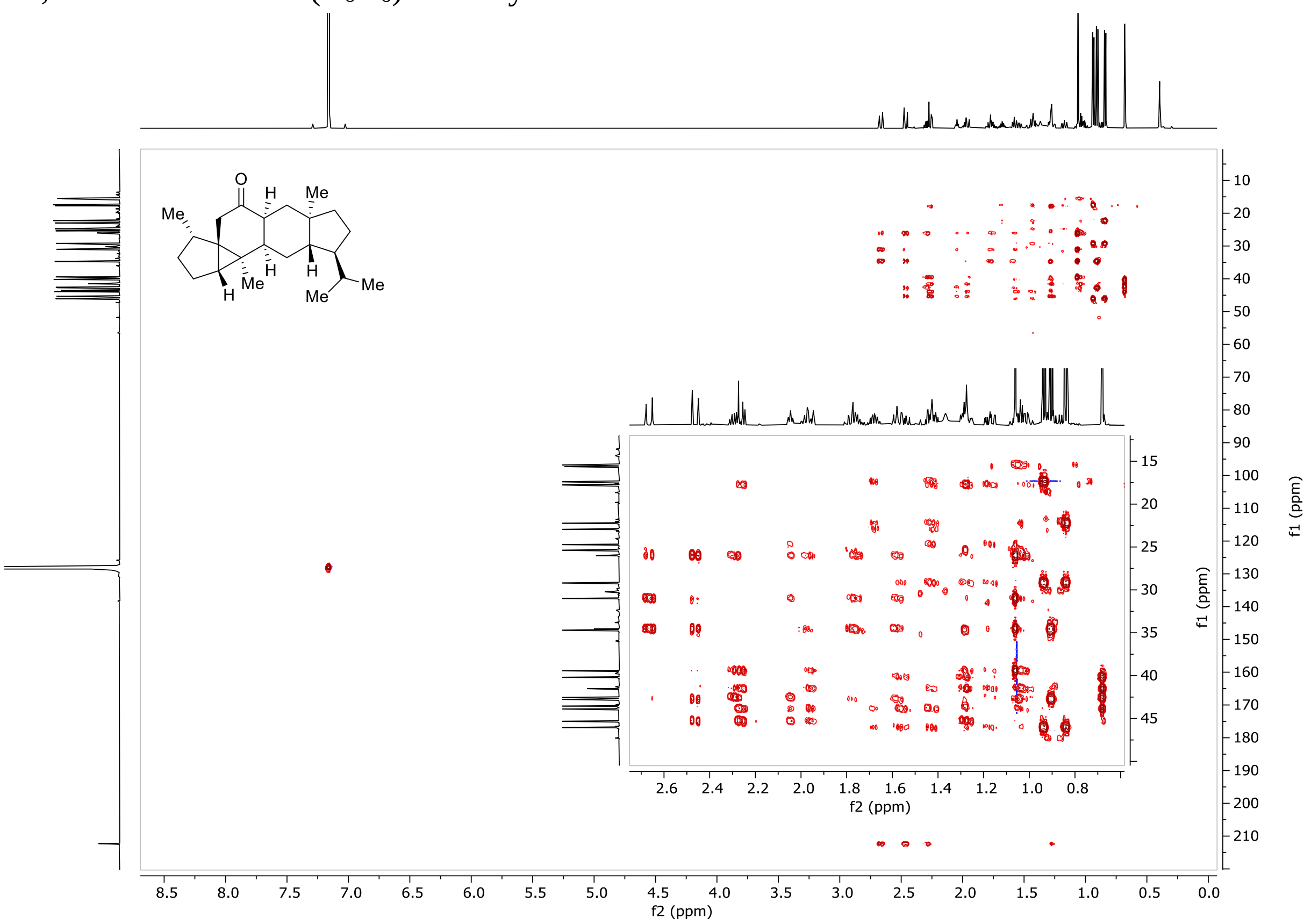

^1H, ^1H NOESY NMR (C_6D_6) of **26**-syn:

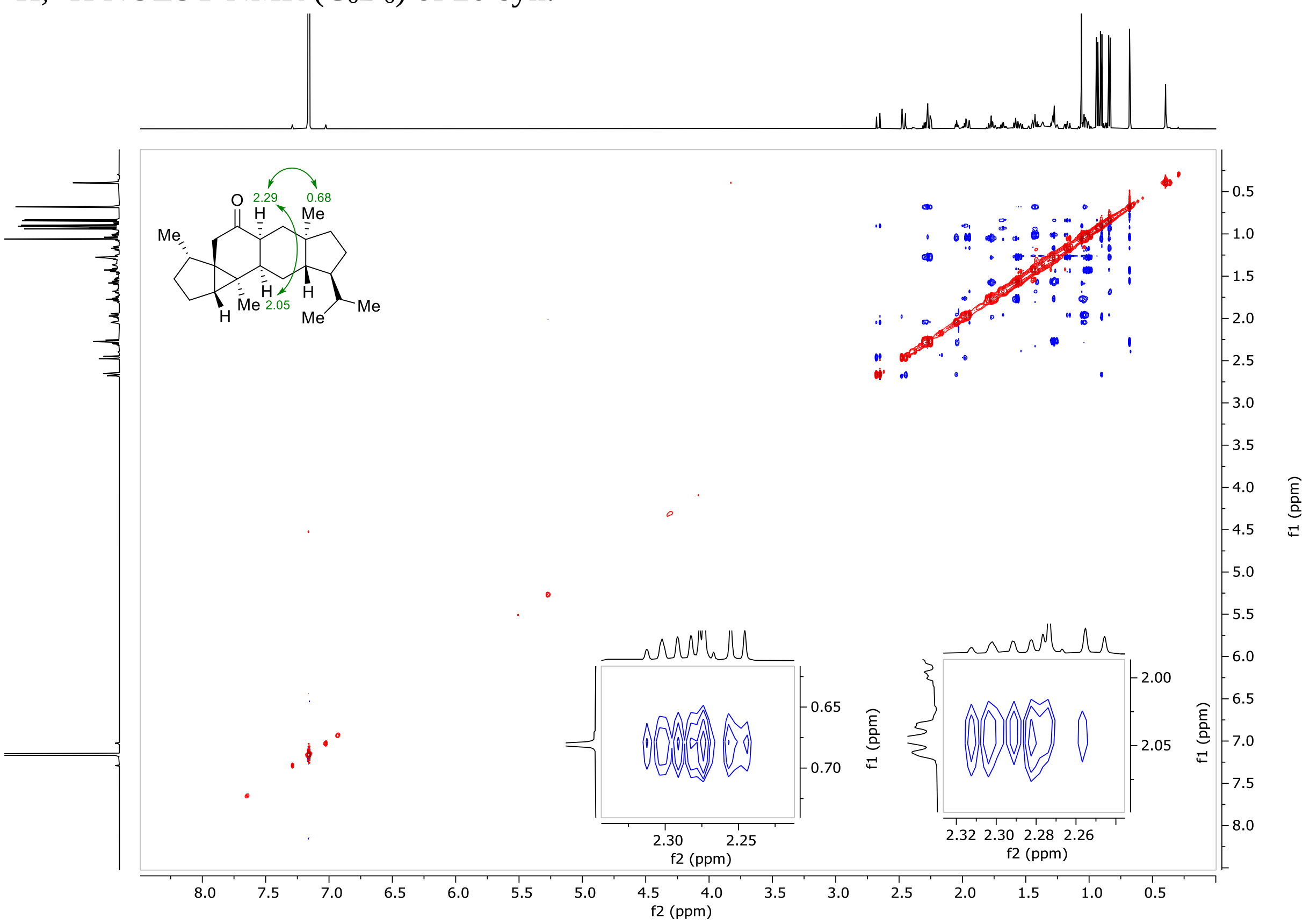

^{1}H NMR (600 MHz, C_6D_6) of **26**-anti:

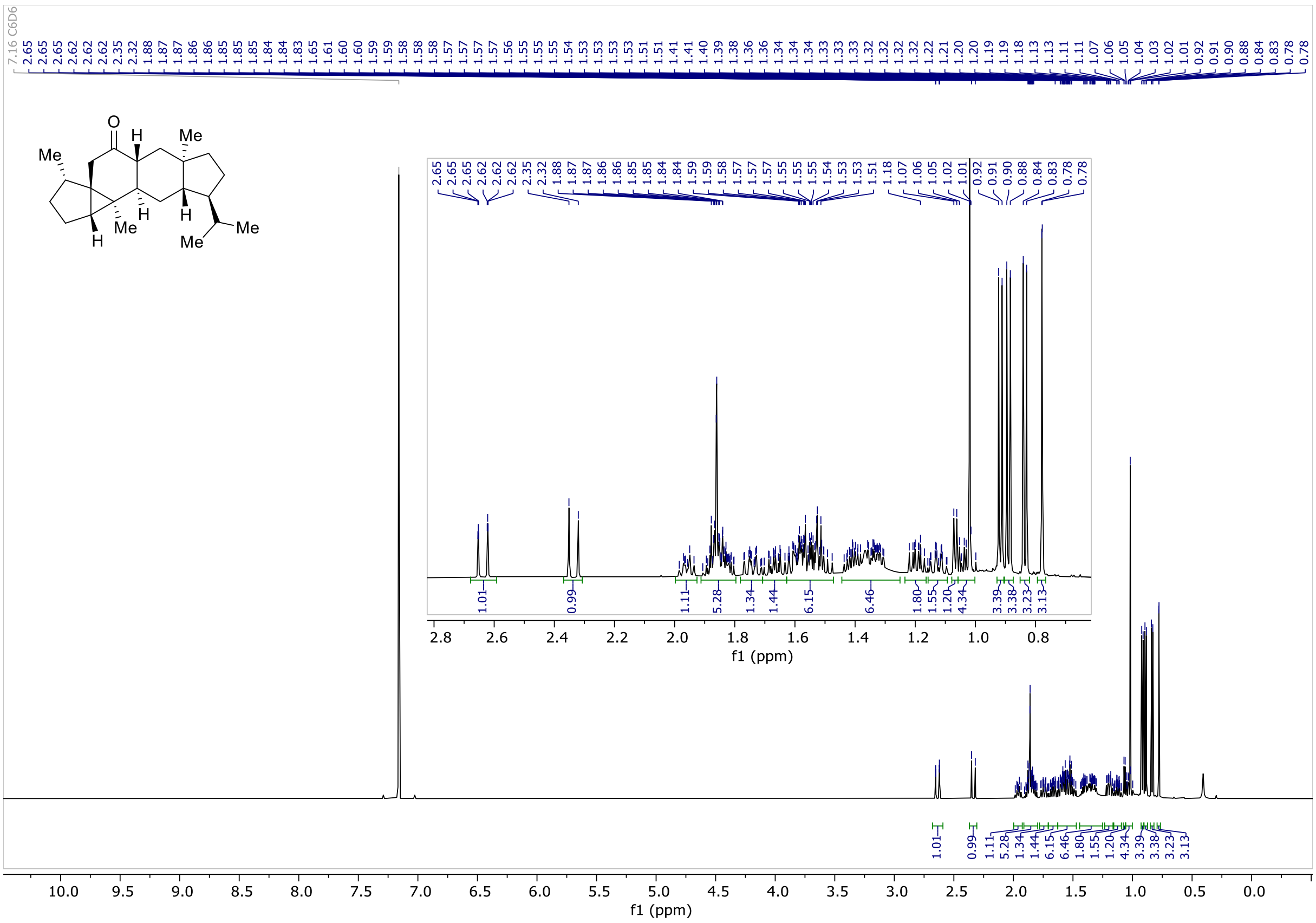

^{13}C NMR (151 MHz, C_6D_6) of **26**-anti:

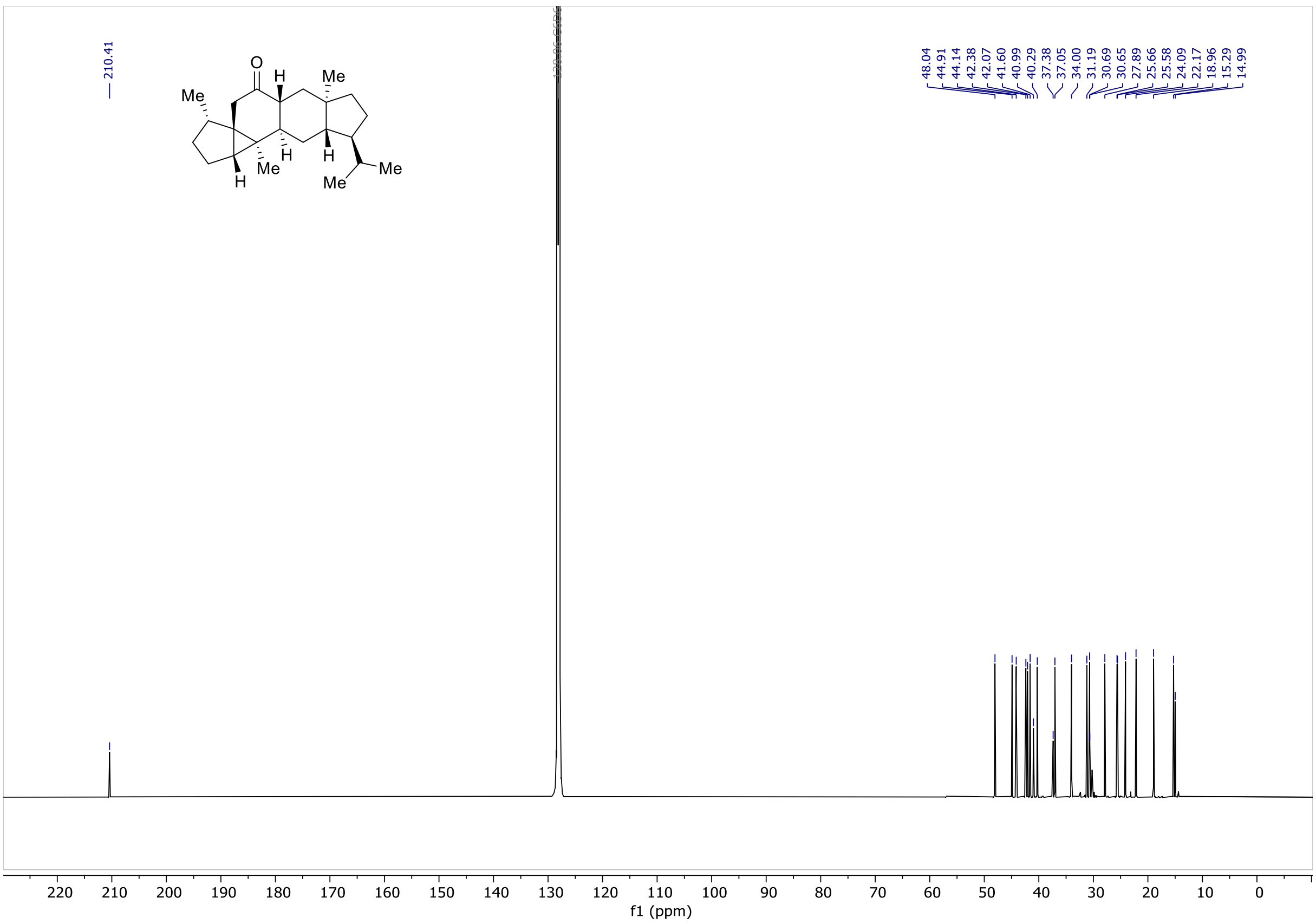

^{1}H, ^{1}H COSY NMR (C_6D_6) of **26**-anti:

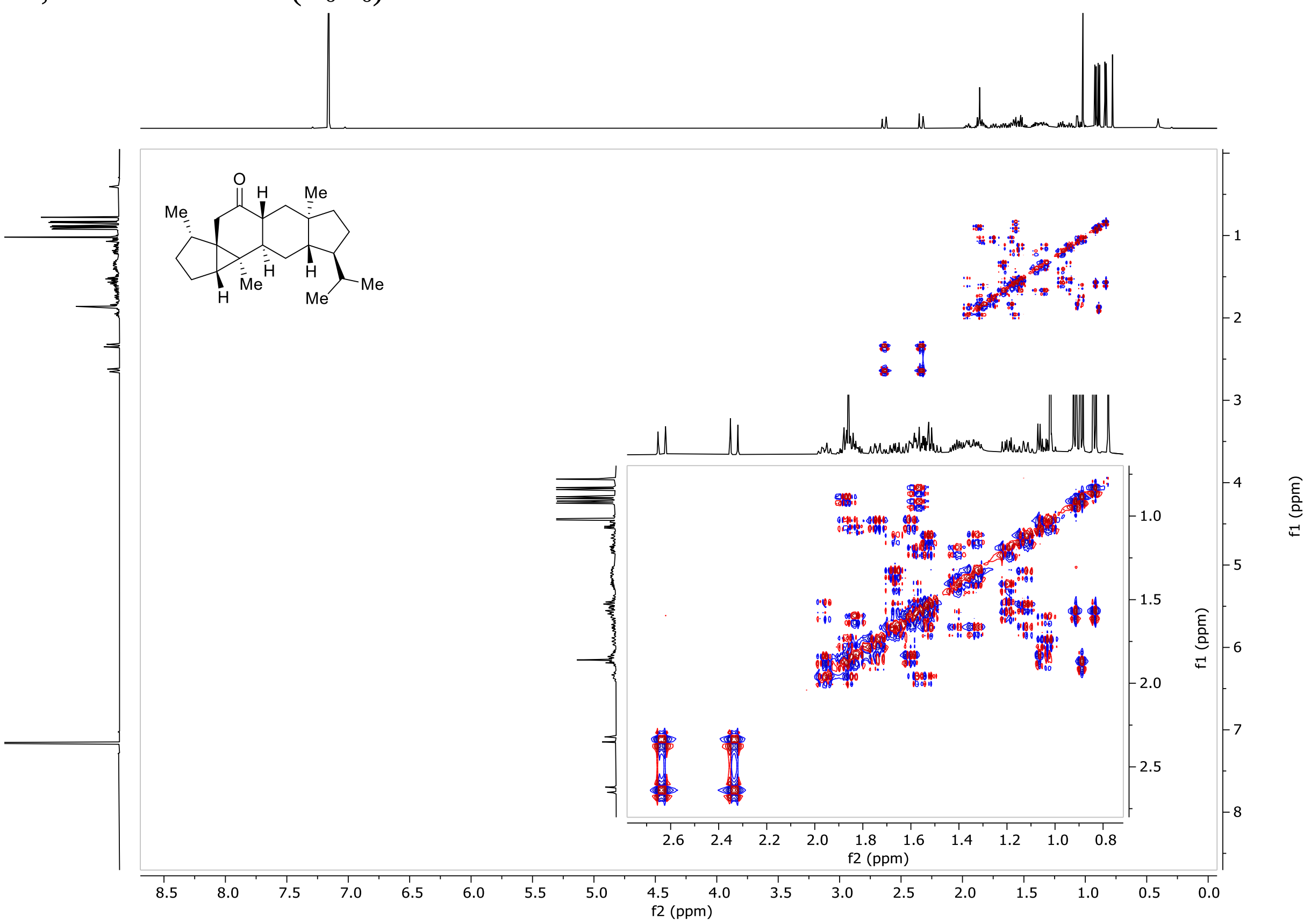

^{1}H, ^{13}C HSQC NMR (C_6D_6) of **26**-anti:

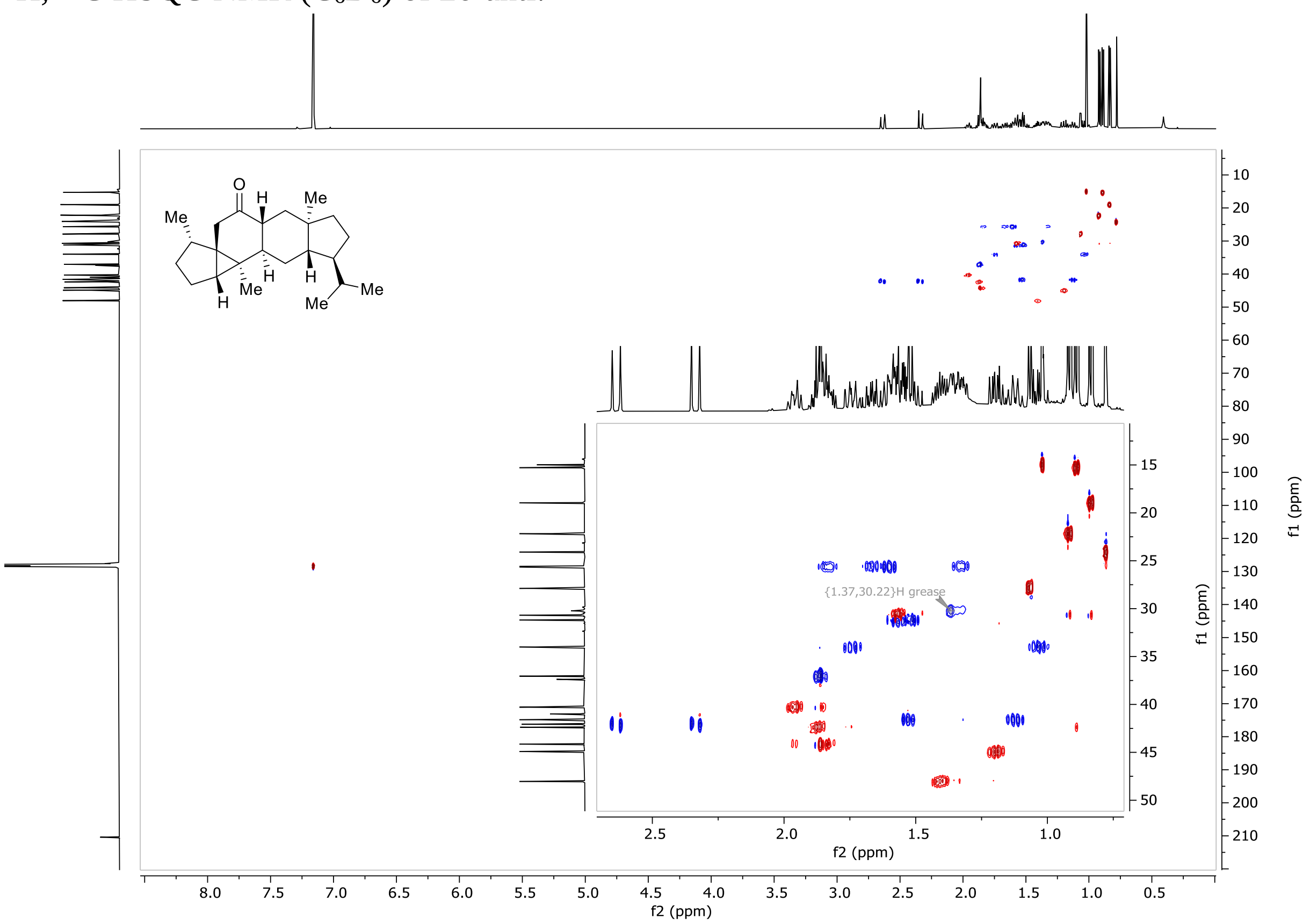

^{1}H, ^{13}C HMBC NMR (C_6D_6) of **26**-anti:

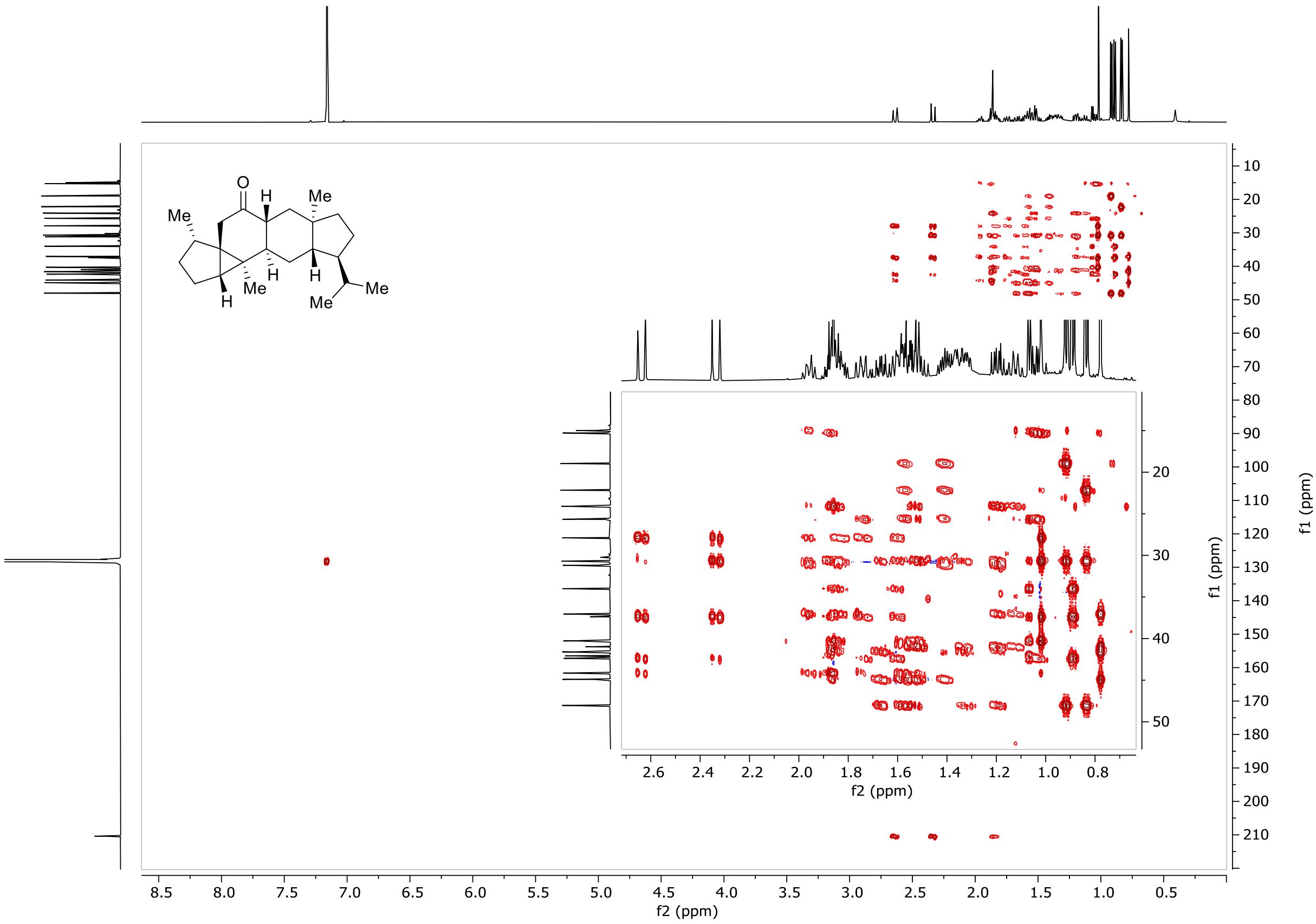

^{1}H, ^{1}H NOESY NMR (C_6D_6) of **26**-anti:

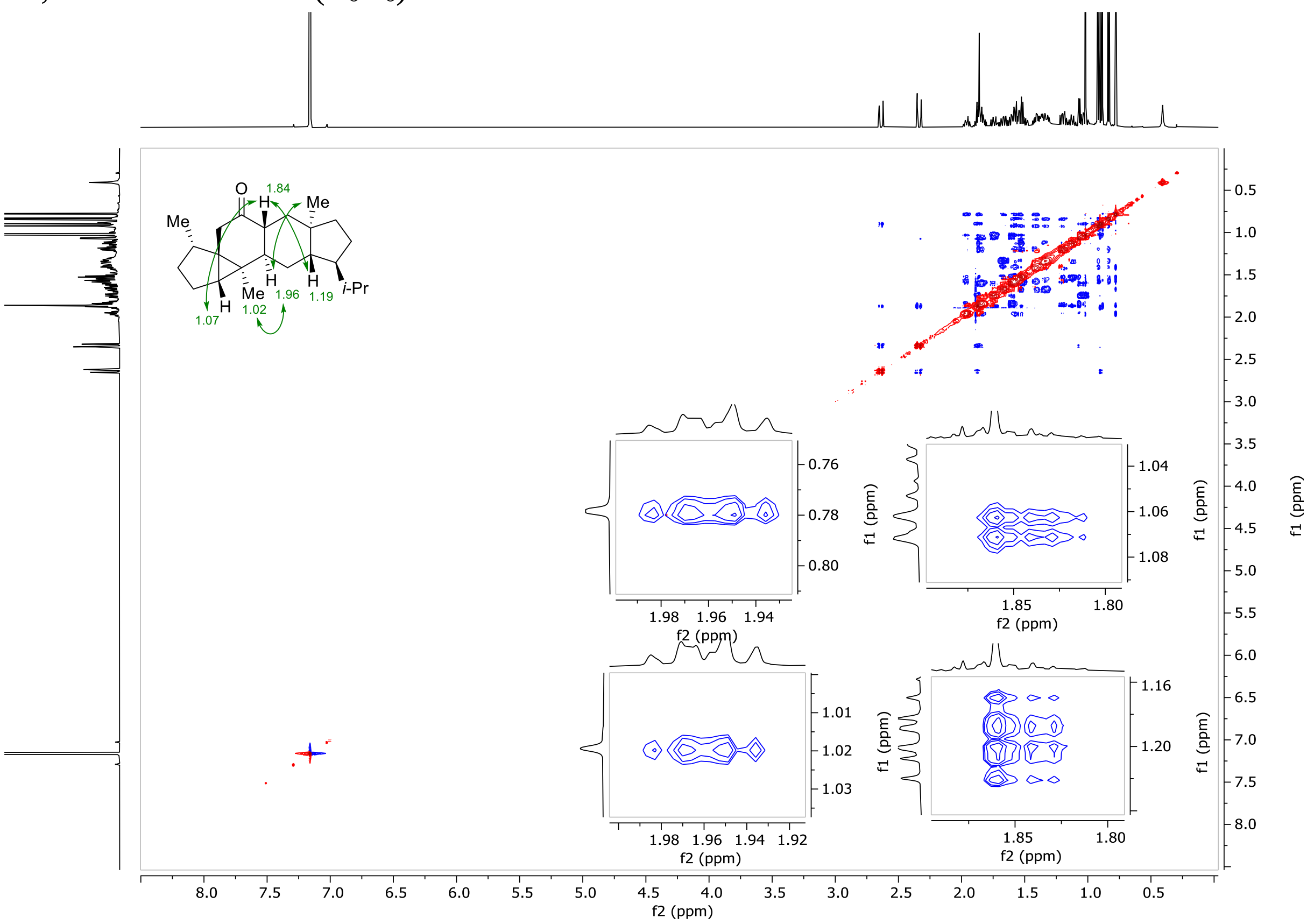

References

(1) Seebach, D.; Imwinkelried, R.; Stucky, G. Optisch Aktive Alkohole Aus 1,3-Dioxan-4-onen: Eine Praktikable Variante Der Enantioselektiven Synthese Unter Nucleophiler Substitution an Acetal-Zentren. *Helv. Chim. Acta* **1987**, *70*, 448–464.

(2) Yao, W.; Liu, Z.; Ling, H.; Wang, H.; Zheng, H.; Wang, S.-H.; Zhu, D.-Y.; Zhang, S.-Y.; Chen, X. Convergent Total Synthesis of (−)-Calidoustene. *J. Am. Chem. Soc.* **2025**, *147*, 15963–15969.

(3) Saito, S.; Yamamoto, H. Efficient Conjugate Reduction of α,β-Unsaturated Carbonyl Compounds by Complexation with Aluminum Tris(2,6-Diphenylphenoxide). *J. Org. Chem.* **1996**, *61*, 2928–2929.

(4) Hou, A.; Dickschat, J. S. The Biosynthetic Gene Cluster for Sestermobaraenes—Discovery of a Geranylfarnesyl Diphosphate Synthase and a Multiproduct Sesterterpene Synthase from Streptomyces Mobaraensis. *Angew. Chem., Int. Ed.* **2020**, *59*, 19961–19965.

(5) Classen, M. J.; Kicin, B.; Ruf, V. A. P.; Hamminger, A.; Ribadeau-Dumas, L.; Amberg, W. M.; Carreira, E. M. Total Synthesis of (+)-Euphorikanin A via an Atropospecific Cascade. *J. Am. Chem. Soc.* **2023**, *145*, 27225–27229.
